# Assessing prevalence, knowledge and use of cognitive enhancers among university students in the United Arab Emirates: A quantitative study

**DOI:** 10.1371/journal.pone.0262704

**Published:** 2022-01-26

**Authors:** Safia Sharif, Suzanne Fergus, Amira Guirguis, Nigel Smeeton, Fabrizio Schifano

**Affiliations:** 1 Psychopharmacology, Substance Misuse and Novel Psychoactive Substances Research Unit, School of Life and Medical Sciences, University of Hertfordshire, Hatfield, United Kingdom; 2 Institute of Life Sciences 2, Swansea University, Swansea, Wales, United Kingdom; 3 Centre for Research in Public Health and Community Care, School of Health and Social Work, University of Hertfordshire, Hatfield, United Kingdom; University of Bern: Universitat Bern, SWITZERLAND

## Abstract

**Background:**

Cognitive enhancers (CE) are often used to improve memory, alertness and cognitive capacity. These products are commercially and pharmaceutically available. Due to high academic pressure, university students are at risk of CE misuse. However, data regarding this issue are limited, especially in the United Arab Emirates (UAE).

**Aims:**

To assess the prevalence of CE intake; evaluate students’ knowledge of these substances; and identify student characteristics associated with CE usage.

**Method:**

A cross sectional study based on a validated online survey that was distributed using university-licensed software (Qualtrics) as a direct web link via email and social media to all Medical, Pharmacy, Dentistry, Nursing and Engineering students enrolled in six UAE universities. Associations between student characteristics and CE use were investigated using the chi-squared test and multiple logistic regression. Reasons for CE use, temporal patterns of use, details regarding purchase and types of CE used were compared by gender.

**Results:**

One quarter of students had used CEs. There was a clear difference between users and non-users in terms of gender (p<0.001). CE users were disproportionately represented by students from either UAE or other Arab countries (p<0.001), and by students of Medicine, followed by Pharmacy, Dentistry, and Engineering (p<0.001). CE use increased with year of study, reaching the highest level in the fourth year (p<0.001), which for most programmes is the final year. Modafinil was self-administered, especially in males, for concentration and alertness; B12 was typically taken by female students for academic performance and concentration; and high-dosage caffeine compounds were ingested to improve alertness levels. Use of the internet for both obtaining information and purchasing CEs was frequently reported. Multiple logistic regression analysis showed that gender, nationality, and year of study were associated with CE use among UAE university students.

**Conclusions:**

Universities need to address the prevalence of CE use amongst their students by providing effective support programs.

## Introduction

Most substances referred to as pharmacological cognitive enhancers were originally developed to treat neuropsychiatric disorders that are often accompanied by cognitive deficits [1]. These pharmacological cognitive enhancers (CEs), also known as study drugs and nootropics, are additionally used due to their alleged potential to improve memory, mental alertness, concentration and boost energy levels in healthy individuals, despite the reported drawbacks of its use such as dependency and the occurrence of psychiatric disorders [2, 3]. Cognitive enhancement is defined as an amplification or extension of core capacity of the mind by improving the internal and external information processing systems [4]. Substance use for cognitive enhancement has been reported by healthy university students to boost their academic performance [5].

A systematic literature review on an updated overview of 48 studies in various countries on the use of the most popular CEs prescription and non-prescription drugs among healthy university students has recently been published [6]. Prescription CEs including amphetamine salt mixtures, methylphenidate, modafinil and piracetam; and non-prescription CEs including caffeine, cobalamin (vitamin B12), guarana, pyridoxine (vitamin B6) and vinpocetine [6] were mentioned, although the most popular prescription CEs used among the university students were modafinil, methylphenidate and amphetamine salt mixtures [7]. Methylphenidate was the most popular prescription CE [8], and caffeine pills the most popular non-prescription CE [9]. However, the effectiveness of CEs among healthy participants and their efficacy is still unclear [2, 10].

The number of recent studies published concerning CE use indicates the rising interest in academic research, initially in the US and more recently in Europe, to understand the use of CEs by university students [11]. In the UK, a study showed that out of 1614 students, 33% of them had used prescription CEs that had not been prescribed for them [12]. From a sample of 877 students from UK and Irish universities, it was found that the lifetime prevalence of the use of modafinil, methylphenidate and amphetamine were, respectively, 6.2%, 5.9%, and 2% [11]. A meta-analysis from the US estimated that the misuse of CEs among university students was 17% [13]. In a study conducted in Brazil, out of 1865 students, 4.2% reported to having had used CEs in the last 12 months, with the most popular being methylphenidate [14]. Based on the review of the literature, only one study has been conducted in the UAE on the use of caffeine consumption as a CE among University students [15].

A survey involving around 60 countries showed that 20% of participants had already tried CEs; some 34% of subjects had purchased these from the internet, 14% from a pharmacy, and 52% from a physician [16]. The Global Drug Survey carried out in 2015 and 2017 among healthy university students reported on CE prescription drug use rates; these increased over time in all 15 countries for which data were analysed [17]. Main reported sources of supply for CEs included friends (47.8%); the web (11.8%); family members (6.1%); and physicians (3.8%) [17].

There may be levels of substantial CE use among students in high-ranking universities and highly competitive courses such as Medicine [18] and Pharmacy [19]. A study conducted in Saudi Arabia, a country that is geographically and socially similar to UAE, assessed the prevalence and motivation of illicit use of stimulants in 1177 medical students; some 29 (2.46%) were found to be using stimulants illicitly [20].

In UAE, the rates of both non-medical use of prescription stimulants and the use of legal/illegal drugs to enhance cognitive performance in healthy individuals are still not known. This study was designed to estimate the prevalence of CE use in six UAE undergraduate universities; describe the socio-demographics of CE-using students compared to non-users; identify factors associated with higher risk of CE use; and evaluate characteristics of the use of CEs within the UAE Higher Education institutions involved.

The main study hypotheses were as follows: (1) the prevalence of CE use in UAE universities is comparable to that reported elsewhere; (2) gender, age, year of study, study programs and nationalities are candidate variables/factors associated with CE use among the UAE university students; (3) Male and female students differ in their use of CEs.

## Material and methods

### Study design and setting

A cross-sectional survey-based study was conducted from March 2020 until November 2020. The period included the examination and typical end-of-term deadlines characteristic of all universities in the UAE.

### Study population

The target population for this study comprised undergraduate students at various university levels in the UAE.

The study was conducted in six institutions: Al-Ain university (AAU) which is located in the Southwest; Mohammed Bin Rashid University of Medicine and Health Science university (MBRU) in the Northeast; and four institutions located in the Northern Emirates of UAE (Ajman University (AU), University of Science and Technology (USTF), Ras Al Khaimah Medical and Health Sciences University (RAKMHSU) and University of Sharjah (UOS). These universities were selected based on their geographical location. Also, this study identified one Ministry of Higher and Scientific Research accredited university offering Dentistry, Medicine, Pharmacy, Engineering, and Nursing courses per state. All selected courses were highly competitive programmes requiring top grades from applicants for entry.

### Inclusion and exclusion criteria

All students registered in the selected undergraduate universities and the selected programmes aged 18 years or more were eligible for inclusion. Younger, postgraduate, and students registered on other courses were excluded.

#### Sample size determination

The sample size *n* required to estimate a prevalence *p* with a specified margin of error *e* was calculated using the formula as follows [21]:

$$n=z^{2}\;p(1‐p)/e^{2}$$ (1)

The value *p* = 0.5 was selected rather than the likely prevalence, as this gives the maximum possible requirement for a specified margin of error. In this study, the chosen *e* = 0.05 and z represents the value from a standard normal distribution corresponding to a 95% confidence level = 1.96, giving a requirement of *n* ≈ 385 participants. The target population of our study comprised 7760 eligible students registered on the medical, pharmacy, dental, engineering and nursing courses at the six universities during the academic year 2020/2021.

### Survey design and validation

The questionnaire was adapted from surveys available from the literature [2, 11, 22–26]. To validate the questionnaire, the questions were checked by the researcher followed by the supervisory team consistent with the ‘Face Validity’ approach [27]. Again, a UK Royal Pharmaceutical Society member expert in the field of quantitative surveys provided further advice and minor changes were made to improve the quality of the questions, in line with the ‘Content Validity’ [28] approach. The survey was piloted amongst 10 postgraduate students to ensure that questions were coherent, and further changes were implemented to improve the comprehension of some of the questions (‘Discriminant Validity’) [27].

The questionnaire was in English. While the first spoken language in UAE is Arabic, over the last decade as reported by the Ministry of Higher Education and Scientific Research (MOHESR), English has become a commonly spoken language, taught at all Higher Education and Research Institutions [29]. Based on the findings from a recent systematic review [6], 9 drugs/substances (modafinil, amphetamines mixtures, methylphenidate, pyridoxine/vitamin B6, super strength caffeine pills, guarana, piracetam, vinpocetine, cobalamin/vitamin B12) were included in the study. These drugs/substances have proved to be most popular among university students and frequently mentioned on the web as well [30].

Due to better ease of access [31]; the current COVID pandemic; and the administrative difficulties of paper surveys, use of an online tool was here considered the best option. The online questionnaire consisted of 17 questions covering a range of topics concerning substances used to enhance cognition for study/academic/work purposes. It included questions on personal data such as gender, age group, nationality, programme of study and year of course for information gathering purposes whilst ensuring full anonymity. The possible characteristics of CE use was tested by a range of questions (see S1 Appendix), which included the name/s of drug used, reasons for use, frequency and duration of consumption, source of drugs/substances, cost, if the users achieved the required benefits; and if they recommended using these molecules. To encourage participation in what could be seen as a sensitive area of research, CE non-users could record just their gender and age group. Non-CE users who provided information on nationality, programme of study and year of course were here referred to as ‘non-user reporters’.

### Ethical approval and study procedures

The study received full Ethical approval from the University of Hertfordshire UH (UK) [LMS/PGR/UH/04025], RAK Medical and Health Sciences RAKMHSU (UAE) [RAKMHSU-REC-178-2020-PG-P], and the Ministry of Health and Prevention Research Ethics Committee RAK Subcommittee (UAE) [MOHAPIREC/2020/35-2020-PG-P).

Participation was voluntary and anonymous, in compliance with the General Data Protection Regulatory (GDPR) requirements [32]. Prior to participation in the study, all potential participants were informed about the aims of the study and their rights to refuse participation or withdraw from the study at any stage without any consequences. Each student gave their consent before participating in the study.

The Dean of each student union received a telephone call from the researcher and an email was sent to all universities selected for the study requesting their permission to run the study in their academic institutions following the approval of the Ethical Boards.

The questionnaire was distributed using university-licensed software (Qualtrics) and posted as a direct web link to students on emails concerning their chosen course. Students were emailed directly with a short explanation (consent), that included a title and the aims of the study; it also provided the student with a link to complete the survey online. Participants who completed the survey were entered into a draw for a £100 internet purchase voucher. The questionnaire took on average about 5 minutes to complete. To facilitate the process and increase the ease of participation, survey access was made possible through the smartphone as well; an email reminder was sent to the students once a week for three months to increase the response rate.

### Data analysis

Files were kept on a password-protected laptop used by the researcher only, with scanned versions of study data having been kept on an encrypted folder until the completion of the project.

Data collected from the survey were automatically generated on an Excel sheet. All data were checked for incomplete access and rogue entries, and questionnaires providing meaningful information were retained. Data were then coded for analysis. Statistical analyses were carried out by using IBM SPSS version 26.0 software package. The number of participants with missing data due to not reporting or incomplete survey entries was recorded for each variable and indicated in the tables. For each statistical analysis, participants with missing values were excluded.

The reliability and validity of the survey questionnaire were assessed using Cronbach’s alpha [33]. Variables were summarised by frequencies and percentages. The chi-squared test was used to compare users and non-users of CE with regard to gender and age-group (Fisher’s exact test [34] was used when cell entries were small, i.e., more than 20% of cells had an expected value of less than 5) and users with non CE users reporters on nationality, programme of study and year of study. The significance level was taken to be 0.05. Univariate analyses were used to select variables for inclusion in the subsequent multiple logistic regression analyses. Subject to both gender and age group giving a p value of less than 0.25 in univariate analyses [35], users and non-users of CEs were compared by multiple logistic regression with gender and age group as explanatory variables. Independence of observations was assumed as the students who participated studied at universities with different geographical locations. Lockdown and distance teaching meant that students answered the questionnaire online. Spending most of the time physically apart from their fellow students, they were more likely to give independent responses.

As an exploratory exercise, CE users were compared with the CE non-user reporters first by simple logistic regression then by multiple logistic regression using gender, age group, nationality, programme of study and year of study as explanatory variables. To check for potential bias, non-user reporters were compared with the remaining non-users on gender and age group.

For CE users only, demographics and characteristics of CE use were compared by gender with the chi-squared test or Fisher’s exact test using the same criteria as described above (i.e., Fisher’s exact test [34] chosen when expected cell entries were small).

## Results

In total, 560 completed questionnaires were received, exceeding the minimum sample size required for this study. Following checks for incomplete access and spam entries, 38 of these were removed. Hence, 522 (7%) of the eligible students completed the questionnaire correctly and participated (S1 Fig)

The survey questionnaire was found to be highly consistent and indicated high reliability (Cronbach’s alpha = 0.903). Table 1 summarises the demographic data for all participants. Around 60% of the respondents were female, and nearly all (94.6%) were from the 18–25-year-old age group. The prevalence of CE usage in the total sample of study was 25.3% (132 out of 522 students). Of the 390 non-users, 109 (27.9%) reported their nationality, 71 (18.2%) reported their programme of study and 101 (25.9%) reported their year of study, with 109 non-users reporting at least one of these items.

**Table 1:** Demographic characteristics of respondents from the UAE Universities.

Number		522
**Gender**	Female	307 (59.5%)
	Male	209 (40.5%)
	(Missing)	6
**Age**	18–25 years	494 (94.6%)
	26–35 years	15 (2.9%)
	above 35	13 (2.5%)
**CE consumer**	No	390 (74.7%)
	Yes	132 (25.3%)
**Nationality**	UK	14 (5.8%)
	EU	1 (0.4%)
	USA	2 (0.8%)
	UAE	69 (28.8%)
	Arab countries	73 (30.4%)
	Asian countries	62 (25.8%)
	Other	19 (7.9%)
	(Missing)	282
**Degree**	Medicine	104 (51.2%)
	Pharmacy	38 (18.7%)
	Dentistry	33 (16.3%)
	Nursing	9 (4.4%)
	Engineering	19 (9.4%)
	(Missing)	319
**Year of Study**	First	37 (15.7%)
	Second	40 (17.0%)
	Third	67 (28.4%)
	Fourth	71 (30.1%)
	Fifth	19 (8.1%)
	Sixth	2 (0.9%)
	(Missing)	286

Data are presented as frequencies (percentage of the non-missing responses from the UAE universities), missing values are shown for comprehensive reporting purpose only. Percentages may not sum to 100 due to rounding.

The distribution of nationality for the CE users was similar to that for the population of students on these programmes as a whole, with UAE nationals and Asians representing the majority of the population.

Tables 2 and 3 present a comparison of CE users with either non-users (2) or non-user reporters (3). There were significant differences between users and non-users regarding gender (p<0.001) but not on age group (p = 0.875). With only one significant univariate result relating to all non-users, it was not necessary to perform multiple logistic regression on the complete sample of students.

**Table 2:** UAE students; demographics of CEs users vs non-users.

Number		users 132	non-users 390	p value
**Gender**	Female	40 (30.5%)	267 (69.4%)	p<0.001 *
	Male (Missing)	91 (69.5%) 1	118 (30.6%) 5	
**Age**	18–25	126 (95.5%)	368 (94.4%)	p = 0.875
	26–35	3 (2.3%)	12 (3.1%)	
	above 35	3 (2.3%)	10 (2.9%)	

* Significant difference between CE users and non-users.

**Table 3:** UAE students; demographics of CEs users vs non-user reporters.

**Number Nationality#**	**UK**	**users 132 8 (6.1%)**	**non-user reporters 109 6 (5.5%)**	**p<0.001** **
	EU	1 (0.8%)	0	
	USA	1 (0.8%)	1 (0.9%)	
	UAE	55 (42%)	14 (12.8%)	
	Arab countries	49 (37.4%)	24 (22%)	
	Asian countries	16 (12.2%)	46 (42.2%)	
	Other (Missing)	1 (0.8%) 1	18 (16.5%) not applicable	
**Degree**	Medicine	64 (50%)	36(50.7%)	p<0.001 **
	Pharmacy	24 (18.8%)	14 (19.7%)	
	Dentistry	19 (14.8%)	14 (19.7%)	
	Nursing	2 (1.6%)	7 (9.9%)	
	Engineering (Missing)	19 (14.8%) 4	0 38	
**Year of Study**	First	9 (6.9%)	27 (26.7%)	p<0.001 **
	Second	16 (12.1%)	24 (23.8%)	
	Third	39 (29.5%)	27 (26.7%)	
	Fourth	53 (40.2%)	17 (16.8%)	
	Fifth	12 (1.5%)	6 (5.9%)	
	Sixth (Missing)	2 (1.5%) 1	0 8	

** Significant difference between CE users v. non-user reporters.

# Fisher’s exact test.

Percentages may not sum to 100 due to rounding.

CE users were disproportionately represented by students from either UAE or other Arab countries (p<0.001), and by students of Medicine, followed by Pharmacy, Dentistry, and Engineering (p<0.001). CE use increased in line with year of study, reaching the highest level in the fourth year (p<0.001), which for most programmes is the final year.

In the assessment of potential bias, CE non-user reporters were comparable to remaining non-users in terms of age group: 101/109 (92.7%) vs 267/281 (95.0%) aged 18–25 years. However, non-user reporters differed somewhat from the remaining non-users on gender: 88/109 (80.7%) vs 179/276 (64.9%) female, respectively. Working with the assumption that CE non-user reporters were broadly representative of all CE non-users, simple logistic regression followed by multiple logistic regression for CE use was applied to the CE users and CE non-user reporters with gender, age group (18–25 vs above 25), nationality (UK, EU and USA vs UAE and Arab vs Asian), programme of study (Medicine v. others) and year of study (first two years vs final years) as explanatory variables.

The simple logistic regression analyses showed that students who were males; 18-25-year-old; from Emirati/remaining Arab origins; studying Medicine; and in the final years of their study programmes presented with increased odds of using CEs. Students of Asian nationality had reduced odds of using CEs. Based on the p value threshold of 0.25 for entry into the multiple logistic regression, only gender, nationality, and year of study were selected.

The multiple logistic regression analysis showed that for university students in the UAE, male gender and being in the final years of study were independently associated with a higher risk of CE use. There was an indication of higher CE use with students of Emirati/ remaining Arab origin whereas being of Asian nationality was associated with a reduced risk, although these findings did not reach statistical significance (Table 4).

**Table 4:** Logistic regression analysis for CE use among university students in the UAE.

Variables	Simple regression analysis		Multiple regression analysis	
	Crude OR^a^ (95% CI)	p value	Adjusted OR^b^ (95%CI)	p value
**Gender**				
**(baseline: Female)**				
**Male**	5.15 (3.35–7.92)	<0.001	6.91 (3.440–13.91)	<0.001
**Age**				
**(baseline: Above 25 years)**				
**18–25 years**	1.26 (0.50–3.20)	0.630		
**Nationality**				
**(baseline: UK, EU and USA)**				
**UAE and Arab**	1.19 (0.50–2.90)	0.707	2.68 (0.95–7.51)	0.062
**Asian**	0.16 (0.10–0.30)	0.001	0.43 (0.17–1.08)	0.073
**Programme of Study**				
**(baseline: Other programs)**				
**Medicine**	0.988 (0.55–0.1.77)	0.967		
**Year of study**				
**(baseline: First two years)**				
**Final years (3–5)**	4.28 (2.39–7.69)	<0.001	2.40 (1.20–4.80)	0.001

^a^: Simple logistic regression

^b^: Multiple logistic regression.

The model reasonably fits well, assumptions met and no interaction or multicollinearity problems.

For CE female users, the proportion studying Dentistry exceeded that for males whereas the reverse was true for Engineering students (p = 0.012 for differences in degrees’ distribution; Table 5). The distribution for the year of study did not differ significantly between males and females.

**Table 5:** Gender-based demographic distribution of CEs’ users among the UAE students.

		Female (40)	Male (91)	p value*
**Age#**	18–25 years	36 (90%)	89 (97.8%)	p = 0.053
	26–35 years	3 (7.5%)	0	
	above 35 years	1 (2.5%)	2 (2.2%)	
**CE usage**	Multiple CEs	16 (43.2%)	48 (53.3%)	p = 0.301
	Single CE	21 (56.8%)	42 (46.7%)	
**Nationality#**	UK	7 (17.5%)	1 (1.1%)	p = 0.076
	EU	0	1 (1.1%)	
	USA	1 (1.1%)	0	
	UAE	12 (30%)	43 (47.3%)	
	Arabs	13 (32.5%)	35 (38.5%)	
	Asians	6 (15%)	10 (10.1%)	
	Other nationalities	1 (2.5%)	0	
**Degree#**	Medicine	17 (44.7%)	47 (52.8%)	p = 0.012
	Pharmacy	8 (21.1%)	16 (18%)	
	Dentistry	10 (26.3%)	8 (9%)	
	Nursing	2 (5.3%)	0	
	Engineering	1 (2.6%)	18 (20.2%)	
**Year of Study#**	First	7 (17.5%)	2 (2.2%)	p = 0.121
	Second	7 (17.5%)	9 (9.9%)	
	Third	9 (22.5%)	29 (31.9%)	
	Fourth	15 (37.5%)	38 (41.8%)	
	Fifth	1 (2.5%)	11 (12.1%)	
	Sixth	1 (2.5%)	1 (1.1%)	

*p values for chi-squared test (or #Fisher’s exact test where necessary) to compare the gender-based differences in the demographics among the CE users only.

Percentages may not sum to 100 due to rounding.

Characteristics of CE use are compared by gender in Table 6. Most common reasons behind CE intake was “academic performance” (30.5%) followed by “concentration” (27.5%) and “alertness” (25.2%). With respect to females, male users felt that a positive effect was more frequently associated with CE use (87.9% vs 60.0; p = 0.008). CEs were mostly ingested during the “Exams” period (80.1%) and most typically for a period of “1–6 months” (53.4%) and “<1 month” (29%), with some 52.6% having reported a CE intake once/less than once per semester. The duration of CE use seemed more prolonged in males (e.g., 67% used CEs for 1–12 months, whilst 40% of females used CEs for less than 1 month). Conversely, about one third of students reported a daily CE intake. The web, and especially so for males, was identified as the main CE source in about two thirds of cases, while only 9% of users had a CE prescribed to them. Friends and online advertisements represented here the major channels through which the students were made aware of CEs, with no significant differences by gender. Half of CE users considered CEs as “expensive”; finally, vitamin B12, caffeine and modafinil were the most frequently reported CEs. Vitamin B6 intake was more common in females (22.5% vs 5.5%; p = 0.014) and modafinil in males (48.4% vs 7.5%; p<0.001).

**Table 6:** CE characteristics of use among UAE students; differences according to gender.

		Female(40)	Male (91)	p value*
**Reason**	Concentration	12(30.0%)	24(26.4%)	0.676
	Memory	2 (5.0%)	9 (9.9%)	0.502
	Alertness	7(17.5%)	26(28.6%)	0.198
	Academic performance	9(22.5%)	31(34.1%)	0.220
	Other reasons	10 (25.0%)	1 (1.1%)	<0.001
**Positive effect (yes)#**		24 (60.0%)	80(87.9%)	0.008
**Time#**	Exams	27 (67.5%)	78(85.7%)	0.267
	Course deadlines	2 (5.0%)	1 (1.1%)	
	Studying	5 (12.5%)	9 (9.9%)	
	Daily	4 (10.0%)	1 (1.1%)	
	Other	2 (5.0%)	2 (2.2%)	
**Duration#**	< 1 month	16 (40.0%)	22(24.2%)	0.027
	1–6 months	12 (30.0%)	58(63.7%)	
	6–12 months	7 (17.5%)	8 (8.8%)	
	1–2 years	0	2 (2.2%)	
	2 years or more	5 (12.5%)	1 (1.1%)	
**Frequency#**	Daily	15 (37.5%)	23(25.3%)	0.705
	Weekly	3 (7.5%)	7 (7.7%)	
	Monthly	3 (7.5%)	8 (8.8%)	
	Once/semester	9 (22.5%)	34(37.4%)	
	< Once a semester	8 (20.0%)	18(19.8%)	
**Source of acquisition#**	Prescribed to me	9 (22.5%)	3 (3.3%)	0.022
	Friend	6 (15.0%)	17(18.7%)	
	Online	17 (42.5%)	64(70.3%)	
	Other	8 (20.0%)	7 (7.7%)	
**Cost**	Very expensive/ expensive	12 (31.6%)	56(62.9%)	0.002
	Fair/Cheap	26 (68.4%)	33(37.1%)	
**Heard about#**	Social media	3 (7.5%)	10 (11%)	0.052
	Scientific literature	3 (12.5%)	2 (2.2%)	
	Internet	9 (22.5%)	24(26.4%)	
	Friends	14 (35.0%)	49(53.8%)	
	Family	2 (5.0%)	4 (4.4%)	
	Other	7 (17.5%)	2 (2.2%)	
**Drug used**	B12	18 (55.0%)	32(35.2%)	0.255
	B6#	9 (22.5%)	5 (5.5%)	0.014
	Methylphenidate	3 (7.5%)	9 (9.9%)	0.864
	Adderall/amphetamines#	1 (2.5%)	9 (9.9%)	0.325
	Super caffeine pills	16 (40.0%)	35(38.5%)	0.718
	Modafinil	3 (7.5%)	44(48.4%)	<0.001
	Paullinia Cupana/Guarana#	1 (2.5%)	3 (3.3%)	0.955

*p values for Chi-square test (or #Fisher’s exact when necessary) to compare the gender-based differences in characteristics of CE use in CE users only. Percentages are reported as fraction of the total number of male/female in the CEs users.

Medical students reportedly took CEs for a range of different reasons, whilst memory performance improvement did not represent a reason for CE use among other programmes (p<0.001; chi-squared test). Similarly, compared to other programme students, medical students were those most frequently reporting: a daily use of CEs (p<0.001); resourcing to the web for CE acquisition (p<0.05); and of having been influenced by peers in deciding to access CEs (p<0.05).

Modafinil was self-administered, especially in males, for concentration and alertness; B12 was typically taken by female students for academic performance and concentration; and high-dosage caffeine compounds were ingested to improve alertness levels.

## Discussion

To the best of our understanding, this paper is the first one to focus on CE use in Higher Education Universities in the United Arab Emirates. This study explored the prevalence of use; the users’ socio-demographics, and the CE characteristics of use in a large sample (*n =* 522) of students. Among the large sample of participants who completed the online survey, one quarter were found to be CE users; a result which is in line with those identified among university students from both the UK [36] and Iran [37]. This level of CE use seems to be at odds with previous suggestions [11] that students are resistant to using CEs.

Around two thirds of CE users were males, a finding which is consistent with several previous studies [7, 13, 38–40]. Indeed, there may be gender-related different attitudes towards both recreational [41] and CE drug use [5, 42, 43] worldwide, although some conflicting results have been published [44, 45].

About one half of CE users were medical students, followed by Pharmacy, Dentistry, Engineering and Nursing, with their usage increasing in the final years of study programmes when the medical students start to have more patient interaction and get under the stress of clinical rotations. This may tentatively suggest that the usage of CEs is in most cases transient and may start only in association of increasing levels of academic load. Present findings support previous studies, which reported levels of use of CEs among both medical [39, 42, 46, 47] and pharmacy students [19]; levels which are higher than those recorded in other schools [22].

Improving academic performance, concentration and alertness were among the main reasons to ingest CEs, and especially so during the exam preparation times. Other studies also reported the same motivations for use [37, 42, 48–50]. However, relative to their male counterparts, the reasons for CEs use included concentration (30.0% vs 26.4 respectively in females and males) but not alertness (17.5% vs 28.6% respectively in females and males).

In line with previous findings [36, 43] peer influence and the web were here reported as facilitating the students’ uptake of CEs. Most users, but especially so males (e.g., 70% vs 40%) accessed their CEs from online sources, with the web having been previously described as the focus of drug acquisition activities [51–53]. Indeed, a high circulation UK newspaper has reported on the high levels of online sales of modafinil, being shipped to students from the universities of Cambridge, Oxford and London Imperial College [54] and more frequently so at the time of the exams. Sources of CEs was another aspect where nationality played a role in differences in responses among CEs users. While Emirati and Arab students favoured the internet option, other nationalities relied on a range of different sources to access the index CEs.

Overall, B12 was mainly taken for academic performance reasons, followed by concentration. Modafinil was also taken for concentration and alertness, confirming previous findings [55]. Conversely, in line with previous studies [11, 15], caffeine/ super-strength caffeine pills were mainly ingested for alertness. The distribution of the CEs drugs ingested showed differences relating to gender. In particular, B6 was more popular in females than in males (e.g., 22.5% vs 5.5%), whilst modafinil was more significantly reported in males (e.g., 48.4% vs 7.5%). In line with a recent study conducted in the UK [36], the intake of remaining CEs was not significantly different between the two groups.

This study has shown that it is important to raise awareness of the harms of CE use, provide knowledge, counteract myths regarding the safety of CE use and address cognitive enhancement at an early stage of higher education as preventative public health measures [6]. Indeed, universities should focus on correcting the wrong impression of the benefits of the sustained use of ‘smart drugs’, a practice here suggested to be highly prevalent among UAE university students.

## Strengths

This is the first study to estimate the prevalence of CE use among UAE university students for the purpose of improving academic performance, concentration, and alertness. In addition, despite the sensitive nature of the subject, we were able to compare CE users with non-users and identify characteristics associated with a higher risk of CE use. Participation in the study was anonymous, limiting the effects of bias.

## Limitations

There are a few limitations in our study to be acknowledged. The major and first limitation was the delay of data collection from UAE universities due to the COVID-19 pandemic; whilst the initial plan was for three months only, the survey remained available for almost 8 months. The second limitation to acknowledge is that the current study focused only on undergraduate students from the Medical, Pharmacy, Dentistry, Engineering and Nursing undergraduate courses; combined with a response rate of 7% this may limit the generalizability of the results. The third limitation is that due to the sensitive nature of the subject, CE non-users were asked to provide only their gender and age group. Since only 30% of the CE non-users reported additional demographic information, there is a potential for bias in the current findings.

## Conclusions

The high levels of reported prevalence of CE use may be a reason of concern. Indeed, these molecules appeared here to be mostly acquired without the consultation of a physician for both diagnostic and monitoring purposes. Furthermore, it should be noted that in the UAE it is possible to access medicinal compounds without a prescription from pharmacies [56].

At this stage, a few issues need to be better addressed in future studies, including health of student CE users; and understanding if these drugs may put some of these students at an unfair advantage over remaining non using students.

Universities may need to develop better awareness levels regarding the prevalence of cognitive enhancers’ use amongst their students and consider taking an active approach in reducing their use, educating students on the dangers of CEs use whilst providing them with a safe space to seek help.

## Supporting information

S1 Fig Study flow chart. (TIF)

S1 Dataset (PDF)

S1 Appendix (DOCX)

## References

[1] Schelle K.J.; Olthof B.M.J.; Reintjes W.; Bundt C.; Gusman-Vermeer J.; van Mil A.C. C.M. A Survey of Substance Use for Cognitive Enhancement by University Students in the Netherlands. *Front*. *Syst*. *Neurosci*. 2015, 9, 10, doi: 10.3389/fnsys.2015.00010 25741248 PMC4330699

[2] Smith M.E.; Farah M.J. Are Prescription Stimulants “Smart Pills”? The Epidemiology and Cognitive Neuroscience of Prescription Stimulant Use by Normal Healthy Individuals. *Psychol*. *Bull*. 2011, 137, 717–741, doi: 10.1037/a0023825 21859174 PMC3591814

[3] Husain M.; Mehta M.A. Cognitive Enhancement by Drugs in Health and Disease. *Trends Cogn*. *Sci*. 2011, 15, 28–36, doi: 10.1016/j.tics.2010.11.002 21146447 PMC3020278

[4] Bostrom N.; Sandberg A. Cognitive Enhancement: Methods, Ethics, Regulatory Challenges. *Sci Eng Ethics* 2009, 15, doi: 10.1007/s11948-009-9142-5 19543814

[5] Maier L.J.; Liechti M.E.; Herzig F.; Schaub M.P. To Dope or Not to Dope: Neuroenhancement with Prescription Drugs and Drugs of Abuse among Swiss University Students. *PLOS ONE* 2013, 8, e77967. doi: 10.1371/journal.pone.0077967 24236008 PMC3827185

[6] Sharif S.; Guirguis A.; Fergus S.; Schifano F. *The Use and Impact of Cognitive Enhancers among University Students: A Systematic Review*; 2021; Vol. 11; ISBN 2076–3425. 10.3390/brainsci11030355 PMC8000838 33802176

[7] Franke A. G.1, C.B. Non-Medical Use of Prescription Stimulants and Illicit Use of Stimulants for Cognitive Enhancement in Pupils and Students in Germany. 2010. 10.1055/s-0030-1268417 21161883

[8] Carlier J.; Giorgetti R.; Varì M.R.; Pirani F.; Ricci G.; Busardò F.P. Use of Cognitive Enhancers: Methylphenidate and Analogs. *Eur*. *Rev*. *Med*. *Pharmacol*. *Sci*. 2019, 23, 3–15, doi: 10.26355/eurrev_201901_16741 30657540

[9] Cappelletti S.; Piacentino D.; Sani G.; Aromatario M. Caffeine: Cognitive and Physical Performance Enhancer or Psychoactive Drug? *Curr. Neuropharmacol*. 2015, 13, 71–88, doi: 10.2174/1570159X13666141210215655 26074744 PMC4462044

[10] Dresler M.; Sandberg A.; Bublitz C.; Ohla K.; Trenado C.; Mroczko-Wąsowicz A.; et al. Hacking the Brain: Dimensions of Cognitive Enhancement. *ACS Chem*. *Neurosci*. 2019, 10, 1137–1148, doi: 10.1021/acschemneuro.8b00571 30550256 PMC6429408

[11] Singh I.; Bard I.; Jackson J. Robust Resilience and Substantial Interest: A Survey of Pharmacological Cognitive Enhancement among University Students in the UK and Ireland. *PLoS ONE* 2014, 9, doi: 10.1371/journal.pone.0105969 25356917 PMC4214670

[12] Holloway K.; Bennett T. Prescription Drug Misuse among University Staff and Students: A Survey of Motives, Nature and Extent. *Drugs Educ*. *Prev*. *Policy* 2012, 19, 137–144, doi: 10.3109/09687637.2011.594114

[13] Benson K.; Flory K.; Humphreys K.L.; Lee S.S. Misuse of Stimulant Medication Among College Students: A Comprehensive Review and Meta-Analysis. *Clin*. *Child Fam*. *Psychol*. *Rev*. 2015, 18, 50–76, doi: 10.1007/s10567-014-0177-z 25575768

[14] Cândido R.C.F.; Perini E.; Pádua C.M.; Junqueira D.R. Prevalence of and Factors Associated with the Use of Methylphenidate for Cognitive Enhancement among University Students. *Einstein Sao Paulo Braz*. 2019, 18, eAO4745, doi: 10.31744/einstein_journal/2020AO4745 31664322 PMC6896602

[15] Ghali R.M.A.; Shaibi H.A.; Majed H.A.; Haroun D. Caffeine Consumption among Zayed University Students in Dubai, United Arab Emirates: A Cross-Sectional Study. *Arab J*. *Nutr*. *Exerc*. *AJNE* 2016, 131–141, doi: 10.18502/ajne.v1i3.1230

[16] Greely H.; Sahakian B.; Harris J.; Kessler R.C.; Gazzaniga M.; Campbell P.; et al. Towards Responsible Use of Cognitive-Enhancing Drugs by the Healthy. *Nature* 2008, 456, 702–705, doi: 10.1038/456702a 19060880

[17] Maier L.J.; Ferris J.A.; Winstock A.R. Pharmacological Cognitive Enhancement among Non-ADHD Individuals—A Cross-Sectional Study in 15 Countries. *Int*. *J*. *Drug Policy* 2018, 58, 104–112, doi: 10.1016/j.drugpo.2018.05.009 29902691

[18] Fallah G.; Moudi S.; Hamidia A.; Bijani A. Stimulant Use in Medical Students and Residents Requires More Careful Attention. *Casp*. *J*. *Intern*. *Med*. 2018, 9, 87–91, doi: 10.22088/cjim.9.1.87 29387325 PMC5771366

[19] Hanna L.; Rainey J.; Hall M. A Questionnaire Study Investigating Future Pharmacists ‘ Use of, and Views on Cognitive Enhancers. 2018, 18, 76–84.

[20] Alrakaf F.; Binyousef F.; Altammami A.; Alharbi A.; Shadid A.; Alrahili N. Illicit Stimulant Use among Medical Students in Riyadh, Saudi Arabia. *Cureus* 2020, 12, doi: 10.7759/cureus.6688 32104625 PMC7026881

[21] Awosanya E.J.; Ogundipe G.; Babalobi O.; Omilabu S. Prevalence and Correlates of Influenza-A in Piggery Workers and Pigs in Two Communities in Lagos, Nigeria. *Pan Afr. Med. J.* 2013, 16, 102, doi: 10.11604/pamj.2013.16.102.1450 24711881 PMC3972905

[22] Bossaer J.B.; Gray J.A.; Miller S.E.; Enck G.; Gaddipati V.C.; Enck R.E. The Use and Misuse of Prescription Stimulants as “Cognitive Enhancers” by Students at One Academic Health Sciences Center. *Acad*. *Med*. 2013, 88. doi: 10.1097/ACM.0b013e318294fc7b 23702522

[23] Castaldi S.; Gelatti U.; Orizio G.; Hartung U.; Moreno-Londono A.M.; Nobile M.; et al. Use of Cognitive Enhancement Medication Among Northern Italian University Students. *J*. *Addict*. *Med*. 2012, 6. doi: 10.1097/ADM.0b013e3182479584 22456492

[24] Jensen C.; Forlini C.; Partridge B.; Hall W. Australian University Students’ Coping Strategies and Use of Pharmaceutical Stimulants as Cognitive Enhancers. *Front*. *Psychol*. 2016, 7, 1–9, doi: 10.3389/fpsyg.2016.00001 26973573 PMC4771940

[25] Ragan C.I.; Bard I.; Singh I. What Should We Do about Student Use of Cognitive Enhancers? An Analysis of Current Evidence 2013. doi: 10.1016/j.neuropharm.2012.06.016 22732441

[26] Smith D.H. Cognitive Enhancers. *Cmaj* 2013, 185, 799, doi: 10.1503/cmaj.113-2118 23754827 PMC3680561

[27] Bowling A. *Research Methods In Health*: *Investigating Health And Health Services*; Open University Press; Open University Press, 2014; ISBN 978-0-335-26274-8.

[28] McGartland Rubio D. Content Validity. In; Kempf-Leonard, K.B.T.-E. of S.M., Ed.; Elsevier: New York, 2005; pp. 495–498 ISBN 978-0-12-369398-3.

[29] Al-Issa A. English As a Medium of Instruction and the Endangerment of Arabic Literacy: The Case of the United Arab Emirates. *Arab World Engl. J*. 2017, 8, 3–17, doi: 10.24093/awej/vol8no3.1

[30] Zaami S.; Tagliabracci A.; Berretta P.; Busardò F.P.; Marinelli E. *Use of Methylphenidate Analogues as Cognitive Enhancers*: *The Prelude to Cosmetic Neurology and an Ethical Issue*; 2020; Vol. 10; ISBN 1664–0640. 10.3389/fpsyt.2019.01006 PMC6989549 32038333

[31] Reips U.-D. Standards for Internet-Based Experimenting. *Exp*. *Psychol*. 2002, 49, 243–256, doi: 10.1026/1618-3169.49.4.243 12455331

[32] Information Commissioner’s Office Guide to the General Data Protection Regulation (GDPR). *Guide Gen. Data Prot. Regul*. 2018, n/a, doi: 10.1111/j.1751-1097.1994.tb09662.x 8066118

[33] Tavakol M.; Dennick R. Making Sense of Cronbach’s Alpha. *Int*. *J*. *Med*. *Educ*. 2011, 2, 53–55, doi: 10.5116/ijme.4dfb.8dfd 28029643 PMC4205511

[34] Kim H.-Y. Statistical Notes for Clinical Researchers: Chi-Squared Test and Fisher’s Exact Test. *Restor*. *Dent*. *Endod*. 2017, 42, 152–155, doi: 10.5395/rde.2017.42.2.152 28503482 PMC5426219

[35] David W. Hosmer, Stanley Lemeshow *Applied Logistic Regression*, *Second Edition*; New York, 2000; ISBN 978-0-471-35632-5.

[36] McDermott H.; Lane H.; Alonso M. Working Smart: The Use of ‘cognitive Enhancers’ by UK University Students. *J*. *Furth*. *High*. *Educ*. 2020, 1–14, doi: 10.1080/0309877X.2020.1753179

[37] Abbasi-Ghahramanloo A.; Fotouhi A.; Zeraati H.; Rahimi-Movaghar A. Prescription Drugs, Alcohol, and Illicit Substance Use and Their Correlations among Medical Sciences Students in Iran. *Int. J. High Risk Behav. Addict*. 2015, 4, e21945–e21945, doi: 10.5812/ijhrba.21945 25821750 PMC4360541

[38] Champagne J.; Gardner B.; Dommett E.J. Modelling Predictors of UK Undergraduates’ Attitudes towards Smart Drugs. *Trends Neurosci*. *Educ*. 2019, 14, 33–39, doi: 10.1016/j.tine.2019.02.001 30929857

[39] Emanuel R.M.; Frellsen S.L.; Kashima K.J.; Sanguino S.M.; Sierles F.S.; Lazarus C.J. Cognitive Enhancement Drug Use among Future Physicians: Findings from a Multi-Institutional Census of Medical Students. *J*. *Gen*. *Intern*. *Med*. 2013, 28, 1028–1034, doi: 10.1007/s11606-012-2249-4 23595918 PMC3710394

[40] Gudmundsdottir B.G.; Weyandt L.; Ernudottir G.B. Prescription Stimulant Misuse and ADHD Symptomatology Among College Students in Iceland. *J*. *Atten*. *Disord*. 2016, 108705471668437, doi: 10.1177/1087054716684379 28013572

[41] Schifano F. Is Urbanization a Risk Factor for Substance Misuse? *Curr. Opin. Psychiatry* 2008, 21. doi: 10.1097/YCO.0b013e328303e2b7 18520745

[42] Lengvenytė A.; Strumila R. Do Medical Students Use Cognitive Enhancers to Study? Prevalence and Correlates from Lithuanian Medical Students Sample. *Eur*. *Psychiatry* 2016, 33, S304, 10.1016/j.eurpsy.2016.01.1041.

[43] Mache S.; Eickenhorst P.; Vitzthum K.; Klapp B.F.; Groneberg D.A. Cognitive-Enhancing Substance Use at German Universities: Frequency, Reasons and Gender Differences. *Wien*. *Med*. *Wochenschr*. 2012, 162, 262–271, doi: 10.1007/s10354-012-0115-y 22707077

[44] McNeil A.; Muzzin K.; DeWald J.; McCann A.; Schneiderman E.; Scofield J.; et al. The Nonmedical Use of Prescription Stimulants Among Dental and Dental Hygiene Students. *J*. *Dent*. *Educ*. 2011, March, 365–376. 21368261

[45] Weyandt L. No Title. *J*. *Atten*. *Disord*. 2009, 13, 3. doi: 10.1177/1087054708323005 19834126

[46] Habibzadeh A.; Alizadeh M.; Malek A.; Maghbooli L.; Shoja M.M.; Ghabili K. Illicit Methylphenidate Use among Iranian Medical Students: Prevalence and Knowledge. *Drug Des*. *Devel*. *Ther*. 2011, 71–76, doi: 10.2147/DDDT.S13818 21340040 PMC3038997

[47] Pighi M.; Pontoni G.; Sinisi A.; Ferrari S.; Mattei G.; Pingani L.; et al. Use and Propensity to Use Substances as Cognitive Enhancers in Italian Medical Students. *Brain Sci*. 2018, 1–9, doi: 10.3390/brainsci8110197 30423911 PMC6266090

[48] Emanuel R.M.; Frellsen S.L.; Kashima K.J.; Sanguino S.M.; Sierles F.S.; Lazarus C.J. Cognitive Enhancement Drug Use among Future Physicians: Findings from a Multi-Institutional Census of Medical Students. *J*. *Gen*. *Intern*. *Med*. 2013, 28, 1028–1034, doi: 10.1007/s11606-012-2249-4 23595918 PMC3710394

[49] Mazanov J.; Dunn M.; Connor J.; Fielding M.-L. Substance Use to Enhance Academic Performance among Australian University Students. *Perform. Enhanc. Health* 2013, 2, 110–118, doi: 10.1016/j.peh.2013.08.017

[50] Ram S. (Sanya); Hussainy S.; Henning M.; Stewart K.; Jensen M.; Russell B. Attitudes Toward Cognitive Enhancer Use Among New Zealand Tertiary Students. *Subst*. *Use Misuse* 2017, 52, 1387–1392, doi: 10.1080/10826084.2017.1281313 28429997

[51] Corazza O.; Bersani F.S.; Brunoro R.; Valeriani G.; Martinotti G.; Schifano F. The Diffusion of Performance and Image-Enhancing Drugs (PIEDs) on the Internet: The Abuse of the Cognitive Enhancer Piracetam. *Subst*. *Use Misuse* 2014, 49, 1849–1856, doi: 10.3109/10826084.2014.912232 24827869

[52] Hockenhull J.; Wood D.M.; Dargan P.I. The Availability of Modafinil and Methylphenidate Purchased from the Internet in the United Kingdom Without a Prescription. *Subst*. *Use Misuse* 2020, 55, 56–65, doi: 10.1080/10826084.2019.1654516 31431114

[53] Mooney R.; Simonato P.; Ruparelia R.; Roman-Urrestarazu A.; Martinotti G.; Corazza O. The Use of Supplements and Performance and Image Enhancing Drugs in Fitness Settings: A Exploratory Cross-Sectional Investigation in the United Kingdom. *Hum. Psychopharmacol. Clin. Exp*. 2017, 32, e2619, doi: 10.1002/hup.2619 28657184

[54] Marsh S. Universities Must Do More to Tackle Use of Smart Drugs, Say Experts. *The Guardian* 2017.

[55] Turner D.C.; Robbins T.W.; Clark L.; Aron A.R.; Dowson J.; Sahakian B.J. Cognitive Enhancing Effects of Modafinil in Healthy Volunteers. *Psychopharmacology (Berl.)* 2003, 165, 260–269, doi: 10.1007/s00213-002-1250-8 12417966

[56] Sharif S.I.; Bugaighis L.M.T.; Sharif R.S. Self-Medication Practice among Pharmacists in UAE. *Pharmacol*. *Pharm*. 2015, 06, 428, doi: 10.4236/pp.2015.69044

