# Supplementary figures and images for: Assessing prevalence, knowledge and use of cognitive enhancers among university students in the United Arab Emirates: A quantitative study
Source: PLoS One. 2022 Jan 26;17(1):e0262704. doi: 10.1371/journal.pone.0262704 (PMC8791475; doi:10.1371/journal.pone.0262704)

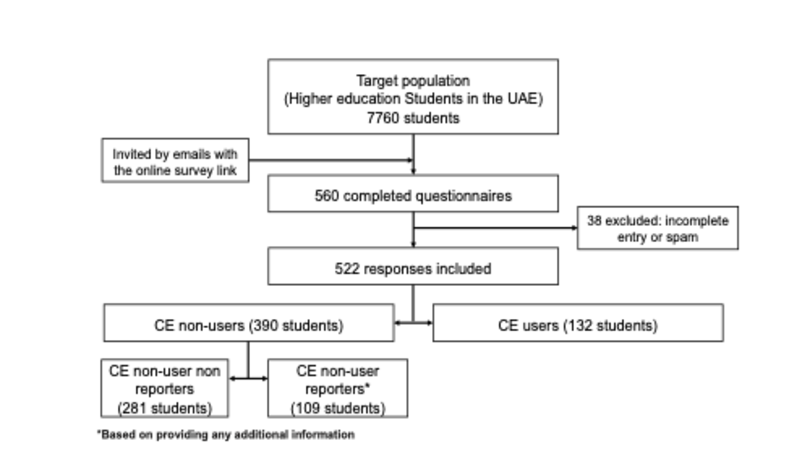

Supplement: S1 Fig — (TIF) [file pone.0262704.s001.tif]
