# Supplementary material for: Assessing prevalence, knowledge and use of cognitive enhancers among university students in the United Arab Emirates: A quantitative study
Source: PLoS One. 2022 Jan 26;17(1):e0262704. doi: 10.1371/journal.pone.0262704 (PMC8791475; doi:10.1371/journal.pone.0262704)
Supplement: S1 Dataset — (PDF) [file pone.0262704.s002.pdf]

| country | Duration (in seconds) distributionChannel<br>Duration (in seconds) | distributionChannel | gender | age | usage | drugUsed | guarana | B12 |
|---|---|---|---|---|---|---|---|---|
| 2 | 83 | 2 | 1 | 1 | 2 | | 0 | 0 |
| 2 | 109 | 2 | 1 | 1 | 2 | | 0 | 0 |
| 2 | 797 | 2 | 1 | 1 | 1 | 8,9 | 0 | 1 |
| 2 | 121 | 2 | 1 | 1 | 2 | | 0 | 0 |
| 2 | 64 | 2 | 1 | 1 | 2 | | 0 | 0 |
| 2 | 107 | 2 | 1 | 1 | 2 | | 0 | 0 |
| 2 | 83 | 2 | 1 | 1 | 2 | | 0 | 0 |
| 2 | 60 | 2 | 2 | 1 | 2 | | 0 | 0 |
| 2 | 239 | 2 | 1 | 1 | 2 | | 0 | 0 |
| 2 | 114 | 2 | 1 | 1 | 2 | | 0 | 0 |
| 2 | 34 | 2 | 1 | 1 | 2 | | 0 | 0 |
| 2 | 32 | 2 | 2 | 1 | 2 | | 0 | 0 |
| 2 | 127 | 2 | 1 | 1 | 2 | | 0 | 0 |
| 2 | 78 | 2 | 1 | 1 | 2 | | 0 | 0 |
| 2 | 80 | 2 | 1 | 1 | 2 | | 0 | 0 |
| 2 | 134 | 2 | 1 | 1 | 2 | | 0 | 0 |
| 2 | 83 | 2 | 1 | 1 | 2 | | 0 | 0 |
| 2 | 45 | 2 | 1 | 1 | 2 | | 0 | 0 |
| 2 | 94 | 2 | 2 | 1 | 2 | | 0 | 0 |
| 2 | 300 | 2 | 1 | 1 | 1 | 9 | 0 | 1 |
| 2 | 157 | 2 | 1 | 3 | 2 | | 0 | 0 |
| 2 | 106 | 2 | 1 | 1 | 2 | | 0 | 0 |
| 2 | 85 | 2 | 1 | 1 | 2 | | 0 | 0 |
| 2 | 44 | 2 | 1 | 1 | 2 | | 0 | 0 |
| 2 | 107 | 2 | 1 | 1 | 2 | | 0 | 0 |
| 2 | 85 | 2 | 1 | 1 | 2 | | 0 | 0 |
| 2 | 300 | 2 | 2 | 1 | 1 | 5 | 0 | 0 |
| 2 | 187 | 2 | 1 | 1 | 2 | | 0 | 0 |
| 2 | 43 | 2 | 1 | 1 | 2 | | 0 | 0 |
| 2 | 134 | 2 | 3 | 1 | 2 | | 0 | 0 |
| 2 | 314 | 2 | 2 | 1 | 1 | 5,8,9,11 | 0 | 1 |
| 2 | 38 | 2 | 1 | 1 | 2 | | 0 | 0 |
| 2 | 78 | 2 | 2 | 1 | 2 | | 0 | 0 |
| 2 | 50 | 2 | 2 | 1 | 2 | | 0 | 0 |
| 2 | 205 | 2 | 2 | 1 | 3 | 9 | 0 | 1 |
| 2 | 366 | 2 | 1 | 1 | 2 | | 0 | 0 |
| 2 | 71 | 2 | 2 | 1 | 2 | | 0 | 0 |
| 2 | 101 | 2 | 1 | 1 | 2 | | 0 | 0 |
| 2 | 45 | 2 | 1 | 1 | 2 | | 0 | 0 |
| 2 | 63 | 2 | 2 | 1 | 2 | | 0 | 0 |
| 2 | 139 | 2 | 1 | 1 | 2 | | 0 | 0 |
| 2 | 45 | 2 | 1 | 1 | 2 | | 0 | 0 |
| 2 | 55 | 2 | 1 | 1 | 2 | | 0 | 0 |
| 2 | 18 | 2 | 2 | 1 | 2 | | 0 | 0 |
| 2 | 103 | 2 | 1 | 1 | 3 | 5 | 0 | 0 |
| 2 | 69 | 2 | 1 | 1 | 2 | | 0 | 0 |

| | | | | | | | | | |
|---|---|---|---|---|---|---|---|---|---|
| 2 | 35 | 2 | 1 | 1 | 2 | | | 0 | 0 |
| 2 | 172 | 2 | 1 | 1 | 1 | 5,8,9 | | 0 | 1 |
| 2 | 38 | 2 | 1 | 3 | 2 | | | 0 | 0 |
| 2 | 121 | 2 | 1 | 1 | 1 | | 5 | 0 | 0 |
| 2 | 582 | 2 | 2 | 1 | 2 | | | 0 | 0 |
| 2 | 103 | 2 | 1 | 1 | 2 | | | 0 | 0 |
| 2 | 61 | 2 | 1 | 1 | 1 | | | 0 | 0 |
| 2 | 36 | 2 | 1 | 1 | 2 | | | 0 | 0 |
| 2 | 314 | 2 | 1 | 1 | 3 | 8,9 | | 0 | 1 |
| 2 | 71 | 2 | 1 | 1 | 2 | | | 0 | 0 |
| 2 | 47 | 2 | 1 | 1 | 2 | | | 0 | 0 |
| 2 | 401 | 2 | 1 | 1 | 2 | | 11 | 0 | 0 |
| 2 | 98 | 2 | 1 | 1 | 2 | | | 0 | 0 |
| 2 | 383 | 2 | 1 | 1 | 1 | | 4 | 0 | 0 |
| 2 | 221 | 2 | 2 | 1 | 1 | 1,9 | | 0 | 1 |
| 2 | 254 | 2 | 2 | 1 | 2 | | | 0 | 0 |
| 2 | 64 | 2 | 2 | 1 | 2 | | | 0 | 0 |
| 2 | 277877 | 2 | 2 | 1 | 2 | | | 0 | 0 |
| 2 | 188 | 2 | 1 | 1 | 1 | | 5 | 0 | 0 |
| 2 | 165 | 3 | 2 | 1 | 1 | | 2 | 0 | 0 |
| 2 | 32 | 2 | 2 | 1 | 2 | | | 0 | 0 |
| 2 | 83 | 2 | 1 | 1 | 2 | | | 0 | 0 |
| 2 | 58 | 2 | 1 | 1 | 2 | | | 0 | 0 |
| 2 | 284 | 2 | 2 | 1 | 2 | | | 0 | 0 |
| 2 | 836 | 2 | 1 | 1 | 2 | | | 0 | 0 |
| 2 | 106 | 2 | 1 | 1 | 2 | | | 0 | 0 |
| 2 | 46 | 2 | 1 | 1 | 2 | | | 0 | 0 |
| 2 | 58 | 2 | 2 | 1 | 2 | | | 0 | 0 |
| 2 | 127 | 2 | 1 | 1 | 2 | | | 0 | 0 |
| 2 | 68 | 2 | 1 | 1 | 2 | | | 0 | 0 |
| 2 | 297445 | 2 | 1 | 1 | 2 | | | 0 | 0 |
| 2 | 123 | 3 | 2 | 1 | 1 | 2,9 | | 0 | 1 |
| 2 | 101 | 2 | 2 | 1 | 1 | | 3 | 0 | 0 |
| 2 | 218 | 3 | 2 | 1 | 1 | | 5 | 0 | 0 |
| 2 | 122 | 2 | 2 | 1 | 1 | | 5 | 0 | 0 |
| 2 | 38 | 2 | 1 | 1 | 2 | | | 0 | 0 |
| 2 | 90 | 2 | 1 | 1 | 1 | 5,9 | | 0 | 1 |
| 2 | 106 | 2 | 2 | 1 | 1 | | 2 | 0 | 0 |
| 2 | 71 | 2 | 2 | 1 | 1 | 2,5 | | 0 | 0 |
| 2 | 179 | 2 | 2 | 1 | 1 | 2,5,9 | | 0 | 1 |
| 2 | 194 | 2 | 2 | 1 | 1 | 2,9 | | 0 | 1 |
| 2 | 62 | 2 | 2 | 1 | 1 | | 9 | 0 | 1 |
| 2 | 54 | 2 | 2 | 1 | 1 | | 2 | 0 | 0 |
| 2 | 62 | 2 | 2 | 1 | 1 | 2,9 | | 0 | 1 |
| 2 | 88 | 2 | 2 | 1 | 1 | 2,9 | | 0 | 1 |
| 2 | 166 | 2 | 1 | 1 | 1 | | 5 | 0 | 0 |
| 2 | 141 | 2 | 1 | 1 | 2 | | | 0 | 0 |
| 2 | 81 | 2 | 2 | 1 | 1 | 2,5 | | 0 | 0 |

| | | | | | | | | | |
|---|---|---|---|---|---|---|---|---|---|
| 2 | 100 | 2 | 2 | 1 | 1 | 2,5,6 | | 1 | 0 |
| 2 | 61 | 2 | 2 | 1 | 1 | 5,9 | | 0 | 1 |
| 2 | 70 | 2 | 2 | 1 | 1 | 2,5,9 | | 0 | 1 |
| 2 | 54 | 2 | 2 | 1 | 1 | | 5 | 0 | 0 |
| 2 | 25 | 2 | 1 | 1 | 2 | | | 0 | 0 |
| 2 | 75 | 2 | 2 | 1 | 1 | 2,9 | | 0 | 1 |
| 2 | 49 | 2 | 2 | 1 | 1 | | 2 | 0 | 0 |
| 2 | 78 | 2 | 2 | 1 | 1 | | 3 | 0 | 0 |
| 2 | 27 | 2 | 2 | 1 | 2 | | | 0 | 0 |
| 2 | 11 | 2 | 2 | 1 | 2 | | | 0 | 0 |
| 2 | 88 | 2 | 2 | 1 | 1 | 2,5 | | 0 | 0 |
| 2 | 62 | 2 | 2 | 1 | 1 | | 3 | 0 | 0 |
| 2 | 11 | 2 | 2 | 1 | 2 | | | 0 | 0 |
| 2 | 88 | 2 | 2 | 1 | 1 | 3,4 | | 0 | 0 |
| 2 | 51 | 3 | 2 | 1 | 1 | 2,9 | | 0 | 1 |
| 2 | 328 | 2 | 2 | 1 | 1 | 2,9 | | 0 | 1 |
| 2 | 56 | 2 | 2 | 1 | 1 | 2,5 | | 0 | 0 |
| 2 | 63 | 2 | 2 | 1 | 1 | 3,,4 | | 0 | 0 |
| 2 | 12 | 2 | 1 | 1 | 2 | | | 0 | 0 |
| 2 | 11 | 2 | 1 | 1 | 2 | | | 0 | 0 |
| 2 | 50 | 2 | 1 | 1 | 1 | 2,5 | | 0 | 0 |
| 2 | 13 | 2 | 2 | 1 | 2 | | | 0 | 0 |
| 2 | 11 | 2 | 1 | 1 | 2 | | | 0 | 0 |
| 2 | 56 | 2 | 2 | 1 | 1 | 3,4 | | 0 | 0 |
| 2 | 14 | 2 | 2 | 1 | 2 | | | 0 | 0 |
| 2 | 11 | 2 | 2 | 1 | 2 | | | 0 | 0 |
| 2 | 10 | 2 | 1 | 1 | 2 | | | 0 | 0 |
| 2 | 12 | 2 | 1 | 1 | 2 | | | 0 | 0 |
| 2 | 62 | 2 | 2 | 1 | 1 | 2,9 | | 0 | 1 |
| 2 | 12 | 2 | 2 | 1 | 2 | | | 0 | 0 |
| 2 | 60 | 2 | 2 | 1 | 1 | | 1 | 0 | 0 |
| 2 | 21 | 2 | 2 | 1 | 2 | | | 0 | 0 |
| 2 | 4 | 3 | 3 | 3 | 2 | | | 0 | 0 |
| 2 | 4 | 3 | 3 | 3 | 2 | | | 0 | 0 |
| 2 | 43 | 2 | 2 | 3 | 1 | | 2 | 0 | 0 |
| 2 | 79 | 2 | 2 | 1 | 1 | 2,5 | | 0 | 0 |
| 2 | 29 | 2 | 1 | 1 | 2 | | | 0 | 0 |
| 2 | 11 | 2 | 2 | 1 | 2 | | | 0 | 0 |
| 2 | 15 | 2 | 1 | 1 | 2 | | | 0 | 0 |
| 2 | 10 | 2 | 2 | 1 | 2 | | | 0 | 0 |
| 2 | 12 | 2 | 1 | 1 | 2 | | | 0 | 0 |
| 2 | 93 | 2 | 2 | 1 | 1 | | 4 | 0 | 0 |
| 2 | 10 | 2 | 2 | 1 | 2 | | | 0 | 0 |
| 2 | 75 | 2 | 2 | 1 | 2 | | | 0 | 0 |
| 2 | 75 | 2 | 2 | 1 | 1 | | 2 | 0 | 0 |
| 2 | 75 | 2 | 2 | 1 | 1 | | 2 | 0 | 0 |
| 2 | 11 | 2 | 1 | 1 | 2 | | | 0 | 0 |
| 2 | 11 | 2 | 1 | 1 | 2 | | | 0 | 0 |

| | | | | | | | | | |
|---|---|---|---|---|---|---|---|---|---|
| 2 | 90 | 2 | 1 | 1 | 1 | 6,7 | | 1 | 0 |
| 2 | 131 | 2 | 2 | 1 | 1 | | 3 | 0 | 0 |
| 2 | 50 | 2 | 2 | 1 | 1 | | 2 | 0 | 0 |
| 2 | 11 | 2 | 1 | 1 | 2 | | | 0 | 0 |
| 2 | 19 | 3 | 1 | 2 | 2 | | | 0 | 0 |
| 2 | 12 | 3 | 1 | 1 | 2 | | | 0 | 0 |
| 2 | 12 | 3 | 1 | 1 | 2 | | | 0 | 0 |
| 2 | 17 | 3 | 2 | 2 | 2 | | | 0 | 0 |
| 2 | 13 | 3 | 2 | 1 | 2 | | | 0 | 0 |
| 2 | 11 | 3 | 1 | 1 | 2 | | | 0 | 0 |
| 2 | 10 | 3 | 2 | 1 | 2 | | | 0 | 0 |
| 2 | 53 | 3 | 2 | 1 | 1 | | 2 | 0 | 0 |
| 2 | 56 | 2 | 2 | 1 | 1 | 2,5 | | 0 | 0 |
| 2 | 9 | 2 | 1 | 1 | 2 | | | 0 | 0 |
| 2 | 8 | 2 | 2 | 1 | 2 | | | 0 | 0 |
| 2 | 8 | 2 | 1 | 1 | 2 | | | 0 | 0 |
| 2 | 47 | 2 | 2 | 1 | 1 | | 2 | 0 | 0 |
| 2 | 84 | 2 | 2 | 1 | 1 | | 2 | 0 | 0 |
| 2 | 11 | 2 | 2 | 1 | 2 | | | 0 | 0 |
| 2 | 10 | 2 | 1 | 1 | 2 | | | 0 | 0 |
| 2 | 9 | 2 | 2 | 1 | 2 | | | 0 | 0 |
| 2 | 12 | 2 | 2 | 1 | 2 | | | 0 | 0 |
| 2 | 14 | 2 | 1 | 1 | 2 | | | 0 | 0 |
| 2 | 83 | 2 | 2 | 1 | 1 | 2,4 | | 0 | 0 |
| 2 | 12 | 2 | 1 | 1 | 2 | | | 0 | 0 |
| 2 | 10 | 2 | 2 | 1 | 2 | | | 0 | 0 |
| 2 | 9 | 2 | 1 | 1 | 2 | | | 0 | 0 |
| 2 | 81 | 2 | 2 | 1 | 1 | 2,4 | | 0 | 0 |
| 2 | 11 | 2 | 1 | 1 | 2 | | | 0 | 0 |
| 2 | 10 | 2 | 2 | 1 | 2 | | | 0 | 0 |
| 2 | 11 | 2 | 1 | 1 | 2 | | | 0 | 0 |
| 2 | 10 | 2 | 2 | 1 | 2 | | | 0 | 0 |
| 2 | 12 | 2 | 1 | 1 | 2 | | | 0 | 0 |
| 2 | 307 | 2 | 1 | 3 | 2 | | | 0 | 0 |
| 2 | 92 | 2 | 1 | 2 | 2 | | | 0 | 0 |
| 2 | 177 | 2 | 2 | 3 | 1 | | 2 | 0 | 0 |
| 2 | 13 | 2 | 2 | 1 | 2 | | | 0 | 0 |
| 2 | 17 | 2 | 2 | 1 | 2 | | | 0 | 0 |
| 2 | 10 | 2 | 2 | 1 | 2 | | | 0 | 0 |
| 2 | 9 | 2 | 1 | 1 | 2 | | | 0 | 0 |
| 2 | 11 | 2 | 1 | 1 | 2 | | | 0 | 0 |
| 2 | 11 | 2 | 2 | 1 | 2 | | | 0 | 0 |
| 2 | 23 | 2 | 1 | 1 | 2 | | | 0 | 0 |
| 2 | 65 | 2 | 1 | 1 | 1 | | 5 | 0 | 0 |
| 2 | 14 | 2 | 2 | 1 | 2 | | | 0 | 0 |
| 2 | 80 | 2 | 2 | 1 | 1 | 2,5 | | 0 | 0 |
| 2 | 11 | 2 | 2 | 1 | 2 | | | 0 | 0 |
| 2 | 72 | 2 | 1 | 1 | 1 | | 5 | 0 | 0 |

| | | | | | | | | | |
|---|---|---|---|---|---|---|---|---|---|
| 2 | 10 | 2 | 1 | 1 | 2 | | | 0 | 0 |
| 2 | 71 | 2 | 2 | 1 | 1 | 2,6 | | 1 | 0 |
| 2 | 10 | 2 | 2 | 1 | 2 | | | 0 | 0 |
| 2 | 101 | 2 | 2 | 1 | 1 | 2,5 | | 0 | 0 |
| 2 | 36 | 2 | 1 | 1 | 2 | | | 0 | 0 |
| 2 | 47 | 2 | 1 | 1 | 2 | | | 0 | 0 |
| 2 | 47 | 2 | 2 | 1 | 2 | | | 0 | 0 |
| 2 | 55 | 2 | 1 | 1 | 2 | | | 0 | 0 |
| 2 | 66 | 2 | 1 | 1 | 2 | | | 0 | 0 |
| 2 | 58 | 2 | 1 | 1 | 2 | | | 0 | 0 |
| 2 | 36 | 2 | 1 | 1 | 2 | | | 0 | 0 |
| 2 | 130 | 2 | 1 | 1 | 2 | | | 0 | 0 |
| 2 | 57 | 2 | 2 | 1 | 2 | | | 0 | 0 |
| 2 | 74 | 2 | 1 | 1 | 2 | | | 0 | 0 |
| 2 | 87 | 2 | 1 | 1 | 2 | | | 0 | 0 |
| 2 | 37 | 2 | 2 | 1 | 2 | | | 0 | 0 |
| 2 | 127 | 2 | 1 | 1 | 2 | | | 0 | 0 |
| 2 | 44 | 2 | 1 | 1 | 2 | | | 0 | 0 |
| 2 | 332 | 2 | 1 | 1 | 2 | | | 0 | 0 |
| 2 | 68 | 2 | 1 | 1 | 2 | | | 0 | 0 |
| 2 | 191 | 2 | 1 | 1 | 2 | | | 0 | 0 |
| 2 | 56 | 2 | 2 | 1 | 2 | | | 0 | 0 |
| 2 | 324 | 2 | 2 | 1 | 1 | | 11 | 0 | 0 |
| 2 | 457 | 2 | 1 | 1 | 2 | | | 0 | 0 |
| 2 | 122 | 2 | 1 | 1 | 2 | | | 0 | 0 |
| 2 | 155 | 2 | 2 | 1 | 2 | | | 0 | 0 |
| 2 | 45 | 2 | 1 | 1 | 2 | | | 0 | 0 |
| 2 | 320 | 2 | 2 | 1 | 3 | | 11 | 0 | 0 |
| 2 | 168 | 2 | 1 | 1 | 1 | | 9 | 0 | 1 |
| 2 | 67 | 2 | 1 | 1 | 2 | | | 0 | 0 |
| 2 | 112 | 2 | 1 | 1 | 2 | | | 0 | 0 |
| 2 | 99 | 2 | 1 | 1 | 2 | | | 0 | 0 |
| 2 | 78 | 2 | 1 | 1 | 2 | | | 0 | 0 |
| 2 | 333 | 2 | 2 | 3 | 2 | | | 0 | 0 |
| 2 | 72 | 2 | 1 | 1 | 2 | | | 0 | 0 |
| 2 | 44 | 2 | 2 | 1 | 2 | | | 0 | 0 |
| 2 | 38 | 2 | 1 | 1 | 2 | | | 0 | 0 |
| 2 | 148 | 2 | 1 | 2 | 1 | 4,8,9 | | 0 | 1 |
| 2 | 129 | 2 | 1 | 1 | 2 | | | 0 | 0 |
| 2 | 116 | 2 | 1 | 1 | 2 | | | 0 | 0 |
| 2 | 35 | 2 | 1 | 1 | 2 | | | 0 | 0 |
| 2 | 81 | 2 | 3 | 1 | 2 | | | 0 | 0 |
| 2 | 118 | 2 | 1 | 1 | 2 | | | 0 | 0 |
| 2 | 90 | 2 | 1 | 2 | 2 | | | 0 | 0 |
| 2 | 35 | 2 | 1 | 2 | 2 | | | 0 | 0 |
| 2 | 76 | 2 | 1 | 1 | 2 | | | 0 | 0 |
| 2 | 249 | 2 | 1 | 1 | 1 | | 9 | 0 | 1 |
| 2 | 90 | 2 | 1 | 1 | 2 | | | 0 | 0 |

| | | | | | | | | | |
|---|---|---|---|---|---|---|---|---|---|
| 2 | 251 | 2 | 1 | 2 | 1 | | 4 | 0 | 0 |
| 2 | 49 | 2 | 1 | 1 | 2 | | | 0 | 0 |
| 2 | 373 | 2 | 1 | 1 | 1 | | 9 | 0 | 1 |
| 2 | 51 | 2 | 1 | 1 | 2 | | | 0 | 0 |
| 2 | 207 | 2 | 1 | 1 | 2 | | | 0 | 0 |
| 2 | 45 | 2 | 1 | 1 | 2 | | | 0 | 0 |
| 2 | 64 | 2 | 1 | 1 | 2 | | | 0 | 0 |
| 2 | 62 | 2 | 1 | 1 | 2 | | | 0 | 0 |
| 2 | 95 | 2 | 1 | 1 | 2 | | | 0 | 0 |
| 2 | 150 | 2 | 2 | 1 | 2 | | | 0 | 0 |
| 2 | 80 | 2 | 1 | 1 | 2 | | | 0 | 0 |
| 2 | 196 | 2 | 1 | 1 | 2 | | | 0 | 0 |
| 2 | 39 | 2 | 1 | 1 | 2 | | | 0 | 0 |
| 2 | 72 | 2 | 1 | 2 | 2 | | | 0 | 0 |
| 2 | 91 | 2 | 1 | 1 | 2 | | | 0 | 0 |
| 2 | 82 | 2 | 1 | 1 | 2 | | | 0 | 0 |
| 2 | 95 | 2 | 1 | 1 | 2 | | | 0 | 0 |
| 2 | 426 | 2 | 1 | 1 | 3 | 8,9 | | 0 | 1 |
| 2 | 38 | 2 | 1 | 2 | 2 | | | 0 | 0 |
| 2 | 112 | 2 | 2 | 1 | 2 | | | 0 | 0 |
| 2 | 79 | 2 | 1 | 1 | 2 | | | 0 | 0 |
| 2 | 71 | 2 | 1 | 1 | 2 | | | 0 | 0 |
| 2 | 142 | 2 | 1 | 1 | 2 | | | 0 | 0 |
| 2 | 92 | 2 | 1 | 1 | 2 | | | 0 | 0 |
| 2 | 100 | 2 | 2 | 1 | 2 | | | 0 | 0 |
| 2 | 13 | 2 | 1 | 1 | 2 | | | 0 | 0 |
| 2 | 35 | 2 | 1 | 1 | 2 | | | 0 | 0 |
| 2 | 86 | 2 | 1 | 1 | 2 | | | 0 | 0 |
| 2 | 77 | 2 | 1 | 1 | 2 | | | 0 | 0 |
| 2 | 278 | 2 | 1 | 1 | 1 | 9,11 | | 0 | 1 |
| 2 | 90 | 2 | 1 | 1 | 2 | | | 0 | 0 |
| 2 | 85 | 2 | 1 | 1 | 2 | | | 0 | 0 |
| 2 | 228 | 2 | 1 | 1 | 2 | | | 0 | 0 |
| 2 | 194 | 2 | 2 | 1 | 2 | | | 0 | 0 |
| 2 | 75 | 2 | 1 | 1 | 2 | | | 0 | 0 |
| 2 | 47 | 2 | 1 | 1 | 2 | | | 0 | 0 |
| 2 | 582 | 2 | 1 | 1 | 2 | | | 0 | 0 |
| 2 | 49 | 2 | 1 | 1 | 2 | | | 0 | 0 |
| 2 | 72 | 2 | 1 | 1 | 2 | | | 0 | 0 |
| 2 | 170 | 2 | 1 | 1 | 2 | | | 0 | 0 |
| 2 | 106 | 2 | 1 | 1 | 2 | | | 0 | 0 |
| 2 | 111 | 2 | 1 | 1 | 2 | | | 0 | 0 |
| 2 | 75 | 2 | 2 | 1 | 2 | | | 0 | 0 |
| 2 | 83 | 2 | 1 | 1 | 2 | | | 0 | 0 |
| 2 | 30 | 2 | 1 | 1 | 2 | | | 0 | 0 |
| 2 | 387 | 2 | 1 | 1 | 1 | | 9 | 0 | 1 |
| 2 | 286 | 2 | 1 | 2 | 2 | | | 0 | 0 |
| 2 | 138 | 2 | 2 | 1 | 2 | | | 0 | 0 |

| | | | | | | | | | |
|---|---|---|---|---|---|---|---|---|---|
| 2 | 47 | 2 | 1 | 1 | 2 | | | 0 | 0 |
| 2 | 56 | 2 | 1 | 1 | 2 | | | 0 | 0 |
| 2 | 187 | 2 | 1 | 1 | 2 | | | 0 | 0 |
| 2 | 118 | 2 | 1 | 1 | 2 | | | 0 | 0 |
| 2 | 159 | 2 | 1 | 1 | 2 | | | 0 | 0 |
| 2 | 39 | 2 | 2 | 1 | 2 | | | 0 | 0 |
| 2 | 88 | 2 | 1 | 1 | 2 | | | 0 | 0 |
| 2 | 109 | 2 | 1 | 1 | 2 | | | 0 | 0 |
| 2 | 120 | 2 | 1 | 1 | 2 | | | 0 | 0 |
| 2 | 84 | 2 | 1 | 1 | 2 | | | 0 | 0 |
| 2 | 1510 | 2 | 1 | 1 | 2 | | | 0 | 0 |
| 2 | 99 | 2 | 2 | 1 | 2 | | | 0 | 0 |
| 2 | 252 | 2 | 1 | 1 | 1 | 1,8 | | 0 | 0 |
| 2 | 458 | 2 | 1 | 1 | 2 | | | 0 | 0 |
| 2 | 813 | 2 | 2 | 1 | 1 | | 8 | 0 | 0 |
| 2 | 142 | 2 | 1 | 1 | 2 | | | 0 | 0 |
| 2 | 39 | 2 | 1 | 1 | 2 | | | 0 | 0 |
| 2 | 99 | 2 | 2 | 1 | 2 | | | 0 | 0 |
| 2 | 97 | 2 | 1 | 1 | 2 | | | 0 | 0 |
| 2 | 154 | 2 | 1 | 1 | 2 | | | 0 | 0 |
| 2 | 190 | 2 | 2 | 1 | 2 | | | 0 | 0 |
| 2 | 110 | 2 | 2 | 1 | 2 | | | 0 | 0 |
| 2 | 126 | 2 | 1 | 1 | 2 | | | 0 | 0 |
| 2 | 379 | 2 | 1 | 1 | 2 | | | 0 | 0 |
| 2 | 77 | 2 | 1 | 1 | 2 | | | 0 | 0 |
| 2 | 64 | 2 | 2 | 1 | 2 | | | 0 | 0 |
| 2 | 76 | 2 | 1 | 1 | 2 | | | 0 | 0 |
| 2 | 68 | 2 | 1 | 1 | 2 | | | 0 | 0 |
| 2 | 88 | 2 | 1 | 1 | 2 | | | 0 | 0 |
| 2 | 141 | 2 | 1 | 1 | 2 | | | 0 | 0 |
| 2 | 37 | 2 | 1 | 1 | 2 | | | 0 | 0 |
| 2 | 29 | 2 | 1 | 1 | 2 | | | 0 | 0 |
| 2 | 35 | 2 | 1 | 1 | 2 | | | 0 | 0 |
| 2 | 77 | 2 | 1 | 1 | 2 | | | 0 | 0 |
| 2 | 42 | 2 | 2 | 1 | 2 | | | 0 | 0 |
| 2 | 47 | 2 | 1 | 1 | 2 | | | 0 | 0 |
| 2 | 213 | 2 | 1 | 1 | 2 | | | 0 | 0 |
| 2 | 99 | 2 | 2 | 1 | 2 | | | 0 | 0 |
| 2 | 46 | 2 | 1 | 1 | 2 | | | 0 | 0 |
| 2 | 192 | 2 | 1 | 2 | 2 | | | 0 | 0 |
| 2 | 180 | 2 | 2 | 1 | 1 | | 4 | 0 | 0 |
| 2 | 84 | 2 | 2 | 1 | 2 | | | 0 | 0 |
| 2 | 92 | 2 | 2 | 1 | 2 | | | 0 | 0 |
| 2 | 77 | 2 | 1 | 1 | 2 | | | 0 | 0 |
| 2 | 265 | 2 | 1 | 1 | 2 | | | 0 | 0 |
| 2 | 74 | 2 | 1 | 1 | 2 | | | 0 | 0 |
| 2 | 73 | 2 | 1 | 1 | 2 | | | 0 | 0 |
| 2 | 1058 | 2 | 1 | 1 | 2 | | | 0 | 0 |

| | | | | | | | | |
|---|---|---|---|---|---|---|---|---|
| 2 | 59 | 2 | 1 | 1 | 2 | | 0 | 0 |
| 2 | 120 | 2 | 1 | 1 | 2 | | 0 | 0 |
| 2 | 167 | 2 | 2 | 1 | 1 | 5 | 0 | 0 |
| 2 | 128 | 2 | 1 | 1 | 2 | | 0 | 0 |
| 2 | 301 | 2 | 1 | 1 | 2 | | 0 | 0 |
| 2 | 169 | 2 | 1 | 1 | 3 | 9 | 0 | 1 |
| 2 | 90 | 2 | 1 | 1 | 2 | | 0 | 0 |
| 2 | 197 | 2 | 1 | 1 | 2 | | 0 | 0 |
| 2 | 14 | 2 | 2 | 1 | 2 | | 0 | 0 |
| 2 | 65 | 2 | 2 | 1 | 1 | 5 | 0 | 0 |
| 2 | 12 | 2 | 1 | 1 | 2 | | 0 | 0 |
| 2 | 385 | 2 | 1 | 1 | 1 | 11 | 0 | 0 |
| 2 | 104 | 2 | 2 | 1 | 1 | 11 | 0 | 0 |
| 2 | 50 | 2 | 1 | 1 | 2 | | 0 | 0 |
| 2 | 191 | 2 | 2 | 2 | 2 | | 0 | 0 |
| 2 | 35 | 2 | 2 | 1 | 2 | | 0 | 0 |
| 2 | 156 | 2 | 1 | 1 | 1 | 5 | 0 | 0 |
| 2 | 90 | 2 | 1 | 1 | 2 | | 0 | 0 |
| 2 | 166 | 2 | 1 | 1 | 2 | | 0 | 0 |
| 2 | 74 | 2 | 1 | 1 | 2 | | 0 | 0 |
| 2 | 73 | 2 | 2 | 1 | 2 | | 0 | 0 |
| 2 | 208 | 2 | 2 | 1 | 2 | | 0 | 0 |
| 2 | 152 | 2 | 1 | 2 | 2 | | 0 | 0 |
| 2 | 93 | 2 | 1 | 1 | 2 | | 0 | 0 |
| 2 | 990 | 2 | 1 | 1 | 2 | | 0 | 0 |
| 2 | 222 | 2 | 1 | 1 | 2 | | 0 | 0 |
| 2 | 208 | 2 | 2 | 1 | 2 | | 0 | 0 |
| 2 | 97 | 2 | 1 | 1 | 2 | | 0 | 0 |
| 2 | 49 | 2 | 1 | 1 | 2 | | 0 | 0 |
| 2 | 109 | 2 | 1 | 1 | 2 | | 0 | 0 |
| 2 | 31 | 2 | 1 | 1 | 2 | | 0 | 0 |
| 2 | 142 | 2 | 1 | 1 | 2 | | 0 | 0 |
| 2 | 55 | 2 | 2 | 1 | 2 | | 0 | 0 |
| 2 | 267 | 2 | 1 | 1 | 2 | | 0 | 0 |
| 2 | 188 | 2 | 1 | 1 | 2 | | 0 | 0 |
| 2 | 27 | 2 | 1 | 1 | 2 | | 0 | 0 |
| 2 | 154 | 2 | 1 | 1 | 2 | | 0 | 0 |
| 2 | 515 | 2 | 2 | 1 | 2 | | 0 | 0 |
| 2 | 265 | 2 | 1 | 1 | 2 | | 0 | 0 |
| 2 | 237 | 2 | 2 | 1 | 2 | | 0 | 0 |
| 2 | 97 | 2 | 2 | 1 | 2 | | 0 | 0 |
| 2 | 79 | 2 | 1 | 1 | 2 | | 0 | 0 |
| 2 | 57 | 2 | 2 | 1 | 2 | | 0 | 0 |
| 2 | 60 | 2 | 1 | 1 | 2 | | 0 | 0 |
| 2 | 66 | 2 | 1 | 1 | 2 | | 0 | 0 |
| 2 | 95 | 2 | 1 | 1 | 2 | | 0 | 0 |
| 2 | 91 | 2 | 1 | 1 | 2 | | 0 | 0 |
| 2 | 77 | 2 | 1 | 1 | 2 | | 0 | 0 |

| | | | | | | | | | |
|---|---|---|---|---|---|---|---|---|---|
| 2 | 18 | 2 | 2 | 1 | 2 | | | 0 | 0 |
| 2 | 10 | 2 | 2 | 1 | 2 | | | 0 | 0 |
| 2 | 37 | 2 | 2 | 1 | 2 | | | 0 | 0 |
| 2 | 111 | 2 | 2 | 1 | 1 | | 2 | 0 | 0 |
| 2 | 163 | 2 | 2 | 1 | 1 | 5,9 | | 0 | 1 |
| 2 | 65 | 2 | 2 | 1 | 1 | 5,9 | | 0 | 1 |
| 2 | 117 | 2 | 1 | 1 | 2 | | | 0 | 0 |
| 2 | 72 | 2 | 1 | 1 | 2 | | | 0 | 0 |
| 2 | 71 | 2 | 1 | 1 | 2 | | | 0 | 0 |
| 2 | 137 | 2 | 1 | 1 | 2 | | | 0 | 0 |
| 2 | 113 | 2 | 1 | 1 | 2 | | | 0 | 0 |
| 2 | 101 | 2 | 1 | 1 | 2 | | | 0 | 0 |
| 2 | 212 | 2 | 1 | 1 | 2 | | | 0 | 0 |
| 2 | 117 | 2 | 1 | 1 | 3 | | | 0 | 0 |
| 2 | 170 | 2 | 1 | 1 | 2 | | | 0 | 0 |
| 2 | 156 | 2 | 1 | 1 | 2 | | | 0 | 0 |
| 2 | 32 | 2 | 2 | 1 | 2 | | | 0 | 0 |
| 2 | 88 | 2 | 1 | 1 | 2 | | | 0 | 0 |
| 2 | 26 | 2 | 3 | 3 | 2 | | | 0 | 0 |
| 2 | 14 | 2 | 2 | 1 | 2 | | | 0 | 0 |
| 2 | 13 | 2 | 2 | 1 | 2 | | | 0 | 0 |
| 2 | 203 | 2 | 2 | 1 | 1 | 2,9 | | 0 | 1 |
| 2 | 104 | 2 | 2 | 1 | 1 | 5,9 | | 0 | 1 |
| 2 | 70 | 2 | 2 | 1 | 1 | 5,9 | | 0 | 1 |
| 2 | 232 | 2 | 2 | 1 | 1 | 5,9 | | 0 | 1 |
| 2 | 65 | 2 | 2 | 1 | 1 | 2,9 | | 0 | 1 |
| 2 | 80 | 2 | 2 | 1 | 1 | | 3 | 0 | 0 |
| 2 | 147 | 2 | 2 | 1 | 1 | 2,8 | | 0 | 0 |
| 2 | 152 | 2 | 2 | 1 | 1 | 5,9 | | 0 | 1 |
| 2 | 46 | 2 | 2 | 1 | 1 | 2,9 | | 0 | 1 |
| 2 | 74 | 2 | 3 | 1 | 1 | 2,9 | | 0 | 1 |
| 2 | 214 | 2 | 1 | 1 | 2 | | | 0 | 0 |
| 2 | 58 | 2 | 1 | 1 | 2 | | | 0 | 0 |
| 2 | 16 | 2 | 2 | 1 | 2 | | | 0 | 0 |
| 2 | 14 | 2 | 2 | 1 | 2 | | | 0 | 0 |
| 2 | 136 | 2 | 2 | 1 | 1 | 3,4 | | 0 | 0 |
| 2 | 11 | 2 | 2 | 1 | 2 | | | 0 | 0 |
| 2 | 69 | 2 | 1 | 1 | 1 | 5,8,9 | | 0 | 1 |
| 2 | 12 | 2 | 1 | 1 | 2 | | | 0 | 0 |
| 2 | 12 | 2 | 2 | 1 | 2 | | | 0 | 0 |
| 2 | 11 | 2 | 2 | 1 | 2 | | | 0 | 0 |
| 2 | 249 | 2 | 2 | 1 | 2 | | | 0 | 0 |
| 2 | 254 | 2 | 1 | 1 | 2 | | | 0 | 0 |
| 2 | 65 | 2 | 1 | 2 | 2 | | | 0 | 0 |
| 2 | 115 | 2 | 1 | 1 | 2 | | | 0 | 0 |
| 2 | 168 | 2 | 2 | 3 | 2 | | | 0 | 0 |
| 2 | 59 | 2 | 1 | 1 | 2 | | | 0 | 0 |
| 2 | 62 | 2 | 1 | 1 | 2 | | | 0 | 0 |

| | | | | | | | | | |
|---|---|---|---|---|---|---|---|---|---|
| 2 | 234 | 2 | 1 | 3 | 2 | | | 0 | 0 |
| 2 | 50 | 2 | 1 | 1 | 2 | | | 0 | 0 |
| 2 | 83 | 2 | 2 | 1 | 1 | 5,9 | | 0 | 1 |
| 2 | 14 | 2 | 1 | 1 | 2 | | | 0 | 0 |
| 2 | 87 | 2 | 2 | 1 | 1 | | 4 | 0 | 0 |
| 2 | 90 | 2 | 1 | 1 | 2 | | | 0 | 0 |
| 2 | 188 | 2 | 1 | 1 | 2 | | | 0 | 0 |
| 2 | 125 | 2 | 1 | 1 | 2 | | | 0 | 0 |
| 2 | 50 | 2 | 2 | 1 | 1 | 5,9 | | 0 | 1 |
| 2 | 9 | 2 | 1 | 1 | 2 | | | 0 | 0 |
| 2 | 11 | 2 | 2 | 1 | 2 | | | 0 | 0 |
| 2 | 17 | 2 | 2 | 1 | 2 | | | 0 | 0 |
| 2 | 78 | 2 | 1 | 1 | 1 | | 3 | 0 | 0 |
| 2 | 66 | 2 | 2 | 1 | 2 | | | 0 | 0 |
| 2 | 51 | 2 | 2 | 1 | 2 | | | 0 | 0 |
| 2 | 20 | 2 | 1 | 1 | 2 | | | 0 | 0 |
| 2 | 13 | 2 | 2 | 1 | 2 | | | 0 | 0 |
| 2 | 88 | 2 | 1 | 1 | 1 | | 2 | 0 | 0 |
| 2 | 90 | 2 | 2 | 1 | 1 | 2,9,10 | | 0 | 1 |
| 2 | 79 | 2 | 2 | 1 | 1 | 3, | | 0 | 0 |
| 2 | 34 | 2 | 2 | 1 | 1 | | 4 | 0 | 0 |
| 2 | 9 | 2 | 1 | 1 | 2 | | | 0 | 0 |
| 2 | 10 | 2 | 1 | 1 | 2 | | | 0 | 0 |
| 2 | 9 | 2 | 1 | 1 | 2 | | | 0 | 0 |
| 2 | 12 | 2 | 1 | 1 | 2 | | | 0 | 0 |
| 2 | 9 | 2 | 1 | 1 | 2 | | | 0 | 0 |
| 2 | 11 | 2 | 1 | 1 | 2 | | | 0 | 0 |
| 2 | 8 | 2 | 1 | 1 | 2 | | | 0 | 0 |
| 2 | 79 | 2 | 2 | 1 | 1 | 5,9 | | 0 | 1 |
| 2 | 8 | 2 | 1 | 1 | 2 | | | 0 | 0 |
| 2 | 8 | 2 | 1 | 1 | 2 | | | 0 | 0 |
| 2 | 9 | 2 | 1 | 1 | 2 | | | 0 | 0 |
| 2 | 57 | 2 | 2 | 1 | 1 | 5,6,9 | | 1 | 1 |
| 2 | 61 | 2 | 1 | 1 | 1 | 5,9 | | 0 | 1 |
| 2 | 8 | 2 | 1 | 1 | 2 | | | 0 | 0 |
| 2 | 8 | 2 | 2 | 1 | 2 | | | 0 | 0 |
| 2 | 7 | 2 | 2 | 1 | 2 | | | 0 | 0 |
| 2 | 8 | 2 | 1 | 1 | 2 | | | 0 | 0 |
| 2 | 8 | 2 | 1 | 1 | 2 | | | 0 | 0 |
| 2 | 8 | 2 | 2 | 1 | 1 | | | 0 | 0 |
| 2 | 13 | 2 | 2 | 1 | 2 | | | 0 | 0 |
| 2 | 7 | 2 | 2 | 1 | 2 | | | 0 | 0 |
| 2 | 9 | 2 | 2 | 1 | 2 | | | 0 | 0 |
| 2 | 10 | 2 | 2 | 1 | 2 | | | 0 | 0 |
| 2 | 10 | 2 | 2 | 1 | 2 | | | 0 | 0 |
| 2 | 8 | 2 | 2 | 1 | 2 | | | 0 | 0 |
| 2 | 8 | 2 | 2 | 1 | 2 | | | 0 | 0 |
| 2 | 7 | 2 | 2 | 1 | 2 | | | 0 | 0 |

| | | | | | | | |
|---|---|---|---|---|---|---|---|
| 2 | 9 | 2 | 2 | 1 | 2 | 0 | 0 |
| 2 | 10 | 2 | 2 | 1 | 2 | 0 | 0 |
| 2 | 8 | 2 | 2 | 1 | 2 | 0 | 0 |
| 2 | 9 | 2 | 2 | 1 | 2 | 0 | 0 |
| 2 | 59 | 2 | 1 | 1 | 2 | 0 | 0 |
| 2 | 40 | 2 | 2 | 1 | 2 | 0 | 0 |
| 2 | 30 | 2 | 1 | 1 | 2 | 0 | 0 |
| 2 | 302 | 2 | 1 | 3 | 2 | 0 | 0 |
| 2 | 52 | 2 | 1 | 1 | 2 | 0 | 0 |
| 2 | 58 | 2 | 1 | 1 | 2 | 0 | 0 |
| 2 | 62 | 2 | 1 | 1 | 2 | 0 | 0 |
| 2 | 81 | 2 | 1 | 1 | 2 | 0 | 0 |
| 2 | 276 | 2 | 2 | 1 | 1 | 0 | 1 |
| 2 | 99 | 2 | 1 | 1 | 2 | 0 | 0 |
| 2 | 102 | 2 | 1 | 3 | 1 | 0 | 0 |
| 2 | 122 | 2 | 1 | 1 | 2 | 0 | 0 |
| 2 | 251 | 2 | 2 | 1 | 1 | 0 | 1 |
| 2 | 100 | 2 | 1 | 1 | 2 | 0 | 0 |
| 2 | 195 | 2 | 1 | 1 | 1 | 0 | 0 |
| 2 | 111 | 2 | 1 | 1 | 2 | 0 | 0 |
| 2 | 76 | 2 | 1 | 1 | 2 | 0 | 0 |
| 2 | 95 | 2 | 2 | 1 | 2 | 0 | 0 |
| 2 | 362 | 2 | 2 | 1 | 1 | 0 | 0 |
| 2 | 211 | 2 | 2 | 1 | 2 | 0 | 0 |
| 2 | 118 | 2 | 2 | 1 | 1 | 0 | 0 |
| 2 | 513 | 2 | 2 | 1 | 1 | 0 | 0 |
| 2 | 84 | 2 | 2 | 1 | 2 | 0 | 0 |
| 2 | 180 | 2 | 1 | 1 | 1 | 0 | 1 |
| 2 | 329 | 2 | 2 | 1 | 1 | 0 | 1 |
| 2 | 274 | 2 | 1 | 1 | 1 | 0 | 1 |
| 2 | 139 | 2 | 1 | 1 | 2 | 0 | 0 |
| 2 | 84 | 2 | 2 | 1 | 2 | 0 | 0 |
| 2 | 113 | 2 | 1 | 1 | 2 | 0 | 0 |
| 2 | 181 | 2 | 1 | 1 | 1 | 0 | 0 |
| 2 | 260 | 2 | 2 | 1 | 1 | 0 | 0 |
| 2 | 144 | 2 | 2 | 1 | 2 | 0 | 0 |
| 2 | 251 | 2 | 2 | 1 | 1 | 0 | 0 |
| 2 | 198 | 2 | 2 | 1 | 2 | 0 | 0 |
| 2 | 264 | 2 | 1 | 1 | 1 | 0 | 1 |
| 2 | 23360 | 1 | 2 | 1 | 2 | 0 | 0 |
| 2 | 155 | 1 | 1 | 2 | 1 | 0 | 0 |
| 2 | 301 | 2 | 2 | 1 | 1 | 0 | 0 |
| 2 | 276 | 2 | 2 | 1 | 1 | 0 | 0 |
| 2 | 264 | 2 | 1 | 1 | 1 | 0 | 0 |

| | | | | |
|---|---|---|---|---|
| | code: 1 preview | 1 female | 1 18-25 | 1 yes |
| 2 UAE | 2 anonymous | 2 male | 2 26-35 | 2 no |
| | 3 social | 3 not specified | 3 above | 3 not sure |

| B6 | ritalin4 | adderall3 | coffe | modafinil | singleMultiple | nationality | degree |
|---|---|---|---|---|---|---|---|
| 0 | 0 | 0 | 0 | 0 | | 6 | 1 |
| 0 | 0 | 0 | 0 | 0 | | 5 | 4 |
| 1 | 0 | 0 | 0 | 0 | 2 | 5 | 3 |
| 0 | 0 | 0 | 0 | 0 | | 4 | 3 |
| 0 | 0 | 0 | 0 | 0 | | 8 | 6 |
| 0 | 0 | 0 | 0 | 0 | | 8 | 6 |
| 0 | 0 | 0 | 0 | 0 | | 6 | 1 |
| 0 | 0 | 0 | 0 | 0 | | 8 | 6 |
| 0 | 0 | 0 | 0 | 0 | | 8 | 6 |
| 0 | 0 | 0 | 0 | 0 | | 5 | 1 |
| 0 | 0 | 0 | 0 | 0 | | 8 | 6 |
| 0 | 0 | 0 | 0 | 0 | | 8 | 6 |
| 0 | 0 | 0 | 0 | 0 | | 7 | 1 |
| 0 | 0 | 0 | 0 | 0 | | 8 | 6 |
| 0 | 0 | 0 | 0 | 0 | | 8 | 6 |
| 0 | 0 | 0 | 0 | 0 | | 8 | 6 |
| 0 | 0 | 0 | 0 | 0 | | 8 | 6 |
| 0 | 0 | 0 | 0 | 0 | | 8 | 6 |
| 0 | 0 | 0 | 0 | 0 | | 4 | 1 |
| 0 | 0 | 0 | 0 | 0 | 1 | 5 | 1 |
| 0 | 0 | 0 | 0 | 0 | | 4 | 4 |
| 0 | 0 | 0 | 0 | 0 | | 5 | 3 |
| 0 | 0 | 0 | 0 | 0 | | 5 | 2 |
| 0 | 0 | 0 | 0 | 0 | | 5 | 3 |
| 0 | 0 | 0 | 0 | 0 | | 4 | 1 |
| 0 | 0 | 0 | 0 | 0 | | 8 | 6 |
| 0 | 0 | 0 | 1 | 0 | 1 | 6 | 1 |
| 0 | 0 | 0 | 0 | 0 | | 6 | 4 |
| 0 | 0 | 0 | 0 | 0 | | 4 | 6 |
| 0 | 0 | 0 | 0 | 0 | | 8 | 6 |
| 1 | 0 | 0 | 1 | 0 | 2 | 5 | 1 |
| 0 | 0 | 0 | 0 | 0 | | 8 | 6 |
| 0 | 0 | 0 | 0 | 0 | | 8 | 6 |
| 0 | 0 | 0 | 0 | 0 | | 8 | 6 |
| 0 | 0 | 0 | 0 | 0 | 1 | 5 | 1 |
| 0 | 0 | 0 | 0 | 0 | | 5 | 4 |
| 0 | 0 | 0 | 0 | 0 | | 6 | 1 |
| 0 | 0 | 0 | 0 | 0 | | 8 | 6 |
| 0 | 0 | 0 | 0 | 0 | | 8 | 6 |
| 0 | 0 | 0 | 0 | 0 | | 8 | 6 |
| 0 | 0 | 0 | 0 | 0 | | 6 | 1 |
| 0 | 0 | 0 | 0 | 0 | | 8 | 6 |
| 0 | 0 | 0 | 0 | 0 | | 1 | 2 |
| 0 | 0 | 0 | 0 | 0 | | 8 | 6 |
| 0 | 0 | 0 | 1 | 0 | 1 | 1 | 2 |
| 0 | 0 | 0 | 0 | 0 | | 8 | 6 |

| | | | | | | | |
|---|---|---|---|---|---|---|---|
| 0 | 0 | 0 | 0 | 0 | | 8 | 6 |
| 1 | 0 | 0 | 1 | 0 | 2 | 1 | 2 |
| 0 | 0 | 0 | 0 | 0 | | 8 | 6 |
| 0 | 0 | 0 | 1 | 0 | 1 | 4 | 2 |
| 0 | 0 | 0 | | 0 | | 8 | 6 |
| 0 | 0 | 0 | 0 | 0 | | 8 | 6 |
| 0 | 0 | 0 | 0 | 0 | | 1 | 2 |
| 0 | 0 | 0 | 0 | 0 | | 8 | 6 |
| 1 | 0 | 0 | 0 | 0 | 2 | 1 | 2 |
| 0 | 0 | 0 | 0 | 0 | | 8 | 6 |
| 0 | 0 | 0 | 0 | 0 | | 1 | 2 |
| 0 | 0 | 0 | 0 | 0 | 1 | 1 | 2 |
| 0 | 0 | 0 | 0 | 0 | | 8 | 6 |
| 0 | 1 | 0 | 0 | 0 | 1 | 6 | 1 |
| 0 | 0 | 0 | 0 | 0 | 2 | 6 | 1 |
| 0 | 0 | 0 | 0 | 0 | | 5 | 3 |
| 0 | 0 | 0 | 0 | 0 | | 8 | 6 |
| 0 | 0 | 0 | 0 | 0 | | 8 | 6 |
| 0 | 0 | 0 | 1 | 0 | 1 | 1 | 2 |
| 0 | 0 | 0 | 0 | 1 | 1 | 4 | 1 |
| 0 | 0 | 0 | 0 | 0 | | 8 | 6 |
| 0 | 0 | 0 | 0 | 0 | | 5 | 3 |
| 0 | 0 | 0 | 0 | 0 | | 8 | 6 |
| 0 | 0 | 0 | 0 | 0 | | 8 | 6 |
| 0 | 0 | 0 | 0 | 0 | | 8 | 6 |
| 0 | 0 | 0 | 0 | 0 | | 4 | 1 |
| 0 | 0 | 0 | 0 | 0 | | 8 | 6 |
| 0 | 0 | 0 | 0 | 0 | | 8 | 6 |
| 0 | 0 | 0 | 0 | 0 | | 8 | 6 |
| 0 | 0 | 0 | 0 | 0 | | 8 | 6 |
| 0 | 0 | 0 | 0 | 0 | | 1 | 1 |
| 0 | 0 | 0 | 0 | 1 | 2 | 4 | 1 |
| 0 | 0 | 1 | 0 | 0 | 1 | 4 | 1 |
| 0 | 0 | 0 | 1 | 0 | 1 | 4 | 1 |
| 0 | 0 | 0 | 1 | 0 | 1 | 4 | 1 |
| 0 | 0 | 0 | 0 | 0 | | 8 | 6 |
| 0 | 0 | 0 | 1 | 0 | 2 | 4 | 1 |
| 0 | 0 | 0 | 0 | 1 | 1 | 4 | 1 |
| 0 | 0 | 0 | 1 | 1 | 2 | 4 | 1 |
| 0 | 0 | 0 | 1 | 1 | 2 | 4 | 1 |
| 0 | 0 | 0 | 0 | 1 | 2 | 4 | 1 |
| 0 | 0 | 0 | 0 | 0 | 1 | 5 | 1 |
| 0 | 0 | 0 | 0 | 1 | 1 | 5 | 2 |
| 0 | 0 | 0 | 0 | 1 | 2 | 4 | 2 |
| 0 | 0 | 0 | 0 | 1 | 2 | 4 | 1 |
| 0 | 0 | 0 | 1 | 0 | 1 | 4 | 1 |
| 0 | 0 | 0 | 0 | 0 | | 4 | 6 |
| 0 | 0 | 0 | 1 | 1 | 2 | 5 | 2 |

| | | | | | | | |
|---|---|---|---|---|---|---|---|
| 0 | 0 | 0 | 1 | 1 | 2 | 4 | 1 |
| 0 | 0 | 0 | 1 | 0 | 2 | 4 | 1 |
| 0 | 0 | 0 | 1 | 1 | 2 | 4 | 1 |
| 0 | 0 | 0 | 1 | 0 | 1 | 4 | 1 |
| 0 | 0 | 0 | 0 | 0 | | 8 | 6 |
| 0 | 0 | 0 | 0 | 1 | 2 | 4 | 1 |
| 0 | 0 | 0 | 0 | 1 | 1 | 5 | 2 |
| 0 | 0 | 1 | 0 | 0 | 1 | 5 | 1 |
| 0 | 0 | 0 | 0 | 0 | | 8 | 6 |
| 0 | 0 | 0 | 0 | 0 | | 8 | 6 |
| 0 | 0 | 0 | 1 | 1 | 2 | 4 | 2 |
| 0 | 0 | 1 | 0 | 0 | 1 | 4 | 1 |
| 0 | 0 | 0 | 0 | 0 | | 8 | 6 |
| 0 | 1 | 1 | 0 | 0 | 2 | 4 | 1 |
| 0 | 0 | 0 | 0 | 1 | 2 | 4 | 1 |
| 0 | 0 | 0 | 0 | 1 | 2 | 4 | 1 |
| 0 | 0 | 0 | 1 | 1 | 2 | 4 | 1 |
| 0 | 1 | 1 | 0 | 0 | 2 | 5 | 1 |
| 0 | 0 | 0 | 0 | 0 | | 8 | 6 |
| 0 | 0 | 0 | 0 | 0 | | 8 | 6 |
| 0 | 0 | 0 | 1 | 1 | 2 | 4 | 1 |
| 0 | 0 | 0 | 0 | 0 | | 8 | 6 |
| 0 | 0 | 0 | 0 | 0 | | 8 | 6 |
| 0 | 1 | 1 | 0 | 0 | 2 | 4 | 1 |
| 0 | 0 | 0 | 0 | 0 | | 8 | 6 |
| 0 | 0 | 0 | 0 | 0 | | 8 | 6 |
| 0 | 0 | 0 | 0 | 0 | | 8 | 6 |
| 0 | 0 | 0 | 0 | 0 | | 8 | 6 |
| 0 | 0 | 0 | 0 | 1 | 2 | 5 | 2 |
| 0 | 0 | 0 | 0 | 0 | | 8 | 6 |
| 0 | 0 | 0 | 0 | 0 | 1 | 5 | 1 |
| 0 | 0 | 0 | 0 | 0 | | 8 | 6 |
| 0 | 0 | 0 | 0 | 0 | | 8 | 6 |
| 0 | 0 | 0 | 0 | 0 | | 8 | 6 |
| 0 | 0 | 0 | 0 | 1 | 1 | 5 | 2 |
| 0 | 0 | 0 | 1 | 1 | 2 | 5 | 2 |
| 0 | 0 | 0 | 0 | 0 | | 8 | 6 |
| 0 | 0 | 0 | 0 | 0 | | 8 | 6 |
| 0 | 0 | 0 | 0 | 0 | | 8 | 6 |
| 0 | 0 | 0 | 0 | 0 | | 8 | 6 |
| 0 | 0 | 0 | 0 | 0 | | 8 | 6 |
| 0 | 1 | 0 | 0 | 0 | 1 | 6 | 1 |
| 0 | 0 | 0 | 0 | 0 | | 8 | 6 |
| 0 | 0 | 0 | 0 | 0 | | 8 | 6 |
| 0 | 0 | 0 | 0 | 1 | 1 | 4 | 2 |
| 0 | 0 | 0 | 0 | 1 | 1 | 4 | 1 |
| 0 | 0 | 0 | 0 | 0 | | 8 | 6 |
| 0 | 0 | 0 | 0 | 0 | | 8 | 6 |

| | | | | | | | |
|---|---|---|---|---|---|---|---|
| 0 | 0 | 0 | 0 | 0 | 2 | 4 | 3 |
| 0 | 0 | 1 | 0 | 0 | 1 | 4 | 3 |
| 0 | 0 | 0 | 0 | 1 | 1 | 4 | 2 |
| 0 | 0 | 0 | 0 | 0 | | 8 | 6 |
| 0 | 0 | 0 | 0 | 0 | | 8 | 6 |
| 0 | 0 | 0 | 0 | 0 | | 8 | 6 |
| 0 | 0 | 0 | 0 | 0 | | 8 | 6 |
| 0 | 0 | 0 | 0 | 0 | | 8 | 6 |
| 0 | 0 | 0 | 0 | 0 | | 8 | 6 |
| 0 | 0 | 0 | 0 | 0 | | 8 | 6 |
| 0 | 0 | 0 | 0 | 0 | | 8 | 6 |
| 0 | 0 | 0 | 0 | 1 | 1 | 1 | 5 |
| 0 | 0 | 0 | 1 | 1 | 2 | 5 | 1 |
| 0 | 0 | 0 | 0 | 0 | | 8 | 6 |
| 0 | 0 | 0 | 0 | 0 | | 8 | 6 |
| 0 | 0 | 0 | 0 | 0 | | 8 | 6 |
| 0 | 0 | 0 | 0 | 1 | 1 | 4 | 1 |
| 0 | 0 | 0 | 0 | 1 | 1 | 4 | 1 |
| 0 | 0 | 0 | 0 | 0 | | 8 | 6 |
| 0 | 0 | 0 | 0 | 0 | | 8 | 6 |
| 0 | 0 | 0 | 0 | 0 | | 8 | 6 |
| 0 | 0 | 0 | 0 | 0 | | 8 | 6 |
| 0 | 0 | 0 | 0 | 0 | | 8 | 6 |
| 0 | 1 | 0 | 0 | 1 | 2 | 5 | 1 |
| 0 | 0 | 0 | 0 | 0 | | 8 | 6 |
| 0 | 0 | 0 | 0 | 0 | | 8 | 6 |
| 0 | 0 | 0 | 0 | 0 | | 8 | 6 |
| 0 | 1 | 0 | 0 | 1 | 2 | 5 | 5 |
| 0 | 0 | 0 | 0 | 0 | | 8 | 6 |
| 0 | 0 | 0 | 0 | 0 | | 8 | 6 |
| 0 | 0 | 0 | 0 | 0 | | 8 | 6 |
| 0 | 0 | 0 | 0 | 0 | | 8 | 6 |
| 0 | 0 | 0 | 0 | 0 | | 8 | 6 |
| 0 | 0 | 0 | 0 | 0 | | 8 | 6 |
| 0 | 0 | 0 | 0 | 0 | | 8 | 6 |
| 0 | 0 | 0 | 0 | 1 | 1 | 5 | 6 |
| 0 | 0 | 0 | 0 | 0 | | 8 | 6 |
| 0 | 0 | 0 | 0 | 0 | | 8 | 6 |
| 0 | 0 | 0 | 0 | 0 | | 8 | 6 |
| 0 | 0 | 0 | 0 | 0 | | 8 | 6 |
| 0 | 0 | 0 | 0 | 0 | | 8 | 6 |
| 0 | 0 | 0 | 0 | 0 | | 8 | 6 |
| 0 | 0 | 0 | 0 | 0 | | 8 | 6 |
| 0 | 0 | 0 | 1 | 0 | 1 | 4 | 3 |
| 0 | 0 | 0 | 0 | 0 | | 8 | 6 |
| 0 | 0 | 0 | 1 | 1 | 2 | 4 | 2 |
| 0 | 0 | 0 | 0 | 0 | | 8 | 6 |
| 0 | 0 | 0 | 1 | 0 | 1 | 4 | 1 |

| | | | | | | | |
|---|---|---|---|---|---|---|---|
| 0 | 0 | 0 | 0 | 0 | | 8 | 6 |
| 0 | 0 | 0 | 0 | 1 | 2 | 5 | 3 |
| 0 | 0 | 0 | 0 | 0 | | 8 | 6 |
| 0 | 0 | 0 | 1 | 1 | 2 | 4 | 1 |
| 0 | 0 | 0 | 0 | 0 | | 8 | 6 |
| 0 | 0 | 0 | 0 | 0 | | 8 | 6 |
| 0 | 0 | 0 | 0 | 0 | | 8 | 6 |
| 0 | 0 | 0 | 0 | 0 | | 8 | 6 |
| 0 | 0 | 0 | 0 | 0 | | 5 | 3 |
| 0 | 0 | 0 | 0 | 0 | | 8 | 6 |
| 0 | 0 | 0 | 0 | 0 | | 8 | 6 |
| 0 | 0 | 0 | 0 | 0 | | 8 | 6 |
| 0 | 0 | 0 | 0 | 0 | | 8 | 6 |
| 0 | 0 | 0 | 0 | 0 | | 8 | 6 |
| 0 | 0 | 0 | 0 | 0 | | 8 | 6 |
| 0 | 0 | 0 | 0 | 0 | | 8 | 6 |
| 0 | 0 | 0 | 0 | 0 | | 6 | 6 |
| 0 | 0 | 0 | 0 | 0 | | 8 | 6 |
| 0 | 0 | 0 | 0 | 0 | | 8 | 1 |
| 0 | 0 | 0 | 0 | 0 | | 6 | 6 |
| 0 | 0 | 0 | 0 | 0 | | 6 | 3 |
| 0 | 0 | 0 | 0 | 0 | | 8 | 6 |
| 0 | 0 | 0 | 0 | 0 | 1 | 5 | 1 |
| 0 | 0 | 0 | 0 | 0 | | 7 | 6 |
| 0 | 0 | 0 | 0 | 0 | | 8 | 6 |
| 0 | 0 | 0 | 0 | 0 | | 8 | 1 |
| 0 | 0 | 0 | 0 | 0 | | 8 | 6 |
| 0 | 0 | 0 | 0 | 0 | 1 | 6 | 2 |
| 0 | 0 | 0 | 0 | 0 | 1 | 3 | 1 |
| 0 | 0 | 0 | 0 | 0 | | 8 | 6 |
| 0 | 0 | 0 | 0 | 0 | | 1 | 3 |
| 0 | 0 | 0 | 0 | 0 | | 7 | 1 |
| 0 | 0 | 0 | 0 | 0 | | 8 | 6 |
| 0 | 0 | 0 | 0 | 0 | | 5 | 6 |
| 0 | 0 | 0 | 0 | 0 | | 8 | 6 |
| 0 | 0 | 0 | 0 | 0 | | 8 | 6 |
| 0 | 0 | 0 | 0 | 0 | | 8 | 6 |
| 1 | 1 | 0 | 0 | 0 | 2 | 7 | 1 |
| 0 | 0 | 0 | 0 | 0 | | 6 | 2 |
| 0 | 0 | 0 | 0 | 0 | | 6 | 6 |
| 0 | 0 | 0 | 0 | 0 | | 8 | 6 |
| 0 | 0 | 0 | 0 | 0 | | 8 | 6 |
| 0 | 0 | 0 | 0 | 0 | | 7 | 1 |
| 0 | 0 | 0 | 0 | 0 | | 6 | 3 |
| 0 | 0 | 0 | 0 | 0 | | 8 | 6 |
| 0 | 0 | 0 | 0 | 0 | | 8 | 6 |
| 0 | 0 | 0 | 0 | 0 | 1 | 5 | 3 |
| 0 | 0 | 0 | 0 | 0 | | 6 | 1 |

| | | | | | | | |
|---|---|---|---|---|---|---|---|
| 0 | 1 | 0 | 0 | 0 | 1 | 5 | 1 |
| 0 | 0 | 0 | 0 | 0 | | 8 | 6 |
| 0 | 0 | 0 | 0 | 0 | 1 | 4 | 4 |
| 0 | 0 | 0 | 0 | 0 | | 8 | 6 |
| 0 | 0 | 0 | 0 | 0 | | 8 | 6 |
| 0 | 0 | 0 | 0 | 0 | | 8 | 6 |
| 0 | 0 | 0 | 0 | 0 | | 8 | 6 |
| 0 | 0 | 0 | 0 | 0 | | 8 | 6 |
| 0 | 0 | 0 | 0 | 0 | | 5 | 6 |
| 0 | 0 | 0 | 0 | 0 | | 6 | 6 |
| 0 | 0 | 0 | 0 | 0 | | 6 | 6 |
| 0 | 0 | 0 | 0 | 0 | | 4 | 2 |
| 0 | 0 | 0 | 0 | 0 | | 8 | 6 |
| 0 | 0 | 0 | 0 | 0 | | 8 | 6 |
| 0 | 0 | 0 | 0 | 0 | | 6 | 2 |
| 0 | 0 | 0 | 0 | 0 | | 8 | 6 |
| 0 | 0 | 0 | 0 | 0 | | 8 | 6 |
| 1 | 0 | 0 | 0 | 0 | 2 | 4 | 3 |
| 0 | 0 | 0 | 0 | 0 | | 8 | 6 |
| 0 | 0 | 0 | 0 | 0 | | 1 | 3 |
| 0 | 0 | 0 | 0 | 0 | | 8 | 6 |
| 0 | 0 | 0 | 0 | 0 | | 8 | 6 |
| 0 | 0 | 0 | 0 | 0 | | 7 | 6 |
| 0 | 0 | 0 | 0 | 0 | | 8 | 6 |
| 0 | 0 | 0 | 0 | 0 | | 4 | 1 |
| 0 | 0 | 0 | 0 | 0 | | 8 | 6 |
| 0 | 0 | 0 | 0 | 0 | | 8 | 6 |
| 0 | 0 | 0 | 0 | 0 | | 6 | 1 |
| 0 | 0 | 0 | 0 | 0 | | 4 | 3 |
| 0 | 0 | 0 | 0 | 0 | 2 | 6 | 1 |
| 0 | 0 | 0 | 0 | 0 | | 6 | 1 |
| 0 | 0 | 0 | 0 | 0 | | 5 | 6 |
| 0 | 0 | 0 | 0 | 0 | | 5 | 1 |
| 0 | 0 | 0 | 0 | 0 | | 8 | 6 |
| 0 | 0 | 0 | 0 | 0 | | 7 | 2 |
| 0 | 0 | 0 | 0 | 0 | | 8 | 6 |
| 0 | 0 | 0 | 0 | 0 | | 5 | 1 |
| 0 | 0 | 0 | 0 | 0 | | 8 | 6 |
| 0 | 0 | 0 | 0 | 0 | | 5 | 2 |
| 0 | 0 | 0 | 0 | 0 | | 8 | 6 |
| 0 | 0 | 0 | 0 | 0 | | 4 | 6 |
| 0 | 0 | 0 | 0 | 0 | | 8 | 6 |
| 0 | 0 | 0 | 0 | 0 | | 6 | 2 |
| 0 | 0 | 0 | 0 | 0 | | 8 | 6 |
| 0 | 0 | 0 | 0 | 0 | | 8 | 6 |
| 0 | 0 | 0 | 0 | 0 | 1 | 6 | 1 |
| 0 | 0 | 0 | 0 | 0 | | 6 | 4 |
| 0 | 0 | 0 | 0 | 0 | | 6 | 1 |

| | | | | | | | |
|---|---|---|---|---|---|---|---|
| 0 | 0 | 0 | 0 | 0 | | 8 | 6 |
| 0 | 0 | 0 | 0 | 0 | | 8 | 6 |
| 0 | 0 | 0 | 0 | 0 | | 8 | 6 |
| 0 | 0 | 0 | 0 | 0 | | 8 | 6 |
| 0 | 0 | 0 | 0 | 0 | | 8 | 6 |
| 0 | 0 | 0 | 0 | 0 | | 8 | 6 |
| 0 | 0 | 0 | 0 | 0 | | 7 | 3 |
| 0 | 0 | 0 | 0 | 0 | | 8 | 6 |
| 0 | 0 | 0 | 0 | 0 | | 7 | 2 |
| 0 | 0 | 0 | 0 | 0 | | 8 | 6 |
| 0 | 0 | 0 | 0 | 0 | | 6 | 2 |
| 0 | 0 | 0 | 0 | 0 | | 4 | 6 |
| 1 | 0 | 0 | 0 | 0 | 2 | 5 | 3 |
| 0 | 0 | 0 | 0 | 0 | | 5 | 6 |
| 1 | 0 | 0 | 0 | 0 | 1 | 6 | 1 |
| 0 | 0 | 0 | 0 | 0 | | 4 | 1 |
| 0 | 0 | 0 | 0 | 0 | | 8 | 6 |
| 0 | 0 | 0 | 0 | 0 | | 6 | 1 |
| 0 | 0 | 0 | 0 | 0 | | 8 | 6 |
| 0 | 0 | 0 | 0 | 0 | | 6 | 2 |
| 0 | 0 | 0 | 0 | 0 | | 5 | 6 |
| 0 | 0 | 0 | 0 | 0 | | 6 | 1 |
| 0 | 0 | 0 | 0 | 0 | | 8 | 6 |
| 0 | 0 | 0 | 0 | 0 | | 8 | 6 |
| 0 | 0 | 0 | 0 | 0 | | 8 | 6 |
| 0 | 0 | 0 | 0 | 0 | | 5 | 1 |
| 0 | 0 | 0 | 0 | 0 | | 8 | 6 |
| 0 | 0 | 0 | 0 | 0 | | 6 | 6 |
| 0 | 0 | 0 | 0 | 0 | | 7 | 3 |
| 0 | 0 | 0 | 0 | 0 | | 7 | 6 |
| 0 | 0 | 0 | 0 | 0 | | 8 | 6 |
| 0 | 0 | 0 | 0 | 0 | | 8 | 6 |
| 0 | 0 | 0 | 0 | 0 | | 8 | 6 |
| 0 | 0 | 0 | 0 | 0 | | 8 | 6 |
| 0 | 0 | 0 | 0 | 0 | | 8 | 6 |
| 0 | 0 | 0 | 0 | 0 | | 8 | 6 |
| 0 | 0 | 0 | 0 | 0 | | 8 | 6 |
| 0 | 0 | 0 | 0 | 0 | | 6 | 1 |
| 0 | 0 | 0 | 0 | 0 | | 7 | 6 |
| 0 | 0 | 0 | 0 | 0 | | 7 | 4 |
| 0 | 1 | 0 | 0 | 0 | 1 | 5 | 1 |
| 0 | 0 | 0 | 0 | 0 | | 7 | 6 |
| 0 | 0 | 0 | 0 | 0 | | 8 | 6 |
| 0 | 0 | 0 | 0 | 0 | | 8 | 6 |
| 0 | 0 | 0 | 0 | 0 | | 6 | 1 |
| 0 | 0 | 0 | 0 | 0 | | 8 | 6 |
| 0 | 0 | 0 | 0 | 0 | | 8 | 6 |
| 0 | 0 | 0 | 0 | 0 | | 8 | 6 |

| | | | | | | | |
|---|---|---|---|---|---|---|---|
| 0 | 0 | 0 | 0 | 0 | | 8 | 6 |
| 0 | 0 | 0 | 0 | 0 | | 6 | 1 |
| 0 | 0 | 0 | 1 | 0 | 1 | 6 | 1 |
| 0 | 0 | 0 | 0 | 0 | | 6 | 6 |
| 0 | 0 | 0 | 0 | 0 | | 6 | 6 |
| 0 | 0 | 0 | 0 | 0 | 1 | 5 | 1 |
| 0 | 0 | 0 | 0 | 0 | | 7 | 1 |
| 0 | 0 | 0 | 0 | 0 | | 5 | 6 |
| 0 | 0 | 0 | 0 | 0 | | 8 | 6 |
| 0 | 0 | 0 | 1 | 0 | 1 | 5 | 3 |
| 0 | 0 | 0 | 0 | 0 | | 8 | 6 |
| 0 | 0 | 0 | 0 | 0 | 1 | 6 | 2 |
| 0 | 0 | 0 | 0 | 0 | 1 | 2 | 3 |
| 0 | 0 | 0 | 0 | 0 | | 7 | 6 |
| 0 | 0 | 0 | 0 | 0 | | 8 | 6 |
| 0 | 0 | 0 | 0 | 0 | | 8 | 6 |
| 0 | 0 | 0 | 1 | 0 | 1 | 5 | 3 |
| 0 | 0 | 0 | 0 | 0 | | 7 | 1 |
| 0 | 0 | 0 | 0 | 0 | | 5 | 1 |
| 0 | 0 | 0 | 0 | 0 | | 6 | 1 |
| 0 | 0 | 0 | 0 | 0 | | 8 | 6 |
| 0 | 0 | 0 | 0 | 0 | | 6 | 6 |
| 0 | 0 | 0 | 0 | 0 | | 8 | 6 |
| 0 | 0 | 0 | 0 | 0 | | 8 | 1 |
| 0 | 0 | 0 | 0 | 0 | | 8 | 6 |
| 0 | 0 | 0 | 0 | 0 | | 6 | 6 |
| 0 | 0 | 0 | 0 | 0 | | 5 | 1 |
| 0 | 0 | 0 | 0 | 0 | | 8 | 6 |
| 0 | 0 | 0 | 0 | 0 | | 8 | 6 |
| 0 | 0 | 0 | 0 | 0 | | 8 | 1 |
| 0 | 0 | 0 | 0 | 0 | | 8 | 6 |
| 0 | 0 | 0 | 0 | 0 | | 6 | 1 |
| 0 | 0 | 0 | 0 | 0 | | 8 | 6 |
| 0 | 0 | 0 | 0 | 0 | | 8 | 6 |
| 0 | 0 | 0 | 0 | 0 | | 7 | 1 |
| 0 | 0 | 0 | 0 | 0 | | 8 | 6 |
| 0 | 0 | 0 | 0 | 0 | | 8 | 6 |
| 0 | 0 | 0 | 0 | 0 | | 8 | 6 |
| 0 | 0 | 0 | 0 | 0 | | 6 | 6 |
| 0 | 0 | 0 | 0 | 0 | | 8 | 6 |
| 0 | 0 | 0 | 0 | 0 | | 3 | 1 |
| 0 | 0 | 0 | 0 | 0 | | 6 | 6 |
| 0 | 0 | 0 | 0 | 0 | | 8 | 6 |
| 0 | 0 | 0 | 0 | 0 | | 8 | 6 |
| 0 | 0 | 0 | 0 | 0 | | 8 | 6 |
| 0 | 0 | 0 | 0 | 0 | | 8 | 6 |
| 0 | 0 | 0 | 0 | 0 | | 8 | 6 |
| 0 | 0 | 0 | 0 | 0 | | 4 | 6 |

| | | | | | | | |
|---|---|---|---|---|---|---|---|
| 0 | 0 | 0 | 0 | 0 | | 8 | 6 |
| 0 | 0 | 0 | 0 | 0 | | 8 | 6 |
| 0 | 0 | 0 | 0 | 0 | | 8 | 6 |
| 0 | 0 | 0 | 0 | 1 | 1 | 4 | 5 |
| 0 | 0 | 0 | 1 | 0 | 2 | 4 | 5 |
| 0 | 0 | 0 | 1 | 0 | 2 | 5 | 5 |
| 0 | 0 | 0 | 0 | 0 | | 8 | 6 |
| 0 | 0 | 0 | 0 | 0 | | 8 | 6 |
| 0 | 0 | 0 | 0 | 0 | | 8 | 6 |
| 0 | 0 | 0 | 0 | 0 | | 8 | 6 |
| 0 | 0 | 0 | 0 | 0 | | 8 | 6 |
| 0 | 0 | 0 | 0 | 0 | | 8 | 6 |
| 0 | 0 | 0 | 0 | 0 | | 6 | 6 |
| 0 | 0 | 0 | 0 | 0 | | 6 | 6 |
| 0 | 0 | 0 | 0 | 0 | | 8 | 6 |
| 0 | 0 | 0 | 0 | 0 | | 6 | 3 |
| 0 | 0 | 0 | 0 | 0 | | 8 | 6 |
| 0 | 0 | 0 | 0 | 0 | | 6 | 2 |
| 0 | 0 | 0 | 0 | 0 | | 8 | 6 |
| 0 | 0 | 0 | 0 | 0 | | 8 | 6 |
| 0 | 0 | 0 | 0 | 0 | | 8 | 6 |
| 0 | 0 | 0 | 0 | 1 | 2 | 4 | 5 |
| 0 | 0 | 0 | 1 | 0 | 2 | 4 | 5 |
| 0 | 0 | 0 | 1 | 0 | 2 | 5 | 5 |
| 0 | 0 | 0 | 1 | 0 | 2 | 5 | 3 |
| 0 | 0 | 0 | 0 | 1 | 2 | 5 | 5 |
| 0 | 0 | 0 | 0 | 0 | 1 | 6 | 1 |
| 1 | 0 | 0 | 0 | 1 | 2 | 4 | 5 |
| 0 | 0 | 0 | 1 | 0 | 2 | 4 | 3 |
| 0 | 0 | 0 | 0 | 1 | 2 | 5 | 5 |
| 0 | 0 | 0 | 0 | 1 | 2 | 5 | 3 |
| 0 | 0 | 0 | 0 | 0 | | 6 | 6 |
| 0 | 0 | 0 | 0 | 0 | | 6 | 6 |
| 0 | 0 | 0 | 0 | 0 | | 8 | 6 |
| 0 | 0 | 0 | 0 | 0 | | 8 | 6 |
| 0 | 1 | 1 | 0 | 0 | 2 | 6 | 1 |
| 0 | 0 | 0 | 0 | 0 | | 8 | 6 |
| 1 | 0 | 0 | 1 | 0 | 2 | 5 | 3 |
| 0 | 0 | 0 | 0 | 0 | | 8 | 6 |
| 0 | 0 | 0 | 0 | 0 | | 8 | 6 |
| 0 | 0 | 0 | 0 | 0 | | 8 | 6 |
| 0 | 0 | 0 | 0 | 0 | | 6 | 1 |
| 0 | 0 | 0 | 0 | 0 | | 6 | 1 |
| 0 | 0 | 0 | 0 | 0 | | 8 | 6 |
| 0 | 0 | 0 | 0 | 0 | | 8 | 6 |
| 0 | 0 | 0 | 0 | 0 | | 5 | 6 |
| 0 | 0 | 0 | 0 | 0 | | 8 | 6 |
| 0 | 0 | 0 | 0 | 0 | | 8 | 6 |

| | | | | | | | |
|---|---|---|---|---|---|---|---|
| 0 | 0 | 0 | 0 | 0 | | 5 | 6 |
| 0 | 0 | 0 | 0 | 0 | | 8 | 6 |
| 0 | 0 | 0 | 1 | 0 | 2 | 5 | 3 |
| 0 | 0 | 0 | 0 | 0 | | 8 | 6 |
| 0 | 1 | 0 | 0 | 0 | 1 | 5 | 5 |
| 0 | 0 | 0 | 0 | 0 | | 6 | 6 |
| 0 | 0 | 0 | 0 | 0 | | 8 | 6 |
| 0 | 0 | 0 | 0 | 0 | | 5 | 6 |
| 0 | 0 | 0 | 1 | 0 | 2 | 4 | 3 |
| 0 | 0 | 0 | 0 | 0 | | 8 | 6 |
| 0 | 0 | 0 | 0 | 0 | | 8 | 6 |
| 0 | 0 | 0 | 0 | 0 | | 8 | 6 |
| 0 | 0 | 1 | 0 | 0 | 1 | 5 | 1 |
| 0 | 0 | 0 | 0 | 0 | | 8 | 6 |
| 0 | 0 | 0 | 0 | 0 | | 8 | 6 |
| 0 | 0 | 0 | 0 | 0 | | 8 | 6 |
| 0 | 0 | 0 | 0 | 0 | | 8 | 6 |
| 0 | 0 | 0 | 0 | 1 | 1 | 6 | 3 |
| 0 | 0 | 0 | 0 | 1 | 2 | 4 | 1 |
| 0 | 0 | 1 | 0 | 0 | 1 | 5 | 5 |
| 0 | 0 | 0 | 0 | 0 | 1 | 5 | 5 |
| 0 | 0 | 0 | 0 | 0 | | 8 | 6 |
| 0 | 0 | 0 | 0 | 0 | | 8 | 6 |
| 0 | 0 | 0 | 0 | 0 | | 8 | 6 |
| 0 | 0 | 0 | 0 | 0 | | 8 | 6 |
| 0 | 0 | 0 | 0 | 0 | | 8 | 6 |
| 0 | 0 | 0 | 0 | 0 | | 8 | 6 |
| 0 | 0 | 0 | 0 | 0 | | 8 | 6 |
| 0 | 0 | 0 | 1 | 0 | 2 | 6 | 5 |
| 0 | 0 | 0 | 0 | 0 | | 8 | 6 |
| 0 | 0 | 0 | 0 | 0 | | 8 | 6 |
| 0 | 0 | 0 | 0 | 0 | | 8 | 6 |
| 0 | 0 | 0 | 1 | 0 | 2 | 5 | 1 |
| 0 | 0 | 0 | 1 | 0 | 2 | 4 | 1 |
| 0 | 0 | 0 | 0 | 0 | | 8 | 6 |
| 0 | 0 | 0 | 0 | 0 | | 8 | 6 |
| 0 | 0 | 0 | 0 | 0 | | 8 | 6 |
| 0 | 0 | 0 | 0 | 0 | | 8 | 6 |
| 0 | 0 | 0 | 0 | 0 | | 8 | 6 |
| 0 | 0 | 0 | 0 | 0 | | 8 | 6 |
| 0 | 0 | 0 | 0 | 0 | | 8 | 6 |
| 0 | 0 | 0 | 0 | 0 | | 8 | 6 |
| 0 | 0 | 0 | 0 | 0 | | 8 | 6 |
| 0 | 0 | 0 | 0 | 0 | | 8 | 6 |
| 0 | 0 | 0 | 0 | 0 | | 8 | 6 |
| 0 | 0 | 0 | 0 | 0 | | 8 | 6 |
| 0 | 0 | 0 | 0 | 0 | | 8 | 6 |
| 0 | 0 | 0 | 0 | 0 | | 8 | 6 |

| | | | | | | | |
|---|---|---|---|---|---|---|---|
| 0 | 0 | 0 | 0 | 0 | | 8 | 6 |
| 0 | 0 | 0 | 0 | 0 | | 8 | 6 |
| 0 | 0 | 0 | 0 | 0 | | 8 | 6 |
| 0 | 0 | 0 | 0 | 0 | | 8 | 6 |
| 0 | 0 | 0 | 0 | 0 | | 8 | 6 |
| 0 | 0 | 0 | 0 | 0 | | 8 | 6 |
| 0 | 0 | 0 | 0 | 0 | | 8 | 6 |
| 0 | 0 | 0 | 0 | 0 | | 7 | 4 |
| 0 | 0 | 0 | 0 | 0 | | 6 | 6 |
| 0 | 0 | 0 | 0 | 0 | | 8 | 6 |
| 0 | 0 | 0 | 0 | 0 | | 6 | 6 |
| 0 | 0 | 0 | 0 | 0 | | 8 | 6 |
| 0 | 0 | 0 | 1 | 0 | 2 | 4 | 2 |
| 0 | 0 | 0 | 0 | 0 | | 8 | 6 |
| 0 | 0 | 0 | 0 | 0 | | 5 | 4 |
| 0 | 0 | 0 | 0 | 0 | | 8 | 6 |
| 0 | 0 | 0 | 1 | 1 | 2 | 4 | 5 |
| 0 | 0 | 0 | 0 | 0 | | 8 | 6 |
| 1 | 0 | 0 | 1 | 0 | 2 | 4 | 1 |
| 0 | 0 | 0 | 0 | 0 | | 8 | 6 |
| 0 | 0 | 0 | 0 | 0 | | 8 | 6 |
| 0 | 0 | 0 | 0 | 0 | | 8 | 6 |
| 0 | 0 | 0 | 1 | 0 | 1 | 5 | 5 |
| 0 | 0 | 0 | 0 | 0 | | 8 | 6 |
| 0 | 0 | 0 | 1 | 0 | 1 | 4 | 1 |
| 1 | 0 | 0 | 0 | 0 | 1 | 5 | 2 |
| 0 | 0 | 0 | 0 | 0 | | 8 | 6 |
| 0 | 0 | 0 | 1 | 0 | 2 | 5 | 3 |
| 0 | 0 | 0 | 1 | 0 | 2 | 4 | 5 |
| 1 | 0 | 0 | 0 | 0 | 2 | 1 | 2 |
| 0 | 0 | 0 | 0 | 0 | | 8 | 6 |
| 0 | 0 | 0 | 0 | 0 | | 8 | 6 |
| 0 | 0 | 0 | 0 | 0 | | 8 | 6 |
| 0 | 0 | 0 | 1 | 0 | 2 | 4 | 1 |
| 0 | 0 | 0 | 0 | 1 | 1 | 5 | 1 |
| 0 | 0 | 0 | 0 | 0 | | 8 | 6 |
| 0 | 0 | 0 | 0 | 1 | 1 | 5 | 2 |
| 0 | 0 | 0 | 0 | 0 | | 8 | 6 |
| 0 | 0 | 0 | 0 | 0 | 1 | 5 | 1 |
| 0 | 0 | 0 | 0 | 0 | | 8 | 6 |
| 0 | 0 | 0 | 0 | 1 | 1 | 1 | 6 |
| 0 | 0 | 0 | 0 | 1 | 1 | 5 | 2 |
| 1 | 0 | 0 | 1 | 0 | 2 | 6 | 1 |
| 0 | 0 | 0 | 1 | 0 | 1 | 5 | 5 |

| | | | |
|---|---|---|---|
| o no | 1 single | 1 UK | 1 medicien |
| 1 yes | 2 multiple | 2 EU | 2 pharmacy |
| | | 3 USA | 3 dentistry |

| | |
|---|---|
| 4 UAE | 4 nursing |
| 5 Arab | 5 engineering |
| 6 asian (india, pakistan, srilanka, Philip) | 6 other/not specified |
| 7 other | |
| 8 NA | |

| year | why | positiveEffects | when | howLong | frequency | buyFrom | cost |
|---|---|---|---|---|---|---|---|
| 3 | 6 | 3 | 5 | 6 | 6 | 7 | 6 |
| 3 | 6 | 3 | 5 | 6 | 6 | 7 | 6 |
| 4 | 1 | 1 | 1 | 1 | 4 | 1 | 3 |
| 4 | 6 | 3 | 5 | 6 | 6 | 7 | 6 |
| 7 | 6 | 3 | 5 | 6 | 6 | 7 | 6 |
| 7 | 6 | 3 | 5 | 6 | 6 | 7 | 6 |
| 3 | 6 | 3 | 5 | 6 | 6 | 7 | 6 |
| 7 | 6 | 3 | 5 | 6 | 6 | 7 | 6 |
| 7 | 6 | 3 | 5 | 6 | 6 | 7 | 6 |
| 4 | 6 | 3 | 5 | 6 | 6 | 7 | 6 |
| 7 | 6 | 3 | 5 | 6 | 6 | 7 | 6 |
| 7 | 6 | 3 | 5 | 6 | 6 | 7 | 6 |
| 1 | 6 | 3 | 5 | 6 | 6 | 7 | 6 |
| 7 | 6 | 3 | 5 | 6 | 6 | 7 | 6 |
| 7 | 6 | 3 | 5 | 6 | 6 | 7 | 6 |
| 7 | 6 | 3 | 5 | 6 | 6 | 7 | 6 |
| 7 | 6 | 3 | 5 | 6 | 6 | 7 | 6 |
| 7 | 6 | 3 | 5 | 6 | 6 | 7 | 6 |
| 4 | 6 | 3 | 5 | 6 | 6 | 7 | 6 |
| 4 | 2 | 2 | 1 | 1 | 1 | 6 | 2 |
| 1 | 6 | 3 | 5 | 6 | 6 | 7 | 6 |
| 1 | 6 | 3 | 5 | 6 | 6 | 7 | 6 |
| 3 | 6 | 3 | 5 | 6 | 6 | 7 | 6 |
| 2 | 6 | 3 | 5 | 6 | 6 | 7 | 6 |
| 3 | 5 | 1 | 5 | 6 | 6 | 7 | 6 |
| 7 | 6 | 3 | 5 | 6 | 6 | 7 | 6 |
| 4 | 1 | 1 | 1 | 1 | 5 | 6 | 2 |
| 1 | 6 | 2 | 5 | 6 | 6 | 7 | 6 |
| 7 | 6 | 3 | 5 | 6 | 6 | 7 | 6 |
| 7 | 6 | 3 | 5 | 6 | 6 | 7 | 6 |
| 2 | 1 | 1 | 1 | 4 | 2 | 5 | 3 |
| 7 | 6 | 3 | 5 | 6 | 6 | 7 | 6 |
| 7 | 6 | 3 | 5 | 6 | 6 | 7 | 6 |
| 7 | 6 | 3 | 5 | 6 | 6 | 7 | 6 |
| 1 | 2 | 2 | 3 | 1 | 2 | 6 | 2 |
| 4 | 6 | 3 | 5 | 6 | 6 | 7 | 6 |
| 4 | 6 | 3 | 5 | 6 | 6 | 7 | 6 |
| 7 | 6 | 3 | 5 | 6 | 6 | 7 | 6 |
| 7 | 6 | 3 | 5 | 6 | 6 | 7 | 6 |
| 7 | 6 | 3 | 5 | 6 | 6 | 7 | 6 |
| 3 | 6 | 3 | 5 | 6 | 6 | 7 | 6 |
| 7 | 6 | 3 | 5 | 6 | 6 | 7 | 6 |
| 1 | 6 | 3 | 5 | 6 | 6 | 7 | 6 |
| 7 | 6 | 3 | 5 | 6 | 6 | 7 | 6 |
| 3 | 1 | 2 | 1 | 2 | 1 | 2 | 3 |
| 7 | 6 | 3 | 5 | 6 | 6 | 7 | 6 |

| | | | | | | | |
|---|---|---|---|---|---|---|---|
| 7 | 6 | 3 | 5 | 6 | 6 | 7 | 6 |
| 1 | 4 | 1 | 3 | 2 | 1 | 5 | 3 |
| 7 | 6 | 3 | 5 | 6 | 6 | 7 | 6 |
| 2 | 1 | 2 | 1 | 1 | 1 | 5 | 3 |
| 7 | 6 | 3 | 5 | 6 | 6 | 7 | 6 |
| 7 | 6 | 3 | 5 | 6 | 6 | 7 | 6 |
| 2 | 6 | 3 | 5 | 6 | 6 | 7 | 6 |
| 7 | 6 | 3 | 5 | 6 | 6 | 7 | 6 |
| 1 | 1 | 2 | 1 | 2 | 1 | 6 | 3 |
| 7 | 6 | 3 | 5 | 6 | 6 | 7 | 6 |
| 2 | 6 | 2 | 5 | 6 | 6 | 7 | 6 |
| 4 | 5 | 2 | 3 | 1 | 4 | 6 | 3 |
| 7 | 6 | 3 | 5 | 6 | 6 | 7 | 6 |
| 1 | 2 | 2 | 1 | 1 | 5 | 1 | 3 |
| 4 | 2 | 1 | 1 | 1 | 5 | 2 | 2 |
| 4 | 4 | 2 | 2 | 1 | 4 | 4 | 4 |
| 7 | 6 | 3 | 5 | 6 | 6 | 7 | 6 |
| 7 | 6 | 3 | 5 | 6 | 6 | 7 | 6 |
| 3 | 1 | 1 | 1 | 2 | 1 | 5 | 2 |
| 4 | 1 | 2 | 1 | 1 | 5 | 5 | 2 |
| 7 | 6 | 3 | 5 | 6 | 6 | 7 | 6 |
| 1 | 6 | 3 | 5 | 6 | 6 | 7 | 6 |
| 7 | 6 | 3 | 5 | 6 | 6 | 7 | 6 |
| 7 | 6 | 3 | 5 | 6 | 6 | 7 | 6 |
| 7 | 6 | 3 | 5 | 6 | 6 | 7 | 6 |
| 2 | 6 | 3 | 5 | 6 | 6 | 7 | 6 |
| 7 | 6 | 3 | 5 | 6 | 6 | 7 | 6 |
| 7 | 6 | 3 | 5 | 6 | 6 | 7 | 6 |
| 7 | 6 | 3 | 5 | 6 | 6 | 7 | 6 |
| 7 | 6 | 3 | 5 | 6 | 6 | 7 | 6 |
| 3 | 6 | 3 | 5 | 6 | 6 | 7 | 6 |
| 4 | 1 | 1 | 1 | 1 | 5 | 5 | 2 |
| 4 | 1 | 2 | 1 | 1 | 5 | 2 | 2 |
| 2 | 3 | 1 | 1 | 2 | 4 | 5 | 3 |
| 4 | 1 | 1 | 1 | 1 | 1 | 5 | 3 |
| 7 | 6 | 3 | 5 | 6 | 6 | 7 | 6 |
| 3 | 1 | 2 | 1 | 1 | 1 | 2 | 2 |
| 4 | 3 | 1 | 1 | 1 | 1 | 5 | 2 |
| 5 | 3 | 1 | 1 | 2 | 1 | 5 | 2 |
| 4 | 1 | 1 | 1 | 2 | 1 | 5 | 2 |
| 4 | 2 | 1 | 1 | 2 | 1 | 5 | 2 |
| 3 | 2 | 1 | 1 | 2 | 1 | 5 | 2 |
| 3 | 3 | 2 | 1 | 1 | 1 | 5 | 2 |
| 3 | 3 | 1 | 1 | 2 | 1 | 5 | 2 |
| 3 | 1 | 1 | 1 | 2 | 1 | 5 | 2 |
| 3 | 3 | 1 | 1 | 2 | 1 | 5 | 2 |
| 7 | 6 | 3 | 5 | 6 | 6 | 7 | 6 |
| 4 | 3 | 1 | 1 | 2 | 1 | 5 | 2 |

| | | | | | | | |
|---|---|---|---|---|---|---|---|
| 5 | 2 | 2 | 1 | 1 | 4 | 5 | 3 |
| 3 | 1 | 1 | 1 | 2 | 4 | 5 | 3 |
| 3 | 1 | 1 | 1 | 2 | 1 | 5 | 3 |
| 2 | 3 | 1 | 1 | 2 | 1 | 5 | 3 |
| 7 | 6 | 3 | 5 | 6 | 6 | 7 | 6 |
| 4 | 3 | 1 | 1 | 2 | 1 | 5 | 2 |
| 3 | 1 | 1 | 1 | 3 | 1 | 5 | 2 |
| 4 | 2 | 1 | 1 | 1 | 1 | 2 | 3 |
| 7 | 6 | 3 | 5 | 6 | 6 | 7 | 6 |
| 7 | 6 | 3 | 5 | 6 | 6 | 7 | 6 |
| 3 | 3 | 1 | 1 | 2 | 1 | 5 | 2 |
| 5 | 2 | 1 | 1 | 2 | 4 | 2 | 2 |
| 7 | 6 | 3 | 5 | 6 | 6 | 7 | 6 |
| 5 | 4 | 1 | 1 | 2 | 1 | 2 | 2 |
| 4 | 2 | 1 | 1 | 2 | 1 | 5 | 2 |
| 3 | 2 | 1 | 1 | 2 | 1 | 7 | 6 |
| 4 | 3 | 1 | 1 | 2 | 1 | 5 | 3 |
| 5 | 4 | 1 | 1 | 2 | 1 | 5 | 3 |
| 7 | 6 | 3 | 5 | 6 | 6 | 7 | 6 |
| 7 | 6 | 3 | 5 | 6 | 6 | 7 | 6 |
| 4 | 4 | 1 | 1 | 2 | 1 | 5 | 3 |
| 7 | 6 | 3 | 5 | 6 | 6 | 7 | 6 |
| 7 | 6 | 3 | 5 | 6 | 6 | 7 | 6 |
| 5 | 4 | 1 | 1 | 2 | 1 | 2 | 3 |
| 7 | 6 | 3 | 5 | 6 | 6 | 7 | 6 |
| 7 | 6 | 3 | 5 | 6 | 6 | 7 | 6 |
| 7 | 6 | 3 | 5 | 6 | 6 | 7 | 6 |
| 7 | 6 | 3 | 5 | 6 | 6 | 7 | 6 |
| 4 | 4 | 2 | 1 | 2 | 2 | 5 | 3 |
| 7 | 6 | 3 | 5 | 6 | 6 | 7 | 6 |
| 5 | 4 | 2 | 1 | 1 | 5 | 2 | 2 |
| 7 | 6 | 3 | 5 | 6 | 6 | 7 | 6 |
| 7 | 6 | 3 | 5 | 6 | 6 | 7 | 6 |
| 7 | 6 | 3 | 5 | 6 | 6 | 7 | 6 |
| 3 | 3 | 1 | 1 | 1 | 4 | 5 | 3 |
| 4 | 3 | 1 | 1 | 4 | 4 | 5 | 2 |
| 7 | 6 | 3 | 5 | 6 | 6 | 7 | 6 |
| 7 | 6 | 3 | 5 | 6 | 6 | 7 | 6 |
| 7 | 6 | 3 | 5 | 6 | 6 | 7 | 6 |
| 7 | 6 | 3 | 5 | 6 | 6 | 7 | 6 |
| 7 | 6 | 3 | 5 | 6 | 6 | 7 | 6 |
| 3 | 4 | 1 | 1 | 2 | 4 | 2 | 2 |
| 7 | 6 | 3 | 5 | 6 | 6 | 7 | 6 |
| 7 | 6 | 3 | 5 | 6 | 6 | 7 | 6 |
| 3 | 1 | 1 | 1 | 2 | 4 | 5 | 3 |
| 4 | 1 | 1 | 1 | 3 | 4 | 5 | 2 |
| 7 | 6 | 3 | 5 | 6 | 6 | 7 | 6 |
| 7 | 6 | 3 | 5 | 6 | 6 | 7 | 6 |

| | | | | | | | |
|---|---|---|---|---|---|---|---|
| 4 | 1 | 1 | 1 | 3 | 4 | 5 | 3 |
| 3 | 4 | 1 | 3 | 2 | 2 | 2 | 2 |
| 2 | 3 | 1 | 1 | 2 | 3 | 5 | 3 |
| 7 | 6 | 3 | 5 | 6 | 6 | 7 | 6 |
| 7 | 6 | 3 | 5 | 6 | 6 | 7 | 6 |
| 7 | 6 | 3 | 5 | 6 | 6 | 7 | 6 |
| 7 | 6 | 3 | 5 | 6 | 6 | 7 | 6 |
| 7 | 6 | 3 | 5 | 6 | 6 | 7 | 6 |
| 7 | 6 | 3 | 5 | 6 | 6 | 7 | 6 |
| 7 | 6 | 3 | 5 | 6 | 6 | 7 | 6 |
| 7 | 6 | 3 | 5 | 6 | 6 | 7 | 6 |
| 3 | 3 | 1 | 1 | 1 | 4 | 5 | 2 |
| 5 | 3 | 1 | 1 | 2 | 5 | 5 | 2 |
| 7 | 6 | 3 | 5 | 6 | 6 | 7 | 6 |
| 7 | 6 | 3 | 5 | 6 | 6 | 7 | 6 |
| 7 | 6 | 3 | 5 | 6 | 6 | 7 | 6 |
| 5 | 3 | 1 | 1 | 2 | 5 | 5 | 2 |
| 4 | 1 | 1 | 1 | 2 | 4 | 5 | 2 |
| 7 | 6 | 3 | 5 | 6 | 6 | 7 | 6 |
| 7 | 6 | 3 | 5 | 6 | 6 | 7 | 6 |
| 7 | 6 | 3 | 5 | 6 | 6 | 7 | 6 |
| 7 | 6 | 3 | 5 | 6 | 6 | 7 | 6 |
| 7 | 6 | 3 | 5 | 6 | 6 | 7 | 6 |
| 5 | 1 | 1 | 5 | 2 | 4 | 5 | 2 |
| 7 | 6 | 3 | 5 | 6 | 6 | 7 | 6 |
| 7 | 6 | 3 | 5 | 6 | 6 | 7 | 6 |
| 7 | 6 | 3 | 5 | 6 | 6 | 7 | 6 |
| 3 | 4 | 1 | 1 | 1 | 4 | 2 | 2 |
| 7 | 6 | 3 | 5 | 6 | 6 | 7 | 6 |
| 7 | 6 | 3 | 5 | 6 | 6 | 7 | 6 |
| 7 | 6 | 3 | 5 | 6 | 6 | 7 | 6 |
| 7 | 6 | 3 | 5 | 6 | 6 | 7 | 6 |
| 7 | 6 | 3 | 5 | 6 | 6 | 7 | 6 |
| 7 | 1 | 3 | 5 | 6 | 6 | 7 | 6 |
| 7 | 6 | 3 | 5 | 6 | 6 | 7 | 6 |
| 6 | 1 | 1 | 1 | 1 | 3 | 1 | 2 |
| 7 | 6 | 3 | 5 | 6 | 6 | 7 | 6 |
| 7 | 6 | 3 | 5 | 6 | 6 | 7 | 6 |
| 7 | 6 | 3 | 5 | 6 | 6 | 7 | 6 |
| 7 | 6 | 3 | 5 | 6 | 6 | 7 | 6 |
| 7 | 6 | 3 | 5 | 6 | 6 | 7 | 6 |
| 7 | 6 | 3 | 5 | 6 | 6 | 7 | 6 |
| 7 | 6 | 3 | 5 | 6 | 6 | 7 | 6 |
| 4 | 3 | 1 | 1 | 2 | 3 | 5 | 3 |
| 7 | 6 | 3 | 5 | 6 | 6 | 7 | 6 |
| 3 | 1 | 1 | 1 | 2 | 3 | 5 | 2 |
| 7 | 6 | 3 | 5 | 6 | 6 | 7 | 6 |
| 4 | 3 | 1 | 1 | 1 | 4 | 5 | 3 |

| | | | | | | | |
|---|---|---|---|---|---|---|---|
| 7 | 6 | 3 | 5 | 6 | 6 | 7 | 6 |
| 4 | 1 | 1 | 1 | 2 | 4 | 5 | 2 |
| 7 | 6 | 3 | 5 | 6 | 6 | 7 | 6 |
| 4 | 1 | 1 | 1 | 2 | 4 | 5 | 2 |
| 7 | 6 | 3 | 5 | 6 | 6 | 7 | 6 |
| 7 | 6 | 3 | 5 | 6 | 6 | 7 | 6 |
| 7 | 6 | 3 | 5 | 6 | 6 | 7 | 6 |
| 7 | 6 | 3 | 5 | 6 | 6 | 7 | 6 |
| 2 | 6 | 3 | 5 | 6 | 6 | 7 | 6 |
| 7 | 6 | 3 | 5 | 6 | 6 | 7 | 6 |
| 7 | 6 | 3 | 5 | 6 | 6 | 7 | 6 |
| 7 | 6 | 3 | 5 | 6 | 6 | 7 | 6 |
| 7 | 6 | 3 | 5 | 6 | 6 | 7 | 6 |
| 7 | 6 | 3 | 5 | 6 | 6 | 7 | 6 |
| 7 | 6 | 3 | 5 | 6 | 6 | 7 | 6 |
| 7 | 6 | 3 | 5 | 6 | 6 | 7 | 6 |
| 3 | 6 | 3 | 5 | 6 | 6 | 7 | 6 |
| 7 | 6 | 3 | 5 | 6 | 6 | 7 | 6 |
| 4 | 6 | 3 | 5 | 6 | 6 | 7 | 6 |
| 3 | 6 | 3 | 5 | 6 | 6 | 7 | 2 |
| 3 | 6 | 3 | 5 | 6 | 6 | 7 | 6 |
| 7 | 6 | 3 | 5 | 6 | 6 | 7 | 6 |
| 4 | 3 | 2 | 1 | 1 | 4 | 2 | 3 |
| 2 | 5 | 1 | 5 | 6 | 6 | 7 | 6 |
| 7 | 6 | 3 | 5 | 6 | 6 | 7 | 6 |
| 3 | 6 | 3 | 5 | 6 | 6 | 7 | 6 |
| 7 | 6 | 3 | 5 | 6 | 6 | 7 | 6 |
| 2 | 3 | 2 | 2 | 1 | 5 | 4 | 2 |
| 2 | 1 | 2 | 4 | 5 | 1 | 5 | 3 |
| 7 | 6 | 3 | 5 | 6 | 6 | 7 | 6 |
| 4 | 6 | 3 | 5 | 6 | 6 | 7 | 6 |
| 1 | 6 | 2 | 5 | 6 | 6 | 7 | 6 |
| 7 | 6 | 3 | 5 | 6 | 6 | 7 | 6 |
| 3 | 5 | 1 | 5 | 6 | 6 | 6 | 6 |
| 7 | 6 | 3 | 5 | 6 | 6 | 7 | 6 |
| 7 | 6 | 3 | 5 | 6 | 6 | 7 | 6 |
| 7 | 6 | 3 | 5 | 6 | 6 | 7 | 6 |
| 4 | 5 | 1 | 1 | 1 | 1 | 1 | 1 |
| 2 | 6 | 3 | 5 | 6 | 6 | 7 | 6 |
| 1 | 6 | 3 | 5 | 6 | 6 | 7 | 6 |
| 7 | 6 | 3 | 5 | 6 | 6 | 7 | 6 |
| 7 | 6 | 3 | 5 | 6 | 6 | 7 | 6 |
| 4 | 6 | 3 | 5 | 6 | 6 | 7 | 6 |
| 3 | 6 | 3 | 5 | 6 | 6 | 7 | 6 |
| 7 | 6 | 3 | 5 | 6 | 6 | 7 | 6 |
| 7 | 6 | 3 | 5 | 6 | 6 | 7 | 6 |
| 2 | 5 | 2 | 1 | 1 | 1 | 5 | 3 |
| 2 | 6 | 3 | 5 | 6 | 6 | 7 | 6 |

| | | | | | | | |
|---|---|---|---|---|---|---|---|
| 6 | 1 | 1 | 2 | 3 | 2 | 1 | 1 |
| 7 | 6 | 3 | 5 | 6 | 6 | 7 | 6 |
| 1 | 5 | 2 | 4 | 1 | 3 | 6 | 2 |
| 7 | 6 | 3 | 5 | 6 | 6 | 7 | 6 |
| 7 | 6 | 3 | 5 | 6 | 6 | 7 | 6 |
| 7 | 6 | 3 | 5 | 6 | 6 | 7 | 6 |
| 7 | 6 | 3 | 5 | 6 | 6 | 7 | 6 |
| 7 | 6 | 3 | 5 | 6 | 6 | 7 | 6 |
| 1 | 6 | 3 | 5 | 6 | 6 | 7 | 6 |
| 1 | 6 | 3 | 5 | 6 | 6 | 7 | 6 |
| 3 | 6 | 3 | 5 | 6 | 6 | 7 | 6 |
| 1 | 6 | 2 | 5 | 6 | 6 | 7 | 6 |
| 7 | 6 | 3 | 5 | 6 | 6 | 7 | 6 |
| 7 | 6 | 3 | 5 | 6 | 6 | 7 | 6 |
| 2 | 6 | 3 | 5 | 6 | 6 | 7 | 6 |
| 7 | 6 | 3 | 5 | 6 | 6 | 7 | 6 |
| 7 | 6 | 3 | 5 | 6 | 6 | 7 | 6 |
| 1 | 4 | 2 | 1 | 2 | 5 | 5 | 3 |
| 7 | 6 | 3 | 5 | 6 | 6 | 7 | 6 |
| 2 | 6 | 3 | 5 | 6 | 6 | 7 | 6 |
| 7 | 6 | 3 | 5 | 6 | 6 | 7 | 6 |
| 7 | 6 | 3 | 5 | 6 | 6 | 7 | 6 |
| 3 | 6 | 3 | 5 | 6 | 6 | 7 | 6 |
| 7 | 6 | 3 | 5 | 6 | 6 | 7 | 6 |
| 1 | 6 | 3 | 5 | 6 | 6 | 7 | 6 |
| 7 | 6 | 3 | 5 | 6 | 6 | 7 | 6 |
| 7 | 6 | 3 | 5 | 6 | 6 | 7 | 6 |
| 5 | 6 | 3 | 5 | 6 | 6 | 7 | 6 |
| 5 | 6 | 3 | 5 | 6 | 6 | 7 | 6 |
| 1 | 5 | 1 | 4 | 2 | 1 | 1 | 4 |
| 1 | 6 | 3 | 5 | 6 | 6 | 7 | 6 |
| 2 | 6 | 3 | 5 | 6 | 6 | 7 | 6 |
| 4 | 1 | 1 | 4 | 1 | 5 | 6 | 6 |
| 7 | 6 | 3 | 5 | 6 | 6 | 7 | 6 |
| 1 | 6 | 2 | 5 | 6 | 6 | 7 | 6 |
| 7 | 6 | 3 | 5 | 6 | 6 | 7 | 6 |
| 3 | 6 | 3 | 5 | 6 | 6 | 7 | 6 |
| 7 | 6 | 3 | 5 | 6 | 6 | 7 | 6 |
| 5 | 1 | 2 | 3 | 1 | 5 | 7 | 6 |
| 7 | 6 | 3 | 5 | 6 | 6 | 7 | 6 |
| 4 | 6 | 3 | 5 | 6 | 6 | 7 | 6 |
| 7 | 6 | 3 | 5 | 6 | 6 | 7 | 6 |
| 2 | 6 | 3 | 5 | 6 | 6 | 7 | 6 |
| 7 | 6 | 3 | 5 | 6 | 6 | 7 | 6 |
| 7 | 6 | 3 | 5 | 6 | 6 | 7 | 6 |
| 4 | 5 | 1 | 1 | 1 | 3 | 1 | 3 |
| 2 | 4 | 2 | 5 | 6 | 6 | 7 | 6 |
| 5 | 6 | 3 | 5 | 6 | 6 | 7 | 6 |

| | | | | | | | |
|---|---|---|---|---|---|---|---|
| 7 | 6 | 3 | 5 | 6 | 6 | 7 | 6 |
| 7 | 6 | 3 | 5 | 6 | 6 | 7 | 6 |
| 7 | 6 | 3 | 5 | 6 | 6 | 7 | 6 |
| 7 | 6 | 3 | 5 | 6 | 6 | 7 | 6 |
| 7 | 6 | 3 | 5 | 6 | 6 | 7 | 6 |
| 7 | 6 | 3 | 5 | 6 | 6 | 7 | 6 |
| 1 | 6 | 3 | 5 | 6 | 6 | 7 | 6 |
| 7 | 6 | 3 | 5 | 6 | 6 | 7 | 6 |
| 4 | 6 | 3 | 5 | 6 | 6 | 7 | 6 |
| 7 | 6 | 3 | 5 | 6 | 6 | 7 | 6 |
| 5 | 6 | 3 | 5 | 6 | 6 | 7 | 6 |
| 7 | 5 | 3 | 5 | 6 | 6 | 7 | 6 |
| 3 | 3 | 2 | 1 | 1 | 5 | 5 | 2 |
| 1 | 3 | 2 | 1 | 1 | 1 | 6 | 3 |
| 3 | 4 | 1 | 1 | 2 | 3 | 1 | 3 |
| 3 | 6 | 3 | 5 | 6 | 6 | 7 | 6 |
| 7 | 6 | 3 | 5 | 6 | 6 | 7 | 6 |
| 1 | 6 | 3 | 5 | 6 | 6 | 7 | 6 |
| 7 | 6 | 3 | 5 | 6 | 6 | 7 | 6 |
| 3 | 6 | 3 | 5 | 6 | 6 | 7 | 6 |
| 4 | 6 | 3 | 5 | 6 | 6 | 7 | 6 |
| 2 | 6 | 3 | 5 | 6 | 6 | 7 | 6 |
| 7 | 6 | 3 | 5 | 6 | 6 | 7 | 6 |
| 7 | 6 | 3 | 5 | 6 | 6 | 7 | 6 |
| 7 | 6 | 3 | 5 | 6 | 6 | 7 | 6 |
| 2 | 6 | 3 | 5 | 6 | 6 | 7 | 6 |
| 7 | 6 | 3 | 5 | 6 | 6 | 7 | 6 |
| 7 | 6 | 3 | 5 | 6 | 6 | 7 | 6 |
| 1 | 6 | 3 | 5 | 6 | 6 | 7 | 6 |
| 3 | 6 | 3 | 5 | 6 | 6 | 7 | 6 |
| 7 | 6 | 3 | 5 | 6 | 6 | 7 | 6 |
| 7 | 6 | 3 | 5 | 6 | 6 | 7 | 6 |
| 7 | 6 | 3 | 5 | 6 | 6 | 7 | 6 |
| 7 | 6 | 3 | 5 | 6 | 6 | 7 | 6 |
| 7 | 6 | 3 | 5 | 6 | 6 | 7 | 6 |
| 7 | 6 | 3 | 5 | 6 | 6 | 7 | 6 |
| 7 | 6 | 3 | 5 | 6 | 6 | 7 | 6 |
| 3 | 6 | 3 | 5 | 6 | 6 | 7 | 6 |
| 7 | 6 | 3 | 5 | 6 | 6 | 7 | 6 |
| 1 | 6 | 3 | 5 | 6 | 6 | 7 | 6 |
| 2 | 1 | 2 | 4 | 2 | 1 | 1 | 3 |
| 7 | 5 | 1 | 5 | 6 | 6 | 7 | 6 |
| 7 | 6 | 3 | 5 | 6 | 6 | 7 | 6 |
| 7 | 6 | 3 | 5 | 6 | 6 | 7 | 6 |
| 1 | 6 | 3 | 5 | 6 | 6 | 7 | 6 |
| 7 | 6 | 3 | 5 | 6 | 6 | 7 | 6 |
| 7 | 6 | 3 | 5 | 6 | 6 | 7 | 6 |
| 7 | 6 | 3 | 5 | 6 | 6 | 7 | 6 |

| | | | | | | | |
|---|---|---|---|---|---|---|---|
| 7 | 6 | 3 | 5 | 6 | 6 | 7 | 6 |
| 3 | 6 | 3 | 5 | 6 | 6 | 7 | 6 |
| 2 | 1 | 1 | 1 | 1 | 4 | 5 | 2 |
| 2 | 6 | 3 | 5 | 6 | 6 | 7 | 6 |
| 1 | 6 | 2 | 5 | 6 | 6 | 7 | 6 |
| 3 | 5 | 2 | 3 | 3 | 5 | 1 | 3 |
| 3 | 6 | 3 | 5 | 6 | 6 | 7 | 6 |
| 2 | 6 | 3 | 5 | 6 | 6 | 7 | 6 |
| 7 | 6 | 3 | 5 | 6 | 6 | 7 | 6 |
| 4 | 3 | 1 | 1 | 1 | 4 | 5 | 2 |
| 7 | 6 | 3 | 5 | 6 | 6 | 7 | 6 |
| 2 | 5 | 2 | 1 | 1 | 5 | 1 | 3 |
| 1 | 4 | 1 | 1 | 1 | 4 | 5 | 2 |
| 7 | 6 | 3 | 5 | 6 | 6 | 7 | 6 |
| 7 | 6 | 3 | 5 | 6 | 6 | 7 | 6 |
| 7 | 6 | 3 | 5 | 6 | 6 | 7 | 6 |
| 1 | 3 | 1 | 1 | 6 | 4 | 5 | 3 |
| 2 | 6 | 3 | 5 | 6 | 6 | 7 | 6 |
| 1 | 6 | 3 | 5 | 6 | 6 | 7 | 6 |
| 3 | 6 | 3 | 5 | 6 | 6 | 7 | 6 |
| 7 | 6 | 3 | 5 | 6 | 6 | 7 | 6 |
| 1 | 5 | 2 | 5 | 6 | 6 | 7 | 6 |
| 7 | 6 | 3 | 5 | 6 | 6 | 7 | 6 |
| 5 | 6 | 3 | 5 | 6 | 6 | 7 | 6 |
| 7 | 6 | 3 | 5 | 6 | 6 | 7 | 6 |
| 1 | 6 | 3 | 5 | 6 | 6 | 7 | 6 |
| 3 | 6 | 3 | 5 | 6 | 6 | 7 | 6 |
| 7 | 6 | 3 | 5 | 6 | 6 | 7 | 6 |
| 7 | 6 | 3 | 5 | 6 | 6 | 7 | 6 |
| 1 | 6 | 3 | 5 | 6 | 6 | 7 | 6 |
| 7 | 6 | 3 | 5 | 6 | 6 | 7 | 6 |
| 4 | 6 | 2 | 5 | 6 | 6 | 7 | 6 |
| 7 | 6 | 3 | 5 | 6 | 6 | 7 | 6 |
| 7 | 6 | 3 | 5 | 6 | 6 | 7 | 6 |
| 5 | 6 | 3 | 5 | 6 | 6 | 7 | 6 |
| 7 | 6 | 3 | 5 | 6 | 6 | 7 | 6 |
| 7 | 6 | 3 | 5 | 6 | 6 | 7 | 6 |
| 7 | 6 | 3 | 5 | 6 | 6 | 7 | 6 |
| 1 | 6 | 3 | 5 | 6 | 6 | 7 | 6 |
| 7 | 6 | 3 | 5 | 6 | 6 | 7 | 6 |
| 2 | 6 | 3 | 5 | 6 | 6 | 7 | 6 |
| 4 | 6 | 3 | 5 | 6 | 6 | 7 | 6 |
| 7 | 6 | 3 | 5 | 6 | 6 | 7 | 6 |
| 7 | 6 | 3 | 5 | 6 | 6 | 7 | 6 |
| 7 | 6 | 3 | 5 | 6 | 6 | 7 | 6 |
| 7 | 6 | 3 | 5 | 6 | 6 | 7 | 6 |
| 7 | 6 | 3 | 5 | 6 | 6 | 7 | 6 |
| 7 | 5 | 3 | 5 | 6 | 6 | 7 | 6 |

| | | | | | | | |
|---|---|---|---|---|---|---|---|
| 7 | 6 | 3 | 5 | 6 | 6 | 7 | 6 |
| 7 | 6 | 3 | 5 | 6 | 6 | 7 | 6 |
| 7 | 6 | 3 | 5 | 6 | 6 | 7 | 6 |
| 3 | 1 | 1 | 1 | 2 | 4 | 5 | 2 |
| 2 | 4 | 1 | 1 | 3 | 5 | 5 | 2 |
| 4 | 4 | 1 | 1 | 2 | 5 | 5 | 2 |
| 7 | 6 | 3 | 5 | 6 | 6 | 7 | 6 |
| 7 | 6 | 3 | 5 | 6 | 6 | 7 | 6 |
| 7 | 6 | 3 | 5 | 6 | 6 | 7 | 6 |
| 7 | 6 | 3 | 5 | 6 | 6 | 7 | 6 |
| 7 | 6 | 3 | 5 | 6 | 6 | 7 | 6 |
| 7 | 6 | 3 | 5 | 6 | 6 | 7 | 6 |
| 2 | 4 | 2 | 5 | 6 | 6 | 7 | 6 |
| 4 | 6 | 3 | 5 | 6 | 6 | 7 | 6 |
| 7 | 6 | 3 | 5 | 6 | 6 | 7 | 6 |
| 3 | 6 | 2 | 5 | 6 | 6 | 7 | 6 |
| 7 | 6 | 3 | 5 | 6 | 6 | 7 | 6 |
| 1 | 6 | 3 | 5 | 6 | 6 | 7 | 6 |
| 7 | 6 | 3 | 5 | 6 | 6 | 7 | 6 |
| 7 | 6 | 3 | 5 | 6 | 6 | 7 | 6 |
| 7 | 6 | 3 | 5 | 6 | 6 | 7 | 6 |
| 3 | 4 | 1 | 1 | 2 | 5 | 5 | 3 |
| 4 | 4 | 1 | 1 | 2 | 5 | 5 | 2 |
| 3 | 3 | 1 | 1 | 2 | 4 | 5 | 2 |
| 3 | 4 | 1 | 1 | 2 | 4 | 5 | 2 |
| 4 | 4 | 1 | 1 | 2 | 5 | 5 | 2 |
| 4 | 4 | 1 | 1 | 2 | 4 | 2 | 2 |
| 3 | 4 | 1 | 1 | 2 | 5 | 5 | 2 |
| 4 | 4 | 1 | 1 | 2 | 4 | 5 | 2 |
| 4 | 4 | 1 | 1 | 2 | 4 | 5 | 2 |
| 3 | 4 | 1 | 1 | 2 | 4 | 5 | 2 |
| 2 | 6 | 2 | 5 | 6 | 6 | 7 | 6 |
| 2 | 6 | 2 | 5 | 6 | 6 | 7 | 6 |
| 7 | 6 | 3 | 5 | 6 | 6 | 7 | 6 |
| 7 | 6 | 3 | 5 | 6 | 6 | 7 | 6 |
| 4 | 4 | 1 | 1 | 2 | 4 | 5 | 2 |
| 7 | 6 | 3 | 5 | 6 | 6 | 7 | 6 |
| 4 | 4 | 1 | 1 | 3 | 5 | 5 | 2 |
| 7 | 6 | 3 | 5 | 6 | 6 | 7 | 6 |
| 7 | 6 | 3 | 5 | 6 | 6 | 7 | 6 |
| 7 | 6 | 3 | 5 | 6 | 6 | 7 | 6 |
| 3 | 6 | 3 | 5 | 6 | 6 | 7 | 6 |
| 3 | 6 | 3 | 5 | 6 | 6 | 7 | 6 |
| 7 | 6 | 3 | 5 | 6 | 6 | 7 | 6 |
| 7 | 6 | 3 | 5 | 6 | 6 | 7 | 6 |
| 3 | 6 | 3 | 5 | 6 | 6 | 7 | 6 |
| 7 | 6 | 3 | 5 | 6 | 6 | 7 | 6 |
| 7 | 6 | 3 | 5 | 6 | 6 | 7 | 6 |

| | | | | | | | |
|---|---|---|---|---|---|---|---|
| 1 | 4 | 3 | 3 | 6 | 6 | 7 | 6 |
| 7 | 6 | 3 | 5 | 6 | 6 | 7 | 6 |
| 3 | 4 | 1 | 1 | 2 | 4 | 5 | 3 |
| 7 | 6 | 3 | 5 | 6 | 6 | 7 | 6 |
| 4 | 4 | 1 | 3 | 1 | 5 | 2 | 3 |
| 2 | 6 | 3 | 5 | 6 | 6 | 7 | 6 |
| 7 | 6 | 3 | 5 | 6 | 6 | 7 | 6 |
| 4 | 6 | 3 | 5 | 6 | 6 | 7 | 6 |
| 3 | 4 | 1 | 1 | 2 | 5 | 5 | 3 |
| 7 | 6 | 3 | 5 | 6 | 6 | 7 | 6 |
| 7 | 6 | 3 | 5 | 6 | 6 | 7 | 6 |
| 7 | 6 | 3 | 5 | 6 | 6 | 7 | 6 |
| 3 | 4 | 1 | 1 | 1 | 5 | 2 | 3 |
| 7 | 6 | 3 | 5 | 6 | 6 | 7 | 6 |
| 7 | 6 | 3 | 5 | 6 | 6 | 7 | 6 |
| 7 | 6 | 3 | 5 | 6 | 6 | 7 | 6 |
| 7 | 6 | 3 | 5 | 6 | 6 | 7 | 6 |
| 4 | 4 | 1 | 1 | 1 | 5 | 2 | 3 |
| 4 | 4 | 1 | 1 | 2 | 5 | 5 | 3 |
| 3 | 4 | 1 | 1 | 2 | 4 | 2 | 3 |
| 3 | 4 | 1 | 1 | 2 | 4 | 2 | 3 |
| 7 | 6 | 3 | 5 | 6 | 6 | 7 | 6 |
| 7 | 6 | 3 | 5 | 6 | 6 | 7 | 6 |
| 7 | 6 | 3 | 5 | 6 | 6 | 7 | 6 |
| 7 | 6 | 3 | 5 | 6 | 6 | 7 | 6 |
| 7 | 6 | 3 | 5 | 6 | 6 | 7 | 6 |
| 7 | 6 | 3 | 5 | 6 | 6 | 7 | 6 |
| 7 | 6 | 3 | 5 | 6 | 6 | 7 | 6 |
| 3 | 3 | 1 | 3 | 2 | 2 | 5 | 3 |
| 7 | 6 | 3 | 5 | 6 | 6 | 7 | 6 |
| 7 | 6 | 3 | 5 | 6 | 6 | 7 | 6 |
| 7 | 6 | 3 | 5 | 6 | 6 | 7 | 6 |
| 5 | 3 | 1 | 3 | 3 | 2 | 5 | 3 |
| 4 | 1 | 1 | 3 | 3 | 2 | 5 | 3 |
| 7 | 6 | 3 | 5 | 6 | 6 | 7 | 6 |
| 7 | 6 | 3 | 5 | 6 | 6 | 7 | 6 |
| 7 | 6 | 3 | 5 | 6 | 6 | 7 | 6 |
| 7 | 6 | 3 | 5 | 6 | 6 | 7 | 6 |
| 7 | 6 | 3 | 5 | 6 | 6 | 7 | 6 |
| 7 | 6 | 3 | 5 | 6 | 6 | 7 | 6 |
| 7 | 6 | 3 | 5 | 6 | 6 | 7 | 6 |
| 7 | 6 | 3 | 5 | 6 | 6 | 7 | 6 |
| 7 | 6 | 3 | 5 | 6 | 6 | 7 | 6 |
| 7 | 6 | 3 | 5 | 6 | 6 | 7 | 6 |
| 7 | 6 | 3 | 5 | 6 | 6 | 7 | 6 |
| 7 | 6 | 3 | 5 | 6 | 6 | 7 | 6 |
| 7 | 6 | 3 | 5 | 6 | 6 | 7 | 6 |
| 7 | 6 | 3 | 5 | 6 | 6 | 7 | 6 |

| | | | | | | | |
|---|---|---|---|---|---|---|---|
| 7 | 6 | 3 | 5 | 6 | 6 | 7 | 6 |
| 7 | 6 | 3 | 5 | 6 | 6 | 7 | 6 |
| 7 | 6 | 3 | 5 | 6 | 6 | 7 | 6 |
| 7 | 6 | 3 | 5 | 6 | 6 | 7 | 6 |
| 7 | 6 | 3 | 5 | 6 | 6 | 7 | 6 |
| 7 | 6 | 3 | 5 | 6 | 6 | 7 | 6 |
| 7 | 6 | 3 | 5 | 6 | 6 | 7 | 6 |
| 2 | 6 | 3 | 5 | 6 | 6 | 7 | 6 |
| 2 | 6 | 3 | 5 | 6 | 6 | 7 | 6 |
| 7 | 6 | 3 | 5 | 6 | 6 | 7 | 6 |
| 4 | 6 | 3 | 5 | 6 | 6 | 7 | 6 |
| 7 | 6 | 3 | 5 | 6 | 6 | 7 | 6 |
| 4 | 1 | 1 | 1 | 2 | 4 | 5 | 3 |
| 7 | 6 | 3 | 5 | 6 | 6 | 7 | 6 |
| 2 | 1 | 1 | 1 | 1 | 4 | 1 | 2 |
| 7 | 6 | 3 | 5 | 6 | 6 | 7 | 6 |
| 4 | 1 | 1 | 1 | 3 | 4 | 5 | 2 |
| 7 | 6 | 3 | 5 | 6 | 6 | 7 | 6 |
| 3 | 3 | 1 | 1 | 2 | 4 | 2 | 3 |
| 7 | 6 | 3 | 5 | 6 | 6 | 7 | 6 |
| 7 | 6 | 3 | 5 | 6 | 6 | 7 | 6 |
| 7 | 6 | 3 | 5 | 6 | 6 | 7 | 6 |
| 4 | 3 | 1 | 3 | 2 | 3 | 6 | 3 |
| 7 | 6 | 3 | 5 | 6 | 6 | 7 | 6 |
| 2 | 3 | 1 | 1 | 2 | 4 | 5 | 3 |
| 4 | 4 | 1 | 3 | 3 | 2 | 6 | 2 |
| 7 | 6 | 3 | 5 | 6 | 6 | 7 | 6 |
| 4 | 4 | 1 | 1 | 2 | 4 | 6 | 2 |
| 3 | 4 | 1 | 1 | 3 | 3 | 5 | 3 |
| 2 | 6 | 2 | 4 | 5 | 1 | 6 | 3 |
| 7 | 6 | 3 | 5 | 6 | 6 | 7 | 6 |
| 7 | 6 | 3 | 5 | 6 | 6 | 7 | 6 |
| 7 | 6 | 3 | 5 | 6 | 6 | 7 | 6 |
| 5 | 4 | 1 | 3 | 3 | 2 | 6 | 3 |
| 3 | 4 | 1 | 1 | 2 | 4 | 2 | 3 |
| 7 | 6 | 3 | 5 | 6 | 6 | 7 | 6 |
| 4 | 3 | 1 | 1 | 2 | 4 | 2 | 2 |
| 7 | 6 | 3 | 5 | 6 | 6 | 7 | 6 |
| 4 | 4 | 1 | 1 | 3 | 4 | 5 | 3 |
| 7 | 6 | 3 | 5 | 6 | 6 | 7 | 6 |
| 3 | 1 | 1 | 2 | 1 | 1 | 2 | 3 |
| 4 | 3 | 1 | 3 | 3 | 3 | 5 | 2 |
| 4 | 4 | 1 | 3 | 2 | 3 | 5 | 3 |
| 4 | 3 | 1 | 3 | 2 | 4 | 5 | 3 |

| | | | | | | | |
|---|---|---|---|---|---|---|---|
| 1 first | 1 concentration | 1 yes | 1 exam | 1 less month | 1 daily | 1 me | 1 v exp |
| 2 second | 2 memory | 2 no | 2 work deadlines | 2 1-6 | 2 weeky | 2 friend | 2 exp |
| 7 not specified | 3 alertness | 3 NA | 3 studying | 3 6-12 | 3 month | 3 stranger | 3 fair |

| | | | | | |
|---|---|---|---|---|---|
| 4 academic | 4 daily | 4 1-2 year | 4 once semster | 4 somebody else | 4 cheap |
| 5 others | 5 NA | 5 more 2 y | 5 less than semster | 5 online | 5 v cheap |
| 6 NA | | 6 NA | 6 NA | 6 other | 6 NA |
| | | | | 7 NA | |

| KnowSB | heardFrom | recommend |
|---|---|---|
| 3 | 7 | 3 |
| 3 | 7 | 3 |
| 1 | 6 | 2 |
| 1 | 6 | 2 |
| 3 | 7 | 3 |
| 3 | 7 | 3 |
| 3 | 7 | 3 |
| 3 | 7 | 3 |
| 3 | 7 | 3 |
| 3 | 6 | 3 |
| 3 | 7 | 3 |
| 3 | 7 | 3 |
| 3 | 7 | 3 |
| 3 | 7 | 3 |
| 3 | 7 | 3 |
| 3 | 7 | 3 |
| 3 | 7 | 3 |
| 3 | 7 | 3 |
| 1 | 7 | 2 |
| 2 | 2 | 1 |
| 3 | 7 | 3 |
| 1 | 7 | 3 |
| 3 | 7 | 3 |
| 2 | 6 | 1 |
| 1 | 7 | 3 |
| 3 | 7 | 3 |
| 2 | 2 | 2 |
| 1 | 1 | 2 |
| 3 | 7 | 3 |
| 3 | 7 | 3 |
| 1 | 2 | 1 |
| 3 | 7 | 3 |
| 3 | 7 | 3 |
| 3 | 7 | 3 |
| 1 | 5 | 2 |
| 3 | 7 | 2 |
| 3 | 7 | 3 |
| 3 | 7 | 3 |
| 3 | 7 | 3 |
| 3 | 7 | 3 |
| 3 | 7 | 3 |
| 3 | 7 | 3 |
| 3 | 7 | 3 |
| 3 | 7 | 3 |
| 2 | 1 | 2 |
| 3 | 7 | 3 |

| | | |
|---|---|---|
| 3 | 7 | 3 |
| 2 | 3 | 2 |
| 3 | 7 | 3 |
| 2 | 4 | 1 |
| 3 | 7 | 3 |
| 3 | 7 | 3 |
| 3 | 7 | 2 |
| 3 | 7 | 3 |
| 2 | 4 | 2 |
| 3 | 7 | 3 |
| 3 | 7 | 3 |
| 2 | 5 | 2 |
| 3 | 7 | 3 |
| 1 | 4 | 2 |
| 2 | 4 | 2 |
| 1 | 2 | 1 |
| 3 | 7 | 3 |
| 3 | 7 | 3 |
| 2 | 4 | 1 |
| 2 | 4 | 2 |
| 3 | 7 | 3 |
| 3 | 7 | 3 |
| 3 | 7 | 3 |
| 3 | 7 | 3 |
| 3 | 7 | 3 |
| 3 | 7 | 3 |
| 3 | 7 | 3 |
| 3 | 7 | 3 |
| 3 | 7 | 3 |
| 3 | 7 | 3 |
| 3 | 7 | 3 |
| 1 | 4 | 1 |
| 2 | 4 | 2 |
| 2 | 4 | 1 |
| 2 | 4 | 1 |
| 3 | 7 | 3 |
| 2 | 4 | 2 |
| 1 | 1 | 2 |
| 1 | 1 | 1 |
| 2 | 4 | 2 |
| 1 | 1 | 2 |
| 2 | 4 | 2 |
| 1 | 4 | 3 |
| 1 | 4 | 2 |
| 2 | 4 | 2 |
| 2 | 4 | 2 |
| 3 | 7 | 3 |
| 1 | 4 | 2 |

| | | |
|---|---|---|
| 2 | 4 | 2 |
| 2 | 4 | 2 |
| 2 | 4 | 2 |
| 2 | 1 | 2 |
| 3 | 7 | 3 |
| 2 | 4 | 2 |
| 2 | 4 | 2 |
| 2 | 4 | 2 |
| 3 | 7 | 3 |
| 3 | 7 | 3 |
| 1 | 1 | 2 |
| 2 | 4 | 2 |
| 3 | 7 | 3 |
| 2 | 4 | 2 |
| 2 | 4 | 2 |
| 3 | 1 | 2 |
| 2 | 1 | 2 |
| 2 | 4 | 2 |
| 3 | 7 | 3 |
| 3 | 7 | 3 |
| 2 | 3 | 2 |
| 3 | 7 | 3 |
| 3 | 7 | 3 |
| 2 | 4 | 2 |
| 3 | 7 | 3 |
| 3 | 7 | 3 |
| 3 | 7 | 3 |
| 3 | 7 | 3 |
| 2 | 3 | 2 |
| 3 | 7 | 3 |
| 2 | 4 | 2 |
| 3 | 7 | 3 |
| 3 | 7 | 3 |
| 3 | 7 | 3 |
| 2 | 1 | 2 |
| 2 | 1 | 2 |
| 3 | 7 | 3 |
| 3 | 7 | 3 |
| 3 | 7 | 3 |
| 3 | 7 | 3 |
| 3 | 7 | 3 |
| 2 | 4 | 1 |
| 3 | 7 | 3 |
| 3 | 7 | 3 |
| 2 | 4 | 2 |
| 2 | 4 | 1 |
| 3 | 7 | 3 |
| 3 | 7 | 3 |

| | | |
|---|---|---|
| 2 | 4 | 1 |
| 2 | 4 | 1 |
| 2 | 3 | 2 |
| 3 | 7 | 3 |
| 3 | 7 | 3 |
| 3 | 7 | 3 |
| 3 | 7 | 3 |
| 3 | 7 | 3 |
| 3 | 7 | 3 |
| 3 | 7 | 3 |
| 3 | 7 | 3 |
| 2 | 3 | 1 |
| 2 | 3 | 2 |
| 3 | 7 | 3 |
| 3 | 7 | 3 |
| 3 | 7 | 3 |
| 2 | 4 | 2 |
| 2 | 4 | 2 |
| 3 | 7 | 3 |
| 3 | 7 | 3 |
| 3 | 7 | 3 |
| 3 | 7 | 3 |
| 3 | 7 | 3 |
| 2 | 3 | 2 |
| 3 | 7 | 3 |
| 3 | 7 | 3 |
| 3 | 7 | 3 |
| 2 | 4 | 2 |
| 3 | 7 | 3 |
| 3 | 7 | 3 |
| 3 | 7 | 3 |
| 3 | 7 | 3 |
| 3 | 7 | 3 |
| 3 | 7 | 3 |
| 3 | 7 | 3 |
| 2 | 1 | 2 |
| 3 | 7 | 3 |
| 3 | 7 | 3 |
| 3 | 7 | 3 |
| 3 | 7 | 3 |
| 3 | 7 | 3 |
| 3 | 7 | 3 |
| 3 | 7 | 3 |
| 2 | 3 | 2 |
| 3 | 7 | 3 |
| 2 | 3 | 2 |
| 3 | 7 | 3 |
| 2 | 3 | 2 |

| | | |
|---|---|---|
| 3 | 7 | 3 |
| 2 | 3 | 2 |
| 3 | 7 | 3 |
| 2 | 3 | 2 |
| 3 | 7 | 3 |
| 3 | 7 | 3 |
| 3 | 7 | 3 |
| 3 | 7 | 3 |
| 3 | 7 | 3 |
| 3 | 7 | 3 |
| 3 | 7 | 3 |
| 3 | 7 | 3 |
| 3 | 7 | 3 |
| 3 | 7 | 3 |
| 3 | 7 | 3 |
| 3 | 7 | 3 |
| 1 | 3 | 2 |
| 3 | 7 | 3 |
| 3 | 7 | 3 |
| 1 | 3 | 2 |
| 3 | 7 | 3 |
| 3 | 7 | 3 |
| 2 | 4 | 2 |
| 3 | 7 | 3 |
| 3 | 7 | 3 |
| 3 | 7 | 3 |
| 3 | 7 | 3 |
| 1 | 3 | 2 |
| 2 | 3 | 2 |
| 3 | 7 | 3 |
| 3 | 7 | 3 |
| 1 | 7 | 2 |
| 3 | 7 | 3 |
| 3 | 7 | 3 |
| 3 | 7 | 3 |
| 3 | 7 | 3 |
| 3 | 7 | 3 |
| 1 | 6 | 2 |
| 3 | 7 | 3 |
| 3 | 7 | 3 |
| 3 | 7 | 3 |
| 3 | 7 | 3 |
| 1 | 7 | 3 |
| 3 | 7 | 3 |
| 3 | 7 | 3 |
| 3 | 7 | 3 |
| 1 | 2 | 2 |
| 1 | 7 | 3 |

| | | |
|---|---|---|
| 2 | 1 | 1 |
| 3 | 7 | 3 |
| 1 | 6 | 2 |
| 3 | 7 | 3 |
| 3 | 7 | 3 |
| 3 | 7 | 3 |
| 3 | 7 | 3 |
| 3 | 7 | 3 |
| 1 | 7 | 2 |
| 3 | 7 | 3 |
| 3 | 7 | 3 |
| 3 | 7 | 3 |
| 3 | 7 | 3 |
| 3 | 7 | 3 |
| 3 | 7 | 3 |
| 3 | 7 | 3 |
| 3 | 7 | 3 |
| 1 | 3 | 2 |
| 3 | 7 | 3 |
| 3 | 7 | 3 |
| 3 | 7 | 3 |
| 3 | 7 | 3 |
| 1 | 3 | 2 |
| 3 | 7 | 3 |
| 1 | 6 | 2 |
| 3 | 7 | 3 |
| 3 | 7 | 3 |
| 3 | 7 | 3 |
| 3 | 7 | 3 |
| 1 | 5 | 1 |
| 3 | 7 | 3 |
| 1 | 7 | 2 |
| 1 | 2 | 2 |
| 3 | 7 | 3 |
| 3 | 7 | 3 |
| 3 | 7 | 3 |
| 3 | 7 | 3 |
| 3 | 7 | 3 |
| 3 | 7 | 3 |
| 3 | 7 | 3 |
| 3 | 7 | 3 |
| 3 | 7 | 3 |
| 3 | 7 | 3 |
| 3 | 7 | 3 |
| 3 | 7 | 3 |
| 2 | 2 | 1 |
| 1 | 6 | 2 |
| 3 | 7 | 3 |

| | | |
|---|---|---|
| 3 | 7 | 3 |
| 3 | 7 | 3 |
| 3 | 7 | 3 |
| 3 | 7 | 3 |
| 3 | 7 | 3 |
| 3 | 7 | 3 |
| 3 | 7 | 3 |
| 3 | 7 | 3 |
| 2 | 5 | 2 |
| 3 | 7 | 3 |
| 3 | 7 | 3 |
| 3 | 7 | 3 |
| 2 | 3 | 2 |
| 1 | 6 | 2 |
| 2 | 5 | 1 |
| 3 | 7 | 3 |
| 3 | 7 | 3 |
| 3 | 7 | 3 |
| 3 | 7 | 3 |
| 3 | 7 | 3 |
| 1 | 2 | 2 |
| 3 | 7 | 3 |
| 3 | 7 | 3 |
| 3 | 7 | 3 |
| 3 | 7 | 3 |
| 3 | 7 | 3 |
| 3 | 7 | 3 |
| 3 | 7 | 3 |
| 3 | 7 | 3 |
| 1 | 7 | 2 |
| 3 | 7 | 3 |
| 3 | 7 | 3 |
| 3 | 7 | 3 |
| 3 | 7 | 3 |
| 3 | 7 | 3 |
| 3 | 7 | 3 |
| 3 | 7 | 3 |
| 2 | 7 | 3 |
| 3 | 7 | 3 |
| 1 | 3 | 3 |
| 1 | 6 | 2 |
| 3 | 7 | 3 |
| 3 | 7 | 3 |
| 3 | 7 | 3 |
| 3 | 7 | 3 |
| 3 | 7 | 3 |
| 3 | 7 | 3 |
| 3 | 7 | 3 |

| | | |
|---|---|---|
| 3 | 7 | 3 |
| 3 | 7 | 3 |
| 2 | 3 | 1 |
| 3 | 7 | 3 |
| 1 | 1 | 2 |
| 2 | 6 | 1 |
| 3 | 7 | 3 |
| 1 | 7 | 2 |
| 3 | 7 | 3 |
| 2 | 4 | 2 |
| 3 | 7 | 3 |
| 2 | 5 | 1 |
| 2 | 5 | 1 |
| 3 | 7 | 3 |
| 3 | 7 | 3 |
| 3 | 7 | 3 |
| 2 | 1 | 2 |
| 3 | 7 | 3 |
| 3 | 7 | 3 |
| 3 | 7 | 3 |
| 3 | 7 | 3 |
| 1 | 3 | 2 |
| 3 | 7 | 3 |
| 3 | 7 | 3 |
| 3 | 7 | 3 |
| 3 | 7 | 3 |
| 3 | 7 | 3 |
| 3 | 7 | 3 |
| 3 | 7 | 3 |
| 3 | 7 | 3 |
| 3 | 7 | 3 |
| 3 | 7 | 3 |
| 3 | 7 | 3 |
| 3 | 7 | 3 |
| 3 | 2 | 3 |
| 3 | 7 | 3 |
| 3 | 7 | 3 |
| 3 | 7 | 3 |
| 3 | 7 | 3 |
| 3 | 7 | 3 |
| 3 | 7 | 3 |
| 3 | 7 | 3 |
| 3 | 7 | 3 |
| 3 | 7 | 3 |
| 3 | 7 | 3 |
| 3 | 7 | 3 |
| 3 | 7 | 3 |
| 3 | 7 | 3 |

| | | |
|---|---|---|
| 3 | 7 | 3 |
| 3 | 7 | 3 |
| 3 | 7 | 3 |
| 2 | 3 | 2 |
| 2 | 4 | 2 |
| 2 | 4 | 1 |
| 3 | 7 | 3 |
| 3 | 7 | 3 |
| 3 | 7 | 3 |
| 3 | 7 | 3 |
| 3 | 7 | 3 |
| 3 | 7 | 3 |
| 3 | 7 | 3 |
| 3 | 7 | 3 |
| 3 | 7 | 3 |
| 1 | 7 | 2 |
| 3 | 7 | 3 |
| 3 | 7 | 3 |
| 3 | 7 | 3 |
| 3 | 7 | 3 |
| 3 | 7 | 3 |
| 2 | 4 | 2 |
| 2 | 4 | 2 |
| 2 | 4 | 2 |
| 2 | 3 | 1 |
| 2 | 3 | 1 |
| 2 | 4 | 2 |
| 2 | 3 | 1 |
| 2 | 3 | 1 |
| 2 | 3 | 2 |
| 2 | 3 | 2 |
| 1 | 1 | 2 |
| 1 | 1 | 2 |
| 3 | 7 | 3 |
| 3 | 7 | 3 |
| 2 | 3 | 1 |
| 3 | 7 | 3 |
| 2 | 3 | 2 |
| 3 | 7 | 3 |
| 3 | 7 | 3 |
| 3 | 7 | 3 |
| 3 | 7 | 3 |
| 1 | 1 | 2 |
| 3 | 7 | 3 |
| 3 | 7 | 3 |
| 3 | 7 | 3 |
| 3 | 7 | 3 |
| 3 | 7 | 3 |

| | | |
|---|---|---|
| 3 | 7 | 3 |
| 3 | 7 | 3 |
| 2 | 3 | 2 |
| 3 | 7 | 3 |
| 2 | 3 | 2 |
| 3 | 7 | 3 |
| 3 | 7 | 3 |
| 3 | 7 | 3 |
| 2 | 3 | 2 |
| 3 | 7 | 3 |
| 3 | 7 | 3 |
| 3 | 7 | 3 |
| 2 | 4 | 2 |
| 3 | 7 | 3 |
| 3 | 7 | 3 |
| 3 | 7 | 3 |
| 3 | 7 | 3 |
| 2 | 4 | 2 |
| 2 | 3 | 1 |
| 2 | 4 | 1 |
| 2 | 4 | 1 |
| 3 | 7 | 3 |
| 3 | 7 | 3 |
| 3 | 7 | 3 |
| 3 | 7 | 3 |
| 3 | 7 | 3 |
| 3 | 7 | 3 |
| 3 | 7 | 3 |
| 2 | 3 | 1 |
| 3 | 7 | 3 |
| 3 | 7 | 3 |
| 3 | 7 | 3 |
| 2 | 3 | 1 |
| 2 | 3 | 1 |
| 3 | 7 | 3 |
| 3 | 7 | 3 |
| 3 | 7 | 3 |
| 3 | 7 | 3 |
| 3 | 7 | 3 |
| 3 | 7 | 3 |
| 3 | 7 | 3 |
| 3 | 7 | 3 |
| 3 | 7 | 3 |
| 3 | 7 | 3 |
| 3 | 7 | 3 |
| 3 | 7 | 3 |
| 3 | 7 | 3 |
| 3 | 7 | 3 |

| | | |
|---|---|---|
| 3 | 7 | 3 |
| 3 | 7 | 3 |
| 3 | 7 | 3 |
| 3 | 7 | 3 |
| 3 | 7 | 3 |
| 3 | 7 | 3 |
| 3 | 7 | 3 |
| 1 | 7 | 3 |
| 3 | 7 | 3 |
| 3 | 7 | 3 |
| 3 | 7 | 3 |
| 3 | 7 | 3 |
| 2 | 3 | 1 |
| 3 | 7 | 3 |
| 1 | 6 | 2 |
| 3 | 7 | 3 |
| 2 | 4 | 1 |
| 3 | 7 | 3 |
| 2 | 4 | 1 |
| 3 | 7 | 3 |
| 3 | 7 | 3 |
| 3 | 7 | 3 |
| 2 | 4 | 1 |
| 3 | 7 | 3 |
| 2 | 4 | 1 |
| 2 | 5 | 1 |
| 3 | 7 | 3 |
| 2 | 4 | 1 |
| 2 | 4 | 1 |
| 1 | 2 | 2 |
| 3 | 7 | 3 |
| 3 | 7 | 3 |
| 3 | 7 | 3 |
| 2 | 4 | 1 |
| 2 | 4 | 1 |
| 3 | 7 | 3 |
| 2 | 4 | 1 |
| 3 | 7 | 3 |
| 2 | 4 | 1 |
| 3 | 7 | 3 |
| 2 | 2 | 1 |
| 2 | 4 | 1 |
| 2 | 4 | 1 |
| 2 | 4 | 1 |

| | | |
|---|---|---|
| 1 no | 1 SM | 1 yes |
| 2 yes | 2 SL | 2 no |
| 3 NA | 3 internet | 3 NA |

4 friends
5 family

6 other
7 NA

| StartDate | EndDate | Duration (in seconds) | distributionChannel | DistributionChannel | gender |
|---|---|---|---|---|---|
| Start Date | End Date | Duration (in seconds) | | Distribution Channel | |
| 15/03/2020 23:32 | 15/03/2020 23:33 | 83 | 2 | anonymous | 1 |
| 15/03/2020 23:32 | 15/03/2020 23:34 | 109 | 2 | anonymous | 1 |
| 15/03/2020 23:31 | 15/03/2020 23:45 | 797 | 2 | anonymous | 1 |
| 15/03/2020 23:55 | 15/03/2020 23:57 | 121 | 2 | anonymous | 1 |
| 16/03/2020 00:06 | 16/03/2020 00:07 | 64 | 2 | anonymous | 1 |
| 16/03/2020 00:08 | 16/03/2020 00:10 | 107 | 2 | anonymous | 1 |
| 16/03/2020 00:24 | 16/03/2020 00:25 | 83 | 2 | anonymous | 1 |
| 16/03/2020 00:31 | 16/03/2020 00:32 | 60 | 2 | anonymous | 2 |
| 16/03/2020 00:36 | 16/03/2020 00:40 | 239 | 2 | anonymous | 1 |
| 16/03/2020 00:44 | 16/03/2020 00:46 | 114 | 2 | anonymous | 1 |
| 16/03/2020 00:48 | 16/03/2020 00:49 | 34 | 2 | anonymous | 1 |
| 16/03/2020 00:51 | 16/03/2020 00:51 | 32 | 2 | anonymous | 2 |
| 16/03/2020 00:59 | 16/03/2020 01:02 | 127 | 2 | anonymous | 1 |
| 16/03/2020 01:18 | 16/03/2020 01:19 | 78 | 2 | anonymous | 1 |
| 16/03/2020 01:44 | 16/03/2020 01:45 | 80 | 2 | anonymous | 1 |
| 16/03/2020 02:06 | 16/03/2020 02:08 | 134 | 2 | anonymous | 1 |
| 16/03/2020 02:09 | 16/03/2020 02:10 | 83 | 2 | anonymous | 1 |
| 16/03/2020 02:11 | 16/03/2020 02:12 | 45 | 2 | anonymous | 1 |
| 16/03/2020 02:32 | 16/03/2020 02:33 | 94 | 2 | anonymous | 2 |
| 16/03/2020 03:11 | 16/03/2020 03:16 | 300 | 2 | anonymous | 1 |
| 16/03/2020 03:21 | 16/03/2020 03:24 | 157 | 2 | anonymous | 1 |
| 16/03/2020 03:28 | 16/03/2020 03:30 | 106 | 2 | anonymous | 1 |
| 16/03/2020 03:31 | 16/03/2020 03:32 | 85 | 2 | anonymous | 1 |
| 16/03/2020 04:24 | 16/03/2020 04:24 | 44 | 2 | anonymous | 1 |
| 16/03/2020 04:34 | 16/03/2020 04:36 | 107 | 2 | anonymous | 1 |
| 16/03/2020 04:41 | 16/03/2020 04:42 | 85 | 2 | anonymous | 1 |
| 16/03/2020 05:26 | 16/03/2020 05:31 | 300 | 2 | anonymous | 2 |
| 16/03/2020 05:30 | 16/03/2020 05:34 | 187 | 2 | anonymous | 1 |
| 16/03/2020 05:36 | 16/03/2020 05:37 | 43 | 2 | anonymous | 1 |
| 16/03/2020 06:21 | 16/03/2020 06:23 | 134 | 2 | anonymous | 3 |
| 16/03/2020 06:57 | 16/03/2020 07:02 | 314 | 2 | anonymous | 2 |
| 16/03/2020 07:18 | 16/03/2020 07:19 | 38 | 2 | anonymous | 1 |
| 16/03/2020 07:44 | 16/03/2020 07:45 | 78 | 2 | anonymous | 2 |
| 16/03/2020 08:44 | 16/03/2020 08:45 | 50 | 2 | anonymous | 2 |
| 16/03/2020 09:23 | 16/03/2020 09:26 | 205 | 2 | anonymous | 2 |
| 16/03/2020 09:44 | 16/03/2020 09:50 | 366 | 2 | anonymous | 1 |
| 16/03/2020 10:55 | 16/03/2020 10:56 | 71 | 2 | anonymous | 2 |
| 16/03/2020 11:58 | 16/03/2020 12:00 | 101 | 2 | anonymous | 1 |
| 16/03/2020 14:27 | 16/03/2020 14:28 | 45 | 2 | anonymous | 1 |
| 16/03/2020 17:38 | 16/03/2020 17:39 | 63 | 2 | anonymous | 2 |
| 17/03/2020 01:07 | 17/03/2020 01:09 | 139 | 2 | anonymous | 1 |
| 17/03/2020 01:55 | 17/03/2020 01:56 | 45 | 2 | anonymous | 1 |
| 17/03/2020 01:55 | 17/03/2020 01:56 | 55 | 2 | anonymous | 1 |
| 17/03/2020 01:57 | 17/03/2020 01:57 | 18 | 2 | anonymous | 2 |
| 17/03/2020 01:55 | 17/03/2020 01:57 | 103 | 2 | anonymous | 1 |
| 17/03/2020 01:57 | 17/03/2020 01:58 | 69 | 2 | anonymous | 1 |
| 17/03/2020 02:13 | 17/03/2020 02:13 | 35 | 2 | anonymous | 1 |
| 17/03/2020 03:01 | 17/03/2020 03:04 | 172 | 2 | anonymous | 1 |
| 17/03/2020 03:06 | 17/03/2020 03:07 | 38 | 2 | anonymous | 1 |
| 17/03/2020 03:05 | 17/03/2020 03:07 | 121 | 2 | anonymous | 1 |

| | | | | | |
|---|---|---|---|---|---|
| 17/03/2020 03:43 | 17/03/2020 03:52 | 582 | 2 | anonymous | 2 |
| 17/03/2020 04:30 | 17/03/2020 04:32 | 103 | 2 | anonymous | 1 |
| 17/03/2020 04:37 | 17/03/2020 04:38 | 61 | 2 | anonymous | 1 |
| 17/03/2020 04:48 | 17/03/2020 04:48 | 36 | 2 | anonymous | 1 |
| 17/03/2020 05:27 | 17/03/2020 05:33 | 314 | 2 | anonymous | 1 |
| 17/03/2020 05:56 | 17/03/2020 05:57 | 71 | 2 | anonymous | 1 |
| 17/03/2020 07:09 | 17/03/2020 07:09 | 47 | 2 | anonymous | 1 |
| 17/03/2020 07:46 | 17/03/2020 07:53 | 401 | 2 | anonymous | 1 |
| 17/03/2020 09:32 | 17/03/2020 09:34 | 98 | 2 | anonymous | 1 |
| 17/03/2020 12:58 | 17/03/2020 13:05 | 383 | 2 | anonymous | 1 |
| 18/03/2020 01:22 | 18/03/2020 01:26 | 221 | 2 | anonymous | 2 |
| 18/03/2020 10:15 | 18/03/2020 10:19 | 254 | 2 | anonymous | 2 |
| 19/03/2020 01:25 | 19/03/2020 01:26 | 64 | 2 | anonymous | 2 |
| 16/03/2020 03:54 | 19/03/2020 09:06 | 277877 | 2 | anonymous | 2 |
| 19/03/2020 15:47 | 19/03/2020 15:50 | 188 | 2 | anonymous | 1 |
| 20/03/2020 15:18 | 20/03/2020 15:21 | 165 | 3 | qr | 2 |
| 22/03/2020 03:32 | 22/03/2020 03:33 | 32 | 2 | anonymous | 2 |
| 22/03/2020 03:41 | 22/03/2020 03:42 | 83 | 2 | anonymous | 1 |
| 22/03/2020 03:42 | 22/03/2020 03:43 | 58 | 2 | anonymous | 1 |
| 22/03/2020 03:47 | 22/03/2020 03:52 | 284 | 2 | anonymous | 2 |
| 22/03/2020 04:05 | 22/03/2020 04:19 | 836 | 2 | anonymous | 1 |
| 22/03/2020 05:30 | 22/03/2020 05:32 | 106 | 2 | anonymous | 1 |
| 22/03/2020 06:13 | 22/03/2020 06:14 | 46 | 2 | anonymous | 1 |
| 22/03/2020 07:11 | 22/03/2020 07:12 | 58 | 2 | anonymous | 2 |
| 23/03/2020 01:21 | 23/03/2020 01:23 | 127 | 2 | anonymous | 1 |
| 23/03/2020 09:19 | 23/03/2020 09:20 | 68 | 2 | anonymous | 1 |
| 20/03/2020 23:31 | 24/03/2020 10:08 | 297445 | 2 | anonymous | 1 |
| 26/03/2020 12:48 | 26/03/2020 12:50 | 123 | 3 | social | 2 |
| 01/04/2020 17:30 | 01/04/2020 17:31 | 101 | 2 | anonymous | 2 |
| 04/04/2020 18:00 | 04/04/2020 18:03 | 218 | 3 | social | 2 |
| 07/04/2020 13:07 | 07/04/2020 13:10 | 122 | 2 | anonymous | 2 |
| 12/04/2020 08:36 | 12/04/2020 08:36 | 38 | 2 | anonymous | 1 |
| 12/04/2020 08:38 | 12/04/2020 08:40 | 90 | 2 | anonymous | 1 |
| 12/04/2020 08:44 | 12/04/2020 08:45 | 106 | 2 | anonymous | 2 |
| 12/04/2020 08:46 | 12/04/2020 08:47 | 71 | 2 | anonymous | 2 |
| 12/04/2020 08:47 | 12/04/2020 08:50 | 179 | 2 | anonymous | 2 |
| 12/04/2020 08:52 | 12/04/2020 08:55 | 194 | 2 | anonymous | 2 |
| 12/04/2020 08:56 | 12/04/2020 08:57 | 62 | 2 | anonymous | 2 |
| 12/04/2020 09:00 | 12/04/2020 09:01 | 54 | 2 | anonymous | 2 |
| 12/04/2020 09:04 | 12/04/2020 09:05 | 62 | 2 | anonymous | 2 |
| 12/04/2020 09:05 | 12/04/2020 09:07 | 88 | 2 | anonymous | 2 |
| 12/04/2020 15:15 | 12/04/2020 15:18 | 166 | 2 | anonymous | 1 |
| 12/04/2020 15:18 | 12/04/2020 15:21 | 141 | 2 | anonymous | 1 |
| 12/04/2020 15:23 | 12/04/2020 15:24 | 81 | 2 | anonymous | 2 |
| 12/04/2020 15:24 | 12/04/2020 15:26 | 100 | 2 | anonymous | 2 |
| 12/04/2020 15:26 | 12/04/2020 15:27 | 61 | 2 | anonymous | 2 |
| 13/04/2020 08:23 | 13/04/2020 08:24 | 70 | 2 | anonymous | 2 |
| 13/04/2020 08:24 | 13/04/2020 08:25 | 54 | 2 | anonymous | 2 |
| 13/04/2020 08:25 | 13/04/2020 08:26 | 25 | 2 | anonymous | 1 |
| 14/04/2020 07:38 | 14/04/2020 07:39 | 75 | 2 | anonymous | 2 |
| 14/04/2020 07:40 | 14/04/2020 07:40 | 49 | 2 | anonymous | 2 |
| 14/04/2020 09:15 | 14/04/2020 09:16 | 78 | 2 | anonymous | 2 |

| | | | | | |
|---|---|---|---|---|---|
| 14/04/2020 09:17 | 14/04/2020 09:17 | 27 | 2 | anonymous | 2 |
| 14/04/2020 09:17 | 14/04/2020 09:17 | 11 | 2 | anonymous | 2 |
| 14/04/2020 09:17 | 14/04/2020 09:19 | 88 | 2 | anonymous | 2 |
| 14/04/2020 09:19 | 14/04/2020 09:20 | 62 | 2 | anonymous | 2 |
| 14/04/2020 09:30 | 14/04/2020 09:30 | 11 | 2 | anonymous | 2 |
| 14/04/2020 09:33 | 14/04/2020 09:35 | 88 | 2 | anonymous | 2 |
| 14/04/2020 12:44 | 14/04/2020 12:45 | 51 | 3 | social | 2 |
| 14/04/2020 16:34 | 14/04/2020 16:39 | 328 | 2 | anonymous | 2 |
| 15/04/2020 07:07 | 15/04/2020 07:07 | 56 | 2 | anonymous | 2 |
| 15/04/2020 07:34 | 15/04/2020 07:35 | 63 | 2 | anonymous | 2 |
| 15/04/2020 07:35 | 15/04/2020 07:35 | 12 | 2 | anonymous | 1 |
| 15/04/2020 07:35 | 15/04/2020 07:35 | 11 | 2 | anonymous | 1 |
| 15/04/2020 07:35 | 15/04/2020 07:36 | 50 | 2 | anonymous | 1 |
| 15/04/2020 07:36 | 15/04/2020 07:37 | 13 | 2 | anonymous | 2 |
| 15/04/2020 07:37 | 15/04/2020 07:37 | 11 | 2 | anonymous | 1 |
| 15/04/2020 07:40 | 15/04/2020 07:41 | 56 | 2 | anonymous | 2 |
| 15/04/2020 07:41 | 15/04/2020 07:41 | 14 | 2 | anonymous | 2 |
| 15/04/2020 07:41 | 15/04/2020 07:42 | 11 | 2 | anonymous | 2 |
| 15/04/2020 07:42 | 15/04/2020 07:42 | 10 | 2 | anonymous | 1 |
| 15/04/2020 07:44 | 15/04/2020 07:44 | 12 | 2 | anonymous | 1 |
| 15/04/2020 07:44 | 15/04/2020 07:45 | 62 | 2 | anonymous | 2 |
| 15/04/2020 07:45 | 15/04/2020 07:46 | 12 | 2 | anonymous | 2 |
| 15/04/2020 07:47 | 15/04/2020 07:48 | 60 | 2 | anonymous | 2 |
| 15/04/2020 07:49 | 15/04/2020 07:49 | 21 | 2 | anonymous | 2 |
| 15/04/2020 10:08 | 15/04/2020 10:08 | 4 | 3 | social | 3 |
| 15/04/2020 10:08 | 15/04/2020 10:08 | 4 | 3 | social | 3 |
| 15/04/2020 18:54 | 15/04/2020 18:55 | 43 | 2 | anonymous | 2 |
| 16/04/2020 18:27 | 16/04/2020 18:28 | 79 | 2 | anonymous | 2 |
| 16/04/2020 18:28 | 16/04/2020 18:29 | 29 | 2 | anonymous | 1 |
| 16/04/2020 18:29 | 16/04/2020 18:29 | 11 | 2 | anonymous | 2 |
| 16/04/2020 18:29 | 16/04/2020 18:29 | 15 | 2 | anonymous | 1 |
| 16/04/2020 18:30 | 16/04/2020 18:30 | 10 | 2 | anonymous | 2 |
| 16/04/2020 18:30 | 16/04/2020 18:30 | 12 | 2 | anonymous | 1 |
| 16/04/2020 18:31 | 16/04/2020 18:32 | 93 | 2 | anonymous | 2 |
| 16/04/2020 18:32 | 16/04/2020 18:32 | 10 | 2 | anonymous | 2 |
| 16/04/2020 23:19 | 16/04/2020 23:20 | 75 | 2 | anonymous | 2 |
| 21/04/2020 13:20 | 21/04/2020 13:21 | 75 | 2 | anonymous | 2 |
| 26/04/2020 15:31 | 26/04/2020 15:32 | 75 | 2 | anonymous | 2 |
| 26/04/2020 15:33 | 26/04/2020 15:33 | 11 | 2 | anonymous | 1 |
| 26/04/2020 15:36 | 26/04/2020 15:36 | 11 | 2 | anonymous | 1 |
| 26/04/2020 15:36 | 26/04/2020 15:38 | 90 | 2 | anonymous | 1 |
| 26/04/2020 15:38 | 26/04/2020 15:41 | 131 | 2 | anonymous | 2 |
| 26/04/2020 15:41 | 26/04/2020 15:42 | 50 | 2 | anonymous | 2 |
| 26/04/2020 15:42 | 26/04/2020 15:42 | 11 | 2 | anonymous | 1 |
| 26/04/2020 17:04 | 26/04/2020 17:05 | 19 | 3 | social | 1 |
| 26/04/2020 19:01 | 26/04/2020 19:01 | 12 | 3 | social | 1 |
| 26/04/2020 19:01 | 26/04/2020 19:01 | 12 | 3 | social | 1 |
| 26/04/2020 19:03 | 26/04/2020 19:03 | 17 | 3 | social | 2 |
| 26/04/2020 19:36 | 26/04/2020 19:36 | 13 | 3 | social | 2 |
| 26/04/2020 19:36 | 26/04/2020 19:37 | 11 | 3 | social | 1 |
| 26/04/2020 19:37 | 26/04/2020 19:37 | 10 | 3 | social | 2 |
| 26/04/2020 19:37 | 26/04/2020 19:38 | 53 | 3 | social | 2 |

| | | | | | |
|---|---|---|---|---|---|
| 28/04/2020 09:43 | 28/04/2020 09:44 | 56 | 2 | anonymous | 2 |
| 28/04/2020 09:44 | 28/04/2020 09:44 | 9 | 2 | anonymous | 1 |
| 28/04/2020 09:44 | 28/04/2020 09:44 | 8 | 2 | anonymous | 2 |
| 28/04/2020 09:44 | 28/04/2020 09:44 | 8 | 2 | anonymous | 1 |
| 28/04/2020 09:45 | 28/04/2020 09:45 | 47 | 2 | anonymous | 2 |
| 30/04/2020 18:11 | 30/04/2020 18:12 | 84 | 2 | anonymous | 2 |
| 30/04/2020 18:12 | 30/04/2020 18:12 | 11 | 2 | anonymous | 2 |
| 30/04/2020 18:13 | 30/04/2020 18:13 | 10 | 2 | anonymous | 1 |
| 30/04/2020 18:13 | 30/04/2020 18:13 | 9 | 2 | anonymous | 2 |
| 30/04/2020 18:14 | 30/04/2020 18:14 | 12 | 2 | anonymous | 2 |
| 30/04/2020 18:14 | 30/04/2020 18:14 | 14 | 2 | anonymous | 1 |
| 01/05/2020 19:43 | 01/05/2020 19:44 | 83 | 2 | anonymous | 2 |
| 01/05/2020 19:45 | 01/05/2020 19:45 | 12 | 2 | anonymous | 1 |
| 01/05/2020 19:45 | 01/05/2020 19:45 | 10 | 2 | anonymous | 2 |
| 01/05/2020 19:45 | 01/05/2020 19:45 | 9 | 2 | anonymous | 1 |
| 02/05/2020 06:28 | 02/05/2020 06:29 | 81 | 2 | anonymous | 2 |
| 02/05/2020 06:29 | 02/05/2020 06:30 | 11 | 2 | anonymous | 1 |
| 02/05/2020 06:30 | 02/05/2020 06:30 | 10 | 2 | anonymous | 2 |
| 02/05/2020 06:30 | 02/05/2020 06:30 | 11 | 2 | anonymous | 1 |
| 02/05/2020 06:31 | 02/05/2020 06:31 | 10 | 2 | anonymous | 2 |
| 02/05/2020 06:51 | 02/05/2020 06:51 | 12 | 2 | anonymous | 1 |
| 02/05/2020 08:11 | 02/05/2020 08:17 | 307 | 2 | anonymous | 1 |
| 02/05/2020 08:28 | 02/05/2020 08:29 | 92 | 2 | anonymous | 1 |
| 02/05/2020 08:29 | 02/05/2020 08:32 | 177 | 2 | anonymous | 2 |
| 02/05/2020 08:55 | 02/05/2020 08:55 | 13 | 2 | anonymous | 2 |
| 05/05/2020 21:06 | 05/05/2020 21:06 | 17 | 2 | anonymous | 2 |
| 05/05/2020 21:06 | 05/05/2020 21:06 | 10 | 2 | anonymous | 2 |
| 05/05/2020 21:06 | 05/05/2020 21:06 | 9 | 2 | anonymous | 1 |
| 05/05/2020 21:06 | 05/05/2020 21:07 | 11 | 2 | anonymous | 1 |
| 05/05/2020 21:07 | 05/05/2020 21:07 | 11 | 2 | anonymous | 2 |
| 08/05/2020 18:12 | 08/05/2020 18:13 | 23 | 2 | anonymous | 1 |
| 08/05/2020 18:13 | 08/05/2020 18:14 | 65 | 2 | anonymous | 1 |
| 08/05/2020 18:14 | 08/05/2020 18:14 | 14 | 2 | anonymous | 2 |
| 08/05/2020 18:15 | 08/05/2020 18:16 | 80 | 2 | anonymous | 2 |
| 08/05/2020 18:16 | 08/05/2020 18:17 | 11 | 2 | anonymous | 2 |
| 10/05/2020 10:40 | 10/05/2020 10:42 | 72 | 2 | anonymous | 1 |
| 10/05/2020 10:42 | 10/05/2020 10:42 | 10 | 2 | anonymous | 1 |
| 10/05/2020 10:42 | 10/05/2020 10:43 | 71 | 2 | anonymous | 2 |
| 10/05/2020 10:44 | 10/05/2020 10:45 | 10 | 2 | anonymous | 2 |
| 10/05/2020 10:45 | 10/05/2020 10:46 | 101 | 2 | anonymous | 2 |
| 12/05/2020 02:34 | 12/05/2020 02:35 | 36 | 2 | anonymous | 1 |
| 12/05/2020 02:34 | 12/05/2020 02:35 | 47 | 2 | anonymous | 1 |
| 12/05/2020 02:34 | 12/05/2020 02:35 | 47 | 2 | anonymous | 2 |
| 12/05/2020 02:34 | 12/05/2020 02:35 | 55 | 2 | anonymous | 1 |
| 12/05/2020 02:34 | 12/05/2020 02:35 | 66 | 2 | anonymous | 1 |
| 12/05/2020 02:34 | 12/05/2020 02:35 | 58 | 2 | anonymous | 1 |
| 12/05/2020 02:35 | 12/05/2020 02:35 | 36 | 2 | anonymous | 1 |
| 12/05/2020 02:35 | 12/05/2020 02:38 | 130 | 2 | anonymous | 1 |
| 12/05/2020 02:37 | 12/05/2020 02:38 | 57 | 2 | anonymous | 2 |
| 12/05/2020 02:37 | 12/05/2020 02:38 | 74 | 2 | anonymous | 1 |
| 12/05/2020 02:37 | 12/05/2020 02:38 | 87 | 2 | anonymous | 1 |
| 12/05/2020 02:38 | 12/05/2020 02:39 | 37 | 2 | anonymous | 2 |

| | | | | | |
|---|---|---|---|---|---|
| 12/05/2020 02:37 | 12/05/2020 02:39 | 127 | 2 | anonymous | 1 |
| 12/05/2020 02:38 | 12/05/2020 02:39 | 44 | 2 | anonymous | 1 |
| 12/05/2020 02:34 | 12/05/2020 02:39 | 332 | 2 | anonymous | 1 |
| 12/05/2020 02:39 | 12/05/2020 02:40 | 68 | 2 | anonymous | 1 |
| 12/05/2020 02:38 | 12/05/2020 02:41 | 191 | 2 | anonymous | 1 |
| 12/05/2020 02:41 | 12/05/2020 02:41 | 56 | 2 | anonymous | 2 |
| 12/05/2020 02:37 | 12/05/2020 02:42 | 324 | 2 | anonymous | 2 |
| 12/05/2020 02:35 | 12/05/2020 02:43 | 457 | 2 | anonymous | 1 |
| 12/05/2020 02:42 | 12/05/2020 02:44 | 122 | 2 | anonymous | 1 |
| 12/05/2020 02:43 | 12/05/2020 02:45 | 155 | 2 | anonymous | 2 |
| 12/05/2020 02:45 | 12/05/2020 02:46 | 45 | 2 | anonymous | 1 |
| 12/05/2020 02:41 | 12/05/2020 02:46 | 320 | 2 | anonymous | 2 |
| 12/05/2020 02:44 | 12/05/2020 02:46 | 168 | 2 | anonymous | 1 |
| 12/05/2020 02:45 | 12/05/2020 02:46 | 67 | 2 | anonymous | 1 |
| 12/05/2020 02:45 | 12/05/2020 02:47 | 112 | 2 | anonymous | 1 |
| 12/05/2020 02:45 | 12/05/2020 02:47 | 99 | 2 | anonymous | 1 |
| 12/05/2020 02:48 | 12/05/2020 02:49 | 78 | 2 | anonymous | 1 |
| 12/05/2020 02:44 | 12/05/2020 02:50 | 333 | 2 | anonymous | 2 |
| 12/05/2020 02:49 | 12/05/2020 02:50 | 72 | 2 | anonymous | 1 |
| 12/05/2020 02:50 | 12/05/2020 02:51 | 44 | 2 | anonymous | 2 |
| 12/05/2020 02:53 | 12/05/2020 02:54 | 38 | 2 | anonymous | 1 |
| 12/05/2020 02:52 | 12/05/2020 02:54 | 148 | 2 | anonymous | 1 |
| 12/05/2020 02:55 | 12/05/2020 02:57 | 129 | 2 | anonymous | 1 |
| 12/05/2020 02:56 | 12/05/2020 02:58 | 116 | 2 | anonymous | 1 |
| 12/05/2020 02:57 | 12/05/2020 02:58 | 35 | 2 | anonymous | 1 |
| 12/05/2020 02:58 | 12/05/2020 03:00 | 81 | 2 | anonymous | 3 |
| 12/05/2020 02:59 | 12/05/2020 03:01 | 118 | 2 | anonymous | 1 |
| 12/05/2020 03:01 | 12/05/2020 03:03 | 90 | 2 | anonymous | 1 |
| 12/05/2020 03:02 | 12/05/2020 03:03 | 35 | 2 | anonymous | 1 |
| 12/05/2020 03:02 | 12/05/2020 03:03 | 76 | 2 | anonymous | 1 |
| 12/05/2020 03:00 | 12/05/2020 03:04 | 249 | 2 | anonymous | 1 |
| 12/05/2020 03:05 | 12/05/2020 03:06 | 90 | 2 | anonymous | 1 |
| 12/05/2020 03:03 | 12/05/2020 03:07 | 251 | 2 | anonymous | 1 |
| 12/05/2020 03:07 | 12/05/2020 03:08 | 49 | 2 | anonymous | 1 |
| 12/05/2020 03:03 | 12/05/2020 03:10 | 373 | 2 | anonymous | 1 |
| 12/05/2020 03:09 | 12/05/2020 03:10 | 51 | 2 | anonymous | 1 |
| 12/05/2020 03:15 | 12/05/2020 03:19 | 207 | 2 | anonymous | 1 |
| 12/05/2020 03:19 | 12/05/2020 03:20 | 45 | 2 | anonymous | 1 |
| 12/05/2020 03:20 | 12/05/2020 03:21 | 64 | 2 | anonymous | 1 |
| 12/05/2020 03:20 | 12/05/2020 03:21 | 62 | 2 | anonymous | 1 |
| 12/05/2020 03:21 | 12/05/2020 03:23 | 95 | 2 | anonymous | 1 |
| 12/05/2020 03:26 | 12/05/2020 03:28 | 150 | 2 | anonymous | 2 |
| 12/05/2020 03:32 | 12/05/2020 03:33 | 80 | 2 | anonymous | 1 |
| 12/05/2020 03:30 | 12/05/2020 03:33 | 196 | 2 | anonymous | 1 |
| 12/05/2020 03:35 | 12/05/2020 03:35 | 39 | 2 | anonymous | 1 |
| 12/05/2020 03:38 | 12/05/2020 03:39 | 72 | 2 | anonymous | 1 |
| 12/05/2020 03:40 | 12/05/2020 03:41 | 91 | 2 | anonymous | 1 |
| 12/05/2020 03:40 | 12/05/2020 03:41 | 82 | 2 | anonymous | 1 |
| 12/05/2020 03:42 | 12/05/2020 03:43 | 95 | 2 | anonymous | 1 |
| 12/05/2020 03:37 | 12/05/2020 03:44 | 426 | 2 | anonymous | 1 |
| 12/05/2020 03:52 | 12/05/2020 03:53 | 38 | 2 | anonymous | 1 |
| 12/05/2020 03:53 | 12/05/2020 03:54 | 112 | 2 | anonymous | 2 |

| | | | | | |
|---|---|---|---|---|---|
| 12/05/2020 03:54 | 12/05/2020 03:55 | 79 | 2 | anonymous | 1 |
| 12/05/2020 03:58 | 12/05/2020 04:00 | 71 | 2 | anonymous | 1 |
| 12/05/2020 04:17 | 12/05/2020 04:19 | 142 | 2 | anonymous | 1 |
| 12/05/2020 04:20 | 12/05/2020 04:21 | 92 | 2 | anonymous | 1 |
| 12/05/2020 04:20 | 12/05/2020 04:21 | 100 | 2 | anonymous | 2 |
| 12/05/2020 04:22 | 12/05/2020 04:22 | 13 | 2 | anonymous | 1 |
| 12/05/2020 04:24 | 12/05/2020 04:24 | 35 | 2 | anonymous | 1 |
| 12/05/2020 04:30 | 12/05/2020 04:31 | 86 | 2 | anonymous | 1 |
| 12/05/2020 04:41 | 12/05/2020 04:43 | 77 | 2 | anonymous | 1 |
| 12/05/2020 04:42 | 12/05/2020 04:47 | 278 | 2 | anonymous | 1 |
| 12/05/2020 04:48 | 12/05/2020 04:49 | 90 | 2 | anonymous | 1 |
| 12/05/2020 04:53 | 12/05/2020 04:54 | 85 | 2 | anonymous | 1 |
| 12/05/2020 04:54 | 12/05/2020 04:58 | 228 | 2 | anonymous | 1 |
| 12/05/2020 04:58 | 12/05/2020 05:01 | 194 | 2 | anonymous | 2 |
| 12/05/2020 05:08 | 12/05/2020 05:09 | 75 | 2 | anonymous | 1 |
| 12/05/2020 05:18 | 12/05/2020 05:18 | 47 | 2 | anonymous | 1 |
| 12/05/2020 05:09 | 12/05/2020 05:19 | 582 | 2 | anonymous | 1 |
| 12/05/2020 05:19 | 12/05/2020 05:20 | 49 | 2 | anonymous | 1 |
| 12/05/2020 05:19 | 12/05/2020 05:20 | 72 | 2 | anonymous | 1 |
| 12/05/2020 05:24 | 12/05/2020 05:26 | 170 | 2 | anonymous | 1 |
| 12/05/2020 05:28 | 12/05/2020 05:30 | 106 | 2 | anonymous | 1 |
| 12/05/2020 05:32 | 12/05/2020 05:34 | 111 | 2 | anonymous | 1 |
| 12/05/2020 05:39 | 12/05/2020 05:41 | 75 | 2 | anonymous | 2 |
| 12/05/2020 05:41 | 12/05/2020 05:42 | 83 | 2 | anonymous | 1 |
| 12/05/2020 05:44 | 12/05/2020 05:44 | 30 | 2 | anonymous | 1 |
| 12/05/2020 05:38 | 12/05/2020 05:44 | 387 | 2 | anonymous | 1 |
| 12/05/2020 05:44 | 12/05/2020 05:48 | 286 | 2 | anonymous | 1 |
| 12/05/2020 05:56 | 12/05/2020 05:58 | 138 | 2 | anonymous | 2 |
| 12/05/2020 06:31 | 12/05/2020 06:32 | 47 | 2 | anonymous | 1 |
| 12/05/2020 06:33 | 12/05/2020 06:34 | 56 | 2 | anonymous | 1 |
| 12/05/2020 06:32 | 12/05/2020 06:35 | 187 | 2 | anonymous | 1 |
| 12/05/2020 06:33 | 12/05/2020 06:35 | 118 | 2 | anonymous | 1 |
| 12/05/2020 06:33 | 12/05/2020 06:36 | 159 | 2 | anonymous | 1 |
| 12/05/2020 06:45 | 12/05/2020 06:45 | 39 | 2 | anonymous | 2 |
| 12/05/2020 06:48 | 12/05/2020 06:49 | 88 | 2 | anonymous | 1 |
| 12/05/2020 06:47 | 12/05/2020 06:49 | 109 | 2 | anonymous | 1 |
| 12/05/2020 06:49 | 12/05/2020 06:51 | 120 | 2 | anonymous | 1 |
| 12/05/2020 06:53 | 12/05/2020 06:54 | 84 | 2 | anonymous | 1 |
| 12/05/2020 06:40 | 12/05/2020 07:05 | 1510 | 2 | anonymous | 1 |
| 12/05/2020 07:05 | 12/05/2020 07:06 | 99 | 2 | anonymous | 2 |
| 12/05/2020 07:02 | 12/05/2020 07:07 | 252 | 2 | anonymous | 1 |
| 12/05/2020 07:02 | 12/05/2020 07:10 | 458 | 2 | anonymous | 1 |
| 12/05/2020 07:04 | 12/05/2020 07:17 | 813 | 2 | anonymous | 2 |
| 12/05/2020 07:30 | 12/05/2020 07:32 | 142 | 2 | anonymous | 1 |
| 12/05/2020 07:34 | 12/05/2020 07:34 | 39 | 2 | anonymous | 1 |
| 12/05/2020 07:44 | 12/05/2020 07:45 | 99 | 2 | anonymous | 2 |
| 12/05/2020 07:53 | 12/05/2020 07:54 | 97 | 2 | anonymous | 1 |
| 12/05/2020 07:54 | 12/05/2020 07:56 | 154 | 2 | anonymous | 1 |
| 12/05/2020 08:07 | 12/05/2020 08:10 | 190 | 2 | anonymous | 2 |
| 12/05/2020 08:12 | 12/05/2020 08:14 | 110 | 2 | anonymous | 2 |
| 12/05/2020 08:31 | 12/05/2020 08:34 | 126 | 2 | anonymous | 1 |
| 12/05/2020 08:28 | 12/05/2020 08:34 | 379 | 2 | anonymous | 1 |

| | | | | | |
|---|---|---|---|---|---|
| 12/05/2020 09:10 | 12/05/2020 09:12 | 77 | 2 | anonymous | 1 |
| 12/05/2020 09:15 | 12/05/2020 09:16 | 64 | 2 | anonymous | 2 |
| 12/05/2020 09:18 | 12/05/2020 09:19 | 76 | 2 | anonymous | 1 |
| 12/05/2020 09:25 | 12/05/2020 09:26 | 68 | 2 | anonymous | 1 |
| 12/05/2020 09:27 | 12/05/2020 09:28 | 88 | 2 | anonymous | 1 |
| 12/05/2020 09:35 | 12/05/2020 09:37 | 141 | 2 | anonymous | 1 |
| 12/05/2020 09:37 | 12/05/2020 09:37 | 37 | 2 | anonymous | 1 |
| 12/05/2020 09:38 | 12/05/2020 09:38 | 29 | 2 | anonymous | 1 |
| 12/05/2020 09:40 | 12/05/2020 09:41 | 35 | 2 | anonymous | 1 |
| 12/05/2020 10:13 | 12/05/2020 10:14 | 77 | 2 | anonymous | 1 |
| 12/05/2020 10:59 | 12/05/2020 11:00 | 42 | 2 | anonymous | 2 |
| 12/05/2020 11:04 | 12/05/2020 11:04 | 47 | 2 | anonymous | 1 |
| 12/05/2020 11:01 | 12/05/2020 11:05 | 213 | 2 | anonymous | 1 |
| 12/05/2020 11:07 | 12/05/2020 11:09 | 99 | 2 | anonymous | 2 |
| 12/05/2020 11:09 | 12/05/2020 11:10 | 46 | 2 | anonymous | 1 |
| 12/05/2020 11:16 | 12/05/2020 11:19 | 192 | 2 | anonymous | 1 |
| 12/05/2020 11:33 | 12/05/2020 11:36 | 180 | 2 | anonymous | 2 |
| 12/05/2020 11:37 | 12/05/2020 11:39 | 84 | 2 | anonymous | 2 |
| 12/05/2020 11:48 | 12/05/2020 11:50 | 92 | 2 | anonymous | 2 |
| 12/05/2020 12:03 | 12/05/2020 12:04 | 77 | 2 | anonymous | 1 |
| 12/05/2020 12:28 | 12/05/2020 12:33 | 265 | 2 | anonymous | 1 |
| 12/05/2020 12:41 | 12/05/2020 12:43 | 74 | 2 | anonymous | 1 |
| 12/05/2020 12:45 | 12/05/2020 12:47 | 73 | 2 | anonymous | 1 |
| 12/05/2020 13:07 | 12/05/2020 13:25 | 1058 | 2 | anonymous | 1 |
| 12/05/2020 13:59 | 12/05/2020 14:00 | 59 | 2 | anonymous | 1 |
| 12/05/2020 13:59 | 12/05/2020 14:01 | 120 | 2 | anonymous | 1 |
| 12/05/2020 14:08 | 12/05/2020 14:10 | 167 | 2 | anonymous | 2 |
| 12/05/2020 14:15 | 12/05/2020 14:18 | 128 | 2 | anonymous | 1 |
| 12/05/2020 14:41 | 12/05/2020 14:46 | 301 | 2 | anonymous | 1 |
| 12/05/2020 14:49 | 12/05/2020 14:52 | 169 | 2 | anonymous | 1 |
| 12/05/2020 15:56 | 12/05/2020 15:58 | 90 | 2 | anonymous | 1 |
| 12/05/2020 17:00 | 12/05/2020 17:03 | 197 | 2 | anonymous | 1 |
| 12/05/2020 17:25 | 12/05/2020 17:25 | 14 | 2 | anonymous | 2 |
| 12/05/2020 17:25 | 12/05/2020 17:26 | 65 | 2 | anonymous | 2 |
| 12/05/2020 17:27 | 12/05/2020 17:27 | 12 | 2 | anonymous | 1 |
| 12/05/2020 18:00 | 12/05/2020 18:07 | 385 | 2 | anonymous | 1 |
| 12/05/2020 18:19 | 12/05/2020 18:21 | 104 | 2 | anonymous | 2 |
| 12/05/2020 18:34 | 12/05/2020 18:35 | 50 | 2 | anonymous | 1 |
| 12/05/2020 19:16 | 12/05/2020 19:19 | 191 | 2 | anonymous | 2 |
| 12/05/2020 19:36 | 12/05/2020 19:37 | 35 | 2 | anonymous | 2 |
| 12/05/2020 19:55 | 12/05/2020 19:58 | 156 | 2 | anonymous | 1 |
| 12/05/2020 20:57 | 12/05/2020 20:59 | 90 | 2 | anonymous | 1 |
| 12/05/2020 21:05 | 12/05/2020 21:08 | 166 | 2 | anonymous | 1 |
| 12/05/2020 22:41 | 12/05/2020 22:42 | 74 | 2 | anonymous | 1 |
| 12/05/2020 22:55 | 12/05/2020 22:56 | 73 | 2 | anonymous | 2 |
| 12/05/2020 23:01 | 12/05/2020 23:04 | 208 | 2 | anonymous | 2 |
| 12/05/2020 23:52 | 12/05/2020 23:54 | 152 | 2 | anonymous | 1 |
| 13/05/2020 00:43 | 13/05/2020 00:45 | 93 | 2 | anonymous | 1 |
| 13/05/2020 00:46 | 13/05/2020 01:02 | 990 | 2 | anonymous | 1 |
| 13/05/2020 01:35 | 13/05/2020 01:39 | 222 | 2 | anonymous | 1 |
| 13/05/2020 01:59 | 13/05/2020 02:03 | 208 | 2 | anonymous | 2 |
| 13/05/2020 02:20 | 13/05/2020 02:22 | 97 | 2 | anonymous | 1 |

| | | | | | |
|---|---|---|---|---|---|
| 13/05/2020 02:56 | 13/05/2020 02:57 | 49 | 2 | anonymous | 1 |
| 13/05/2020 02:56 | 13/05/2020 02:58 | 109 | 2 | anonymous | 1 |
| 13/05/2020 03:16 | 13/05/2020 03:17 | 31 | 2 | anonymous | 1 |
| 13/05/2020 03:33 | 13/05/2020 03:36 | 142 | 2 | anonymous | 1 |
| 13/05/2020 03:47 | 13/05/2020 03:48 | 55 | 2 | anonymous | 2 |
| 13/05/2020 03:57 | 13/05/2020 04:01 | 267 | 2 | anonymous | 1 |
| 13/05/2020 03:59 | 13/05/2020 04:02 | 188 | 2 | anonymous | 1 |
| 13/05/2020 04:14 | 13/05/2020 04:15 | 27 | 2 | anonymous | 1 |
| 13/05/2020 05:27 | 13/05/2020 05:30 | 154 | 2 | anonymous | 1 |
| 13/05/2020 06:36 | 13/05/2020 06:44 | 515 | 2 | anonymous | 2 |
| 13/05/2020 07:21 | 13/05/2020 07:26 | 265 | 2 | anonymous | 1 |
| 13/05/2020 07:53 | 13/05/2020 07:57 | 237 | 2 | anonymous | 2 |
| 13/05/2020 08:50 | 13/05/2020 08:52 | 97 | 2 | anonymous | 2 |
| 13/05/2020 09:44 | 13/05/2020 09:46 | 79 | 2 | anonymous | 1 |
| 13/05/2020 09:49 | 13/05/2020 09:50 | 57 | 2 | anonymous | 2 |
| 13/05/2020 10:58 | 13/05/2020 10:59 | 60 | 2 | anonymous | 1 |
| 13/05/2020 11:22 | 13/05/2020 11:23 | 66 | 2 | anonymous | 1 |
| 13/05/2020 12:06 | 13/05/2020 12:08 | 95 | 2 | anonymous | 1 |
| 13/05/2020 12:38 | 13/05/2020 12:40 | 91 | 2 | anonymous | 1 |
| 13/05/2020 14:16 | 13/05/2020 14:17 | 77 | 2 | anonymous | 1 |
| 13/05/2020 14:36 | 13/05/2020 14:37 | 18 | 2 | anonymous | 2 |
| 13/05/2020 14:37 | 13/05/2020 14:37 | 10 | 2 | anonymous | 2 |
| 13/05/2020 14:46 | 13/05/2020 14:46 | 37 | 2 | anonymous | 2 |
| 13/05/2020 17:20 | 13/05/2020 17:22 | 111 | 2 | anonymous | 2 |
| 13/05/2020 17:24 | 13/05/2020 17:27 | 163 | 2 | anonymous | 2 |
| 13/05/2020 17:40 | 13/05/2020 17:42 | 65 | 2 | anonymous | 2 |
| 13/05/2020 21:00 | 13/05/2020 21:02 | 117 | 2 | anonymous | 1 |
| 13/05/2020 22:40 | 13/05/2020 22:41 | 72 | 2 | anonymous | 1 |
| 13/05/2020 22:49 | 13/05/2020 22:50 | 71 | 2 | anonymous | 1 |
| 14/05/2020 00:31 | 14/05/2020 00:33 | 137 | 2 | anonymous | 1 |
| 14/05/2020 01:23 | 14/05/2020 01:24 | 113 | 2 | anonymous | 1 |
| 14/05/2020 01:46 | 14/05/2020 01:48 | 101 | 2 | anonymous | 1 |
| 14/05/2020 01:54 | 14/05/2020 01:58 | 212 | 2 | anonymous | 1 |
| 14/05/2020 02:59 | 14/05/2020 03:01 | 117 | 2 | anonymous | 1 |
| 14/05/2020 03:56 | 14/05/2020 03:59 | 170 | 2 | anonymous | 1 |
| 14/05/2020 04:24 | 14/05/2020 04:27 | 156 | 2 | anonymous | 1 |
| 14/05/2020 05:50 | 14/05/2020 05:50 | 32 | 2 | anonymous | 2 |
| 14/05/2020 05:49 | 14/05/2020 05:51 | 88 | 2 | anonymous | 1 |
| 14/05/2020 05:51 | 14/05/2020 05:51 | 26 | 2 | anonymous | 3 |
| 14/05/2020 06:49 | 14/05/2020 06:49 | 14 | 2 | anonymous | 2 |
| 14/05/2020 06:49 | 14/05/2020 06:49 | 13 | 2 | anonymous | 2 |
| 14/05/2020 08:55 | 14/05/2020 08:59 | 203 | 2 | anonymous | 2 |
| 14/05/2020 08:59 | 14/05/2020 09:01 | 104 | 2 | anonymous | 2 |
| 14/05/2020 09:06 | 14/05/2020 09:07 | 70 | 2 | anonymous | 2 |
| 14/05/2020 09:07 | 14/05/2020 09:11 | 232 | 2 | anonymous | 2 |
| 14/05/2020 09:11 | 14/05/2020 09:12 | 65 | 2 | anonymous | 2 |
| 14/05/2020 09:13 | 14/05/2020 09:14 | 80 | 2 | anonymous | 2 |
| 14/05/2020 09:14 | 14/05/2020 09:17 | 147 | 2 | anonymous | 2 |
| 14/05/2020 09:17 | 14/05/2020 09:20 | 152 | 2 | anonymous | 2 |
| 14/05/2020 09:20 | 14/05/2020 09:21 | 46 | 2 | anonymous | 2 |
| 14/05/2020 09:21 | 14/05/2020 09:22 | 74 | 2 | anonymous | 3 |
| 14/05/2020 13:49 | 14/05/2020 13:53 | 214 | 2 | anonymous | 1 |

| | | | | | |
|---|---|---|---|---|---|
| 14/05/2020 13:53 | 14/05/2020 13:54 | 58 | 2 | anonymous | 1 |
| 14/05/2020 17:40 | 14/05/2020 17:40 | 16 | 2 | anonymous | 2 |
| 14/05/2020 17:40 | 14/05/2020 17:41 | 14 | 2 | anonymous | 2 |
| 14/05/2020 17:41 | 14/05/2020 17:43 | 136 | 2 | anonymous | 2 |
| 14/05/2020 17:44 | 14/05/2020 17:44 | 11 | 2 | anonymous | 2 |
| 14/05/2020 17:44 | 14/05/2020 17:45 | 69 | 2 | anonymous | 1 |
| 14/05/2020 17:45 | 14/05/2020 17:45 | 12 | 2 | anonymous | 1 |
| 14/05/2020 17:45 | 14/05/2020 17:46 | 12 | 2 | anonymous | 2 |
| 14/05/2020 17:46 | 14/05/2020 17:46 | 11 | 2 | anonymous | 2 |
| 15/05/2020 03:45 | 15/05/2020 03:49 | 249 | 2 | anonymous | 2 |
| 15/05/2020 03:45 | 15/05/2020 03:49 | 254 | 2 | anonymous | 1 |
| 15/05/2020 06:29 | 15/05/2020 06:30 | 65 | 2 | anonymous | 1 |
| 15/05/2020 10:51 | 15/05/2020 10:53 | 115 | 2 | anonymous | 1 |
| 15/05/2020 15:23 | 15/05/2020 15:26 | 168 | 2 | anonymous | 2 |
| 15/05/2020 19:40 | 15/05/2020 19:41 | 59 | 2 | anonymous | 1 |
| 15/05/2020 19:42 | 15/05/2020 19:43 | 62 | 2 | anonymous | 1 |
| 16/05/2020 01:45 | 16/05/2020 01:49 | 234 | 2 | anonymous | 1 |
| 16/05/2020 15:42 | 16/05/2020 15:43 | 50 | 2 | anonymous | 1 |
| 16/05/2020 21:08 | 16/05/2020 21:10 | 83 | 2 | anonymous | 2 |
| 16/05/2020 21:56 | 16/05/2020 21:56 | 14 | 2 | anonymous | 1 |
| 16/05/2020 21:56 | 16/05/2020 21:58 | 87 | 2 | anonymous | 2 |
| 17/05/2020 01:45 | 17/05/2020 01:47 | 90 | 2 | anonymous | 1 |
| 17/05/2020 03:01 | 17/05/2020 03:04 | 188 | 2 | anonymous | 1 |
| 17/05/2020 15:38 | 17/05/2020 15:40 | 125 | 2 | anonymous | 1 |
| 17/05/2020 21:32 | 17/05/2020 21:33 | 50 | 2 | anonymous | 2 |
| 17/05/2020 21:33 | 17/05/2020 21:33 | 9 | 2 | anonymous | 1 |
| 17/05/2020 21:33 | 17/05/2020 21:33 | 11 | 2 | anonymous | 2 |
| 17/05/2020 21:33 | 17/05/2020 21:34 | 17 | 2 | anonymous | 2 |
| 17/05/2020 21:34 | 17/05/2020 21:35 | 78 | 2 | anonymous | 1 |
| 18/05/2020 04:40 | 18/05/2020 04:41 | 66 | 2 | anonymous | 2 |
| 18/05/2020 08:33 | 18/05/2020 08:34 | 51 | 2 | anonymous | 2 |
| 18/05/2020 11:17 | 18/05/2020 11:17 | 20 | 2 | anonymous | 1 |
| 18/05/2020 11:17 | 18/05/2020 11:17 | 13 | 2 | anonymous | 2 |
| 18/05/2020 11:18 | 18/05/2020 11:19 | 88 | 2 | anonymous | 1 |
| 19/05/2020 06:10 | 19/05/2020 06:12 | 90 | 2 | anonymous | 2 |
| 19/05/2020 06:39 | 19/05/2020 06:40 | 79 | 2 | anonymous | 2 |
| 19/05/2020 06:40 | 19/05/2020 06:41 | 34 | 2 | anonymous | 2 |
| 19/05/2020 06:41 | 19/05/2020 06:41 | 9 | 2 | anonymous | 1 |
| 19/05/2020 06:41 | 19/05/2020 06:41 | 10 | 2 | anonymous | 1 |
| 19/05/2020 06:41 | 19/05/2020 06:41 | 9 | 2 | anonymous | 1 |
| 19/05/2020 06:41 | 19/05/2020 06:42 | 12 | 2 | anonymous | 1 |
| 19/05/2020 06:42 | 19/05/2020 06:42 | 9 | 2 | anonymous | 1 |
| 19/05/2020 06:42 | 19/05/2020 06:43 | 11 | 2 | anonymous | 1 |
| 19/05/2020 06:46 | 19/05/2020 06:46 | 8 | 2 | anonymous | 1 |
| 19/05/2020 06:46 | 19/05/2020 06:47 | 79 | 2 | anonymous | 2 |
| 19/05/2020 06:47 | 19/05/2020 06:47 | 8 | 2 | anonymous | 1 |
| 19/05/2020 06:47 | 19/05/2020 06:48 | 8 | 2 | anonymous | 1 |
| 19/05/2020 06:48 | 19/05/2020 06:48 | 9 | 2 | anonymous | 1 |
| 19/05/2020 06:48 | 19/05/2020 06:49 | 57 | 2 | anonymous | 2 |
| 19/05/2020 06:49 | 19/05/2020 06:50 | 61 | 2 | anonymous | 1 |
| 19/05/2020 06:50 | 19/05/2020 06:50 | 8 | 2 | anonymous | 1 |
| 19/05/2020 06:50 | 19/05/2020 06:50 | 8 | 2 | anonymous | 2 |

| | | | | | |
|---|---|---|---|---|---|
| 19/05/2020 06:51 | 19/05/2020 06:51 | 7 | 2 | anonymous | 2 |
| 19/05/2020 06:51 | 19/05/2020 06:51 | 8 | 2 | anonymous | 1 |
| 19/05/2020 06:51 | 19/05/2020 06:51 | 8 | 2 | anonymous | 1 |
| 19/05/2020 06:51 | 19/05/2020 06:51 | 8 | 2 | anonymous | 2 |
| 19/05/2020 06:52 | 19/05/2020 06:52 | 13 | 2 | anonymous | 2 |
| 19/05/2020 06:52 | 19/05/2020 06:52 | 7 | 2 | anonymous | 2 |
| 19/05/2020 06:52 | 19/05/2020 06:52 | 9 | 2 | anonymous | 2 |
| 19/05/2020 06:53 | 19/05/2020 06:53 | 10 | 2 | anonymous | 2 |
| 19/05/2020 06:53 | 19/05/2020 06:53 | 10 | 2 | anonymous | 2 |
| 19/05/2020 06:53 | 19/05/2020 06:53 | 8 | 2 | anonymous | 2 |
| 19/05/2020 06:53 | 19/05/2020 06:54 | 8 | 2 | anonymous | 2 |
| 19/05/2020 06:54 | 19/05/2020 06:54 | 7 | 2 | anonymous | 2 |
| 19/05/2020 06:54 | 19/05/2020 06:54 | 9 | 2 | anonymous | 2 |
| 19/05/2020 06:54 | 19/05/2020 06:54 | 10 | 2 | anonymous | 2 |
| 19/05/2020 06:54 | 19/05/2020 06:54 | 8 | 2 | anonymous | 2 |
| 19/05/2020 06:54 | 19/05/2020 06:55 | 9 | 2 | anonymous | 2 |
| 21/05/2020 08:49 | 21/05/2020 08:50 | 59 | 2 | anonymous | 1 |
| 21/05/2020 19:13 | 21/05/2020 19:13 | 40 | 2 | anonymous | 2 |
| 23/05/2020 05:43 | 23/05/2020 05:43 | 30 | 2 | anonymous | 1 |
| 27/05/2020 04:27 | 27/05/2020 04:32 | 302 | 2 | anonymous | 1 |
| 01/06/2020 16:10 | 01/06/2020 16:11 | 52 | 2 | anonymous | 1 |
| 01/06/2020 20:41 | 01/06/2020 20:42 | 58 | 2 | anonymous | 1 |
| 03/06/2020 15:59 | 03/06/2020 16:00 | 62 | 2 | anonymous | 1 |
| 09/06/2020 16:45 | 09/06/2020 16:46 | 81 | 2 | anonymous | 1 |
| 10/06/2020 17:08 | 10/06/2020 17:13 | 276 | 2 | anonymous | 2 |
| 10/06/2020 17:13 | 10/06/2020 17:15 | 99 | 2 | anonymous | 1 |
| 25/06/2020 00:53 | 25/06/2020 00:55 | 102 | 2 | anonymous | 1 |
| 15/09/2020 06:08 | 15/09/2020 06:10 | 122 | 2 | anonymous | 1 |

| | | | | | |
|---|---|---|---|---|---|
| 15/09/2020 06:11 | 15/09/2020 06:15 | 251 | 2 | anonymous | 2 |
| 15/09/2020 06:16 | 15/09/2020 06:17 | 100 | 2 | anonymous | 1 |
| 15/09/2020 06:18 | 15/09/2020 06:21 | 195 | 2 | anonymous | 1 |
| 16/09/2020 15:17 | 16/09/2020 15:19 | 111 | 2 | anonymous | 1 |
| 23/09/2020 08:07 | 23/09/2020 08:08 | 76 | 2 | anonymous | 1 |
| 23/09/2020 08:08 | 23/09/2020 08:09 | 95 | 2 | anonymous | 2 |
| 23/09/2020 08:09 | 23/09/2020 08:15 | 362 | 2 | anonymous | 2 |
| 23/09/2020 08:12 | 23/09/2020 08:15 | 211 | 2 | anonymous | 2 |
| 23/09/2020 08:17 | 23/09/2020 08:19 | 118 | 2 | anonymous | 2 |
| 23/09/2020 14:10 | 23/09/2020 14:18 | 513 | 2 | anonymous | 2 |
| 25/09/2020 04:17 | 25/09/2020 04:19 | 84 | 2 | anonymous | 2 |
| 25/09/2020 04:19 | 25/09/2020 04:22 | 180 | 2 | anonymous | 1 |
| 28/09/2020 04:34 | 28/09/2020 04:39 | 329 | 2 | anonymous | 2 |

| | | | | | |
|---|---|---|---|---|---|
| 29/09/2020 17:32 | 29/09/2020 17:37 | 274 | 2 | anonymous | 1 |
| 30/09/2020 05:02 | 30/09/2020 05:04 | 139 | 2 | anonymous | 1 |
| 30/09/2020 05:04 | 30/09/2020 05:06 | 84 | 2 | anonymous | 2 |
| 30/09/2020 05:07 | 30/09/2020 05:09 | 113 | 2 | anonymous | 1 |
| 30/09/2020 05:11 | 30/09/2020 05:14 | 181 | 2 | anonymous | 1 |
| 30/09/2020 05:18 | 30/09/2020 05:22 | 260 | 2 | anonymous | 2 |
| 30/09/2020 05:37 | 30/09/2020 05:39 | 144 | 2 | anonymous | 2 |
| 30/09/2020 05:40 | 30/09/2020 05:45 | 251 | 2 | anonymous | 2 |
| 04/10/2020 13:48 | 04/10/2020 13:51 | 198 | 2 | anonymous | 2 |
| 04/10/2020 13:52 | 04/10/2020 13:57 | 264 | 2 | anonymous | 1 |
| 09/10/2020 06:39 | 09/10/2020 13:09 | 23360 | 1 | preview | 2 |
| 20/10/2020 02:38 | 20/10/2020 02:41 | 155 | 1 | preview | 1 |
| 05/11/2020 04:29 | 05/11/2020 04:34 | 301 | 2 | anonymous | 2 |

| | | | | | |
|---|---|---|---|---|---|
| 05/11/2020 04:35 | 05/11/2020 04:40 | 276 | 2 | anonymous | 2 |
| 05/11/2020 04:41 | 05/11/2020 04:46 | 264 | 2 | anonymous | 1 |

code: 1 preview
2 anonymous
3 social

1 female
2 male
3 not specified

| Q1 Gender | age | Q2 Age | Q3 .......... | usage | drugUsed | guarana | B12 | B6 |
|---|---|---|---|---|---|---|---|---|
| Female | 1 | 18 to 25 | .......... | 2 | | 0 | 0 | 0 |
| Female | 1 | 18 to 25 | .......... | 2 | | 0 | 0 | 0 |
| Female | 1 | 18 to 25 | Yes | 1 | 8,9 | 0 | 1 | 1 |
| Female | 1 | 18 to 25 | .......... | 2 | | 0 | 0 | 0 |
| Female | 1 | 18 to 25 | .......... | 2 | | 0 | 0 | 0 |
| Female | 1 | 18 to 25 | .......... | 2 | | 0 | 0 | 0 |
| Female | 1 | 18 to 25 | .......... | 2 | | 0 | 0 | 0 |
| Male | 1 | 18 to 25 | .......... | 2 | | 0 | 0 | 0 |
| Female | 1 | 18 to 25 | .......... | 2 | | 0 | 0 | 0 |
| Female | 1 | 18 to 25 | .......... | 2 | | 0 | 0 | 0 |
| Female | 1 | 18 to 25 | .......... | 2 | | 0 | 0 | 0 |
| Male | 1 | 18 to 25 | .......... | 2 | | 0 | 0 | 0 |
| Female | 1 | 18 to 25 | .......... | 2 | | 0 | 0 | 0 |
| Female | 1 | 18 to 25 | .......... | 2 | | 0 | 0 | 0 |
| Female | 1 | 18 to 25 | .......... | 2 | | 0 | 0 | 0 |
| Female | 1 | 18 to 25 | .......... | 2 | | 0 | 0 | 0 |
| Female | 1 | 18 to 25 | .......... | 2 | | 0 | 0 | 0 |
| Female | 1 | 18 to 25 | .......... | 2 | | 0 | 0 | 0 |
| Male | 1 | 18 to 25 | .......... | 2 | | 0 | 0 | 0 |
| Female | 1 | 18 to 25 | Yes | 1 | 9 | 0 | 1 | 0 |
| Female | 3 | 36 to 45 | .......... | 2 | | 0 | 0 | 0 |
| Female | 1 | 18 to 25 | .......... | 2 | | 0 | 0 | 0 |
| Female | 1 | 18 to 25 | .......... | 2 | | 0 | 0 | 0 |
| Female | 1 | 18 to 25 | .......... | 2 | | 0 | 0 | 0 |
| Female | 1 | 18 to 25 | .......... | 2 | | 0 | 0 | 0 |
| Female | 1 | 18 to 25 | .......... | 2 | | 0 | 0 | 0 |
| Male | 1 | 18 to 25 | Yes | 1 | 5 | 0 | 0 | 0 |
| Female | 1 | 18 to 25 | .......... | 2 | | 0 | 0 | 0 |
| Female | 1 | 18 to 25 | .......... | 2 | | 0 | 0 | 0 |
| Prefer not say | 1 | 18 to 25 | .......... | 2 | | 0 | 0 | 0 |
| Male | 1 | 18 to 25 | Yes | 1 | 5,8,9,11 | 0 | 1 | 1 |
| Female | 1 | 18 to 25 | .......... | 2 | | 0 | 0 | 0 |
| Male | 1 | 18 to 25 | .......... | 2 | | 0 | 0 | 0 |
| Male | 1 | 18 to 25 | .......... | 2 | | 0 | 0 | 0 |
| Male | 1 | 18 to 25 | Do not know/unsure | 3 | 9 | 0 | 1 | 0 |
| Female | 1 | 18 to 25 | .......... | 2 | | 0 | 0 | 0 |
| Male | 1 | 18 to 25 | .......... | 2 | | 0 | 0 | 0 |
| Female | 1 | 18 to 25 | .......... | 2 | | 0 | 0 | 0 |
| Female | 1 | 18 to 25 | .......... | 2 | | 0 | 0 | 0 |
| Male | 1 | 18 to 25 | .......... | 2 | | 0 | 0 | 0 |
| Female | 1 | 18 to 25 | .......... | 2 | | 0 | 0 | 0 |
| Female | 1 | 18 to 25 | .......... | 2 | | 0 | 0 | 0 |
| Female | 1 | 18 to 25 | .......... | 2 | | 0 | 0 | 0 |
| Male | 1 | 18 to 25 | .......... | 2 | | 0 | 0 | 0 |
| Female | 1 | 18 to 25 | Do not know/unsure | 3 | 5 | 0 | 0 | 0 |
| Female | 1 | 18 to 25 | .......... | 2 | | 0 | 0 | 0 |
| Female | 1 | 18 to 25 | .......... | 2 | | 0 | 0 | 0 |
| Female | 1 | 18 to 25 | Yes | 1 | 5,8,9 | 0 | 1 | 1 |
| Female | 3 | 46 to 55 | .......... | 2 | | 0 | 0 | 0 |
| Female | 1 | 18 to 25 | Yes | 1 | 5 | 0 | 0 | 0 |

| | | | | | | | | | |
|---|---|---|---|---|---|---|---|---|---|
| Male | 1 | 18 to 25 | [illegible] | 2 | | | 0 | 0 | 0 |
| Female | 1 | 18 to 25 | [illegible] | 2 | | | 0 | 0 | 0 |
| Female | 1 | 18 to 25 | Yes | 1 | | | 0 | 0 | 0 |
| Female | 1 | 18 to 25 | [illegible] | 2 | | | 0 | 0 | 0 |
| Female | 1 | 18 to 25 | Do not know/unsure | 3 | 8,9 | | 0 | 1 | 1 |
| Female | 1 | 18 to 25 | [illegible] | 2 | | | 0 | 0 | 0 |
| Female | 1 | 18 to 25 | [illegible] | 2 | | | 0 | 0 | 0 |
| Female | 1 | 18 to 25 | [illegible] | 2 | | 11 | 0 | 0 | 0 |
| Female | 1 | 18 to 25 | [illegible] | 2 | | | 0 | 0 | 0 |
| Female | 1 | 18 to 25 | Yes | 1 | | 4 | 0 | 0 | 0 |
| Male | 1 | 18 to 25 | Yes | 1 | 1,9 | | 0 | 1 | 0 |
| Male | 1 | 18 to 25 | [illegible] | 2 | | | 0 | 0 | 0 |
| Male | 1 | 18 to 25 | [illegible] | 2 | | | 0 | 0 | 0 |
| Male | 1 | 18 to 25 | [illegible] | 2 | | | 0 | 0 | 0 |
| Female | 1 | 18 to 25 | Yes | 1 | | 5 | 0 | 0 | 0 |
| Male | 1 | 18 to 25 | Yes | 1 | | 2 | 0 | 0 | 0 |
| Male | 1 | 18 to 25 | [illegible] | 2 | | | 0 | 0 | 0 |
| Female | 1 | 18 to 25 | [illegible] | 2 | | | 0 | 0 | 0 |
| Female | 1 | 18 to 25 | [illegible] | 2 | | | 0 | 0 | 0 |
| Male | 1 | 18 to 25 | [illegible] | 2 | | | 0 | 0 | 0 |
| Female | 1 | 18 to 25 | [illegible] | 2 | | | 0 | 0 | 0 |
| Female | 1 | 18 to 25 | [illegible] | 2 | | | 0 | 0 | 0 |
| Female | 1 | 18 to 25 | [illegible] | 2 | | | 0 | 0 | 0 |
| Male | 1 | 18 to 25 | [illegible] | 2 | | | 0 | 0 | 0 |
| Female | 1 | 18 to 25 | [illegible] | 2 | | | 0 | 0 | 0 |
| Female | 1 | 18 to 25 | [illegible] | 2 | | | 0 | 0 | 0 |
| Female | 1 | 18 to 25 | [illegible] | 2 | | | 0 | 0 | 0 |
| Male | 1 | 18 to 25 | Yes | 1 | 2,9 | | 0 | 1 | 0 |
| Male | 1 | 18 to 25 | Yes | 1 | | 3 | 0 | 0 | 0 |
| Male | 1 | 18 to 25 | Yes | 1 | | 5 | 0 | 0 | 0 |
| Male | 1 | 18 to 25 | Yes | 1 | | 5 | 0 | 0 | 0 |
| Female | 1 | 18 to 25 | [illegible] | 2 | | | 0 | 0 | 0 |
| Female | 1 | 18 to 25 | Yes | 1 | 5,9 | | 0 | 1 | 0 |
| Male | 1 | 18 to 25 | Yes | 1 | | 2 | 0 | 0 | 0 |
| Male | 1 | 18 to 25 | Yes | 1 | 2,5 | | 0 | 0 | 0 |
| Male | 1 | 18 to 25 | Yes | 1 | 2,5,9 | | 0 | 1 | 0 |
| Male | 1 | 18 to 25 | Yes | 1 | 2,9 | | 0 | 1 | 0 |
| Male | 1 | 18 to 25 | Yes | 1 | | 9 | 0 | 1 | 0 |
| Male | 1 | 18 to 25 | Yes | 1 | | 2 | 0 | 0 | 0 |
| Male | 1 | 18 to 25 | Yes | 1 | 2,9 | | 0 | 1 | 0 |
| Male | 1 | 18 to 25 | Yes | 1 | 2,9 | | 0 | 1 | 0 |
| Female | 1 | 18 to 25 | Yes | 1 | | 5 | 0 | 0 | 0 |
| Female | 1 | 18 to 25 | [illegible] | 2 | | | 0 | 0 | 0 |
| Male | 1 | 18 to 25 | Yes | 1 | 2,5 | | 0 | 0 | 0 |
| Male | 1 | 18 to 25 | Yes | 1 | 2,5,6 | | 1 | 0 | 0 |
| Male | 1 | 18 to 25 | Yes | 1 | 5,9 | | 0 | 1 | 0 |
| Male | 1 | 18 to 25 | Yes | 1 | 2,5,9 | | 0 | 1 | 0 |
| Male | 1 | 18 to 25 | Yes | 1 | | 5 | 0 | 0 | 0 |
| Female | 1 | 18 to 25 | [illegible] | 2 | | | 0 | 0 | 0 |
| Male | 1 | 18 to 25 | Yes | 1 | 2,9 | | 0 | 1 | 0 |
| Male | 1 | 18 to 25 | Yes | 1 | | 2 | 0 | 0 | 0 |
| Male | 1 | 18 to 25 | Yes | 1 | | 3 | 0 | 0 | 0 |

| | | | | | | | | | |
|---|---|---|---|---|---|---|---|---|---|
| Male | 1 | 18 to 25 | ............ | 2 | | | 0 | 0 | 0 |
| Male | 1 | 18 to 25 | ............ | 2 | | | 0 | 0 | 0 |
| Male | 1 | 18 to 25 | Yes | 1 | 2,5 | | 0 | 0 | 0 |
| Male | 1 | 18 to 25 | Yes | 1 | | 3 | 0 | 0 | 0 |
| Male | 1 | 18 to 25 | ............ | 2 | | | 0 | 0 | 0 |
| Male | 1 | 18 to 25 | Yes | 1 | 3,4 | | 0 | 0 | 0 |
| Male | 1 | 18 to 25 | Yes | 1 | 2,9 | | 0 | 1 | 0 |
| Male | 1 | 18 to 25 | Yes | 1 | 2,9 | | 0 | 1 | 0 |
| Male | 1 | 18 to 25 | Yes | 1 | 2,5 | | 0 | 0 | 0 |
| Male | 1 | 18 to 25 | Yes | 1 | 3,,4 | | 0 | 0 | 0 |
| Female | 1 | 18 to 25 | ............ | 2 | | | 0 | 0 | 0 |
| Female | 1 | 18 to 25 | ............ | 2 | | | 0 | 0 | 0 |
| Female | 1 | 18 to 25 | Yes | 1 | 2,5 | | 0 | 0 | 0 |
| Male | 1 | 18 to 25 | ............ | 2 | | | 0 | 0 | 0 |
| Female | 1 | 18 to 25 | ............ | 2 | | | 0 | 0 | 0 |
| Male | 1 | 18 to 25 | Yes | 1 | 3,4 | | 0 | 0 | 0 |
| Male | 1 | 18 to 25 | ............ | 2 | | | 0 | 0 | 0 |
| Male | 1 | 18 to 25 | ............ | 2 | | | 0 | 0 | 0 |
| Female | 1 | 18 to 25 | ............ | 2 | | | 0 | 0 | 0 |
| Female | 1 | 18 to 25 | ............ | 2 | | | 0 | 0 | 0 |
| Male | 1 | 18 to 25 | Yes | 1 | 2,9 | | 0 | 1 | 0 |
| Male | 1 | 18 to 25 | ............ | 2 | | | 0 | 0 | 0 |
| Male | 1 | 18 to 25 | Yes | 1 | | 1 | 0 | 0 | 0 |
| Male | 1 | 18 to 25 | ............ | 2 | | | 0 | 0 | 0 |
| | 3 | | | 2 | | | 0 | 0 | 0 |
| | 3 | | | 2 | | | 0 | 0 | 0 |
| Male | 3 | | Yes | 1 | | 2 | 0 | 0 | 0 |
| Male | 1 | 18 to 25 | Yes | 1 | 2,5 | | 0 | 0 | 0 |
| Female | 1 | 18 to 25 | ............ | 2 | | | 0 | 0 | 0 |
| Male | 1 | 18 to 25 | ............ | 2 | | | 0 | 0 | 0 |
| Female | 1 | 18 to 25 | ............ | 2 | | | 0 | 0 | 0 |
| Male | 1 | 18 to 25 | ............ | 2 | | | 0 | 0 | 0 |
| Female | 1 | 18 to 25 | ............ | 2 | | | 0 | 0 | 0 |
| Male | 1 | 18 to 25 | Yes | 1 | | 4 | 0 | 0 | 0 |
| Male | 1 | 18 to 25 | ............ | 2 | | | 0 | 0 | 0 |
| Male | 1 | 18 to 25 | ............ | 2 | | | 0 | 0 | 0 |
| Male | 1 | 18 to 25 | Yes | 1 | | 2 | 0 | 0 | 0 |
| Male | 1 | 18 to 25 | Yes | 1 | | 2 | 0 | 0 | 0 |
| Female | 1 | 18 to 25 | ............ | 2 | | | 0 | 0 | 0 |
| Female | 1 | 18 to 25 | ............ | 2 | | | 0 | 0 | 0 |
| Female | 1 | 18 to 25 | Yes | 1 | 6,7 | | 1 | 0 | 0 |
| Male | 1 | 18 to 25 | Yes | 1 | | 3 | 0 | 0 | 0 |
| Male | 1 | 18 to 25 | Yes | 1 | | 2 | 0 | 0 | 0 |
| Female | 1 | 18 to 25 | ............ | 2 | | | 0 | 0 | 0 |
| Female | 2 | 26 to 35 | ............ | 2 | | | 0 | 0 | 0 |
| Female | 1 | 18 to 25 | ............ | 2 | | | 0 | 0 | 0 |
| Female | 1 | 18 to 25 | ............ | 2 | | | 0 | 0 | 0 |
| Male | 2 | 26 to 35 | ............ | 2 | | | 0 | 0 | 0 |
| Male | 1 | 18 to 25 | ............ | 2 | | | 0 | 0 | 0 |
| Female | 1 | 18 to 25 | ............ | 2 | | | 0 | 0 | 0 |
| Male | 1 | 18 to 25 | ............ | 2 | | | 0 | 0 | 0 |
| Male | 1 | 18 to 25 | Yes | 1 | | 2 | 0 | 0 | 0 |

| | | | | | | | | | |
|---|---|---|---|---|---|---|---|---|---|
| Male | 1 | 18 to 25 | Yes | 1 | 2,5 | | 0 | 0 | 0 |
| Female | 1 | 18 to 25 | [illegible] | 2 | | | 0 | 0 | 0 |
| Male | 1 | 18 to 25 | [illegible] | 2 | | | 0 | 0 | 0 |
| Female | 1 | 18 to 25 | [illegible] | 2 | | | 0 | 0 | 0 |
| Male | 1 | 18 to 25 | Yes | 1 | | 2 | 0 | 0 | 0 |
| Male | 1 | 18 to 25 | Yes | 1 | | 2 | 0 | 0 | 0 |
| Male | 1 | 18 to 25 | [illegible] | 2 | | | 0 | 0 | 0 |
| Female | 1 | 18 to 25 | [illegible] | 2 | | | 0 | 0 | 0 |
| Male | 1 | 18 to 25 | [illegible] | 2 | | | 0 | 0 | 0 |
| Male | 1 | 18 to 25 | [illegible] | 2 | | | 0 | 0 | 0 |
| Female | 1 | 18 to 25 | [illegible] | 2 | | | 0 | 0 | 0 |
| Male | 1 | 18 to 25 | Yes | 1 | 2,4 | | 0 | 0 | 0 |
| Female | 1 | 18 to 25 | [illegible] | 2 | | | 0 | 0 | 0 |
| Male | 1 | 18 to 25 | [illegible] | 2 | | | 0 | 0 | 0 |
| Female | 1 | 18 to 25 | [illegible] | 2 | | | 0 | 0 | 0 |
| Male | 1 | 18 to 25 | Yes | 1 | 2,4 | | 0 | 0 | 0 |
| Female | 1 | 18 to 25 | [illegible] | 2 | | | 0 | 0 | 0 |
| Male | 1 | 18 to 25 | [illegible] | 2 | | | 0 | 0 | 0 |
| Female | 1 | 18 to 25 | [illegible] | 2 | | | 0 | 0 | 0 |
| Male | 1 | 18 to 25 | [illegible] | 2 | | | 0 | 0 | 0 |
| Female | 1 | 18 to 25 | [illegible] | 2 | | | 0 | 0 | 0 |
| Female | 3 | | | 2 | | | 0 | 0 | 0 |
| Female | 2 | 26 to 35 | [illegible] | 2 | | | 0 | 0 | 0 |
| Male | 3 | 46 to 55 | Yes | 1 | | 2 | 0 | 0 | 0 |
| Male | 1 | 18 to 25 | [illegible] | 2 | | | 0 | 0 | 0 |
| Male | 1 | 18 to 25 | [illegible] | 2 | | | 0 | 0 | 0 |
| Male | 1 | 18 to 25 | [illegible] | 2 | | | 0 | 0 | 0 |
| Female | 1 | 18 to 25 | [illegible] | 2 | | | 0 | 0 | 0 |
| Female | 1 | 18 to 25 | [illegible] | 2 | | | 0 | 0 | 0 |
| Male | 1 | 18 to 25 | [illegible] | 2 | | | 0 | 0 | 0 |
| Female | 1 | 18 to 25 | [illegible] | 2 | | | 0 | 0 | 0 |
| Female | 1 | 18 to 25 | Yes | 1 | | 5 | 0 | 0 | 0 |
| Male | 1 | 18 to 25 | [illegible] | 2 | | | 0 | 0 | 0 |
| Male | 1 | 18 to 25 | Yes | 1 | 2,5 | | 0 | 0 | 0 |
| Male | 1 | 18 to 25 | [illegible] | 2 | | | 0 | 0 | 0 |
| Female | 1 | 18 to 25 | Yes | 1 | | 5 | 0 | 0 | 0 |
| Female | 1 | 18 to 25 | [illegible] | 2 | | | 0 | 0 | 0 |
| Male | 1 | 18 to 25 | Yes | 1 | 2,6 | | 1 | 0 | 0 |
| Male | 1 | 18 to 25 | [illegible] | 2 | | | 0 | 0 | 0 |
| Male | 1 | 18 to 25 | Yes | 1 | 2,5 | | 0 | 0 | 0 |
| Female | 1 | 18 to 25 | [illegible] | 2 | | | 0 | 0 | 0 |
| Female | 1 | 18 to 25 | [illegible] | 2 | | | 0 | 0 | 0 |
| Male | 1 | 18 to 25 | [illegible] | 2 | | | 0 | 0 | 0 |
| Female | 1 | 18 to 25 | [illegible] | 2 | | | 0 | 0 | 0 |
| Female | 1 | 18 to 25 | [illegible] | 2 | | | 0 | 0 | 0 |
| Female | 1 | 18 to 25 | [illegible] | 2 | | | 0 | 0 | 0 |
| Female | 1 | 18 to 25 | [illegible] | 2 | | | 0 | 0 | 0 |
| Female | 1 | 18 to 25 | [illegible] | 2 | | | 0 | 0 | 0 |
| Male | 1 | 18 to 25 | [illegible] | 2 | | | 0 | 0 | 0 |
| Female | 1 | 18 to 25 | [illegible] | 2 | | | 0 | 0 | 0 |
| Female | 1 | 18 to 25 | [illegible] | 2 | | | 0 | 0 | 0 |
| Male | 1 | 18 to 25 | [illegible] | 2 | | | 0 | 0 | 0 |

| | | | | | | | | | |
|---|---|---|---|---|---|---|---|---|---|
| Female | 1 | 18 to 25 | ................ | 2 | | | 0 | 0 | 0 |
| Female | 1 | 18 to 25 | ................ | 2 | | | 0 | 0 | 0 |
| Female | 1 | 18 to 25 | ................ | 2 | | | 0 | 0 | 0 |
| Female | 1 | 18 to 25 | ................ | 2 | | | 0 | 0 | 0 |
| Female | 1 | 18 to 25 | ................ | 2 | | | 0 | 0 | 0 |
| Male | 1 | 18 to 25 | ................ | 2 | | | 0 | 0 | 0 |
| Male | 1 | 18 to 25 | Yes | 1 | | 11 | 0 | 0 | 0 |
| Female | 1 | 18 to 25 | ................ | 2 | | | 0 | 0 | 0 |
| Female | 1 | 18 to 25 | ................ | 2 | | | 0 | 0 | 0 |
| Male | 1 | 18 to 25 | ................ | 2 | | | 0 | 0 | 0 |
| Female | 1 | 18 to 25 | ................ | 2 | | | 0 | 0 | 0 |
| Male | 1 | 18 to 25 | Do not know/unsure | 3 | | 11 | 0 | 0 | 0 |
| Female | 1 | 18 to 25 | Yes | 1 | | 9 | 0 | 1 | 0 |
| Female | 1 | 18 to 25 | ................ | 2 | | | 0 | 0 | 0 |
| Female | 1 | 18 to 25 | ................ | 2 | | | 0 | 0 | 0 |
| Female | 1 | 18 to 25 | ................ | 2 | | | 0 | 0 | 0 |
| Female | 1 | 18 to 25 | ................ | 2 | | | 0 | 0 | 0 |
| Male | 3 | 46 to 55 | ................ | 2 | | | 0 | 0 | 0 |
| Female | 1 | 18 to 25 | ................ | 2 | | | 0 | 0 | 0 |
| Male | 1 | 18 to 25 | ................ | 2 | | | 0 | 0 | 0 |
| Female | 1 | 18 to 25 | ................ | 2 | | | 0 | 0 | 0 |
| Female | 2 | 26 to 35 | Yes | 1 | 4,8,9 | | 0 | 1 | 1 |
| Female | 1 | 18 to 25 | ................ | 2 | | | 0 | 0 | 0 |
| Female | 1 | 18 to 25 | ................ | 2 | | | 0 | 0 | 0 |
| Female | 1 | 18 to 25 | ................ | 2 | | | 0 | 0 | 0 |
| Prefer not say | 1 | 18 to 25 | ................ | 2 | | | 0 | 0 | 0 |
| Female | 1 | 18 to 25 | ................ | 2 | | | 0 | 0 | 0 |
| Female | 2 | 26 to 35 | ................ | 2 | | | 0 | 0 | 0 |
| Female | 2 | 26 to 35 | ................ | 2 | | | 0 | 0 | 0 |
| Female | 1 | 18 to 25 | ................ | 2 | | | 0 | 0 | 0 |
| Female | 1 | 18 to 25 | Yes | 1 | | 9 | 0 | 1 | 0 |
| Female | 1 | 18 to 25 | ................ | 2 | | | 0 | 0 | 0 |
| Female | 2 | 26 to 35 | Yes | 1 | | 4 | 0 | 0 | 0 |
| Female | 1 | 18 to 25 | ................ | 2 | | | 0 | 0 | 0 |
| Female | 1 | 18 to 25 | Yes | 1 | | 9 | 0 | 1 | 0 |
| Female | 1 | 18 to 25 | ................ | 2 | | | 0 | 0 | 0 |
| Female | 1 | 18 to 25 | ................ | 2 | | | 0 | 0 | 0 |
| Female | 1 | 18 to 25 | ................ | 2 | | | 0 | 0 | 0 |
| Female | 1 | 18 to 25 | ................ | 2 | | | 0 | 0 | 0 |
| Female | 1 | 18 to 25 | ................ | 2 | | | 0 | 0 | 0 |
| Female | 1 | 18 to 25 | ................ | 2 | | | 0 | 0 | 0 |
| Male | 1 | 18 to 25 | ................ | 2 | | | 0 | 0 | 0 |
| Female | 1 | 18 to 25 | ................ | 2 | | | 0 | 0 | 0 |
| Female | 1 | 18 to 25 | ................ | 2 | | | 0 | 0 | 0 |
| Female | 1 | 18 to 25 | ................ | 2 | | | 0 | 0 | 0 |
| Female | 2 | 26 to 35 | ................ | 2 | | | 0 | 0 | 0 |
| Female | 1 | 18 to 25 | ................ | 2 | | | 0 | 0 | 0 |
| Female | 1 | 18 to 25 | ................ | 2 | | | 0 | 0 | 0 |
| Female | 1 | 18 to 25 | ................ | 2 | | | 0 | 0 | 0 |
| Female | 1 | 18 to 25 | Do not know/unsure | 3 | 8,9 | | 0 | 1 | 1 |
| Female | 2 | 26 to 35 | ................ | 2 | | | 0 | 0 | 0 |
| Male | 1 | 18 to 25 | ................ | 2 | | | 0 | 0 | 0 |

| | | | | | | | | | |
|---|---|---|---|---|---|---|---|---|---|
| Female | 1 | 18 to 25 | ............... | 2 | | | 0 | 0 | 0 |
| Female | 1 | 18 to 25 | ............... | 2 | | | 0 | 0 | 0 |
| Female | 1 | 18 to 25 | ............... | 2 | | | 0 | 0 | 0 |
| Female | 1 | 18 to 25 | ............... | 2 | | | 0 | 0 | 0 |
| Male | 1 | 18 to 25 | ............... | 2 | | | 0 | 0 | 0 |
| Female | 1 | 18 to 25 | ............... | 2 | | | 0 | 0 | 0 |
| Female | 1 | 18 to 25 | ............... | 2 | | | 0 | 0 | 0 |
| Female | 1 | 18 to 25 | ............... | 2 | | | 0 | 0 | 0 |
| Female | 1 | 18 to 25 | ............... | 2 | | | 0 | 0 | 0 |
| Female | 1 | 18 to 25 | Yes | 1 | 9,11 | | 0 | 1 | 0 |
| Female | 1 | 18 to 25 | ............... | 2 | | | 0 | 0 | 0 |
| Female | 1 | 18 to 25 | ............... | 2 | | | 0 | 0 | 0 |
| Female | 1 | 18 to 25 | ............... | 2 | | | 0 | 0 | 0 |
| Male | 1 | 18 to 25 | ............... | 2 | | | 0 | 0 | 0 |
| Female | 1 | 18 to 25 | ............... | 2 | | | 0 | 0 | 0 |
| Female | 1 | 18 to 25 | ............... | 2 | | | 0 | 0 | 0 |
| Female | 1 | 18 to 25 | ............... | 2 | | | 0 | 0 | 0 |
| Female | 1 | 18 to 25 | ............... | 2 | | | 0 | 0 | 0 |
| Female | 1 | 18 to 25 | ............... | 2 | | | 0 | 0 | 0 |
| Female | 1 | 18 to 25 | ............... | 2 | | | 0 | 0 | 0 |
| Female | 1 | 18 to 25 | ............... | 2 | | | 0 | 0 | 0 |
| Female | 1 | 18 to 25 | ............... | 2 | | | 0 | 0 | 0 |
| Male | 1 | 18 to 25 | ............... | 2 | | | 0 | 0 | 0 |
| Female | 1 | 18 to 25 | ............... | 2 | | | 0 | 0 | 0 |
| Female | 1 | 18 to 25 | ............... | 2 | | | 0 | 0 | 0 |
| Female | 1 | 18 to 25 | Yes | 1 | | 9 | 0 | 1 | 0 |
| Female | 2 | 26 to 35 | ............... | 2 | | | 0 | 0 | 0 |
| Male | 1 | 18 to 25 | ............... | 2 | | | 0 | 0 | 0 |
| Female | 1 | 18 to 25 | ............... | 2 | | | 0 | 0 | 0 |
| Female | 1 | 18 to 25 | ............... | 2 | | | 0 | 0 | 0 |
| Female | 1 | 18 to 25 | ............... | 2 | | | 0 | 0 | 0 |
| Female | 1 | 18 to 25 | ............... | 2 | | | 0 | 0 | 0 |
| Female | 1 | 18 to 25 | ............... | 2 | | | 0 | 0 | 0 |
| Male | 1 | 18 to 25 | ............... | 2 | | | 0 | 0 | 0 |
| Female | 1 | 18 to 25 | ............... | 2 | | | 0 | 0 | 0 |
| Female | 1 | 18 to 25 | ............... | 2 | | | 0 | 0 | 0 |
| Female | 1 | 18 to 25 | ............... | 2 | | | 0 | 0 | 0 |
| Female | 1 | 18 to 25 | ............... | 2 | | | 0 | 0 | 0 |
| Female | 1 | 18 to 25 | ............... | 2 | | | 0 | 0 | 0 |
| Male | 1 | 18 to 25 | ............... | 2 | | | 0 | 0 | 0 |
| Female | 1 | 18 to 25 | Yes | 1 | 1,8 | | 0 | 0 | 1 |
| Female | 1 | 18 to 25 | ............... | 2 | | | 0 | 0 | 0 |
| Male | 1 | 18 to 25 | Yes | 1 | | 8 | 0 | 0 | 1 |
| Female | 1 | 18 to 25 | ............... | 2 | | | 0 | 0 | 0 |
| Female | 1 | 18 to 25 | ............... | 2 | | | 0 | 0 | 0 |
| Male | 1 | 18 to 25 | ............... | 2 | | | 0 | 0 | 0 |
| Female | 1 | 18 to 25 | ............... | 2 | | | 0 | 0 | 0 |
| Female | 1 | 18 to 25 | ............... | 2 | | | 0 | 0 | 0 |
| Male | 1 | 18 to 25 | ............... | 2 | | | 0 | 0 | 0 |
| Male | 1 | 18 to 25 | ............... | 2 | | | 0 | 0 | 0 |
| Female | 1 | 18 to 25 | ............... | 2 | | | 0 | 0 | 0 |
| Female | 1 | 18 to 25 | ............... | 2 | | | 0 | 0 | 0 |

| | | | | | | | | |
|---|---|---|---|---|---|---|---|---|
| Female | 1 | 18 to 25 | ............... | 2 | | 0 | 0 | 0 |
| Male | 1 | 18 to 25 | ............... | 2 | | 0 | 0 | 0 |
| Female | 1 | 18 to 25 | ............... | 2 | | 0 | 0 | 0 |
| Female | 1 | 18 to 25 | ............... | 2 | | 0 | 0 | 0 |
| Female | 1 | 18 to 25 | ............... | 2 | | 0 | 0 | 0 |
| Female | 1 | 18 to 25 | ............... | 2 | | 0 | 0 | 0 |
| Female | 1 | 18 to 25 | ............... | 2 | | 0 | 0 | 0 |
| Female | 1 | 18 to 25 | ............... | 2 | | 0 | 0 | 0 |
| Female | 1 | 18 to 25 | ............... | 2 | | 0 | 0 | 0 |
| Female | 1 | 18 to 25 | ............... | 2 | | 0 | 0 | 0 |
| Male | 1 | 18 to 25 | ............... | 2 | | 0 | 0 | 0 |
| Female | 1 | 18 to 25 | ............... | 2 | | 0 | 0 | 0 |
| Female | 1 | 18 to 25 | ............... | 2 | | 0 | 0 | 0 |
| Male | 1 | 18 to 25 | ............... | 2 | | 0 | 0 | 0 |
| Female | 1 | 18 to 25 | ............... | 2 | | 0 | 0 | 0 |
| Female | 2 | 26 to 35 | ............... | 2 | | 0 | 0 | 0 |
| Male | 1 | 18 to 25 | Yes | 1 | 4 | 0 | 0 | 0 |
| Male | 1 | 18 to 25 | ............... | 2 | | 0 | 0 | 0 |
| Male | 1 | 18 to 25 | ............... | 2 | | 0 | 0 | 0 |
| Female | 1 | 18 to 25 | ............... | 2 | | 0 | 0 | 0 |
| Female | 1 | 18 to 25 | ............... | 2 | | 0 | 0 | 0 |
| Female | 1 | 18 to 25 | ............... | 2 | | 0 | 0 | 0 |
| Female | 1 | 18 to 25 | ............... | 2 | | 0 | 0 | 0 |
| Female | 1 | 18 to 25 | ............... | 2 | | 0 | 0 | 0 |
| Female | 1 | 18 to 25 | ............... | 2 | | 0 | 0 | 0 |
| Female | 1 | 18 to 25 | ............... | 2 | | 0 | 0 | 0 |
| Male | 1 | 18 to 25 | Yes | 1 | 5 | 0 | 0 | 0 |
| Female | 1 | 18 to 25 | ............... | 2 | | 0 | 0 | 0 |
| Female | 1 | 18 to 25 | ............... | 2 | | 0 | 0 | 0 |
| Female | 1 | 18 to 25 | Do not know/unsure | 3 | 9 | 0 | 1 | 0 |
| Female | 1 | 18 to 25 | ............... | 2 | | 0 | 0 | 0 |
| Female | 1 | 18 to 25 | ............... | 2 | | 0 | 0 | 0 |
| Male | 1 | 18 to 25 | ............... | 2 | | 0 | 0 | 0 |
| Male | 1 | 18 to 25 | Yes | 1 | 5 | 0 | 0 | 0 |
| Female | 1 | 18 to 25 | ............... | 2 | | 0 | 0 | 0 |
| Female | 1 | 18 to 25 | Yes | 1 | 11 | 0 | 0 | 0 |
| Male | 1 | 18 to 25 | Yes | 1 | 11 | 0 | 0 | 0 |
| Female | 1 | 18 to 25 | ............... | 2 | | 0 | 0 | 0 |
| Male | 2 | 26 to 35 | ............... | 2 | | 0 | 0 | 0 |
| Male | 1 | 18 to 25 | ............... | 2 | | 0 | 0 | 0 |
| Female | 1 | 18 to 25 | Yes | 1 | 5 | 0 | 0 | 0 |
| Female | 1 | 18 to 25 | ............... | 2 | | 0 | 0 | 0 |
| Female | 1 | 18 to 25 | ............... | 2 | | 0 | 0 | 0 |
| Female | 1 | 18 to 25 | ............... | 2 | | 0 | 0 | 0 |
| Male | 1 | 18 to 25 | ............... | 2 | | 0 | 0 | 0 |
| Male | 1 | 18 to 25 | ............... | 2 | | 0 | 0 | 0 |
| Female | 2 | 26 to 35 | ............... | 2 | | 0 | 0 | 0 |
| Female | 1 | 18 to 25 | ............... | 2 | | 0 | 0 | 0 |
| Female | 1 | 18 to 25 | ............... | 2 | | 0 | 0 | 0 |
| Female | 1 | 18 to 25 | ............... | 2 | | 0 | 0 | 0 |
| Male | 1 | 18 to 25 | ............... | 2 | | 0 | 0 | 0 |
| Female | 1 | 18 to 25 | ............... | 2 | | 0 | 0 | 0 |

| | | | | | | | | | |
|---|---|---|---|---|---|---|---|---|---|
| Female | 1 | 18 to 25 | .............. | 2 | | | 0 | 0 | 0 |
| Female | 1 | 18 to 25 | .............. | 2 | | | 0 | 0 | 0 |
| Female | 1 | 18 to 25 | .............. | 2 | | | 0 | 0 | 0 |
| Female | 1 | 18 to 25 | .............. | 2 | | | 0 | 0 | 0 |
| Male | 1 | 18 to 25 | .............. | 2 | | | 0 | 0 | 0 |
| Female | 1 | 18 to 25 | .............. | 2 | | | 0 | 0 | 0 |
| Female | 1 | 18 to 25 | .............. | 2 | | | 0 | 0 | 0 |
| Female | 1 | 18 to 25 | .............. | 2 | | | 0 | 0 | 0 |
| Female | 1 | 18 to 25 | .............. | 2 | | | 0 | 0 | 0 |
| Male | 1 | 18 to 25 | .............. | 2 | | | 0 | 0 | 0 |
| Female | 1 | 18 to 25 | .............. | 2 | | | 0 | 0 | 0 |
| Male | 1 | 18 to 25 | .............. | 2 | | | 0 | 0 | 0 |
| Male | 1 | 18 to 25 | .............. | 2 | | | 0 | 0 | 0 |
| Female | 1 | 18 to 25 | .............. | 2 | | | 0 | 0 | 0 |
| Male | 1 | 18 to 25 | .............. | 2 | | | 0 | 0 | 0 |
| Female | 1 | 18 to 25 | .............. | 2 | | | 0 | 0 | 0 |
| Female | 1 | 18 to 25 | .............. | 2 | | | 0 | 0 | 0 |
| Female | 1 | 18 to 25 | .............. | 2 | | | 0 | 0 | 0 |
| Female | 1 | 18 to 25 | .............. | 2 | | | 0 | 0 | 0 |
| Female | 1 | 18 to 25 | .............. | 2 | | | 0 | 0 | 0 |
| Male | 1 | 18 to 25 | .............. | 2 | | | 0 | 0 | 0 |
| Male | 1 | 18 to 25 | .............. | 2 | | | 0 | 0 | 0 |
| Male | 1 | 18 to 25 | .............. | 2 | | | 0 | 0 | 0 |
| Male | 1 | 18 to 25 | Yes | 1 | | 2 | 0 | 0 | 0 |
| Male | 1 | 18 to 25 | Yes | 1 | 5,9 | | 0 | 1 | 0 |
| Male | 1 | 18 to 25 | Yes | 1 | 5,9 | | 0 | 1 | 0 |
| Female | 1 | 18 to 25 | .............. | 2 | | | 0 | 0 | 0 |
| Female | 1 | 18 to 25 | .............. | 2 | | | 0 | 0 | 0 |
| Female | 1 | 18 to 25 | .............. | 2 | | | 0 | 0 | 0 |
| Female | 1 | 18 to 25 | .............. | 2 | | | 0 | 0 | 0 |
| Female | 1 | 18 to 25 | .............. | 2 | | | 0 | 0 | 0 |
| Female | 1 | 18 to 25 | .............. | 2 | | | 0 | 0 | 0 |
| Female | 1 | 18 to 25 | .............. | 2 | | | 0 | 0 | 0 |
| Female | 1 | 18 to 25 | Do not know/unsure | 3 | | | 0 | 0 | 0 |
| Female | 1 | 18 to 25 | .............. | 2 | | | 0 | 0 | 0 |
| Female | 1 | 18 to 25 | .............. | 2 | | | 0 | 0 | 0 |
| Male | 1 | 18 to 25 | .............. | 2 | | | 0 | 0 | 0 |
| Female | 1 | 18 to 25 | .............. | 2 | | | 0 | 0 | 0 |
| | 3 | | | 2 | | | 0 | 0 | 0 |
| Male | 1 | 18 to 25 | .............. | 2 | | | 0 | 0 | 0 |
| Male | 1 | 18 to 25 | .............. | 2 | | | 0 | 0 | 0 |
| Male | 1 | 18 to 25 | Yes | 1 | 2,9 | | 0 | 1 | 0 |
| Male | 1 | 18 to 25 | Yes | 1 | 5,9 | | 0 | 1 | 0 |
| Male | 1 | 18 to 25 | Yes | 1 | 5,9 | | 0 | 1 | 0 |
| Male | 1 | 18 to 25 | Yes | 1 | 5,9 | | 0 | 1 | 0 |
| Male | 1 | 18 to 25 | Yes | 1 | 2,9 | | 0 | 1 | 0 |
| Male | 1 | 18 to 25 | Yes | 1 | | 3 | 0 | 0 | 0 |
| Male | 1 | 18 to 25 | Yes | 1 | 2,8 | | 0 | 0 | 1 |
| Male | 1 | 18 to 25 | Yes | 1 | 5,9 | | 0 | 1 | 0 |
| Male | 1 | 18 to 25 | Yes | 1 | 2,9 | | 0 | 1 | 0 |
| | 1 | 18 to 25 | Yes | 1 | 2,9 | | 0 | 1 | 0 |
| Female | 1 | 18 to 25 | .............. | 2 | | | 0 | 0 | 0 |

| | | | | | | | | | |
|---|---|---|---|---|---|---|---|---|---|
| Female | 1 | 18 to 25 | … | 2 | | | 0 | 0 | 0 |
| Male | 1 | 18 to 25 | … | 2 | | | 0 | 0 | 0 |
| Male | 1 | 18 to 25 | … | 2 | | | 0 | 0 | 0 |
| Male | 1 | 18 to 25 | Yes | 1 | 3,4 | | 0 | 0 | 0 |
| Male | 1 | 18 to 25 | … | 2 | | | 0 | 0 | 0 |
| Female | 1 | 18 to 25 | Yes | 1 | 5,8,9 | | 0 | 1 | 1 |
| Female | 1 | 18 to 25 | … | 2 | | | 0 | 0 | 0 |
| Male | 1 | 18 to 25 | … | 2 | | | 0 | 0 | 0 |
| Male | 1 | 18 to 25 | … | 2 | | | 0 | 0 | 0 |
| Male | 1 | 18 to 25 | … | 2 | | | 0 | 0 | 0 |
| Female | 1 | 18 to 25 | … | 2 | | | 0 | 0 | 0 |
| Female | 2 | 26 to 35 | … | 2 | | | 0 | 0 | 0 |
| Female | 1 | 18 to 25 | … | 2 | | | 0 | 0 | 0 |
| Male | 3 | 36 to 45 | … | 2 | | | 0 | 0 | 0 |
| Female | 1 | 18 to 25 | … | 2 | | | 0 | 0 | 0 |
| Female | 1 | 18 to 25 | … | 2 | | | 0 | 0 | 0 |
| Female | 3 | 46 to 55 | … | 2 | | | 0 | 0 | 0 |
| Female | 1 | 18 to 25 | … | 2 | | | 0 | 0 | 0 |
| Male | 1 | 18 to 25 | Yes | 1 | 5,9 | | 0 | 1 | 0 |
| Female | 1 | 18 to 25 | … | 2 | | | 0 | 0 | 0 |
| Male | 1 | 18 to 25 | Yes | 1 | | 4 | 0 | 0 | 0 |
| Female | 1 | 18 to 25 | … | 2 | | | 0 | 0 | 0 |
| Female | 1 | 18 to 25 | … | 2 | | | 0 | 0 | 0 |
| Female | 1 | 18 to 25 | … | 2 | | | 0 | 0 | 0 |
| Male | 1 | 18 to 25 | Yes | 1 | 5,9 | | 0 | 1 | 0 |
| Female | 1 | 18 to 25 | … | 2 | | | 0 | 0 | 0 |
| Male | 1 | 18 to 25 | … | 2 | | | 0 | 0 | 0 |
| Male | 1 | 18 to 25 | … | 2 | | | 0 | 0 | 0 |
| Female | 1 | 18 to 25 | Yes | 1 | | 3 | 0 | 0 | 0 |
| Male | 1 | 18 to 25 | … | 2 | | | 0 | 0 | 0 |
| Male | 1 | 18 to 25 | … | 2 | | | 0 | 0 | 0 |
| Female | 1 | 18 to 25 | … | 2 | | | 0 | 0 | 0 |
| Male | 1 | 18 to 25 | … | 2 | | | 0 | 0 | 0 |
| Female | 1 | 18 to 25 | Yes | 1 | | 2 | 0 | 0 | 0 |
| Male | 1 | 18 to 25 | Yes | 1 | 2,9,10 | | 0 | 1 | 0 |
| Male | 1 | 18 to 25 | Yes | 1 | 3, | | 0 | 0 | 0 |
| Male | 1 | 18 to 25 | Yes | 1 | | 4 | 0 | 0 | 0 |
| Female | 1 | 18 to 25 | … | 2 | | | 0 | 0 | 0 |
| Female | 1 | 18 to 25 | … | 2 | | | 0 | 0 | 0 |
| Female | 1 | 18 to 25 | … | 2 | | | 0 | 0 | 0 |
| Female | 1 | 18 to 25 | … | 2 | | | 0 | 0 | 0 |
| Female | 1 | 18 to 25 | … | 2 | | | 0 | 0 | 0 |
| Female | 1 | 18 to 25 | … | 2 | | | 0 | 0 | 0 |
| Female | 1 | 18 to 25 | … | 2 | | | 0 | 0 | 0 |
| Male | 1 | 18 to 25 | Yes | 1 | 5,9 | | 0 | 1 | 0 |
| Female | 1 | 18 to 25 | … | 2 | | | 0 | 0 | 0 |
| Female | 1 | 18 to 25 | … | 2 | | | 0 | 0 | 0 |
| Female | 1 | 18 to 25 | … | 2 | | | 0 | 0 | 0 |
| Male | 1 | 18 to 25 | Yes | 1 | 5,6,9 | | 1 | 1 | 0 |
| Female | 1 | 18 to 25 | Yes | 1 | 5,9 | | 0 | 1 | 0 |
| Female | 1 | 18 to 25 | … | 2 | | | 0 | 0 | 0 |
| Male | 1 | 18 to 25 | … | 2 | | | 0 | 0 | 0 |

| | | | | | | | |
|---|---|---|---|---|---|---|---|
| Male | 1 | 18 to 25 | [illegible] | 2 | 0 | 0 | 0 |
| Female | 1 | 18 to 25 | [illegible] | 2 | 0 | 0 | 0 |
| Female | 1 | 18 to 25 | [illegible] | 2 | 0 | 0 | 0 |
| Male | 1 | 18 to 25 | Yes | 1 | 0 | 0 | 0 |
| Male | 1 | 18 to 25 | [illegible] | 2 | 0 | 0 | 0 |
| Male | 1 | 18 to 25 | [illegible] | 2 | 0 | 0 | 0 |
| Male | 1 | 18 to 25 | [illegible] | 2 | 0 | 0 | 0 |
| Male | 1 | 18 to 25 | [illegible] | 2 | 0 | 0 | 0 |
| Male | 1 | 18 to 25 | [illegible] | 2 | 0 | 0 | 0 |
| Male | 1 | 18 to 25 | [illegible] | 2 | 0 | 0 | 0 |
| Male | 1 | 18 to 25 | [illegible] | 2 | 0 | 0 | 0 |
| Male | 1 | 18 to 25 | [illegible] | 2 | 0 | 0 | 0 |
| Male | 1 | 18 to 25 | [illegible] | 2 | 0 | 0 | 0 |
| Male | 1 | 18 to 25 | [illegible] | 2 | 0 | 0 | 0 |
| Male | 1 | 18 to 25 | [illegible] | 2 | 0 | 0 | 0 |
| Male | 1 | 18 to 25 | [illegible] | 2 | 0 | 0 | 0 |
| Female | 1 | 18 to 25 | | 2 | 0 | 0 | 0 |
| Male | 1 | 18 to 25 | | 2 | 0 | 0 | 0 |
| Female | 1 | 18 to 25 | | 2 | 0 | 0 | 0 |
| Female | 3 | 46 to 55 | | 2 | 0 | 0 | 0 |
| Female | 1 | 18 to 25 | | 2 | 0 | 0 | 0 |
| Female | 1 | 18 to 25 | | 2 | 0 | 0 | 0 |
| Female | 1 | 18 to 25 | | 2 | 0 | 0 | 0 |
| Female | 1 | 18 to 25 | | 2 | 0 | 0 | 0 |
| Male | 1 | 18 to 25 | Yes | 1 | 0 | 1 | 0 |
| Female | 1 | 18 to 25 | | 2 | 0 | 0 | 0 |
| Female | 3 | 36 to 45 | Yes | 1 | 0 | 0 | 0 |
| Female | 1 | 18 to 25 | | 2 | 0 | 0 | 0 |

| | | | | | | | |
|---|---|---|---|---|---|---|---|
| Male | 1 | 18 to 25 | Yes | 1 | 0 | 1 | 0 |
| Female | 1 | 18 to 25 | | 2 | 0 | 0 | 0 |
| Female | 1 | 18 to 25 | Yes | 1 | 0 | 0 | 1 |
| Female | 1 | 18 to 25 | | 2 | 0 | 0 | 0 |
| Female | 1 | 18 to 25 | | 2 | 0 | 0 | 0 |
| Male | 1 | 18 to 25 | | 2 | 0 | 0 | 0 |
| Male | 1 | 18 to 25 | Yes | 1 | 0 | 0 | 0 |
| Male | 1 | 18 to 25 | | 2 | 0 | 0 | 0 |
| Male | 1 | 18 to 25 | Yes | 1 | 0 | 0 | 0 |
| Male | 1 | 18 to 25 | Yes | 1 | 0 | 0 | 1 |
| Male | 1 | 18 to 25 | | 2 | 0 | 0 | 0 |
| Female | 1 | 18 to 25 | Yes | 1 | 0 | 1 | 0 |
| Male | 1 | 18 to 25 | Yes | 1 | 0 | 1 | 0 |

| | | | | | | | |
|---|---|---|---|---|---|---|---|
| Female | 1 | 18 to 25 | Yes | 1 | 0 | 1 | 1 |
| Female | 1 | 18 to 25 | | 2 | 0 | 0 | 0 |
| Male | 1 | 18 to 25 | | 2 | 0 | 0 | 0 |
| Female | 1 | 18 to 25 | | 2 | 0 | 0 | 0 |
| Female | 1 | 18 to 25 | Yes | 1 | 0 | 0 | 0 |
| Male | 1 | 18 to 25 | Yes | 1 | 0 | 0 | 0 |
| Male | 1 | 18 to 25 | | 2 | 0 | 0 | 0 |
| Male | 1 | 18 to 25 | Yes | 1 | 0 | 0 | 0 |
| Male | 1 | 18 to 25 | | 2 | 0 | 0 | 0 |
| Female | 1 | 18 to 25 | Yes | 1 | 0 | 1 | 0 |
| Male | 1 | 18 to 25 | | 2 | 0 | 0 | 0 |
| Female | 2 | 26 to 35 | Yes | 1 | 0 | 0 | 0 |
| Male | 1 | 18 to 25 | Yes | 1 | 0 | 0 | 0 |

| | | | | | | | |
|---|---|---|---|---|---|---|---|
| Male | 1 | 18 to 25 | Yes | 1 | 0 | 0 | 1 |
| Female | 1 | 18 to 25 | Yes | 1 | 0 | 0 | 0 |

| | |
|---|---|
| 1 18-25 | 1 yes |
| 2 26-35 | 2 no |
| 3 above | 3 not sure |

| ritalin4 | adderall3 | coffe | modafinil | singleMultiple | Q4 If 'Yes', which one? (select all that apply) |
|---|---|---|---|---|---|
| 0 | 0 | 0 | 0 | | |
| 0 | 0 | 0 | 0 | | |
| 0 | 0 | 0 | 0 | 2 | Alpha Brain/vitamin B6,Cobalamin/ vitamin B12 |
| 0 | 0 | 0 | 0 | | |
| 0 | 0 | 0 | 0 | | |
| 0 | 0 | 0 | 0 | | |
| 0 | 0 | 0 | 0 | | |
| 0 | 0 | 0 | 0 | | |
| 0 | 0 | 0 | 0 | | |
| 0 | 0 | 0 | 0 | | |
| 0 | 0 | 0 | 0 | | |
| 0 | 0 | 0 | 0 | | |
| 0 | 0 | 0 | 0 | | |
| 0 | 0 | 0 | 0 | | |
| 0 | 0 | 0 | 0 | | |
| 0 | 0 | 0 | 0 | | |
| 0 | 0 | 0 | 0 | | |
| 0 | 0 | 0 | 0 | | |
| 0 | 0 | 0 | 0 | | |
| 0 | 0 | 0 | 0 | 1 | Cobalamin/ vitamin B12 |
| 0 | 0 | 0 | 0 | | |
| 0 | 0 | 0 | 0 | | |
| 0 | 0 | 0 | 0 | | |
| 0 | 0 | 0 | 0 | | |
| 0 | 0 | 0 | 0 | | |
| 0 | 0 | 0 | 0 | | |
| 0 | 0 | 1 | 0 | 1 | Super strength caffeine pills |
| 0 | 0 | 0 | 0 | | |
| 0 | 0 | 0 | 0 | | |
| 0 | 0 | 0 | 0 | | |
| 0 | 0 | 1 | 0 | 2 | [illegible] |
| 0 | 0 | 0 | 0 | | |
| 0 | 0 | 0 | 0 | | |
| 0 | 0 | 0 | 0 | | |
| 0 | 0 | 0 | 0 | 1 | Cobalamin/ vitamin B12 |
| 0 | 0 | 0 | 0 | | |
| 0 | 0 | 0 | 0 | | |
| 0 | 0 | 0 | 0 | | |
| 0 | 0 | 0 | 0 | | |
| 0 | 0 | 0 | 0 | | |
| 0 | 0 | 0 | 0 | | |
| 0 | 0 | 0 | 0 | | |
| 0 | 0 | 0 | 0 | | |
| 0 | 0 | 0 | 0 | | |
| 0 | 0 | 1 | 0 | 1 | Super strength caffeine pills |
| 0 | 0 | 0 | 0 | | |
| 0 | 0 | 0 | 0 | | |
| 0 | 0 | 1 | 0 | 2 | Super strength caffeine pills,Alpha Brain/vitamin B6,Cobalamin/vitamin B12 |
| 0 | 0 | 0 | 0 | | |
| 0 | 0 | 1 | 0 | 1 | Super strength caffeine pills |

| | | | | | |
|---|---|---|---|---|---|
| 0 | 0 | | 0 | | |
| 0 | 0 | 0 | 0 | | |
| 0 | 0 | 0 | 0 | | |
| 0 | 0 | 0 | 0 | | |
| 0 | 0 | 0 | 0 | 2 | Alpha Brain/vitamin B6,Cobalamin/ vitamin B12 |
| 0 | 0 | 0 | 0 | | |
| 0 | 0 | 0 | 0 | | |
| 0 | 0 | 0 | 0 | 1 | Other drug or substance used |
| 0 | 0 | 0 | 0 | | |
| 1 | 0 | 0 | 0 | 1 | Ritalin/amphetamine |
| 0 | 0 | 0 | 0 | 2 | MDMA/ ecstasy,Cobalamin/ vitamin B12 |
| 0 | 0 | 0 | 0 | | |
| 0 | 0 | 0 | 0 | | |
| 0 | 0 | 0 | 0 | | |
| 0 | 0 | 1 | 0 | 1 | Super strength caffeine pills,Guarana |
| 0 | 0 | 0 | 1 | 1 | Modafinil |
| 0 | 0 | 0 | 0 | | |
| 0 | 0 | 0 | 0 | | |
| 0 | 0 | 0 | 0 | | |
| 0 | 0 | 0 | 0 | | |
| 0 | 0 | 0 | 0 | | |
| 0 | 0 | 0 | 0 | | |
| 0 | 0 | 0 | 0 | | |
| 0 | 0 | 0 | 0 | | |
| 0 | 0 | 0 | 0 | | |
| 0 | 0 | 0 | 0 | | |
| 0 | 0 | 0 | 0 | | |
| 0 | 0 | 0 | 1 | 2 | Modafinil,Cobalamin/ vitamin B12 |
| 0 | 1 | 0 | 0 | 1 | Adderall/ amphetamines mixture |
| 0 | 0 | 1 | 0 | 1 | Super strength caffeine pills |
| 0 | 0 | 1 | 0 | 1 | Super strength caffeine pills |
| 0 | 0 | 0 | 0 | | |
| 0 | 0 | 1 | 0 | 2 | Super strength caffeine pills,Cobalamin/ vitamin B12 |
| 0 | 0 | 0 | 1 | 1 | Modafinil |
| 0 | 0 | 1 | 1 | 2 | Modafinil,Super strength caffeine pills |
| 0 | 0 | 1 | 1 | 2 | Modafinil,Super strength caffeine pills,Cobalamin/ vitamin B12 |
| 0 | 0 | 0 | 1 | 2 | Modafinil,Cobalamin/ vitamin B12 |
| 0 | 0 | 0 | 0 | 1 | Cobalamin/ vitamin B12 |
| 0 | 0 | 0 | 1 | 1 | Modafinil |
| 0 | 0 | 0 | 1 | 2 | Modafinil,Cobalamin/ vitamin B12 |
| 0 | 0 | 0 | 1 | 2 | Modafinil,Cobalamin/ vitamin B12 |
| 0 | 0 | 1 | 0 | 1 | Super strength caffeine pills |
| 0 | 0 | 0 | 0 | | |
| 0 | 0 | 1 | 1 | 2 | Modafinil,Super strength caffeine pills |
| 0 | 0 | 1 | 1 | 2 | Modafinil,Super strength caffeine pills,Guarana |
| 0 | 0 | 1 | 0 | 2 | Super strength caffeine pills,Cobalamin/ vitamin B12 |
| 0 | 0 | 1 | 1 | 2 | Modafinil,Super strength caffeine pills,Cobalamin/ vitamin B12 |
| 0 | 0 | 1 | 0 | 1 | Super strength caffeine pills |
| 0 | 0 | 0 | 0 | | |
| 0 | 0 | 0 | 1 | 2 | Modafinil,Cobalamin/ vitamin B12 |
| 0 | 0 | 0 | 1 | 1 | Modafinil |
| 0 | 1 | 0 | 0 | 1 | Adderall/ amphetamines mixture |

| | | | | | |
|---|---|---|---|---|---|
| 0 | 0 | 0 | 0 | | |
| 0 | 0 | 0 | 0 | | |
| 0 | 0 | 1 | 1 | 2 | Modafinil,Super strength caffeine pills |
| 0 | 1 | 0 | 0 | 1 | Adderall/amphetamines mixture |
| 0 | 0 | 0 | 0 | | |
| 1 | 1 | 0 | 0 | 2 | Adderall/amphetamines mixture,Ritalin/amphetamine |
| 0 | 0 | 0 | 1 | 2 | Modafinil,Cobalamin/ vitamin B12 |
| 0 | 0 | 0 | 1 | 2 | Modafinil,Cobalamin/ vitamin B12 |
| 0 | 0 | 1 | 1 | 2 | Modafinil,Super strength caffeine pills |
| 1 | 1 | 0 | 0 | 2 | Adderall/amphetamines mixture,Ritalin/amphetamine |
| 0 | 0 | 0 | 0 | | |
| 0 | 0 | 0 | 0 | | |
| 0 | 0 | 1 | 1 | 2 | Modafinil,Super strength caffeine pills |
| 0 | 0 | 0 | 0 | | |
| 0 | 0 | 0 | 0 | | |
| 1 | 1 | 0 | 0 | 2 | Adderall/amphetamines mixture,Ritalin/amphetamine |
| 0 | 0 | 0 | 0 | | |
| 0 | 0 | 0 | 0 | | |
| 0 | 0 | 0 | 0 | | |
| 0 | 0 | 0 | 0 | | |
| 0 | 0 | 0 | 1 | 2 | Modafinil,Cobalamin/ vitamin B12 |
| 0 | 0 | 0 | 0 | | |
| 0 | 0 | 0 | 0 | 1 | MDMA/ ecstasy |
| 0 | 0 | 0 | 0 | | |
| 0 | 0 | 0 | 0 | | |
| 0 | 0 | 0 | 0 | | |
| 0 | 0 | 0 | 1 | 1 | Modafinil |
| 0 | 0 | 1 | 1 | 2 | Modafinil,Super strength caffeine pills |
| 0 | 0 | 0 | 0 | | |
| 0 | 0 | 0 | 0 | | |
| 0 | 0 | 0 | 0 | | |
| 0 | 0 | 0 | 0 | | |
| 0 | 0 | 0 | 0 | | |
| 1 | 0 | 0 | 0 | 1 | Ritalin/amphetamine |
| 0 | 0 | 0 | 0 | | |
| 0 | 0 | 0 | 0 | | |
| 0 | 0 | 0 | 1 | 1 | Modafinil |
| 0 | 0 | 0 | 1 | 1 | Modafinil |
| 0 | 0 | 0 | 0 | | |
| 0 | 0 | 0 | 0 | | |
| 0 | 0 | 0 | 0 | 2 | Guarana,Piracetam |
| 0 | 1 | 0 | 0 | 1 | Adderall/amphetamines mixture |
| 0 | 0 | 0 | 1 | 1 | Modafinil |
| 0 | 0 | 0 | 0 | | |
| 0 | 0 | 0 | 0 | | |
| 0 | 0 | 0 | 0 | | |
| 0 | 0 | 0 | 0 | | |
| 0 | 0 | 0 | 0 | | |
| 0 | 0 | 0 | 0 | | |
| 0 | 0 | 0 | 0 | | |
| 0 | 0 | 0 | 0 | | |
| 0 | 0 | 0 | 1 | 1 | Modafinil |

| | | | | | |
|---|---|---|---|---|---|
| 0 | 0 | 1 | 1 | 2 | Modafinil,Super strength caffeine pills |
| 0 | 0 | 0 | 0 | | |
| 0 | 0 | 0 | 0 | | |
| 0 | 0 | 0 | 0 | | |
| 0 | 0 | 0 | 1 | 1 | Modafinil |
| 0 | 0 | 0 | 1 | 1 | Modafinil |
| 0 | 0 | 0 | 0 | | |
| 0 | 0 | 0 | 0 | | |
| 0 | 0 | 0 | 0 | | |
| 0 | 0 | 0 | 0 | | |
| 0 | 0 | 0 | 0 | | |
| 1 | 0 | 0 | 1 | 2 | Modafinil,Ritalin/amphetamine |
| 0 | 0 | 0 | 0 | | |
| 0 | 0 | 0 | 0 | | |
| 0 | 0 | 0 | 0 | | |
| 1 | 0 | 0 | 1 | 2 | Modafinil,Ritalin/amphetamine |
| 0 | 0 | 0 | 0 | | |
| 0 | 0 | 0 | 0 | | |
| 0 | 0 | 0 | 0 | | |
| 0 | 0 | 0 | 0 | | |
| 0 | 0 | 0 | 0 | | |
| 0 | 0 | 0 | 0 | | |
| 0 | 0 | 0 | 0 | | |
| 0 | 0 | 0 | 1 | 1 | Modafinil |
| 0 | 0 | 0 | 0 | | |
| 0 | 0 | 0 | 0 | | |
| 0 | 0 | 0 | 0 | | |
| 0 | 0 | 0 | 0 | | |
| 0 | 0 | 0 | 0 | | |
| 0 | 0 | 0 | 0 | | |
| 0 | 0 | 0 | 0 | | |
| 0 | 0 | 1 | 0 | 1 | Super strength caffeine pills |
| 0 | 0 | 0 | 0 | | |
| 0 | 0 | 1 | 1 | 2 | Modafinil,Super strength caffeine pills |
| 0 | 0 | 0 | 0 | | |
| 0 | 0 | 1 | 0 | 1 | Super strength caffeine pills |
| 0 | 0 | 0 | 0 | | |
| 0 | 0 | 0 | 1 | 2 | Modafinil,Guarana |
| 0 | 0 | 0 | 0 | | |
| 0 | 0 | 1 | 1 | 2 | Modafinil,Super strength caffeine pills |
| 0 | 0 | 0 | 0 | | |
| 0 | 0 | 0 | 0 | | |
| 0 | 0 | 0 | 0 | | |
| 0 | 0 | 0 | 0 | | |
| 0 | 0 | 0 | 0 | | |
| 0 | 0 | 0 | 0 | | |
| 0 | 0 | 0 | 0 | | |
| 0 | 0 | 0 | 0 | | |
| 0 | 0 | 0 | 0 | | |
| 0 | 0 | 0 | 0 | | |
| 0 | 0 | 0 | 0 | | |
| 0 | 0 | 0 | 0 | | |

| | | | | | |
|---|---|---|---|---|---|
| 0 | 0 | 0 | 0 | | |
| 0 | 0 | 0 | 0 | | |
| 0 | 0 | 0 | 0 | | |
| 0 | 0 | 0 | 0 | | |
| 0 | 0 | 0 | 0 | | |
| 0 | 0 | 0 | 0 | | |
| 0 | 0 | 0 | 0 | 1 | Other drug or substance used |
| 0 | 0 | 0 | 0 | | |
| 0 | 0 | 0 | 0 | | |
| 0 | 0 | 0 | 0 | | |
| 0 | 0 | 0 | 0 | | |
| 0 | 0 | 0 | 0 | 1 | Other drug or substance used |
| 0 | 0 | 0 | 0 | 1 | Cobalamin/vitamin B12 |
| 0 | 0 | 0 | 0 | | |
| 0 | 0 | 0 | 0 | | |
| 0 | 0 | 0 | 0 | | |
| 0 | 0 | 0 | 0 | | |
| 0 | 0 | 0 | 0 | | |
| 0 | 0 | 0 | 0 | | |
| 0 | 0 | 0 | 0 | | |
| 0 | 0 | 0 | 0 | | |
| 1 | 0 | 0 | 0 | 2 | Ritalin/amphetamine,AlphaBrain/vitamin B6,Cobalamin/vitamin B12 |
| 0 | 0 | 0 | 0 | | |
| 0 | 0 | 0 | 0 | | |
| 0 | 0 | 0 | 0 | | |
| 0 | 0 | 0 | 0 | | |
| 0 | 0 | 0 | 0 | | |
| 0 | 0 | 0 | 0 | | |
| 0 | 0 | 0 | 0 | | |
| 0 | 0 | 0 | 0 | | |
| 0 | 0 | 0 | 0 | 1 | Cobalamin/vitamin B12 |
| 0 | 0 | 0 | 0 | | |
| 1 | 0 | 0 | 0 | 1 | Ritalin/amphetamine |
| 0 | 0 | 0 | 0 | | |
| 0 | 0 | 0 | 0 | 1 | Cobalamin/vitamin B12 |
| 0 | 0 | 0 | 0 | | |
| 0 | 0 | 0 | 0 | | |
| 0 | 0 | 0 | 0 | | |
| 0 | 0 | 0 | 0 | | |
| 0 | 0 | 0 | 0 | | |
| 0 | 0 | 0 | 0 | | |
| 0 | 0 | 0 | 0 | | |
| 0 | 0 | 0 | 0 | | |
| 0 | 0 | 0 | 0 | | |
| 0 | 0 | 0 | 0 | | |
| 0 | 0 | 0 | 0 | | |
| 0 | 0 | 0 | 0 | | |
| 0 | 0 | 0 | 0 | | |
| 0 | 0 | 0 | 0 | | |
| 0 | 0 | 0 | 0 | 2 | Alpha Brain/vitamin B6,Cobalamin/vitamin B12 |
| 0 | 0 | 0 | 0 | | |
| 0 | 0 | 0 | 0 | | |

| | | | | | |
|---|---|---|---|---|---|
| 0 | 0 | 0 | 0 | | |
| 0 | 0 | 0 | 0 | | |
| 0 | 0 | 0 | 0 | | |
| 0 | 0 | 0 | 0 | | |
| 0 | 0 | 0 | 0 | | |
| 0 | 0 | 0 | 0 | | |
| 0 | 0 | 0 | 0 | | |
| 0 | 0 | 0 | 0 | | |
| 0 | 0 | 0 | 0 | | |
| 0 | 0 | 0 | 0 | 2 | Cobalamin/vitamin B12,Other drug or substance used |
| 0 | 0 | 0 | 0 | | |
| 0 | 0 | 0 | 0 | | |
| 0 | 0 | 0 | 0 | | |
| 0 | 0 | 0 | 0 | | |
| 0 | 0 | 0 | 0 | | |
| 0 | 0 | 0 | 0 | | |
| 0 | 0 | 0 | 0 | | |
| 0 | 0 | 0 | 0 | | |
| 0 | 0 | 0 | 0 | | |
| 0 | 0 | 0 | 0 | | |
| 0 | 0 | 0 | 0 | | |
| 0 | 0 | 0 | 0 | | |
| 0 | 0 | 0 | 0 | | |
| 0 | 0 | 0 | 0 | | |
| 0 | 0 | 0 | 0 | | |
| 0 | 0 | 0 | 0 | 1 | Cobalamin/vitamin B12 |
| 0 | 0 | 0 | 0 | | |
| 0 | 0 | 0 | 0 | | |
| 0 | 0 | 0 | 0 | | |
| 0 | 0 | 0 | 0 | | |
| 0 | 0 | 0 | 0 | | |
| 0 | 0 | 0 | 0 | | |
| 0 | 0 | 0 | 0 | | |
| 0 | 0 | 0 | 0 | | |
| 0 | 0 | 0 | 0 | | |
| 0 | 0 | 0 | 0 | | |
| 0 | 0 | 0 | 0 | | |
| 0 | 0 | 0 | 0 | | |
| 0 | 0 | 0 | 0 | | |
| 0 | 0 | 0 | 0 | | |
| 0 | 0 | 0 | 0 | 2 | MDMA/ ecstasy,Alpha Brain/vitamin B6 |
| 0 | 0 | 0 | 0 | | |
| 0 | 0 | 0 | 0 | 1 | Alpha Brain/vitamin B6 |
| 0 | 0 | 0 | 0 | | |
| 0 | 0 | 0 | 0 | | |
| 0 | 0 | 0 | 0 | | |
| 0 | 0 | 0 | 0 | | |
| 0 | 0 | 0 | 0 | | |
| 0 | 0 | 0 | 0 | | |
| 0 | 0 | 0 | 0 | | |
| 0 | 0 | 0 | 0 | | |
| 0 | 0 | 0 | 0 | | |

| | | | | | |
|---|---|---|---|---|---|
| 0 | 0 | 0 | 0 | | |
| 0 | 0 | 0 | 0 | | |
| 0 | 0 | 0 | 0 | | |
| 0 | 0 | 0 | 0 | | |
| 0 | 0 | 0 | 0 | | |
| 0 | 0 | 0 | 0 | | |
| 0 | 0 | 0 | 0 | | |
| 0 | 0 | 0 | 0 | | |
| 0 | 0 | 0 | 0 | | |
| 0 | 0 | 0 | 0 | | |
| 0 | 0 | 0 | 0 | | |
| 0 | 0 | 0 | 0 | | |
| 0 | 0 | 0 | 0 | | |
| 0 | 0 | 0 | 0 | | |
| 0 | 0 | 0 | 0 | | |
| 0 | 0 | 0 | 0 | | |
| 1 | 0 | 0 | 0 | 1 | Ritalin/amphetamine |
| 0 | 0 | 0 | 0 | | |
| 0 | 0 | 0 | 0 | | |
| 0 | 0 | 0 | 0 | | |
| 0 | 0 | 0 | 0 | | |
| 0 | 0 | 0 | 0 | | |
| 0 | 0 | 0 | 0 | | |
| 0 | 0 | 0 | 0 | | |
| 0 | 0 | 0 | 0 | | |
| 0 | 0 | 0 | 0 | | |
| 0 | 0 | 1 | 0 | 1 | Super strength caffeine pills |
| 0 | 0 | 0 | 0 | | |
| 0 | 0 | 0 | 0 | | |
| 0 | 0 | 0 | 0 | 1 | Cobalamin/vitamin B12 |
| 0 | 0 | 0 | 0 | | |
| 0 | 0 | 0 | 0 | | |
| 0 | 0 | 0 | 0 | | |
| 0 | 0 | 1 | 0 | 1 | Super strength caffeine pills |
| 0 | 0 | 0 | 0 | | |
| 0 | 0 | 0 | 0 | 1 | Other drug or substance used |
| 0 | 0 | 0 | 0 | 1 | Other drug or substance used |
| 0 | 0 | 0 | 0 | | |
| 0 | 0 | 0 | 0 | | |
| 0 | 0 | 0 | 0 | | |
| 0 | 0 | 1 | 0 | 1 | Super strength caffeine pills |
| 0 | 0 | 0 | 0 | | |
| 0 | 0 | 0 | 0 | | |
| 0 | 0 | 0 | 0 | | |
| 0 | 0 | 0 | 0 | | |
| 0 | 0 | 0 | 0 | | |
| 0 | 0 | 0 | 0 | | |
| 0 | 0 | 0 | 0 | | |
| 0 | 0 | 0 | 0 | | |
| 0 | 0 | 0 | 0 | | |
| 0 | 0 | 0 | 0 | | |
| 0 | 0 | 0 | 0 | | |

| | | | | | |
|---|---|---|---|---|---|
| 0 | 0 | 0 | 0 | | |
| 0 | 0 | 0 | 0 | | |
| 0 | 0 | 0 | 0 | | |
| 0 | 0 | 0 | 0 | | |
| 0 | 0 | 0 | 0 | | |
| 0 | 0 | 0 | 0 | | |
| 0 | 0 | 0 | 0 | | |
| 0 | 0 | 0 | 0 | | |
| 0 | 0 | 0 | 0 | | |
| 0 | 0 | 0 | 0 | | |
| 0 | 0 | 0 | 0 | | |
| 0 | 0 | 0 | 0 | | |
| 0 | 0 | 0 | 0 | | |
| 0 | 0 | 0 | 0 | | |
| 0 | 0 | 0 | 0 | | |
| 0 | 0 | 0 | 0 | | |
| 0 | 0 | 0 | 0 | | |
| 0 | 0 | 0 | 0 | | |
| 0 | 0 | 0 | 0 | | |
| 0 | 0 | 0 | 0 | | |
| 0 | 0 | 0 | 0 | | |
| 0 | 0 | 0 | 0 | | |
| 0 | 0 | 0 | 0 | | |
| 0 | 0 | 0 | 1 | 1 | Modafinil |
| 0 | 0 | 1 | 0 | 2 | Super strength caffeine pills,Cobalamin/ vitamin B12 |
| 0 | 0 | 1 | 0 | 2 | Super strength caffeine pills,Cobalamin/ vitamin B12 |
| 0 | 0 | 0 | 0 | | |
| 0 | 0 | 0 | 0 | | |
| 0 | 0 | 0 | 0 | | |
| 0 | 0 | 0 | 0 | | |
| 0 | 0 | 0 | 0 | | |
| 0 | 0 | 0 | 0 | | |
| 0 | 0 | 0 | 0 | | |
| 0 | 0 | 0 | 0 | | |
| 0 | 0 | 0 | 0 | | |
| 0 | 0 | 0 | 0 | | |
| 0 | 0 | 0 | 0 | | |
| 0 | 0 | 0 | 0 | | |
| 0 | 0 | 0 | 0 | | |
| 0 | 0 | 0 | 0 | | |
| 0 | 0 | 0 | 0 | | |
| 0 | 0 | 0 | 1 | 2 | Modafinil,Cobalamin/ vitamin B12 |
| 0 | 0 | 1 | 0 | 2 | Super strength caffeine pills,Cobalamin/ vitamin B12 |
| 0 | 0 | 1 | 0 | 2 | Super strength caffeine pills,Cobalamin/ vitamin B12 |
| 0 | 0 | 1 | 0 | 2 | Super strength caffeine pills,Cobalamin/ vitamin B12 |
| 0 | 0 | 0 | 1 | 2 | Modafinil,Cobalamin/ vitamin B12 |
| 0 | 0 | 0 | 0 | 1 | Adderall/ amphetamines mixture |
| 0 | 0 | 0 | 1 | 2 | Modafinil,Alpha Brain/ vitamin B6 |
| 0 | 0 | 1 | 0 | 2 | Super strength caffeine pills,Cobalamin/ vitamin B12 |
| 0 | 0 | 0 | 1 | 2 | Modafinil,Cobalamin/ vitamin B12 |
| 0 | 0 | 0 | 1 | 2 | Modafinil,Cobalamin/ vitamin B12 |
| 0 | 0 | 0 | 0 | | |

| | | | | | |
|---|---|---|---|---|---|
| 0 | 0 | 0 | 0 | | |
| 0 | 0 | 0 | 0 | | |
| 0 | 0 | 0 | 0 | | |
| 1 | 1 | 0 | 0 | 2 | Adderall/amphetamines mixture,Ritalin/amphetamine |
| 0 | 0 | 0 | 0 | | |
| 0 | 0 | 1 | 0 | 2 | Super strength caffeine pills,AlphaBrain/vitamin B6,Cobalamin/vitamin B12 |
| 0 | 0 | 0 | 0 | | |
| 0 | 0 | 0 | 0 | | |
| 0 | 0 | 0 | 0 | | |
| 0 | 0 | 0 | 0 | | |
| 0 | 0 | 0 | 0 | | |
| 0 | 0 | 0 | 0 | | |
| 0 | 0 | 0 | 0 | | |
| 0 | 0 | 0 | 0 | | |
| 0 | 0 | 0 | 0 | | |
| 0 | 0 | 0 | 0 | | |
| 0 | 0 | 0 | 0 | | |
| 0 | 0 | 0 | 0 | | |
| 0 | 0 | 1 | 0 | 2 | Super strength caffeine pills,Cobalamin/vitamin B12 |
| 0 | 0 | 0 | 0 | | |
| 1 | 0 | 0 | 0 | 1 | Ritalin/amphetamine |
| 0 | 0 | 0 | 0 | | |
| 0 | 0 | 0 | 0 | | |
| 0 | 0 | 0 | 0 | | |
| 0 | 0 | 1 | 0 | 2 | Super strength caffeine pills,Cobalamin/vitamin B12 |
| 0 | 0 | 0 | 0 | | |
| 0 | 0 | 0 | 0 | | |
| 0 | 0 | 0 | 0 | | |
| 0 | 1 | 0 | 0 | 1 | Adderall/amphetamines mixture |
| 0 | 0 | 0 | 0 | | |
| 0 | 0 | 0 | 0 | | |
| 0 | 0 | 0 | 0 | | |
| 0 | 0 | 0 | 0 | | |
| 0 | 0 | 0 | 1 | 1 | Modafinil |
| 0 | 0 | 0 | 1 | 2 | Modafinil,Cobalamin/vitamin B12,Vinpocetine |
| 0 | 1 | 0 | 0 | 1 | Adderall/amphetamines mixture |
| 0 | 0 | 0 | 0 | 1 | Ritalin/amphetamine |
| 0 | 0 | 0 | 0 | | |
| 0 | 0 | 0 | 0 | | |
| 0 | 0 | 0 | 0 | | |
| 0 | 0 | 0 | 0 | | |
| 0 | 0 | 0 | 0 | | |
| 0 | 0 | 0 | 0 | | |
| 0 | 0 | 0 | 0 | | |
| 0 | 0 | 1 | 0 | 2 | Super strength caffeine pills,Cobalamin/vitamin B12 |
| 0 | 0 | 0 | 0 | | |
| 0 | 0 | 0 | 0 | | |
| 0 | 0 | 0 | 0 | | |
| 0 | 0 | 1 | 0 | 2 | Super strength caffeine pills,Guarana,Cobalamin/vitamin B12 |
| 0 | 0 | 1 | 0 | 2 | Super strength caffeine pills,Cobalamin/vitamin B12 |
| 0 | 0 | 0 | 0 | | |
| 0 | 0 | 0 | 0 | | |

| | | | | | |
|---|---|---|---|---|---|
| 0 | 0 | 0 | 0 | | |
| 0 | 0 | 0 | 0 | | |
| 0 | 0 | 0 | 0 | | |
| 0 | 0 | 0 | 0 | | |
| 0 | 0 | 0 | 0 | | |
| 0 | 0 | 0 | 0 | | |
| 0 | 0 | 0 | 0 | | |
| 0 | 0 | 0 | 0 | | |
| 0 | 0 | 0 | 0 | | |
| 0 | 0 | 0 | 0 | | |
| 0 | 0 | 0 | 0 | | |
| 0 | 0 | 0 | 0 | | |
| 0 | 0 | 0 | 0 | | |
| 0 | 0 | 0 | 0 | | |
| 0 | 0 | 0 | 0 | | |
| 0 | 0 | 0 | 0 | | |
| 0 | 0 | 0 | 0 | | |
| 0 | 0 | 0 | 0 | | |
| 0 | 0 | 0 | 0 | | |
| 0 | 0 | 0 | 0 | | |
| 0 | 0 | 0 | 0 | | |
| 0 | 0 | 0 | 0 | | |
| 0 | 0 | 0 | 0 | | |
| 0 | 0 | 0 | 0 | | |
| 0 | 0 | 1 | 0 | 2 | Super strength caffeine pills,Cobalamin/ vitamin B12 |
| 0 | 0 | 0 | 0 | | |
| 0 | 0 | 0 | 0 | | Other drug or substance used |
| 0 | 0 | 0 | 0 | | |

| | | | | | |
|---|---|---|---|---|---|
| 0 | 0 | 1 | 1 | 2 | Modafinil,Super strength caffeine pills,Cobalamin/ vitamin B12 |
| 0 | 0 | 0 | 0 | | |
| 0 | 0 | 1 | 0 | 2 | Super strength caffeine pills,Alpha Brain/vitamin B6 |
| 0 | 0 | 0 | 0 | | |
| 0 | 0 | 0 | 0 | | |
| 0 | 0 | 0 | 0 | | |
| 0 | 0 | 1 | 0 | 1 | Super strength caffeine pills |
| 0 | 0 | 0 | 0 | | |
| 0 | 0 | 1 | 0 | 1 | Super strength caffeine pills |
| 0 | 0 | 0 | 0 | 1 | Alpha Brain/vitamin B6 |
| 0 | 0 | 0 | 0 | | |
| 0 | 0 | 1 | 0 | 2 | Cobalamin/ vitamin B12 |
| 0 | 0 | 1 | 0 | 2 | Cobalamin/ vitamin B12,Vinpocetine |

| | | | | | |
|---|---|---|---|---|---|
| 0 | 0 | 0 | 0 | 2 | Alpha Brain/vitamin B6,Cobalamin/ vitamin B12 |
| 0 | 0 | 0 | 0 | | |
| 0 | 0 | 0 | 0 | | |
| 0 | 0 | 0 | 0 | | |
| 0 | 0 | 1 | 0 | 2 | Super strength caffeine pills,Vinpocetine |
| 0 | 0 | 0 | 1 | 1 | Modafinil |
| 0 | 0 | 0 | 0 | | |
| 0 | 0 | 0 | 1 | 1 | Modafinil |
| 0 | 0 | 0 | 0 | | |
| 0 | 0 | 0 | 0 | 1 | Cobalamin/ vitamin B12 |
| 0 | 0 | 0 | 0 | | |
| 0 | 0 | 0 | 1 | 1 | Modafinil |
| 0 | 0 | 0 | 1 | 1 | Modafinil |

| | | | | | |
|---|---|---|---|---|---|
| 0 | 0 | 1 | 0 | 2 | Super strength caffeine pills,Alpha Brain/vitamin B6 |
| 0 | 0 | 1 | 0 | 1 | Super strength caffeine pills |
| | | o no<br>1 yes | 1 single<br>2 multiple | | |
| | | | | | 5 Arab |
| | | | | | 6 asian (india, pakistan, srilanka, Philip)<br>7 other<br>8 NA |

| nationality | Q5 | Q5_6_TEXT | degree | Q6 | year |
|---|---|---|---|---|---|
| | Nationality - Selected Choice | Nationality - Other, (please specify) - Text | | What degree are you studying? | |
| 6 | Asian | Indian | 1 | Medicine | 3 |
| 5 | Arab Nationality | | 4 | Nursing | 3 |
| 5 | Arab Nationality | | 3 | Dentistry | 4 |
| 4 | UAE | | 3 | Dentistry | 4 |
| 8 | | | 6 | | 7 |
| 8 | | | 6 | | 7 |
| 6 | Asian | Indian | 1 | Medicine | 3 |
| 8 | | | 6 | | 7 |
| 8 | | | 6 | | 7 |
| 5 | Arab Nationality | | 1 | Medicine | 4 |
| 8 | | | 6 | | 7 |
| 8 | | | 6 | | 7 |
| 7 | Other, (please specify) | Tanzania | 1 | Medicine | 1 |
| 8 | | | 6 | | 7 |
| 8 | | | 6 | | 7 |
| 8 | | | 6 | | 7 |
| 8 | | | 6 | | 7 |
| 8 | | | 6 | | 7 |
| 4 | UAE | | 1 | Medicine | 4 |
| 5 | Arab Nationality | | 1 | Medicine | 4 |
| 4 | UAE | | 4 | Nursing | 1 |
| 5 | Arab Nationality | | 3 | Dentistry | 1 |
| 5 | Arab Nationality | | 2 | Pharmacy | 3 |
| 5 | Arab Nationality | | 3 | Dentistry | 2 |
| 4 | UAE | Ras Al Khaimah | 1 | Medicine | 3 |
| 8 | | | 6 | | 7 |
| 6 | Asian | Indian | 1 | Medicine | 4 |
| 6 | Asian | Pakistani | 4 | Nursing | 1 |
| 4 | UAE | Dubai | 6 | | 7 |
| 8 | | | 6 | | 7 |
| 5 | Arab Nationality | | 1 | Medicine | 2 |
| 8 | | | 6 | | 7 |
| 8 | | | 6 | | 7 |
| 8 | | | 6 | | 7 |
| 5 | Arab Nationality | | 1 | Medicine | 1 |
| 5 | Arab Nationality | | 4 | Nursing | 4 |
| 6 | Asian | indain | 1 | Medicine | 4 |
| 8 | | | 6 | | 7 |
| 8 | | | 6 | | 7 |
| 8 | | | 6 | | 7 |
| 6 | Asian | Indian | 1 | Medicine | 3 |
| 8 | | | 6 | | 7 |
| 1 | UK | | 2 | Pharmacy | 1 |
| 8 | | | 6 | | 7 |
| 1 | UK | | 2 | Pharmacy | 3 |
| 8 | | | 6 | | 7 |
| 8 | | | 6 | | 7 |
| 1 | UK | | 2 | Pharmacy | 1 |
| 8 | | | 6 | | 7 |
| 4 | UAE | | 2 | Pharmacy | 2 |

| | | | | | |
|---|---|---|---|---|---|
| 8 | | | 6 | | 7 |
| 8 | | | 6 | | 7 |
| 1 | UK | | 2 | Pharmacy | 2 |
| 8 | | | 6 | | 7 |
| 1 | UK | | 2 | Pharmacy | 1 |
| 8 | | | 6 | | 7 |
| 1 | UK | | 2 | Pharmacy | 2 |
| 1 | UK | | 2 | Pharmacy | 4 |
| 8 | | | 6 | | 7 |
| 6 | Asian | Indian | 1 | Medicine | 1 |
| 6 | Asian | Indian | 1 | Medicine | 4 |
| 5 | Arab Nationality | | 3 | Dentistry | 4 |
| 8 | | | 6 | | 7 |
| 8 | | | 6 | | 7 |
| 1 | UK | | 2 | Pharmacy | 3 |
| 4 | UAE | | 1 | Medicine | 4 |
| 8 | | | 6 | | 7 |
| 5 | Arab Nationality | | 3 | Dentistry | 1 |
| 8 | | | 6 | | 7 |
| 8 | | | 6 | | 7 |
| 8 | | | 6 | | 7 |
| 4 | UAE | | 1 | Medicine | 2 |
| 8 | | | 6 | | 7 |
| 8 | | | 6 | | 7 |
| 8 | | | 6 | | 7 |
| 8 | | | 6 | | 7 |
| 1 | UK | | 1 | Medicine | 3 |
| 4 | UAE | | 1 | Medicine | 4 |
| 4 | UAE | | 1 | Medicine | 4 |
| 4 | UAE | | 1 | Medicine | 2 |
| 4 | UAE | | 1 | Medicine | 4 |
| 8 | | | 6 | | 7 |
| 4 | UAE | | 1 | Medicine | 3 |
| 4 | UAE | | 1 | Medicine | 4 |
| 4 | UAE | | 1 | Medicine | 5 |
| 4 | UAE | | 1 | Medicine | 4 |
| 4 | UAE | | 1 | Medicine | 4 |
| 5 | Arab Nationality | | 1 | Medicine | 3 |
| 5 | Arab Nationality | | 2 | Pharmacy | 3 |
| 4 | UAE | | 2 | Pharmacy | 3 |
| 4 | UAE | | 1 | Medicine | 3 |
| 4 | UAE | | 1 | Medicine | 3 |
| 4 | UAE | | 6 | | 7 |
| 5 | Arab Nationality | | 2 | Pharmacy | 4 |
| 4 | UAE | | 1 | Medicine | 5 |
| 4 | UAE | | 1 | Medicine | 3 |
| 4 | UAE | | 1 | Medicine | 3 |
| 4 | UAE | | 1 | Medicine | 2 |
| 8 | | | 6 | | 7 |
| 4 | UAE | | 1 | Medicine | 4 |
| 5 | Arab Nationality | | 2 | Pharmacy | 3 |
| 5 | Arab Nationality | | 1 | Medicine | 4 |

| | | | | | |
|---|---|---|---|---|---|
| 8 | | | 6 | | 7 |
| 8 | | | 6 | | 7 |
| 4 | UAE | | 2 | Pharmacy | 3 |
| 4 | UAE | | 1 | Medicine | 5 |
| 8 | | | 6 | | 7 |
| 4 | UAE | | 1 | Medicine | 5 |
| 4 | UAE | | 1 | Medicine | 4 |
| 4 | UAE | | 1 | Medicine | 3 |
| 4 | UAE | | 1 | Medicine | 4 |
| 5 | Arab Nationality | | 1 | Medicine | 5 |
| 8 | | | 6 | | 7 |
| 8 | | | 6 | | 7 |
| 4 | UAE | | 1 | Medicine | 4 |
| 8 | | | 6 | | 7 |
| 8 | | | 6 | | 7 |
| 4 | UAE | | 1 | Medicine | 5 |
| 8 | | | 6 | | 7 |
| 8 | | | 6 | | 7 |
| 8 | | | 6 | | 7 |
| 8 | | | 6 | | 7 |
| 5 | Arab Nationality | | 2 | Pharmacy | 4 |
| 8 | | | 6 | | 7 |
| 5 | Arab Nationality | | 1 | Medicine | 5 |
| 8 | | | 6 | | 7 |
| 8 | | | 6 | | 7 |
| 8 | | | 6 | | 7 |
| 5 | Arab Nationality | | 2 | Pharmacy | 3 |
| 5 | Arab Nationality | | 2 | Pharmacy | 4 |
| 8 | | | 6 | | 7 |
| 8 | | | 6 | | 7 |
| 8 | | | 6 | | 7 |
| 8 | | | 6 | | 7 |
| 8 | | | 6 | | 7 |
| 6 | Asian | India | 1 | Medicine | 3 |
| 8 | | | 6 | | 7 |
| 8 | | | 6 | | 7 |
| 4 | UAE | | 2 | Pharmacy | 3 |
| 4 | UAE | | 1 | Medicine | 4 |
| 8 | | | 6 | | 7 |
| 8 | | | 6 | | 7 |
| 4 | UAE | | 3 | Dentistry | 4 |
| 4 | UAE | | 3 | Dentistry | 3 |
| 4 | UAE | | 2 | Pharmacy | 2 |
| 8 | | | 6 | | 7 |
| 8 | | | 6 | | 7 |
| 8 | | | 6 | | 7 |
| 8 | | | 6 | | 7 |
| 8 | | | 6 | | 7 |
| 8 | | | 6 | | 7 |
| 8 | | | 6 | | 7 |
| 8 | | | 6 | | 7 |
| 1 | UK | | 5 | Engineering | 3 |

| | | | | |
|---|---|---|---|---|
| 5 | Arab Nationality | 1 | Medicine | 5 |
| 8 | | 6 | | 7 |
| 8 | | 6 | | 7 |
| 8 | | 6 | | 7 |
| 4 | UAE | 1 | Medicine | 5 |
| 4 | UAE | 1 | Medicine | 4 |
| 8 | | 6 | | 7 |
| 8 | | 6 | | 7 |
| 8 | | 6 | | 7 |
| 8 | | 6 | | 7 |
| 8 | | 6 | | 7 |
| 5 | Arab Nationality | 1 | Medicine | 5 |
| 8 | | 6 | | 7 |
| 8 | | 6 | | 7 |
| 8 | | 6 | | 7 |
| 5 | Arab Nationality | 5 | Engineering | 3 |
| 8 | | 6 | | 7 |
| 8 | | 6 | | 7 |
| 8 | | 6 | | 7 |
| 8 | | 6 | | 7 |
| 8 | | 6 | | 7 |
| 8 | | 6 | | 7 |
| 8 | | 6 | | 7 |
| 5 | Arab Nationality | 6 | Other, (please specify) | 6 |
| 8 | | 6 | | 7 |
| 8 | | 6 | | 7 |
| 8 | | 6 | | 7 |
| 8 | | 6 | | 7 |
| 8 | | 6 | | 7 |
| 8 | | 6 | | 7 |
| 8 | | 6 | | 7 |
| 4 | UAE | 3 | Dentistry | 4 |
| 8 | | 6 | | 7 |
| 4 | UAE | 2 | Pharmacy | 3 |
| 8 | | 6 | | 7 |
| 4 | UAE | 1 | Medicine | 4 |
| 8 | | 6 | | 7 |
| 5 | Arab Nationality | 3 | Dentistry | 4 |
| 8 | | 6 | | 7 |
| 4 | UAE | 1 | Medicine | 4 |
| 8 | | 6 | | 7 |
| 8 | | 6 | | 7 |
| 8 | | 6 | | 7 |
| 8 | | 6 | | 7 |
| 5 | Arab Nationality | 3 | Dentistry | 2 |
| 8 | | 6 | | 7 |
| 8 | | 6 | | 7 |
| 8 | | 6 | | 7 |
| 8 | | 6 | | 7 |
| 8 | | 6 | | 7 |
| 8 | | 6 | | 7 |
| 8 | | 6 | | 7 |

| | | | | | |
|---|---|---|---|---|---|
| 6 | Asian | Indian | 6 | Other, (please specify) | 3 |
| 8 | | | 6 | | 7 |
| 8 | | | 1 | Medicine | 4 |
| 6 | Asian | Indian | 6 | Other, (please specify) | 3 |
| 6 | Asian | Indian | 3 | Dentistry | 3 |
| 8 | | | 6 | | 7 |
| 5 | Arab Nationality | | 1 | Medicine | 4 |
| 7 | Other, (please specify) | Tanzanian | 6 | Other, (please specify) | 2 |
| 8 | | | 6 | | 7 |
| 8 | | | 1 | Medicine | 3 |
| 8 | | | 6 | | 7 |
| 6 | Asian | Bangladesh | 2 | Pharmacy | 2 |
| 3 | USA | | 1 | Medicine | 2 |
| 8 | | | 6 | | 7 |
| 1 | UK | | 3 | Dentistry | 4 |
| 7 | Other, (please specify) | South African | 1 | Medicine | 1 |
| 8 | | | 6 | | 7 |
| 5 | Arab Nationality | | 6 | Other, (please specify) | 3 |
| 8 | | | 6 | | 7 |
| 8 | | | 6 | | 7 |
| 8 | | | 6 | | 7 |
| 7 | Other, (please specify) | African | 1 | Medicine | 4 |
| 6 | Asian | indian | 2 | Pharmacy | 2 |
| 6 | Asian | Indian | 6 | Other, (please specify) | 1 |
| 8 | | | 6 | | 7 |
| 8 | | | 6 | | 7 |
| 7 | Other, (please specify) | Nigerian | 1 | Medicine | 4 |
| 6 | Asian | Indian | 3 | Dentistry | 3 |
| 8 | | | 6 | | 7 |
| 8 | | | 6 | | 7 |
| 5 | Arab Nationality | | 3 | Dentistry | 2 |
| 6 | Asian | indian | 1 | Medicine | 2 |
| 5 | Arab Nationality | | 1 | Medicine | 6 |
| 8 | | | 6 | | 7 |
| 4 | UAE | | 4 | Nursing | 1 |
| 8 | | | 6 | | 7 |
| 8 | | | 6 | | 7 |
| 8 | | | 6 | | 7 |
| 8 | | | 6 | | 7 |
| 8 | | | 6 | | 7 |
| 5 | Arab Nationality | | 6 | Other, (please specify) | 1 |
| 6 | Asian | Indian | 6 | Other, (please specify) | 1 |
| 6 | Asian | India | 6 | Other, (please specify) | 3 |
| 4 | UAE | | 2 | Pharmacy | 1 |
| 8 | | | 6 | | 7 |
| 8 | | | 6 | | 7 |
| 6 | Asian | Indian | 2 | Pharmacy | 2 |
| 8 | | | 6 | | 7 |
| 8 | | | 6 | | 7 |
| 4 | UAE | | 3 | Dentistry | 1 |
| 8 | | | 6 | | 7 |
| 1 | UK | | 3 | Dentistry | 2 |

| | | | | | |
|---|---|---|---|---|---|
| 8 | | | 6 | | 7 |
| 8 | | | 6 | | 7 |
| 7 | Other, (please specify) | Nigerian | 6 | Other, (please specify) | 3 |
| 8 | | | 6 | | 7 |
| 4 | UAE | | 1 | Medicine | 1 |
| 8 | | | 6 | | 7 |
| 8 | | | 6 | | 7 |
| 6 | Asian | Pakistani | 1 | Medicine | 5 |
| 4 | UAE | | 3 | Dentistry | 5 |
| 6 | Asian | srilanka | 1 | Medicine | 1 |
| 6 | Asian | Indian | 1 | Medicine | 1 |
| 5 | Arab Nationality | | 6 | Other, (please specify) | 2 |
| 5 | Arab Nationality | | 1 | Medicine | 4 |
| 8 | | | 6 | | 7 |
| 7 | Other, (please specify) | Nigeria | 2 | Pharmacy | 1 |
| 8 | | | 6 | | 7 |
| 5 | Arab Nationality | | 1 | Medicine | 3 |
| 8 | | | 6 | | 7 |
| 5 | Arab Nationality | | 2 | Pharmacy | 5 |
| 8 | | | 6 | | 7 |
| 4 | UAE | | 6 | Other, (please specify) | 4 |
| 8 | | | 6 | | 7 |
| 6 | Asian | Indian | 2 | Pharmacy | 2 |
| 8 | | | 6 | | 7 |
| 8 | | | 6 | | 7 |
| 6 | Asian | India | 1 | Medicine | 4 |
| 6 | Asian | India | 4 | Nursing | 2 |
| 6 | Asian | Indian | 1 | Medicine | 5 |
| 8 | | | 6 | | 7 |
| 8 | | | 6 | | 7 |
| 8 | | | 6 | | 7 |
| 8 | | | 6 | | 7 |
| 8 | | | 6 | | 7 |
| 8 | | | 6 | | 7 |
| 7 | Other, (please specify) | Nigerian | 3 | Dentistry | 1 |
| 8 | | | 6 | | 7 |
| 7 | Other, (please specify) | | 2 | Pharmacy | 4 |
| 8 | | | 6 | | 7 |
| 6 | Asian | Indian | 2 | Pharmacy | 5 |
| 4 | UAE | Abu Dhabi | 6 | | 7 |
| 5 | Arab Nationality | | 3 | Dentistry | 3 |
| 5 | Arab Nationality | | 6 | Other, (please specify) | 1 |
| 6 | Asian | Philippines | 1 | Medicine | 3 |
| 4 | UAE | | 1 | Medicine | 3 |
| 8 | | | 6 | | 7 |
| 6 | Asian | INDIAN | 1 | Medicine | 1 |
| 8 | | | 6 | | 7 |
| 6 | Asian | Pakistani | 2 | Pharmacy | 3 |
| 5 | Arab Nationality | | 6 | Other, (please specify) | 4 |
| 6 | Asian | Pakistani | 1 | Medicine | 2 |
| 8 | | | 6 | | 7 |
| 8 | | | 6 | | 7 |

| | | | | | |
|---|---|---|---|---|---|
| 8 | | | 6 | | 7 |
| 5 | Arab Nationality | | 1 | Medicine | 2 |
| 8 | | | 6 | | 7 |
| 6 | Asian | Indian | 6 | | 7 |
| 7 | Other, (please specify) | | 3 | Dentistry | 1 |
| 7 | Other, (please specify) | Nigerian | 6 | Other, (please specify) | 3 |
| 8 | | | 6 | | 7 |
| 8 | | | 6 | | 7 |
| 8 | | | 6 | | 7 |
| 8 | | | 6 | | 7 |
| 8 | | | 6 | | 7 |
| 8 | | | 6 | | 7 |
| 8 | | | 6 | | 7 |
| 6 | Asian | Indian | 1 | Medicine | 3 |
| 7 | Other, (please specify) | | 6 | | 7 |
| 7 | Other, (please specify) | Nigeria | 4 | Nursing | 1 |
| 5 | Arab Nationality | | 1 | Medicine | 2 |
| 7 | Other, (please specify) | MI | 6 | | 7 |
| 8 | | | 6 | | 7 |
| 8 | | | 6 | | 7 |
| 6 | Asian | Indian | 1 | Medicine | 1 |
| 8 | | | 6 | | 7 |
| 8 | | | 6 | | 7 |
| 8 | | | 6 | | 7 |
| 8 | | | 6 | | 7 |
| 6 | Asian | Indian | 1 | Medicine | 3 |
| 6 | Asian | India | 1 | Medicine | 2 |
| 6 | Asian | pakistan | 6 | Other, (please specify) | 2 |
| 6 | Asian | Indian | 6 | Other, (please specify) | 1 |
| 5 | Arab Nationality | | 1 | Medicine | 3 |
| 7 | Other, (please specify) | chinese and syrian | 1 | Medicine | 3 |
| 5 | Arab Nationality | | 6 | Other, (please specify) | 2 |
| 8 | | | 6 | | 7 |
| 5 | Arab Nationality | | 3 | Dentistry | 4 |
| 8 | | | 6 | | 7 |
| 6 | Asian | INDIAN | 2 | Pharmacy | 2 |
| 2 | EU | | 3 | Dentistry | 1 |
| 7 | Other, (please specify) | | 6 | | 7 |
| 8 | | | 6 | | 7 |
| 8 | | | 6 | | 7 |
| 5 | Arab Nationality | | 3 | Dentistry | 1 |
| 7 | Other, (please specify) | ZImbabwe | 1 | Medicine | 2 |
| 5 | Arab Nationality | | 1 | Medicine | 1 |
| 6 | Asian | Indian | 1 | Medicine | 3 |
| 8 | | | 6 | | 7 |
| 6 | Asian | Indian | 6 | Other, (please specify) | 1 |
| 8 | | | 6 | | 7 |
| 8 | | | 1 | Medicine | 5 |
| 8 | | | 6 | | 7 |
| 6 | Asian | pakistan | 6 | Other, (please specify) | 1 |
| 5 | Arab Nationality | | 1 | Medicine | 3 |
| 8 | | | 6 | | 7 |

| | | | | | |
|---|---|---|---|---|---|
| 8 | | | 6 | | 7 |
| 8 | | | 1 | Medicine | 1 |
| 8 | | | 6 | | 7 |
| 6 | Asian | Indian | 1 | Medicine | 4 |
| 8 | | | 6 | | 7 |
| 8 | | | 6 | | 7 |
| 7 | Other, (please specify) | | 1 | Medicine | 5 |
| 8 | | | 6 | | 7 |
| 8 | | | 6 | | 7 |
| 8 | | | 6 | | 7 |
| 6 | Asian | Iran | 6 | Other, (please specify) | 1 |
| 8 | | | 6 | | 7 |
| 3 | USA | | 1 | Medicine | 2 |
| 6 | Asian | Indian | 6 | Other, (please specify) | 4 |
| 8 | | | 6 | | 7 |
| 8 | | | 6 | | 7 |
| 8 | | | 6 | | 7 |
| 8 | | | 6 | | 7 |
| 8 | | | 6 | | 7 |
| 4 | UAE | إمارة دبيّ | 6 | | 7 |
| 8 | | | 6 | | 7 |
| 8 | | | 6 | | 7 |
| 8 | | | 6 | | 7 |
| 4 | UAE | | 5 | Engineering | 3 |
| 4 | UAE | | 5 | Engineering | 2 |
| 5 | Arab Nationality | | 5 | Engineering | 4 |
| 8 | | | 6 | | 7 |
| 8 | | | 6 | | 7 |
| 8 | | | 6 | | 7 |
| 8 | | | 6 | | 7 |
| 8 | | | 6 | | 7 |
| 8 | | | 6 | | 7 |
| 6 | Asian | India | 6 | Other, (please specify) | 2 |
| 6 | Asian | India | 6 | Other, (please specify) | 4 |
| 8 | | | 6 | | 7 |
| 6 | Asian | Persian | 3 | Dentistry | 3 |
| 8 | | | 6 | | 7 |
| 6 | Asian | Indian | 2 | Pharmacy | 1 |
| 8 | | | 6 | | 7 |
| 8 | | | 6 | | 7 |
| 8 | | | 6 | | 7 |
| 4 | UAE | | 5 | Engineering | 3 |
| 4 | UAE | | 5 | Engineering | 4 |
| 5 | Arab Nationality | | 5 | Engineering | 3 |
| 5 | Arab Nationality | | 3 | Dentistry | 3 |
| 5 | Arab Nationality | | 5 | Engineering | 4 |
| 6 | Asian | Indian | 1 | Medicine | 4 |
| 4 | UAE | | 5 | Engineering | 3 |
| 4 | UAE | | 3 | Dentistry | 4 |
| 5 | Arab Nationality | | 5 | Engineering | 4 |
| 5 | Arab Nationality | | 3 | Dentistry | 3 |
| 6 | Asian | Indian | 6 | Other, (please specify) | 2 |

| | | | | | |
|---|---|---|---|---|---|
| 6 | Asian | Indian | 6 | Other, (please specify) | 2 |
| 8 | | | 6 | | 7 |
| 8 | | | 6 | | 7 |
| 6 | Asian | Iran | 1 | Medicine | 4 |
| 8 | | | 6 | | 7 |
| 5 | Arab Nationality | | 3 | Dentistry | 4 |
| 8 | | | 6 | | 7 |
| 8 | | | 6 | | 7 |
| 8 | | | 6 | | 7 |
| 6 | Asian | INDIAN | 1 | Medicine | 3 |
| 6 | Asian | Indian | 1 | Medicine | 3 |
| 8 | | | 6 | | 7 |
| 8 | | | 6 | | 7 |
| 5 | Arab Nationality | | 6 | Other, (please specify) | 3 |
| 8 | | | 6 | | 7 |
| 8 | | | 6 | | 7 |
| 5 | Arab Nationality | | 6 | Other, (please specify) | 1 |
| 8 | | | 6 | | 7 |
| 5 | Arab Nationality | | 3 | Dentistry | 3 |
| 8 | | | 6 | | 7 |
| 5 | Arab Nationality | | 5 | Engineering | 4 |
| 6 | Asian | Pakistan | 6 | Other, (please specify) | 2 |
| 8 | | | 6 | | 7 |
| 5 | Arab Nationality | | 6 | Other, (please specify) | 4 |
| 4 | UAE | | 3 | Dentistry | 3 |
| 8 | | | 6 | | 7 |
| 8 | | | 6 | | 7 |
| 8 | | | 6 | | 7 |
| 5 | Arab Nationality | | 1 | Medicine | 3 |
| 8 | | | 6 | | 7 |
| 8 | | | 6 | | 7 |
| 8 | | | 6 | | 7 |
| 8 | | | 6 | | 7 |
| 6 | Asian | india | 3 | Dentistry | 4 |
| 4 | UAE | | 1 | Medicine | 4 |
| 5 | Arab Nationality | | 5 | Engineering | 3 |
| 5 | Arab Nationality | | 5 | Engineering | 3 |
| 8 | | | 6 | | 7 |
| 8 | | | 6 | | 7 |
| 8 | | | 6 | | 7 |
| 8 | | | 6 | | 7 |
| 8 | | | 6 | | 7 |
| 8 | | | 6 | | 7 |
| 8 | | | 6 | | 7 |
| 6 | Asian | Pakistan | 5 | Engineering | 3 |
| 8 | | | 6 | | 7 |
| 8 | | | 6 | | 7 |
| 8 | | | 6 | | 7 |
| 5 | Arab Nationality | | 1 | Medicine | 5 |
| 4 | UAE | | 1 | Medicine | 4 |
| 8 | | | 6 | | 7 |
| 8 | | | 6 | | 7 |

| | | | | | |
|---|---|---|---|---|---|
| 8 | | | 6 | | 7 |
| 8 | | | 6 | | 7 |
| 8 | | | 6 | | 7 |
| 8 | | | 6 | | 7 |
| 8 | | | 6 | | 7 |
| 8 | | | 6 | | 7 |
| 8 | | | 6 | | 7 |
| 8 | | | 6 | | 7 |
| 8 | | | 6 | | 7 |
| 8 | | | 6 | | 7 |
| 8 | | | 6 | | 7 |
| 8 | | | 6 | | 7 |
| 8 | | | 6 | | 7 |
| 8 | | | 6 | | 7 |
| 8 | | | 6 | | 7 |
| 8 | | | 6 | | 7 |
| 8 | | | 6 | | 7 |
| 8 | | | 6 | | 7 |
| 8 | | | 6 | | 7 |
| 7 | Other, (please specify) | | 6 | 4 | 2 |
| 7 | Other, (please specify) | Indian | 6 | 6 | 2 |
| 8 | | | 6 | | 7 |
| 7 | Other, (please specify) | India | 6 | 6 | 4 |
| 8 | | | 6 | 6 | 7 |
| 4 | UAE | | 2 | Pharmacy | 4 |
| 8 | | | 6 | | 7 |
| 7 | Other, (please specify) | Palastine | 6 | 4 | 2 |
| 8 | | | 6 | | 7 |

| | | | | | |
|---|---|---|---|---|---|
| 4 | UAE | 6 | | 5 | 4 |
| 8 | | 6 | | | 7 |
| | | | | | |
| 4 | UAE | 1 | Medicine | | 3 |
| 8 | | 6 | | | 7 |
| 8 | | 6 | | | 7 |
| 8 | | 6 | | | 7 |
| 5 | Arab Nationality | 6 | | 5 | 4 |
| 8 | | 6 | | | 7 |
| 4 | UAE | 1 | Medicine | | 2 |
| 5 | Arab Nationality | 2 | Pharmacy | | 4 |
| 8 | | 6 | | | 7 |
| 5 | Arab Nationality | 6 | | 3 | 4 |
| 4 | UAE | 6 | | 5 | 3 |

| | | | | | |
|---|---|---|---|---|---|
| 1 | UK | 2 | Pharmacy | | 2 |
| 8 | | 6 | | | 7 |
| 8 | | 6 | | | 7 |
| 8 | | 6 | | | 7 |
| 4 | UAE | 1 | Medicine | | 5 |
| 5 | Arab Nationality | 1 | Medicine | | 3 |
| 8 | | 6 | | | 7 |
| 5 | Arab Nationality | 2 | Pharmacy | | 4 |
| 8 | | 6 | | | 7 |
| 5 | Arab Nationality | 1 | Medicine | | 4 |
| 8 | | 6 | | | 7 |
| 1 | UK | 6 | | 6 | 3 |
| 5 | Arab Nationality | 2 | Pharmacy | | 4 |

| | | | | | | |
|---|---|---|---|---|---|---|
| 7 | Other, (please specify) | Indian | 1 | Medicine | | 4 |
| 5 | Arab Nationality | | 6 | | 5 | 4 |

1 UK
2 EU
3 USA
4 UAE

1 medicien
2 pharmacy
3 dentistry
4 nursing
5 engineering
6 other/not specified

1 first
2 second
7 not specified

| Q7 | why | Q8 | positiveEffects | Q9 | Q9_9_TEXT | when | Q10 |
|---|---|---|---|---|---|---|---|
| Year of Education | | Please specify the reason(s) for use? (select all that apply) - Selected Choice | | [illegible] | [illegible] | | When was the time of your most recent consumption? |
| Third year | 6 | | 3 | | | 5 | |
| Third year | 6 | | 3 | | | 5 | |
| Fourth year | 1 | To improve concentration | 1 | Yes. If yes, please specify | Reduces stress and helps to stay focus | 1 | During exam weeks |
| Fourth year | 6 | | 3 | | | 5 | |
| | 6 | | 3 | | | 5 | |
| | 6 | | 3 | | | 5 | |
| Third year | 6 | | 3 | | | 5 | |
| | 6 | | 3 | | | 5 | |
| | 6 | | 3 | | | 5 | |
| Fourth year | 6 | | 3 | | | 5 | |
| | 6 | | 3 | | | 5 | |
| | 6 | | 3 | | | 5 | |
| First year | 6 | | 3 | | | 5 | |
| | 6 | | 3 | | | 5 | |
| | 6 | | 3 | | | 5 | |
| | 6 | | 3 | | | 5 | |
| | 6 | | 3 | | | 5 | |
| | 6 | | 3 | | | 5 | |
| Fourth year | 6 | | 3 | | | 5 | |
| Fourth year | 2 | To improve your memory | 2 | No | | 1 | During exam weeks |
| First year | 6 | | 3 | | | 5 | |
| First year | 6 | | 3 | | | 5 | |
| Third year | 6 | | 3 | | | 5 | |
| Second year | 6 | | 3 | | | 5 | |
| Third year | 5 | Other, please specify | 1 | Yes. If yes, please specify | none | 5 | |
| | 6 | | 3 | | | 5 | |
| Fourth year | 1 | To improve concentration | 1 | Yes. If yes, please specify | Increased alertness, concentration | 1 | During exam weeks |
| First year | 6 | | 2 | No | | 5 | |
| | 6 | | 3 | | | 5 | |
| | 6 | | 3 | | | 5 | |
| Second year | 1 | To improve concentration | 1 | Yes. If yes, please specify | | 1 | During exam weeks |
| | 6 | | 3 | | | 5 | |
| | 6 | | 3 | | | 5 | |
| | 6 | | 3 | | | 5 | |
| First year | 2 | To improve your memory | 2 | No | | 3 | During studying |
| Fourth year | 6 | | 3 | | | 5 | |
| Fourth year | 6 | | 3 | | | 5 | |
| | 6 | | 3 | | | 5 | |
| | 6 | | 3 | | | 5 | |
| | 6 | | 3 | | | 5 | |
| Third year | 6 | | 3 | | | 5 | |
| | 6 | | 3 | | | 5 | |
| First year | 6 | | 3 | | | 5 | |
| | 6 | | 3 | | | 5 | |
| Third year | 1 | To improve concentration | 2 | No | | 1 | During exam weeks |
| | 6 | | 3 | | | 5 | |
| | 6 | | 3 | | | 5 | |
| First year | 4 | To improve academic performance | 1 | Yes. If yes, please specify | | 3 | During studying |
| | 6 | | 3 | | | 5 | |
| Second year | 1 | To improve concentration | 2 | No | | 1 | During exam weeks |

| | | | | | | | |
|---|---|---|---|---|---|---|---|
| | 6 | | 3 | | | 5 | |
| | 6 | | 3 | | | 5 | |
| Second year | 6 | | 3 | | | 5 | |
| | 6 | | 3 | | | 5 | |
| First year | 1 | To improve concentration | 2 | No | | 1 | During exam weeks |
| | 6 | | 3 | | | 5 | |
| Second year | 6 | | 2 | No | | 5 | |
| Fourth year | 5 | Other, please specify | 2 | No | | 3 | During studying |
| | 6 | | 3 | | | 5 | |
| First year | 2 | To improve your memory | 2 | No | | 1 | During exam weeks |
| Fourth year | 2 | To improve your memory | 1 | Yes. If yes, please specify | | 1 | During exam weeks |
| Fourth year | 4 | To improve academic performance | 2 | No | | 2 | During course work deadlines |
| | 6 | | 3 | | | 5 | |
| | 6 | | 3 | | | 5 | |
| Third year | 1 | To improve concentration | 1 | Yes. If yes, please specify | alertness | 1 | During exam weeks |
| Fourth year | 1 | To improve concentration | 2 | No | | 1 | During exam weeks |
| | 6 | | 3 | | | 5 | |
| First year | 6 | | 3 | | | 5 | |
| | 6 | | 3 | | | 5 | |
| | 6 | | 3 | | | 5 | |
| | 6 | | 3 | | | 5 | |
| Second year | 6 | | 3 | | | 5 | |
| | 6 | | 3 | | | 5 | |
| | 6 | | 3 | | | 5 | |
| | 6 | | 3 | | | 5 | |
| | 6 | | 3 | | | 5 | |
| Third year | 6 | | 3 | | | 5 | |
| Fourth year | 1 | To improve concentration | 1 | Yes. If yes, please specify | | 1 | During exam weeks |
| Fourth year | 1 | To improve concentration | 2 | No | | 1 | During exam weeks |
| Second year | 3 | To increase alertness | 1 | Yes. If yes, please specify | Alertness | 1 | During exam weeks |
| Fourth year | 1 | To improve concentration | 1 | Yes. If yes, please specify | alretness | 1 | During exam weeks |
| | 6 | | 3 | | | 5 | |
| Third year | 1 | To improve concentration | 2 | No | | 1 | During exam weeks |
| Fourth year | 3 | To increase alertness | 1 | Yes. If yes, please specify | alretness | 1 | During exam weeks |
| Fifth year | 3 | To increase alertness | 1 | Yes. If yes, please specify | alertness | 1 | During exam weeks |
| Fourth year | 1 | To improve concentration | 1 | Yes. If yes, please specify | alertness | 1 | During exam weeks |
| Fourth year | 2 | To improve your memory | 1 | Yes. If yes, please specify | | 1 | During exam weeks |
| Third year | 2 | To improve your memory | 1 | Yes. If yes, please specify | | 1 | During exam weeks |
| Third year | 3 | To increase alertness | 2 | No | | 1 | During exam weeks |
| Third year | 3 | To increase alertness | 1 | Yes. If yes, please specify | alertness | 1 | During exam weeks |
| Third year | 1 | To improve concentration | 1 | Yes. If yes, please specify | | 1 | During exam weeks |
| Third year | 3 | To increase alertness | 1 | Yes. If yes, please specify | alertness | 1 | During exam weeks |
| | 6 | | 3 | | | 5 | |
| Fourth year | 3 | To increase alertness | 1 | Yes. If yes, please specify | alertness | 1 | During exam weeks |
| Fifth year | 2 | To improve your memory | 2 | No | | 1 | During exam weeks |
| Third year | 1 | To improve concentration | 1 | Yes. If yes, please specify | | 1 | During exam weeks |
| Third year | 1 | To improve concentration | 1 | Yes. If yes, please specify | | 1 | During exam weeks |
| Second year | 3 | To increase alertness | 1 | Yes. If yes, please specify | alertness | 1 | During exam weeks |
| | 6 | | 3 | | | 5 | |
| Fourth year | 3 | To increase alertness | 1 | Yes. If yes, please specify | alretness | 1 | During exam weeks |
| Third year | 1 | To improve concentration | 1 | Yes. If yes, please specify | | 1 | During exam weeks |
| Fourth year | 2 | To improve your memory | 1 | Yes. If yes, please specify | | 1 | During exam weeks |

| | | | | | | | |
|---|---|---|---|---|---|---|---|
| | 6 | | 3 | | | 5 | |
| | 6 | | 3 | | | 5 | |
| Third year | 3 | To increase alertness | 1 | Yes. If yes, please specify | | 1 | During exam weeks |
| Fifth year | 2 | To improve your memory | 1 | Yes. If yes, please specify | memory | 1 | During exam weeks |
| | 6 | | 3 | | | 5 | |
| Fifth year | 4 | To improve academic performance | 1 | Yes. If yes, please specify | | 1 | During exam weeks |
| Fourth year | 2 | To improve your memory | 1 | Yes. If yes, please specify | | 1 | During exam weeks |
| Third year | 2 | To improve your memory | 1 | Yes. If yes, please specify | | 1 | During exam weeks |
| Fourth year | 3 | To increase alertness | 1 | Yes. If yes, please specify | | 1 | During exam weeks |
| Fifth year | 4 | To improve academic performance | 1 | Yes. If yes, please specify | | 1 | During exam weeks |
| | 6 | | 3 | | | 5 | |
| | 6 | | 3 | | | 5 | |
| Fourth year | 4 | To improve academic performance | 1 | Yes. If yes, please specify | | 1 | During exam weeks |
| | 6 | | 3 | | | 5 | |
| | 6 | | 3 | | | 5 | |
| Fifth year | 4 | To improve academic performance | 1 | Yes. If yes, please specify | | 1 | During exam weeks |
| | 6 | | 3 | | | 5 | |
| | 6 | | 3 | | | 5 | |
| | 6 | | 3 | | | 5 | |
| | 6 | | 3 | | | 5 | |
| Fourth year | 4 | To improve academic performance | 2 | No | | 1 | During exam weeks |
| | 6 | | 3 | | | 5 | |
| Fifth year | 4 | To improve academic performance | 2 | No | | 1 | During exam weeks |
| | 6 | | 3 | | | 5 | |
| | 6 | | 3 | | | 5 | |
| | 6 | | 3 | | | 5 | |
| Third year | 3 | To increase alertness | 1 | Yes. If yes, please specify | | 1 | During exam weeks |
| Fourth year | 3 | To increase alertness | 1 | Yes. If yes, please specify | | 1 | During exam weeks |
| | 6 | | 3 | | | 5 | |
| | 6 | | 3 | | | 5 | |
| | 6 | | 3 | | | 5 | |
| | 6 | | 3 | | | 5 | |
| | 6 | | 3 | | | 5 | |
| Third year | 4 | To improve academic performance | 1 | Yes. If yes, please specify | | 1 | During exam weeks |
| | 6 | | 3 | | | 5 | |
| | 6 | | 3 | | | 5 | |
| Third year | 1 | To improve concentration | 1 | Yes. If yes, please specify | | 1 | During exam weeks |
| Fourth year | 1 | To improve concentration | 1 | Yes. If yes, please specify | | 1 | During exam weeks |
| | 6 | | 3 | | | 5 | |
| | 6 | | 3 | | | 5 | |
| Fourth year | 1 | To improve concentration | 1 | Yes. If yes, please specify | | 1 | During exam weeks |
| Third year | 4 | To improve academic performance | 1 | Yes. If yes, please specify | | 3 | During studying |
| Second year | 3 | To increase alertness | 1 | Yes. If yes, please specify | | 1 | During exam weeks |
| | 6 | | 3 | | | 5 | |
| | 6 | | 3 | | | 5 | |
| | 6 | | 3 | | | 5 | |
| | 6 | | 3 | | | 5 | |
| | 6 | | 3 | | | 5 | |
| | 6 | | 3 | | | 5 | |
| | 6 | | 3 | | | 5 | |
| | 6 | | 3 | | | 5 | |
| Third year | 3 | To increase alertness | 1 | Yes. If yes, please specify | | 1 | During exam weeks |

| | | | | | | | |
|---|---|---|---|---|---|---|---|
| Fifth year | 3 | To increase alertness | 1 | Yes. If yes, please specify | | 1 | During exam weeks |
| | 6 | | 3 | | | 5 | |
| | 6 | | 3 | | | 5 | |
| | 6 | | 3 | | | 5 | |
| Fifth year | 3 | To increase alertness | 1 | Yes. If yes, please specify | | 1 | During exam weeks |
| Fourth year | 1 | To improve concentration | 1 | Yes. If yes, please specify | | 1 | During exam weeks |
| | 6 | | 3 | | | 5 | |
| | 6 | | 3 | | | 5 | |
| | 6 | | 3 | | | 5 | |
| | 6 | | 3 | | | 5 | |
| | 6 | | 3 | | | 5 | |
| Fifth year | 1 | To improve concentration | 1 | Yes. If yes, please specify | | 5 | |
| | 6 | | 3 | | | 5 | |
| | 6 | | 3 | | | 5 | |
| | 6 | | 3 | | | 5 | |
| Third year | 4 | To improve academic performance | 1 | Yes. If yes, please specify | | 1 | During exam weeks |
| | 6 | | 3 | | | 5 | |
| | 6 | | 3 | | | 5 | |
| | 6 | | 3 | | | 5 | |
| | 6 | | 3 | | | 5 | |
| | 6 | | 3 | | | 5 | |
| | 1 | To improve concentration | 3 | | | 5 | |
| | 6 | | 3 | | | 5 | |
| Sixth year | 1 | To improve concentration | 1 | Yes. If yes, please specify | To active | 1 | During exam weeks |
| | 6 | | 3 | | | 5 | |
| | 6 | | 3 | | | 5 | |
| | 6 | | 3 | | | 5 | |
| | 6 | | 3 | | | 5 | |
| | 6 | | 3 | | | 5 | |
| | 6 | | 3 | | | 5 | |
| | 6 | | 3 | | | 5 | |
| Fourth year | 3 | To increase alertness | 1 | Yes. If yes, please specify | | 1 | During exam weeks |
| | 6 | | 3 | | | 5 | |
| Third year | 1 | To improve concentration | 1 | Yes. If yes, please specify | | 1 | During exam weeks |
| | 6 | | 3 | | | 5 | |
| Fourth year | 3 | To increase alertness | 1 | Yes. If yes, please specify | | 1 | During exam weeks |
| | 6 | | 3 | | | 5 | |
| Fourth year | 1 | To improve concentration | 1 | Yes. If yes, please specify | | 1 | During exam weeks |
| | 6 | | 3 | | | 5 | |
| Fourth year | 1 | To improve concentration | 1 | Yes. If yes, please specify | | 1 | During exam weeks |
| | 6 | | 3 | | | 5 | |
| | 6 | | 3 | | | 5 | |
| | 6 | | 3 | | | 5 | |
| | 6 | | 3 | | | 5 | |
| Second year | 6 | | 3 | | | 5 | |
| | 6 | | 3 | | | 5 | |
| | 6 | | 3 | | | 5 | |
| | 6 | | 3 | | | 5 | |
| | 6 | | 3 | | | 5 | |
| | 6 | | 3 | | | 5 | |
| | 6 | | 3 | | | 5 | |
| | 6 | | 3 | | | 5 | |

| | | | | | | | |
|---|---|---|---|---|---|---|---|
| Third year | 6 | | 3 | | | 5 | |
| | 6 | | 3 | | | 5 | |
| Fourth year | 6 | | 3 | | | 5 | |
| Third year | 6 | | 3 | | | 5 | |
| Third year | 6 | | 3 | | | 5 | |
| | 6 | | 3 | | | 5 | |
| Fourth year | 3 | To increase alertness | 2 | No | | 1 | During exam weeks |
| Second year | 5 | Other, please specify | 1 | Yes. If yes, please specify | I dont take them | 5 | |
| | 6 | | 3 | | | 5 | |
| Third year | 6 | | 3 | | | 5 | |
| | 6 | | 3 | | | 5 | |
| Second year | 3 | To increase alertness | 2 | No | | 2 | During course work deadlines |
| Second year | 1 | To improve concentration | 2 | No | | 4 | Daily basis |
| | 6 | | 3 | | | 5 | |
| Fourth year | 6 | | 3 | | | 5 | |
| First year | 6 | | 2 | No | | 5 | |
| | 6 | | 3 | | | 5 | |
| Third year | 5 | Other, please specify | 1 | Yes. If yes, please specify | not used | 5 | |
| | 6 | | 3 | | | 5 | |
| | 6 | | 3 | | | 5 | |
| | 6 | | 3 | | | 5 | |
| Fourth year | 5 | Other, please specify | 1 | Yes. If yes, please specify | .................... | 1 | During exam weeks |
| Second year | 6 | | 3 | | | 5 | |
| First year | 6 | | 3 | | | 5 | |
| | 6 | | 3 | | | 5 | |
| | 6 | | 3 | | | 5 | |
| Fourth year | 6 | | 3 | | | 5 | |
| Third year | 6 | | 3 | | | 5 | |
| | 6 | | 3 | | | 5 | |
| | 6 | | 3 | | | 5 | |
| Second year | 5 | Other, please specify | 2 | No | | 1 | During exam weeks |
| Second year | 6 | | 3 | | | 5 | |
| Sixth year | 1 | To improve concentration | 1 | Yes. If yes, please specify | Longer and sharper concentration | 2 | During course work deadlines |
| | 6 | | 3 | | | 5 | |
| First year | 5 | Other, please specify | 2 | No | | 4 | Daily basis |
| | 6 | | 3 | | | 5 | |
| | 6 | | 3 | | | 5 | |
| | 6 | | 3 | | | 5 | |
| | 6 | | 3 | | | 5 | |
| | 6 | | 3 | | | 5 | |
| First year | 6 | | 3 | | | 5 | |
| First year | 6 | | 3 | | | 5 | |
| Third year | 6 | | 3 | | | 5 | |
| First year | 6 | | 2 | No | | 5 | |
| | 6 | | 3 | | | 5 | |
| | 6 | | 3 | | | 5 | |
| Second year | 6 | | 3 | | | 5 | |
| | 6 | | 3 | | | 5 | |
| | 6 | | 3 | | | 5 | |
| First year | 4 | To improve academic performance | 2 | No | | 1 | During exam weeks |
| | 6 | | 3 | | | 5 | |
| Second year | 6 | | 3 | | | 5 | |

| | | | | | | | |
|---|---|---|---|---|---|---|---|
| | 6 | | 3 | | | 5 | |
| | 6 | | 3 | | | 5 | |
| Third year | 6 | | 3 | | | 5 | |
| | 6 | | 3 | | | 5 | |
| First year | 6 | | 3 | | | 5 | |
| | 6 | | 3 | | | 5 | |
| | 6 | | 3 | | | 5 | |
| Fifth year | 6 | | 3 | | | 5 | |
| Fifth year | 6 | | 3 | | | 5 | |
| First year | 5 | Other, please specify | 1 | Yes. If yes, please specify | alertness | 4 | Daily basis |
| First year | 6 | | 3 | | | 5 | |
| Second year | 6 | | 3 | | | 5 | |
| Fourth year | 1 | To improve concentration | 1 | Yes. If yes, please specify | Motivation | 4 | Daily basis |
| | 6 | | 3 | | | 5 | |
| First year | 6 | | 2 | No | | 5 | |
| | 6 | | 3 | | | 5 | |
| Third year | 6 | | 3 | | | 5 | |
| | 6 | | 3 | | | 5 | |
| Fifth year | 1 | To improve concentration | 2 | No | | 3 | During studying |
| | 6 | | 3 | | | 5 | |
| Fourth year | 6 | | 3 | | | 5 | |
| | 6 | | 3 | | | 5 | |
| Second year | 6 | | 3 | | | 5 | |
| | 6 | | 3 | | | 5 | |
| | 6 | | 3 | | | 5 | |
| Fourth year | 5 | Other, please specify | 1 | Yes. If yes, please specify | good mood, alertness | 1 | During exam weeks |
| Second year | 4 | To improve academic performance | 2 | No | | 5 | |
| Fifth year | 6 | | 3 | | | 5 | |
| | 6 | | 3 | | | 5 | |
| | 6 | | 3 | | | 5 | |
| | 6 | | 3 | | | 5 | |
| | 6 | | 3 | | | 5 | |
| | 6 | | 3 | | | 5 | |
| | 6 | | 3 | | | 5 | |
| First year | 6 | | 3 | | | 5 | |
| | 6 | | 3 | | | 5 | |
| Fourth year | 6 | | 3 | | | 5 | |
| | 6 | | 3 | | | 5 | |
| Fifth year | 6 | | 3 | | | 5 | |
| | 5 | Other, please specify | 3 | | | 5 | |
| Third year | 3 | To increase alertness | 2 | No | | 1 | During exam weeks |
| First year | 3 | To increase alertness | 2 | No | | 1 | During exam weeks |
| Third year | 4 | To improve academic performance | 1 | Yes. If yes, please specify | Increased alertness and focus | 1 | During exam weeks |
| Third year | 6 | | 3 | | | 5 | |
| | 6 | | 3 | | | 5 | |
| First year | 6 | | 3 | | | 5 | |
| | 6 | | 3 | | | 5 | |
| Third year | 6 | | 3 | | | 5 | |
| Fourth year | 6 | | 3 | | | 5 | |
| Second year | 6 | | 3 | | | 5 | |
| | 6 | | 3 | | | 5 | |
| | 6 | | 3 | | | 5 | |

| | | | | | | | |
|---|---|---|---|---|---|---|---|
| | 6 | | 3 | | | 5 | |
| Second year | 6 | | 3 | | | 5 | |
| | 6 | | 3 | | | 5 | |
| | 6 | | 3 | | | 5 | |
| First year | 6 | | 3 | | | 5 | |
| Third year | 6 | | 3 | | | 5 | |
| | 6 | | 3 | | | 5 | |
| | 6 | | 3 | | | 5 | |
| | 6 | | 3 | | | 5 | |
| | 6 | | 3 | | | 5 | |
| | 6 | | 3 | | | 5 | |
| | 6 | | 3 | | | 5 | |
| | 6 | | 3 | | | 5 | |
| Third year | 6 | | 3 | | | 5 | |
| | 6 | | 3 | | | 5 | |
| First year | 6 | | 3 | | | 5 | |
| Second year | 1 | To improve concentration | 2 | No | | 4 | Daily basis |
| | 5 | Other, please specify | 1 | Yes. If yes, please specify | 48331 | 5 | |
| | 6 | | 3 | | | 5 | |
| | 6 | | 3 | | | 5 | |
| First year | 6 | | 3 | | | 5 | |
| | 6 | | 3 | | | 5 | |
| | 6 | | 3 | | | 5 | |
| | 6 | | 3 | | | 5 | |
| | 6 | | 3 | | | 5 | |
| Third year | 6 | | 3 | | | 5 | |
| Second year | 1 | To improve concentration | 1 | Yes. If yes, please specify | Increased focus | 1 | During exam weeks |
| Second year | 6 | | 3 | | | 5 | |
| First year | 6 | | 2 | No | | 5 | |
| Third year | 5 | Other, please specify | 2 | No | | 3 | During studying |
| Third year | 6 | | 3 | | | 5 | |
| Second year | 6 | | 3 | | | 5 | |
| | 6 | | 3 | | | 5 | |
| Fourth year | 3 | To increase alertness | 1 | Yes. If yes, please specify | | 1 | During exam weeks |
| | 6 | | 3 | | | 5 | |
| Second year | 5 | Other, please specify | 2 | No | | 1 | During exam weeks |
| First year | 4 | To improve academic performance | 1 | Yes. If yes, please specify | | 1 | During exam weeks |
| | 6 | | 3 | | | 5 | |
| | 6 | | 3 | | | 5 | |
| | 6 | | 3 | | | 5 | |
| First year | 3 | To increase alertness | 1 | Yes. If yes, please specify | | 1 | During exam weeks |
| Second year | 6 | | 3 | | | 5 | |
| First year | 6 | | 3 | | | 5 | |
| Third year | 6 | | 3 | | | 5 | |
| | 6 | | 3 | | | 5 | |
| First year | 5 | Other, please specify | 2 | No | | 5 | |
| | 6 | | 3 | | | 5 | |
| Fifth year | 6 | | 3 | | | 5 | |
| | 6 | | 3 | | | 5 | |
| First year | 6 | | 3 | | | 5 | |
| Third year | 6 | | 3 | | | 5 | |
| | 6 | | 3 | | | 5 | |

| | | | | | | |
|---|---|---|---|---|---|---|
| | 6 | | 3 | | 5 | |
| First year | 6 | | 3 | | 5 | |
| | 6 | | 3 | | 5 | |
| Fourth year | 6 | | 2 | No | 5 | |
| | 6 | | 3 | | 5 | |
| | 6 | | 3 | | 5 | |
| Fifth year | 6 | | 3 | | 5 | |
| | 6 | | 3 | | 5 | |
| | 6 | | 3 | | 5 | |
| | 6 | | 3 | | 5 | |
| First year | 6 | | 3 | | 5 | |
| | 6 | | 3 | | 5 | |
| Second year | 6 | | 3 | | 5 | |
| Fourth year | 6 | | 3 | | 5 | |
| | 6 | | 3 | | 5 | |
| | 6 | | 3 | | 5 | |
| | 6 | | 3 | | 5 | |
| | 6 | | 3 | | 5 | |
| | 6 | | 3 | | 5 | |
| | 5 | Other, please specify | 3 | | 5 | |
| | 6 | | 3 | | 5 | |
| | 6 | | 3 | | 5 | |
| | 6 | | 3 | | 5 | |
| Third year | 1 | To improve concentration | 1 | Yes. If yes, please specify | 1 | During exam weeks |
| Second year | 4 | To improve academic performance | 1 | Yes. If yes, please specify | 1 | During exam weeks |
| Fourth year | 4 | To improve academic performance | 1 | Yes. If yes, please specify | 1 | During exam weeks |
| | 6 | | 3 | | 5 | |
| | 6 | | 3 | | 5 | |
| | 6 | | 3 | | 5 | |
| | 6 | | 3 | | 5 | |
| | 6 | | 3 | | 5 | |
| | 6 | | 3 | | 5 | |
| Second year | 4 | To improve academic performance | 2 | No | 5 | |
| Fourth year | 6 | | 3 | | 5 | |
| | 6 | | 3 | | 5 | |
| Third year | 6 | | 2 | No | 5 | |
| | 6 | | 3 | | 5 | |
| First year | 6 | | 3 | | 5 | |
| | 6 | | 3 | | 5 | |
| | 6 | | 3 | | 5 | |
| | 6 | | 3 | | 5 | |
| Third year | 4 | To improve academic performance | 1 | Yes. If yes, please specify | 1 | During exam weeks |
| Fourth year | 4 | To improve academic performance | 1 | Yes. If yes, please specify | 1 | During exam weeks |
| Third year | 3 | To increase alertness | 1 | Yes. If yes, please specify | 1 | During exam weeks |
| Third year | 4 | To improve academic performance | 1 | Yes. If yes, please specify | 1 | During exam weeks |
| Fourth year | 4 | To improve academic performance | 1 | Yes. If yes, please specify | 1 | During exam weeks |
| Fourth year | 4 | To improve academic performance | 1 | Yes. If yes, please specify | 1 | During exam weeks |
| Third year | 4 | To improve academic performance | 1 | Yes. If yes, please specify | 1 | During exam weeks |
| Fourth year | 4 | To improve academic performance | 1 | Yes. If yes, please specify | 1 | During exam weeks |
| Fourth year | 4 | To improve academic performance | 1 | Yes. If yes, please specify | 1 | During exam weeks |
| Third year | 4 | To improve academic performance | 1 | Yes. If yes, please specify | 1 | During exam weeks |
| Second year | 6 | | 2 | No | 5 | |

| | | | | | | |
|---|---|---|---|---|---|---|
| Second year | 6 | | 2 | No | 5 | |
| | 6 | | 3 | | 5 | |
| | 6 | | 3 | | 5 | |
| Fourth year | 4 | To improve academic performance | 1 | Yes. If yes, please specify | 1 | During exam weeks |
| | 6 | | 3 | | 5 | |
| Fourth year | 4 | To improve academic performance | 1 | Yes. If yes, please specify | 1 | During exam weeks |
| | 6 | | 3 | | 5 | |
| | 6 | | 3 | | 5 | |
| | 6 | | 3 | | 5 | |
| Third year | 6 | | 3 | | 5 | |
| Third year | 6 | | 3 | | 5 | |
| | 6 | | 3 | | 5 | |
| | 6 | | 3 | | 5 | |
| Third year | 6 | | 3 | | 5 | |
| | 6 | | 3 | | 5 | |
| | 6 | | 3 | | 5 | |
| First year | 4 | To improve academic performance | 3 | | 3 | During studying |
| | 6 | | 3 | | 5 | |
| Third year | 4 | To improve academic performance | 1 | Yes. If yes, please specify | 1 | During exam weeks |
| | 6 | | 3 | | 5 | |
| Fourth year | 4 | To improve academic performance | 1 | Yes. If yes, please specify | 3 | During studying |
| Second year | 6 | | 3 | | 5 | |
| | 6 | | 3 | | 5 | |
| Fourth year | 6 | | 3 | | 5 | |
| Third year | 4 | To improve academic performance | 1 | Yes. If yes, please specify | 1 | During exam weeks |
| | 6 | | 3 | | 5 | |
| | 6 | | 3 | | 5 | |
| | 6 | | 3 | | 5 | |
| Third year | 4 | To improve academic performance | 1 | Yes. If yes, please specify | 1 | During exam weeks |
| | 6 | | 3 | | 5 | |
| | 6 | | 3 | | 5 | |
| | 6 | | 3 | | 5 | |
| | 6 | | 3 | | 5 | |
| Fourth year | 4 | To improve academic performance | 1 | Yes. If yes, please specify | 1 | During exam weeks |
| Fourth year | 4 | To improve academic performance | 1 | Yes. If yes, please specify | 1 | During exam weeks |
| Third year | 4 | To improve academic performance | 1 | Yes. If yes, please specify | 1 | During exam weeks |
| Third year | 4 | To improve academic performance | 1 | Yes. If yes, please specify | 1 | During exam weeks |
| | 6 | | 3 | | 5 | |
| | 6 | | 3 | | 5 | |
| | 6 | | 3 | | 5 | |
| | 6 | | 3 | | 5 | |
| | 6 | | 3 | | 5 | |
| | 6 | | 3 | | 5 | |
| | 6 | | 3 | | 5 | |
| Third year | 3 | To increase alertness | 1 | Yes. If yes, please specify | 3 | During studying |
| | 6 | | 3 | | 5 | |
| | 6 | | 3 | | 5 | |
| | 6 | | 3 | | 5 | |
| Fifth year | 3 | To increase alertness | 1 | Yes. If yes, please specify | 3 | During studying |
| Fourth year | 1 | To improve concentration | 1 | Yes. If yes, please specify | 3 | During studying |
| | 6 | | 3 | | 5 | |
| | 6 | | 3 | | 5 | |

| | | | | | | |
|---|---|---|---|---|---|---|
| | 6 | | 3 | | 5 | |
| | 6 | | 3 | | 5 | |
| | 6 | | 3 | | 5 | |
| | 6 | | 3 | | 5 | |
| | 6 | | 3 | | 5 | |
| | 6 | | 3 | | 5 | |
| | 6 | | 3 | | 5 | |
| | 6 | | 3 | | 5 | |
| | 6 | | 3 | | 5 | |
| | 6 | | 3 | | 5 | |
| | 6 | | 3 | | 5 | |
| | 6 | | 3 | | 5 | |
| | 6 | | 3 | | 5 | |
| | 6 | | 3 | | 5 | |
| | 6 | | 3 | | 5 | |
| | 6 | | 3 | | 5 | |
| | 6 | | 3 | | 5 | |
| | 6 | | 3 | | 5 | |
| | 6 | | 3 | | 5 | |
| Second year | 6 | | 3 | | 5 | |
| Second year | 6 | | 3 | | 5 | |
| | 6 | | 3 | | 5 | |
| Fourth year | 6 | | 3 | | 5 | |
| | 6 | | 3 | | 5 | |
| Fourth year | 1 | To improve concentration | 1 | Yes. If yes, please specify | 1 | During exam weeks |
| | 6 | | 3 | | 5 | |
| Second year | 1 | To improve concentration | 1 | Yes. If yes, please specify | 1 | During exam weeks |
| | 6 | | 3 | | 5 | |

| | | | | | | |
|---|---|---|---|---|---|---|
| Fourth year | 1 | To improve concentration | 1 | Yes. If yes, please specify | 1 | During exam weeks |
| | 6 | | 3 | | 5 | |
| Third year | 3 | To increase alertness | 1 | Yes. If yes, please specify | 1 | During exam weeks |
| | 6 | | 3 | | 5 | |
| | 6 | | 3 | | 5 | |
| | 6 | | 3 | | 5 | |
| Fourth year | 3 | To increase alertness | 1 | Yes. If yes, please specify | 3 | During studying |
| | 6 | | 3 | | 5 | |
| Second year | 3 | To increase alertness | 1 | Yes. If yes, please specify | 1 | During exam weeks |
| Fourth year | 4 | To improve academic performance | 1 | Yes. If yes, please specify | 3 | During studying |
| | 6 | | 3 | | 5 | |
| Fourth year | 4 | To improve academic performance | 1 | Yes. If yes, please specify | 1 | During exam weeks |
| Third year | 4 | To improve academic performance | 1 | Yes. If yes, please specify | 1 | During exam weeks |

| | | | | | | |
|---|---|---|---|---|---|---|
| Second year | 6 | | 2 | No | 4 | Daily basis |
| | 6 | | 3 | | 5 | |
| | 6 | | 3 | | 5 | |
| | 6 | | 3 | | 5 | |
| Fifth year | 4 | To improve academic performance | 1 | Yes. If yes, please specify | 3 | During studying |
| Third year | 4 | To improve academic performance | 1 | Yes. If yes, please specify | 1 | During exam weeks |
| | 6 | | 3 | | 5 | |
| Fourth year | 3 | To increase alertness | 1 | Yes. If yes, please specify | 1 | During exam weeks |
| | 6 | | 3 | | 5 | |
| Fourth year | 4 | To improve academic performance | 1 | Yes. If yes, please specify | 1 | During exam weeks |
| | 6 | | 3 | | 5 | |
| Third year | 1 | To improve concentration | 1 | Yes. If yes, please specify | 2 | During course work deadlines |
| Fourth year | 3 | To increase alertness | 1 | Yes. If yes, please specify | 3 | During studying |

| | | | | | | | |
|---|---|---|---|---|---|---|---|
| Fourth year | 4 | To improve academic performance | 1 | Yes. If yes, please specify | | 3 | During studying |
| Fourth year | 3 | To increase alertness | 1 | Yes. If yes, please specify | | 3 | During studying |
| | 1 concentration<br>2 memory<br>3 alertness<br>4 academic | | 1 yes<br>2 no<br>3 NA | | | 1 exam<br>2 work deadlines<br>3 studying<br>4 daily | |
| 5 others | | | | | 5 NA | | 5 more 2 y |
| 6 NA | | | | | | | 6 NA |

| howLong | Q11 | frequency | Q12 | buyFrom | Q13 | Q13_6_TEXT | cost | Q14 |
|---|---|---|---|---|---|---|---|---|
| | How long did you take the CE for? | | [illegible] | | [illegible] | Where did you obtain these drugs/substances? - Other please specify - Text | | [illegible] |
| 6 | | 6 | | 7 | | | 6 | |
| 6 | | 6 | | 7 | | | 6 | |
| 1 | Less than 1 month | 4 | Once a semester/term | 1 | They are prescribed for me | | 3 | Fair |
| 6 | | 6 | | 7 | | | 6 | |
| 6 | | 6 | | 7 | | | 6 | |
| 6 | | 6 | | 7 | | | 6 | |
| 6 | | 6 | | 7 | | | 6 | |
| 6 | | 6 | | 7 | | | 6 | |
| 6 | | 6 | | 7 | | | 6 | |
| 6 | | 6 | | 7 | | | 6 | |
| 6 | | 6 | | 7 | | | 6 | |
| 6 | | 6 | | 7 | | | 6 | |
| 6 | | 6 | | 7 | | | 6 | |
| 6 | | 6 | | 7 | | | 6 | |
| 6 | | 6 | | 7 | | | 6 | |
| 6 | | 6 | | 7 | | | 6 | |
| 6 | | 6 | | 7 | | | 6 | |
| 6 | | 6 | | 7 | | | 6 | |
| 6 | | 6 | | 7 | | | 6 | |
| 1 | Less than 1 month | 1 | Daily | 6 | Other, please specify | Over counter | 2 | Expensive |
| 6 | | 6 | | 7 | | | 6 | |
| 6 | | 6 | | 7 | | | 6 | |
| 6 | | 6 | | 7 | | | 6 | |
| 6 | | 6 | | 7 | | | 6 | |
| 6 | | 6 | | 7 | | | 6 | |
| 6 | | 6 | | 7 | | | 6 | |
| 1 | Less than 1 month | 5 | Less than once a semester/term | 6 | Other, please specify | Bought it from supplement store | 2 | Expensive |
| 6 | | 6 | | 7 | | | 6 | |
| 6 | | 6 | | 7 | | | 6 | |
| 6 | | 6 | | 7 | | | 6 | |
| 4 | 1 year but less than 2 years | 2 | Weekly | 5 | I purchased them online | | 3 | Fair |
| 6 | | 6 | | 7 | | | 6 | |
| 6 | | 6 | | 7 | | | 6 | |
| 6 | | 6 | | 7 | | | 6 | |
| 1 | Less than 1 month | 2 | Weekly | 6 | Other, please specify | i bought them from the pharmacy | 2 | Expensive |
| 6 | | 6 | | 7 | | | 6 | |
| 6 | | 6 | | 7 | | | 6 | |
| 6 | | 6 | | 7 | | | 6 | |
| 6 | | 6 | | 7 | | | 6 | |
| 6 | | 6 | | 7 | | | 6 | |
| 6 | | 6 | | 7 | | | 6 | |
| 6 | | 6 | | 7 | | | 6 | |
| 6 | | 6 | | 7 | | | 6 | |
| 6 | | 6 | | 7 | | | 6 | |
| 2 | 1 month but less than 6 months | 1 | Daily | 2 | They were given to me by a friend | | 3 | Fair |
| 6 | | 6 | | 7 | | | 6 | |
| 6 | | 6 | | 7 | | | 6 | |
| 2 | 1 month but less than 6 months | 1 | Daily | 5 | I purchased them online | | 3 | Fair |
| 6 | | 6 | | 7 | | | 6 | |
| 1 | Less than 1 month | 1 | Daily | 5 | I purchased them online | | 3 | Fair |

| | | | | | | | | |
|---|---|---|---|---|---|---|---|---|
| 6 | | 6 | | 7 | | | 6 | |
| 6 | | 6 | | 7 | | | 6 | |
| 6 | | 6 | | 7 | | | 6 | |
| 6 | | 6 | | 7 | | | 6 | |
| 2 | 1 month but less than 6 months | 1 | Daily | 6 | Other, please specify | purchased from boots | 3 | Fair |
| 6 | | 6 | | 7 | | | 6 | |
| 6 | | 6 | | 7 | | | 6 | |
| 1 | Less than 1 month | 4 | Once a semester/term | 6 | Other, please specify | ............ | 3 | Fair |
| 6 | | 6 | | 7 | | | 6 | |
| 1 | Less than 1 month | 5 | Less than once a semester/term | 1 | They are prescribed for me | | 3 | Fair |
| 1 | Less than 1 month | 5 | Less than once a semester/term | 2 | They were given to me by a friend | | 2 | Expensive |
| 1 | Less than 1 month | 4 | Once a semester/term | 4 | They were prescribed for somebody else | | 4 | Cheap |
| 6 | | 6 | | 7 | | | 6 | |
| 6 | | 6 | | 7 | | | 6 | |
| 2 | 1 month but less than 6 months | 1 | Daily | 5 | I purchased them online | | 2 | Expensive |
| 1 | Less than 1 month | 5 | Less than once a semester/term | 5 | I purchased them online | | 2 | Expensive |
| 6 | | 6 | | 7 | | | 6 | |
| 6 | | 6 | | 7 | | | 6 | |
| 6 | | 6 | | 7 | | | 6 | |
| 6 | | 6 | | 7 | | | 6 | |
| 6 | | 6 | | 7 | | | 6 | |
| 6 | | 6 | | 7 | | | 6 | |
| 6 | | 6 | | 7 | | | 6 | |
| 6 | | 6 | | 7 | | | 6 | |
| 6 | | 6 | | 7 | | | 6 | |
| 6 | | 6 | | 7 | | | 6 | |
| 6 | | 6 | | 7 | | | 6 | |
| 1 | Less than 1 month | 5 | Less than once a semester/term | 5 | I purchased them online | | 2 | Expensive |
| 1 | Less than 1 month | 5 | Less than once a semester/term | 2 | They were given to me by a friend | | 2 | Expensive |
| 2 | 1 month but less than 6 months | 4 | Once a semester/term | 5 | I purchased them online | | 3 | Fair |
| 1 | Less than 1 month | 1 | Daily | 5 | I purchased them online | | 3 | Fair |
| 6 | | 6 | | 7 | | | 6 | |
| 1 | Less than 1 month | 1 | Daily | 2 | They were given to me by a friend | | 2 | Expensive |
| 1 | Less than 1 month | 1 | Daily | 5 | I purchased them online | | 2 | Expensive |
| 2 | 1 month but less than 6 months | 1 | Daily | 5 | I purchased them online | | 2 | Expensive |
| 2 | 1 month but less than 6 months | 1 | Daily | 5 | I purchased them online | | 2 | Expensive |
| 2 | 1 month but less than 6 months | 1 | Daily | 5 | I purchased them online | | 2 | Expensive |
| 2 | 1 month but less than 6 months | 1 | Daily | 5 | I purchased them online | | 2 | Expensive |
| 1 | Less than 1 month | 1 | Daily | 5 | I purchased them online | | 2 | Expensive |
| 2 | 1 month but less than 6 months | 1 | Daily | 5 | I purchased them online | | 2 | Expensive |
| 2 | 1 month but less than 6 months | 1 | Daily | 5 | I purchased them online | | 2 | Expensive |
| 2 | 1 month but less than 6 months | 1 | Daily | 5 | I purchased them online | | 2 | Expensive |
| 6 | | 6 | | 7 | | | 6 | |
| 2 | 1 month but less than 6 months | 1 | Daily | 5 | I purchased them online | | 2 | Expensive |
| 1 | Less than 1 month | 4 | Once a semester/term | 5 | I purchased them online | | 3 | Fair |
| 2 | 1 month but less than 6 months | 4 | Once a semester/term | 5 | I purchased them online | | 3 | Fair |
| 2 | 1 month but less than 6 months | 1 | Daily | 5 | I purchased them online | | 3 | Fair |
| 2 | 1 month but less than 6 months | 1 | Daily | 5 | I purchased them online | | 3 | Fair |
| 6 | | 6 | | 7 | | | 6 | |
| 2 | 1 month but less than 6 months | 1 | Daily | 5 | I purchased them online | | 2 | Expensive |
| 3 | 6 months but less than 1 year | 1 | Daily | 5 | I purchased them online | | 2 | Expensive |
| 1 | Less than 1 month | 1 | Daily | 2 | They were given to me by a friend | | 3 | Fair |

| | | | | | | | |
|---|---|---|---|---|---|---|---|
| 6 | | 6 | | 7 | | 6 | |
| 6 | | 6 | | 7 | | 6 | |
| 2 | 1 month but less than 6 months | 1 | Daily | 5 | I purchased them online | 2 | Expensive |
| 2 | 1 month but less than 6 months | 4 | Once a semester/term | 2 | They were given to me by a friend | 2 | Expensive |
| 6 | | 6 | | 7 | | 6 | |
| 2 | 1 month but less than 6 months | 1 | Daily | 2 | They were given to me by a friend | 2 | Expensive |
| 2 | 1 month but less than 6 months | 1 | Daily | 5 | I purchased them online | 2 | Expensive |
| 2 | 1 month but less than 6 months | 1 | Daily | 7 | | 6 | |
| 2 | 1 month but less than 6 months | 1 | Daily | 5 | I purchased them online | 3 | Fair |
| 2 | 1 month but less than 6 months | 1 | Daily | 5 | I purchased them online | 3 | Fair |
| 6 | | 6 | | 7 | | 6 | |
| 6 | | 6 | | 7 | | 6 | |
| 2 | 1 month but less than 6 months | 1 | Daily | 5 | I purchased them online | 3 | Fair |
| 6 | | 6 | | 7 | | 6 | |
| 6 | | 6 | | 7 | | 6 | |
| 2 | 1 month but less than 6 months | 1 | Daily | 2 | They were given to me by a friend | 3 | Fair |
| 6 | | 6 | | 7 | | 6 | |
| 6 | | 6 | | 7 | | 6 | |
| 6 | | 6 | | 7 | | 6 | |
| 6 | | 6 | | 7 | | 6 | |
| 2 | 1 month but less than 6 months | 2 | Weekly | 5 | I purchased them online | 3 | Fair |
| 6 | | 6 | | 7 | | 6 | |
| 1 | Less than 1 month | 5 | Less than once a semester/term | 2 | They were given to me by a friend | 2 | Expensive |
| 6 | | 6 | | 7 | | 6 | |
| 6 | | 6 | | 7 | | 6 | |
| 6 | | 6 | | 7 | | 6 | |
| 1 | Less than 1 month | 4 | Once a semester/term | 5 | I purchased them online | 3 | Fair |
| 4 | 1 year but less than 2 years | 4 | Once a semester/term | 5 | I purchased them online | 2 | Expensive |
| 6 | | 6 | | 7 | | 6 | |
| 6 | | 6 | | 7 | | 6 | |
| 6 | | 6 | | 7 | | 6 | |
| 6 | | 6 | | 7 | | 6 | |
| 6 | | 6 | | 7 | | 6 | |
| 2 | 1 month but less than 6 months | 4 | Once a semester/term | 2 | They were given to me by a friend | 2 | Expensive |
| 6 | | 6 | | 7 | | 6 | |
| 6 | | 6 | | 7 | | 6 | |
| 2 | 1 month but less than 6 months | 4 | Once a semester/term | 5 | I purchased them online | 3 | Fair |
| 3 | 6 months but less than 1 year | 4 | Once a semester/term | 5 | I purchased them online | 2 | Expensive |
| 6 | | 6 | | 7 | | 6 | |
| 6 | | 6 | | 7 | | 6 | |
| 3 | 6 months but less than 1 year | 4 | Once a semester/term | 5 | I purchased them online | 3 | Fair |
| 2 | 1 month but less than 6 months | 2 | Weekly | 2 | They were given to me by a friend | 2 | Expensive |
| 2 | 1 month but less than 6 months | 3 | Monthly | 5 | I purchased them online | 3 | Fair |
| 6 | | 6 | | 7 | | 6 | |
| 6 | | 6 | | 7 | | 6 | |
| 6 | | 6 | | 7 | | 6 | |
| 6 | | 6 | | 7 | | 6 | |
| 6 | | 6 | | 7 | | 6 | |
| 6 | | 6 | | 7 | | 6 | |
| 6 | | 6 | | 7 | | 6 | |
| 6 | | 6 | | 7 | | 6 | |
| 1 | Less than 1 month | 4 | Once a semester/term | 5 | I purchased them online | 2 | Expensive |

| | | | | | | | |
|---|---|---|---|---|---|---|---|
| 2 | 1 month but less than 6 months | 5 | Less than once a semester/term | 5 | I purchased them online | 2 | Expensive |
| 6 | | 6 | | 7 | | 6 | |
| 6 | | 6 | | 7 | | 6 | |
| 6 | | 6 | | 7 | | 6 | |
| 2 | 1 month but less than 6 months | 5 | Less than once a semester/term | 5 | I purchased them online | 2 | Expensive |
| 2 | 1 month but less than 6 months | 4 | Once a semester/term | 5 | I purchased them online | 2 | Expensive |
| 6 | | 6 | | 7 | | 6 | |
| 6 | | 6 | | 7 | | 6 | |
| 6 | | 6 | | 7 | | 6 | |
| 6 | | 6 | | 7 | | 6 | |
| 6 | | 6 | | 7 | | 6 | |
| 2 | 1 month but less than 6 months | 4 | Once a semester/term | 5 | I purchased them online | 2 | Expensive |
| 6 | | 6 | | 7 | | 6 | |
| 6 | | 6 | | 7 | | 6 | |
| 6 | | 6 | | 7 | | 6 | |
| 1 | Less than 1 month | 4 | Once a semester/term | 2 | They were given to me by a friend | 2 | Expensive |
| 6 | | 6 | | 7 | | 6 | |
| 6 | | 6 | | 7 | | 6 | |
| 6 | | 6 | | 7 | | 6 | |
| 6 | | 6 | | 7 | | 6 | |
| 6 | | 6 | | 7 | | 6 | |
| 6 | | 6 | | 7 | | 6 | |
| 6 | | 6 | | 7 | | 6 | |
| 1 | Less than 1 month | 3 | Monthly | 1 | They are prescribed for me | 2 | Expensive |
| 6 | | 6 | | 7 | | 6 | |
| 6 | | 6 | | 7 | | 6 | |
| 6 | | 6 | | 7 | | 6 | |
| 6 | | 6 | | 7 | | 6 | |
| 6 | | 6 | | 7 | | 6 | |
| 6 | | 6 | | 7 | | 6 | |
| 6 | | 6 | | 7 | | 6 | |
| 2 | 1 month but less than 6 months | 3 | Monthly | 5 | I purchased them online | 3 | Fair |
| 6 | | 6 | | 7 | | 6 | |
| 2 | 1 month but less than 6 months | 3 | Monthly | 5 | I purchased them online | 2 | Expensive |
| 6 | | 6 | | 7 | | 6 | |
| 1 | Less than 1 month | 4 | Once a semester/term | 5 | I purchased them online | 3 | Fair |
| 6 | | 6 | | 7 | | 6 | |
| 2 | 1 month but less than 6 months | 4 | Once a semester/term | 5 | I purchased them online | 2 | Expensive |
| 6 | | 6 | | 7 | | 6 | |
| 2 | 1 month but less than 6 months | 4 | Once a semester/term | 5 | I purchased them online | 2 | Expensive |
| 6 | | 6 | | 7 | | 6 | |
| 6 | | 6 | | 7 | | 6 | |
| 6 | | 6 | | 7 | | 6 | |
| 6 | | 6 | | 7 | | 6 | |
| 6 | | 6 | | 7 | | 6 | |
| 6 | | 6 | | 7 | | 6 | |
| 6 | | 6 | | 7 | | 6 | |
| 6 | | 6 | | 7 | | 6 | |
| 6 | | 6 | | 7 | | 6 | |
| 6 | | 6 | | 7 | | 6 | |
| 6 | | 6 | | 7 | | 6 | |
| 6 | | 6 | | 7 | | 6 | |

| | | | | | | | | |
|---|---|---|---|---|---|---|---|---|
| 6 | | 6 | | 7 | | | 6 | |
| 6 | | 6 | | 7 | | | 6 | |
| 6 | | 6 | | 7 | | | 6 | |
| 6 | | 6 | | 7 | | | 2 | Expensive |
| 6 | | 6 | | 7 | | | 6 | |
| 6 | | 6 | | 7 | | | 6 | |
| 1 | Less than 1 month | 4 | Once a semester/term | 2 | They were given to me by a friend | | 3 | Fair |
| 6 | | 6 | | 7 | | | 6 | |
| 6 | | 6 | | 7 | | | 6 | |
| 6 | | 6 | | 7 | | | 6 | |
| 6 | | 6 | | 7 | | | 6 | |
| 1 | Less than 1 month | 5 | Less than once a semester/term | 4 | They were prescribed for somebody else | | 2 | Expensive |
| 5 | 2 years and more | 1 | Daily | 5 | I purchased them online | | 3 | Fair |
| 6 | | 6 | | 7 | | | 6 | |
| 6 | | 6 | | 7 | | | 6 | |
| 6 | | 6 | | 7 | | | 6 | |
| 6 | | 6 | | 7 | | | 6 | |
| 6 | | 6 | | 6 | Other, please specify | not used | 6 | |
| 6 | | 6 | | 7 | | | 6 | |
| 6 | | 6 | | 7 | | | 6 | |
| 6 | | 6 | | 7 | | | 6 | |
| 1 | Less than 1 month | 1 | Daily | 1 | They are prescribed for me | | 1 | Very expensive |
| 6 | | 6 | | 7 | | | 6 | |
| 6 | | 6 | | 7 | | | 6 | |
| 6 | | 6 | | 7 | | | 6 | |
| 6 | | 6 | | 7 | | | 6 | |
| 6 | | 6 | | 7 | | | 6 | |
| 6 | | 6 | | 7 | | | 6 | |
| 6 | | 6 | | 7 | | | 6 | |
| 6 | | 6 | | 7 | | | 6 | |
| 1 | Less than 1 month | 1 | Daily | 5 | I purchased them online | | 3 | Fair |
| 6 | | 6 | | 7 | | | 6 | |
| 3 | 6 months but less than 1 year | 2 | Weekly | 1 | They are prescribed for me | | 1 | Very expensive |
| 6 | | 6 | | 7 | | | 6 | |
| 1 | Less than 1 month | 3 | Monthly | 6 | Other, please specify | Not your business | 2 | Expensive |
| 6 | | 6 | | 7 | | | 6 | |
| 6 | | 6 | | 7 | | | 6 | |
| 6 | | 6 | | 7 | | | 6 | |
| 6 | | 6 | | 7 | | | 6 | |
| 6 | | 6 | | 7 | | | 6 | |
| 6 | | 6 | | 7 | | | 6 | |
| 6 | | 6 | | 7 | | | 6 | |
| 6 | | 6 | | 7 | | | 6 | |
| 6 | | 6 | | 7 | | | 6 | |
| 6 | | 6 | | 7 | | | 6 | |
| 6 | | 6 | | 7 | | | 6 | |
| 6 | | 6 | | 7 | | | 6 | |
| 6 | | 6 | | 7 | | | 6 | |
| 6 | | 6 | | 7 | | | 6 | |
| 2 | 1 month but less than 6 months | 5 | Less than once a semester/term | 5 | I purchased them online | | 3 | Fair |
| 6 | | 6 | | 7 | | | 6 | |
| 6 | | 6 | | 7 | | | 6 | |

| | | | | | | | | |
|---|---|---|---|---|---|---|---|---|
| 6 | | 6 | | 7 | | | 6 | |
| 6 | | 6 | | 7 | | | 6 | |
| 6 | | 6 | | 7 | | | 6 | |
| 6 | | 6 | | 7 | | | 6 | |
| 6 | | 6 | | 7 | | | 6 | |
| 6 | | 6 | | 7 | | | 6 | |
| 6 | | 6 | | 7 | | | 6 | |
| 6 | | 6 | | 7 | | | 6 | |
| 6 | | 6 | | 7 | | | 6 | |
| 2 | 1 month but less than 6 months | 1 | Daily | 1 | They are prescribed for me | | 4 | Cheap |
| 6 | | 6 | | 7 | | | 6 | |
| 6 | | 6 | | 7 | | | 6 | |
| 1 | Less than 1 month | 5 | Less than once a semester/term | 6 | Other, please specify | Does caffeinated drinks count?? | 6 | |
| 6 | | 6 | | 7 | | | 6 | |
| 6 | | 6 | | 7 | | | 6 | |
| 6 | | 6 | | 7 | | | 6 | |
| 6 | | 6 | | 7 | | | 6 | |
| 6 | | 6 | | 7 | | | 6 | |
| 1 | Less than 1 month | 5 | Less than once a semester/term | 7 | | | 6 | |
| 6 | | 6 | | 7 | | | 6 | |
| 6 | | 6 | | 7 | | | 6 | |
| 6 | | 6 | | 7 | | | 6 | |
| 6 | | 6 | | 7 | | | 6 | |
| 6 | | 6 | | 7 | | | 6 | |
| 6 | | 6 | | 7 | | | 6 | |
| 1 | Less than 1 month | 3 | Monthly | 1 | They are prescribed for me | | 3 | Fair |
| 6 | | 6 | | 7 | | | 6 | |
| 6 | | 6 | | 7 | | | 6 | |
| 6 | | 6 | | 7 | | | 6 | |
| 6 | | 6 | | 7 | | | 6 | |
| 6 | | 6 | | 7 | | | 6 | |
| 6 | | 6 | | 7 | | | 6 | |
| 6 | | 6 | | 7 | | | 6 | |
| 6 | | 6 | | 7 | | | 6 | |
| 6 | | 6 | | 7 | | | 6 | |
| 6 | | 6 | | 7 | | | 6 | |
| 6 | | 6 | | 7 | | | 6 | |
| 6 | | 6 | | 7 | | | 6 | |
| 6 | | 6 | | 7 | | | 6 | |
| 6 | | 6 | | 7 | | | 6 | |
| 1 | Less than 1 month | 5 | Less than once a semester/term | 5 | I purchased them online | | 2 | Expensive |
| 1 | Less than 1 month | 1 | Daily | 6 | Other, please specify | I taked from pharmacy or hospital | 3 | Fair |
| 2 | 1 month but less than 6 months | 3 | Monthly | 1 | They are prescribed for me | | 3 | Fair |
| 6 | | 6 | | 7 | | | 6 | |
| 6 | | 6 | | 7 | | | 6 | |
| 6 | | 6 | | 7 | | | 6 | |
| 6 | | 6 | | 7 | | | 6 | |
| 6 | | 6 | | 7 | | | 6 | |
| 6 | | 6 | | 7 | | | 6 | |
| 6 | | 6 | | 7 | | | 6 | |
| 6 | | 6 | | 7 | | | 6 | |
| 6 | | 6 | | 7 | | | 6 | |

| | | | | | | | |
|---|---|---|---|---|---|---|---|
| 6 | | 6 | | 7 | | 6 | |
| 6 | | 6 | | 7 | | 6 | |
| 6 | | 6 | | 7 | | 6 | |
| 6 | | 6 | | 7 | | 6 | |
| 6 | | 6 | | 7 | | 6 | |
| 6 | | 6 | | 7 | | 6 | |
| 6 | | 6 | | 7 | | 6 | |
| 6 | | 6 | | 7 | | 6 | |
| 6 | | 6 | | 7 | | 6 | |
| 6 | | 6 | | 7 | | 6 | |
| 6 | | 6 | | 7 | | 6 | |
| 6 | | 6 | | 7 | | 6 | |
| 6 | | 6 | | 7 | | 6 | |
| 6 | | 6 | | 7 | | 6 | |
| 6 | | 6 | | 7 | | 6 | |
| 6 | | 6 | | 7 | | 6 | |
| 2 | 1 month but less than 6 months | 1 | Daily | 1 | They are prescribed for me | 3 | Fair |
| 6 | | 6 | | 7 | | 6 | |
| 6 | | 6 | | 7 | | 6 | |
| 6 | | 6 | | 7 | | 6 | |
| 6 | | 6 | | 7 | | 6 | |
| 6 | | 6 | | 7 | | 6 | |
| 6 | | 6 | | 7 | | 6 | |
| 6 | | 6 | | 7 | | 6 | |
| 6 | | 6 | | 7 | | 6 | |
| 6 | | 6 | | 7 | | 6 | |
| 1 | Less than 1 month | 4 | Once a semester/term | 5 | I purchased them online | 2 | Expensive |
| 6 | | 6 | | 7 | | 6 | |
| 6 | | 6 | | 7 | | 6 | |
| 3 | 6 months but less than 1 year | 5 | Less than once a semester/term | 1 | They are prescribed for me | 3 | Fair |
| 6 | | 6 | | 7 | | 6 | |
| 6 | | 6 | | 7 | | 6 | |
| 6 | | 6 | | 7 | | 6 | |
| 1 | Less than 1 month | 4 | Once a semester/term | 5 | I purchased them online | 2 | Expensive |
| 6 | | 6 | | 7 | | 6 | |
| 1 | Less than 1 month | 5 | Less than once a semester/term | 1 | They are prescribed for me | 3 | Fair |
| 1 | Less than 1 month | 4 | Once a semester/term | 5 | I purchased them online | 2 | Expensive |
| 6 | | 6 | | 7 | | 6 | |
| 6 | | 6 | | 7 | | 6 | |
| 6 | | 6 | | 7 | | 6 | |
| 6 | | 4 | Once a semester/term | 5 | I purchased them online | 3 | Fair |
| 6 | | 6 | | 7 | | 6 | |
| 6 | | 6 | | 7 | | 6 | |
| 6 | | 6 | | 7 | | 6 | |
| 6 | | 6 | | 7 | | 6 | |
| 6 | | 6 | | 7 | | 6 | |
| 6 | | 6 | | 7 | | 6 | |
| 6 | | 6 | | 7 | | 6 | |
| 6 | | 6 | | 7 | | 6 | |
| 6 | | 6 | | 7 | | 6 | |
| 6 | | 6 | | 7 | | 6 | |
| 6 | | 6 | | 7 | | 6 | |

| | | | | | | | |
|---|---|---|---|---|---|---|---|
| 6 | | 6 | | 7 | | 6 | |
| 6 | | 6 | | 7 | | 6 | |
| 6 | | 6 | | 7 | | 6 | |
| 6 | | 6 | | 7 | | 6 | |
| 6 | | 6 | | 7 | | 6 | |
| 6 | | 6 | | 7 | | 6 | |
| 6 | | 6 | | 7 | | 6 | |
| 6 | | 6 | | 7 | | 6 | |
| 6 | | 6 | | 7 | | 6 | |
| 6 | | 6 | | 7 | | 6 | |
| 6 | | 6 | | 7 | | 6 | |
| 6 | | 6 | | 7 | | 6 | |
| 6 | | 6 | | 7 | | 6 | |
| 6 | | 6 | | 7 | | 6 | |
| 6 | | 6 | | 7 | | 6 | |
| 6 | | 6 | | 7 | | 6 | |
| 6 | | 6 | | 7 | | 6 | |
| 6 | | 6 | | 7 | | 6 | |
| 6 | | 6 | | 7 | | 6 | |
| 6 | | 6 | | 7 | | 6 | |
| 6 | | 6 | | 7 | | 6 | |
| 6 | | 6 | | 7 | | 6 | |
| 6 | | 6 | | 7 | | 6 | |
| 2 | 1 month but less than 6 months | 4 | Once a semester/term | 5 | I purchased them online | 2 | Expensive |
| 3 | 6 months but less than 1 year | 5 | Less than once a semester/term | 5 | I purchased them online | 2 | Expensive |
| 2 | 1 month but less than 6 months | 5 | Less than once a semester/term | 5 | I purchased them online | 2 | Expensive |
| 6 | | 6 | | 7 | | 6 | |
| 6 | | 6 | | 7 | | 6 | |
| 6 | | 6 | | 7 | | 6 | |
| 6 | | 6 | | 7 | | 6 | |
| 6 | | 6 | | 7 | | 6 | |
| 6 | | 6 | | 7 | | 6 | |
| 6 | | 6 | | 7 | | 6 | |
| 6 | | 6 | | 7 | | 6 | |
| 6 | | 6 | | 7 | | 6 | |
| 6 | | 6 | | 7 | | 6 | |
| 6 | | 6 | | 7 | | 6 | |
| 6 | | 6 | | 7 | | 6 | |
| 6 | | 6 | | 7 | | 6 | |
| 6 | | 6 | | 7 | | 6 | |
| 6 | | 6 | | 7 | | 6 | |
| 2 | 1 month but less than 6 months | 5 | Less than once a semester/term | 5 | I purchased them online | 3 | Fair |
| 2 | 1 month but less than 6 months | 5 | Less than once a semester/term | 5 | I purchased them online | 2 | Expensive |
| 2 | 1 month but less than 6 months | 4 | Once a semester/term | 5 | I purchased them online | 2 | Expensive |
| 2 | 1 month but less than 6 months | 4 | Once a semester/term | 5 | I purchased them online | 2 | Expensive |
| 2 | 1 month but less than 6 months | 5 | Less than once a semester/term | 5 | I purchased them online | 2 | Expensive |
| 2 | 1 month but less than 6 months | 4 | Once a semester/term | 2 | They were given to me by a friend | 2 | Expensive |
| 2 | 1 month but less than 6 months | 5 | Less than once a semester/term | 5 | I purchased them online | 2 | Expensive |
| 2 | 1 month but less than 6 months | 4 | Once a semester/term | 5 | I purchased them online | 2 | Expensive |
| 2 | 1 month but less than 6 months | 4 | Once a semester/term | 5 | I purchased them online | 2 | Expensive |
| 2 | 1 month but less than 6 months | 4 | Once a semester/term | 5 | I purchased them online | 2 | Expensive |
| 6 | | 6 | | 7 | | 6 | |

| | | | | | | | |
|---|---|---|---|---|---|---|---|
| 6 | | 6 | | 7 | | 6 | |
| 6 | | 6 | | 7 | | 6 | |
| 6 | | 6 | | 7 | | 6 | |
| 2 | 1 month but less than 6 months | 4 | Once a semester/term | 5 | I purchased them online | 2 | Expensive |
| 6 | | 6 | | 7 | | 6 | |
| 3 | 6 months but less than 1 year | 5 | Less than once a semester/term | 5 | I purchased them online | 2 | Expensive |
| 6 | | 6 | | 7 | | 6 | |
| 6 | | 6 | | 7 | | 6 | |
| 6 | | 6 | | 7 | | 6 | |
| 6 | | 6 | | 7 | | 6 | |
| 6 | | 6 | | 7 | | 6 | |
| 6 | | 6 | | 7 | | 6 | |
| 6 | | 6 | | 7 | | 6 | |
| 6 | | 6 | | 7 | | 6 | |
| 6 | | 6 | | 7 | | 6 | |
| 6 | | 6 | | 7 | | 6 | |
| 6 | | 6 | | 7 | | 6 | |
| 6 | | 6 | | 7 | | 6 | |
| 2 | 1 month but less than 6 months | 4 | Once a semester/term | 5 | I purchased them online | 3 | Fair |
| 6 | | 6 | | 7 | | 6 | |
| 1 | Less than 1 month | 5 | Less than once a semester/term | 2 | They were given to me by a friend | 3 | Fair |
| 6 | | 6 | | 7 | | 6 | |
| 6 | | 6 | | 7 | | 6 | |
| 6 | | 6 | | 7 | | 6 | |
| 2 | 1 month but less than 6 months | 5 | Less than once a semester/term | 5 | I purchased them online | 3 | Fair |
| 6 | | 6 | | 7 | | 6 | |
| 6 | | 6 | | 7 | | 6 | |
| 6 | | 6 | | 7 | | 6 | |
| 1 | Less than 1 month | 5 | Less than once a semester/term | 2 | They were given to me by a friend | 3 | Fair |
| 6 | | 6 | | 7 | | 6 | |
| 6 | | 6 | | 7 | | 6 | |
| 6 | | 6 | | 7 | | 6 | |
| 6 | | 6 | | 7 | | 6 | |
| 1 | Less than 1 month | 5 | Less than once a semester/term | 2 | They were given to me by a friend | 3 | Fair |
| 2 | 1 month but less than 6 months | 5 | Less than once a semester/term | 5 | I purchased them online | 3 | Fair |
| 2 | 1 month but less than 6 months | 4 | Once a semester/term | 2 | They were given to me by a friend | 3 | Fair |
| 2 | 1 month but less than 6 months | 4 | Once a semester/term | 2 | They were given to me by a friend | 3 | Fair |
| 6 | | 6 | | 7 | | 6 | |
| 6 | | 6 | | 7 | | 6 | |
| 6 | | 6 | | 7 | | 6 | |
| 6 | | 6 | | 7 | | 6 | |
| 6 | | 6 | | 7 | | 6 | |
| 6 | | 6 | | 7 | | 6 | |
| 6 | | 6 | | 7 | | 6 | |
| 2 | 1 month but less than 6 months | 2 | Weekly | 5 | I purchased them online | 3 | Fair |
| 6 | | 6 | | 7 | | 6 | |
| 6 | | 6 | | 7 | | 6 | |
| 6 | | 6 | | 7 | | 6 | |
| 3 | 6 months but less than 1 year | 2 | Weekly | 5 | I purchased them online | 3 | Fair |
| 3 | 6 months but less than 1 year | 2 | Weekly | 5 | I purchased them online | 3 | Fair |
| 6 | | 6 | | 7 | | 6 | |
| 6 | | 6 | | 7 | | 6 | |

| | | | | | | | |
|---|---|---|---|---|---|---|---|
| 6 | | 6 | | 7 | | 6 | |
| 6 | | 6 | | 7 | | 6 | |
| 6 | | 6 | | 7 | | 6 | |
| 6 | | 6 | | 7 | | 6 | |
| 6 | | 6 | | 7 | | 6 | |
| 6 | | 6 | | 7 | | 6 | |
| 6 | | 6 | | 7 | | 6 | |
| 6 | | 6 | | 7 | | 6 | |
| 6 | | 6 | | 7 | | 6 | |
| 6 | | 6 | | 7 | | 6 | |
| 6 | | 6 | | 7 | | 6 | |
| 6 | | 6 | | 7 | | 6 | |
| 6 | | 6 | | 7 | | 6 | |
| 6 | | 6 | | 7 | | 6 | |
| 6 | | 6 | | 7 | | 6 | |
| 6 | | 6 | | 7 | | 6 | |
| 6 | | 6 | | 7 | | 6 | |
| 6 | | 6 | | 7 | | 6 | |
| 6 | | 6 | | 7 | | 6 | |
| 6 | | 6 | | 7 | | 6 | |
| 6 | | 6 | | 7 | | 6 | |
| 6 | | 6 | | 7 | | 6 | |
| 6 | | 6 | | 7 | | 6 | |
| 6 | | 6 | | 7 | | 6 | |
| 2 | 1 month but less than 6 months | 4 | Once a semester/ term | 5 | I purchased them online | 3 | Fair |
| 6 | | 6 | | 7 | | 6 | |
| 1 | Less than 1 month | 4 | Once a semester/ term | 1 | They are prescribed for me | 2 | Expensive |
| 6 | | 6 | | 7 | | 6 | |

| | | | | | | | | |
|---|---|---|---|---|---|---|---|---|
| 3 | 6 months but less than 1 year | 4 | Once a semester/ term | 5 | I purchased them online | | 2 | Expensive |
| 6 | | 6 | | 7 | | | 6 | |
| 2 | 1 month but less than 6 months | 4 | Once a semester/ term | 2 | They were given to me by a friend | | 3 | Fair |
| 6 | | 6 | | 7 | | | 6 | |
| 6 | | 6 | | 7 | | | 6 | |
| 6 | | 6 | | 7 | | | 6 | |
| 2 | 1 month but less than 6 months | 3 | Monthly | 6 | Other, please specify | Pharmacy | 3 | Fair |
| 6 | | 6 | | 7 | | | 6 | |
| 2 | 1 month but less than 6 months | 4 | Once a semester/ term | 5 | I purchased them online | | 3 | Fair |
| 3 | 6 months but less than 1 year | 2 | Weekly | 6 | Other, please specify | Pharmacy | 2 | Expensive |
| 6 | | 6 | | 7 | | | 6 | |
| 2 | 1 month but less than 6 months | 4 | Once a semester/ term | 6 | Other, please specify | Pharmacy | 2 | Expensive |
| 3 | 6 months but less than 1 year | 3 | Monthly | 5 | I purchased them online | | 3 | Fair |

| | | | | | | | | |
|---|---|---|---|---|---|---|---|---|
| 5 | 2 years and more | 1 | Daily | 6 | Other, please specify | Over the counter | 3 | Fair |
| 6 | | 6 | | 7 | | | 6 | |
| 6 | | 6 | | 7 | | | 6 | |
| 6 | | 6 | | 7 | | | 6 | |
| 3 | 6 months but less than 1 year | 2 | Weekly | 6 | Other, please specify | Pharmacy | 3 | Fair |
| 2 | 1 month but less than 6 months | 4 | Once a semester/ term | 2 | They were given to me by a friend | | 3 | Fair |
| 6 | | 6 | | 7 | | | 6 | |
| 2 | 1 month but less than 6 months | 4 | Once a semester/ term | 2 | They were given to me by a friend | | 2 | Expensive |
| 6 | | 6 | | 7 | | | 6 | |
| 3 | 6 months but less than 1 year | 4 | Once a semester/ term | 5 | I purchased them online | | 3 | Fair |
| 6 | | 6 | | 7 | | | 6 | |
| 1 | Less than 1 month | 1 | Daily | 2 | They were given to me by a friend | | 3 | Fair |
| 3 | 6 months but less than 1 year | 3 | Monthly | 5 | I purchased them online | | 2 | Expensive |

| | | | | | | | |
|---|---|---|---|---|---|---|---|
| 2 | 1 month but less than 6 months | 3 | Monthly | 5 | I purchased them online | 3 | Fair |
| 2 | 1 month but less than 6 months | 4 | Once a semester/ term | 5 | I purchased them online | 3 | Fair |

1 less month
2 1-6
3 6-12
4 1-2 year
5 less than semster
6 NA

1 daily
2 weeky
3 month
4 once semster
5 online
6 other
7 NA

1 me
2 friend
3 stranger
4 somebody else
5 v cheap
6 NA

1 v exp
2 exp
3 fair
4 cheap

| KnowSB | Q15<br>Doyouknowanyonewhousesthese(drugs/substances)? | heardFrom | Q16<br>Wherehaveyouheardaboutthesedrugs/substances? | recommend | Q17<br>Wlyourecommendthesedrugstoothers? |
|---|---|---|---|---|---|
| 3 | | 7 | | 3 | |
| 3 | | 7 | | 3 | |
| 1 | No | 6 | Other | 2 | No |
| 1 | No | 6 | Other | 2 | No |
| 3 | | 7 | | 3 | |
| 3 | | 7 | | 3 | |
| 3 | | 7 | | 3 | |
| 3 | | 7 | | 3 | |
| 3 | | 7 | | 3 | |
| 3 | | 6 | Other | 3 | |
| 3 | | 7 | | 3 | |
| 3 | | 7 | | 3 | |
| 3 | | 7 | | 3 | |
| 3 | | 7 | | 3 | |
| 3 | | 7 | | 3 | |
| 3 | | 7 | | 3 | |
| 3 | | 7 | | 3 | |
| 3 | | 7 | | 3 | |
| 1 | No | 7 | | 2 | No |
| 2 | Yes | 2 | Scientific literature | 1 | Yes |
| 3 | | 7 | | 3 | |
| 1 | No | 7 | | 3 | |
| 3 | | 7 | | 3 | |
| 2 | Yes | 6 | Other | 1 | Yes |
| 1 | No | 7 | | 3 | |
| 3 | | 7 | | 3 | |
| 2 | Yes | 2 | Scientific literature | 2 | No |
| 1 | No | 1 | Social media | 2 | No |
| 3 | | 7 | | 3 | |
| 3 | | 7 | | 3 | |
| 1 | No | 2 | Scientific literature | 1 | Yes |
| 3 | | 7 | | 3 | |
| 3 | | 7 | | 3 | |
| 3 | | 7 | | 3 | |
| 1 | No | 5 | Family | 2 | No |
| 3 | | 7 | | 2 | No |
| 3 | | 7 | | 3 | |
| 3 | | 7 | | 3 | |
| 3 | | 7 | | 3 | |
| 3 | | 7 | | 3 | |
| 3 | | 7 | | 3 | |
| 3 | | 7 | | 3 | |
| 3 | | 7 | | 3 | |
| 3 | | 7 | | 3 | |
| 2 | Yes | 1 | Social media | 2 | No |
| 3 | | 7 | | 3 | |
| 3 | | 7 | | 3 | |
| 2 | Yes | 3 | Internet | 2 | No |
| 3 | | 7 | | 3 | |
| 2 | Yes | 4 | Friends | 1 | Yes |

| | | |
|---|---|---|
| 3 | 7 | 3 |
| 3 | 7 | 3 |
| 3 | 7 | 2 No |
| 3 | 7 | 3 |
| 2 Yes | 4 Friends | 2 No |
| 3 | 7 | 3 |
| 3 | 7 | 3 |
| 2 Yes | 5 Family | 2 No |
| 3 | 7 | 3 |
| 1 No | 4 Friends | 2 No |
| 2 Yes | 4 Friends | 2 No |
| 1 No | 2 Scientific literature | 1 Yes |
| 3 | 7 | 3 |
| 3 | 7 | 3 |
| 2 Yes | 4 Friends | 1 Yes |
| 2 Yes | 4 Friends | 2 No |
| 3 | 7 | 3 |
| 3 | 7 | 3 |
| 3 | 7 | 3 |
| 3 | 7 | 3 |
| 3 | 7 | 3 |
| 3 | 7 | 3 |
| 3 | 7 | 3 |
| 3 | 7 | 3 |
| 3 | 7 | 3 |
| 3 | 7 | 3 |
| 3 | 7 | 3 |
| 1 No | 4 Friends | 1 Yes |
| 2 Yes | 4 Friends | 2 No |
| 2 Yes | 4 Friends | 1 Yes |
| 2 Yes | 4 Friends | 1 Yes |
| 3 | 7 | 3 |
| 2 Yes | 4 Friends | 2 No |
| 1 No | 1 Social media | 2 No |
| 1 No | 1 Social media | 1 Yes |
| 2 Yes | 4 Friends | 2 No |
| 1 No | 1 Social media | 2 No |
| 2 Yes | 4 Friends | 2 No |
| 1 No | 4 Friends | 3 |
| 1 No | 4 Friends | 2 No |
| 2 Yes | 4 Friends | 2 No |
| 2 Yes | 4 Friends | 2 No |
| 3 | 7 | 3 |
| 1 No | 4 Friends | 2 No |
| 2 Yes | 4 Friends | 2 No |
| 2 Yes | 4 Friends | 2 No |
| 2 Yes | 4 Friends | 2 No |
| 2 Yes | 1 Social media | 2 No |
| 3 | 7 | 3 |
| 2 Yes | 4 Friends | 2 No |
| 2 Yes | 4 Friends | 2 No |
| 2 Yes | 4 Friends | 2 No |

| | | | | | |
|---|---|---|---|---|---|
| 3 | | 7 | | 3 | |
| 3 | | 7 | | 3 | |
| 1 | No | 1 | Social media | 2 | No |
| 2 | Yes | 4 | Friends | 2 | No |
| 3 | | 7 | | 3 | |
| 2 | Yes | 4 | Friends | 2 | No |
| 2 | Yes | 4 | Friends | 2 | No |
| 3 | | 1 | Social media | 2 | No |
| 2 | Yes | 1 | Social media | 2 | No |
| 2 | Yes | 4 | Friends | 2 | No |
| 3 | | 7 | | 3 | |
| 3 | | 7 | | 3 | |
| 2 | Yes | 3 | Internet | 2 | No |
| 3 | | 7 | | 3 | |
| 3 | | 7 | | 3 | |
| 2 | Yes | 4 | Friends | 2 | No |
| 3 | | 7 | | 3 | |
| 3 | | 7 | | 3 | |
| 3 | | 7 | | 3 | |
| 3 | | 7 | | 3 | |
| 2 | Yes | 3 | Internet | 2 | No |
| 3 | | 7 | | 3 | |
| 2 | Yes | 4 | Friends | 2 | No |
| 3 | | 7 | | 3 | |
| 3 | | 7 | | 3 | |
| 3 | | 7 | | 3 | |
| 2 | Yes | 1 | Social media | 2 | No |
| 2 | Yes | 1 | Social media | 2 | No |
| 3 | | 7 | | 3 | |
| 3 | | 7 | | 3 | |
| 3 | | 7 | | 3 | |
| 3 | | 7 | | 3 | |
| 3 | | 7 | | 3 | |
| 2 | Yes | 4 | Friends | 1 | Yes |
| 3 | | 7 | | 3 | |
| 3 | | 7 | | 3 | |
| 2 | Yes | 4 | Friends | 2 | No |
| 2 | Yes | 4 | Friends | 1 | Yes |
| 3 | | 7 | | 3 | |
| 3 | | 7 | | 3 | |
| 2 | Yes | 4 | Friends | 1 | Yes |
| 2 | Yes | 4 | Friends | 1 | Yes |
| 2 | Yes | 3 | Internet | 2 | No |
| 3 | | 7 | | 3 | |
| 3 | | 7 | | 3 | |
| 3 | | 7 | | 3 | |
| 3 | | 7 | | 3 | |
| 3 | | 7 | | 3 | |
| 3 | | 7 | | 3 | |
| 3 | | 7 | | 3 | |
| 3 | | 7 | | 3 | |
| 2 | Yes | 3 | Internet | 1 | Yes |

| | | | | | |
|---|---|---|---|---|---|
| 2 | Yes | 3 | Internet | 2 | No |
| 3 | | 7 | | 3 | |
| 3 | | 7 | | 3 | |
| 3 | | 7 | | 3 | |
| 2 | Yes | 4 | Friends | 2 | No |
| 2 | Yes | 4 | Friends | 2 | No |
| 3 | | 7 | | 3 | |
| 3 | | 7 | | 3 | |
| 3 | | 7 | | 3 | |
| 3 | | 7 | | 3 | |
| 3 | | 7 | | 3 | |
| 2 | Yes | 3 | Internet | 2 | No |
| 3 | | 7 | | 3 | |
| 3 | | 7 | | 3 | |
| 3 | | 7 | | 3 | |
| 2 | Yes | 4 | Friends | 2 | No |
| 3 | | 7 | | 3 | |
| 3 | | 7 | | 3 | |
| 3 | | 7 | | 3 | |
| 3 | | 7 | | 3 | |
| 3 | | 7 | | 3 | |
| 3 | | 7 | | 3 | |
| 3 | | 7 | | 3 | |
| 2 | Yes | 1 | Social media | 2 | No |
| 3 | | 7 | | 3 | |
| 3 | | 7 | | 3 | |
| 3 | | 7 | | 3 | |
| 3 | | 7 | | 3 | |
| 3 | | 7 | | 3 | |
| 3 | | 7 | | 3 | |
| 3 | | 7 | | 3 | |
| 2 | Yes | 3 | Internet | 2 | No |
| 3 | | 7 | | 3 | |
| 2 | Yes | 3 | Internet | 2 | No |
| 3 | | 7 | | 3 | |
| 2 | Yes | 3 | Internet | 2 | No |
| 3 | | 7 | | 3 | |
| 2 | Yes | 3 | Internet | 2 | No |
| 3 | | 7 | | 3 | |
| 2 | Yes | 3 | Internet | 2 | No |
| 3 | | 7 | | 3 | |
| 3 | | 7 | | 3 | |
| 3 | | 7 | | 3 | |
| 3 | | 7 | | 3 | |
| 3 | | 7 | | 3 | |
| 3 | | 7 | | 3 | |
| 3 | | 7 | | 3 | |
| 3 | | 7 | | 3 | |
| 3 | | 7 | | 3 | |
| 3 | | 7 | | 3 | |
| 3 | | 7 | | 3 | |
| 3 | | 7 | | 3 | |

| | | | | | |
|---|---|---|---|---|---|
| 1 | No | 3 | Internet | 2 | No |
| 3 | | 7 | | 3 | |
| 3 | | 7 | | 3 | |
| 1 | No | 3 | Internet | 2 | No |
| 3 | | 7 | | 3 | |
| 3 | | 7 | | 3 | |
| 2 | Yes | 4 | Friends | 2 | No |
| 3 | | 7 | | 3 | |
| 3 | | 7 | | 3 | |
| 3 | | 7 | | 3 | |
| 3 | | 7 | | 3 | |
| 1 | No | 3 | Internet | 2 | No |
| 2 | Yes | 3 | Internet | 2 | No |
| 3 | | 7 | | 3 | |
| 3 | | 7 | | 3 | |
| 1 | No | 7 | | 2 | No |
| 3 | | 7 | | 3 | |
| 3 | | 7 | | 3 | |
| 3 | | 7 | | 3 | |
| 3 | | 7 | | 3 | |
| 3 | | 7 | | 3 | |
| 1 | No | 6 | Other | 2 | No |
| 3 | | 7 | | 3 | |
| 3 | | 7 | | 3 | |
| 3 | | 7 | | 3 | |
| 3 | | 7 | | 3 | |
| 1 | No | 7 | | 3 | |
| 3 | | 7 | | 3 | |
| 3 | | 7 | | 3 | |
| 3 | | 7 | | 3 | |
| 1 | No | 2 | Scientific literature | 2 | No |
| 1 | No | 7 | | 3 | |
| 2 | Yes | 1 | Social media | 1 | Yes |
| 3 | | 7 | | 3 | |
| 1 | No | 6 | Other | 2 | No |
| 3 | | 7 | | 3 | |
| 3 | | 7 | | 3 | |
| 3 | | 7 | | 3 | |
| 3 | | 7 | | 3 | |
| 3 | | 7 | | 3 | |
| 1 | No | 7 | | 2 | No |
| 3 | | 7 | | 3 | |
| 3 | | 7 | | 3 | |
| 3 | | 7 | | 3 | |
| 3 | | 7 | | 3 | |
| 3 | | 7 | | 3 | |
| 3 | | 7 | | 3 | |
| 3 | | 7 | | 3 | |
| 3 | | 7 | | 3 | |
| 1 | No | 3 | Internet | 2 | No |
| 3 | | 7 | | 3 | |
| 3 | | 7 | | 3 | |

| | | | | | |
|---|---|---|---|---|---|
| 3 | | 7 | | 3 | |
| 3 | | 7 | | 3 | |
| 1 | No | 3 | Internet | 2 | No |
| 3 | | 7 | | 3 | |
| 1 | No | 6 | Other | 2 | No |
| 3 | | 7 | | 3 | |
| 3 | | 7 | | 3 | |
| 3 | | 7 | | 3 | |
| 3 | | 7 | | 3 | |
| 1 | No | 5 | Family | 1 | Yes |
| 3 | | 7 | | 3 | |
| 1 | No | 7 | | 2 | No |
| 1 | No | 2 | Scientific literature | 2 | No |
| 3 | | 7 | | 3 | |
| 3 | | 7 | | 3 | |
| 3 | | 7 | | 3 | |
| 3 | | 7 | | 3 | |
| 3 | | 7 | | 3 | |
| 3 | | 7 | | 3 | |
| 3 | | 7 | | 3 | |
| 3 | | 7 | | 3 | |
| 3 | | 7 | | 3 | |
| 3 | | 7 | | 3 | |
| 3 | | 7 | | 3 | |
| 3 | | 7 | | 3 | |
| 2 | Yes | 2 | Scientific literature | 1 | Yes |
| 1 | No | 6 | Other | 2 | No |
| 3 | | 7 | | 3 | |
| 3 | | 7 | | 3 | |
| 3 | | 7 | | 3 | |
| 3 | | 7 | | 3 | |
| 3 | | 7 | | 3 | |
| 3 | | 7 | | 3 | |
| 3 | | 7 | | 3 | |
| 3 | | 7 | | 3 | |
| 3 | | 7 | | 3 | |
| 2 | Yes | 5 | Family | 2 | No |
| 3 | | 7 | | 3 | |
| 3 | | 7 | | 3 | |
| 3 | | 7 | | 3 | |
| 2 | Yes | 3 | Internet | 2 | No |
| 1 | No | 6 | Other | 2 | No |
| 2 | Yes | 5 | Family | 1 | Yes |
| 3 | | 7 | | 3 | |
| 3 | | 7 | | 3 | |
| 3 | | 7 | | 3 | |
| 3 | | 7 | | 3 | |
| 3 | | 7 | | 3 | |
| 1 | No | 2 | Scientific literature | 2 | No |
| 3 | | 7 | | 3 | |
| 3 | | 7 | | 3 | |
| 3 | | 7 | | 3 | |

| | | | | | |
|---|---|---|---|---|---|
| 3 | | 7 | | 3 | |
| 3 | | 7 | | 3 | |
| 3 | | 7 | | 3 | |
| 3 | | 7 | | 3 | |
| 3 | | 7 | | 3 | |
| 1 | No | 7 | | 2 | No |
| 3 | | 7 | | 3 | |
| 3 | | 7 | | 3 | |
| 3 | | 7 | | 3 | |
| 3 | | 7 | | 3 | |
| 3 | | 7 | | 3 | |
| 3 | | 7 | | 3 | |
| 3 | | 7 | | 3 | |
| 2 | Yes | 7 | | 3 | |
| 3 | | 7 | | 3 | |
| 1 | No | 3 | Internet | 3 | |
| 1 | No | 6 | Other | 2 | No |
| 3 | | 7 | | 3 | |
| 3 | | 7 | | 3 | |
| 3 | | 7 | | 3 | |
| 3 | | 7 | | 3 | |
| 3 | | 7 | | 3 | |
| 3 | | 7 | | 3 | |
| 3 | | 7 | | 3 | |
| 3 | | 7 | | 3 | |
| 3 | | 7 | | 3 | |
| 2 | Yes | 3 | Internet | 1 | Yes |
| 3 | | 7 | | 3 | |
| 1 | No | 1 | Social media | 2 | No |
| 2 | Yes | 6 | Other | 1 | Yes |
| 3 | | 7 | | 3 | |
| 1 | No | 7 | | 2 | No |
| 3 | | 7 | | 3 | |
| 2 | Yes | 4 | Friends | 2 | No |
| 3 | | 7 | | 3 | |
| 2 | Yes | 5 | Family | 1 | Yes |
| 2 | Yes | 5 | Family | 1 | Yes |
| 3 | | 7 | | 3 | |
| 3 | | 7 | | 3 | |
| 3 | | 7 | | 3 | |
| 2 | Yes | 1 | Social media | 2 | No |
| 3 | | 7 | | 3 | |
| 3 | | 7 | | 3 | |
| 3 | | 7 | | 3 | |
| 3 | | 7 | | 3 | |
| 1 | No | 3 | Internet | 2 | No |
| 3 | | 7 | | 3 | |
| 3 | | 7 | | 3 | |
| 3 | | 7 | | 3 | |
| 3 | | 7 | | 3 | |
| 3 | | 7 | | 3 | |
| 3 | | 7 | | 3 | |

| | | | | | |
|---|---|---|---|---|---|
| 3 | | 7 | | 3 | |
| 3 | | 7 | | 3 | |
| 3 | | 7 | | 3 | |
| 3 | | 7 | | 3 | |
| 3 | | 7 | | 3 | |
| 3 | | 7 | | 3 | |
| 3 | | 2 | Scientific literature | 3 | |
| 3 | | 7 | | 3 | |
| 3 | | 7 | | 3 | |
| 3 | | 7 | | 3 | |
| 3 | | 7 | | 3 | |
| 3 | | 7 | | 3 | |
| 3 | | 7 | | 3 | |
| 3 | | 7 | | 3 | |
| 3 | | 7 | | 3 | |
| 3 | | 7 | | 3 | |
| 3 | | 7 | | 3 | |
| 3 | | 7 | | 3 | |
| 3 | | 7 | | 3 | |
| 3 | | 7 | | 3 | |
| 3 | | 7 | | 3 | |
| 3 | | 7 | | 3 | |
| 3 | | 7 | | 3 | |
| 2 | Yes | 3 | Internet | 2 | No |
| 2 | Yes | 4 | Friends | 2 | No |
| 2 | Yes | 4 | Friends | 1 | Yes |
| 3 | | 7 | | 3 | |
| 3 | | 7 | | 3 | |
| 3 | | 7 | | 3 | |
| 3 | | 7 | | 3 | |
| 3 | | 7 | | 3 | |
| 3 | | 7 | | 3 | |
| 3 | | 7 | | 3 | |
| 3 | | 7 | | 3 | |
| 3 | | 7 | | 3 | |
| 1 | No | 7 | | 2 | No |
| 3 | | 7 | | 3 | |
| 3 | | 7 | | 3 | |
| 3 | | 7 | | 3 | |
| 3 | | 7 | | 3 | |
| 3 | | 7 | | 3 | |
| 2 | Yes | 4 | Friends | 2 | No |
| 2 | Yes | 4 | Friends | 2 | No |
| 2 | Yes | 4 | Friends | 2 | No |
| 2 | Yes | 3 | Internet | 1 | Yes |
| 2 | Yes | 3 | Internet | 1 | Yes |
| 2 | Yes | 4 | Friends | 2 | No |
| 2 | Yes | 3 | Internet | 1 | Yes |
| 2 | Yes | 3 | Internet | 1 | Yes |
| 2 | Yes | 3 | Internet | 2 | No |
| 2 | Yes | 3 | Internet | 2 | No |
| 1 | No | 1 | Social media | 2 | No |

| | | | | | |
|---|---|---|---|---|---|
| 1 | No | 1 | Social media | 2 | No |
| 3 | | 7 | | 3 | |
| 3 | | 7 | | 3 | |
| 2 | Yes | 3 | Internet | 1 | Yes |
| 3 | | 7 | | 3 | |
| 2 | Yes | 3 | Internet | 2 | No |
| 3 | | 7 | | 3 | |
| 3 | | 7 | | 3 | |
| 3 | | 7 | | 3 | |
| 3 | | 7 | | 3 | |
| 1 | No | 1 | Social media | 2 | No |
| 3 | | 7 | | 3 | |
| 3 | | 7 | | 3 | |
| 3 | | 7 | | 3 | |
| 3 | | 7 | | 3 | |
| 3 | | 7 | | 3 | |
| 3 | | 7 | | 3 | |
| 3 | | 7 | | 3 | |
| 2 | Yes | 3 | Internet | 2 | No |
| 3 | | 7 | | 3 | |
| 2 | Yes | 3 | Internet | 2 | No |
| 3 | | 7 | | 3 | |
| 3 | | 7 | | 3 | |
| 3 | | 7 | | 3 | |
| 2 | Yes | 3 | Internet | 2 | No |
| 3 | | 7 | | 3 | |
| 3 | | 7 | | 3 | |
| 3 | | 7 | | 3 | |
| 2 | Yes | 4 | Friends | 2 | No |
| 3 | | 7 | | 3 | |
| 3 | | 7 | | 3 | |
| 3 | | 7 | | 3 | |
| 3 | | 7 | | 3 | |
| 2 | Yes | 4 | Friends | 2 | No |
| 2 | Yes | 3 | Internet | 1 | Yes |
| 2 | Yes | 4 | Friends | 1 | Yes |
| 2 | Yes | 4 | Friends | 1 | Yes |
| 3 | | 7 | | 3 | |
| 3 | | 7 | | 3 | |
| 3 | | 7 | | 3 | |
| 3 | | 7 | | 3 | |
| 3 | | 7 | | 3 | |
| 3 | | 7 | | 3 | |
| 3 | | 7 | | 3 | |
| 2 | Yes | 3 | Internet | 1 | Yes |
| 3 | | 7 | | 3 | |
| 3 | | 7 | | 3 | |
| 3 | | 7 | | 3 | |
| 2 | Yes | 3 | Internet | 1 | Yes |
| 2 | Yes | 3 | Internet | 1 | Yes |
| 3 | | 7 | | 3 | |
| 3 | | 7 | | 3 | |

| | | |
|---|---|---|
| 3 | 7 | 3 |
| 3 | 7 | 3 |
| 3 | 7 | 3 |
| 3 | 7 | 3 |
| 3 | 7 | 3 |
| 3 | 7 | 3 |
| 3 | 7 | 3 |
| 3 | 7 | 3 |
| 3 | 7 | 3 |
| 3 | 7 | 3 |
| 3 | 7 | 3 |
| 3 | 7 | 3 |
| 3 | 7 | 3 |
| 3 | 7 | 3 |
| 3 | 7 | 3 |
| 3 | 7 | 3 |
| 3 | 7 | 3 |
| 3 | 7 | 3 |
| 3 | 7 | 3 |
| 1 No | 7 | 3 |
| 3 | 7 | 3 |
| 3 | 7 | 3 |
| 3 | 7 | 3 |
| 3 | 7 | 3 |
| 2 Yes | 3 Internet | 1 Yes |
| 3 | 7 | 3 |
| 1 No | 6 Other | 2 No |
| 3 | 7 | 3 |

| | | |
|---|---|---|
| 2 Yes | 4 Friends | 1 Yes |
| 3 | 7 | 3 |
| | | |
| 2 Yes | 4 Friends | 1 Yes |
| 3 | 7 | 3 |
| 3 | 7 | 3 |
| 3 | 7 | 3 |
| | | |
| 2 Yes | 4 Friends | 1 Yes |
| 3 | 7 | 3 |
| | | |
| 2 Yes | 4 Friends | 1 Yes |
| | | |
| 2 Yes | 5 Family | 1 Yes |
| 3 | 7 | 3 |
| | | |
| 2 Yes | 4 Friends | 1 Yes |
| | | |
| 2 Yes | 4 Friends | 1 Yes |

| | | | | | |
|---|---|---|---|---|---|
| 1 | No | 2 | Scientific literature | 2 | No |
| 3 | | 7 | | 3 | |
| 3 | | 7 | | 3 | |
| 3 | | 7 | | 3 | |
| 2 | Yes | 4 | Friends | 1 | Yes |
| 2 | Yes | 4 | Friends | 1 | Yes |
| 3 | | 7 | | 3 | |
| 2 | Yes | 4 | Friends | 1 | Yes |
| 3 | | 7 | | 3 | |
| 2 | Yes | 4 | Friends | 1 | Yes |
| 3 | | 7 | | 3 | |
| 2 | Yes | 2 | Scientific literature | 1 | Yes |
| 2 | Yes | 4 | Friends | 1 | Yes |

| | | |
|---|---|---|
| 2 Yes | 4 Friends | 1 Yes |
| 2 Yes | 4 Friends | 1 Yes |

1 no
2 yes
3 NA

1 SM
2 SL
3 internet
4 friends
5 family
6 other
7 NA

1 yes
2 no
3 NA

| StartDate | EndDate | Status | Progress | Duration (in seconds) |
|---|---|---|---|---|
| Start Date | End Date | Response Type | Progress | Duration (in seconds) |
| 15/03/2020 23:32 | 15/03/2020 23:33 | IP Address | 100 | 83 |
| 15/03/2020 23:32 | 15/03/2020 23:34 | IP Address | 100 | 109 |
| 15/03/2020 23:31 | 15/03/2020 23:45 | IP Address | 100 | 797 |
| 15/03/2020 23:55 | 15/03/2020 23:57 | IP Address | 100 | 121 |
| 16/03/2020 00:06 | 16/03/2020 00:07 | IP Address | 100 | 64 |
| 16/03/2020 00:08 | 16/03/2020 00:10 | IP Address | 100 | 107 |
| 16/03/2020 00:24 | 16/03/2020 00:25 | IP Address | 100 | 83 |
| 16/03/2020 00:31 | 16/03/2020 00:32 | IP Address | 100 | 60 |
| 16/03/2020 00:36 | 16/03/2020 00:40 | IP Address | 100 | 239 |
| 16/03/2020 00:44 | 16/03/2020 00:46 | IP Address | 100 | 114 |
| 16/03/2020 00:48 | 16/03/2020 00:49 | IP Address | 100 | 34 |
| 16/03/2020 00:51 | 16/03/2020 00:51 | IP Address | 100 | 32 |
| 16/03/2020 00:59 | 16/03/2020 01:02 | IP Address | 100 | 127 |
| 16/03/2020 01:18 | 16/03/2020 01:19 | IP Address | 100 | 78 |
| 16/03/2020 01:44 | 16/03/2020 01:45 | IP Address | 100 | 80 |
| 16/03/2020 02:06 | 16/03/2020 02:08 | IP Address | 100 | 134 |
| 16/03/2020 02:09 | 16/03/2020 02:10 | IP Address | 100 | 83 |
| 16/03/2020 02:11 | 16/03/2020 02:12 | IP Address | 100 | 45 |
| 16/03/2020 02:32 | 16/03/2020 02:33 | IP Address | 100 | 94 |
| 16/03/2020 03:11 | 16/03/2020 03:16 | IP Address | 100 | 300 |
| 16/03/2020 03:21 | 16/03/2020 03:24 | IP Address | 100 | 157 |
| 16/03/2020 03:28 | 16/03/2020 03:30 | IP Address | 100 | 106 |
| 16/03/2020 03:31 | 16/03/2020 03:32 | IP Address | 100 | 85 |
| 16/03/2020 04:24 | 16/03/2020 04:24 | IP Address | 100 | 44 |
| 16/03/2020 04:34 | 16/03/2020 04:36 | IP Address | 100 | 107 |
| 16/03/2020 04:41 | 16/03/2020 04:42 | IP Address | 100 | 85 |
| 16/03/2020 05:26 | 16/03/2020 05:31 | IP Address | 100 | 300 |
| 16/03/2020 05:30 | 16/03/2020 05:34 | IP Address | 100 | 187 |
| 16/03/2020 05:36 | 16/03/2020 05:37 | IP Address | 100 | 43 |
| 16/03/2020 06:21 | 16/03/2020 06:23 | IP Address | 100 | 134 |
| 16/03/2020 06:57 | 16/03/2020 07:02 | IP Address | 100 | 314 |
| 16/03/2020 07:18 | 16/03/2020 07:19 | IP Address | 100 | 38 |
| 16/03/2020 07:44 | 16/03/2020 07:45 | IP Address | 100 | 78 |
| 16/03/2020 08:44 | 16/03/2020 08:45 | IP Address | 100 | 50 |
| 16/03/2020 09:23 | 16/03/2020 09:26 | IP Address | 100 | 205 |
| 16/03/2020 09:44 | 16/03/2020 09:50 | IP Address | 100 | 366 |
| 16/03/2020 10:55 | 16/03/2020 10:56 | IP Address | 100 | 71 |
| 16/03/2020 11:58 | 16/03/2020 12:00 | IP Address | 100 | 101 |
| 16/03/2020 14:27 | 16/03/2020 14:28 | IP Address | 100 | 45 |
| 16/03/2020 17:38 | 16/03/2020 17:39 | IP Address | 100 | 63 |
| 17/03/2020 01:07 | 17/03/2020 01:09 | IP Address | 100 | 139 |
| 17/03/2020 01:55 | 17/03/2020 01:56 | IP Address | 100 | 45 |
| 17/03/2020 01:55 | 17/03/2020 01:56 | IP Address | 100 | 55 |
| 17/03/2020 01:57 | 17/03/2020 01:57 | IP Address | 100 | 18 |
| 17/03/2020 01:55 | 17/03/2020 01:57 | IP Address | 100 | 103 |
| 17/03/2020 01:57 | 17/03/2020 01:58 | IP Address | 100 | 69 |
| 17/03/2020 02:13 | 17/03/2020 02:13 | IP Address | 100 | 35 |

| | | | | |
|---|---|---|---|---|
| 17/03/2020 03:01 | 17/03/2020 03:04 | IP Address | 100 | 172 |
| 17/03/2020 03:06 | 17/03/2020 03:07 | IP Address | 100 | 38 |
| 17/03/2020 03:05 | 17/03/2020 03:07 | IP Address | 100 | 121 |
| 17/03/2020 03:43 | 17/03/2020 03:52 | IP Address | 100 | 582 |
| 17/03/2020 04:30 | 17/03/2020 04:32 | IP Address | 100 | 103 |
| 17/03/2020 04:37 | 17/03/2020 04:38 | IP Address | 100 | 61 |
| 17/03/2020 04:48 | 17/03/2020 04:48 | IP Address | 100 | 36 |
| 17/03/2020 05:27 | 17/03/2020 05:33 | IP Address | 100 | 314 |
| 17/03/2020 05:56 | 17/03/2020 05:57 | IP Address | 100 | 71 |
| 17/03/2020 07:09 | 17/03/2020 07:09 | IP Address | 100 | 47 |
| 17/03/2020 07:46 | 17/03/2020 07:53 | IP Address | 100 | 401 |
| 17/03/2020 09:32 | 17/03/2020 09:34 | IP Address | 100 | 98 |
| 17/03/2020 12:58 | 17/03/2020 13:05 | IP Address | 100 | 383 |
| 18/03/2020 01:22 | 18/03/2020 01:26 | IP Address | 100 | 221 |
| 18/03/2020 10:15 | 18/03/2020 10:19 | IP Address | 100 | 254 |
| 19/03/2020 01:25 | 19/03/2020 01:26 | IP Address | 100 | 64 |
| 16/03/2020 03:54 | 19/03/2020 09:06 | IP Address | 100 | 277877 |
| 19/03/2020 15:47 | 19/03/2020 15:50 | IP Address | 100 | 188 |
| 20/03/2020 15:18 | 20/03/2020 15:21 | IP Address | 100 | 165 |
| 22/03/2020 03:32 | 22/03/2020 03:33 | IP Address | 100 | 32 |
| 22/03/2020 03:41 | 22/03/2020 03:42 | IP Address | 100 | 83 |
| 22/03/2020 03:42 | 22/03/2020 03:43 | IP Address | 100 | 58 |
| 22/03/2020 03:47 | 22/03/2020 03:52 | IP Address | 100 | 284 |
| 22/03/2020 04:05 | 22/03/2020 04:19 | IP Address | 100 | 836 |
| 22/03/2020 05:30 | 22/03/2020 05:32 | IP Address | 100 | 106 |
| 22/03/2020 06:13 | 22/03/2020 06:14 | IP Address | 100 | 46 |
| 22/03/2020 07:11 | 22/03/2020 07:12 | IP Address | 100 | 58 |
| 23/03/2020 01:21 | 23/03/2020 01:23 | IP Address | 100 | 127 |
| 23/03/2020 09:19 | 23/03/2020 09:20 | IP Address | 100 | 68 |
| 20/03/2020 23:31 | 24/03/2020 10:08 | IP Address | 100 | 297445 |
| 26/03/2020 12:48 | 26/03/2020 12:50 | IP Address | 100 | 123 |
| 28/03/2020 03:08 | 28/03/2020 03:08 | Spam | 100 | 4 |
| 01/04/2020 16:21 | 01/04/2020 16:21 | Spam | 100 | 7 |
| 01/04/2020 16:21 | 01/04/2020 16:21 | Spam | 100 | 5 |
| 01/04/2020 17:30 | 01/04/2020 17:31 | IP Address | 100 | 101 |
| 04/04/2020 18:00 | 04/04/2020 18:03 | IP Address | 100 | 218 |
| 07/04/2020 13:07 | 07/04/2020 13:10 | IP Address | 100 | 122 |
| 12/04/2020 08:36 | 12/04/2020 08:36 | IP Address | 100 | 38 |
| 12/04/2020 08:38 | 12/04/2020 08:40 | IP Address | 100 | 90 |
| 12/04/2020 08:44 | 12/04/2020 08:45 | IP Address | 100 | 106 |
| 12/04/2020 08:46 | 12/04/2020 08:47 | IP Address | 100 | 71 |
| 12/04/2020 08:47 | 12/04/2020 08:50 | IP Address | 100 | 179 |
| 12/04/2020 08:52 | 12/04/2020 08:55 | IP Address | 100 | 194 |
| 12/04/2020 08:56 | 12/04/2020 08:57 | IP Address | 100 | 62 |
| 12/04/2020 09:00 | 12/04/2020 09:01 | IP Address | 100 | 54 |
| 12/04/2020 09:04 | 12/04/2020 09:05 | IP Address | 100 | 62 |
| 12/04/2020 09:05 | 12/04/2020 09:07 | IP Address | 100 | 88 |
| 12/04/2020 15:15 | 12/04/2020 15:18 | IP Address | 100 | 166 |

| | | | | |
|---|---|---|---|---|
| 12/04/2020 15:18 | 12/04/2020 15:21 | IP Address | 100 | 141 |
| 12/04/2020 15:23 | 12/04/2020 15:24 | IP Address | 100 | 81 |
| 12/04/2020 15:24 | 12/04/2020 15:26 | IP Address | 100 | 100 |
| 12/04/2020 15:26 | 12/04/2020 15:27 | IP Address | 100 | 61 |
| 13/04/2020 08:23 | 13/04/2020 08:24 | IP Address | 100 | 70 |
| 13/04/2020 08:24 | 13/04/2020 08:25 | IP Address | 100 | 54 |
| 13/04/2020 08:25 | 13/04/2020 08:26 | IP Address | 100 | 25 |
| 14/04/2020 07:38 | 14/04/2020 07:39 | IP Address | 100 | 75 |
| 14/04/2020 07:40 | 14/04/2020 07:40 | IP Address | 100 | 49 |
| 14/04/2020 09:15 | 14/04/2020 09:16 | IP Address | 100 | 78 |
| 14/04/2020 09:17 | 14/04/2020 09:17 | IP Address | 100 | 27 |
| 14/04/2020 09:17 | 14/04/2020 09:17 | IP Address | 100 | 11 |
| 14/04/2020 09:17 | 14/04/2020 09:19 | IP Address | 100 | 88 |
| 14/04/2020 09:19 | 14/04/2020 09:20 | IP Address | 100 | 62 |
| 14/04/2020 09:30 | 14/04/2020 09:30 | IP Address | 100 | 11 |
| 14/04/2020 09:33 | 14/04/2020 09:35 | IP Address | 100 | 88 |
| 14/04/2020 12:44 | 14/04/2020 12:45 | IP Address | 100 | 51 |
| 14/04/2020 16:34 | 14/04/2020 16:39 | IP Address | 100 | 328 |
| 15/04/2020 07:07 | 15/04/2020 07:07 | IP Address | 100 | 56 |
| 15/04/2020 07:34 | 15/04/2020 07:35 | IP Address | 100 | 63 |
| 15/04/2020 07:35 | 15/04/2020 07:35 | IP Address | 100 | 12 |
| 15/04/2020 07:35 | 15/04/2020 07:35 | IP Address | 100 | 11 |
| 15/04/2020 07:35 | 15/04/2020 07:36 | IP Address | 100 | 50 |
| 15/04/2020 07:36 | 15/04/2020 07:37 | IP Address | 100 | 13 |
| 15/04/2020 07:37 | 15/04/2020 07:37 | IP Address | 100 | 11 |
| 15/04/2020 07:40 | 15/04/2020 07:41 | IP Address | 100 | 56 |
| 15/04/2020 07:41 | 15/04/2020 07:41 | IP Address | 100 | 14 |
| 15/04/2020 07:41 | 15/04/2020 07:42 | IP Address | 100 | 11 |
| 15/04/2020 07:42 | 15/04/2020 07:42 | IP Address | 100 | 10 |
| 15/04/2020 07:44 | 15/04/2020 07:44 | IP Address | 100 | 12 |
| 15/04/2020 07:44 | 15/04/2020 07:45 | IP Address | 100 | 62 |
| 15/04/2020 07:45 | 15/04/2020 07:46 | IP Address | 100 | 12 |
| 15/04/2020 07:47 | 15/04/2020 07:48 | IP Address | 100 | 60 |
| 15/04/2020 07:49 | 15/04/2020 07:49 | IP Address | 100 | 21 |
| 15/04/2020 10:08 | 15/04/2020 10:08 | IP Address | 100 | 4 |
| 15/04/2020 10:08 | 15/04/2020 10:08 | IP Address | 100 | 4 |
| 15/04/2020 18:54 | 15/04/2020 18:55 | IP Address | 100 | 43 |
| 16/04/2020 18:27 | 16/04/2020 18:28 | IP Address | 100 | 79 |
| 16/04/2020 18:28 | 16/04/2020 18:29 | IP Address | 100 | 29 |
| 16/04/2020 18:29 | 16/04/2020 18:29 | IP Address | 100 | 11 |
| 16/04/2020 18:29 | 16/04/2020 18:29 | IP Address | 100 | 15 |
| 16/04/2020 18:30 | 16/04/2020 18:30 | IP Address | 100 | 10 |
| 16/04/2020 18:30 | 16/04/2020 18:30 | IP Address | 100 | 12 |
| 16/04/2020 18:31 | 16/04/2020 18:32 | IP Address | 100 | 93 |
| 16/04/2020 18:32 | 16/04/2020 18:32 | IP Address | 100 | 10 |
| 16/04/2020 23:19 | 16/04/2020 23:20 | IP Address | 100 | 75 |
| 21/04/2020 13:20 | 21/04/2020 13:21 | IP Address | 100 | 75 |
| 26/04/2020 15:31 | 26/04/2020 15:32 | IP Address | 100 | 75 |

| | | | | |
|---|---|---|---|---|
| 26/04/2020 15:33 | 26/04/2020 15:33 | IP Address | 100 | 11 |
| 26/04/2020 15:36 | 26/04/2020 15:36 | IP Address | 100 | 11 |
| 26/04/2020 15:36 | 26/04/2020 15:38 | IP Address | 100 | 90 |
| 26/04/2020 15:38 | 26/04/2020 15:41 | IP Address | 100 | 131 |
| 26/04/2020 15:41 | 26/04/2020 15:42 | IP Address | 100 | 50 |
| 26/04/2020 15:42 | 26/04/2020 15:42 | IP Address | 100 | 11 |
| 26/04/2020 17:04 | 26/04/2020 17:05 | IP Address | 100 | 19 |
| 26/04/2020 18:42 | 26/04/2020 18:42 | Spam | 100 | 4 |
| 26/04/2020 18:42 | 26/04/2020 18:42 | Spam | 100 | 5 |
| 26/04/2020 19:01 | 26/04/2020 19:01 | IP Address | 100 | 12 |
| 26/04/2020 19:01 | 26/04/2020 19:01 | IP Address | 100 | 12 |
| 26/04/2020 19:03 | 26/04/2020 19:03 | IP Address | 100 | 17 |
| 26/04/2020 19:36 | 26/04/2020 19:36 | IP Address | 100 | 13 |
| 26/04/2020 19:36 | 26/04/2020 19:37 | IP Address | 100 | 11 |
| 26/04/2020 19:37 | 26/04/2020 19:37 | IP Address | 100 | 10 |
| 26/04/2020 19:37 | 26/04/2020 19:38 | IP Address | 100 | 53 |
| 28/04/2020 09:43 | 28/04/2020 09:44 | IP Address | 100 | 56 |
| 28/04/2020 09:44 | 28/04/2020 09:44 | IP Address | 100 | 9 |
| 28/04/2020 09:44 | 28/04/2020 09:44 | IP Address | 100 | 8 |
| 28/04/2020 09:44 | 28/04/2020 09:44 | IP Address | 100 | 8 |
| 28/04/2020 09:45 | 28/04/2020 09:45 | IP Address | 100 | 47 |
| 30/04/2020 18:11 | 30/04/2020 18:12 | IP Address | 100 | 84 |
| 30/04/2020 18:12 | 30/04/2020 18:12 | IP Address | 100 | 11 |
| 30/04/2020 18:13 | 30/04/2020 18:13 | IP Address | 100 | 10 |
| 30/04/2020 18:13 | 30/04/2020 18:13 | IP Address | 100 | 9 |
| 30/04/2020 18:14 | 30/04/2020 18:14 | IP Address | 100 | 12 |
| 30/04/2020 18:14 | 30/04/2020 18:14 | IP Address | 100 | 14 |
| 01/05/2020 19:43 | 01/05/2020 19:44 | IP Address | 100 | 83 |
| 01/05/2020 19:45 | 01/05/2020 19:45 | IP Address | 100 | 12 |
| 01/05/2020 19:45 | 01/05/2020 19:45 | IP Address | 100 | 10 |
| 01/05/2020 19:45 | 01/05/2020 19:45 | IP Address | 100 | 9 |
| 02/05/2020 06:28 | 02/05/2020 06:29 | IP Address | 100 | 81 |
| 02/05/2020 06:29 | 02/05/2020 06:30 | IP Address | 100 | 11 |
| 02/05/2020 06:30 | 02/05/2020 06:30 | IP Address | 100 | 10 |
| 02/05/2020 06:30 | 02/05/2020 06:30 | IP Address | 100 | 11 |
| 02/05/2020 06:31 | 02/05/2020 06:31 | IP Address | 100 | 10 |
| 02/05/2020 06:51 | 02/05/2020 06:51 | IP Address | 100 | 12 |
| 02/05/2020 08:11 | 02/05/2020 08:17 | IP Address | 100 | 307 |
| 02/05/2020 08:28 | 02/05/2020 08:29 | IP Address | 100 | 92 |
| 02/05/2020 08:29 | 02/05/2020 08:32 | IP Address | 100 | 177 |
| 02/05/2020 08:55 | 02/05/2020 08:55 | IP Address | 100 | 13 |
| 05/05/2020 21:06 | 05/05/2020 21:06 | IP Address | 100 | 17 |
| 05/05/2020 21:06 | 05/05/2020 21:06 | IP Address | 100 | 10 |
| 05/05/2020 21:06 | 05/05/2020 21:06 | IP Address | 100 | 9 |
| 05/05/2020 21:06 | 05/05/2020 21:07 | IP Address | 100 | 11 |
| 05/05/2020 21:07 | 05/05/2020 21:07 | IP Address | 100 | 11 |
| 08/05/2020 18:12 | 08/05/2020 18:13 | IP Address | 100 | 23 |
| 08/05/2020 18:13 | 08/05/2020 18:14 | IP Address | 100 | 65 |

| | | | | |
|---|---|---|---|---|
| 08/05/2020 18:14 | 08/05/2020 18:14 | IP Address | 100 | 14 |
| 08/05/2020 18:15 | 08/05/2020 18:16 | IP Address | 100 | 80 |
| 08/05/2020 18:16 | 08/05/2020 18:17 | IP Address | 100 | 11 |
| 10/05/2020 10:40 | 10/05/2020 10:42 | IP Address | 100 | 72 |
| 10/05/2020 10:42 | 10/05/2020 10:42 | IP Address | 100 | 10 |
| 10/05/2020 10:42 | 10/05/2020 10:43 | IP Address | 100 | 71 |
| 10/05/2020 10:44 | 10/05/2020 10:45 | IP Address | 100 | 10 |
| 10/05/2020 10:45 | 10/05/2020 10:46 | IP Address | 100 | 101 |
| 12/05/2020 02:34 | 12/05/2020 02:35 | IP Address | 100 | 36 |
| 12/05/2020 02:34 | 12/05/2020 02:35 | IP Address | 100 | 47 |
| 12/05/2020 02:34 | 12/05/2020 02:35 | IP Address | 100 | 47 |
| 12/05/2020 02:34 | 12/05/2020 02:35 | IP Address | 100 | 55 |
| 12/05/2020 02:34 | 12/05/2020 02:35 | IP Address | 100 | 66 |
| 12/05/2020 02:34 | 12/05/2020 02:35 | IP Address | 100 | 58 |
| 12/05/2020 02:35 | 12/05/2020 02:35 | IP Address | 100 | 36 |
| 12/05/2020 02:35 | 12/05/2020 02:38 | IP Address | 100 | 130 |
| 12/05/2020 02:37 | 12/05/2020 02:38 | IP Address | 100 | 57 |
| 12/05/2020 02:37 | 12/05/2020 02:38 | IP Address | 100 | 74 |
| 12/05/2020 02:37 | 12/05/2020 02:38 | IP Address | 100 | 87 |
| 12/05/2020 02:38 | 12/05/2020 02:39 | IP Address | 100 | 37 |
| 12/05/2020 02:37 | 12/05/2020 02:39 | IP Address | 100 | 127 |
| 12/05/2020 02:38 | 12/05/2020 02:39 | IP Address | 100 | 44 |
| 12/05/2020 02:34 | 12/05/2020 02:39 | IP Address | 100 | 332 |
| 12/05/2020 02:39 | 12/05/2020 02:40 | IP Address | 100 | 68 |
| 12/05/2020 02:38 | 12/05/2020 02:41 | IP Address | 100 | 191 |
| 12/05/2020 02:41 | 12/05/2020 02:41 | IP Address | 100 | 56 |
| 12/05/2020 02:37 | 12/05/2020 02:42 | IP Address | 100 | 324 |
| 12/05/2020 02:35 | 12/05/2020 02:43 | IP Address | 100 | 457 |
| 12/05/2020 02:42 | 12/05/2020 02:44 | IP Address | 100 | 122 |
| 12/05/2020 02:43 | 12/05/2020 02:45 | IP Address | 100 | 155 |
| 12/05/2020 02:45 | 12/05/2020 02:46 | IP Address | 100 | 45 |
| 12/05/2020 02:41 | 12/05/2020 02:46 | IP Address | 100 | 320 |
| 12/05/2020 02:44 | 12/05/2020 02:46 | IP Address | 100 | 168 |
| 12/05/2020 02:45 | 12/05/2020 02:46 | IP Address | 100 | 67 |
| 12/05/2020 02:45 | 12/05/2020 02:47 | IP Address | 100 | 112 |
| 12/05/2020 02:45 | 12/05/2020 02:47 | IP Address | 100 | 99 |
| 12/05/2020 02:48 | 12/05/2020 02:49 | IP Address | 100 | 78 |
| 12/05/2020 02:44 | 12/05/2020 02:50 | IP Address | 100 | 333 |
| 12/05/2020 02:49 | 12/05/2020 02:50 | IP Address | 100 | 72 |
| 12/05/2020 02:50 | 12/05/2020 02:51 | IP Address | 100 | 44 |
| 12/05/2020 02:53 | 12/05/2020 02:54 | IP Address | 100 | 38 |
| 12/05/2020 02:52 | 12/05/2020 02:54 | IP Address | 100 | 148 |
| 12/05/2020 02:55 | 12/05/2020 02:57 | IP Address | 100 | 129 |
| 12/05/2020 02:56 | 12/05/2020 02:58 | IP Address | 100 | 116 |
| 12/05/2020 02:57 | 12/05/2020 02:58 | IP Address | 100 | 35 |
| 12/05/2020 02:58 | 12/05/2020 03:00 | IP Address | 100 | 81 |
| 12/05/2020 02:59 | 12/05/2020 03:01 | IP Address | 100 | 118 |
| 12/05/2020 03:01 | 12/05/2020 03:03 | IP Address | 100 | 90 |

| | | | | |
|---|---|---|---|---|
| 12/05/2020 03:02 | 12/05/2020 03:03 | IP Address | 100 | 35 |
| 12/05/2020 03:02 | 12/05/2020 03:03 | IP Address | 100 | 76 |
| 12/05/2020 03:00 | 12/05/2020 03:04 | IP Address | 100 | 249 |
| 12/05/2020 03:05 | 12/05/2020 03:06 | IP Address | 100 | 90 |
| 12/05/2020 03:03 | 12/05/2020 03:07 | IP Address | 100 | 251 |
| 12/05/2020 03:07 | 12/05/2020 03:08 | IP Address | 100 | 49 |
| 12/05/2020 03:03 | 12/05/2020 03:10 | IP Address | 100 | 373 |
| 12/05/2020 03:09 | 12/05/2020 03:10 | IP Address | 100 | 51 |
| 12/05/2020 03:15 | 12/05/2020 03:19 | IP Address | 100 | 207 |
| 12/05/2020 03:19 | 12/05/2020 03:20 | IP Address | 100 | 45 |
| 12/05/2020 03:20 | 12/05/2020 03:21 | IP Address | 100 | 64 |
| 12/05/2020 03:20 | 12/05/2020 03:21 | IP Address | 100 | 62 |
| 12/05/2020 03:21 | 12/05/2020 03:23 | IP Address | 100 | 95 |
| 12/05/2020 03:26 | 12/05/2020 03:28 | IP Address | 100 | 150 |
| 12/05/2020 03:32 | 12/05/2020 03:33 | IP Address | 100 | 80 |
| 12/05/2020 03:30 | 12/05/2020 03:33 | IP Address | 100 | 196 |
| 12/05/2020 03:35 | 12/05/2020 03:35 | IP Address | 100 | 39 |
| 12/05/2020 03:38 | 12/05/2020 03:39 | IP Address | 100 | 72 |
| 12/05/2020 03:40 | 12/05/2020 03:41 | IP Address | 100 | 91 |
| 12/05/2020 03:40 | 12/05/2020 03:41 | IP Address | 100 | 82 |
| 12/05/2020 03:42 | 12/05/2020 03:43 | IP Address | 100 | 95 |
| 12/05/2020 03:37 | 12/05/2020 03:44 | IP Address | 100 | 426 |
| 12/05/2020 03:52 | 12/05/2020 03:53 | IP Address | 100 | 38 |
| 12/05/2020 03:53 | 12/05/2020 03:54 | IP Address | 100 | 112 |
| 12/05/2020 03:54 | 12/05/2020 03:55 | IP Address | 100 | 79 |
| 12/05/2020 03:58 | 12/05/2020 04:00 | IP Address | 100 | 71 |
| 12/05/2020 04:17 | 12/05/2020 04:19 | IP Address | 100 | 142 |
| 12/05/2020 04:20 | 12/05/2020 04:21 | IP Address | 100 | 92 |
| 12/05/2020 04:20 | 12/05/2020 04:21 | IP Address | 100 | 100 |
| 12/05/2020 04:22 | 12/05/2020 04:22 | IP Address | 100 | 13 |
| 12/05/2020 04:24 | 12/05/2020 04:24 | IP Address | 100 | 35 |
| 12/05/2020 04:30 | 12/05/2020 04:31 | IP Address | 100 | 86 |
| 12/05/2020 04:41 | 12/05/2020 04:43 | IP Address | 100 | 77 |
| 12/05/2020 04:42 | 12/05/2020 04:47 | IP Address | 100 | 278 |
| 12/05/2020 04:48 | 12/05/2020 04:49 | IP Address | 100 | 90 |
| 12/05/2020 04:53 | 12/05/2020 04:54 | IP Address | 100 | 85 |
| 12/05/2020 04:54 | 12/05/2020 04:58 | IP Address | 100 | 228 |
| 12/05/2020 04:58 | 12/05/2020 05:01 | IP Address | 100 | 194 |
| 12/05/2020 05:08 | 12/05/2020 05:09 | IP Address | 100 | 75 |
| 12/05/2020 05:18 | 12/05/2020 05:18 | IP Address | 100 | 47 |
| 12/05/2020 05:09 | 12/05/2020 05:19 | IP Address | 100 | 582 |
| 12/05/2020 05:19 | 12/05/2020 05:20 | IP Address | 100 | 49 |
| 12/05/2020 05:19 | 12/05/2020 05:20 | IP Address | 100 | 72 |
| 12/05/2020 05:24 | 12/05/2020 05:26 | IP Address | 100 | 170 |
| 12/05/2020 05:28 | 12/05/2020 05:30 | IP Address | 100 | 106 |
| 12/05/2020 05:32 | 12/05/2020 05:34 | IP Address | 100 | 111 |
| 12/05/2020 05:39 | 12/05/2020 05:41 | IP Address | 100 | 75 |
| 12/05/2020 05:41 | 12/05/2020 05:42 | IP Address | 100 | 83 |

| | | | | |
|---|---|---|---|---|
| 12/05/2020 05:44 | 12/05/2020 05:44 | IP Address | 100 | 30 |
| 12/05/2020 05:38 | 12/05/2020 05:44 | IP Address | 100 | 387 |
| 12/05/2020 05:44 | 12/05/2020 05:48 | IP Address | 100 | 286 |
| 12/05/2020 05:56 | 12/05/2020 05:58 | IP Address | 100 | 138 |
| 12/05/2020 06:31 | 12/05/2020 06:32 | IP Address | 100 | 47 |
| 12/05/2020 06:33 | 12/05/2020 06:34 | IP Address | 100 | 56 |
| 12/05/2020 06:32 | 12/05/2020 06:35 | IP Address | 100 | 187 |
| 12/05/2020 06:33 | 12/05/2020 06:35 | IP Address | 100 | 118 |
| 12/05/2020 06:33 | 12/05/2020 06:36 | IP Address | 100 | 159 |
| 12/05/2020 06:45 | 12/05/2020 06:45 | IP Address | 100 | 39 |
| 12/05/2020 06:48 | 12/05/2020 06:49 | IP Address | 100 | 88 |
| 12/05/2020 06:47 | 12/05/2020 06:49 | IP Address | 100 | 109 |
| 12/05/2020 06:49 | 12/05/2020 06:51 | IP Address | 100 | 120 |
| 12/05/2020 06:53 | 12/05/2020 06:54 | IP Address | 100 | 84 |
| 12/05/2020 06:40 | 12/05/2020 07:05 | IP Address | 100 | 1510 |
| 12/05/2020 07:05 | 12/05/2020 07:06 | IP Address | 100 | 99 |
| 12/05/2020 07:02 | 12/05/2020 07:07 | IP Address | 100 | 252 |
| 12/05/2020 07:02 | 12/05/2020 07:10 | IP Address | 100 | 458 |
| 12/05/2020 07:04 | 12/05/2020 07:17 | IP Address | 100 | 813 |
| 12/05/2020 07:30 | 12/05/2020 07:32 | IP Address | 100 | 142 |
| 12/05/2020 07:34 | 12/05/2020 07:34 | IP Address | 100 | 39 |
| 12/05/2020 07:44 | 12/05/2020 07:45 | IP Address | 100 | 99 |
| 12/05/2020 07:53 | 12/05/2020 07:54 | IP Address | 100 | 97 |
| 12/05/2020 07:54 | 12/05/2020 07:56 | IP Address | 100 | 154 |
| 12/05/2020 08:07 | 12/05/2020 08:10 | IP Address | 100 | 190 |
| 12/05/2020 08:12 | 12/05/2020 08:14 | IP Address | 100 | 110 |
| 12/05/2020 08:31 | 12/05/2020 08:34 | IP Address | 100 | 126 |
| 12/05/2020 08:28 | 12/05/2020 08:34 | IP Address | 100 | 379 |
| 12/05/2020 09:10 | 12/05/2020 09:12 | IP Address | 100 | 77 |
| 12/05/2020 09:15 | 12/05/2020 09:16 | IP Address | 100 | 64 |
| 12/05/2020 09:18 | 12/05/2020 09:19 | IP Address | 100 | 76 |
| 12/05/2020 09:25 | 12/05/2020 09:26 | IP Address | 100 | 68 |
| 12/05/2020 09:27 | 12/05/2020 09:28 | IP Address | 100 | 88 |
| 12/05/2020 09:35 | 12/05/2020 09:37 | IP Address | 100 | 141 |
| 12/05/2020 09:37 | 12/05/2020 09:37 | IP Address | 100 | 37 |
| 12/05/2020 09:38 | 12/05/2020 09:38 | IP Address | 100 | 29 |
| 12/05/2020 09:40 | 12/05/2020 09:41 | IP Address | 100 | 35 |
| 12/05/2020 10:13 | 12/05/2020 10:14 | IP Address | 100 | 77 |
| 12/05/2020 10:59 | 12/05/2020 11:00 | IP Address | 100 | 42 |
| 12/05/2020 11:04 | 12/05/2020 11:04 | IP Address | 100 | 47 |
| 12/05/2020 11:01 | 12/05/2020 11:05 | IP Address | 100 | 213 |
| 12/05/2020 11:07 | 12/05/2020 11:09 | IP Address | 100 | 99 |
| 12/05/2020 11:09 | 12/05/2020 11:10 | IP Address | 100 | 46 |
| 12/05/2020 11:16 | 12/05/2020 11:19 | IP Address | 100 | 192 |
| 12/05/2020 11:33 | 12/05/2020 11:36 | IP Address | 100 | 180 |
| 12/05/2020 11:37 | 12/05/2020 11:39 | IP Address | 100 | 84 |
| 12/05/2020 11:48 | 12/05/2020 11:50 | IP Address | 100 | 92 |
| 12/05/2020 12:03 | 12/05/2020 12:04 | IP Address | 100 | 77 |

| | | | | |
|---|---|---|---|---|
| 12/05/2020 12:28 | 12/05/2020 12:33 | IP Address | 100 | 265 |
| 12/05/2020 12:41 | 12/05/2020 12:43 | IP Address | 100 | 74 |
| 12/05/2020 12:45 | 12/05/2020 12:47 | IP Address | 100 | 73 |
| 12/05/2020 13:07 | 12/05/2020 13:25 | IP Address | 100 | 1058 |
| 12/05/2020 13:59 | 12/05/2020 14:00 | IP Address | 100 | 59 |
| 12/05/2020 13:59 | 12/05/2020 14:01 | IP Address | 100 | 120 |
| 12/05/2020 14:08 | 12/05/2020 14:10 | IP Address | 100 | 167 |
| 12/05/2020 14:15 | 12/05/2020 14:18 | IP Address | 100 | 128 |
| 12/05/2020 14:41 | 12/05/2020 14:46 | IP Address | 100 | 301 |
| 12/05/2020 14:49 | 12/05/2020 14:52 | IP Address | 100 | 169 |
| 12/05/2020 15:56 | 12/05/2020 15:58 | IP Address | 100 | 90 |
| 12/05/2020 17:00 | 12/05/2020 17:03 | IP Address | 100 | 197 |
| 12/05/2020 17:25 | 12/05/2020 17:25 | IP Address | 100 | 14 |
| 12/05/2020 17:25 | 12/05/2020 17:26 | IP Address | 100 | 65 |
| 12/05/2020 17:27 | 12/05/2020 17:27 | IP Address | 100 | 12 |
| 12/05/2020 18:00 | 12/05/2020 18:07 | IP Address | 100 | 385 |
| 12/05/2020 18:19 | 12/05/2020 18:21 | IP Address | 100 | 104 |
| 12/05/2020 18:34 | 12/05/2020 18:35 | IP Address | 100 | 50 |
| 12/05/2020 19:16 | 12/05/2020 19:19 | IP Address | 100 | 191 |
| 12/05/2020 19:36 | 12/05/2020 19:37 | IP Address | 100 | 35 |
| 12/05/2020 19:55 | 12/05/2020 19:58 | IP Address | 100 | 156 |
| 12/05/2020 20:57 | 12/05/2020 20:59 | IP Address | 100 | 90 |
| 12/05/2020 21:05 | 12/05/2020 21:08 | IP Address | 100 | 166 |
| 12/05/2020 22:41 | 12/05/2020 22:42 | IP Address | 100 | 74 |
| 12/05/2020 22:55 | 12/05/2020 22:56 | IP Address | 100 | 73 |
| 12/05/2020 23:01 | 12/05/2020 23:04 | IP Address | 100 | 208 |
| 12/05/2020 23:52 | 12/05/2020 23:54 | IP Address | 100 | 152 |
| 13/05/2020 00:43 | 13/05/2020 00:45 | IP Address | 100 | 93 |
| 13/05/2020 00:46 | 13/05/2020 01:02 | IP Address | 100 | 990 |
| 13/05/2020 01:35 | 13/05/2020 01:39 | IP Address | 100 | 222 |
| 13/05/2020 01:59 | 13/05/2020 02:03 | IP Address | 100 | 208 |
| 13/05/2020 02:20 | 13/05/2020 02:22 | IP Address | 100 | 97 |
| 13/05/2020 02:56 | 13/05/2020 02:57 | IP Address | 100 | 49 |
| 13/05/2020 02:56 | 13/05/2020 02:58 | IP Address | 100 | 109 |
| 13/05/2020 03:16 | 13/05/2020 03:17 | IP Address | 100 | 31 |
| 13/05/2020 03:33 | 13/05/2020 03:36 | IP Address | 100 | 142 |
| 13/05/2020 03:47 | 13/05/2020 03:48 | IP Address | 100 | 55 |
| 13/05/2020 03:57 | 13/05/2020 04:01 | IP Address | 100 | 267 |
| 13/05/2020 03:59 | 13/05/2020 04:02 | IP Address | 100 | 188 |
| 13/05/2020 04:14 | 13/05/2020 04:15 | IP Address | 100 | 27 |
| 13/05/2020 05:27 | 13/05/2020 05:30 | IP Address | 100 | 154 |
| 13/05/2020 06:36 | 13/05/2020 06:44 | IP Address | 100 | 515 |
| 13/05/2020 07:21 | 13/05/2020 07:26 | IP Address | 100 | 265 |
| 13/05/2020 07:53 | 13/05/2020 07:57 | IP Address | 100 | 237 |
| 13/05/2020 08:50 | 13/05/2020 08:52 | IP Address | 100 | 97 |
| 13/05/2020 09:44 | 13/05/2020 09:46 | IP Address | 100 | 79 |
| 13/05/2020 09:49 | 13/05/2020 09:50 | IP Address | 100 | 57 |
| 13/05/2020 10:58 | 13/05/2020 10:59 | IP Address | 100 | 60 |

| | | | | |
|---|---|---|---|---|
| 13/05/2020 11:22 | 13/05/2020 11:23 | IP Address | 100 | 66 |
| 13/05/2020 12:06 | 13/05/2020 12:08 | IP Address | 100 | 95 |
| 13/05/2020 12:38 | 13/05/2020 12:40 | IP Address | 100 | 91 |
| 13/05/2020 14:16 | 13/05/2020 14:17 | IP Address | 100 | 77 |
| 13/05/2020 14:36 | 13/05/2020 14:37 | IP Address | 100 | 18 |
| 13/05/2020 14:37 | 13/05/2020 14:37 | IP Address | 100 | 10 |
| 13/05/2020 14:46 | 13/05/2020 14:46 | IP Address | 100 | 37 |
| 13/05/2020 15:07 | 13/05/2020 15:31 | Spam | 100 | 1422 |
| 13/05/2020 17:20 | 13/05/2020 17:22 | IP Address | 100 | 111 |
| 13/05/2020 17:24 | 13/05/2020 17:27 | IP Address | 100 | 163 |
| 13/05/2020 17:40 | 13/05/2020 17:42 | IP Address | 100 | 65 |
| 13/05/2020 21:00 | 13/05/2020 21:02 | IP Address | 100 | 117 |
| 13/05/2020 22:40 | 13/05/2020 22:41 | IP Address | 100 | 72 |
| 13/05/2020 22:49 | 13/05/2020 22:50 | IP Address | 100 | 71 |
| 14/05/2020 00:31 | 14/05/2020 00:33 | IP Address | 100 | 137 |
| 14/05/2020 01:23 | 14/05/2020 01:24 | IP Address | 100 | 113 |
| 14/05/2020 01:46 | 14/05/2020 01:48 | IP Address | 100 | 101 |
| 14/05/2020 01:54 | 14/05/2020 01:58 | IP Address | 100 | 212 |
| 14/05/2020 02:59 | 14/05/2020 03:01 | IP Address | 100 | 117 |
| 14/05/2020 03:56 | 14/05/2020 03:59 | IP Address | 100 | 170 |
| 14/05/2020 04:24 | 14/05/2020 04:27 | IP Address | 100 | 156 |
| 14/05/2020 05:50 | 14/05/2020 05:50 | IP Address | 100 | 32 |
| 14/05/2020 05:49 | 14/05/2020 05:51 | IP Address | 100 | 88 |
| 14/05/2020 05:51 | 14/05/2020 05:51 | IP Address | 100 | 26 |
| 14/05/2020 06:49 | 14/05/2020 06:49 | IP Address | 100 | 14 |
| 14/05/2020 06:49 | 14/05/2020 06:49 | IP Address | 100 | 13 |
| 14/05/2020 08:55 | 14/05/2020 08:59 | IP Address | 100 | 203 |
| 14/05/2020 08:59 | 14/05/2020 09:01 | IP Address | 100 | 104 |
| 14/05/2020 09:06 | 14/05/2020 09:07 | IP Address | 100 | 70 |
| 14/05/2020 09:07 | 14/05/2020 09:11 | IP Address | 100 | 232 |
| 14/05/2020 09:11 | 14/05/2020 09:12 | IP Address | 100 | 65 |
| 14/05/2020 09:13 | 14/05/2020 09:14 | IP Address | 100 | 80 |
| 14/05/2020 09:14 | 14/05/2020 09:17 | IP Address | 100 | 147 |
| 14/05/2020 09:17 | 14/05/2020 09:20 | IP Address | 100 | 152 |
| 14/05/2020 09:20 | 14/05/2020 09:21 | IP Address | 100 | 46 |
| 14/05/2020 09:21 | 14/05/2020 09:22 | IP Address | 100 | 74 |
| 14/05/2020 13:49 | 14/05/2020 13:53 | IP Address | 100 | 214 |
| 14/05/2020 13:53 | 14/05/2020 13:54 | IP Address | 100 | 58 |
| 14/05/2020 17:40 | 14/05/2020 17:40 | IP Address | 100 | 16 |
| 14/05/2020 17:40 | 14/05/2020 17:41 | IP Address | 100 | 14 |
| 14/05/2020 17:41 | 14/05/2020 17:43 | IP Address | 100 | 136 |
| 14/05/2020 17:44 | 14/05/2020 17:44 | IP Address | 100 | 11 |
| 14/05/2020 17:44 | 14/05/2020 17:45 | IP Address | 100 | 69 |
| 14/05/2020 17:45 | 14/05/2020 17:45 | IP Address | 100 | 12 |
| 14/05/2020 17:45 | 14/05/2020 17:46 | IP Address | 100 | 12 |
| 14/05/2020 17:46 | 14/05/2020 17:46 | IP Address | 100 | 11 |
| 15/05/2020 03:45 | 15/05/2020 03:49 | IP Address | 100 | 249 |
| 15/05/2020 03:45 | 15/05/2020 03:49 | IP Address | 100 | 254 |

| | | | | |
|---|---|---|---|---|
| 15/05/2020 06:29 | 15/05/2020 06:30 | IP Address | 100 | 65 |
| 15/05/2020 10:51 | 15/05/2020 10:53 | IP Address | 100 | 115 |
| 15/05/2020 15:23 | 15/05/2020 15:26 | IP Address | 100 | 168 |
| 15/05/2020 19:40 | 15/05/2020 19:41 | IP Address | 100 | 59 |
| 15/05/2020 19:42 | 15/05/2020 19:43 | IP Address | 100 | 62 |
| 16/05/2020 01:45 | 16/05/2020 01:49 | IP Address | 100 | 234 |
| 16/05/2020 15:42 | 16/05/2020 15:43 | IP Address | 100 | 50 |
| 16/05/2020 21:08 | 16/05/2020 21:10 | IP Address | 100 | 83 |
| 16/05/2020 21:56 | 16/05/2020 21:56 | IP Address | 100 | 14 |
| 16/05/2020 21:56 | 16/05/2020 21:58 | IP Address | 100 | 87 |
| 17/05/2020 01:45 | 17/05/2020 01:47 | IP Address | 100 | 90 |
| 17/05/2020 03:01 | 17/05/2020 03:04 | IP Address | 100 | 188 |
| 17/05/2020 15:38 | 17/05/2020 15:40 | IP Address | 100 | 125 |
| 17/05/2020 21:32 | 17/05/2020 21:33 | IP Address | 100 | 50 |
| 17/05/2020 21:33 | 17/05/2020 21:33 | IP Address | 100 | 9 |
| 17/05/2020 21:33 | 17/05/2020 21:33 | IP Address | 100 | 11 |
| 17/05/2020 21:33 | 17/05/2020 21:34 | IP Address | 100 | 17 |
| 17/05/2020 21:34 | 17/05/2020 21:35 | IP Address | 100 | 78 |
| 18/05/2020 04:40 | 18/05/2020 04:41 | IP Address | 100 | 66 |
| 18/05/2020 08:33 | 18/05/2020 08:34 | IP Address | 100 | 51 |
| 18/05/2020 11:17 | 18/05/2020 11:17 | IP Address | 100 | 20 |
| 18/05/2020 11:17 | 18/05/2020 11:17 | IP Address | 100 | 13 |
| 18/05/2020 11:18 | 18/05/2020 11:19 | IP Address | 100 | 88 |
| 18/05/2020 19:43 | 18/05/2020 19:43 | Spam | 100 | 4 |
| 19/05/2020 06:10 | 19/05/2020 06:12 | IP Address | 100 | 90 |
| 19/05/2020 06:39 | 19/05/2020 06:40 | IP Address | 100 | 79 |
| 19/05/2020 06:40 | 19/05/2020 06:41 | IP Address | 100 | 34 |
| 19/05/2020 06:41 | 19/05/2020 06:41 | IP Address | 100 | 9 |
| 19/05/2020 06:41 | 19/05/2020 06:41 | IP Address | 100 | 10 |
| 19/05/2020 06:41 | 19/05/2020 06:41 | IP Address | 100 | 9 |
| 19/05/2020 06:41 | 19/05/2020 06:42 | IP Address | 100 | 12 |
| 19/05/2020 06:42 | 19/05/2020 06:42 | IP Address | 100 | 9 |
| 19/05/2020 06:42 | 19/05/2020 06:43 | IP Address | 100 | 11 |
| 19/05/2020 06:46 | 19/05/2020 06:46 | IP Address | 100 | 8 |
| 19/05/2020 06:46 | 19/05/2020 06:47 | IP Address | 100 | 79 |
| 19/05/2020 06:47 | 19/05/2020 06:47 | IP Address | 100 | 8 |
| 19/05/2020 06:47 | 19/05/2020 06:48 | IP Address | 100 | 8 |
| 19/05/2020 06:48 | 19/05/2020 06:48 | IP Address | 100 | 9 |
| 19/05/2020 06:48 | 19/05/2020 06:49 | IP Address | 100 | 57 |
| 19/05/2020 06:49 | 19/05/2020 06:50 | IP Address | 100 | 61 |
| 19/05/2020 06:50 | 19/05/2020 06:50 | IP Address | 100 | 8 |
| 19/05/2020 06:50 | 19/05/2020 06:50 | IP Address | 100 | 8 |
| 19/05/2020 06:51 | 19/05/2020 06:51 | IP Address | 100 | 7 |
| 19/05/2020 06:51 | 19/05/2020 06:51 | IP Address | 100 | 8 |
| 19/05/2020 06:51 | 19/05/2020 06:51 | IP Address | 100 | 8 |
| 19/05/2020 06:51 | 19/05/2020 06:51 | IP Address | 100 | 8 |
| 19/05/2020 06:52 | 19/05/2020 06:52 | IP Address | 100 | 13 |
| 19/05/2020 06:52 | 19/05/2020 06:52 | IP Address | 100 | 7 |

| | | | | |
|---|---|---|---|---|
| 19/05/2020 06:52 | 19/05/2020 06:52 | IP Address | 100 | 9 |
| 19/05/2020 06:53 | 19/05/2020 06:53 | IP Address | 100 | 10 |
| 19/05/2020 06:53 | 19/05/2020 06:53 | IP Address | 100 | 10 |
| 19/05/2020 06:53 | 19/05/2020 06:53 | IP Address | 100 | 8 |
| 19/05/2020 06:53 | 19/05/2020 06:54 | IP Address | 100 | 8 |
| 19/05/2020 06:54 | 19/05/2020 06:54 | IP Address | 100 | 7 |
| 19/05/2020 06:54 | 19/05/2020 06:54 | IP Address | 100 | 9 |
| 19/05/2020 06:54 | 19/05/2020 06:54 | IP Address | 100 | 10 |
| 19/05/2020 06:54 | 19/05/2020 06:54 | IP Address | 100 | 8 |
| 19/05/2020 06:54 | 19/05/2020 06:55 | IP Address | 100 | 9 |
| 21/05/2020 08:49 | 21/05/2020 08:50 | IP Address | 100 | 59 |
| 21/05/2020 19:13 | 21/05/2020 19:13 | IP Address | 100 | 40 |
| 23/05/2020 05:43 | 23/05/2020 05:43 | IP Address | 100 | 30 |
| 27/05/2020 04:27 | 27/05/2020 04:32 | IP Address | 100 | 302 |
| 01/06/2020 16:10 | 01/06/2020 16:11 | IP Address | 100 | 52 |
| 01/06/2020 20:41 | 01/06/2020 20:42 | IP Address | 100 | 58 |
| 03/06/2020 15:59 | 03/06/2020 16:00 | IP Address | 100 | 62 |
| 09/06/2020 16:45 | 09/06/2020 16:46 | IP Address | 100 | 81 |
| 10/06/2020 17:08 | 10/06/2020 17:13 | IP Address | 100 | 276 |
| 10/06/2020 17:13 | 10/06/2020 17:15 | IP Address | 100 | 99 |
| 25/06/2020 00:53 | 25/06/2020 00:55 | IP Address | 100 | 102 |
| 15/09/2020 06:08 | 15/09/2020 06:10 | IP Address | 100 | 122 |
| 15/09/2020 06:11 | 15/09/2020 06:15 | IP Address | 100 | 251 |
| 15/09/2020 06:16 | 15/09/2020 06:17 | IP Address | 100 | 100 |
| 15/09/2020 06:18 | 15/09/2020 06:21 | IP Address | 100 | 195 |
| 16/09/2020 15:17 | 16/09/2020 15:19 | IP Address | 100 | 111 |
| 23/09/2020 08:07 | 23/09/2020 08:08 | IP Address | 100 | 76 |
| 23/09/2020 08:08 | 23/09/2020 08:09 | IP Address | 100 | 95 |
| 23/09/2020 08:09 | 23/09/2020 08:15 | IP Address | 100 | 362 |
| 23/09/2020 08:12 | 23/09/2020 08:15 | IP Address | 100 | 211 |
| 23/09/2020 08:17 | 23/09/2020 08:19 | IP Address | 100 | 118 |
| 23/09/2020 14:10 | 23/09/2020 14:18 | IP Address | 100 | 513 |
| 25/09/2020 04:17 | 25/09/2020 04:19 | IP Address | 100 | 84 |
| 25/09/2020 04:19 | 25/09/2020 04:22 | IP Address | 100 | 180 |
| 28/09/2020 04:34 | 28/09/2020 04:39 | IP Address | 100 | 329 |
| 29/09/2020 17:32 | 29/09/2020 17:37 | IP Address | 100 | 274 |
| 30/09/2020 05:02 | 30/09/2020 05:04 | IP Address | 100 | 139 |
| 30/09/2020 05:04 | 30/09/2020 05:06 | IP Address | 100 | 84 |
| 30/09/2020 05:07 | 30/09/2020 05:09 | IP Address | 100 | 113 |
| 30/09/2020 05:11 | 30/09/2020 05:14 | IP Address | 100 | 181 |
| 30/09/2020 05:18 | 30/09/2020 05:22 | IP Address | 100 | 260 |
| 30/09/2020 05:37 | 30/09/2020 05:39 | IP Address | 100 | 144 |
| 30/09/2020 05:40 | 30/09/2020 05:45 | IP Address | 100 | 251 |
| 04/10/2020 13:48 | 04/10/2020 13:51 | IP Address | 100 | 198 |
| 04/10/2020 13:52 | 04/10/2020 13:57 | IP Address | 100 | 264 |
| 09/10/2020 06:39 | 09/10/2020 13:09 | Survey Preview | 100 | 23360 |
| 20/10/2020 02:38 | 20/10/2020 02:41 | Survey Preview | 100 | 155 |
| 05/11/2020 04:29 | 05/11/2020 04:34 | IP Address | 100 | 301 |

| | | | | |
|---|---|---|---|---|
| 05/11/2020 04:35 | 05/11/2020 04:40 | IP Address | 100 | 276 |
| 05/11/2020 04:41 | 05/11/2020 04:46 | IP Address | 100 | 264 |

| Finished | RecordedDate | ResponseId | DistributionChannel | UserLanguage |
|---|---|---|---|---|
| Finished | Recorded Date | Response ID | Distribution Channel | User Language |
| True | ################# | R_ZrfE5I7mlGOb2Yp | anonymous | EN-GB |
| True | ################# | R_5Bi8aJc5Mg0BCqR | anonymous | EN-GB |
| True | ################# | R_2bTrqTHeY4YwDfE | anonymous | EN-GB |
| True | ################# | R_bNGqfWnYH0ZfuEN | anonymous | EN-GB |
| True | ################# | R_3njBHfqzdlAUBPg | anonymous | EN-GB |
| True | ################# | R_O7n1fnbPHaI5kDn | anonymous | EN-GB |
| True | ################# | R_2zYXtkrdDdCr5I5 | anonymous | EN-GB |
| True | ################# | R_3QENe4Qj3yeCy8A | anonymous | EN-GB |
| True | ################# | R_2B5vdIOcJVOgFof | anonymous | EN-GB |
| True | ################# | R_UsHdPW5UNuBlNXX | anonymous | EN-GB |
| True | ################# | R_2tbrRMbHdTj8vPy | anonymous | EN-GB |
| True | ################# | R_3fJGn2GQS9AbisJ | anonymous | EN-GB |
| True | ################# | R_1lsOjRlZysHeboJ | anonymous | EN-GB |
| True | ################# | R_2WY9YIlrLwzP0B2 | anonymous | EN-GB |
| True | ################# | R_qWQKDpVaSCN5YbL | anonymous | EN-GB |
| True | ################# | R_0wENgcYaIXML64V | anonymous | EN-GB |
| True | ################# | R_O8Q4d87g9swUgpz | anonymous | EN-GB |
| True | ################# | R_XA5ZU1hU5hbfYxH | anonymous | EN-GB |
| True | ################# | R_3rNHFOZz6H2dfTA | anonymous | EN-GB |
| True | ################# | R_2D1t2OyAWuEQh6c | anonymous | EN-GB |
| True | ################# | R_3Rl3TWZu0Q8ooPE | anonymous | EN-GB |
| True | ################# | R_24qjrq8yeB3Zafo | anonymous | EN-GB |
| True | ################# | R_3nfusX4hO2yMZjI | anonymous | EN-GB |
| True | ################# | R_AsXtYuaUEC8kOY1 | anonymous | EN-GB |
| True | ################# | R_279GCwJoR3vc5qW | anonymous | EN-GB |
| True | ################# | R_elf1VE5WPIC70VX | anonymous | EN-GB |
| True | ################# | R_2Ybp3doJ06e303H | anonymous | EN-GB |
| True | ################# | R_1C78vKofTyAXGuW | anonymous | EN-GB |
| True | ################# | R_2eXxMEAoNfaupWL | anonymous | EN-GB |
| True | ################# | R_sOpaXeDeS25xw1H | anonymous | EN-GB |
| True | ################# | R_2WJs2HYSetjYNnV | anonymous | EN-GB |
| True | ################# | R_2ClAoZq4v7UHht2 | anonymous | EN-GB |
| True | ################# | R_1k1XaRQ4bWWEGtg | anonymous | EN-GB |
| True | ################# | R_qKFAkBdRpqDN7wJ | anonymous | EN-GB |
| True | ################# | R_31Z5ck9KYUduHy8 | anonymous | EN-GB |
| True | ################# | R_1gCKnQqX4GgPHBi | anonymous | EN-GB |
| True | ################# | R_1yMyXJUaCczFkdj | anonymous | EN-GB |
| True | ################# | R_1OOYuBrZcN259rZ | anonymous | EN-GB |
| True | ################# | R_2EzY6B7bSQUtHC1 | anonymous | EN-GB |
| True | ################# | R_1r2oMLxrWhSVOBu | anonymous | EN-GB |
| True | ################# | R_21tBWqQSKfowaib | anonymous | EN-GB |
| True | ################# | R_2Enl06LOp3ULejm | anonymous | EN-GB |
| True | ################# | R_ahfgtNHsXzHfgeR | anonymous | EN-GB |
| True | ################# | R_22YwHRHgESCEgJw | anonymous | EN-GB |
| True | ################# | R_1Fs1jKb8PnaDk0n | anonymous | EN-GB |
| True | ################# | R_XMl6WkoCOKnBhpn | anonymous | EN-GB |
| True | ################# | R_RrHg7JyzTiNKhvb | anonymous | EN-GB |

| | | | | |
|---|---|---|---|---|
| True | ################ | R_plVyALnsZLMeMbn | anonymous | EN-GB |
| True | ################ | R_2fPE93KGuTddfOh | anonymous | EN-GB |
| True | ################ | R_Ui1t8diszT4TdEB | anonymous | EN-GB |
| True | ################ | R_2ZTQvcq7uHwddLP | anonymous | EN-GB |
| True | ################ | R_1pA01B96MxWKreG | anonymous | EN-GB |
| True | ################ | R_31nLSFjBztbeUX0 | anonymous | EN-GB |
| True | ################ | R_10Vkz9ROvLEzw9k | anonymous | EN-GB |
| True | ################ | R_2Pp6uKeeJIl3Yyv | anonymous | EN-GB |
| True | ################ | R_9BOtQqcLq92WsV3 | anonymous | EN-GB |
| True | ################ | R_2Bg5S85Q776jXdm | anonymous | EN-GB |
| True | ################ | R_wXdVfi30hwbfIdz | anonymous | EN-GB |
| True | ################ | R_6m0MUgUzq2igGid | anonymous | EN-GB |
| True | ################ | R_AHzUeZrMLJA81ON | anonymous | EN-GB |
| True | ################ | R_PNEhr94kRMf4q89 | anonymous | EN-GB |
| True | ################ | R_3ER50ohfVex9Br3 | anonymous | EN-GB |
| True | ################ | R_1jdARPR0iWNq1Wv | anonymous | EN-GB |
| True | ################ | R_2n0NWohbLILuWfD | anonymous | EN-GB |
| True | ################ | R_1ddibALeWyg0V9Y | anonymous | EN-GB |
| True | ################ | R_1Qyp19rnbgT49c2 | qr | EN-GB |
| True | ################ | R_2w4yv4MRSOTCXrs | anonymous | EN-GB |
| True | ################ | R_umqemMNMWWxsPpD | anonymous | EN-GB |
| True | ################ | R_QoxmH2d1DtCsVmp | anonymous | EN-GB |
| True | ################ | R_e9FqUrGgfQjs67n | anonymous | EN-GB |
| True | ################ | R_77l5bYcTX9ho5wJ | anonymous | EN-GB |
| True | ################ | R_3LcmT4iSyzBlehW | anonymous | EN-GB |
| True | ################ | R_2PA20PSiaXx7z3y | anonymous | EN-GB |
| True | ################ | R_217lrgG358etZ8U | anonymous | EN-GB |
| True | ################ | R_3EZjWxtkzakNfsA | anonymous | EN-GB |
| True | ################ | R_UMZtSkRIf4NPQwF | anonymous | EN-GB |
| True | ################ | R_2bHU6wCCipPHn7L | anonymous | EN-GB |
| True | ################ | R_0x5eBdjeEOAxQYx | social | EN-GB |
| True | ################ | R_1IiPrbxOmqi0WAe | anonymous | EN-GB |
| True | ################ | R_2sbUxEQJ2b3Hx89 | social | EN-GB |
| True | ################ | R_2RVYyW0HEexOxpa | anonymous | EN-GB |
| True | ################ | R_2aFtAQQ03QKWBLz | anonymous | EN-GB |
| True | ################ | R_Q9Zm5eyAd8fyQPD | social | EN-GB |
| True | ################ | R_cMyZJzONSMkDY1X | anonymous | EN-GB |
| True | ################ | R_3jUnSOwjapdk7Tc | anonymous | EN-GB |
| True | ################ | R_2BrhBF1l3fpTnKR | anonymous | EN-GB |
| True | ################ | R_2tEwmkPITaJDnoZ | anonymous | EN-GB |
| True | ################ | R_2RQBkDQVTCpJll4 | anonymous | EN-GB |
| True | ################ | R_2aayP6Sg7UxW7Hk | anonymous | EN-GB |
| True | ################ | R_ZgvFf4Ev3p6Q5mF | anonymous | EN-GB |
| True | ################ | R_1OOEJkuM8E1dHBv | anonymous | EN-GB |
| True | ################ | R_1EaQtUIuPT08tOE | anonymous | EN-GB |
| True | ################ | R_UzFczNqk4UYAsGR | anonymous | EN-GB |
| True | ################ | R_OrRqvXXBdsqf2sV | anonymous | EN-GB |
| True | ################ | R_2y4xgmBGbjPZeJL | anonymous | EN-GB |

| | | | | |
|---|---|---|---|---|
| True | ################ | R_12AzI8tGs4QgVHk | anonymous | EN-GB |
| True | ################ | R_3dQTX7CUYZKyURE | anonymous | EN-GB |
| True | ################ | R_12h8i8XISe5e0k9 | anonymous | EN-GB |
| True | ################ | R_1rNZdRLIfv0ptIb | anonymous | EN-GB |
| True | ################ | R_2ayOnIG1cqNft4c | anonymous | EN-GB |
| True | ################ | R_2TLvDjYJgtph3FY | anonymous | EN-GB |
| True | ################ | R_1GOKNWiCt62tNXe | anonymous | EN-GB |
| True | ################ | R_2EibhciR0laAJ6H | anonymous | EN-GB |
| True | ################ | R_1ClqidqZyjAxB30 | anonymous | EN-GB |
| True | ################ | R_Y4gsafjfikJuyv7 | anonymous | EN-GB |
| True | ################ | R_1OpKCtH7krRzhOs | anonymous | EN-GB |
| True | ################ | R_2q7YloFVimiepI1 | anonymous | EN-GB |
| True | ################ | R_2qxD8yFCY4kS31D | anonymous | EN-GB |
| True | ################ | R_2whLBt0UnrZWPtu | anonymous | EN-GB |
| True | ################ | R_TnijldDbw246cwN | anonymous | EN-GB |
| True | ################ | R_3P6S5hBEaaC0Ull | anonymous | EN-GB |
| True | ################ | R_25SnPMuT0O6yGhQ | social | EN-GB |
| True | ################ | R_124OUZsgmzzC5gY | anonymous | EN-GB |
| True | ################ | R_12P8glrXF5oSEAx | anonymous | EN-GB |
| True | ################ | R_3g6OECW4es04xW1 | anonymous | EN-GB |
| True | ################ | R_4OevD0kfFSwFNF7 | anonymous | EN-GB |
| True | ################ | R_OjwUSlUVzDV7W3n | anonymous | EN-GB |
| True | ################ | R_3MiIkgdB68WTB9p | anonymous | EN-GB |
| True | ################ | R_1Is0OoWqLPJ0pR7 | anonymous | EN-GB |
| True | ################ | R_3oGwlqwaxYBKKcF | anonymous | EN-GB |
| True | ################ | R_1mxQ1Secl9JJ9TN | anonymous | EN-GB |
| True | ################ | R_3oLrUD8lLD0aGrL | anonymous | EN-GB |
| True | ################ | R_2CVYbQXQVpp7q78 | anonymous | EN-GB |
| True | ################ | R_u2PWmtlSzo9uStP | anonymous | EN-GB |
| True | ################ | R_2QWh97aUCMvJEZb | anonymous | EN-GB |
| True | ################ | R_3Ea3Y4AIVigju3x | anonymous | EN-GB |
| True | ################ | R_2Pp0b1PBLY3SfCA | anonymous | EN-GB |
| True | ################ | R_2uDjfeNrOwbYoe6 | anonymous | EN-GB |
| True | ################ | R_1NksDsY5ZmSWjKm | anonymous | EN-GB |
| True | ################ | R_UcjdYUfJmKT90cx | social | EN-GB |
| True | ################ | R_2Y3VKswJLUuHBTu | social | EN-GB |
| True | ################ | R_3GddSJD9BV7F2D6 | anonymous | EN-GB |
| True | ################ | R_3KARCjblPI7Q92V | anonymous | EN-GB |
| True | ################ | R_23918a25CHX0wwP | anonymous | EN-GB |
| True | ################ | R_Zkv3THOyOkTiAJr | anonymous | EN-GB |
| True | ################ | R_bgeeLRDaTEAAIbT | anonymous | EN-GB |
| True | ################ | R_27OPTFD64SFvt9N | anonymous | EN-GB |
| True | ################ | R_do2nDd9z0K7QezL | anonymous | EN-GB |
| True | ################ | R_UmUf597qnA651VD | anonymous | EN-GB |
| True | ################ | R_1N48HghBTuQkS7p | anonymous | EN-GB |
| True | ################ | R_2Pu1XDTkp7vcuYK | anonymous | EN-GB |
| True | ################ | R_3mavYyg0WVDt96a | anonymous | EN-GB |
| True | ################ | R_qxbHRw1QOa85Vgl | anonymous | EN-GB |

| | | | | |
|---|---|---|---|---|
| True | ################ | R_296ejo84v80bbF8 | anonymous | EN-GB |
| True | ################ | R_1n1w1BnblxUUNru | anonymous | EN-GB |
| True | ################ | R_12SIFLxzyzeO050 | anonymous | EN-GB |
| True | ################ | R_2X1lDHJCPuQ7kr2 | anonymous | EN-GB |
| True | ################ | R_26sLyCZhZWk28DL | anonymous | EN-GB |
| True | ################ | R_2s6VDNujzVkxPNc | anonymous | EN-GB |
| True | ################ | R_ahHemjft9dKHAKl | social | EN-GB |
| True | ################ | R_31QQ2dzpOAWX5i3 | social | EN-GB |
| True | ################ | R_27424msS46WNrLW | social | EN-GB |
| True | ################ | R_2dBbNS1oY1v4C5k | social | EN-GB |
| True | ################ | R_bKuUR5iY9Xevj9L | social | EN-GB |
| True | ################ | R_31mSx1yr33iNUkM | social | EN-GB |
| True | ################ | R_ehcsr8MKYF3706B | social | EN-GB |
| True | ################ | R_3G8LpAPKncsdNBv | social | EN-GB |
| True | ################ | R_3KIPTkdHVFToYSt | social | EN-GB |
| True | ################ | R_DpmhFRbEDGS4ohP | social | EN-GB |
| True | ################ | R_Wp6lCwse9hk17Jn | anonymous | EN-GB |
| True | ################ | R_C3oetF4XNk2x1W9 | anonymous | EN-GB |
| True | ################ | R_27xRtrbn0QAdb61 | anonymous | EN-GB |
| True | ################ | R_1feIz6CFQ5uTqKU | anonymous | EN-GB |
| True | ################ | R_1M0Y0OXLe8ouhwe | anonymous | EN-GB |
| True | ################ | R_12PRZEeiWiChtE3 | anonymous | EN-GB |
| True | ################ | R_sNlNUBqLCAeV2fL | anonymous | EN-GB |
| True | ################ | R_1DvRwxPGaZgjOQr | anonymous | EN-GB |
| True | ################ | R_1ojyeXd05TP8kXQ | anonymous | EN-GB |
| True | ################ | R_2XiosvRjdBsVkvj | anonymous | EN-GB |
| True | ################ | R_1r2ryIwqUy6FlXk | anonymous | EN-GB |
| True | ################ | R_2CCJGvGxBABSZV9 | anonymous | EN-GB |
| True | ################ | R_1GIj3jOEFCSTfVd | anonymous | EN-GB |
| True | ################ | R_3O3omAZROdxr9q6 | anonymous | EN-GB |
| True | ################ | R_2ErWhpw8rTdUqZp | anonymous | EN-GB |
| True | ################ | R_2zCOHiM6M3HIAMU | anonymous | EN-GB |
| True | ################ | R_21nPqZKFcEHSpG6 | anonymous | EN-GB |
| True | ################ | R_3QLPTiwsGhdH9qx | anonymous | EN-GB |
| True | ################ | R_2YVUpXNqfGEBOmX | anonymous | EN-GB |
| True | ################ | R_1FqQ7Nk74qBeCAh | anonymous | EN-GB |
| True | ################ | R_Ub7F8w1rRPd82it | anonymous | EN-GB |
| True | ################ | R_VJxy0CTsCZYxdnj | anonymous | EN-GB |
| True | ################ | R_3iVjRwyorjlMpml | anonymous | EN-GB |
| True | ################ | R_aVhNVR2TMWf735T | anonymous | EN-GB |
| True | ################ | R_2Y67SDxoDAVvRAC | anonymous | EN-GB |
| True | ################ | R_UVAL2d0jAH3n1EB | anonymous | EN-GB |
| True | ################ | R_yR9nEF7BCRPFWhz | anonymous | EN-GB |
| True | ################ | R_1M6OX4YtfFjgxTK | anonymous | EN-GB |
| True | ################ | R_QoXDloTG5JVIoSJ | anonymous | EN-GB |
| True | ################ | R_NVaG4Cfdnf3gxVf | anonymous | EN-GB |
| True | ################ | R_BPVqfQG3xSCJpNT | anonymous | EN-GB |
| True | ################ | R_24OEr6DnIjQ1MSZ | anonymous | EN-GB |

| | | | | |
|---|---|---|---|---|
| True | ################## | R_24B27aWpthEBTWt | anonymous | EN-GB |
| True | ################## | R_2yjUu0oMH7O46lo | anonymous | EN-GB |
| True | ################## | R_0MRiReeShbamPwl | anonymous | EN-GB |
| True | ################## | R_ClTsWXTC5TNdOj7 | anonymous | EN-GB |
| True | ################## | R_3PAl1zXFjKaJRIk | anonymous | EN-GB |
| True | ################## | R_2rllGbhTWdGCPwS | anonymous | EN-GB |
| True | ################## | R_2tJ4L55ryqe1OuE | anonymous | EN-GB |
| True | ################## | R_27wAxQoMRiNAdp5 | anonymous | EN-GB |
| True | ################## | R_8wYAfIjlifRuXRL | anonymous | EN-GB |
| True | ################## | R_1NdWkcvburQDMT5 | anonymous | EN-GB |
| True | ################## | R_1gjxs6jXQPrudSr | anonymous | EN-GB |
| True | ################## | R_1Nrd93vebMeQXZK | anonymous | EN-GB |
| True | ################## | R_1rGEfOrZO2xg3yM | anonymous | EN-GB |
| True | ################## | R_31apqSmXVGGOY7O | anonymous | EN-GB |
| True | ################## | R_1IikyfVMHJhAiNX | anonymous | EN-GB |
| True | ################## | R_3DuM2w8eZxQZmPp | anonymous | EN-GB |
| True | ################## | R_1gMwJ7IPVrVJAKg | anonymous | EN-GB |
| True | ################## | R_3gZx5gHnjVEfB2k | anonymous | EN-GB |
| True | ################## | R_1LHgOSC3q76IP0S | anonymous | EN-GB |
| True | ################## | R_1MNjOnVTvfWa11E | anonymous | EN-GB |
| True | ################## | R_1OPMIM486V74Bz1 | anonymous | EN-GB |
| True | ################## | R_2fvvGiuWxTpLfQF | anonymous | EN-GB |
| True | ################## | R_tF2w2IGsmnbbMUF | anonymous | EN-GB |
| True | ################## | R_2XhOB9XSJqfdRhs | anonymous | EN-GB |
| True | ################## | R_1GIzrjDZZO7lkM3 | anonymous | EN-GB |
| True | ################## | R_2zMfUEVo3iavCzG | anonymous | EN-GB |
| True | ################## | R_2E4QxMf8cJMSCLK | anonymous | EN-GB |
| True | ################## | R_vjZ4HPmq2PkIqcx | anonymous | EN-GB |
| True | ################## | R_3O6L7yRp117sYWU | anonymous | EN-GB |
| True | ################## | R_1DwklJr3nzsBCc1 | anonymous | EN-GB |
| True | ################## | R_PNfAc7kdW6at2yR | anonymous | EN-GB |
| True | ################## | R_0Bf8by2p0da5L5D | anonymous | EN-GB |
| True | ################## | R_24jUzxasLMa6BlD | anonymous | EN-GB |
| True | ################## | R_1jCKhztJfnnPQnl | anonymous | EN-GB |
| True | ################## | R_2WwHPexw5YhAs16 | anonymous | EN-GB |
| True | ################## | R_2Qi9wbcMiixHDnK | anonymous | EN-GB |
| True | ################## | R_3DjbpDH4hinW5OF | anonymous | EN-GB |
| True | ################## | R_2TT12e9YblRfOb3 | anonymous | EN-GB |
| True | ################## | R_eJLR1673IAdrk4N | anonymous | EN-GB |
| True | ################## | R_1mVlYMg12HoPojJ | anonymous | EN-GB |
| True | ################## | R_2atY3zcgnwJjb8D | anonymous | EN-GB |
| True | ################## | R_240lMmTHQ4lkCTB | anonymous | EN-GB |
| True | ################## | R_cNQzjrXQUkCz0Up | anonymous | EN-GB |
| True | ################## | R_PGQFpRHVbXx5Al3 | anonymous | EN-GB |
| True | ################## | R_2bTcAMG4OZfHLfH | anonymous | EN-GB |
| True | ################## | R_eGackFD9N92Smxb | anonymous | EN-GB |
| True | ################## | R_2CmnvaVacvUVMt8 | anonymous | EN-GB |
| True | ################## | R_2yfwkutmhx16sX5 | anonymous | EN-GB |

| | | | | |
|---|---|---|---|---|
| True | ################# | R_4NP7EC8N8jL4VQ5 | anonymous | EN-GB |
| True | ################# | R_1PZOwslLw8ACLHP | anonymous | EN-GB |
| True | ################# | R_2CWDLN2gXfTL04x | anonymous | EN-GB |
| True | ################# | R_1LSt9KCEJFzYPaE | anonymous | EN-GB |
| True | ################# | R_3NxtdflExVi4KSC | anonymous | EN-GB |
| True | ################# | R_blTkgaYgyYCirhn | anonymous | EN-GB |
| True | ################# | R_3iU0GJbhl7c1IIX | anonymous | EN-GB |
| True | ################# | R_3dEAJNnvwzXX51X | anonymous | EN-GB |
| True | ################# | R_1KpYhwjJ7vgbAQ3 | anonymous | EN-GB |
| True | ################# | R_2fkrumBzaYwLmvU | anonymous | EN-GB |
| True | ################# | R_1FwEmZQ1FncN79f | anonymous | EN-GB |
| True | ################# | R_RwcK1Kc22KoiFWh | anonymous | EN-GB |
| True | ################# | R_1ILUUYw9z6eUmFr | anonymous | EN-GB |
| True | ################# | R_3oHJs7PKJtXWbUN | anonymous | EN-GB |
| True | ################# | R_1jk6QMYci3Qn3S0 | anonymous | EN-GB |
| True | ################# | R_3pg3KOTeOygwFqI | anonymous | EN-GB |
| True | ################# | R_1LzobVd7bjxZGcU | anonymous | EN-GB |
| True | ################# | R_Z1MyfYM5suzdPtn | anonymous | EN-GB |
| True | ################# | R_sZqM8RsPaHTaZfr | anonymous | EN-GB |
| True | ################# | R_2rOerSo1Da0Ly0a | anonymous | EN-GB |
| True | ################# | R_2qawHNcO8owOY1G | anonymous | EN-GB |
| True | ################# | R_0dq43H3aPNYUSZP | anonymous | EN-GB |
| True | ################# | R_3HHsod8isCMDYrk | anonymous | EN-GB |
| True | ################# | R_12FrQKY7FCDHZP7 | anonymous | EN-GB |
| True | ################# | R_1BYqAaytdc9qyuB | anonymous | EN-GB |
| True | ################# | R_28MsKCKAyiO51GK | anonymous | EN-GB |
| True | ################# | R_RIgCybXEB0ZGAJX | anonymous | EN-GB |
| True | ################# | R_3hayzYtP1EM6NZx | anonymous | EN-GB |
| True | ################# | R_1F3ajLXUU6TTU6o | anonymous | EN-GB |
| True | ################# | R_33kYRFVQn2HHF4y | anonymous | EN-GB |
| True | ################# | R_3oYZmnvtXlyZzZh | anonymous | EN-GB |
| True | ################# | R_2feTrW5ZTNphOGD | anonymous | EN-GB |
| True | ################# | R_2ZV5UiNSHpt1Fil | anonymous | EN-GB |
| True | ################# | R_1jBOOD9cDduUYIW | anonymous | EN-GB |
| True | ################# | R_e9UbpP1eYnHlXKV | anonymous | EN-GB |
| True | ################# | R_3PdA0MQ6YGxBWrO | anonymous | EN-GB |
| True | ################# | R_qVqVUOeAyZ6bMMF | anonymous | EN-GB |
| True | ################# | R_w5HCRuzd4KAwRUd | anonymous | EN-GB |
| True | ################# | R_2VjVR5rfE7tD2aA | anonymous | EN-GB |
| True | ################# | R_vIbr4VBP1OCnq7L | anonymous | EN-GB |
| True | ################# | R_2ZQxlaNIlSbv97R | anonymous | EN-GB |
| True | ################# | R_6hdgTClSoNNBuBX | anonymous | EN-GB |
| True | ################# | R_2QWe2FPc6a2rRiN | anonymous | EN-GB |
| True | ################# | R_1Gyw8IXOhS5nNaR | anonymous | EN-GB |
| True | ################# | R_1CscUZwlW5jsCZk | anonymous | EN-GB |
| True | ################# | R_2y2FRfmqMkQ89Gx | anonymous | EN-GB |
| True | ################# | R_3kvaZUPiG8mGhmQ | anonymous | EN-GB |
| True | ################# | R_ugeUWHsOOPDW5DH | anonymous | EN-GB |

| | | | | |
|---|---|---|---|---|
| True | ################## | R_2YaYa6DPi8OhlpN | anonymous | EN-GB |
| True | ################## | R_2tlNLWYzwc4lRZG | anonymous | EN-GB |
| True | ################## | R_3iLvtNgFojTjhjr | anonymous | EN-GB |
| True | ################## | R_2YSvPvtgwMHyeJq | anonymous | EN-GB |
| True | ################## | R_3kkemOG5g45WJEK | anonymous | EN-GB |
| True | ################## | R_1OZu81QjdICpMrW | anonymous | EN-GB |
| True | ################## | R_10Ss9A356EQSUDv | anonymous | EN-GB |
| True | ################## | R_3Eu0yGNjW5eXGNs | anonymous | EN-GB |
| True | ################## | R_1eLiWMSioqbaWIo | anonymous | EN-GB |
| True | ################## | R_3luEZIrAYB9AiLk | anonymous | EN-GB |
| True | ################## | R_vJK69EpTPmkElLX | anonymous | EN-GB |
| True | ################## | R_339kjjnxBQPcoxf | anonymous | EN-GB |
| True | ################## | R_1iaOVfB1gn5mh8G | anonymous | EN-GB |
| True | ################## | R_2w0QuT7PgUOriFc | anonymous | EN-GB |
| True | ################## | R_3ElEG6SeRpmh3Kb | anonymous | EN-GB |
| True | ################## | R_3fOCgZkbH4qXt2K | anonymous | EN-GB |
| True | ################## | R_3R4b02AHQDZmn1r | anonymous | EN-GB |
| True | ################## | R_30uGhmAhEBQr6P5 | anonymous | EN-GB |
| True | ################## | R_2ZV3DPDAjS89W9D | anonymous | EN-GB |
| True | ################## | R_AvOYEsiAy6g13DX | anonymous | EN-GB |
| True | ################## | R_USASibJVVjqMJhv | anonymous | EN-GB |
| True | ################## | R_1la7b4xJqDi7OIK | anonymous | EN-GB |
| True | ################## | R_3emhGS9A6cCe6jl | anonymous | EN-GB |
| True | ################## | R_2y3Ysx1nHm0z2Mz | anonymous | EN-GB |
| True | ################## | R_25XVBkVhIA4kHhA | anonymous | EN-GB |
| True | ################## | R_3GfEaAJKKbX8CYw | anonymous | EN-GB |
| True | ################## | R_Q4a64ZU1uCBUSZ3 | anonymous | EN-GB |
| True | ################## | R_1lrTa5OGgUlZD9G | anonymous | EN-GB |
| True | ################## | R_3O7lQsjZgV8v0Wm | anonymous | EN-GB |
| True | ################## | R_2QYXPa6Ng3tGyar | anonymous | EN-GB |
| True | ################## | R_1CCQwCEpw7n5Ck4 | anonymous | EN-GB |
| True | ################## | R_2doQ0vXNbdZFAUc | anonymous | EN-GB |
| True | ################## | R_2P4EsxDELdegL7Q | anonymous | EN-GB |
| True | ################## | R_uxFwdKGYTBNfD0t | anonymous | EN-GB |
| True | ################## | R_3O1w4SH3RPjOsss | anonymous | EN-GB |
| True | ################## | R_2thyeBBb4STkys9 | anonymous | EN-GB |
| True | ################## | R_2Btimttkh1Jsbqm | anonymous | EN-GB |
| True | ################## | R_11ZSqKGe2rsQVJV | anonymous | EN-GB |
| True | ################## | R_1IEF304G370BXgX | anonymous | EN-GB |
| True | ################## | R_1r2of4vKygmcNvX | anonymous | EN-GB |
| True | ################## | R_25Wuz4mK1qqsje5 | anonymous | EN-GB |
| True | ################## | R_3m3qRiG1v5I2mPF | anonymous | EN-GB |
| True | ################## | R_24xcA41Ea0SdLGl | anonymous | EN-GB |
| True | ################## | R_PGqtVTWBa3Hi4aR | anonymous | EN-GB |
| True | ################## | R_AgKPWtWm4WJy8fL | anonymous | EN-GB |
| True | ################## | R_2upwqAs0OhHUgc5 | anonymous | EN-GB |
| True | ################## | R_24pizLCzntb77e6 | anonymous | EN-GB |
| True | ################## | R_1FgraQflEFDUcJd | anonymous | EN-GB |

| | | | | |
|---|---|---|---|---|
| True | ################## | R_2TY7cDJXrbN9ryJ | anonymous | EN-GB |
| True | ################## | R_2QtXj3q5vhEMjjk | anonymous | EN-GB |
| True | ################## | R_VPc4fhmhkkDaSmB | anonymous | EN-GB |
| True | ################## | R_2SGKI3qEnCg1sD6 | anonymous | EN-GB |
| True | ################## | R_1H7dUCAz3Wt3R41 | anonymous | EN-GB |
| True | ################## | R_WomkM1o5dwTikal | anonymous | EN-GB |
| True | ################## | R_2RVSFrXaLbl3t26 | anonymous | EN-GB |
| True | ################## | R_2D7UI9UdzSqMa3c | anonymous | EN-GB |
| True | ################## | R_214IU1LmgTkJpXy | anonymous | EN-GB |
| True | ################## | R_3fdR4PYm1zODU0U | anonymous | EN-GB |
| True | ################## | R_8GNRzgfTTnJ7ULT | anonymous | EN-GB |
| True | ################## | R_1EgsfL5WkJ1AxLd | anonymous | EN-GB |
| True | ################## | R_2eaMyDZZWHb3DvU | anonymous | EN-GB |
| True | ################## | R_1QFRJkjWtzlqGfK | anonymous | EN-GB |
| True | ################## | R_xyEm6IZswNuknsJ | anonymous | EN-GB |
| True | ################## | R_3gSLx3d8TX7Ga9H | anonymous | EN-GB |
| True | ################## | R_2uZAQ4uRsFlzqMf | anonymous | EN-GB |
| True | ################## | R_3Enmaw8oxnIF0Qd | anonymous | EN-GB |
| True | ################## | R_1rlAnbYK218JUfw | anonymous | EN-GB |
| True | ################## | R_2sceoC9Jox9qsod | anonymous | EN-GB |
| True | ################## | R_3e8mt9xor4YJVCo | anonymous | EN-GB |
| True | ################## | R_2qslQaiy67R68mV | anonymous | EN-GB |
| True | ################## | R_cHfWtr9pYAkmVcl | anonymous | EN-GB |
| True | ################## | R_25MGMvFQt4d0lcR | anonymous | EN-GB |
| True | ################## | R_8fiDzR1iyl35eSZ | anonymous | EN-GB |
| True | ################## | R_1rkJQouES4N0KF9 | anonymous | EN-GB |
| True | ################## | R_30jh8Irvn6U0Os6 | anonymous | EN-GB |
| True | ################## | R_C78XaA7ype7wg13 | anonymous | EN-GB |
| True | ################## | R_Zq1t5qzuTdPKmxX | anonymous | EN-GB |
| True | ################## | R_3fcmXcHQ342BFtU | anonymous | EN-GB |
| True | ################## | R_3GoD2hM5h4GpSt3 | anonymous | EN-GB |
| True | ################## | R_3EW2CC3TpfmuqMS | anonymous | EN-GB |
| True | ################## | R_yrOUL3MsfB5zOyl | anonymous | EN-GB |
| True | ################## | R_2S2jkBwv65DXCB8 | anonymous | EN-GB |
| True | ################## | R_2S1ZJtsv1bmTWQe | anonymous | EN-GB |
| True | ################## | R_1IzSmbNeztfCDFQ | anonymous | EN-GB |
| True | ################## | R_3Ed2FW6c0mvzqau | anonymous | EN-GB |
| True | ################## | R_w1tQbfPKSQBsd3j | anonymous | EN-GB |
| True | ################## | R_3eabbsJqFCrgwtC | anonymous | EN-GB |
| True | ################## | R_2TXmM8Wgt5GtqF9 | anonymous | EN-GB |
| True | ################## | R_2yr8KXI5M3nk42P | anonymous | EN-GB |
| True | ################## | R_1mq1t2ZBobMZGbB | anonymous | EN-GB |
| True | ################## | R_037LH7Aq6gc8cCd | anonymous | EN-GB |
| True | ################## | R_1PdZ9fgbhyd1c8S | anonymous | EN-GB |
| True | ################## | R_UJXkpg7WqJB7qFz | anonymous | EN-GB |
| True | ################## | R_3FXJoDAEH3qVEt2 | anonymous | EN-GB |
| True | ################## | R_3PdehoTFZiozoFd | anonymous | EN-GB |
| True | ################## | R_Cf3CTCvTxdpLKPD | anonymous | EN-GB |

| | | | | |
|---|---|---|---|---|
| True | ################ | R_1nMDqEHvwEBC3Nk | anonymous | EN-GB |
| True | ################ | R_3EAU3cNJ03qNtXb | anonymous | EN-GB |
| True | ################ | R_bsgrFa6fsUXdfyN | anonymous | EN-GB |
| True | ################ | R_2E5GSbzbMg4ikrg | anonymous | EN-GB |
| True | ################ | R_20tq3AN0yQWBQSl | anonymous | EN-GB |
| True | ################ | R_1FKyIHegoOcpCAP | anonymous | EN-GB |
| True | ################ | R_T7NxTZbV4CjbGlr | anonymous | EN-GB |
| True | ################ | R_2dQIPTscaNT0G3h | anonymous | EN-GB |
| True | ################ | R_ulb4rDOYssgAzex | anonymous | EN-GB |
| True | ################ | R_2xRwZvzIhCm37DL | anonymous | EN-GB |
| True | ################ | R_2c1QdNxTZrcCNwq | anonymous | EN-GB |
| True | ################ | R_T7KOyp31u512DuN | anonymous | EN-GB |
| True | ################ | R_UrljN4gU9hQnvSV | anonymous | EN-GB |
| True | ################ | R_bq1Nv5Ob9Wx2LtL | anonymous | EN-GB |
| True | ################ | R_UnkI4r3BioeblDz | anonymous | EN-GB |
| True | ################ | R_2Et6TvK3pmmhlYR | anonymous | EN-GB |
| True | ################ | R_2CZJwfpbqfLH5q2 | anonymous | EN-GB |
| True | ################ | R_2Bxs5F8qXEdJaOp | anonymous | EN-GB |
| True | ################ | R_25tmgoEHq3hz60o | anonymous | EN-GB |
| True | ################ | R_82E07j584gBLvl7 | anonymous | EN-GB |
| True | ################ | R_3hmT3DqxkzhsBXK | anonymous | EN-GB |
| True | ################ | R_1FybyaKoNmzHSz5 | anonymous | EN-GB |
| True | ################ | R_2bKk4kZFNAEW5I3 | anonymous | EN-GB |
| True | ################ | R_1LioqWXVFhOlH2Z | anonymous | EN-GB |
| True | ################ | R_URVZ3RY4SgyasYF | anonymous | EN-GB |
| True | ################ | R_SHl0YUILPWyfydr | anonymous | EN-GB |
| True | ################ | R_tM7hM8Vde13mG1H | anonymous | EN-GB |
| True | ################ | R_1rOEFa1Vz9eNAJI | anonymous | EN-GB |
| True | ################ | R_3CQlGKyavPcDeFR | anonymous | EN-GB |
| True | ################ | R_yDxW1NvwQB1XhYd | anonymous | EN-GB |
| True | ################ | R_2a8ngEVIJEYn07E | anonymous | EN-GB |
| True | ################ | R_3NX9B8d56DYOTHg | anonymous | EN-GB |
| True | ################ | R_3EySwD8ETcLtWMF | anonymous | EN-GB |
| True | ################ | R_1QMEO80QKPY8qM7 | anonymous | EN-GB |
| True | ################ | R_ZHV9qA143h4jUu5 | anonymous | EN-GB |
| True | ################ | R_2Bi0lc6I8BvRc7k | anonymous | EN-GB |
| True | ################ | R_2QPNBilG6a9UafU | anonymous | EN-GB |
| True | ################ | R_1igi9bcuYpJj5OJ | anonymous | EN-GB |
| True | ################ | R_22zya0shS1sMk98 | anonymous | EN-GB |
| True | ################ | R_22Dnnd1sfZhtrwy | anonymous | EN-GB |
| True | ################ | R_3nMWElrdYPjpsNa | anonymous | EN-GB |
| True | ################ | R_R2FBWQAns6ihGCZ | anonymous | EN-GB |
| True | ################ | R_2DN7zcnRr2TbjkV | anonymous | EN-GB |
| True | ################ | R_3G0VcnaE3wNjxf6 | anonymous | EN-GB |
| True | ################ | R_BWAy45cZuKrHxOV | anonymous | EN-GB |
| True | ################ | R_3rTxQzlhnjzCUwm | anonymous | EN-GB |
| True | ################ | R_32XUWvF03EzCRBR | anonymous | EN-GB |
| True | ################ | R_PvnoBEkJ37458Vb | anonymous | EN-GB |

| | | | | |
|---|---|---|---|---|
| True | ################ | R_3EQilCUKZVfCTmH | anonymous | EN-GB |
| True | ################ | R_DvfiMGuGuN42jiV | anonymous | EN-GB |
| True | ################ | R_2c5HbtXMTfppvMR | anonymous | EN-GB |
| True | ################ | R_3I0qPLKJy4bbuUx | anonymous | EN-GB |
| True | ################ | R_1BY1xgYvtsco3VF | anonymous | EN-GB |
| True | ################ | R_33vyL1VxNmtI9l5 | anonymous | EN-GB |
| True | ################ | R_1JLPZm7VRt6Ecj0 | anonymous | EN-GB |
| True | ################ | R_UDBt3j08usvmV5n | anonymous | EN-GB |
| True | ################ | R_2g9wyfquA7Ae1XP | anonymous | EN-GB |
| True | ################ | R_3JD7KjMR4wxyzYQ | anonymous | EN-GB |
| True | ################ | R_3nDy7iacQnp4Bnq | anonymous | EN-GB |
| True | ################ | R_1fZRCc1LePffruF | anonymous | EN-GB |
| True | ################ | R_tWlqRIqALRTxkLT | anonymous | EN-GB |
| True | ################ | R_11aqMDuPj8Fmk79 | anonymous | EN-GB |
| True | ################ | R_9yuu5leXTdLtPB7 | anonymous | EN-GB |
| True | ################ | R_1LSXUVZ8lVXaVNX | anonymous | EN-GB |
| True | ################ | R_3lFwcXunpuYyZeX | anonymous | EN-GB |
| True | ################ | R_10UzCMU4irZuJ0R | anonymous | EN-GB |
| True | ################ | R_3Pot9yv8OhuZ2X5 | anonymous | EN-GB |
| True | ################ | R_22Q8Xvk6rUPEuP3 | anonymous | EN-GB |
| True | ################ | R_1CgwhhkRduRUrtg | anonymous | EN-GB |
| True | ################ | R_aa8nYUwtDDXLpD3 | anonymous | EN-GB |
| True | ################ | R_1ITwbW6GYMuCZ2Q | anonymous | EN-GB |
| True | ################ | R_1k20ff14KqZu3xp | anonymous | EN-GB |
| True | ################ | R_XQZP3lrY0cDe2J3 | anonymous | EN-GB |
| True | ################ | R_ujIlzGNeFPMugOB | anonymous | EN-GB |
| True | ################ | R_1gjUO6tWfzZF6g0 | anonymous | EN-GB |
| True | ################ | R_3EPYopiosi8U5TA | anonymous | EN-GB |
| True | ################ | R_0TBEkElEHFEeItb | anonymous | EN-GB |
| True | ################ | R_1JIyZp18IXfqT82 | anonymous | EN-GB |
| True | ################ | R_30xjAkgfbX9rGYN | anonymous | EN-GB |
| True | ################ | R_xr3MC6YoKsEgm8F | anonymous | EN-GB |
| True | ################ | R_2yfPckepOzCg5yg | anonymous | EN-GB |
| True | ################ | R_1IlOXarZv5rg5sQ | anonymous | EN-GB |
| True | ################ | R_AMTcHOIaNz24bXr | anonymous | EN-GB |
| True | ################ | R_1P4WCyHMO56uC49 | anonymous | EN-GB |
| True | ################ | R_7TX0lJw3ysWX5tf | anonymous | EN-GB |
| True | ################ | R_2VDarXIEFYi3pMJ | anonymous | EN-GB |
| True | ################ | R_3KZ6OB4N6tqC1q2 | anonymous | EN-GB |
| True | ################ | R_24OQIbU1nTCn5QI | anonymous | EN-GB |
| True | ################ | R_VXuAXRjgnL07feF | anonymous | EN-GB |
| True | ################ | R_3fI5t8hkWY2ArVi | anonymous | EN-GB |
| True | ################ | R_3q7hBcp1SJmkbJ1 | anonymous | EN-GB |
| True | ################ | R_3LhEHXMLjy9jYVG | anonymous | EN-GB |
| True | ################ | R_1gnnuK1PKHv6u8N | anonymous | EN-GB |
| True | ################ | R_2pSOz7yRPXq8I15 | anonymous | EN-GB |
| True | ################ | R_1DGaPw13jCQ6g28 | anonymous | EN-GB |
| True | ################ | R_ywto87eqdgvs3gR | anonymous | EN-GB |

| | | | | |
|---|---|---|---|---|
| True | ################## | R_3J97Ufl1Dnh23ZX | anonymous | EN-GB |
| True | ################## | R_sbSrmnMJlwPvz3P | anonymous | EN-GB |
| True | ################## | R_1PdDmbROHJOYr0c | anonymous | EN-GB |
| True | ################## | R_3mgb0Td6qxSbjdD | anonymous | EN-GB |
| True | ################## | R_2PorHSRnO5f3Tem | anonymous | EN-GB |
| True | ################## | R_BWZyIuOMK6SPsad | anonymous | EN-GB |
| True | ################## | R_s7t46S6dGu18F33 | anonymous | EN-GB |
| True | ################## | R_1rrxzlJJ0ndcYV2 | anonymous | EN-GB |
| True | ################## | R_0pwKR6JvYxs50TD | anonymous | EN-GB |
| True | ################## | R_ZwUNch2acVepZ29 | anonymous | EN-GB |
| True | ################## | R_2aeRYC6RzLp0sGP | anonymous | EN-GB |
| True | ################## | R_3PZ8AoaG7KR7Jtn | anonymous | EN-GB |
| True | ################## | R_xfJAOOWY9ln7cPf | anonymous | EN-GB |
| True | ################## | R_1r3yjZY3L0wapMK | anonymous | EN-GB |
| True | ################## | R_3nPKDEkFt8gilQM | anonymous | EN-GB |
| True | ################## | R_3CNnoYRhQHRQ8e0 | anonymous | EN-GB |
| True | ################## | R_1cRC7TCAtYEl91w | anonymous | EN-GB |
| True | ################## | R_ZmEoEhrkT80vbmF | anonymous | EN-GB |
| True | ################## | R_11X8xSiOIdD03tf | anonymous | EN-GB |
| True | ################## | R_3OkwQIDzlyJDjLP | anonymous | EN-GB |
| True | ################## | R_3IYqO5j1155DIg8 | anonymous | EN-GB |
| True | ################## | R_3lSI3kuncCjC600 | anonymous | EN-GB |
| True | ################## | R_1K0HXnI1CPtPE0K | anonymous | EN-GB |
| True | ################## | R_246ICp4STsXaW4C | anonymous | EN-GB |
| True | ################## | R_1jJ43D45ftUZBqH | anonymous | EN-GB |
| True | ################## | R_1IT2HBhBLKqv9AL | anonymous | EN-GB |
| True | ################## | R_12hY4ues8E8OASY | anonymous | EN-GB |
| True | ################## | R_2aK7GeH5MabkgKk | anonymous | EN-GB |
| True | ################## | R_22nvJzg2KuD3MbJ | anonymous | EN-GB |
| True | ################## | R_2wdd2CmCZ4SZE7c | anonymous | EN-GB |
| True | ################## | R_yO4xFzh9BHZqYh3 | anonymous | EN-GB |
| True | ################## | R_tQy9LMjsctwEh0d | anonymous | EN-GB |
| True | ################## | R_3GwSVh0aohJdcEb | anonymous | EN-GB |
| True | ################## | R_roJqihukr5eD0M9 | anonymous | EN-GB |
| True | ################## | R_3nuZ23AydB0RrN1 | anonymous | EN-GB |
| True | ################## | R_2SjhuFgfp0pp9ci | anonymous | EN-GB |
| True | ################## | R_1LtHaBY9tTPkP8E | anonymous | EN-GB |
| True | ################## | R_12EcZxJnRCcZaqV | anonymous | EN-GB |
| True | ################## | R_1Ld6s28sGsDLuEI | anonymous | EN-GB |
| True | ################## | R_3CODmAWKQ9npDT7 | anonymous | EN-GB |
| True | ################## | R_erqffQTM9njNEIx | anonymous | EN-GB |
| True | ################## | R_25H4zmyOoDgItXN | anonymous | EN-GB |
| True | ################## | R_27lERJw8YW0Dhgb | anonymous | EN-GB |
| True | ################## | R_2QgLQO8UkqMYOAB | anonymous | EN-GB |
| True | ################## | R_2ypdnPlQZwF7KhU | anonymous | EN-GB |
| True | ################## | R_3kGTrzqPDx7Wrnm | preview | EN-GB |
| True | ################## | R_3PSSHLIsdA3DlnS | preview | EN-GB |
| True | ################## | R_2EbgpHM9GT6sPUq | anonymous | EN-GB |

| | | | | |
|---|---|---|---|---|
| True | ################# | R_0IgRqdrTj5Qoatz | anonymous | EN-GB |
| True | ################# | R_vNpsyM3E94kOejL | anonymous | EN-GB |

| Q1 Gender | Q2 Age |
|---|---|
| Female | 18 to 25 |
| Female | 18 to 25 |
| Female | 18 to 25 |
| Female | 18 to 25 |
| Female | 18 to 25 |
| Female | 18 to 25 |
| Female | 18 to 25 |
| Male | 18 to 25 |
| Female | 18 to 25 |
| Female | 18 to 25 |
| Female | 18 to 25 |
| Male | 18 to 25 |
| Female | 18 to 25 |
| Female | 18 to 25 |
| Female | 18 to 25 |
| Female | 18 to 25 |
| Female | 18 to 25 |
| Female | 18 to 25 |
| Male | 18 to 25 |
| Female | 18 to 25 |
| Female | 36 to 45 |
| Female | 18 to 25 |
| Female | 18 to 25 |
| Female | 18 to 25 |
| Female | 18 to 25 |
| Female | 18 to 25 |
| Male | 18 to 25 |
| Female | 18 to 25 |
| Female | 18 to 25 |
| Prefer not say | 18 to 25 |
| Male | 18 to 25 |
| Female | 18 to 25 |
| Male | 18 to 25 |
| Male | 18 to 25 |
| Male | 18 to 25 |
| Female | 18 to 25 |
| Male | 18 to 25 |
| Female | 18 to 25 |
| Female | 18 to 25 |
| Male | 18 to 25 |
| Female | 18 to 25 |
| Female | 18 to 25 |
| Female | 18 to 25 |
| Male | 18 to 25 |
| Female | 18 to 25 |
| Female | 18 to 25 |
| Female | 18 to 25 |

| | |
|---|---|
| Female | 18 to 25 |
| Female | 46 to 55 |
| Female | 18 to 25 |
| Male | 18 to 25 |
| Female | 18 to 25 |
| Female | 18 to 25 |
| Female | 18 to 25 |
| Female | 18 to 25 |
| Female | 18 to 25 |
| Female | 18 to 25 |
| Female | 18 to 25 |
| Female | 18 to 25 |
| Female | 18 to 25 |
| Male | 18 to 25 |
| Male | 18 to 25 |
| Male | 18 to 25 |
| Male | 18 to 25 |
| Female | 18 to 25 |
| Male | 18 to 25 |
| Male | 18 to 25 |
| Female | 18 to 25 |
| Female | 18 to 25 |
| Male | 18 to 25 |
| Female | 18 to 25 |
| Female | 18 to 25 |
| Female | 18 to 25 |
| Male | 18 to 25 |
| Female | 18 to 25 |
| Female | 18 to 25 |
| Female | 18 to 25 |
| Male | 18 to 25 |

| | |
|---|---|
| Male | 18 to 25 |
| Male | 18 to 25 |
| Male | 18 to 25 |
| Female | 18 to 25 |
| Female | 18 to 25 |
| Male | 18 to 25 |
| Male | 18 to 25 |
| Male | 18 to 25 |
| Male | 18 to 25 |
| Male | 18 to 25 |
| Male | 18 to 25 |
| Male | 18 to 25 |
| Male | 18 to 25 |
| Female | 18 to 25 |

| | |
|---|---|
| Female | 18 to 25 |
| Male | 18 to 25 |
| Male | 18 to 25 |
| Male | 18 to 25 |
| Male | 18 to 25 |
| Male | 18 to 25 |
| Female | 18 to 25 |
| Male | 18 to 25 |
| Male | 18 to 25 |
| Male | 18 to 25 |
| Male | 18 to 25 |
| Male | 18 to 25 |
| Male | 18 to 25 |
| Male | 18 to 25 |
| Male | 18 to 25 |
| Male | 18 to 25 |
| Male | 18 to 25 |
| Male | 18 to 25 |
| Male | 18 to 25 |
| Male | 18 to 25 |
| Female | 18 to 25 |
| Female | 18 to 25 |
| Female | 18 to 25 |
| Male | 18 to 25 |
| Female | 18 to 25 |
| Male | 18 to 25 |
| Male | 18 to 25 |
| Male | 18 to 25 |
| Female | 18 to 25 |
| Female | 18 to 25 |
| Male | 18 to 25 |
| Male | 18 to 25 |
| Male | 18 to 25 |
| Male | 18 to 25 |
| | |
| | |
| Male | |
| Male | 18 to 25 |
| Female | 18 to 25 |
| Male | 18 to 25 |
| Female | 18 to 25 |
| Male | 18 to 25 |
| Female | 18 to 25 |
| Male | 18 to 25 |
| Male | 18 to 25 |
| Male | 18 to 25 |
| Male | 18 to 25 |
| Male | 18 to 25 |

| | |
|---|---|
| Female | 18 to 25 |
| Female | 18 to 25 |
| Female | 18 to 25 |
| Male | 18 to 25 |
| Male | 18 to 25 |
| Female | 18 to 25 |
| Female | 26 to 35 |
| | |
| | |
| Female | 18 to 25 |
| Female | 18 to 25 |
| Male | 26 to 35 |
| Male | 18 to 25 |
| Female | 18 to 25 |
| Male | 18 to 25 |
| Male | 18 to 25 |
| Male | 18 to 25 |
| Female | 18 to 25 |
| Male | 18 to 25 |
| Female | 18 to 25 |
| Male | 18 to 25 |
| Male | 18 to 25 |
| Male | 18 to 25 |
| Female | 18 to 25 |
| Male | 18 to 25 |
| Male | 18 to 25 |
| Female | 18 to 25 |
| Male | 18 to 25 |
| Female | 18 to 25 |
| Male | 18 to 25 |
| Female | 18 to 25 |
| Male | 18 to 25 |
| Female | 18 to 25 |
| Male | 18 to 25 |
| Female | 18 to 25 |
| Male | 18 to 25 |
| Female | 18 to 25 |
| Female | |
| Female | 26 to 35 |
| Male | 46 to 55 |
| Male | 18 to 25 |
| Male | 18 to 25 |
| Male | 18 to 25 |
| Female | 18 to 25 |
| Female | 18 to 25 |
| Male | 18 to 25 |
| Female | 18 to 25 |
| Female | 18 to 25 |

| | |
|---|---|
| Male | 18 to 25 |
| Male | 18 to 25 |
| Male | 18 to 25 |
| Female | 18 to 25 |
| Female | 18 to 25 |
| Male | 18 to 25 |
| Male | 18 to 25 |
| Male | 18 to 25 |
| Female | 18 to 25 |
| Female | 18 to 25 |
| Male | 18 to 25 |
| Female | 18 to 25 |
| Female | 18 to 25 |
| Female | 18 to 25 |
| Female | 18 to 25 |
| Female | 18 to 25 |
| Male | 18 to 25 |
| Female | 18 to 25 |
| Female | 18 to 25 |
| Male | 18 to 25 |
| Female | 18 to 25 |
| Female | 18 to 25 |
| Female | 18 to 25 |
| Female | 18 to 25 |
| Female | 18 to 25 |
| Male | 18 to 25 |
| Male | 18 to 25 |
| Female | 18 to 25 |
| Female | 18 to 25 |
| Male | 18 to 25 |
| Female | 18 to 25 |
| Male | 18 to 25 |
| Female | 18 to 25 |
| Female | 18 to 25 |
| Female | 18 to 25 |
| Female | 18 to 25 |
| Female | 18 to 25 |
| Male | 46 to 55 |
| Female | 18 to 25 |
| Male | 18 to 25 |
| Female | 18 to 25 |
| Female | 26 to 35 |
| Female | 18 to 25 |
| Female | 18 to 25 |
| Female | 18 to 25 |
| Prefer not say | 18 to 25 |
| Female | 18 to 25 |
| Female | 26 to 35 |

| | |
|---|---|
| Female | 26 to 35 |
| Female | 18 to 25 |
| Female | 18 to 25 |
| Female | 18 to 25 |
| Female | 26 to 35 |
| Female | 18 to 25 |
| Female | 18 to 25 |
| Female | 18 to 25 |
| Female | 18 to 25 |
| Female | 18 to 25 |
| Female | 18 to 25 |
| Female | 18 to 25 |
| Female | 18 to 25 |
| Male | 18 to 25 |
| Female | 18 to 25 |
| Female | 18 to 25 |
| Female | 18 to 25 |
| Female | 26 to 35 |
| Female | 18 to 25 |
| Female | 18 to 25 |
| Female | 18 to 25 |
| Female | 18 to 25 |
| Female | 26 to 35 |
| Male | 18 to 25 |
| Female | 18 to 25 |
| Female | 18 to 25 |
| Female | 18 to 25 |
| Female | 18 to 25 |
| Male | 18 to 25 |
| Female | 18 to 25 |
| Female | 18 to 25 |
| Female | 18 to 25 |
| Female | 18 to 25 |
| Female | 18 to 25 |
| Female | 18 to 25 |
| Female | 18 to 25 |
| Female | 18 to 25 |
| Male | 18 to 25 |
| Female | 18 to 25 |
| Female | 18 to 25 |
| Female | 18 to 25 |
| Female | 18 to 25 |
| Female | 18 to 25 |
| Female | 18 to 25 |
| Female | 18 to 25 |
| Female | 18 to 25 |
| Male | 18 to 25 |
| Female | 18 to 25 |

| | |
|---|---|
| Female | 18 to 25 |
| Female | 18 to 25 |
| Female | 26 to 35 |
| Male | 18 to 25 |
| Female | 18 to 25 |
| Female | 18 to 25 |
| Female | 18 to 25 |
| Female | 18 to 25 |
| Female | 18 to 25 |
| Male | 18 to 25 |
| Female | 18 to 25 |
| Female | 18 to 25 |
| Female | 18 to 25 |
| Female | 18 to 25 |
| Female | 18 to 25 |
| Male | 18 to 25 |
| Female | 18 to 25 |
| Female | 18 to 25 |
| Male | 18 to 25 |
| Female | 18 to 25 |
| Female | 18 to 25 |
| Male | 18 to 25 |
| Female | 18 to 25 |
| Female | 18 to 25 |
| Male | 18 to 25 |
| Male | 18 to 25 |
| Female | 18 to 25 |
| Female | 18 to 25 |
| Female | 18 to 25 |
| Male | 18 to 25 |
| Female | 18 to 25 |
| Female | 18 to 25 |
| Female | 18 to 25 |
| Female | 18 to 25 |
| Female | 18 to 25 |
| Female | 18 to 25 |
| Female | 18 to 25 |
| Female | 18 to 25 |
| Male | 18 to 25 |
| Female | 18 to 25 |
| Female | 18 to 25 |
| Male | 18 to 25 |
| Female | 18 to 25 |
| Female | 26 to 35 |
| Male | 18 to 25 |
| Male | 18 to 25 |
| Male | 18 to 25 |
| Female | 18 to 25 |

| | |
|---|---|
| Female | 18 to 25 |
| Female | 18 to 25 |
| Female | 18 to 25 |
| Female | 18 to 25 |
| Female | 18 to 25 |
| Female | 18 to 25 |
| Male | 18 to 25 |
| Female | 18 to 25 |
| Female | 18 to 25 |
| Female | 18 to 25 |
| Female | 18 to 25 |
| Female | 18 to 25 |
| Male | 18 to 25 |
| Male | 18 to 25 |
| Female | 18 to 25 |
| Female | 18 to 25 |
| Male | 18 to 25 |
| Female | 18 to 25 |
| Male | 26 to 35 |
| Male | 18 to 25 |
| Female | 18 to 25 |
| Female | 18 to 25 |
| Female | 18 to 25 |
| Female | 18 to 25 |
| Male | 18 to 25 |
| Male | 18 to 25 |
| Female | 26 to 35 |
| Female | 18 to 25 |
| Female | 18 to 25 |
| Female | 18 to 25 |
| Male | 18 to 25 |
| Female | 18 to 25 |
| Female | 18 to 25 |
| Female | 18 to 25 |
| Female | 18 to 25 |
| Female | 18 to 25 |
| Male | 18 to 25 |
| Female | 18 to 25 |
| Female | 18 to 25 |
| Female | 18 to 25 |
| Female | 18 to 25 |
| Male | 18 to 25 |
| Female | 18 to 25 |
| Male | 18 to 25 |
| Male | 18 to 25 |
| Female | 18 to 25 |
| Male | 18 to 25 |
| Female | 18 to 25 |

| | |
|---|---|
| Female | 18 to 25 |
| Female | 18 to 25 |
| Female | 18 to 25 |
| Female | 18 to 25 |
| Male | 18 to 25 |
| Male | 18 to 25 |
| Male | 18 to 25 |
| | |
| Male | 18 to 25 |
| Male | 18 to 25 |
| Male | 18 to 25 |
| Female | 18 to 25 |
| Female | 18 to 25 |
| Female | 18 to 25 |
| Female | 18 to 25 |
| Female | 18 to 25 |
| Female | 18 to 25 |
| Female | 18 to 25 |
| Female | 18 to 25 |
| Female | 18 to 25 |
| Female | 18 to 25 |
| Male | 18 to 25 |
| Female | 18 to 25 |
| | |
| Male | 18 to 25 |
| Male | 18 to 25 |
| Male | 18 to 25 |
| Male | 18 to 25 |
| Male | 18 to 25 |
| Male | 18 to 25 |
| Male | 18 to 25 |
| Male | 18 to 25 |
| Male | 18 to 25 |
| Male | 18 to 25 |
| Male | 18 to 25 |
| | 18 to 25 |
| Female | 18 to 25 |
| Female | 18 to 25 |
| Male | 18 to 25 |
| Male | 18 to 25 |
| Male | 18 to 25 |
| Male | 18 to 25 |
| Female | 18 to 25 |
| Female | 18 to 25 |
| Male | 18 to 25 |
| Male | 18 to 25 |
| Male | 18 to 25 |
| Female | 18 to 25 |

| | |
|---|---|
| Female | 26 to 35 |
| Female | 18 to 25 |
| Male | 36 to 45 |
| Female | 18 to 25 |
| Female | 18 to 25 |
| Female | 46 to 55 |
| Female | 18 to 25 |
| Male | 18 to 25 |
| Female | 18 to 25 |
| Male | 18 to 25 |
| Female | 18 to 25 |
| Female | 18 to 25 |
| Female | 18 to 25 |
| Male | 18 to 25 |
| Female | 18 to 25 |
| Male | 18 to 25 |
| Male | 18 to 25 |
| Female | 18 to 25 |
| Male | 18 to 25 |
| Male | 18 to 25 |
| Female | 18 to 25 |
| Male | 18 to 25 |
| Female | 18 to 25 |
| | |
| Male | 18 to 25 |
| Male | 18 to 25 |
| Male | 18 to 25 |
| Female | 18 to 25 |
| Female | 18 to 25 |
| Female | 18 to 25 |
| Female | 18 to 25 |
| Female | 18 to 25 |
| Female | 18 to 25 |
| Female | 18 to 25 |
| Male | 18 to 25 |
| Female | 18 to 25 |
| Female | 18 to 25 |
| Female | 18 to 25 |
| Male | 18 to 25 |
| Female | 18 to 25 |
| Female | 18 to 25 |
| Male | 18 to 25 |
| Male | 18 to 25 |
| Female | 18 to 25 |
| Female | 18 to 25 |
| Male | 18 to 25 |
| Male | 18 to 25 |
| Male | 18 to 25 |

| | |
|---|---|
| Male | 18 to 25 |
| Male | 18 to 25 |
| Male | 18 to 25 |
| Male | 18 to 25 |
| Male | 18 to 25 |
| Male | 18 to 25 |
| Male | 18 to 25 |
| Male | 18 to 25 |
| Male | 18 to 25 |
| Male | 18 to 25 |
| Female | 18 to 25 |
| Male | 18 to 25 |
| Female | 18 to 25 |
| Female | 46 to 55 |
| Female | 18 to 25 |
| Female | 18 to 25 |
| Female | 18 to 25 |
| Female | 18 to 25 |
| Male | 18 to 25 |
| Female | 18 to 25 |
| Female | 36 to 45 |
| Female | 18 to 25 |
| Male | 18 to 25 |
| Female | 18 to 25 |
| Female | 18 to 25 |
| Female | 18 to 25 |
| Female | 18 to 25 |
| Male | 18 to 25 |
| Male | 18 to 25 |
| Male | 18 to 25 |
| Male | 18 to 25 |
| Male | 18 to 25 |
| Male | 18 to 25 |
| Female | 18 to 25 |
| Male | 18 to 25 |
| Female | 18 to 25 |
| Female | 18 to 25 |
| Male | 18 to 25 |
| Female | 18 to 25 |
| Female | 18 to 25 |
| Male | 18 to 25 |
| Male | 18 to 25 |
| Male | 18 to 25 |
| Male | 18 to 25 |
| Female | 18 to 25 |
| Male | 18 to 25 |
| Female | 26 to 35 |
| Male | 18 to 25 |

| | |
|---|---|
| Male | 18 to 25 |
| Female | 18 to 25 |

| Q3<br>Have you ever usedpsychostimulants drugs/ nootropics for memory/ concentration/ learning abilit |
| --- |
| No (Thank you for participating and ‘your survey is completed') |
| No (Thank you for participating and ‘your survey is completed') |
| Yes |
| No (Thank you for participating and ‘your survey is completed') |
| No (Thank you for participating and ‘your survey is completed') |
| No (Thank you for participating and ‘your survey is completed') |
| No (Thank you for participating and ‘your survey is completed') |
| No (Thank you for participating and ‘your survey is completed') |
| No (Thank you for participating and ‘your survey is completed') |
| No (Thank you for participating and ‘your survey is completed') |
| No (Thank you for participating and ‘your survey is completed') |
| No (Thank you for participating and ‘your survey is completed') |
| No (Thank you for participating and ‘your survey is completed') |
| No (Thank you for participating and ‘your survey is completed') |
| No (Thank you for participating and ‘your survey is completed') |
| No (Thank you for participating and ‘your survey is completed') |
| No (Thank you for participating and ‘your survey is completed') |
| No (Thank you for participating and ‘your survey is completed') |
| No (Thank you for participating and ‘your survey is completed') |
| Yes |
| No (Thank you for participating and ‘your survey is completed') |
| No (Thank you for participating and ‘your survey is completed') |
| No (Thank you for participating and ‘your survey is completed') |
| No (Thank you for participating and ‘your survey is completed') |
| No (Thank you for participating and ‘your survey is completed') |
| No (Thank you for participating and ‘your survey is completed') |
| Yes |
| No (Thank you for participating and ‘your survey is completed') |
| No (Thank you for participating and ‘your survey is completed') |
| No (Thank you for participating and ‘your survey is completed') |
| Yes |
| No (Thank you for participating and ‘your survey is completed') |
| No (Thank you for participating and ‘your survey is completed') |
| No (Thank you for participating and ‘your survey is completed') |
| Do not know/unsure |
| No (Thank you for participating and ‘your survey is completed') |
| No (Thank you for participating and ‘your survey is completed') |
| No (Thank you for participating and ‘your survey is completed') |
| No (Thank you for participating and ‘your survey is completed') |
| No (Thank you for participating and ‘your survey is completed') |
| No (Thank you for participating and ‘your survey is completed') |
| No (Thank you for participating and ‘your survey is completed') |
| No (Thank you for participating and ‘your survey is completed') |
| No (Thank you for participating and ‘your survey is completed') |
| Do not know/unsure |
| No (Thank you for participating and ‘your survey is completed') |
| No (Thank you for participating and ‘your survey is completed') |

Yes
No (Thank you for participating and ‘your survey is completed')
Yes
No (Thank you for participating and ‘your survey is completed')
No (Thank you for participating and ‘your survey is completed')
Yes
No (Thank you for participating and ‘your survey is completed')
Do not know/unsure
No (Thank you for participating and ‘your survey is completed')
No (Thank you for participating and ‘your survey is completed')
No (Thank you for participating and ‘your survey is completed')
No (Thank you for participating and ‘your survey is completed')
Yes
Yes
No (Thank you for participating and ‘your survey is completed')
No (Thank you for participating and ‘your survey is completed')
No (Thank you for participating and ‘your survey is completed')
Yes
Yes
No (Thank you for participating and ‘your survey is completed')
No (Thank you for participating and ‘your survey is completed')
No (Thank you for participating and ‘your survey is completed')
No (Thank you for participating and ‘your survey is completed')
No (Thank you for participating and ‘your survey is completed')
No (Thank you for participating and ‘your survey is completed')
No (Thank you for participating and ‘your survey is completed')
No (Thank you for participating and ‘your survey is completed')
No (Thank you for participating and ‘your survey is completed')
No (Thank you for participating and ‘your survey is completed')
No (Thank you for participating and ‘your survey is completed')
Yes

Yes
Yes
Yes
No (Thank you for participating and ‘your survey is completed')
Yes
Yes
Yes
Yes
Yes
Yes
Yes
Yes
Yes
Yes

No (Thank you for participating and ‘your survey is completed')
Yes
Yes
Yes
Yes
Yes
No (Thank you for participating and ‘your survey is completed')
Yes
Yes
Yes
No (Thank you for participating and ‘your survey is completed')
No (Thank you for participating and ‘your survey is completed')
Yes
Yes
No (Thank you for participating and ‘your survey is completed')
Yes
Yes
Yes
Yes
Yes
No (Thank you for participating and ‘your survey is completed')
No (Thank you for participating and ‘your survey is completed')
Yes
No (Thank you for participating and ‘your survey is completed')
No (Thank you for participating and ‘your survey is completed')
Yes
No (Thank you for participating and ‘your survey is completed')
No (Thank you for participating and ‘your survey is completed')
No (Thank you for participating and ‘your survey is completed')
No (Thank you for participating and ‘your survey is completed')
Yes
No (Thank you for participating and ‘your survey is completed')
Yes
No (Thank you for participating and ‘your survey is completed')

Yes
Yes
No (Thank you for participating and ‘your survey is completed')
No (Thank you for participating and ‘your survey is completed')
No (Thank you for participating and ‘your survey is completed')
No (Thank you for participating and ‘your survey is completed')
No (Thank you for participating and ‘your survey is completed')
Yes
No (Thank you for participating and ‘your survey is completed')
No (Thank you for participating and ‘your survey is completed')
Yes
Yes

No (Thank you for participating and ‘your survey is completed')
No (Thank you for participating and ‘your survey is completed')
Yes
Yes
Yes
No (Thank you for participating and ‘your survey is completed')
No (Thank you for participating and ‘your survey is completed')

No (Thank you for participating and ‘your survey is completed')
No (Thank you for participating and ‘your survey is completed')
No (Thank you for participating and ‘your survey is completed')
No (Thank you for participating and ‘your survey is completed')
No (Thank you for participating and ‘your survey is completed')
No (Thank you for participating and ‘your survey is completed')
Yes
Yes
No (Thank you for participating and ‘your survey is completed')
No (Thank you for participating and ‘your survey is completed')
No (Thank you for participating and ‘your survey is completed')
Yes
Yes
No (Thank you for participating and ‘your survey is completed')
No (Thank you for participating and ‘your survey is completed')
No (Thank you for participating and ‘your survey is completed')
No (Thank you for participating and ‘your survey is completed')
No (Thank you for participating and ‘your survey is completed')
Yes
No (Thank you for participating and ‘your survey is completed')
No (Thank you for participating and ‘your survey is completed')
No (Thank you for participating and ‘your survey is completed')
Yes
No (Thank you for participating and ‘your survey is completed')
No (Thank you for participating and ‘your survey is completed')
No (Thank you for participating and ‘your survey is completed')
No (Thank you for participating and ‘your survey is completed')
No (Thank you for participating and ‘your survey is completed')

No (Thank you for participating and ‘your survey is completed')
Yes
No (Thank you for participating and ‘your survey is completed')
No (Thank you for participating and ‘your survey is completed')
No (Thank you for participating and ‘your survey is completed')
No (Thank you for participating and ‘your survey is completed')
No (Thank you for participating and ‘your survey is completed')
No (Thank you for participating and ‘your survey is completed')
No (Thank you for participating and ‘your survey is completed')
Yes

No (Thank you for participating and ‘your survey is completed')
Yes
No (Thank you for participating and ‘your survey is completed')
Yes
No (Thank you for participating and ‘your survey is completed')
Yes
No (Thank you for participating and ‘your survey is completed')
Yes
No (Thank you for participating and ‘your survey is completed')
No (Thank you for participating and ‘your survey is completed')
No (Thank you for participating and ‘your survey is completed')
No (Thank you for participating and ‘your survey is completed')
No (Thank you for participating and ‘your survey is completed')
No (Thank you for participating and ‘your survey is completed')
No (Thank you for participating and ‘your survey is completed')
No (Thank you for participating and ‘your survey is completed')
No (Thank you for participating and ‘your survey is completed')
No (Thank you for participating and ‘your survey is completed')
No (Thank you for participating and ‘your survey is completed')
No (Thank you for participating and ‘your survey is completed')
No (Thank you for participating and ‘your survey is completed')
No (Thank you for participating and ‘your survey is completed')
No (Thank you for participating and ‘your survey is completed')
No (Thank you for participating and ‘your survey is completed')
No (Thank you for participating and ‘your survey is completed')
No (Thank you for participating and ‘your survey is completed')
Yes
No (Thank you for participating and ‘your survey is completed')
No (Thank you for participating and ‘your survey is completed')
No (Thank you for participating and ‘your survey is completed')
No (Thank you for participating and ‘your survey is completed')
Do not know/unsure
Yes
No (Thank you for participating and ‘your survey is completed')
No (Thank you for participating and ‘your survey is completed')
No (Thank you for participating and ‘your survey is completed')
No (Thank you for participating and ‘your survey is completed')
No (Thank you for participating and ‘your survey is completed')
No (Thank you for participating and ‘your survey is completed')
No (Thank you for participating and ‘your survey is completed')
No (Thank you for participating and ‘your survey is completed')
Yes
No (Thank you for participating and ‘your survey is completed')
No (Thank you for participating and ‘your survey is completed')
No (Thank you for participating and ‘your survey is completed')
No (Thank you for participating and ‘your survey is completed')
No (Thank you for participating and ‘your survey is completed')
No (Thank you for participating and ‘your survey is completed')

No (Thank you for participating and 'your survey is completed')
No (Thank you for participating and 'your survey is completed')
Yes
No (Thank you for participating and 'your survey is completed')
Yes
No (Thank you for participating and 'your survey is completed')
Yes
No (Thank you for participating and 'your survey is completed')
No (Thank you for participating and 'your survey is completed')
No (Thank you for participating and 'your survey is completed')
No (Thank you for participating and 'your survey is completed')
No (Thank you for participating and 'your survey is completed')
No (Thank you for participating and 'your survey is completed')
No (Thank you for participating and 'your survey is completed')
No (Thank you for participating and 'your survey is completed')
No (Thank you for participating and 'your survey is completed')
No (Thank you for participating and 'your survey is completed')
No (Thank you for participating and 'your survey is completed')
No (Thank you for participating and 'your survey is completed')
No (Thank you for participating and 'your survey is completed')
No (Thank you for participating and 'your survey is completed')
Do not know/unsure
No (Thank you for participating and 'your survey is completed')
No (Thank you for participating and 'your survey is completed')
No (Thank you for participating and 'your survey is completed')
No (Thank you for participating and 'your survey is completed')
No (Thank you for participating and 'your survey is completed')
No (Thank you for participating and 'your survey is completed')
No (Thank you for participating and 'your survey is completed')
No (Thank you for participating and 'your survey is completed')
No (Thank you for participating and 'your survey is completed')
No (Thank you for participating and 'your survey is completed')
No (Thank you for participating and 'your survey is completed')
Yes
No (Thank you for participating and 'your survey is completed')
No (Thank you for participating and 'your survey is completed')
No (Thank you for participating and 'your survey is completed')
No (Thank you for participating and 'your survey is completed')
No (Thank you for participating and 'your survey is completed')
No (Thank you for participating and 'your survey is completed')
No (Thank you for participating and 'your survey is completed')
No (Thank you for participating and 'your survey is completed')
No (Thank you for participating and 'your survey is completed')
No (Thank you for participating and 'your survey is completed')
No (Thank you for participating and 'your survey is completed')
No (Thank you for participating and 'your survey is completed')
No (Thank you for participating and 'your survey is completed')
No (Thank you for participating and 'your survey is completed')

No (Thank you for participating and ‘your survey is completed')
Yes
No (Thank you for participating and ‘your survey is completed')
No (Thank you for participating and ‘your survey is completed')
No (Thank you for participating and ‘your survey is completed')
No (Thank you for participating and ‘your survey is completed')
No (Thank you for participating and ‘your survey is completed')
No (Thank you for participating and ‘your survey is completed')
No (Thank you for participating and ‘your survey is completed')
No (Thank you for participating and ‘your survey is completed')
No (Thank you for participating and ‘your survey is completed')
No (Thank you for participating and ‘your survey is completed')
No (Thank you for participating and ‘your survey is completed')
No (Thank you for participating and ‘your survey is completed')
No (Thank you for participating and ‘your survey is completed')
No (Thank you for participating and ‘your survey is completed')
Yes
No (Thank you for participating and ‘your survey is completed')
Yes
No (Thank you for participating and ‘your survey is completed')
No (Thank you for participating and ‘your survey is completed')
No (Thank you for participating and ‘your survey is completed')
No (Thank you for participating and ‘your survey is completed')
No (Thank you for participating and ‘your survey is completed')
No (Thank you for participating and ‘your survey is completed')
No (Thank you for participating and ‘your survey is completed')
No (Thank you for participating and ‘your survey is completed')
No (Thank you for participating and ‘your survey is completed')
No (Thank you for participating and ‘your survey is completed')
No (Thank you for participating and ‘your survey is completed')
No (Thank you for participating and ‘your survey is completed')
No (Thank you for participating and ‘your survey is completed')
No (Thank you for participating and ‘your survey is completed')
No (Thank you for participating and ‘your survey is completed')
No (Thank you for participating and ‘your survey is completed')
No (Thank you for participating and ‘your survey is completed')
No (Thank you for participating and ‘your survey is completed')
No (Thank you for participating and ‘your survey is completed')
No (Thank you for participating and ‘your survey is completed')
No (Thank you for participating and ‘your survey is completed')
No (Thank you for participating and ‘your survey is completed')
No (Thank you for participating and ‘your survey is completed')
No (Thank you for participating and ‘your survey is completed')
No (Thank you for participating and ‘your survey is completed')
Yes
No (Thank you for participating and ‘your survey is completed')
No (Thank you for participating and ‘your survey is completed')
No (Thank you for participating and ‘your survey is completed')

No (Thank you for participating and ‘your survey is completed')
No (Thank you for participating and ‘your survey is completed')
No (Thank you for participating and ‘your survey is completed')
No (Thank you for participating and ‘your survey is completed')
No (Thank you for participating and ‘your survey is completed')
No (Thank you for participating and ‘your survey is completed')
Yes
No (Thank you for participating and ‘your survey is completed')
No (Thank you for participating and ‘your survey is completed')
Do not know/unsure
No (Thank you for participating and ‘your survey is completed')
No (Thank you for participating and ‘your survey is completed')
No (Thank you for participating and ‘your survey is completed')
Yes
No (Thank you for participating and ‘your survey is completed')
Yes
Yes
No (Thank you for participating and ‘your survey is completed')
No (Thank you for participating and ‘your survey is completed')
No (Thank you for participating and ‘your survey is completed')
Yes
No (Thank you for participating and ‘your survey is completed')
No (Thank you for participating and ‘your survey is completed')
No (Thank you for participating and ‘your survey is completed')
No (Thank you for participating and ‘your survey is completed')
No (Thank you for participating and ‘your survey is completed')
No (Thank you for participating and ‘your survey is completed')
No (Thank you for participating and ‘your survey is completed')
No (Thank you for participating and ‘your survey is completed')
No (Thank you for participating and ‘your survey is completed')
No (Thank you for participating and ‘your survey is completed')
No (Thank you for participating and ‘your survey is completed')
No (Thank you for participating and ‘your survey is completed')
No (Thank you for participating and ‘your survey is completed')
No (Thank you for participating and ‘your survey is completed')
No (Thank you for participating and ‘your survey is completed')
No (Thank you for participating and ‘your survey is completed')
No (Thank you for participating and ‘your survey is completed')
No (Thank you for participating and ‘your survey is completed')
No (Thank you for participating and ‘your survey is completed')
No (Thank you for participating and ‘your survey is completed')
No (Thank you for participating and ‘your survey is completed')
No (Thank you for participating and ‘your survey is completed')
No (Thank you for participating and ‘your survey is completed')
No (Thank you for participating and ‘your survey is completed')
No (Thank you for participating and ‘your survey is completed')
No (Thank you for participating and ‘your survey is completed')
No (Thank you for participating and ‘your survey is completed')

No (Thank you for participating and ‘your survey is completed')
No (Thank you for participating and ‘your survey is completed')
No (Thank you for participating and ‘your survey is completed')
No (Thank you for participating and ‘your survey is completed')
No (Thank you for participating and ‘your survey is completed')
No (Thank you for participating and ‘your survey is completed')
No (Thank you for participating and ‘your survey is completed')

Yes
Yes
Yes
No (Thank you for participating and ‘your survey is completed')
No (Thank you for participating and ‘your survey is completed')
No (Thank you for participating and ‘your survey is completed')
No (Thank you for participating and ‘your survey is completed')
No (Thank you for participating and ‘your survey is completed')
No (Thank you for participating and ‘your survey is completed')
No (Thank you for participating and ‘your survey is completed')
Do not know/unsure
No (Thank you for participating and ‘your survey is completed')
No (Thank you for participating and ‘your survey is completed')
No (Thank you for participating and ‘your survey is completed')
No (Thank you for participating and ‘your survey is completed')

No (Thank you for participating and ‘your survey is completed')
No (Thank you for participating and ‘your survey is completed')
Yes
Yes
Yes
Yes
Yes
Yes
Yes
Yes
Yes
Yes
No (Thank you for participating and ‘your survey is completed')
No (Thank you for participating and ‘your survey is completed')
No (Thank you for participating and ‘your survey is completed')
No (Thank you for participating and ‘your survey is completed')
Yes
No (Thank you for participating and ‘your survey is completed')
Yes
No (Thank you for participating and ‘your survey is completed')
No (Thank you for participating and ‘your survey is completed')
No (Thank you for participating and ‘your survey is completed')
No (Thank you for participating and ‘your survey is completed')
No (Thank you for participating and ‘your survey is completed')

No (Thank you for participating and ‘your survey is completed')
No (Thank you for participating and ‘your survey is completed')
No (Thank you for participating and ‘your survey is completed')
No (Thank you for participating and ‘your survey is completed')
No (Thank you for participating and ‘your survey is completed')
No (Thank you for participating and ‘your survey is completed')
No (Thank you for participating and ‘your survey is completed')
Yes
No (Thank you for participating and ‘your survey is completed')
Yes
No (Thank you for participating and ‘your survey is completed')
No (Thank you for participating and ‘your survey is completed')
No (Thank you for participating and ‘your survey is completed')
Yes
No (Thank you for participating and ‘your survey is completed')
No (Thank you for participating and ‘your survey is completed')
No (Thank you for participating and ‘your survey is completed')
Yes
No (Thank you for participating and ‘your survey is completed')
No (Thank you for participating and ‘your survey is completed')
No (Thank you for participating and ‘your survey is completed')
No (Thank you for participating and ‘your survey is completed')
Yes

Yes
Yes
Yes
No (Thank you for participating and ‘your survey is completed')
No (Thank you for participating and ‘your survey is completed')
No (Thank you for participating and ‘your survey is completed')
No (Thank you for participating and ‘your survey is completed')
No (Thank you for participating and ‘your survey is completed')
No (Thank you for participating and ‘your survey is completed')
No (Thank you for participating and ‘your survey is completed')
Yes
No (Thank you for participating and ‘your survey is completed')
No (Thank you for participating and ‘your survey is completed')
No (Thank you for participating and ‘your survey is completed')
Yes
Yes
No (Thank you for participating and ‘your survey is completed')
No (Thank you for participating and ‘your survey is completed')
No (Thank you for participating and ‘your survey is completed')
No (Thank you for participating and ‘your survey is completed')
No (Thank you for participating and ‘your survey is completed')
Yes
No (Thank you for participating and ‘your survey is completed')
No (Thank you for participating and ‘your survey is completed')

No (Thank you for participating and ‘your survey is completed')
No (Thank you for participating and ‘your survey is completed')
No (Thank you for participating and ‘your survey is completed')
No (Thank you for participating and ‘your survey is completed')
No (Thank you for participating and ‘your survey is completed')
No (Thank you for participating and ‘your survey is completed')
No (Thank you for participating and ‘your survey is completed')
No (Thank you for participating and ‘your survey is completed')
No (Thank you for participating and ‘your survey is completed')
No (Thank you for participating and ‘your survey is completed')
No (Thank you for participating and ‘your survey is completed')
No (Thank you for participating and ‘your survey is completed')
No (Thank you for participating and ‘your survey is completed')
No (Thank you for participating and ‘your survey is completed')
No (Thank you for participating and ‘your survey is completed')
No (Thank you for participating and ‘your survey is completed')
No (Thank you for participating and ‘your survey is completed')
No (Thank you for participating and ‘your survey is completed')
Yes
No (Thank you for participating and ‘your survey is completed')
Yes
No (Thank you for participating and ‘your survey is completed')
Yes
No (Thank you for participating and ‘your survey is completed')
Yes
No (Thank you for participating and ‘your survey is completed')
No (Thank you for participating and ‘your survey is completed')
No (Thank you for participating and ‘your survey is completed')
Yes
No (Thank you for participating and ‘your survey is completed')
Yes
Yes
No (Thank you for participating and ‘your survey is completed')
Yes
Yes
Yes
No (Thank you for participating and ‘your survey is completed')
No (Thank you for participating and ‘your survey is completed')
No (Thank you for participating and ‘your survey is completed')
Yes
Yes
No (Thank you for participating and ‘your survey is completed')
Yes
No (Thank you for participating and ‘your survey is completed')
Yes
No (Thank you for participating and ‘your survey is completed')
Yes
Yes

Yes
Yes

Yes

Yes

Yes

Yes

Yes

Yes
Yes

Yes
Yes
Yes

Yes
Yes

Yes

Yes

Yes
Yes
Yes
Yes

Q4
If 'Yes', which one? (select all that apply)

Alpha Brain/vitamin B6,Cobalamin/ vitamin B12

Cobalamin/ vitamin B12

Super strength caffeine pills

Super strength caffeine pills,Alpha Brain/vitamin B6,Cobalamin/ vitamin B12,Other drug or substance used

Cobalamin/ vitamin B12

Super strength caffeine pills

Super strength caffeine pills,Alpha Brain/vitamin B6,Cobalamin/ vitamin B12

Super strength caffeine pills

Alpha Brain/vitamin B6,Cobalamin/ vitamin B12

Other drug or substance used

Ritalin/amphetamine
MDMA/ ecstasy,Cobalamin/ vitamin B12

Super strength caffeine pills,Guarana
Modafinil

Modafinil,Cobalamin/ vitamin B12

Adderall/amphetamines mixture
Super strength caffeine pills
Super strength caffeine pills

Super strength caffeine pills,Cobalamin/ vitamin B12
Modafinil
Modafinil,Super strength caffeine pills
Modafinil,Super strength caffeine pills,Cobalamin/ vitamin B12
Modafinil,Cobalamin/ vitamin B12
Cobalamin/ vitamin B12
Modafinil
Modafinil,Cobalamin/ vitamin B12
Modafinil,Cobalamin/ vitamin B12
Super strength caffeine pills

Modafinil,Super strength caffeine pills
Modafinil,Super strength caffeine pills,Guarana
Super strength caffeine pills,Cobalamin/ vitamin B12
Modafinil,Super strength caffeine pills,Cobalamin/ vitamin B12
Super strength caffeine pills

Modafinil,Cobalamin/ vitamin B12
Modafinil
Adderall/amphetamines mixture

Modafinil,Super strength caffeine pills
Adderall/amphetamines mixture

Adderall/amphetamines mixture,Ritalin/amphetamine
Modafinil,Cobalamin/ vitamin B12
Modafinil,Cobalamin/ vitamin B12
Modafinil,Super strength caffeine pills
Adderall/amphetamines mixture,Ritalin/amphetamine

Modafinil,Super strength caffeine pills

Adderall/amphetamines mixture,Ritalin/amphetamine

Modafinil,Cobalamin/ vitamin B12

MDMA/ ecstasy

Modafinil
Modafinil,Super strength caffeine pills

Ritalin/amphetamine

Modafinil
Modafinil

Guarana,Piracetam
Adderall/amphetamines mixture
Modafinil

Modafinil
Modafinil,Super strength caffeine pills

Modafinil
Modafinil

Modafinil,Ritalin/amphetamine

Modafinil,Ritalin/amphetamine

Modafinil

Super strength caffeine pills

Modafinil,Super strength caffeine pills

Super strength caffeine pills

Modafinil,Guarana

Modafinil,Super strength caffeine pills

Other drug or substance used

Other drug or substance used
Cobalamin/ vitamin B12

Ritalin/amphetamine,Alpha Brain/vitamin B6,Cobalamin/ vitamin B12

Cobalamin/ vitamin B12

Ritalin/amphetamine

Cobalamin/ vitamin B12

Alpha Brain/vitamin B6,Cobalamin/ vitamin B12

Cobalamin/ vitamin B12,Other drug or substance used

Cobalamin/ vitamin B12

MDMA/ ecstasy,Alpha Brain/vitamin B6

Alpha Brain/vitamin B6

Ritalin/amphetamine

Super strength caffeine pills

Cobalamin/ vitamin B12

Super strength caffeine pills

Other drug or substance used
Other drug or substance used

Super strength caffeine pills

Modafinil
Super strength caffeine pills,Cobalamin/ vitamin B12
Super strength caffeine pills,Cobalamin/ vitamin B12

Modafinil,Cobalamin/ vitamin B12
Super strength caffeine pills,Cobalamin/ vitamin B12
Super strength caffeine pills,Cobalamin/ vitamin B12
Super strength caffeine pills,Cobalamin/ vitamin B12
Modafinil,Cobalamin/ vitamin B12
Adderall/amphetamines mixture
Modafinil,Alpha Brain/vitamin B6
Super strength caffeine pills,Cobalamin/ vitamin B12
Modafinil,Cobalamin/ vitamin B12
Modafinil,Cobalamin/ vitamin B12

Adderall/amphetamines mixture,Ritalin/amphetamine

Super strength caffeine pills,Alpha Brain/vitamin B6,Cobalamin/ vitamin B12

Super strength caffeine pills,Cobalamin/ vitamin B12

Ritalin/amphetamine

Super strength caffeine pills,Cobalamin/ vitamin B12

Adderall/amphetamines mixture

Modafinil

Modafinil,Cobalamin/ vitamin B12,Vinpocetine
Adderall/amphetamines mixture
Ritalin/amphetamine

Super strength caffeine pills,Cobalamin/ vitamin B12

Super strength caffeine pills,Guarana,Cobalamin/ vitamin B12
Super strength caffeine pills,Cobalamin/ vitamin B12

Super strength caffeine pills,Cobalamin/ vitamin B12

Other drug or substance used

Modafinil,Super strength caffeine pills,Cobalamin/ vitamin B12

Super strength caffeine pills,Alpha Brain/vitamin B6

Super strength caffeine pills

Super strength caffeine pills
Alpha Brain/vitamin B6

Cobalamin/ vitamin B12
Cobalamin/ vitamin B12,Vinpocetine
Alpha Brain/vitamin B6,Cobalamin/ vitamin B12

Super strength caffeine pills,Vinpocetine
Modafinil

Modafinil

Cobalamin/ vitamin B12

Modafinil
Modafinil

Super strength caffeine pills,Alpha Brain/vitamin B6
Super strength caffeine pills

| Q5<br>Nationality - Selected Choice | Q5_6_TEXT<br>Nationality - Other, (please specify) - Text |
|---|---|
| Other, (please specify) | Indian |
| Arab Nationality | |
| Arab Nationality | |
| UAE | |
| | |
| | |
| Other, (please specify) | Indian |
| | |
| | |
| Arab Nationality | |
| | |
| | |
| Other, (please specify) | Tanzania |
| | |
| | |
| | |
| | |
| | |
| UAE | |
| Arab Nationality | |
| UAE | |
| Arab Nationality | |
| Arab Nationality | |
| Arab Nationality | |
| Other, (please specify) | Ras Al Khaimah |
| | |
| Other, (please specify) | Indian |
| Other, (please specify) | Pakistani |
| Other, (please specify) | Dubai |
| | |
| Arab Nationality | |
| | |
| | |
| | |
| Arab Nationality | |
| Arab Nationality | |
| Other, (please specify) | indain |
| | |
| | |
| | |
| Other, (please specify) | Indian |
| | |
| UK | |
| | |
| UK | |

UK

UAE

UK

UK

UK
UK

Other, (please specify) Indian
Other, (please specify) Indian
Arab Nationality

UK
UAE

Arab Nationality

UAE

UK
UAE

UAE
UAE
UAE

UAE
UAE
UAE
UAE
UAE
Arab Nationality
Arab Nationality
UAE
UAE
UAE

UAE
Arab Nationality
UAE
UAE
UAE
UAE

UAE
Arab Nationality
Arab Nationality

UAE
UAE

UAE
UAE
UAE
UAE
Arab Nationality

UAE

UAE

Arab Nationality

Arab Nationality

Arab Nationality
Arab Nationality

Other, (please specify) India

UAE
UAE

UAE
UAE
UAE

UK
Arab Nationality

UAE
UAE

Arab Nationality

Arab Nationality

Arab Nationality

UAE

| | |
|---|---|
| UAE | |
| UAE | |
| Arab Nationality | |
| UAE | |
| Arab Nationality | |
| Other, (please specify) | Indian |
| Other, (please specify) | Indian |
| Other, (please specify) | Indian |
| Arab Nationality | |
| Other, (please specify) | Tanzanian |
| Other, (please specify) | Bangladesh |
| USA | |
| UK | |
| Other, (please specify) | South African |
| Arab Nationality | |
| Other, (please specify) | African |
| Other, (please specify) | indian |
| Other, (please specify) | Indian |
| Other, (please specify) | Nigerian |
| Other, (please specify) | Indian |

| | |
|---|---|
| Arab Nationality | |
| Other, (please specify) | indian |
| Arab Nationality | |
| | |
| UAE | |
| | |
| | |
| | |
| | |
| | |
| | |
| Arab Nationality | |
| Other, (please specify) | Indian |
| Other, (please specify) | India |
| UAE | |
| | |
| | |
| Other, (please specify) | Indian |
| | |
| | |
| UAE | |
| | |
| UK | |
| | |
| | |
| Other, (please specify) | Nigerian |
| | |
| UAE | |
| | |
| | |
| Other, (please specify) | Pakistani |
| UAE | |
| Other, (please specify) | srilanka |
| Other, (please specify) | Indian |
| Arab Nationality | |
| Arab Nationality | |
| | |
| Other, (please specify) | Nigeria |
| | |
| Arab Nationality | |
| | |
| Arab Nationality | |
| | |
| UAE | |
| | |
| Other, (please specify) | Indian |

| | |
|---|---|
| Other, (please specify) | India |
| Other, (please specify) | India |
| Other, (please specify) | Indian |
| | |
| Other, (please specify) | Nigerian |
| Other, (please specify) | |
| Other, (please specify) | Indian |
| Other, (please specify) | Abu Dhabi |
| Arab Nationality | |
| Arab Nationality | |
| Other, (please specify) | Philippines |
| UAE | |
| Other, (please specify) | INDIAN |
| Other, (please specify) | Pakistani |
| Arab Nationality | |
| Other, (please specify) | Pakistani |
| Arab Nationality | |
| Other, (please specify) | Indian |
| Other, (please specify) | |
| Other, (please specify) | Nigerian |
| Other, (please specify) | Indian |
| Other, (please specify) | |
| Other, (please specify) | Nigeria |
| Arab Nationality | |
| Other, (please specify) | MI |

| | |
|---|---|
| Other, (please specify) | Indian |
| | |
| | |
| | |
| | |
| Other, (please specify) | Indian |
| Other, (please specify) | India |
| Other, (please specify) | pakistan |
| Other, (please specify) | Indian |
| Arab Nationality | |
| Other, (please specify) | chinese and syrian |
| Arab Nationality | |
| | |
| Arab Nationality | |
| | |
| Other, (please specify) | INDIAN |
| EU | |
| Other, (please specify) | |
| | |
| | |
| Arab Nationality | |
| Other, (please specify) | ZImbabwe |
| Arab Nationality | |
| Other, (please specify) | Indian |
| | |
| Other, (please specify) | Indian |
| | |
| | |
| | |
| Other, (please specify) | pakistan |
| Arab Nationality | |
| | |
| | |
| | |
| | |
| Other, (please specify) | Indian |
| | |
| | |
| Other, (please specify) | |
| | |
| | |
| | |
| Other, (please specify) | Iran |
| | |
| USA | |
| Other, (please specify) | Indian |

| | | |
|---|---|---|
| Other, (please specify) | | إمارة دبيّ |
| UAE | | |
| UAE | | |
| Arab Nationality | | |
| Other, (please specify) | India | |
| Other, (please specify) | India | |
| Other, (please specify) | Persian | |
| Other, (please specify) | Indian | |
| UAE | | |
| UAE | | |
| Arab Nationality | | |
| Arab Nationality | | |
| Arab Nationality | | |
| Other, (please specify) | Indian | |
| UAE | | |
| UAE | | |
| Arab Nationality | | |
| Arab Nationality | | |
| Other, (please specify) | Indian | |
| Other, (please specify) | Indian | |
| Other, (please specify) | Iran | |
| Arab Nationality | | |
| Other, (please specify) | INDIAN | |
| Other, (please specify) | Indian | |

| | |
|---|---|
| Arab Nationality | |
| Arab Nationality | |
| Arab Nationality | |
| Arab Nationality | |
| Other, (please specify) | Pakistan |
| Arab Nationality | |
| UAE | |
| Arab Nationality | |
| Other, (please specify) | india |
| UAE | |
| Arab Nationality | |
| Arab Nationality | |
| Other, (please specify) | Pakistan |
| Arab Nationality | |
| UAE | |

| | |
|---|---|
| Other, (please specify) | |
| Other, (please specify) | Indian |
| | |
| Other, (please specify) | India |
| | |
| UAE | |
| | |
| Other, (please specify) | Palastine |
| | |
| UAE | |
| | |
| UAE | |
| | |
| | |
| | |
| Arab Nationality | |
| | |
| UAE | |
| Arab Nationality | |
| | |
| Arab Nationality | |
| UAE | |
| UK | |
| | |
| | |
| | |
| UAE | |
| Arab Nationality | |
| | |
| Arab Nationality | |
| | |
| Arab Nationality | |
| | |
| UK | |
| Arab Nationality | |

| | |
|---|---|
| Other, (please specify) | Indian |
| Arab Nationality | |

| Q6 | Q7 |
|---|---|
| What degree are youstudyi | Year of Education |
| Medicine | Third year |
| 4 | Third year |
| 3 | Fourth year |
| 3 | Fourth year |
| | |
| | |
| Medicine | Third year |
| | |
| | |
| Medicine | Fourth year |
| | |
| | |
| Medicine | First year |
| | |
| | |
| | |
| | |
| | |
| Medicine | Fourth year |
| Medicine | Fourth year |
| 4 | First year |
| 3 | First year |
| Pharmacy | Third year |
| 3 | Second year |
| Medicine | Third year |
| | |
| Medicine | Fourth year |
| 4 | First year |
| | |
| | |
| Medicine | Second year |
| | |
| | |
| | |
| Medicine | First year |
| 4 | Fourth year |
| Medicine | Fourth year |
| | |
| | |
| | |
| Medicine | Third year |
| | |
| Pharmacy | First year |
| | |
| Pharmacy | Third year |

| | | |
|---|---|---|
| Pharmacy | | First year |
| Pharmacy | | Second year |
| Pharmacy | | Second year |
| Pharmacy | | First year |
| Pharmacy | | Second year |
| Pharmacy | | Fourth year |
| Medicine | | First year |
| Medicine | | Fourth year |
| | 3 | Fourth year |
| Pharmacy | | Third year |
| Medicine | | Fourth year |
| | 3 | First year |
| Medicine | | Second year |
| Medicine | | Third year |
| Medicine | | Fourth year |
| Medicine | | Fourth year |
| Medicine | | Second year |
| Medicine | | Fourth year |
| Medicine | | Third year |
| Medicine | | Fourth year |
| Medicine | | Fifth year |
| Medicine | | Fourth year |
| Medicine | | Fourth year |
| Medicine | | Third year |
| Pharmacy | | Third year |
| Pharmacy | | Third year |
| Medicine | | Third year |
| Medicine | | Third year |

| | |
|---|---|
| Pharmacy | Fourth year |
| Medicine | Fifth year |
| Medicine | Third year |
| Medicine | Third year |
| Medicine | Second year |
| | |
| Medicine | Fourth year |
| Pharmacy | Third year |
| Medicine | Fourth year |
| | |
| | |
| Pharmacy | Third year |
| Medicine | Fifth year |
| | |
| Medicine | Fifth year |
| Medicine | Fourth year |
| Medicine | Third year |
| Medicine | Fourth year |
| Medicine | Fifth year |
| | |
| | |
| Medicine | Fourth year |
| | |
| | |
| Medicine | Fifth year |
| | |
| | |
| | |
| | |
| Pharmacy | Fourth year |
| | |
| Medicine | Fifth year |
| | |
| | |
| | |
| Pharmacy | Third year |
| Pharmacy | Fourth year |
| | |
| | |
| | |
| | |
| | |
| Medicine | Third year |
| | |
| | |
| Pharmacy | Third year |
| Medicine | Fourth year |

| | | |
|---|---|---|
| | 3 | Fourth year |
| | 3 | Third year |
| Pharmacy | | Second year |
| | 5 | Third year |
| Medicine | | Fifth year |
| Medicine | | Fifth year |
| Medicine | | Fourth year |
| Medicine | | Fifth year |
| | 5 | Third year |
| | 6 | Sixth year |
| | 3 | Fourth year |

| | | |
|---|---|---|
| Pharmacy | | Third year |
| Medicine | | Fourth year |
| | 3 | Fourth year |
| Medicine | | Fourth year |
| | 3 | Second year |
| | 6 | Third year |
| Medicine | | Fourth year |
| | 6 | Third year |
| | 3 | Third year |
| Medicine | | Fourth year |
| | 6 | Second year |
| Medicine | | Third year |
| Pharmacy | | Second year |
| Medicine | | Second year |
| | 3 | Fourth year |
| Medicine | | First year |
| | 6 | Third year |
| Medicine | | Fourth year |
| Pharmacy | | Second year |
| | 6 | First year |
| Medicine | | Fourth year |
| | 3 | Third year |

| | | |
|---|---|---|
| | 3 | Second year |
| Medicine | | Second year |
| Medicine | | Sixth year |
| | 4 | First year |
| | 6 | First year |
| | 6 | First year |
| | 6 | Third year |
| Pharmacy | | First year |
| Pharmacy | | Second year |
| | 3 | First year |
| | 3 | Second year |
| | 6 | Third year |
| Medicine | | First year |
| Medicine | | Fifth year |
| | 3 | Fifth year |
| Medicine | | First year |
| Medicine | | First year |
| | 6 | Second year |
| Medicine | | Fourth year |
| Pharmacy | | First year |
| Medicine | | Third year |
| Pharmacy | | Fifth year |
| | 6 | Fourth year |
| Pharmacy | | Second year |

| | | |
|---|---|---|
| Medicine | | Fourth year |
| | 4 | Second year |
| Medicine | | Fifth year |
| | 3 | First year |
| Pharmacy | | Fourth year |
| Pharmacy | | Fifth year |
| | 3 | Third year |
| | 6 | First year |
| Medicine | | Third year |
| Medicine | | Third year |
| Medicine | | First year |
| Pharmacy | | Third year |
| | 6 | Fourth year |
| Medicine | | Second year |
| Medicine | | Second year |
| | 3 | First year |
| | 6 | Third year |
| Medicine | | Third year |
| | 4 | First year |
| Medicine | | Second year |

| | | |
|---|---|---|
| Medicine | | First year |
| Medicine | | Third year |
| Medicine | | Second year |
| | 6 | Second year |
| | 6 | First year |
| Medicine | | Third year |
| Medicine | | Third year |
| | 6 | Second year |
| | 3 | Fourth year |
| Pharmacy | | Second year |
| | 3 | First year |
| | 3 | First year |
| Medicine | | Second year |
| Medicine | | First year |
| Medicine | | Third year |
| | 6 | First year |
| Medicine | | Fifth year |
| | 6 | First year |
| Medicine | | Third year |
| Medicine | | First year |
| Medicine | | Fourth year |
| Medicine | | Fifth year |
| | 6 | First year |
| Medicine | | Second year |
| | 6 | Fourth year |

| | | |
|---|---|---|
| | 5 | Third year |
| | 5 | Second year |
| | 5 | Fourth year |
| | 6 | Second year |
| | 6 | Fourth year |
| | 3 | Third year |
| Pharmacy | | First year |
| | 5 | Third year |
| | 5 | Fourth year |
| | 5 | Third year |
| | 3 | Third year |
| | 5 | Fourth year |
| Medicine | | Fourth year |
| | 5 | Third year |
| | 3 | Fourth year |
| | 5 | Fourth year |
| | 3 | Third year |
| | 6 | Second year |
| | 6 | Second year |
| Medicine | | Fourth year |
| | 3 | Fourth year |
| Medicine | | Third year |
| Medicine | | Third year |

| | | |
|---|---|---|
| | 6 | Third year |
| | 6 | First year |
| | 3 | Third year |
| | 5 | Fourth year |
| | 6 | Second year |
| | 6 | Fourth year |
| | 3 | Third year |
| Medicine | | Third year |
| | 3 | Fourth year |
| Medicine | | Fourth year |
| | 5 | Third year |
| | 5 | Third year |
| | 5 | Third year |
| Medicine | | Fifth year |
| Medicine | | Fourth year |

| | | |
|---|---|---|
| | 4 | Second year |
| | 6 | Second year |
| | 6 | Fourth year |
| | 6 | |
| Pharmacy | | Fourth year |
| | 4 | Second year |
| | 5 | Fourth year |
| Medicine | | Third year |
| | 5 | Fourth year |
| Medicine | | Second year |
| Pharmacy | | Fourth year |
| | 3 | Fourth year |
| | 5 | Third year |
| Pharmacy | | Second year |
| Medicine | | Fifth year |
| Medicine | | Third year |
| Pharmacy | | Fourth year |
| Medicine | | Fourth year |
| | 6 | Third year |
| Pharmacy | | Fourth year |

| | | |
|---|---|---|
| Medicine | | Fourth year |
| | 5 | Fourth year |

| Q8 Pleasespecify the reason (s) for use? (select all that apply) - Selected Choice |
|---|
| To improve concentration |
| To improve your memory |
| Other, please specify |
| To improve concentration |
| To improve concentration |
| To improve your memory |
| To improve concentration |

To improve academic performance

To improve concentration

To improve concentration

Other, please specify

To improve your memory
To improve your memory
To improve academic performance

To improve concentration
To improve concentration

To improve concentration

To improve concentration
To increase alertness
To improve concentration

To improve concentration
To increase alertness
To increase alertness
To improve concentration
To improve your memory
To improve your memory
To increase alertness
To increase alertness
To improve concentration
To increase alertness

To increase alertness
To improve your memory
To improve concentration
To improve concentration
To increase alertness

To increase alertness
To improve concentration
To improve your memory

To increase alertness
To improve your memory

To improve academic performance
To improve your memory
To improve your memory
To increase alertness
To improve academic performance

To improve academic performance

To improve academic performance

To improve academic performance

To improve academic performance

To increase alertness
To increase alertness

To improve academic performance

To improve concentration
To improve concentration

To improve concentration
To improve academic performance
To increase alertness

To increase alertness
To increase alertness

To increase alertness
To improve concentration

To improve concentration

To improve academic performance

To improve concentration

To improve concentration

To increase alertness

To improve concentration

To increase alertness

To improve concentration

To improve concentration

To increase alertness
Other, please specify

To increase alertness
To improve concentration

Other, please specify

Other, please specify

Other, please specify

To improve concentration

Other, please specify

To improve academic performance

Other, please specify

To improve concentration

To improve concentration

Other, please specify
To improve academic performance

Other, please specify
To increase alertness
To increase alertness
To improve academic performance

To improve concentration
Other, please specify

To improve concentration

Other, please specify

To increase alertness

Other, please specify
To improve academic performance

To increase alertness

Other, please specify

Other, please specify

To improve concentration
To improve academic performance
To improve academic performance

To improve academic performance

To improve academic performance
To improve academic performance
To increase alertness
To improve academic performance
To improve academic performance
To improve academic performance
To improve academic performance
To improve academic performance
To improve academic performance
To improve academic performance

To improve academic performance

To improve academic performance

To improve academic performance

To improve academic performance

To improve academic performance

To improve academic performance

To improve academic performance

To improve academic performance

To improve academic performance
To improve academic performance
To improve academic performance

To increase alertness

To increase alertness
To improve concentration

To improve concentration

To improve concentration

To improve concentration

To increase alertness

To increase alertness

To increase alertness
To improve academic performance

To improve academic performance
To improve academic performance

To improve academic performance
To improve academic performance

To increase alertness

To improve academic performance

To improve concentration
To increase alertness

To improve academic performance
To increase alertness

Q8_5_TEXT
Pleasespecify the reason (s) for use? (select all that apply) - Other, please specify - Text

0582062899

I eat almonds(nuts) for improving memory so it is not a drug. it is food.

None

not used

All of the above

I was trying the vegan diet , the supply of B12 was only applicable through syringes or pills

Gmu is unfair university don’t I will told Allah about them in fair day ,they don’t let me go to dentistry because that m

supplementation

general health benefits

0501383649

2489778169

because it was deficient

Reduce stress

I dont use

0569706766

| Q9 Do you get positive effects from the use of these drugs/ substances (e.g. euphoria, alertness)? - Selected Choice |
| --- |
| Yes. If yes, please specify |
| No |
| Yes. If yes, please specify |
| Yes. If yes, please specify |
| No |
| Yes. If yes, please specify |
| No |
| No |

Yes. If yes, please specify

No

No

No
No

No
Yes. If yes, please specify
No

Yes. If yes, please specify
No

Yes. If yes, please specify

No
Yes. If yes, please specify
Yes. If yes, please specify

No
Yes. If yes, please specify
Yes. If yes, please specify
Yes. If yes, please specify
Yes. If yes, please specify
Yes. If yes, please specify
No
Yes. If yes, please specify
Yes. If yes, please specify
Yes. If yes, please specify

Yes. If yes, please specify
No
Yes. If yes, please specify
Yes. If yes, please specify
Yes. If yes, please specify

Yes. If yes, please specify
Yes. If yes, please specify
Yes. If yes, please specify

Yes. If yes, please specify
Yes. If yes, please specify

Yes. If yes, please specify
Yes. If yes, please specify
Yes. If yes, please specify
Yes. If yes, please specify
Yes. If yes, please specify

Yes. If yes, please specify

Yes. If yes, please specify

No

No

Yes. If yes, please specify
Yes. If yes, please specify

Yes. If yes, please specify

Yes. If yes, please specify
Yes. If yes, please specify

Yes. If yes, please specify
Yes. If yes, please specify
Yes. If yes, please specify

Yes. If yes, please specify
Yes. If yes, please specify

Yes. If yes, please specify
Yes. If yes, please specify

Yes. If yes, please specify

Yes. If yes, please specify

Yes. If yes, please specify

Yes. If yes, please specify

Yes. If yes, please specify

Yes. If yes, please specify

Yes. If yes, please specify

Yes. If yes, please specify

No
Yes. If yes, please specify

No
No

No

Yes. If yes, please specify

Yes. If yes, please specify

No

Yes. If yes, please specify

No

No

No

Yes. If yes, please specify

Yes. If yes, please specify

No

No

Yes. If yes, please specify
No

No
No
Yes. If yes, please specify

No
Yes. If yes, please specify

Yes. If yes, please specify

No

No

Yes. If yes, please specify

No

Yes. If yes, please specify

Yes. If yes, please specify

No

No

Yes. If yes, please specify
Yes. If yes, please specify
Yes. If yes, please specify

No

No

Yes. If yes, please specify
Yes. If yes, please specify
Yes. If yes, please specify
Yes. If yes, please specify
Yes. If yes, please specify
Yes. If yes, please specify
Yes. If yes, please specify
Yes. If yes, please specify
Yes. If yes, please specify
Yes. If yes, please specify
No
No

Yes. If yes, please specify

Yes. If yes, please specify

Yes. If yes, please specify

Yes. If yes, please specify

Yes. If yes, please specify

Yes. If yes, please specify

Yes. If yes, please specify

Yes. If yes, please specify
Yes. If yes, please specify
Yes. If yes, please specify

Yes. If yes, please specify

Yes. If yes, please specify
Yes. If yes, please specify

Yes. If yes, please specify

Yes. If yes, please specify

Yes. If yes, please specify

Yes. If yes, please specify

Yes. If yes, please specify

Yes. If yes, please specify
Yes. If yes, please specify

Yes. If yes, please specify
Yes. If yes, please specify
No

Yes. If yes, please specify
Yes. If yes, please specify

Yes. If yes, please specify

Yes. If yes, please specify

Yes. If yes, please specify
Yes. If yes, please specify

Yes. If yes, please specify
Yes. If yes, please specify

Q9_9_TEXT
Do you get positive effects from the use of these drugs/ substances (e.g. euphoria, alertness)? - Yes. If yes, please specify

Reduces stress and helps to stay focus

none

Increased alertness , concentration

alertness

Alertness
alretness

alretness
alertness
alertness

alertness

alertness

alertness

alertness

alretness

memory

To active

I dont take them

not  used

Study faster and more efficiently and perform better on tests and assignments.

Longer and sharper concentration

alertness

Motivation

good mood, alertness

Increased alertness and focus

48331

Increased focus

Alertness and focus
alertness

| Q10<br>When was the time of yourmost recer | Q11<br>How long did you take the CE for? |
|---|---|
| During exam weeks | Less than 1 month |
| During exam weeks | Less than 1 month |
| During exam weeks | Less than 1 month |
| During exam weeks | 1 year but less than 2 years |
| During studying | Less than 1 month |
| During exam weeks | 1 month but less than 6 months |

| | |
|---|---|
| During studying | 1 month but less than 6 months |
| During exam weeks | Less than 1 month |
| During exam weeks | 1 month but less than 6 months |
| During studying | Less than 1 month |
| During exam weeks | Less than 1 month |
| During exam weeks | Less than 1 month |
| During course work deadlines | Less than 1 month |
| During exam weeks | 1 month but less than 6 months |
| During exam weeks | Less than 1 month |
| During exam weeks | Less than 1 month |
| During exam weeks | Less than 1 month |
| During exam weeks | 1 month but less than 6 months |
| During exam weeks | Less than 1 month |
| During exam weeks | Less than 1 month |
| During exam weeks | Less than 1 month |
| During exam weeks | 1 month but less than 6 months |
| During exam weeks | 1 month but less than 6 months |
| During exam weeks | 1 month but less than 6 months |
| During exam weeks | 1 month but less than 6 months |
| During exam weeks | Less than 1 month |
| During exam weeks | 1 month but less than 6 months |
| During exam weeks | 1 month but less than 6 months |
| During exam weeks | 1 month but less than 6 months |

| | |
|---|---|
| During exam weeks | 1 month but less than 6 months |
| During exam weeks | Less than 1 month |
| During exam weeks | 1 month but less than 6 months |
| During exam weeks | 1 month but less than 6 months |
| During exam weeks | 1 month but less than 6 months |
| During exam weeks | 1 month but less than 6 months |
| During exam weeks | 6 months but less than 1 year |
| During exam weeks | Less than 1 month |
| During exam weeks | 1 month but less than 6 months |
| During exam weeks | 1 month but less than 6 months |
| During exam weeks | 1 month but less than 6 months |
| During exam weeks | 1 month but less than 6 months |
| During exam weeks | 1 month but less than 6 months |
| During exam weeks | 1 month but less than 6 months |
| During exam weeks | 1 month but less than 6 months |
| During exam weeks | 1 month but less than 6 months |
| During exam weeks | 1 month but less than 6 months |
| During exam weeks | 1 month but less than 6 months |
| During exam weeks | Less than 1 month |
| During exam weeks | Less than 1 month |
| During exam weeks | 1 year but less than 2 years |
| During exam weeks | 1 month but less than 6 months |
| During exam weeks | 1 month but less than 6 months |
| During exam weeks | 6 months but less than 1 year |

| | |
|---|---|
| During exam weeks | 6 months but less than 1 year |
| During studying | 1 month but less than 6 months |
| During exam weeks | 1 month but less than 6 months |
| During exam weeks | Less than 1 month |
| During exam weeks | 1 month but less than 6 months |
| During exam weeks | 1 month but less than 6 months |
| During exam weeks | 1 month but less than 6 months |
| | 1 month but less than 6 months |
| During exam weeks | Less than 1 month |
| During exam weeks | Less than 1 month |
| During exam weeks | 1 month but less than 6 months |

| | |
|---|---|
| During exam weeks | 1 month but less than 6 months |
| During exam weeks | Less than 1 month |
| During exam weeks | 1 month but less than 6 months |
| During exam weeks | 1 month but less than 6 months |
| During exam weeks | Less than 1 month |
| During course work deadlines | Less than 1 month |
| Daily basis | 2 years and more |
| During exam weeks | Less than 1 month |

| | |
|---|---|
| During exam weeks | Less than 1 month |
| During course work deadlines | 6 months but less than 1 year |
| Daily basis | Less than 1 month |
| During exam weeks | 1 month but less than 6 months |
| Daily basis | 1 month but less than 6 months |
| Daily basis | Less than 1 month |
| During studying | Less than 1 month |

| | |
|---|---|
| During exam weeks | Less than 1 month |
| During exam weeks | Less than 1 month |
| During exam weeks | Less than 1 month |
| During exam weeks | 1 month but less than 6 months |
| Daily basis | 1 month but less than 6 months |

| | |
|---|---|
| During exam weeks | Less than 1 month |
| During studying | 6 months but less than 1 year |
| During exam weeks | Less than 1 month |
| During exam weeks | Less than 1 month |
| During exam weeks | Less than 1 month |
| During exam weeks | |

| | |
|---|---|
| During exam weeks | 1 month but less than 6 months |
| During exam weeks | 6 months but less than 1 year |
| During exam weeks | 1 month but less than 6 months |
| During exam weeks | 1 month but less than 6 months |
| During exam weeks | 1 month but less than 6 months |
| During exam weeks | 1 month but less than 6 months |
| During exam weeks | 1 month but less than 6 months |
| During exam weeks | 1 month but less than 6 months |
| During exam weeks | 1 month but less than 6 months |
| During exam weeks | 1 month but less than 6 months |
| During exam weeks | 1 month but less than 6 months |
| During exam weeks | 1 month but less than 6 months |
| During exam weeks | 1 month but less than 6 months |
| During exam weeks | 1 month but less than 6 months |
| During exam weeks | 6 months but less than 1 year |

| | |
|---|---|
| During studying | |
| During exam weeks | 1 month but less than 6 months |
| During studying | Less than 1 month |
| During exam weeks | 1 month but less than 6 months |
| During exam weeks | Less than 1 month |
| During exam weeks | Less than 1 month |
| During exam weeks | 1 month but less than 6 months |
| During exam weeks | 1 month but less than 6 months |
| During exam weeks | 1 month but less than 6 months |
| During studying | 1 month but less than 6 months |
| During studying | 6 months but less than 1 year |
| During studying | 6 months but less than 1 year |

| | |
|---|---|
| During exam weeks | 1 month but less than 6 months |
| During exam weeks | Less than 1 month |
| During exam weeks | 6 months but less than 1 year |
| During exam weeks | 1 month but less than 6 months |
| | |
| During studying | 1 month but less than 6 months |
| During exam weeks | 1 month but less than 6 months |
| During studying | 6 months but less than 1 year |
| During exam weeks | 1 month but less than 6 months |
| During exam weeks | 6 months but less than 1 year |
| Daily basis | 2 years and more |
| | |
| During studying | 6 months but less than 1 year |
| During exam weeks | 1 month but less than 6 months |
| During exam weeks | 1 month but less than 6 months |
| During exam weeks | 6 months but less than 1 year |
| During course work deadlines | Less than 1 month |
| During studying | 6 months but less than 1 year |

| | |
|---|---|
| During studying | 1 month but less than 6 months |
| During studying | 1 month but less than 6 months |

| Q12<br>Approximately, how frequentlydid you use t | Q13<br>Where did you obtain thesedrugs/substances? - Sele |
|---|---|
| Once a semester/term | They are prescribed for me |
| Daily | Other, please specify |
| Less than once a semester/term | Other, please specify |
| Weekly | I purchased them online |
| Weekly | Other, please specify |
| Daily | They were given to me by a friend |

| | |
|---|---|
| Daily | I purchased them online |
| Daily | I purchased them online |
| Daily | Other, please specify |
| Once a semester/term | Other, please specify |
| Less than once a semester/term | They are prescribed for me |
| Less than once a semester/term | They were given to me by a friend |
| Once a semester/term | They were prescribed for somebody else |
| Daily | I purchased them online |
| Less than once a semester/term | I purchased them online |
| Less than once a semester/term | I purchased them online |
| Less than once a semester/term | They were given to me by a friend |
| Once a semester/term | I purchased them online |
| Daily | I purchased them online |
| Daily | They were given to me by a friend |
| Daily | I purchased them online |
| Daily | I purchased them online |
| Daily | I purchased them online |
| Daily | I purchased them online |
| Daily | I purchased them online |
| Daily | I purchased them online |
| Daily | I purchased them online |
| Daily | I purchased them online |
| Daily | I purchased them online |

| | |
|---|---|
| Daily | I purchased them online |
| Once a semester/term | I purchased them online |
| Once a semester/term | I purchased them online |
| Daily | I purchased them online |
| Daily | I purchased them online |
| Daily | I purchased them online |
| Daily | I purchased them online |
| Daily | They were given to me by a friend |
| Daily | I purchased them online |
| Once a semester/term | They were given to me by a friend |
| Daily | They were given to me by a friend |
| Daily | I purchased them online |
| Daily | |
| Daily | I purchased them online |
| Daily | I purchased them online |
| Daily | I purchased them online |
| Daily | They were given to me by a friend |
| Weekly | I purchased them online |
| Less than once a semester/term | They were given to me by a friend |
| Once a semester/term | I purchased them online |
| Once a semester/term | I purchased them online |
| Once a semester/term | They were given to me by a friend |
| Once a semester/term | I purchased them online |
| Once a semester/term | I purchased them online |

| | |
|---|---|
| Once a semester/term | I purchased them online |
| Weekly | They were given to me by a friend |
| Monthly | I purchased them online |
| Once a semester/term | I purchased them online |
| Less than once a semester/term | I purchased them online |
| Less than once a semester/term | I purchased them online |
| Once a semester/term | I purchased them online |
| Once a semester/term | I purchased them online |
| Once a semester/term | They were given to me by a friend |
| Monthly | They are prescribed for me |
| Monthly | I purchased them online |

| | |
|---|---|
| Monthly | I purchased them online |
| Once a semester/term | I purchased them online |
| Once a semester/term | I purchased them online |
| Once a semester/term | I purchased them online |
| Once a semester/term | They were given to me by a friend |
| Less than once a semester/term | They were prescribed for somebody else |
| Daily | I purchased them online |
| | Other, please specify |
| Daily | They are prescribed for me |

| | |
|---|---|
| Daily | I purchased them online |
| Weekly | They are prescribed for me |
| Monthly | Other, please specify |
| Less than once a semester/term | I purchased them online |
| Daily | They are prescribed for me |
| Less than once a semester/term | Other, please specify |
| Less than once a semester/term | |

| | |
|---|---|
| Monthly | They are prescribed for me |
| Less than once a semester/term | I purchased them online |
| Daily | Other, please specify |
| Monthly | They are prescribed for me |
| Daily | They are prescribed for me |

| | |
|---|---|
| Once a semester/term | I purchased them online |
| Less than once a semester/term | They are prescribed for me |
| Once a semester/term | I purchased them online |
| Less than once a semester/term | They are prescribed for me |
| Once a semester/term | I purchased them online |
| Once a semester/term | I purchased them online |

| | |
|---|---|
| Once a semester/term | I purchased them online |
| Less than once a semester/term | I purchased them online |
| Less than once a semester/term | I purchased them online |
| Less than once a semester/term | I purchased them online |
| Less than once a semester/term | I purchased them online |
| Once a semester/term | I purchased them online |
| Once a semester/term | I purchased them online |
| Less than once a semester/term | I purchased them online |
| Once a semester/term | They were given to me by a friend |
| Less than once a semester/term | I purchased them online |
| Once a semester/term | I purchased them online |
| Once a semester/term | I purchased them online |
| Once a semester/term | I purchased them online |
| Once a semester/term | I purchased them online |
| Less than once a semester/term | I purchased them online |

| | |
|---|---|
| Once a semester/term | I purchased them online |
| Less than once a semester/term | They were given to me by a friend |
| Less than once a semester/term | I purchased them online |
| Less than once a semester/term | They were given to me by a friend |
| Less than once a semester/term | They were given to me by a friend |
| Less than once a semester/term | I purchased them online |
| Once a semester/term | They were given to me by a friend |
| Once a semester/term | They were given to me by a friend |
| Weekly | I purchased them online |
| Weekly | I purchased them online |
| Weekly | I purchased them online |

| | |
|---|---|
| Once a semester/term | I purchased them online |
| Once a semester/term | They are prescribed for me |
| Once a semester/term | I purchased them online |
| Once a semester/term | They were given to me by a friend |
| Monthly | Other, please specify |
| Once a semester/term | I purchased them online |
| Weekly | Other, please specify |
| Once a semester/term | Other, please specify |
| Monthly | I purchased them online |
| Daily | Other, please specify |
| Weekly | Other, please specify |
| Once a semester/term | They were given to me by a friend |
| Once a semester/term | They were given to me by a friend |
| Once a semester/term | I purchased them online |
| Daily | They were given to me by a friend |
| Monthly | I purchased them online |

| | |
|---|---|
| Monthly | I purchased them online |
| Once a semester/term | I purchased them online |

| Q13_6_TEXT<br>Where did you obtain thesedrugs/substances? - Other, please specify - Text |
|---|
| Over counter |
| Bought it from supplement store |
| i bought them from the pharmacy |

purchased from boots

ASDA, Morrisons, Tesco (it is not a drug) it is just almonds that I use for improving my memory.

not used

Not your business

Does caffeinated drinks count??

I taked from pharmacy or hospital

Pharmacy

Pharmacy

Pharmacy

Over the counter

Pharmacy

| Q14<br>How do you consider thecost of the drugs/ | Q15<br>Do you know anyone who usesthese (dr |
| --- | --- |
| Fair | No |
| | No |
| | No |
| Expensive | Yes |
| | No |
| | Yes |
| | No |
| Expensive | Yes |
| | No |
| Fair | No |
| Expensive | No |
| Fair | Yes |

| | |
|---|---|
| Fair | Yes |
| Fair | Yes |
| Fair | Yes |
| Fair | Yes |
| Fair | No |
| Expensive | Yes |
| Cheap | No |
| Expensive | Yes |
| Expensive | Yes |
| Expensive | No |
| Expensive | Yes |
| Fair | Yes |
| Fair | Yes |
| Expensive | Yes |
| Expensive | No |
| Expensive | No |
| Expensive | Yes |
| Expensive | No |
| Expensive | Yes |
| Expensive | No |
| Expensive | No |
| Expensive | Yes |
| Expensive | Yes |

| | |
|---|---|
| Expensive | No |
| Fair | Yes |
| Fair | Yes |
| Fair | Yes |
| Fair | Yes |
| | |
| Expensive | Yes |
| Expensive | Yes |
| Fair | Yes |
| | |
| | |
| Expensive | No |
| Expensive | Yes |
| | |
| Expensive | Yes |
| Expensive | Yes |
| | |
| Fair | Yes |
| Fair | Yes |
| | |
| | |
| Fair | Yes |
| | |
| | |
| Fair | Yes |
| | |
| | |
| | |
| | |
| Fair | Yes |
| | |
| Expensive | Yes |
| | |
| | |
| | |
| Fair | Yes |
| Expensive | Yes |
| | |
| | |
| | |
| | |
| | |
| Expensive | Yes |
| | |
| | |
| Fair | Yes |
| Expensive | Yes |

| | |
|---|---|
| Fair | Yes |
| Expensive | Yes |
| Fair | Yes |
| Expensive | Yes |
| Expensive | Yes |
| Expensive | Yes |
| Expensive | Yes |
| Expensive | Yes |
| Expensive | Yes |
| Expensive | Yes |
| Fair | Yes |

| | |
|---|---|
| Expensive | Yes |
| Fair | Yes |
| Expensive | Yes |
| Expensive | Yes |
| | No |
| Expensive | No |
| Fair | Yes |
| Expensive | No |
| Fair | Yes |
| | No |
| Very expensive | No |
| | No |

| | |
|---|---|
| Fair | No |
| | No |
| Very expensive | Yes |
| Expensive | No |
| | No |
| Fair | No |
| | No |
| | No |
| Cheap | No |
| | No |
| | No |

| | |
|---|---|
| Fair | Yes |
| | No |
| | Yes |
| Expensive | Yes |
| Fair | No |
| Fair | Yes |
| | No |
| | No |
| | Yes |
| | No |
| Fair | No |

| | |
|---|---|
| Expensive | Yes |
| | No |
| Fair | Yes |
| | No |
| Expensive | Yes |
| Fair | Yes |
| Expensive | Yes |
| Fair | Yes |
| | No |

| | |
|---|---|
| Expensive | Yes |
| Expensive | Yes |
| Expensive | Yes |
| | No |
| Fair | Yes |
| Expensive | Yes |
| Expensive | Yes |
| Expensive | Yes |
| Expensive | Yes |
| Expensive | Yes |
| Expensive | Yes |
| Expensive | Yes |
| Expensive | Yes |
| Expensive | Yes |
| | No |
| | No |
| Expensive | Yes |
| Expensive | Yes |
| | No |

| | |
|---|---|
| Fair | Yes |
| Fair | Yes |
| Fair | Yes |
| Fair | Yes |
| Fair | Yes |
| Fair | Yes |
| Fair | Yes |
| Fair | Yes |
| Fair | Yes |
| Fair | Yes |
| Fair | Yes |

| | |
|---|---|
| | No |
| Fair | Yes |
| Expensive | No |
| Expensive | Yes |
| Fair | Yes |
| Fair | Yes |
| Fair | Yes |
| Expensive | Yes |
| Expensive | Yes |
| Fair | Yes |
| Fair | No |
| Fair | Yes |
| Fair | Yes |
| Expensive | Yes |
| Fair | Yes |
| Fair | Yes |
| Expensive | Yes |

| | |
|---|---|
| Fair | Yes |
| Fair | Yes |

| Q16 | Q17 |
| --- | --- |
| Where have you heard aboutthese dr | Will you recommend these drugs to others? |
| Other | No |
| Other | No |
| Other | |
| | No |
| Scientific literature | Yes |
| Other | Yes |
| Scientific literature | No |
| Social media | No |
| Scientific literature | Yes |
| Family | No |
| | No |
| Social media | No |

| | |
|---|---|
| Internet | No |
| Friends | Yes |
| | No |
| Friends | No |
| Family | No |
| Friends | No |
| Friends | No |
| Scientific literature | Yes |
| Friends | Yes |
| Friends | No |
| Friends | Yes |
| Friends | No |
| Friends | Yes |
| Friends | Yes |
| Friends | No |
| Social media | No |
| Social media | Yes |
| Friends | No |
| Social media | No |
| Friends | No |
| Friends | |
| Friends | No |
| Friends | No |
| Friends | No |

| | |
|---|---|
| Friends | No |
| Friends | No |
| Friends | No |
| Friends | No |
| Social media | No |
| | |
| Friends | No |
| Friends | No |
| Friends | No |
| | |
| | |
| Social media | No |
| Friends | No |
| | |
| Friends | No |
| Friends | No |
| Social media | No |
| Social media | No |
| Friends | No |
| | |
| | |
| Internet | No |
| | |
| | |
| Friends | No |
| | |
| | |
| | |
| | |
| Internet | No |
| | |
| Friends | No |
| | |
| | |
| | |
| Social media | No |
| Social media | No |
| | |
| | |
| | |
| | |
| | |
| Friends | Yes |
| | |
| | |
| Friends | No |
| Friends | Yes |

| | |
|---|---|
| Friends | Yes |
| Friends | Yes |
| Internet | No |
| Internet | Yes |
| Internet | No |
| Friends | No |
| Friends | No |
| Internet | No |
| Friends | No |
| Social media | No |
| Internet | No |

| | |
|---|---|
| Internet | No |
| Internet | No |
| Internet | No |
| Internet | No |
| Internet | No |
| Internet | No |
| Friends | No |
| Internet | No |
| Internet | No |
| | No |
| Other | No |

| | |
|---|---|
| Scientific literature | No |
| Social media | Yes |
| Other | No |
| | No |
| Internet | No |
| Internet | No |
| Other | No |
| Family | Yes |
| | No |
| Scientific literature | No |

| | |
|---|---|
| Scientific literature | Yes |
| Other | No |
| Family | No |
| Internet | No |
| Other | No |
| Family | Yes |
| Scientific literature | No |
| | No |
| Internet | |
| Other | No |

| | |
|---|---|
| Internet | Yes |
| Social media | No |
| Other | Yes |
| | No |
| Friends | No |
| Family | Yes |
| Family | Yes |
| Social media | No |
| Internet | No |
| Scientific literature | |

| | |
|---|---|
| Internet | No |
| Friends | No |
| Friends | Yes |
| | No |
| Friends | No |
| Friends | No |
| Friends | No |
| Internet | Yes |
| Internet | Yes |
| Friends | No |
| Internet | Yes |
| Internet | Yes |
| Internet | No |
| Internet | No |
| Social media | No |
| Social media | No |
| Internet | Yes |
| Internet | No |
| Social media | No |

| | |
|---|---|
| Internet | No |
| Internet | No |
| Internet | No |
| Friends | No |
| Friends | No |
| Internet | Yes |
| Friends | Yes |
| Friends | Yes |
| Internet | Yes |
| Internet | Yes |
| Internet | Yes |

| | |
|---|---|
| Internet | Yes |
| Other | No |
| Friends | Yes |
| Friends | Yes |
| Friends | Yes |
| Friends | Yes |
| Family | Yes |
| Friends | Yes |
| Friends | Yes |
| Scientific literature | No |
| Friends | Yes |
| Friends | Yes |
| Friends | Yes |
| Friends | Yes |
| Scientific literature | Yes |
| Friends | Yes |

| | |
|---|---|
| Friends | Yes |
| Friends | Yes |

| QID52<br>Thank you for participating to the study. - Selected Choice |
| --- |
| (If you want to take part of winning the amazon voucher please leave your email on the text below to be contacted) |
| |
| (If you want to take part of winning the amazon voucher please leave your email on the text below to be contacted) |
| |
| |
| (If you want to take part of winning the amazon voucher please leave your email on the text below to be contacted) |
| (If you want to take part of winning the amazon voucher please leave your email on the text below to be contacted) |
| |
| (If you want to take part of winning the amazon voucher please leave your email on the text below to be contacted) |
| |
| |
| |
| |
| (If you want to take part of winning the amazon voucher please leave your email on the text below to be contacted) |
| (If you want to take part of winning the amazon voucher please leave your email on the text below to be contacted) |
| |
| |
| |
| (If you want to take part of winning the amazon voucher please leave your email on the text below to be contacted) |
| (If you want to take part of winning the amazon voucher please leave your email on the text below to be contacted) |
| |
| (If you want to take part of winning the amazon voucher please leave your email on the text below to be contacted) |
| (If you want to take part of winning the amazon voucher please leave your email on the text below to be contacted) |
| |
| (If you want to take part of winning the amazon voucher please leave your email on the text below to be contacted) |
| (If you want to take part of winning the amazon voucher please leave your email on the text below to be contacted) |
| (If you want to take part of winning the amazon voucher please leave your email on the text below to be contacted) |
| (If you want to take part of winning the amazon voucher please leave your email on the text below to be contacted) |
| (If you want to take part of winning the amazon voucher please leave your email on the text below to be contacted) |
| |
| |
| |
| (If you want to take part of winning the amazon voucher please leave your email on the text below to be contacted) |
| |
| (If you want to take part of winning the amazon voucher please leave your email on the text below to be contacted) |
| |
| |
| |
| |
| (If you want to take part of winning the amazon voucher please leave your email on the text below to be contacted) |
| |
| |
| (If you want to take part of winning the amazon voucher please leave your email on the text below to be contacted) |
| |
| (If you want to take part of winning the amazon voucher please leave your email on the text below to be contacted) |
| (If you want to take part of winning the amazon voucher please leave your email on the text below to be contacted) |

(If you want to take part of winning the amazon voucher please leave your email on the text below to be contacted)

(If you want to take part of winning the amazon voucher please leave your email on the text below to be contacted)

(If you want to take part of winning the amazon voucher please leave your email on the text below to be contacted)

(If you want to take part of winning the amazon voucher please leave your email on the text below to be contacted)

(If you want to take part of winning the amazon voucher please leave your email on the text below to be contacted)

(If you want to take part of winning the amazon voucher please leave your email on the text below to be contacted)

(If you want to take part of winning the amazon voucher please leave your email on the text below to be contacted)

(If you want to take part of winning the amazon voucher please leave your email on the text below to be contacted)

(If you want to take part of winning the amazon voucher please leave your email on the text below to be contacted)

(If you want to take part of winning the amazon voucher please leave your email on the text below to be contacted)

(If you want to take part of winning the amazon voucher please leave your email on the text below to be contacted)

(If you want to take part of winning the amazon voucher please leave your email on the text below to be contacted)

(If you want to take part of winning the amazon voucher please leave your email on the text below to be contacted)

(If you want to take part of winning the amazon voucher please leave your email on the text below to be contacted)

(If you want to take part of winning the amazon voucher please leave your email on the text below to be contacted)

(If you want to take part of winning the amazon voucher please leave your email on the text below to be contacted)

(If you want to take part of winning the amazon voucher please leave your email on the text below to be contacted)

(If you want to take part of winning the amazon voucher please leave your email on the text below to be contacted)

(If you want to take part of winning the amazon voucher please leave your email on the text below to be contacted)

(If you want to take part of winning the amazon voucher please leave your email on the text below to be contacted)

(If you want to take part of winning the amazon voucher please leave your email on the text below to be contacted)
(If you want to take part of winning the amazon voucher please leave your email on the text below to be contacted)
(If you want to take part of winning the amazon voucher please leave your email on the text below to be contacted)
(If you want to take part of winning the amazon voucher please leave your email on the text below to be contacted)
(If you want to take part of winning the amazon voucher please leave your email on the text below to be contacted)
(If you want to take part of winning the amazon voucher please leave your email on the text below to be contacted)

(If you want to take part of winning the amazon voucher please leave your email on the text below to be contacted)
(If you want to take part of winning the amazon voucher please leave your email on the text below to be contacted)
(If you want to take part of winning the amazon voucher please leave your email on the text below to be contacted)
(If you want to take part of winning the amazon voucher please leave your email on the text below to be contacted)
(If you want to take part of winning the amazon voucher please leave your email on the text below to be contacted)

(If you want to take part of winning the amazon voucher please leave your email on the text below to be contacted)
(If you want to take part of winning the amazon voucher please leave your email on the text below to be contacted)

(If you want to take part of winning the amazon voucher please leave your email on the text below to be contacted)

(If you want to take part of winning the amazon voucher please leave your email on the text below to be contacted)
(If you want to take part of winning the amazon voucher please leave your email on the text below to be contacted)

(If you want to take part of winning the amazon voucher please leave your email on the text below to be contacted)

(If you want to take part of winning the amazon voucher please leave your email on the text below to be contacted)

(If you want to take part of winning the amazon voucher please leave your email on the text below to be contacted)

(If you want to take part of winning the amazon voucher please leave your email on the text below to be contacted)
(If you want to take part of winning the amazon voucher please leave your email on the text below to be contacted)
(If you want to take part of winning the amazon voucher please leave your email on the text below to be contacted)

(If you want to take part of winning the amazon voucher please leave your email on the text below to be contacted)
(If you want to take part of winning the amazon voucher please leave your email on the text below to be contacted)

(If you want to take part of winning the amazon voucher please leave your email on the text below to be contacted)
(If you want to take part of winning the amazon voucher please leave your email on the text below to be contacted)

(If you want to take part of winning the amazon voucher please leave your email on the text below to be contacted)

(If you want to take part of winning the amazon voucher please leave your email on the text below to be contacted)
(If you want to take part of winning the amazon voucher please leave your email on the text below to be contacted)

(If you want to take part of winning the amazon voucher please leave your email on the text below to be contacted)

(If you want to take part of winning the amazon voucher please leave your email on the text below to be contacted)

(If you want to take part of winning the amazon voucher please leave your email on the text below to be contacted)
(If you want to take part of winning the amazon voucher please leave your email on the text below to be contacted)

(If you want to take part of winning the amazon voucher please leave your email on the text below to be contacted)
(If you want to take part of winning the amazon voucher please leave your email on the text below to be contacted)

(If you want to take part of winning the amazon voucher please leave your email on the text below to be contacted)

(If you want to take part of winning the amazon voucher please leave your email on the text below to be contacted)
(If you want to take part of winning the amazon voucher please leave your email on the text below to be contacted)
(If you want to take part of winning the amazon voucher please leave your email on the text below to be contacted)
(If you want to take part of winning the amazon voucher please leave your email on the text below to be contacted)
(If you want to take part of winning the amazon voucher please leave your email on the text below to be contacted)
(If you want to take part of winning the amazon voucher please leave your email on the text below to be contacted)

(If you want to take part of winning the amazon voucher please leave your email on the text below to be contacted)
(If you want to take part of winning the amazon voucher please leave your email on the text below to be contacted)
(If you want to take part of winning the amazon voucher please leave your email on the text below to be contacted)
(If you want to take part of winning the amazon voucher please leave your email on the text below to be contacted)
(If you want to take part of winning the amazon voucher please leave your email on the text below to be contacted)

(If you want to take part of winning the amazon voucher please leave your email on the text below to be contacted)

(If you want to take part of winning the amazon voucher please leave your email on the text below to be contacted)

(If you want to take part of winning the amazon voucher please leave your email on the text below to be contacted)
(If you want to take part of winning the amazon voucher please leave your email on the text below to be contacted)
(If you want to take part of winning the amazon voucher please leave your email on the text below to be contacted)
(If you want to take part of winning the amazon voucher please leave your email on the text below to be contacted)
(If you want to take part of winning the amazon voucher please leave your email on the text below to be contacted)

(If you want to take part of winning the amazon voucher please leave your email on the text below to be contacted)

(If you want to take part of winning the amazon voucher please leave your email on the text below to be contacted)

(If you want to take part of winning the amazon voucher please leave your email on the text below to be contacted)
(If you want to take part of winning the amazon voucher please leave your email on the text below to be contacted)

(If you want to take part of winning the amazon voucher please leave your email on the text below to be contacted)

(If you want to take part of winning the amazon voucher please leave your email on the text below to be contacted)

(If you want to take part of winning the amazon voucher please leave your email on the text below to be contacted)
(If you want to take part of winning the amazon voucher please leave your email on the text below to be contacted)

(If you want to take part of winning the amazon voucher please leave your email on the text below to be contacted)

(If you want to take part of winning the amazon voucher please leave your email on the text below to be contacted)

(If you want to take part of winning the amazon voucher please leave your email on the text below to be contacted)
(If you want to take part of winning the amazon voucher please leave your email on the text below to be contacted)

(If you want to take part of winning the amazon voucher please leave your email on the text below to be contacted)
(If you want to take part of winning the amazon voucher please leave your email on the text below to be contacted)
(If you want to take part of winning the amazon voucher please leave your email on the text below to be contacted)

(If you want to take part of winning the amazon voucher please leave your email on the text below to be contacted)
(If you want to take part of winning the amazon voucher please leave your email on the text below to be contacted)

(If you want to take part of winning the amazon voucher please leave your email on the text below to be contacted)

(If you want to take part of winning the amazon voucher please leave your email on the text below to be contacted)
(If you want to take part of winning the amazon voucher please leave your email on the text below to be contacted)
(If you want to take part of winning the amazon voucher please leave your email on the text below to be contacted)
(If you want to take part of winning the amazon voucher please leave your email on the text below to be contacted)
(If you want to take part of winning the amazon voucher please leave your email on the text below to be contacted)
(If you want to take part of winning the amazon voucher please leave your email on the text below to be contacted)
(If you want to take part of winning the amazon voucher please leave your email on the text below to be contacted)

(If you want to take part of winning the amazon voucher please leave your email on the text below to be contacted)

(If you want to take part of winning the amazon voucher please leave your email on the text below to be contacted)
(If you want to take part of winning the amazon voucher please leave your email on the text below to be contacted)

(If you want to take part of winning the amazon voucher please leave your email on the text below to be contacted)
(If you want to take part of winning the amazon voucher please leave your email on the text below to be contacted)
(If you want to take part of winning the amazon voucher please leave your email on the text below to be contacted)

(If you want to take part of winning the amazon voucher please leave your email on the text below to be contacted)
(If you want to take part of winning the amazon voucher please leave your email on the text below to be contacted)

(If you want to take part of winning the amazon voucher please leave your email on the text below to be contacted)

(If you want to take part of winning the amazon voucher please leave your email on the text below to be contacted)

(If you want to take part of winning the amazon voucher please leave your email on the text below to be contacted)

(If you want to take part of winning the amazon voucher please leave your email on the text below to be contacted)
(If you want to take part of winning the amazon voucher please leave your email on the text below to be contacted)

(If you want to take part of winning the amazon voucher please leave your email on the text below to be contacted)

(If you want to take part of winning the amazon voucher please leave your email on the text below to be contacted)
(If you want to take part of winning the amazon voucher please leave your email on the text below to be contacted)

(If you want to take part of winning the amazon voucher please leave your email on the text below to be contacted)

(If you want to take part of winning the amazon voucher please leave your email on the text below to be contacted)
(If you want to take part of winning the amazon voucher please leave your email on the text below to be contacted)

(If you want to take part of winning the amazon voucher please leave your email on the text below to be contacted)
(If you want to take part of winning the amazon voucher please leave your email on the text below to be contacted)

(If you want to take part of winning the amazon voucher please leave your email on the text below to be contacted)

(If you want to take part of winning the amazon voucher please leave your email on the text below to be contacted)
(If you want to take part of winning the amazon voucher please leave your email on the text below to be contacted)

(If you want to take part of winning the amazon voucher please leave your email on the text below to be contacted)

(If you want to take part of winning the amazon voucher please leave your email on the text below to be contacted)

(If you want to take part of winning the amazon voucher please leave your email on the text below to be contacted)
(If you want to take part of winning the amazon voucher please leave your email on the text below to be contacted)

(If you want to take part of winning the amazon voucher please leave your email on the text below to be contacted)
(If you want to take part of winning the amazon voucher please leave your email on the text below to be contacted)

(If you want to take part of winning the amazon voucher please leave your email on the text below to be contacted)

(If you want to take part of winning the amazon voucher please leave your email on the text below to be contacted)
(If you want to take part of winning the amazon voucher please leave your email on the text below to be contacted)

(If you want to take part of winning the amazon voucher please leave your email on the text below to be contacted)

(If you want to take part of winning the amazon voucher please leave your email on the text below to be contacted)

| QID52_1_TEXT |
| --- |
| Thank you for participating to the study. - (If you want to take part of winning the amazon voucher please leave your er |

2017bm12@mygmu.ac.ae
2019pt07
seeniasusan@gmail.com
2017pcs02@mygmu.ac.ae
2019pt07@mygmu.ac.ae
saarahbuddha10@gmail.com

shreyarao935@gmail.com
saarahbuddha10@gmail.com
2017d051@mygmu.ac.ae

Majedalghoul@gmail.com

mabelmm98@gmail.com
vaibhavahuja99@gmail.com

aliraxstar900@gmail.com

2015PH06@MYGMU.AC.AE
Themonumentoflife5@gmail.com

daniyah.humair2200@gmail.com

msh1611@gmail.com

Huda.obaid.92@hotmail.com
khadeeja.hala@hotmail.co.uk
2018d054@gmail.com

fathimarahasal@gmail.com

remona1998@gmail.com
aimsorathia@gmail.com

fathima552000@gmail.com

2015m048@gmu.ac.ae
kfariya94@gmail.com

2019m027@mygmu.ac.ae

2015M041@mygmu.ac.ae

rishda_basheer190@hotmail.com
nishatmahveen32@gmail.com

farahismail4997@gmail.com
Waleed_thafer@outlook.com

Najiaziasa@gmail.com

Nayerak113@gmail.com

asoomah01@gmail.com
mizbah_zuber@yahoo.com
christyvinod99@gmail.com
adhna007@gmail.com

minumeer@gmail.com
athira6589@gmail.com
2015m011@mygmu.ac.ae
amna3024@gmail.com
hifsa_randhawa@hotmail.com.au

Sonia_bader@yahoo.com

nahal_gh1@yahoo.com

sharrol456@gmail.com
2019ph19@mygmu.ac.ae
amiraazraq@gmail.com
f0y0a0a0k@gmail.com
mohammedr811@yahoo.com

sadiq.jafrin@gmail.com

2017ph36@mygmu.ac.ae

2018pcs01@mygmu.ac.ae

Fatimamasood99@hotmail.com

2019pt03@mygmu.ac.ae

amirajunaid01@gmail.com

sundus.asim@me.com

marawe14@gmail.com
burhan5253@gmail.com

2019ns16@mygmu.ac.ae
Aliasbali@hotmail.com
basimw14@gmail.com

examsfor12@gmail.com
Sheikha.ahmed.gd@gmail.com

n-ghanem@hotmail.com

2017m032@mygmu.ac.ae
abizerfiroz@gmail.com
alia.zubair07@gmail.com
shreyadhanak@gmail.com
2017m030@mygmu.ac.ae
mariyamda1@gmail.com
jahmd5594@gmail.com

2018ph08@mygmu.ac.ae

tadiwafaranisi1@gmail.com
Alaaa6806@gmail.com

2016m012@mygmu.ac.ae
alenjoy126@gmail.com
raheesahere@gmail.com

2017pcs17@mygmu.ac.ae

Safa.blossom@yahoo.com

2018pcs28@mygmu.ac.ae

aroojtm@hotmail.com

2017m047@mygmu.ac.ae
masudmohammed999@gmail.com

Hamdaalsabouri@gmail.com
zainababdullah2411@gmail.com

nusrath324@gmail.com

2015ph01@mygmu.ac.ae
fateemandanusa@yahoo.com

zainabashraff2@gmail.com

ajmalsainul@gmail.com

2017m021@mygmu.ac.ae
2017m036@mygmu.ac.ae

Anesthesia983@gmail.com

aymannajafi786@gmail.com

2017m068@mygmu.ac.ae

sannashaji@gmail.com

a.saqaaf@gmail.com

Bamigbade.evie@gmail.com

| StartDate | EndDate | Status | Progress |
|---|---|---|---|
| Start Date | End Date | Response Type | Progress |
| 09/03/2020 10:42 | 09/03/2020 10:44 | Survey Preview | 100 |
| 09/03/2020 10:38 | 09/03/2020 10:57 | Survey Preview | 100 |
| 09/03/2020 10:57 | 09/03/2020 10:59 | Survey Preview | 100 |
| 09/03/2020 11:28 | 09/03/2020 11:29 | Survey Preview | 100 |
| 09/03/2020 12:45 | 09/03/2020 12:48 | Survey Preview | 100 |
| 09/03/2020 13:57 | 09/03/2020 13:59 | Survey Preview | 100 |
| 09/03/2020 14:38 | 09/03/2020 14:40 | Survey Preview | 100 |
| 09/03/2020 15:36 | 09/03/2020 15:37 | Survey Preview | 100 |
| 09/03/2020 18:35 | 09/03/2020 18:36 | Survey Preview | 100 |
| 09/03/2020 21:34 | 09/03/2020 21:34 | IP Address | 100 |
| 10/03/2020 13:21 | 10/03/2020 13:24 | Survey Preview | 100 |
| 10/03/2020 14:01 | 10/03/2020 14:02 | Survey Preview | 100 |
| 11/03/2020 06:51 | 11/03/2020 06:54 | Survey Preview | 100 |
| 11/03/2020 06:59 | 11/03/2020 06:59 | Survey Preview | 100 |
| 11/03/2020 06:59 | 11/03/2020 07:00 | Survey Preview | 100 |
| 11/03/2020 06:59 | 11/03/2020 07:00 | Survey Preview | 100 |
| 11/03/2020 06:57 | 11/03/2020 07:00 | Survey Preview | 100 |
| 11/03/2020 07:00 | 11/03/2020 07:01 | Survey Preview | 100 |
| 11/03/2020 07:02 | 11/03/2020 07:05 | Survey Preview | 100 |
| 11/03/2020 07:07 | 11/03/2020 07:08 | Survey Preview | 100 |
| 11/03/2020 07:07 | 11/03/2020 07:08 | Survey Preview | 100 |
| 11/03/2020 07:08 | 11/03/2020 07:10 | Survey Preview | 100 |
| 11/03/2020 07:09 | 11/03/2020 07:10 | Survey Preview | 100 |
| 11/03/2020 07:08 | 11/03/2020 07:11 | Survey Preview | 100 |
| 11/03/2020 07:08 | 11/03/2020 07:12 | Survey Preview | 100 |
| 11/03/2020 07:10 | 11/03/2020 07:14 | Survey Preview | 100 |
| 11/03/2020 07:16 | 11/03/2020 07:17 | Survey Preview | 100 |
| 11/03/2020 07:10 | 11/03/2020 07:21 | Survey Preview | 100 |
| 11/03/2020 07:25 | 11/03/2020 07:25 | Survey Preview | 100 |
| 12/03/2020 04:44 | 12/03/2020 04:47 | Survey Preview | 100 |
| 12/03/2020 04:45 | 12/03/2020 04:47 | Survey Preview | 100 |
| 15/03/2020 23:32 | 15/03/2020 23:33 | IP Address | 100 |
| 15/03/2020 23:32 | 15/03/2020 23:34 | IP Address | 100 |
| 15/03/2020 23:31 | 15/03/2020 23:45 | IP Address | 100 |
| 15/03/2020 23:55 | 15/03/2020 23:57 | IP Address | 100 |
| 16/03/2020 00:06 | 16/03/2020 00:07 | IP Address | 100 |
| 16/03/2020 00:08 | 16/03/2020 00:10 | IP Address | 100 |
| 16/03/2020 00:24 | 16/03/2020 00:25 | IP Address | 100 |
| 16/03/2020 00:31 | 16/03/2020 00:32 | IP Address | 100 |
| 16/03/2020 00:36 | 16/03/2020 00:40 | IP Address | 100 |
| 16/03/2020 00:44 | 16/03/2020 00:46 | IP Address | 100 |
| 16/03/2020 00:48 | 16/03/2020 00:49 | IP Address | 100 |
| 16/03/2020 00:51 | 16/03/2020 00:51 | IP Address | 100 |
| 16/03/2020 00:59 | 16/03/2020 01:02 | IP Address | 100 |
| 16/03/2020 01:18 | 16/03/2020 01:19 | IP Address | 100 |
| 16/03/2020 01:44 | 16/03/2020 01:45 | IP Address | 100 |
| 16/03/2020 02:06 | 16/03/2020 02:08 | IP Address | 100 |

| | | | |
|---|---|---|---|
| 16/03/2020 02:09 | 16/03/2020 02:10 | IP Address | 100 |
| 16/03/2020 02:11 | 16/03/2020 02:12 | IP Address | 100 |
| 16/03/2020 02:32 | 16/03/2020 02:33 | IP Address | 100 |
| 16/03/2020 03:11 | 16/03/2020 03:16 | IP Address | 100 |
| 16/03/2020 03:21 | 16/03/2020 03:24 | IP Address | 100 |
| 16/03/2020 03:28 | 16/03/2020 03:30 | IP Address | 100 |
| 16/03/2020 03:31 | 16/03/2020 03:32 | IP Address | 100 |
| 16/03/2020 04:24 | 16/03/2020 04:24 | IP Address | 100 |
| 16/03/2020 04:34 | 16/03/2020 04:36 | IP Address | 100 |
| 16/03/2020 04:41 | 16/03/2020 04:42 | IP Address | 100 |
| 16/03/2020 05:26 | 16/03/2020 05:31 | IP Address | 100 |
| 16/03/2020 05:30 | 16/03/2020 05:34 | IP Address | 100 |
| 16/03/2020 05:36 | 16/03/2020 05:37 | IP Address | 100 |
| 16/03/2020 06:21 | 16/03/2020 06:23 | IP Address | 100 |
| 16/03/2020 06:57 | 16/03/2020 07:02 | IP Address | 100 |
| 16/03/2020 07:18 | 16/03/2020 07:19 | IP Address | 100 |
| 16/03/2020 07:44 | 16/03/2020 07:45 | IP Address | 100 |
| 16/03/2020 08:44 | 16/03/2020 08:45 | IP Address | 100 |
| 16/03/2020 09:23 | 16/03/2020 09:26 | IP Address | 100 |
| 16/03/2020 09:44 | 16/03/2020 09:50 | IP Address | 100 |
| 16/03/2020 10:55 | 16/03/2020 10:56 | IP Address | 100 |
| 16/03/2020 11:58 | 16/03/2020 12:00 | IP Address | 100 |
| 16/03/2020 14:27 | 16/03/2020 14:28 | IP Address | 100 |
| 16/03/2020 17:38 | 16/03/2020 17:39 | IP Address | 100 |
| 17/03/2020 01:07 | 17/03/2020 01:09 | IP Address | 100 |
| 17/03/2020 01:55 | 17/03/2020 01:56 | IP Address | 100 |
| 17/03/2020 01:55 | 17/03/2020 01:56 | IP Address | 100 |
| 17/03/2020 01:57 | 17/03/2020 01:57 | IP Address | 100 |
| 17/03/2020 01:55 | 17/03/2020 01:57 | IP Address | 100 |
| 17/03/2020 01:57 | 17/03/2020 01:58 | IP Address | 100 |
| 17/03/2020 02:13 | 17/03/2020 02:13 | IP Address | 100 |
| 17/03/2020 03:01 | 17/03/2020 03:04 | IP Address | 100 |
| 17/03/2020 03:06 | 17/03/2020 03:07 | IP Address | 100 |
| 17/03/2020 03:05 | 17/03/2020 03:07 | IP Address | 100 |
| 17/03/2020 03:43 | 17/03/2020 03:52 | IP Address | 100 |
| 17/03/2020 04:30 | 17/03/2020 04:32 | IP Address | 100 |
| 17/03/2020 04:37 | 17/03/2020 04:38 | IP Address | 100 |
| 17/03/2020 04:48 | 17/03/2020 04:48 | IP Address | 100 |
| 17/03/2020 05:27 | 17/03/2020 05:33 | IP Address | 100 |
| 17/03/2020 05:56 | 17/03/2020 05:57 | IP Address | 100 |
| 17/03/2020 07:09 | 17/03/2020 07:09 | IP Address | 100 |
| 17/03/2020 07:46 | 17/03/2020 07:53 | IP Address | 100 |
| 17/03/2020 09:32 | 17/03/2020 09:34 | IP Address | 100 |
| 17/03/2020 12:58 | 17/03/2020 13:05 | IP Address | 100 |
| 18/03/2020 01:22 | 18/03/2020 01:26 | IP Address | 100 |
| 18/03/2020 10:15 | 18/03/2020 10:19 | IP Address | 100 |
| 19/03/2020 01:25 | 19/03/2020 01:26 | IP Address | 100 |
| 16/03/2020 03:54 | 19/03/2020 09:06 | IP Address | 100 |

| | | | |
|---|---|---|---|
| 19/03/2020 15:47 | 19/03/2020 15:50 | IP Address | 100 |
| 20/03/2020 15:18 | 20/03/2020 15:21 | IP Address | 100 |
| 22/03/2020 03:32 | 22/03/2020 03:33 | IP Address | 100 |
| 22/03/2020 03:41 | 22/03/2020 03:42 | IP Address | 100 |
| 22/03/2020 03:42 | 22/03/2020 03:43 | IP Address | 100 |
| 22/03/2020 03:47 | 22/03/2020 03:52 | IP Address | 100 |
| 22/03/2020 04:05 | 22/03/2020 04:19 | IP Address | 100 |
| 22/03/2020 05:30 | 22/03/2020 05:32 | IP Address | 100 |
| 22/03/2020 06:13 | 22/03/2020 06:14 | IP Address | 100 |
| 22/03/2020 07:11 | 22/03/2020 07:12 | IP Address | 100 |
| 23/03/2020 01:21 | 23/03/2020 01:23 | IP Address | 100 |
| 23/03/2020 09:19 | 23/03/2020 09:20 | IP Address | 100 |
| 20/03/2020 23:31 | 24/03/2020 10:08 | IP Address | 100 |
| 26/03/2020 12:48 | 26/03/2020 12:50 | IP Address | 100 |
| 28/03/2020 03:08 | 28/03/2020 03:08 | Spam | 100 |
| 01/04/2020 16:21 | 01/04/2020 16:21 | Spam | 100 |
| 01/04/2020 16:21 | 01/04/2020 16:21 | Spam | 100 |
| 01/04/2020 17:30 | 01/04/2020 17:31 | IP Address | 100 |
| 04/04/2020 18:00 | 04/04/2020 18:03 | IP Address | 100 |
| 07/04/2020 13:07 | 07/04/2020 13:10 | IP Address | 100 |
| 12/04/2020 08:36 | 12/04/2020 08:36 | IP Address | 100 |
| 12/04/2020 08:38 | 12/04/2020 08:40 | IP Address | 100 |
| 12/04/2020 08:44 | 12/04/2020 08:45 | IP Address | 100 |
| 12/04/2020 08:46 | 12/04/2020 08:47 | IP Address | 100 |
| 12/04/2020 08:47 | 12/04/2020 08:50 | IP Address | 100 |
| 12/04/2020 08:52 | 12/04/2020 08:55 | IP Address | 100 |
| 12/04/2020 08:56 | 12/04/2020 08:57 | IP Address | 100 |
| 12/04/2020 09:00 | 12/04/2020 09:01 | IP Address | 100 |
| 12/04/2020 09:04 | 12/04/2020 09:05 | IP Address | 100 |
| 12/04/2020 09:05 | 12/04/2020 09:07 | IP Address | 100 |
| 12/04/2020 15:15 | 12/04/2020 15:18 | IP Address | 100 |
| 12/04/2020 15:18 | 12/04/2020 15:21 | IP Address | 100 |
| 12/04/2020 15:23 | 12/04/2020 15:24 | IP Address | 100 |
| 12/04/2020 15:24 | 12/04/2020 15:26 | IP Address | 100 |
| 12/04/2020 15:26 | 12/04/2020 15:27 | IP Address | 100 |
| 13/04/2020 08:23 | 13/04/2020 08:24 | IP Address | 100 |
| 13/04/2020 08:24 | 13/04/2020 08:25 | IP Address | 100 |
| 13/04/2020 08:25 | 13/04/2020 08:26 | IP Address | 100 |
| 14/04/2020 07:38 | 14/04/2020 07:39 | IP Address | 100 |
| 14/04/2020 07:40 | 14/04/2020 07:40 | IP Address | 100 |
| 14/04/2020 09:15 | 14/04/2020 09:16 | IP Address | 100 |
| 14/04/2020 09:17 | 14/04/2020 09:17 | IP Address | 100 |
| 14/04/2020 09:17 | 14/04/2020 09:17 | IP Address | 100 |
| 14/04/2020 09:17 | 14/04/2020 09:19 | IP Address | 100 |
| 14/04/2020 09:19 | 14/04/2020 09:20 | IP Address | 100 |
| 14/04/2020 09:30 | 14/04/2020 09:30 | IP Address | 100 |
| 14/04/2020 09:33 | 14/04/2020 09:35 | IP Address | 100 |
| 14/04/2020 12:44 | 14/04/2020 12:45 | IP Address | 100 |

| | | | |
|---|---|---|---|
| 14/04/2020 16:34 | 14/04/2020 16:39 | IP Address | 100 |
| 15/04/2020 07:07 | 15/04/2020 07:07 | IP Address | 100 |
| 15/04/2020 07:34 | 15/04/2020 07:35 | IP Address | 100 |
| 15/04/2020 07:35 | 15/04/2020 07:35 | IP Address | 100 |
| 15/04/2020 07:35 | 15/04/2020 07:35 | IP Address | 100 |
| 15/04/2020 07:35 | 15/04/2020 07:36 | IP Address | 100 |
| 15/04/2020 07:36 | 15/04/2020 07:37 | IP Address | 100 |
| 15/04/2020 07:37 | 15/04/2020 07:37 | IP Address | 100 |
| 15/04/2020 07:40 | 15/04/2020 07:41 | IP Address | 100 |
| 15/04/2020 07:41 | 15/04/2020 07:41 | IP Address | 100 |
| 15/04/2020 07:41 | 15/04/2020 07:42 | IP Address | 100 |
| 15/04/2020 07:42 | 15/04/2020 07:42 | IP Address | 100 |
| 15/04/2020 07:44 | 15/04/2020 07:44 | IP Address | 100 |
| 15/04/2020 07:44 | 15/04/2020 07:45 | IP Address | 100 |
| 15/04/2020 07:45 | 15/04/2020 07:46 | IP Address | 100 |
| 15/04/2020 07:47 | 15/04/2020 07:48 | IP Address | 100 |
| 15/04/2020 07:49 | 15/04/2020 07:49 | IP Address | 100 |
| 15/04/2020 10:08 | 15/04/2020 10:08 | IP Address | 100 |
| 15/04/2020 10:08 | 15/04/2020 10:08 | IP Address | 100 |
| 15/04/2020 18:54 | 15/04/2020 18:55 | IP Address | 100 |
| 16/04/2020 18:27 | 16/04/2020 18:28 | IP Address | 100 |
| 16/04/2020 18:28 | 16/04/2020 18:29 | IP Address | 100 |
| 16/04/2020 18:29 | 16/04/2020 18:29 | IP Address | 100 |
| 16/04/2020 18:29 | 16/04/2020 18:29 | IP Address | 100 |
| 16/04/2020 18:30 | 16/04/2020 18:30 | IP Address | 100 |
| 16/04/2020 18:30 | 16/04/2020 18:30 | IP Address | 100 |
| 16/04/2020 18:31 | 16/04/2020 18:32 | IP Address | 100 |
| 16/04/2020 18:32 | 16/04/2020 18:32 | IP Address | 100 |
| 16/04/2020 23:19 | 16/04/2020 23:20 | IP Address | 100 |
| 21/04/2020 13:20 | 21/04/2020 13:21 | IP Address | 100 |
| 26/04/2020 15:31 | 26/04/2020 15:32 | IP Address | 100 |
| 26/04/2020 15:33 | 26/04/2020 15:33 | IP Address | 100 |
| 26/04/2020 15:36 | 26/04/2020 15:36 | IP Address | 100 |
| 26/04/2020 15:36 | 26/04/2020 15:38 | IP Address | 100 |
| 26/04/2020 15:38 | 26/04/2020 15:41 | IP Address | 100 |
| 26/04/2020 15:41 | 26/04/2020 15:42 | IP Address | 100 |
| 26/04/2020 15:42 | 26/04/2020 15:42 | IP Address | 100 |
| 26/04/2020 17:04 | 26/04/2020 17:05 | IP Address | 100 |
| 26/04/2020 18:42 | 26/04/2020 18:42 | Spam | 100 |
| 26/04/2020 18:42 | 26/04/2020 18:42 | Spam | 100 |
| 26/04/2020 19:01 | 26/04/2020 19:01 | IP Address | 100 |
| 26/04/2020 19:01 | 26/04/2020 19:01 | IP Address | 100 |
| 26/04/2020 19:03 | 26/04/2020 19:03 | IP Address | 100 |
| 26/04/2020 19:36 | 26/04/2020 19:36 | IP Address | 100 |
| 26/04/2020 19:36 | 26/04/2020 19:37 | IP Address | 100 |
| 26/04/2020 19:37 | 26/04/2020 19:37 | IP Address | 100 |
| 26/04/2020 19:37 | 26/04/2020 19:38 | IP Address | 100 |
| 28/04/2020 09:43 | 28/04/2020 09:44 | IP Address | 100 |

| | | | |
|---|---|---|---|
| 28/04/2020 09:44 | 28/04/2020 09:44 | IP Address | 100 |
| 28/04/2020 09:44 | 28/04/2020 09:44 | IP Address | 100 |
| 28/04/2020 09:44 | 28/04/2020 09:44 | IP Address | 100 |
| 28/04/2020 09:45 | 28/04/2020 09:45 | IP Address | 100 |
| 30/04/2020 18:11 | 30/04/2020 18:12 | IP Address | 100 |
| 30/04/2020 18:12 | 30/04/2020 18:12 | IP Address | 100 |
| 30/04/2020 18:13 | 30/04/2020 18:13 | IP Address | 100 |
| 30/04/2020 18:13 | 30/04/2020 18:13 | IP Address | 100 |
| 30/04/2020 18:14 | 30/04/2020 18:14 | IP Address | 100 |
| 30/04/2020 18:14 | 30/04/2020 18:14 | IP Address | 100 |
| 01/05/2020 19:43 | 01/05/2020 19:44 | IP Address | 100 |
| 01/05/2020 19:45 | 01/05/2020 19:45 | IP Address | 100 |
| 01/05/2020 19:45 | 01/05/2020 19:45 | IP Address | 100 |
| 01/05/2020 19:45 | 01/05/2020 19:45 | IP Address | 100 |
| 02/05/2020 06:28 | 02/05/2020 06:29 | IP Address | 100 |
| 02/05/2020 06:29 | 02/05/2020 06:30 | IP Address | 100 |
| 02/05/2020 06:30 | 02/05/2020 06:30 | IP Address | 100 |
| 02/05/2020 06:30 | 02/05/2020 06:30 | IP Address | 100 |
| 02/05/2020 06:31 | 02/05/2020 06:31 | IP Address | 100 |
| 02/05/2020 06:51 | 02/05/2020 06:51 | IP Address | 100 |
| 02/05/2020 08:11 | 02/05/2020 08:17 | IP Address | 100 |
| 02/05/2020 08:28 | 02/05/2020 08:29 | IP Address | 100 |
| 02/05/2020 08:29 | 02/05/2020 08:32 | IP Address | 100 |
| 02/05/2020 08:55 | 02/05/2020 08:55 | IP Address | 100 |
| 05/05/2020 21:06 | 05/05/2020 21:06 | IP Address | 100 |
| 05/05/2020 21:06 | 05/05/2020 21:06 | IP Address | 100 |
| 05/05/2020 21:06 | 05/05/2020 21:06 | IP Address | 100 |
| 05/05/2020 21:06 | 05/05/2020 21:07 | IP Address | 100 |
| 05/05/2020 21:07 | 05/05/2020 21:07 | IP Address | 100 |
| 08/05/2020 18:12 | 08/05/2020 18:13 | IP Address | 100 |
| 08/05/2020 18:13 | 08/05/2020 18:14 | IP Address | 100 |
| 08/05/2020 18:14 | 08/05/2020 18:14 | IP Address | 100 |
| 08/05/2020 18:15 | 08/05/2020 18:16 | IP Address | 100 |
| 08/05/2020 18:16 | 08/05/2020 18:17 | IP Address | 100 |
| 10/05/2020 10:40 | 10/05/2020 10:42 | IP Address | 100 |
| 10/05/2020 10:42 | 10/05/2020 10:42 | IP Address | 100 |
| 10/05/2020 10:42 | 10/05/2020 10:43 | IP Address | 100 |
| 10/05/2020 10:44 | 10/05/2020 10:45 | IP Address | 100 |
| 10/05/2020 10:45 | 10/05/2020 10:46 | IP Address | 100 |
| 12/05/2020 02:34 | 12/05/2020 02:35 | IP Address | 100 |
| 12/05/2020 02:34 | 12/05/2020 02:35 | IP Address | 100 |
| 12/05/2020 02:34 | 12/05/2020 02:35 | IP Address | 100 |
| 12/05/2020 02:34 | 12/05/2020 02:35 | IP Address | 100 |
| 12/05/2020 02:34 | 12/05/2020 02:35 | IP Address | 100 |
| 12/05/2020 02:34 | 12/05/2020 02:35 | IP Address | 100 |
| 12/05/2020 02:35 | 12/05/2020 02:35 | IP Address | 100 |
| 12/05/2020 02:35 | 12/05/2020 02:38 | IP Address | 100 |
| 12/05/2020 02:37 | 12/05/2020 02:38 | IP Address | 100 |

| | | | |
|---|---|---|---|
| 12/05/2020 02:37 | 12/05/2020 02:38 | IP Address | 100 |
| 12/05/2020 02:37 | 12/05/2020 02:38 | IP Address | 100 |
| 12/05/2020 02:38 | 12/05/2020 02:39 | IP Address | 100 |
| 12/05/2020 02:37 | 12/05/2020 02:39 | IP Address | 100 |
| 12/05/2020 02:38 | 12/05/2020 02:39 | IP Address | 100 |
| 12/05/2020 02:34 | 12/05/2020 02:39 | IP Address | 100 |
| 12/05/2020 02:39 | 12/05/2020 02:40 | IP Address | 100 |
| 12/05/2020 02:38 | 12/05/2020 02:41 | IP Address | 100 |
| 12/05/2020 02:41 | 12/05/2020 02:41 | IP Address | 100 |
| 12/05/2020 02:37 | 12/05/2020 02:42 | IP Address | 100 |
| 12/05/2020 02:35 | 12/05/2020 02:43 | IP Address | 100 |
| 12/05/2020 02:42 | 12/05/2020 02:44 | IP Address | 100 |
| 12/05/2020 02:43 | 12/05/2020 02:45 | IP Address | 100 |
| 12/05/2020 02:45 | 12/05/2020 02:46 | IP Address | 100 |
| 12/05/2020 02:41 | 12/05/2020 02:46 | IP Address | 100 |
| 12/05/2020 02:44 | 12/05/2020 02:46 | IP Address | 100 |
| 12/05/2020 02:45 | 12/05/2020 02:46 | IP Address | 100 |
| 12/05/2020 02:45 | 12/05/2020 02:47 | IP Address | 100 |
| 12/05/2020 02:45 | 12/05/2020 02:47 | IP Address | 100 |
| 12/05/2020 02:48 | 12/05/2020 02:49 | IP Address | 100 |
| 12/05/2020 02:44 | 12/05/2020 02:50 | IP Address | 100 |
| 12/05/2020 02:49 | 12/05/2020 02:50 | IP Address | 100 |
| 12/05/2020 02:50 | 12/05/2020 02:51 | IP Address | 100 |
| 12/05/2020 02:53 | 12/05/2020 02:54 | IP Address | 100 |
| 12/05/2020 02:52 | 12/05/2020 02:54 | IP Address | 100 |
| 12/05/2020 02:55 | 12/05/2020 02:57 | IP Address | 100 |
| 12/05/2020 02:56 | 12/05/2020 02:58 | IP Address | 100 |
| 12/05/2020 02:57 | 12/05/2020 02:58 | IP Address | 100 |
| 12/05/2020 02:58 | 12/05/2020 03:00 | IP Address | 100 |
| 12/05/2020 02:59 | 12/05/2020 03:01 | IP Address | 100 |
| 12/05/2020 03:01 | 12/05/2020 03:03 | IP Address | 100 |
| 12/05/2020 03:02 | 12/05/2020 03:03 | IP Address | 100 |
| 12/05/2020 03:02 | 12/05/2020 03:03 | IP Address | 100 |
| 12/05/2020 03:00 | 12/05/2020 03:04 | IP Address | 100 |
| 12/05/2020 03:05 | 12/05/2020 03:06 | IP Address | 100 |
| 12/05/2020 03:03 | 12/05/2020 03:07 | IP Address | 100 |
| 12/05/2020 03:07 | 12/05/2020 03:08 | IP Address | 100 |
| 12/05/2020 03:03 | 12/05/2020 03:10 | IP Address | 100 |
| 12/05/2020 03:09 | 12/05/2020 03:10 | IP Address | 100 |
| 12/05/2020 03:15 | 12/05/2020 03:19 | IP Address | 100 |
| 12/05/2020 03:19 | 12/05/2020 03:20 | IP Address | 100 |
| 12/05/2020 03:20 | 12/05/2020 03:21 | IP Address | 100 |
| 12/05/2020 03:20 | 12/05/2020 03:21 | IP Address | 100 |
| 12/05/2020 03:21 | 12/05/2020 03:23 | IP Address | 100 |
| 12/05/2020 03:26 | 12/05/2020 03:28 | IP Address | 100 |
| 12/05/2020 03:32 | 12/05/2020 03:33 | IP Address | 100 |
| 12/05/2020 03:30 | 12/05/2020 03:33 | IP Address | 100 |
| 12/05/2020 03:35 | 12/05/2020 03:35 | IP Address | 100 |

| | | | |
|---|---|---|---|
| 12/05/2020 03:38 | 12/05/2020 03:39 | IP Address | 100 |
| 12/05/2020 03:40 | 12/05/2020 03:41 | IP Address | 100 |
| 12/05/2020 03:40 | 12/05/2020 03:41 | IP Address | 100 |
| 12/05/2020 03:42 | 12/05/2020 03:43 | IP Address | 100 |
| 12/05/2020 03:37 | 12/05/2020 03:44 | IP Address | 100 |
| 12/05/2020 03:52 | 12/05/2020 03:53 | IP Address | 100 |
| 12/05/2020 03:53 | 12/05/2020 03:54 | IP Address | 100 |
| 12/05/2020 03:54 | 12/05/2020 03:55 | IP Address | 100 |
| 12/05/2020 03:58 | 12/05/2020 04:00 | IP Address | 100 |
| 12/05/2020 04:17 | 12/05/2020 04:19 | IP Address | 100 |
| 12/05/2020 04:20 | 12/05/2020 04:21 | IP Address | 100 |
| 12/05/2020 04:20 | 12/05/2020 04:21 | IP Address | 100 |
| 12/05/2020 04:22 | 12/05/2020 04:22 | IP Address | 100 |
| 12/05/2020 04:24 | 12/05/2020 04:24 | IP Address | 100 |
| 12/05/2020 04:30 | 12/05/2020 04:31 | IP Address | 100 |
| 12/05/2020 04:41 | 12/05/2020 04:43 | IP Address | 100 |
| 12/05/2020 04:42 | 12/05/2020 04:47 | IP Address | 100 |
| 12/05/2020 04:48 | 12/05/2020 04:49 | IP Address | 100 |
| 12/05/2020 04:53 | 12/05/2020 04:54 | IP Address | 100 |
| 12/05/2020 04:54 | 12/05/2020 04:58 | IP Address | 100 |
| 12/05/2020 04:58 | 12/05/2020 05:01 | IP Address | 100 |
| 12/05/2020 05:08 | 12/05/2020 05:09 | IP Address | 100 |
| 12/05/2020 05:18 | 12/05/2020 05:18 | IP Address | 100 |
| 12/05/2020 05:09 | 12/05/2020 05:19 | IP Address | 100 |
| 12/05/2020 05:19 | 12/05/2020 05:20 | IP Address | 100 |
| 12/05/2020 05:19 | 12/05/2020 05:20 | IP Address | 100 |
| 12/05/2020 05:24 | 12/05/2020 05:26 | IP Address | 100 |
| 12/05/2020 05:28 | 12/05/2020 05:30 | IP Address | 100 |
| 12/05/2020 05:32 | 12/05/2020 05:34 | IP Address | 100 |
| 12/05/2020 05:39 | 12/05/2020 05:41 | IP Address | 100 |
| 12/05/2020 05:41 | 12/05/2020 05:42 | IP Address | 100 |
| 12/05/2020 05:44 | 12/05/2020 05:44 | IP Address | 100 |
| 12/05/2020 05:38 | 12/05/2020 05:44 | IP Address | 100 |
| 12/05/2020 05:44 | 12/05/2020 05:48 | IP Address | 100 |
| 12/05/2020 05:56 | 12/05/2020 05:58 | IP Address | 100 |
| 12/05/2020 06:31 | 12/05/2020 06:32 | IP Address | 100 |
| 12/05/2020 06:33 | 12/05/2020 06:34 | IP Address | 100 |
| 12/05/2020 06:32 | 12/05/2020 06:35 | IP Address | 100 |
| 12/05/2020 06:33 | 12/05/2020 06:35 | IP Address | 100 |
| 12/05/2020 06:33 | 12/05/2020 06:36 | IP Address | 100 |
| 12/05/2020 06:45 | 12/05/2020 06:45 | IP Address | 100 |
| 12/05/2020 06:48 | 12/05/2020 06:49 | IP Address | 100 |
| 12/05/2020 06:47 | 12/05/2020 06:49 | IP Address | 100 |
| 12/05/2020 06:49 | 12/05/2020 06:51 | IP Address | 100 |
| 12/05/2020 06:53 | 12/05/2020 06:54 | IP Address | 100 |
| 12/05/2020 06:40 | 12/05/2020 07:05 | IP Address | 100 |
| 12/05/2020 07:05 | 12/05/2020 07:06 | IP Address | 100 |
| 12/05/2020 07:02 | 12/05/2020 07:07 | IP Address | 100 |

| | | | |
|---|---|---|---|
| 12/05/2020 07:02 | 12/05/2020 07:10 | IP Address | 100 |
| 12/05/2020 07:04 | 12/05/2020 07:17 | IP Address | 100 |
| 12/05/2020 07:30 | 12/05/2020 07:32 | IP Address | 100 |
| 12/05/2020 07:34 | 12/05/2020 07:34 | IP Address | 100 |
| 12/05/2020 07:44 | 12/05/2020 07:45 | IP Address | 100 |
| 12/05/2020 07:53 | 12/05/2020 07:54 | IP Address | 100 |
| 12/05/2020 07:54 | 12/05/2020 07:56 | IP Address | 100 |
| 12/05/2020 08:07 | 12/05/2020 08:10 | IP Address | 100 |
| 12/05/2020 08:12 | 12/05/2020 08:14 | IP Address | 100 |
| 12/05/2020 08:31 | 12/05/2020 08:34 | IP Address | 100 |
| 12/05/2020 08:28 | 12/05/2020 08:34 | IP Address | 100 |
| 12/05/2020 09:10 | 12/05/2020 09:12 | IP Address | 100 |
| 12/05/2020 09:15 | 12/05/2020 09:16 | IP Address | 100 |
| 12/05/2020 09:18 | 12/05/2020 09:19 | IP Address | 100 |
| 12/05/2020 09:25 | 12/05/2020 09:26 | IP Address | 100 |
| 12/05/2020 09:27 | 12/05/2020 09:28 | IP Address | 100 |
| 12/05/2020 09:35 | 12/05/2020 09:37 | IP Address | 100 |
| 12/05/2020 09:37 | 12/05/2020 09:37 | IP Address | 100 |
| 12/05/2020 09:38 | 12/05/2020 09:38 | IP Address | 100 |
| 12/05/2020 09:40 | 12/05/2020 09:41 | IP Address | 100 |
| 12/05/2020 10:13 | 12/05/2020 10:14 | IP Address | 100 |
| 12/05/2020 10:59 | 12/05/2020 11:00 | IP Address | 100 |
| 12/05/2020 11:04 | 12/05/2020 11:04 | IP Address | 100 |
| 12/05/2020 11:01 | 12/05/2020 11:05 | IP Address | 100 |
| 12/05/2020 11:07 | 12/05/2020 11:09 | IP Address | 100 |
| 12/05/2020 11:09 | 12/05/2020 11:10 | IP Address | 100 |
| 12/05/2020 11:16 | 12/05/2020 11:19 | IP Address | 100 |
| 12/05/2020 11:33 | 12/05/2020 11:36 | IP Address | 100 |
| 12/05/2020 11:37 | 12/05/2020 11:39 | IP Address | 100 |
| 12/05/2020 11:48 | 12/05/2020 11:50 | IP Address | 100 |
| 12/05/2020 12:03 | 12/05/2020 12:04 | IP Address | 100 |
| 12/05/2020 12:28 | 12/05/2020 12:33 | IP Address | 100 |
| 12/05/2020 12:41 | 12/05/2020 12:43 | IP Address | 100 |
| 12/05/2020 12:45 | 12/05/2020 12:47 | IP Address | 100 |
| 12/05/2020 13:07 | 12/05/2020 13:25 | IP Address | 100 |
| 12/05/2020 13:59 | 12/05/2020 14:00 | IP Address | 100 |
| 12/05/2020 13:59 | 12/05/2020 14:01 | IP Address | 100 |
| 12/05/2020 14:08 | 12/05/2020 14:10 | IP Address | 100 |
| 12/05/2020 14:15 | 12/05/2020 14:18 | IP Address | 100 |
| 12/05/2020 14:41 | 12/05/2020 14:46 | IP Address | 100 |
| 12/05/2020 14:49 | 12/05/2020 14:52 | IP Address | 100 |
| 12/05/2020 15:56 | 12/05/2020 15:58 | IP Address | 100 |
| 12/05/2020 17:00 | 12/05/2020 17:03 | IP Address | 100 |
| 12/05/2020 17:25 | 12/05/2020 17:25 | IP Address | 100 |
| 12/05/2020 17:25 | 12/05/2020 17:26 | IP Address | 100 |
| 12/05/2020 17:27 | 12/05/2020 17:27 | IP Address | 100 |
| 12/05/2020 18:00 | 12/05/2020 18:07 | IP Address | 100 |
| 12/05/2020 18:19 | 12/05/2020 18:21 | IP Address | 100 |

| | | | |
|---|---|---|---|
| 12/05/2020 18:34 | 12/05/2020 18:35 | IP Address | 100 |
| 12/05/2020 19:16 | 12/05/2020 19:19 | IP Address | 100 |
| 12/05/2020 19:36 | 12/05/2020 19:37 | IP Address | 100 |
| 12/05/2020 19:55 | 12/05/2020 19:58 | IP Address | 100 |
| 12/05/2020 20:57 | 12/05/2020 20:59 | IP Address | 100 |
| 12/05/2020 21:05 | 12/05/2020 21:08 | IP Address | 100 |
| 12/05/2020 22:41 | 12/05/2020 22:42 | IP Address | 100 |
| 12/05/2020 22:55 | 12/05/2020 22:56 | IP Address | 100 |
| 12/05/2020 23:01 | 12/05/2020 23:04 | IP Address | 100 |
| 12/05/2020 23:52 | 12/05/2020 23:54 | IP Address | 100 |
| 13/05/2020 00:43 | 13/05/2020 00:45 | IP Address | 100 |
| 13/05/2020 00:46 | 13/05/2020 01:02 | IP Address | 100 |
| 13/05/2020 01:35 | 13/05/2020 01:39 | IP Address | 100 |
| 13/05/2020 01:59 | 13/05/2020 02:03 | IP Address | 100 |
| 13/05/2020 02:20 | 13/05/2020 02:22 | IP Address | 100 |
| 13/05/2020 02:56 | 13/05/2020 02:57 | IP Address | 100 |
| 13/05/2020 02:56 | 13/05/2020 02:58 | IP Address | 100 |
| 13/05/2020 03:16 | 13/05/2020 03:17 | IP Address | 100 |
| 13/05/2020 03:33 | 13/05/2020 03:36 | IP Address | 100 |
| 13/05/2020 03:47 | 13/05/2020 03:48 | IP Address | 100 |
| 13/05/2020 03:57 | 13/05/2020 04:01 | IP Address | 100 |
| 13/05/2020 03:59 | 13/05/2020 04:02 | IP Address | 100 |
| 13/05/2020 04:14 | 13/05/2020 04:15 | IP Address | 100 |
| 13/05/2020 05:27 | 13/05/2020 05:30 | IP Address | 100 |
| 13/05/2020 06:36 | 13/05/2020 06:44 | IP Address | 100 |
| 13/05/2020 07:21 | 13/05/2020 07:26 | IP Address | 100 |
| 13/05/2020 07:53 | 13/05/2020 07:57 | IP Address | 100 |
| 13/05/2020 08:50 | 13/05/2020 08:52 | IP Address | 100 |
| 13/05/2020 09:44 | 13/05/2020 09:46 | IP Address | 100 |
| 13/05/2020 09:49 | 13/05/2020 09:50 | IP Address | 100 |
| 13/05/2020 10:58 | 13/05/2020 10:59 | IP Address | 100 |
| 13/05/2020 11:22 | 13/05/2020 11:23 | IP Address | 100 |
| 13/05/2020 12:06 | 13/05/2020 12:08 | IP Address | 100 |
| 13/05/2020 12:38 | 13/05/2020 12:40 | IP Address | 100 |
| 13/05/2020 14:16 | 13/05/2020 14:17 | IP Address | 100 |
| 13/05/2020 14:36 | 13/05/2020 14:37 | IP Address | 100 |
| 13/05/2020 14:37 | 13/05/2020 14:37 | IP Address | 100 |
| 13/05/2020 14:46 | 13/05/2020 14:46 | IP Address | 100 |
| 13/05/2020 15:07 | 13/05/2020 15:31 | Spam | 100 |
| 13/05/2020 17:20 | 13/05/2020 17:22 | IP Address | 100 |
| 13/05/2020 17:24 | 13/05/2020 17:27 | IP Address | 100 |
| 13/05/2020 17:40 | 13/05/2020 17:42 | IP Address | 100 |
| 13/05/2020 21:00 | 13/05/2020 21:02 | IP Address | 100 |
| 13/05/2020 22:40 | 13/05/2020 22:41 | IP Address | 100 |
| 13/05/2020 22:49 | 13/05/2020 22:50 | IP Address | 100 |
| 14/05/2020 00:31 | 14/05/2020 00:33 | IP Address | 100 |
| 14/05/2020 01:23 | 14/05/2020 01:24 | IP Address | 100 |
| 14/05/2020 01:46 | 14/05/2020 01:48 | IP Address | 100 |

| | | | |
|---|---|---|---|
| 14/05/2020 01:54 | 14/05/2020 01:58 | IP Address | 100 |
| 14/05/2020 02:59 | 14/05/2020 03:01 | IP Address | 100 |
| 14/05/2020 03:56 | 14/05/2020 03:59 | IP Address | 100 |
| 14/05/2020 04:24 | 14/05/2020 04:27 | IP Address | 100 |
| 14/05/2020 05:50 | 14/05/2020 05:50 | IP Address | 100 |
| 14/05/2020 05:49 | 14/05/2020 05:51 | IP Address | 100 |
| 14/05/2020 05:51 | 14/05/2020 05:51 | IP Address | 100 |
| 14/05/2020 06:49 | 14/05/2020 06:49 | IP Address | 100 |
| 14/05/2020 06:49 | 14/05/2020 06:49 | IP Address | 100 |
| 14/05/2020 08:55 | 14/05/2020 08:59 | IP Address | 100 |
| 14/05/2020 08:59 | 14/05/2020 09:01 | IP Address | 100 |
| 14/05/2020 09:06 | 14/05/2020 09:07 | IP Address | 100 |
| 14/05/2020 09:07 | 14/05/2020 09:11 | IP Address | 100 |
| 14/05/2020 09:11 | 14/05/2020 09:12 | IP Address | 100 |
| 14/05/2020 09:13 | 14/05/2020 09:14 | IP Address | 100 |
| 14/05/2020 09:14 | 14/05/2020 09:17 | IP Address | 100 |
| 14/05/2020 09:17 | 14/05/2020 09:20 | IP Address | 100 |
| 14/05/2020 09:20 | 14/05/2020 09:21 | IP Address | 100 |
| 14/05/2020 09:21 | 14/05/2020 09:22 | IP Address | 100 |
| 14/05/2020 13:49 | 14/05/2020 13:53 | IP Address | 100 |
| 14/05/2020 13:53 | 14/05/2020 13:54 | IP Address | 100 |
| 14/05/2020 17:40 | 14/05/2020 17:40 | IP Address | 100 |
| 14/05/2020 17:40 | 14/05/2020 17:41 | IP Address | 100 |
| 14/05/2020 17:41 | 14/05/2020 17:43 | IP Address | 100 |
| 14/05/2020 17:44 | 14/05/2020 17:44 | IP Address | 100 |
| 14/05/2020 17:44 | 14/05/2020 17:45 | IP Address | 100 |
| 14/05/2020 17:45 | 14/05/2020 17:45 | IP Address | 100 |
| 14/05/2020 17:45 | 14/05/2020 17:46 | IP Address | 100 |
| 14/05/2020 17:46 | 14/05/2020 17:46 | IP Address | 100 |
| 15/05/2020 03:45 | 15/05/2020 03:49 | IP Address | 100 |
| 15/05/2020 03:45 | 15/05/2020 03:49 | IP Address | 100 |
| 15/05/2020 06:29 | 15/05/2020 06:30 | IP Address | 100 |
| 15/05/2020 10:51 | 15/05/2020 10:53 | IP Address | 100 |
| 15/05/2020 15:23 | 15/05/2020 15:26 | IP Address | 100 |
| 15/05/2020 19:40 | 15/05/2020 19:41 | IP Address | 100 |
| 15/05/2020 19:42 | 15/05/2020 19:43 | IP Address | 100 |
| 16/05/2020 01:45 | 16/05/2020 01:49 | IP Address | 100 |
| 16/05/2020 15:42 | 16/05/2020 15:43 | IP Address | 100 |
| 16/05/2020 21:08 | 16/05/2020 21:10 | IP Address | 100 |
| 16/05/2020 21:56 | 16/05/2020 21:56 | IP Address | 100 |
| 16/05/2020 21:56 | 16/05/2020 21:58 | IP Address | 100 |
| 17/05/2020 01:45 | 17/05/2020 01:47 | IP Address | 100 |
| 17/05/2020 03:01 | 17/05/2020 03:04 | IP Address | 100 |
| 17/05/2020 15:38 | 17/05/2020 15:40 | IP Address | 100 |
| 17/05/2020 21:32 | 17/05/2020 21:33 | IP Address | 100 |
| 17/05/2020 21:33 | 17/05/2020 21:33 | IP Address | 100 |
| 17/05/2020 21:33 | 17/05/2020 21:33 | IP Address | 100 |
| 17/05/2020 21:33 | 17/05/2020 21:34 | IP Address | 100 |

| | | | |
|---|---|---|---|
| 17/05/2020 21:34 | 17/05/2020 21:35 | IP Address | 100 |
| 18/05/2020 04:40 | 18/05/2020 04:41 | IP Address | 100 |
| 18/05/2020 08:33 | 18/05/2020 08:34 | IP Address | 100 |
| 18/05/2020 11:17 | 18/05/2020 11:17 | IP Address | 100 |
| 18/05/2020 11:17 | 18/05/2020 11:17 | IP Address | 100 |
| 18/05/2020 11:18 | 18/05/2020 11:19 | IP Address | 100 |
| 18/05/2020 19:43 | 18/05/2020 19:43 | Spam | 100 |
| 19/05/2020 06:10 | 19/05/2020 06:12 | IP Address | 100 |
| 19/05/2020 06:39 | 19/05/2020 06:40 | IP Address | 100 |
| 19/05/2020 06:40 | 19/05/2020 06:41 | IP Address | 100 |
| 19/05/2020 06:41 | 19/05/2020 06:41 | IP Address | 100 |
| 19/05/2020 06:41 | 19/05/2020 06:41 | IP Address | 100 |
| 19/05/2020 06:41 | 19/05/2020 06:41 | IP Address | 100 |
| 19/05/2020 06:41 | 19/05/2020 06:42 | IP Address | 100 |
| 19/05/2020 06:42 | 19/05/2020 06:42 | IP Address | 100 |
| 19/05/2020 06:42 | 19/05/2020 06:43 | IP Address | 100 |
| 19/05/2020 06:46 | 19/05/2020 06:46 | IP Address | 100 |
| 19/05/2020 06:46 | 19/05/2020 06:47 | IP Address | 100 |
| 19/05/2020 06:47 | 19/05/2020 06:47 | IP Address | 100 |
| 19/05/2020 06:47 | 19/05/2020 06:48 | IP Address | 100 |
| 19/05/2020 06:48 | 19/05/2020 06:48 | IP Address | 100 |
| 19/05/2020 06:48 | 19/05/2020 06:49 | IP Address | 100 |
| 19/05/2020 06:49 | 19/05/2020 06:50 | IP Address | 100 |
| 19/05/2020 06:50 | 19/05/2020 06:50 | IP Address | 100 |
| 19/05/2020 06:50 | 19/05/2020 06:50 | IP Address | 100 |
| 19/05/2020 06:51 | 19/05/2020 06:51 | IP Address | 100 |
| 19/05/2020 06:51 | 19/05/2020 06:51 | IP Address | 100 |
| 19/05/2020 06:51 | 19/05/2020 06:51 | IP Address | 100 |
| 19/05/2020 06:51 | 19/05/2020 06:51 | IP Address | 100 |
| 19/05/2020 06:52 | 19/05/2020 06:52 | IP Address | 100 |
| 19/05/2020 06:52 | 19/05/2020 06:52 | IP Address | 100 |
| 19/05/2020 06:52 | 19/05/2020 06:52 | IP Address | 100 |
| 19/05/2020 06:53 | 19/05/2020 06:53 | IP Address | 100 |
| 19/05/2020 06:53 | 19/05/2020 06:53 | IP Address | 100 |
| 19/05/2020 06:53 | 19/05/2020 06:53 | IP Address | 100 |
| 19/05/2020 06:53 | 19/05/2020 06:54 | IP Address | 100 |
| 19/05/2020 06:54 | 19/05/2020 06:54 | IP Address | 100 |
| 19/05/2020 06:54 | 19/05/2020 06:54 | IP Address | 100 |
| 19/05/2020 06:54 | 19/05/2020 06:54 | IP Address | 100 |
| 19/05/2020 06:54 | 19/05/2020 06:54 | IP Address | 100 |
| 19/05/2020 06:54 | 19/05/2020 06:55 | IP Address | 100 |
| 21/05/2020 08:49 | 21/05/2020 08:50 | IP Address | 100 |
| 21/05/2020 19:13 | 21/05/2020 19:13 | IP Address | 100 |
| 23/05/2020 05:43 | 23/05/2020 05:43 | IP Address | 100 |
| 27/05/2020 04:27 | 27/05/2020 04:32 | IP Address | 100 |
| 01/06/2020 16:10 | 01/06/2020 16:11 | IP Address | 100 |
| 01/06/2020 20:41 | 01/06/2020 20:42 | IP Address | 100 |
| 03/06/2020 15:59 | 03/06/2020 16:00 | IP Address | 100 |

| | | | |
|---|---|---|---|
| 09/06/2020 16:45 | 09/06/2020 16:46 | IP Address | 100 |
| 10/06/2020 17:08 | 10/06/2020 17:13 | IP Address | 100 |
| 10/06/2020 17:13 | 10/06/2020 17:15 | IP Address | 100 |
| 25/06/2020 00:53 | 25/06/2020 00:55 | IP Address | 100 |
| 15/09/2020 06:08 | 15/09/2020 06:10 | IP Address | 100 |
| 15/09/2020 06:11 | 15/09/2020 06:15 | IP Address | 100 |
| 15/09/2020 06:16 | 15/09/2020 06:17 | IP Address | 100 |
| 15/09/2020 06:18 | 15/09/2020 06:21 | IP Address | 100 |
| 16/09/2020 15:17 | 16/09/2020 15:19 | IP Address | 100 |
| 23/09/2020 08:07 | 23/09/2020 08:08 | IP Address | 100 |
| 23/09/2020 08:08 | 23/09/2020 08:09 | IP Address | 100 |
| 23/09/2020 08:09 | 23/09/2020 08:15 | IP Address | 100 |
| 23/09/2020 08:12 | 23/09/2020 08:15 | IP Address | 100 |
| 23/09/2020 08:17 | 23/09/2020 08:19 | IP Address | 100 |
| 23/09/2020 14:10 | 23/09/2020 14:18 | IP Address | 100 |
| 25/09/2020 04:17 | 25/09/2020 04:19 | IP Address | 100 |
| 25/09/2020 04:19 | 25/09/2020 04:22 | IP Address | 100 |
| 28/09/2020 04:34 | 28/09/2020 04:39 | IP Address | 100 |
| 29/09/2020 17:32 | 29/09/2020 17:37 | IP Address | 100 |
| 30/09/2020 05:02 | 30/09/2020 05:04 | IP Address | 100 |
| 30/09/2020 05:04 | 30/09/2020 05:06 | IP Address | 100 |
| 30/09/2020 05:07 | 30/09/2020 05:09 | IP Address | 100 |
| 30/09/2020 05:11 | 30/09/2020 05:14 | IP Address | 100 |
| 30/09/2020 05:18 | 30/09/2020 05:22 | IP Address | 100 |
| 30/09/2020 05:37 | 30/09/2020 05:39 | IP Address | 100 |
| 30/09/2020 05:40 | 30/09/2020 05:45 | IP Address | 100 |
| 04/10/2020 13:48 | 04/10/2020 13:51 | IP Address | 100 |
| 04/10/2020 13:52 | 04/10/2020 13:57 | IP Address | 100 |
| 09/10/2020 06:39 | 09/10/2020 13:09 | Survey Preview | 100 |
| 20/10/2020 02:38 | 20/10/2020 02:41 | Survey Preview | 100 |
| 05/11/2020 04:29 | 05/11/2020 04:34 | IP Address | 100 |
| 05/11/2020 04:35 | 05/11/2020 04:40 | IP Address | 100 |
| 05/11/2020 04:41 | 05/11/2020 04:46 | IP Address | 100 |

| Duration (in seconds) | Finished | RecordedDate | ResponseId |
| --- | --- | --- | --- |
| Duration (in seconds) | Finished | Recorded Date | Response ID |
| 87 | True | ################ | |
| 1125 | True | ################ | |
| 124 | True | ################ | |
| 40 | True | ################ | |
| 164 | True | ################ | |
| 67 | True | ################ | |
| 75 | True | ################ | |
| 49 | True | ################ | |
| 77 | True | ################ | |
| 3 | True | ################ | |
| 166 | True | ################ | |
| 14 | True | ################ | |
| 206 | True | ################ | |
| 22 | True | ################ | |
| 92 | True | ################ | |
| 107 | True | ################ | |
| 190 | True | ################ | |
| 65 | True | ################ | |
| 170 | True | ################ | |
| 52 | True | ################ | |
| 88 | True | ################ | |
| 123 | True | ################ | |
| 63 | True | ################ | |
| 219 | True | ################ | |
| 230 | True | ################ | |
| 241 | True | ################ | |
| 64 | True | ################ | |
| 654 | True | ################ | |
| 42 | True | ################ | |
| 141 | True | ################ | |
| 132 | True | ################ | |
| 83 | True | ################ | |
| 109 | True | ################ | |
| 797 | True | ################ | |
| 121 | True | ################ | |
| 64 | True | ################ | |
| 107 | True | ################ | |
| 83 | True | ################ | |
| 60 | True | ################ | |
| 239 | True | ################ | |
| 114 | True | ################ | |
| 34 | True | ################ | |
| 32 | True | ################ | |
| 127 | True | ################ | |
| 78 | True | ################ | |
| 80 | True | ################ | |
| 134 | True | ################ | |

| | | |
|---|---|---|
| 83 | True | ################ |
| 45 | True | ################ |
| 94 | True | ################ |
| 300 | True | ################ |
| 157 | True | ################ |
| 106 | True | ################ |
| 85 | True | ################ |
| 44 | True | ################ |
| 107 | True | ################ |
| 85 | True | ################ |
| 300 | True | ################ |
| 187 | True | ################ |
| 43 | True | ################ |
| 134 | True | ################ |
| 314 | True | ################ |
| 38 | True | ################ |
| 78 | True | ################ |
| 50 | True | ################ |
| 205 | True | ################ |
| 366 | True | ################ |
| 71 | True | ################ |
| 101 | True | ################ |
| 45 | True | ################ |
| 63 | True | ################ |
| 139 | True | ################ |
| 45 | True | ################ |
| 55 | True | ################ |
| 18 | True | ################ |
| 103 | True | ################ |
| 69 | True | ################ |
| 35 | True | ################ |
| 172 | True | ################ |
| 38 | True | ################ |
| 121 | True | ################ |
| 582 | True | ################ |
| 103 | True | ################ |
| 61 | True | ################ |
| 36 | True | ################ |
| 314 | True | ################ |
| 71 | True | ################ |
| 47 | True | ################ |
| 401 | True | ################ |
| 98 | True | ################ |
| 383 | True | ################ |
| 221 | True | ################ |
| 254 | True | ################ |
| 64 | True | ################ |
| 277877 | True | ################ |

| | | |
|---|---|---|
| 188 | True | ################ |
| 165 | True | ################ |
| 32 | True | ################ |
| 83 | True | ################ |
| 58 | True | ################ |
| 284 | True | ################ |
| 836 | True | ################ |
| 106 | True | ################ |
| 46 | True | ################ |
| 58 | True | ################ |
| 127 | True | ################ |
| 68 | True | ################ |
| 297445 | True | ################ |
| 123 | True | ################ |
| 4 | True | ################ |
| 7 | True | ################ |
| 5 | True | ################ |
| 101 | True | ################ |
| 218 | True | ################ |
| 122 | True | ################ |
| 38 | True | ################ |
| 90 | True | ################ |
| 106 | True | ################ |
| 71 | True | ################ |
| 179 | True | ################ |
| 194 | True | ################ |
| 62 | True | ################ |
| 54 | True | ################ |
| 62 | True | ################ |
| 88 | True | ################ |
| 166 | True | ################ |
| 141 | True | ################ |
| 81 | True | ################ |
| 100 | True | ################ |
| 61 | True | ################ |
| 70 | True | ################ |
| 54 | True | ################ |
| 25 | True | ################ |
| 75 | True | ################ |
| 49 | True | ################ |
| 78 | True | ################ |
| 27 | True | ################ |
| 11 | True | ################ |
| 88 | True | ################ |
| 62 | True | ################ |
| 11 | True | ################ |
| 88 | True | ################ |
| 51 | True | ################ |

| | | | |
|---|---|---|---|
| 328 | True | ################ | |
| 56 | True | ################ | |
| 63 | True | ################ | |
| 12 | True | ################ | |
| 11 | True | ################ | |
| 50 | True | ################ | |
| 13 | True | ################ | |
| 11 | True | ################ | |
| 56 | True | ################ | |
| 14 | True | ################ | |
| 11 | True | ################ | |
| 10 | True | ################ | |
| 12 | True | ################ | |
| 62 | True | ################ | |
| 12 | True | ################ | |
| 60 | True | ################ | |
| 21 | True | ################ | |
| 4 | True | ################ | |
| 4 | True | ################ | |
| 43 | True | ################ | |
| 79 | True | ################ | |
| 29 | True | ################ | |
| 11 | True | ################ | |
| 15 | True | ################ | |
| 10 | True | ################ | |
| 12 | True | ################ | |
| 93 | True | ################ | |
| 10 | True | ################ | |
| 75 | True | ################ | |
| 75 | True | ################ | |
| 75 | True | ################ | |
| 11 | True | ################ | |
| 11 | True | ################ | |
| 90 | True | ################ | |
| 131 | True | ################ | |
| 50 | True | ################ | |
| 11 | True | ################ | |
| 19 | True | ################ | |
| 4 | True | ################ | |
| 5 | True | ################ | |
| 12 | True | ################ | |
| 12 | True | ################ | |
| 17 | True | ################ | |
| 13 | True | ################ | |
| 11 | True | ################ | |
| 10 | True | ################ | |
| 53 | True | ################ | |
| 56 | True | ################ | |

| | | |
|---|---|---|
| 9 | True | ################ |
| 8 | True | ################ |
| 8 | True | ################ |
| 47 | True | ################ |
| 84 | True | ################ |
| 11 | True | ################ |
| 10 | True | ################ |
| 9 | True | ################ |
| 12 | True | ################ |
| 14 | True | ################ |
| 83 | True | ################ |
| 12 | True | ################ |
| 10 | True | ################ |
| 9 | True | ################ |
| 81 | True | ################ |
| 11 | True | ################ |
| 10 | True | ################ |
| 11 | True | ################ |
| 10 | True | ################ |
| 12 | True | ################ |
| 307 | True | ################ |
| 92 | True | ################ |
| 177 | True | ################ |
| 13 | True | ################ |
| 17 | True | ################ |
| 10 | True | ################ |
| 9 | True | ################ |
| 11 | True | ################ |
| 11 | True | ################ |
| 23 | True | ################ |
| 65 | True | ################ |
| 14 | True | ################ |
| 80 | True | ################ |
| 11 | True | ################ |
| 72 | True | ################ |
| 10 | True | ################ |
| 71 | True | ################ |
| 10 | True | ################ |
| 101 | True | ################ |
| 36 | True | ################ |
| 47 | True | ################ |
| 47 | True | ################ |
| 55 | True | ################ |
| 66 | True | ################ |
| 58 | True | ################ |
| 36 | True | ################ |
| 130 | True | ################ |
| 57 | True | ################ |

| | | | |
|---|---|---|---|
| 74 | True | ################ | |
| 87 | True | ################ | |
| 37 | True | ################ | |
| 127 | True | ################ | |
| 44 | True | ################ | |
| 332 | True | ################ | |
| 68 | True | ################ | |
| 191 | True | ################ | |
| 56 | True | ################ | |
| 324 | True | ################ | |
| 457 | True | ################ | |
| 122 | True | ################ | |
| 155 | True | ################ | |
| 45 | True | ################ | |
| 320 | True | ################ | |
| 168 | True | ################ | |
| 67 | True | ################ | |
| 112 | True | ################ | |
| 99 | True | ################ | |
| 78 | True | ################ | |
| 333 | True | ################ | |
| 72 | True | ################ | |
| 44 | True | ################ | |
| 38 | True | ################ | |
| 148 | True | ################ | |
| 129 | True | ################ | |
| 116 | True | ################ | |
| 35 | True | ################ | |
| 81 | True | ################ | |
| 118 | True | ################ | |
| 90 | True | ################ | |
| 35 | True | ################ | |
| 76 | True | ################ | |
| 249 | True | ################ | |
| 90 | True | ################ | |
| 251 | True | ################ | |
| 49 | True | ################ | |
| 373 | True | ################ | |
| 51 | True | ################ | |
| 207 | True | ################ | |
| 45 | True | ################ | |
| 64 | True | ################ | |
| 62 | True | ################ | |
| 95 | True | ################ | |
| 150 | True | ################ | |
| 80 | True | ################ | |
| 196 | True | ################ | |
| 39 | True | ################ | |

| | | | |
|---|---|---|---|
| 72 | True | ################ | |
| 91 | True | ################ | |
| 82 | True | ################ | |
| 95 | True | ################ | |
| 426 | True | ################ | |
| 38 | True | ################ | |
| 112 | True | ################ | |
| 79 | True | ################ | |
| 71 | True | ################ | |
| 142 | True | ################ | |
| 92 | True | ################ | |
| 100 | True | ################ | |
| 13 | True | ################ | |
| 35 | True | ################ | |
| 86 | True | ################ | |
| 77 | True | ################ | |
| 278 | True | ################ | |
| 90 | True | ################ | |
| 85 | True | ################ | |
| 228 | True | ################ | |
| 194 | True | ################ | |
| 75 | True | ################ | |
| 47 | True | ################ | |
| 582 | True | ################ | |
| 49 | True | ################ | |
| 72 | True | ################ | |
| 170 | True | ################ | |
| 106 | True | ################ | |
| 111 | True | ################ | |
| 75 | True | ################ | |
| 83 | True | ################ | |
| 30 | True | ################ | |
| 387 | True | ################ | |
| 286 | True | ################ | |
| 138 | True | ################ | |
| 47 | True | ################ | |
| 56 | True | ################ | |
| 187 | True | ################ | |
| 118 | True | ################ | |
| 159 | True | ################ | |
| 39 | True | ################ | |
| 88 | True | ################ | |
| 109 | True | ################ | |
| 120 | True | ################ | |
| 84 | True | ################ | |
| 1510 | True | ################ | |
| 99 | True | ################ | |
| 252 | True | ################ | |

| | | |
|---|---|---|
| 458 | True | ################ |
| 813 | True | ################ |
| 142 | True | ################ |
| 39 | True | ################ |
| 99 | True | ################ |
| 97 | True | ################ |
| 154 | True | ################ |
| 190 | True | ################ |
| 110 | True | ################ |
| 126 | True | ################ |
| 379 | True | ################ |
| 77 | True | ################ |
| 64 | True | ################ |
| 76 | True | ################ |
| 68 | True | ################ |
| 88 | True | ################ |
| 141 | True | ################ |
| 37 | True | ################ |
| 29 | True | ################ |
| 35 | True | ################ |
| 77 | True | ################ |
| 42 | True | ################ |
| 47 | True | ################ |
| 213 | True | ################ |
| 99 | True | ################ |
| 46 | True | ################ |
| 192 | True | ################ |
| 180 | True | ################ |
| 84 | True | ################ |
| 92 | True | ################ |
| 77 | True | ################ |
| 265 | True | ################ |
| 74 | True | ################ |
| 73 | True | ################ |
| 1058 | True | ################ |
| 59 | True | ################ |
| 120 | True | ################ |
| 167 | True | ################ |
| 128 | True | ################ |
| 301 | True | ################ |
| 169 | True | ################ |
| 90 | True | ################ |
| 197 | True | ################ |
| 14 | True | ################ |
| 65 | True | ################ |
| 12 | True | ################ |
| 385 | True | ################ |
| 104 | True | ################ |

| | | |
|---|---|---|
| 50 | True | ################ |
| 191 | True | ################ |
| 35 | True | ################ |
| 156 | True | ################ |
| 90 | True | ################ |
| 166 | True | ################ |
| 74 | True | ################ |
| 73 | True | ################ |
| 208 | True | ################ |
| 152 | True | ################ |
| 93 | True | ################ |
| 990 | True | ################ |
| 222 | True | ################ |
| 208 | True | ################ |
| 97 | True | ################ |
| 49 | True | ################ |
| 109 | True | ################ |
| 31 | True | ################ |
| 142 | True | ################ |
| 55 | True | ################ |
| 267 | True | ################ |
| 188 | True | ################ |
| 27 | True | ################ |
| 154 | True | ################ |
| 515 | True | ################ |
| 265 | True | ################ |
| 237 | True | ################ |
| 97 | True | ################ |
| 79 | True | ################ |
| 57 | True | ################ |
| 60 | True | ################ |
| 66 | True | ################ |
| 95 | True | ################ |
| 91 | True | ################ |
| 77 | True | ################ |
| 18 | True | ################ |
| 10 | True | ################ |
| 37 | True | ################ |
| 1422 | True | ################ |
| 111 | True | ################ |
| 163 | True | ################ |
| 65 | True | ################ |
| 117 | True | ################ |
| 72 | True | ################ |
| 71 | True | ################ |
| 137 | True | ################ |
| 113 | True | ################ |
| 101 | True | ################ |

| | | |
|---|---|---|
| 212 | True | ################ |
| 117 | True | ################ |
| 170 | True | ################ |
| 156 | True | ################ |
| 32 | True | ################ |
| 88 | True | ################ |
| 26 | True | ################ |
| 14 | True | ################ |
| 13 | True | ################ |
| 203 | True | ################ |
| 104 | True | ################ |
| 70 | True | ################ |
| 232 | True | ################ |
| 65 | True | ################ |
| 80 | True | ################ |
| 147 | True | ################ |
| 152 | True | ################ |
| 46 | True | ################ |
| 74 | True | ################ |
| 214 | True | ################ |
| 58 | True | ################ |
| 16 | True | ################ |
| 14 | True | ################ |
| 136 | True | ################ |
| 11 | True | ################ |
| 69 | True | ################ |
| 12 | True | ################ |
| 12 | True | ################ |
| 11 | True | ################ |
| 249 | True | ################ |
| 254 | True | ################ |
| 65 | True | ################ |
| 115 | True | ################ |
| 168 | True | ################ |
| 59 | True | ################ |
| 62 | True | ################ |
| 234 | True | ################ |
| 50 | True | ################ |
| 83 | True | ################ |
| 14 | True | ################ |
| 87 | True | ################ |
| 90 | True | ################ |
| 188 | True | ################ |
| 125 | True | ################ |
| 50 | True | ################ |
| 9 | True | ################ |
| 11 | True | ################ |
| 17 | True | ################ |

| | | |
|---|---|---|
| 78 | True | ################ |
| 66 | True | ################ |
| 51 | True | ################ |
| 20 | True | ################ |
| 13 | True | ################ |
| 88 | True | ################ |
| 4 | True | ################ |
| 90 | True | ################ |
| 79 | True | ################ |
| 34 | True | ################ |
| 9 | True | ################ |
| 10 | True | ################ |
| 9 | True | ################ |
| 12 | True | ################ |
| 9 | True | ################ |
| 11 | True | ################ |
| 8 | True | ################ |
| 79 | True | ################ |
| 8 | True | ################ |
| 8 | True | ################ |
| 9 | True | ################ |
| 57 | True | ################ |
| 61 | True | ################ |
| 8 | True | ################ |
| 8 | True | ################ |
| 7 | True | ################ |
| 8 | True | ################ |
| 8 | True | ################ |
| 8 | True | ################ |
| 13 | True | ################ |
| 7 | True | ################ |
| 9 | True | ################ |
| 10 | True | ################ |
| 10 | True | ################ |
| 8 | True | ################ |
| 8 | True | ################ |
| 7 | True | ################ |
| 9 | True | ################ |
| 10 | True | ################ |
| 8 | True | ################ |
| 9 | True | ################ |
| 59 | True | ################ |
| 40 | True | ################ |
| 30 | True | ################ |
| 302 | True | ################ |
| 52 | True | ################ |
| 58 | True | ################ |
| 62 | True | ################ |

| | | |
|---|---|---|
| 81 | True | ################ |
| 276 | True | ################ |
| 99 | True | ################ |
| 102 | True | ################ |
| 122 | True | ################ |
| 251 | True | ################ |
| 100 | True | ################ |
| 195 | True | ################ |
| 111 | True | ################ |
| 76 | True | ################ |
| 95 | True | ################ |
| 362 | True | ################ |
| 211 | True | ################ |
| 118 | True | ################ |
| 513 | True | ################ |
| 84 | True | ################ |
| 180 | True | ################ |
| 329 | True | ################ |
| 274 | True | ################ |
| 139 | True | ################ |
| 84 | True | ################ |
| 113 | True | ################ |
| 181 | True | ################ |
| 260 | True | ################ |
| 144 | True | ################ |
| 251 | True | ################ |
| 198 | True | ################ |
| 264 | True | ################ |
| 23360 | True | ################ |
| 155 | True | ################ |
| 301 | True | ################ |
| 276 | True | ################ |
| 264 | True | ################ |

| DistributionChannel<br>Distribution Channel | UserLanguage<br>User Language | Q1<br>Gender | Q2<br>Age |
|---|---|---|---|
| preview | EN-GB | Female | 18 to 25 |
| preview | EN-GB | Female | 18 to 25 |
| preview | EN-GB | Female | 18 to 25 |
| preview | EN-GB | Female | 18 to 25 |
| preview | EN-GB | Female | 18 to 25 |
| preview | EN-GB | Female | 18 to 25 |
| preview | EN-GB | Female | 18 to 25 |
| preview | EN-GB | Female | 18 to 25 |
| preview | EN-GB | Female | 18 to 25 |
| social | EN-GB | | |
| preview | EN-GB | Male | 18 to 25 |
| preview | EN-GB | | |
| preview | EN-GB | Female | 26 to 35 |
| preview | EN-GB | Male | 18 to 25 |
| preview | EN-GB | Male | 18 to 25 |
| preview | EN-GB | Male | 18 to 25 |
| preview | EN-GB | Female | 18 to 25 |
| preview | EN-GB | Male | 18 to 25 |
| preview | EN-GB | Female | 18 to 25 |
| preview | EN-GB | Female | 36 to 45 |
| preview | EN-GB | Female | 26 to 35 |
| preview | EN-GB | Male | 26 to 35 |
| preview | EN-GB | Male | 26 to 35 |
| preview | EN-GB | Female | 36 to 45 |
| preview | EN-GB | Female | 18 to 25 |
| preview | EN-GB | Male | 18 to 25 |
| preview | EN-GB | Female | 18 to 25 |
| preview | EN-GB | Male | 18 to 25 |
| preview | EN-GB | Female | 18 to 25 |
| preview | EN-GB | Female | 18 to 25 |
| preview | EN-GB | Female | 18 to 25 |
| anonymous | EN-GB | Female | 18 to 25 |
| anonymous | EN-GB | Female | 18 to 25 |
| anonymous | EN-GB | Female | 18 to 25 |
| anonymous | EN-GB | Female | 18 to 25 |
| anonymous | EN-GB | Female | 18 to 25 |
| anonymous | EN-GB | Female | 18 to 25 |
| anonymous | EN-GB | Female | 18 to 25 |
| anonymous | EN-GB | Male | 18 to 25 |
| anonymous | EN-GB | Female | 18 to 25 |
| anonymous | EN-GB | Female | 18 to 25 |
| anonymous | EN-GB | Female | 18 to 25 |
| anonymous | EN-GB | Male | 18 to 25 |
| anonymous | EN-GB | Female | 18 to 25 |
| anonymous | EN-GB | Female | 18 to 25 |
| anonymous | EN-GB | Female | 18 to 25 |
| anonymous | EN-GB | Female | 18 to 25 |

| | | | |
|---|---|---|---|
| anonymous | EN-GB | Female | 18 to 25 |
| anonymous | EN-GB | Female | 18 to 25 |
| anonymous | EN-GB | Male | 18 to 25 |
| anonymous | EN-GB | Female | 18 to 25 |
| anonymous | EN-GB | Female | 36 to 45 |
| anonymous | EN-GB | Female | 18 to 25 |
| anonymous | EN-GB | Female | 18 to 25 |
| anonymous | EN-GB | Female | 18 to 25 |
| anonymous | EN-GB | Female | 18 to 25 |
| anonymous | EN-GB | Female | 18 to 25 |
| anonymous | EN-GB | Male | 18 to 25 |
| anonymous | EN-GB | Female | 18 to 25 |
| anonymous | EN-GB | Female | 18 to 25 |
| anonymous | EN-GB | Prefer not say | 18 to 25 |
| anonymous | EN-GB | Male | 18 to 25 |
| anonymous | EN-GB | Female | 18 to 25 |
| anonymous | EN-GB | Male | 18 to 25 |
| anonymous | EN-GB | Male | 18 to 25 |
| anonymous | EN-GB | Male | 18 to 25 |
| anonymous | EN-GB | Female | 18 to 25 |
| anonymous | EN-GB | Male | 18 to 25 |
| anonymous | EN-GB | Female | 18 to 25 |
| anonymous | EN-GB | Female | 18 to 25 |
| anonymous | EN-GB | Male | 18 to 25 |
| anonymous | EN-GB | Female | 18 to 25 |
| anonymous | EN-GB | Female | 18 to 25 |
| anonymous | EN-GB | Female | 18 to 25 |
| anonymous | EN-GB | Male | 18 to 25 |
| anonymous | EN-GB | Female | 18 to 25 |
| anonymous | EN-GB | Female | 18 to 25 |
| anonymous | EN-GB | Female | 18 to 25 |
| anonymous | EN-GB | Female | 18 to 25 |
| anonymous | EN-GB | Female | 46 to 55 |
| anonymous | EN-GB | Female | 18 to 25 |
| anonymous | EN-GB | Male | 18 to 25 |
| anonymous | EN-GB | Female | 18 to 25 |
| anonymous | EN-GB | Female | 18 to 25 |
| anonymous | EN-GB | Female | 18 to 25 |
| anonymous | EN-GB | Female | 18 to 25 |
| anonymous | EN-GB | Female | 18 to 25 |
| anonymous | EN-GB | Female | 18 to 25 |
| anonymous | EN-GB | Female | 18 to 25 |
| anonymous | EN-GB | Female | 18 to 25 |
| anonymous | EN-GB | Female | 18 to 25 |
| anonymous | EN-GB | Male | 18 to 25 |
| anonymous | EN-GB | Male | 18 to 25 |
| anonymous | EN-GB | Male | 18 to 25 |
| anonymous | EN-GB | Male | 18 to 25 |

| | | | |
|---|---|---|---|
| anonymous | EN-GB | Female | 18 to 25 |
| qr | EN-GB | Male | 18 to 25 |
| anonymous | EN-GB | Male | 18 to 25 |
| anonymous | EN-GB | Female | 18 to 25 |
| anonymous | EN-GB | Female | 18 to 25 |
| anonymous | EN-GB | Male | 18 to 25 |
| anonymous | EN-GB | Female | 18 to 25 |
| anonymous | EN-GB | Female | 18 to 25 |
| anonymous | EN-GB | Female | 18 to 25 |
| anonymous | EN-GB | Male | 18 to 25 |
| anonymous | EN-GB | Female | 18 to 25 |
| anonymous | EN-GB | Female | 18 to 25 |
| anonymous | EN-GB | Female | 18 to 25 |
| social | EN-GB | Male | 18 to 25 |
| anonymous | EN-GB | | |
| social | EN-GB | | |
| anonymous | EN-GB | | |
| anonymous | EN-GB | Male | 18 to 25 |
| social | EN-GB | Male | 18 to 25 |
| anonymous | EN-GB | Male | 18 to 25 |
| anonymous | EN-GB | Female | 18 to 25 |
| anonymous | EN-GB | Female | 18 to 25 |
| anonymous | EN-GB | Male | 18 to 25 |
| anonymous | EN-GB | Male | 18 to 25 |
| anonymous | EN-GB | Male | 18 to 25 |
| anonymous | EN-GB | Male | 18 to 25 |
| anonymous | EN-GB | Male | 18 to 25 |
| anonymous | EN-GB | Male | 18 to 25 |
| anonymous | EN-GB | Male | 18 to 25 |
| anonymous | EN-GB | Male | 18 to 25 |
| anonymous | EN-GB | Female | 18 to 25 |
| anonymous | EN-GB | Female | 18 to 25 |
| anonymous | EN-GB | Male | 18 to 25 |
| anonymous | EN-GB | Male | 18 to 25 |
| anonymous | EN-GB | Male | 18 to 25 |
| anonymous | EN-GB | Male | 18 to 25 |
| anonymous | EN-GB | Male | 18 to 25 |
| anonymous | EN-GB | Female | 18 to 25 |
| anonymous | EN-GB | Male | 18 to 25 |
| anonymous | EN-GB | Male | 18 to 25 |
| anonymous | EN-GB | Male | 18 to 25 |
| anonymous | EN-GB | Male | 18 to 25 |
| anonymous | EN-GB | Male | 18 to 25 |
| anonymous | EN-GB | Male | 18 to 25 |
| anonymous | EN-GB | Male | 18 to 25 |
| anonymous | EN-GB | Male | 18 to 25 |
| anonymous | EN-GB | Male | 18 to 25 |
| social | EN-GB | Male | 18 to 25 |

| | | | |
|---|---|---|---|
| anonymous | EN-GB | Male | 18 to 25 |
| anonymous | EN-GB | Male | 18 to 25 |
| anonymous | EN-GB | Male | 18 to 25 |
| anonymous | EN-GB | Female | 18 to 25 |
| anonymous | EN-GB | Female | 18 to 25 |
| anonymous | EN-GB | Female | 18 to 25 |
| anonymous | EN-GB | Male | 18 to 25 |
| anonymous | EN-GB | Female | 18 to 25 |
| anonymous | EN-GB | Male | 18 to 25 |
| anonymous | EN-GB | Male | 18 to 25 |
| anonymous | EN-GB | Male | 18 to 25 |
| anonymous | EN-GB | Female | 18 to 25 |
| anonymous | EN-GB | Female | 18 to 25 |
| anonymous | EN-GB | Male | 18 to 25 |
| anonymous | EN-GB | Male | 18 to 25 |
| anonymous | EN-GB | Male | 18 to 25 |
| anonymous | EN-GB | Male | 18 to 25 |
| social | EN-GB | | |
| social | EN-GB | | |
| anonymous | EN-GB | Male | |
| anonymous | EN-GB | Male | 18 to 25 |
| anonymous | EN-GB | Female | 18 to 25 |
| anonymous | EN-GB | Male | 18 to 25 |
| anonymous | EN-GB | Female | 18 to 25 |
| anonymous | EN-GB | Male | 18 to 25 |
| anonymous | EN-GB | Female | 18 to 25 |
| anonymous | EN-GB | Male | 18 to 25 |
| anonymous | EN-GB | Male | 18 to 25 |
| anonymous | EN-GB | Male | 18 to 25 |
| anonymous | EN-GB | Male | 18 to 25 |
| anonymous | EN-GB | Male | 18 to 25 |
| anonymous | EN-GB | Female | 18 to 25 |
| anonymous | EN-GB | Female | 18 to 25 |
| anonymous | EN-GB | Female | 18 to 25 |
| anonymous | EN-GB | Male | 18 to 25 |
| anonymous | EN-GB | Male | 18 to 25 |
| anonymous | EN-GB | Female | 18 to 25 |
| social | EN-GB | Female | 26 to 35 |
| social | EN-GB | | |
| social | EN-GB | | |
| social | EN-GB | Female | 18 to 25 |
| social | EN-GB | Female | 18 to 25 |
| social | EN-GB | Male | 26 to 35 |
| social | EN-GB | Male | 18 to 25 |
| social | EN-GB | Female | 18 to 25 |
| social | EN-GB | Male | 18 to 25 |
| social | EN-GB | Male | 18 to 25 |
| anonymous | EN-GB | Male | 18 to 25 |

| | | | |
|---|---|---|---|
| anonymous | EN-GB | Female | 18 to 25 |
| anonymous | EN-GB | Male | 18 to 25 |
| anonymous | EN-GB | Female | 18 to 25 |
| anonymous | EN-GB | Male | 18 to 25 |
| anonymous | EN-GB | Male | 18 to 25 |
| anonymous | EN-GB | Male | 18 to 25 |
| anonymous | EN-GB | Female | 18 to 25 |
| anonymous | EN-GB | Male | 18 to 25 |
| anonymous | EN-GB | Male | 18 to 25 |
| anonymous | EN-GB | Female | 18 to 25 |
| anonymous | EN-GB | Male | 18 to 25 |
| anonymous | EN-GB | Female | 18 to 25 |
| anonymous | EN-GB | Male | 18 to 25 |
| anonymous | EN-GB | Female | 18 to 25 |
| anonymous | EN-GB | Male | 18 to 25 |
| anonymous | EN-GB | Female | 18 to 25 |
| anonymous | EN-GB | Male | 18 to 25 |
| anonymous | EN-GB | Female | 18 to 25 |
| anonymous | EN-GB | Male | 18 to 25 |
| anonymous | EN-GB | Female | 18 to 25 |
| anonymous | EN-GB | Female | |
| anonymous | EN-GB | Female | 26 to 35 |
| anonymous | EN-GB | Male | 46 to 55 |
| anonymous | EN-GB | Male | 18 to 25 |
| anonymous | EN-GB | Male | 18 to 25 |
| anonymous | EN-GB | Male | 18 to 25 |
| anonymous | EN-GB | Female | 18 to 25 |
| anonymous | EN-GB | Female | 18 to 25 |
| anonymous | EN-GB | Male | 18 to 25 |
| anonymous | EN-GB | Female | 18 to 25 |
| anonymous | EN-GB | Female | 18 to 25 |
| anonymous | EN-GB | Male | 18 to 25 |
| anonymous | EN-GB | Male | 18 to 25 |
| anonymous | EN-GB | Male | 18 to 25 |
| anonymous | EN-GB | Female | 18 to 25 |
| anonymous | EN-GB | Female | 18 to 25 |
| anonymous | EN-GB | Male | 18 to 25 |
| anonymous | EN-GB | Male | 18 to 25 |
| anonymous | EN-GB | Male | 18 to 25 |
| anonymous | EN-GB | Female | 18 to 25 |
| anonymous | EN-GB | Female | 18 to 25 |
| anonymous | EN-GB | Male | 18 to 25 |
| anonymous | EN-GB | Female | 18 to 25 |
| anonymous | EN-GB | Female | 18 to 25 |
| anonymous | EN-GB | Female | 18 to 25 |
| anonymous | EN-GB | Female | 18 to 25 |
| anonymous | EN-GB | Female | 18 to 25 |
| anonymous | EN-GB | Male | 18 to 25 |

| | | | |
|---|---|---|---|
| anonymous | EN-GB | Female | 18 to 25 |
| anonymous | EN-GB | Female | 18 to 25 |
| anonymous | EN-GB | Male | 18 to 25 |
| anonymous | EN-GB | Female | 18 to 25 |
| anonymous | EN-GB | Female | 18 to 25 |
| anonymous | EN-GB | Female | 18 to 25 |
| anonymous | EN-GB | Female | 18 to 25 |
| anonymous | EN-GB | Female | 18 to 25 |
| anonymous | EN-GB | Male | 18 to 25 |
| anonymous | EN-GB | Male | 18 to 25 |
| anonymous | EN-GB | Female | 18 to 25 |
| anonymous | EN-GB | Female | 18 to 25 |
| anonymous | EN-GB | Male | 18 to 25 |
| anonymous | EN-GB | Female | 18 to 25 |
| anonymous | EN-GB | Male | 18 to 25 |
| anonymous | EN-GB | Female | 18 to 25 |
| anonymous | EN-GB | Female | 18 to 25 |
| anonymous | EN-GB | Female | 18 to 25 |
| anonymous | EN-GB | Female | 18 to 25 |
| anonymous | EN-GB | Female | 18 to 25 |
| anonymous | EN-GB | Male | 46 to 55 |
| anonymous | EN-GB | Female | 18 to 25 |
| anonymous | EN-GB | Male | 18 to 25 |
| anonymous | EN-GB | Female | 18 to 25 |
| anonymous | EN-GB | Female | 26 to 35 |
| anonymous | EN-GB | Female | 18 to 25 |
| anonymous | EN-GB | Female | 18 to 25 |
| anonymous | EN-GB | Female | 18 to 25 |
| anonymous | EN-GB | Prefer not say | 18 to 25 |
| anonymous | EN-GB | Female | 18 to 25 |
| anonymous | EN-GB | Female | 26 to 35 |
| anonymous | EN-GB | Female | 26 to 35 |
| anonymous | EN-GB | Female | 18 to 25 |
| anonymous | EN-GB | Female | 18 to 25 |
| anonymous | EN-GB | Female | 18 to 25 |
| anonymous | EN-GB | Female | 26 to 35 |
| anonymous | EN-GB | Female | 18 to 25 |
| anonymous | EN-GB | Female | 18 to 25 |
| anonymous | EN-GB | Female | 18 to 25 |
| anonymous | EN-GB | Female | 18 to 25 |
| anonymous | EN-GB | Female | 18 to 25 |
| anonymous | EN-GB | Female | 18 to 25 |
| anonymous | EN-GB | Female | 18 to 25 |
| anonymous | EN-GB | Female | 18 to 25 |
| anonymous | EN-GB | Male | 18 to 25 |
| anonymous | EN-GB | Female | 18 to 25 |
| anonymous | EN-GB | Female | 18 to 25 |
| anonymous | EN-GB | Female | 18 to 25 |

| | | | |
|---|---|---|---|
| anonymous | EN-GB | Female | 26 to 35 |
| anonymous | EN-GB | Female | 18 to 25 |
| anonymous | EN-GB | Female | 18 to 25 |
| anonymous | EN-GB | Female | 18 to 25 |
| anonymous | EN-GB | Female | 18 to 25 |
| anonymous | EN-GB | Female | 26 to 35 |
| anonymous | EN-GB | Male | 18 to 25 |
| anonymous | EN-GB | Female | 18 to 25 |
| anonymous | EN-GB | Female | 18 to 25 |
| anonymous | EN-GB | Female | 18 to 25 |
| anonymous | EN-GB | Female | 18 to 25 |
| anonymous | EN-GB | Male | 18 to 25 |
| anonymous | EN-GB | Female | 18 to 25 |
| anonymous | EN-GB | Female | 18 to 25 |
| anonymous | EN-GB | Female | 18 to 25 |
| anonymous | EN-GB | Female | 18 to 25 |
| anonymous | EN-GB | Female | 18 to 25 |
| anonymous | EN-GB | Female | 18 to 25 |
| anonymous | EN-GB | Female | 18 to 25 |
| anonymous | EN-GB | Female | 18 to 25 |
| anonymous | EN-GB | Male | 18 to 25 |
| anonymous | EN-GB | Female | 18 to 25 |
| anonymous | EN-GB | Female | 18 to 25 |
| anonymous | EN-GB | Female | 18 to 25 |
| anonymous | EN-GB | Female | 18 to 25 |
| anonymous | EN-GB | Female | 18 to 25 |
| anonymous | EN-GB | Female | 18 to 25 |
| anonymous | EN-GB | Female | 18 to 25 |
| anonymous | EN-GB | Female | 18 to 25 |
| anonymous | EN-GB | Male | 18 to 25 |
| anonymous | EN-GB | Female | 18 to 25 |
| anonymous | EN-GB | Female | 18 to 25 |
| anonymous | EN-GB | Female | 18 to 25 |
| anonymous | EN-GB | Female | 26 to 35 |
| anonymous | EN-GB | Male | 18 to 25 |
| anonymous | EN-GB | Female | 18 to 25 |
| anonymous | EN-GB | Female | 18 to 25 |
| anonymous | EN-GB | Female | 18 to 25 |
| anonymous | EN-GB | Female | 18 to 25 |
| anonymous | EN-GB | Female | 18 to 25 |
| anonymous | EN-GB | Male | 18 to 25 |
| anonymous | EN-GB | Female | 18 to 25 |
| anonymous | EN-GB | Female | 18 to 25 |
| anonymous | EN-GB | Female | 18 to 25 |
| anonymous | EN-GB | Female | 18 to 25 |
| anonymous | EN-GB | Female | 18 to 25 |
| anonymous | EN-GB | Male | 18 to 25 |
| anonymous | EN-GB | Female | 18 to 25 |

| | | | |
|---|---|---|---|
| anonymous | EN-GB | Female | 18 to 25 |
| anonymous | EN-GB | Male | 18 to 25 |
| anonymous | EN-GB | Female | 18 to 25 |
| anonymous | EN-GB | Female | 18 to 25 |
| anonymous | EN-GB | Male | 18 to 25 |
| anonymous | EN-GB | Female | 18 to 25 |
| anonymous | EN-GB | Female | 18 to 25 |
| anonymous | EN-GB | Male | 18 to 25 |
| anonymous | EN-GB | Male | 18 to 25 |
| anonymous | EN-GB | Female | 18 to 25 |
| anonymous | EN-GB | Female | 18 to 25 |
| anonymous | EN-GB | Female | 18 to 25 |
| anonymous | EN-GB | Male | 18 to 25 |
| anonymous | EN-GB | Female | 18 to 25 |
| anonymous | EN-GB | Female | 18 to 25 |
| anonymous | EN-GB | Female | 18 to 25 |
| anonymous | EN-GB | Female | 18 to 25 |
| anonymous | EN-GB | Female | 18 to 25 |
| anonymous | EN-GB | Female | 18 to 25 |
| anonymous | EN-GB | Female | 18 to 25 |
| anonymous | EN-GB | Female | 18 to 25 |
| anonymous | EN-GB | Male | 18 to 25 |
| anonymous | EN-GB | Female | 18 to 25 |
| anonymous | EN-GB | Female | 18 to 25 |
| anonymous | EN-GB | Male | 18 to 25 |
| anonymous | EN-GB | Female | 18 to 25 |
| anonymous | EN-GB | Female | 26 to 35 |
| anonymous | EN-GB | Male | 18 to 25 |
| anonymous | EN-GB | Male | 18 to 25 |
| anonymous | EN-GB | Male | 18 to 25 |
| anonymous | EN-GB | Female | 18 to 25 |
| anonymous | EN-GB | Female | 18 to 25 |
| anonymous | EN-GB | Female | 18 to 25 |
| anonymous | EN-GB | Female | 18 to 25 |
| anonymous | EN-GB | Female | 18 to 25 |
| anonymous | EN-GB | Female | 18 to 25 |
| anonymous | EN-GB | Female | 18 to 25 |
| anonymous | EN-GB | Male | 18 to 25 |
| anonymous | EN-GB | Female | 18 to 25 |
| anonymous | EN-GB | Female | 18 to 25 |
| anonymous | EN-GB | Female | 18 to 25 |
| anonymous | EN-GB | Female | 18 to 25 |
| anonymous | EN-GB | Female | 18 to 25 |
| anonymous | EN-GB | Male | 18 to 25 |
| anonymous | EN-GB | Male | 18 to 25 |
| anonymous | EN-GB | Female | 18 to 25 |
| anonymous | EN-GB | Female | 18 to 25 |
| anonymous | EN-GB | Male | 18 to 25 |

| | | | |
|---|---|---|---|
| anonymous | EN-GB | Female | 18 to 25 |
| anonymous | EN-GB | Male | 26 to 35 |
| anonymous | EN-GB | Male | 18 to 25 |
| anonymous | EN-GB | Female | 18 to 25 |
| anonymous | EN-GB | Female | 18 to 25 |
| anonymous | EN-GB | Female | 18 to 25 |
| anonymous | EN-GB | Female | 18 to 25 |
| anonymous | EN-GB | Male | 18 to 25 |
| anonymous | EN-GB | Male | 18 to 25 |
| anonymous | EN-GB | Female | 26 to 35 |
| anonymous | EN-GB | Female | 18 to 25 |
| anonymous | EN-GB | Female | 18 to 25 |
| anonymous | EN-GB | Female | 18 to 25 |
| anonymous | EN-GB | Male | 18 to 25 |
| anonymous | EN-GB | Female | 18 to 25 |
| anonymous | EN-GB | Female | 18 to 25 |
| anonymous | EN-GB | Female | 18 to 25 |
| anonymous | EN-GB | Female | 18 to 25 |
| anonymous | EN-GB | Female | 18 to 25 |
| anonymous | EN-GB | Male | 18 to 25 |
| anonymous | EN-GB | Female | 18 to 25 |
| anonymous | EN-GB | Female | 18 to 25 |
| anonymous | EN-GB | Female | 18 to 25 |
| anonymous | EN-GB | Female | 18 to 25 |
| anonymous | EN-GB | Male | 18 to 25 |
| anonymous | EN-GB | Female | 18 to 25 |
| anonymous | EN-GB | Male | 18 to 25 |
| anonymous | EN-GB | Male | 18 to 25 |
| anonymous | EN-GB | Female | 18 to 25 |
| anonymous | EN-GB | Male | 18 to 25 |
| anonymous | EN-GB | Female | 18 to 25 |
| anonymous | EN-GB | Female | 18 to 25 |
| anonymous | EN-GB | Female | 18 to 25 |
| anonymous | EN-GB | Female | 18 to 25 |
| anonymous | EN-GB | Female | 18 to 25 |
| anonymous | EN-GB | Male | 18 to 25 |
| anonymous | EN-GB | Male | 18 to 25 |
| anonymous | EN-GB | Male | 18 to 25 |
| anonymous | EN-GB | | |
| anonymous | EN-GB | Male | 18 to 25 |
| anonymous | EN-GB | Male | 18 to 25 |
| anonymous | EN-GB | Male | 18 to 25 |
| anonymous | EN-GB | Female | 18 to 25 |
| anonymous | EN-GB | Female | 18 to 25 |
| anonymous | EN-GB | Female | 18 to 25 |
| anonymous | EN-GB | Female | 18 to 25 |
| anonymous | EN-GB | Female | 18 to 25 |
| anonymous | EN-GB | Female | 18 to 25 |

| | | | |
|---|---|---|---|
| anonymous | EN-GB | Female | 18 to 25 |
| anonymous | EN-GB | Female | 18 to 25 |
| anonymous | EN-GB | Female | 18 to 25 |
| anonymous | EN-GB | Female | 18 to 25 |
| anonymous | EN-GB | Male | 18 to 25 |
| anonymous | EN-GB | Female | 18 to 25 |
| anonymous | EN-GB | | |
| anonymous | EN-GB | Male | 18 to 25 |
| anonymous | EN-GB | Male | 18 to 25 |
| anonymous | EN-GB | Male | 18 to 25 |
| anonymous | EN-GB | Male | 18 to 25 |
| anonymous | EN-GB | Male | 18 to 25 |
| anonymous | EN-GB | Male | 18 to 25 |
| anonymous | EN-GB | Male | 18 to 25 |
| anonymous | EN-GB | Male | 18 to 25 |
| anonymous | EN-GB | Male | 18 to 25 |
| anonymous | EN-GB | Male | 18 to 25 |
| anonymous | EN-GB | Male | 18 to 25 |
| anonymous | EN-GB | | 18 to 25 |
| anonymous | EN-GB | Female | 18 to 25 |
| anonymous | EN-GB | Female | 18 to 25 |
| anonymous | EN-GB | Male | 18 to 25 |
| anonymous | EN-GB | Male | 18 to 25 |
| anonymous | EN-GB | Male | 18 to 25 |
| anonymous | EN-GB | Male | 18 to 25 |
| anonymous | EN-GB | Female | 18 to 25 |
| anonymous | EN-GB | Female | 18 to 25 |
| anonymous | EN-GB | Male | 18 to 25 |
| anonymous | EN-GB | Male | 18 to 25 |
| anonymous | EN-GB | Male | 18 to 25 |
| anonymous | EN-GB | Female | 18 to 25 |
| anonymous | EN-GB | Female | 26 to 35 |
| anonymous | EN-GB | Female | 18 to 25 |
| anonymous | EN-GB | Male | 36 to 45 |
| anonymous | EN-GB | Female | 18 to 25 |
| anonymous | EN-GB | Female | 18 to 25 |
| anonymous | EN-GB | Female | 46 to 55 |
| anonymous | EN-GB | Female | 18 to 25 |
| anonymous | EN-GB | Male | 18 to 25 |
| anonymous | EN-GB | Female | 18 to 25 |
| anonymous | EN-GB | Male | 18 to 25 |
| anonymous | EN-GB | Female | 18 to 25 |
| anonymous | EN-GB | Female | 18 to 25 |
| anonymous | EN-GB | Female | 18 to 25 |
| anonymous | EN-GB | Male | 18 to 25 |
| anonymous | EN-GB | Female | 18 to 25 |
| anonymous | EN-GB | Male | 18 to 25 |
| anonymous | EN-GB | Male | 18 to 25 |

| | | | |
|---|---|---|---|
| anonymous | EN-GB | Female | 18 to 25 |
| anonymous | EN-GB | Male | 18 to 25 |
| anonymous | EN-GB | Male | 18 to 25 |
| anonymous | EN-GB | Female | 18 to 25 |
| anonymous | EN-GB | Male | 18 to 25 |
| anonymous | EN-GB | Female | 18 to 25 |
| anonymous | EN-GB | | |
| anonymous | EN-GB | Male | 18 to 25 |
| anonymous | EN-GB | Male | 18 to 25 |
| anonymous | EN-GB | Male | 18 to 25 |
| anonymous | EN-GB | Female | 18 to 25 |
| anonymous | EN-GB | Female | 18 to 25 |
| anonymous | EN-GB | Female | 18 to 25 |
| anonymous | EN-GB | Female | 18 to 25 |
| anonymous | EN-GB | Female | 18 to 25 |
| anonymous | EN-GB | Female | 18 to 25 |
| anonymous | EN-GB | Female | 18 to 25 |
| anonymous | EN-GB | Male | 18 to 25 |
| anonymous | EN-GB | Female | 18 to 25 |
| anonymous | EN-GB | Female | 18 to 25 |
| anonymous | EN-GB | Female | 18 to 25 |
| anonymous | EN-GB | Male | 18 to 25 |
| anonymous | EN-GB | Female | 18 to 25 |
| anonymous | EN-GB | Female | 18 to 25 |
| anonymous | EN-GB | Male | 18 to 25 |
| anonymous | EN-GB | Male | 18 to 25 |
| anonymous | EN-GB | Female | 18 to 25 |
| anonymous | EN-GB | Female | 18 to 25 |
| anonymous | EN-GB | Male | 18 to 25 |
| anonymous | EN-GB | Male | 18 to 25 |
| anonymous | EN-GB | Male | 18 to 25 |
| anonymous | EN-GB | Male | 18 to 25 |
| anonymous | EN-GB | Male | 18 to 25 |
| anonymous | EN-GB | Male | 18 to 25 |
| anonymous | EN-GB | Male | 18 to 25 |
| anonymous | EN-GB | Male | 18 to 25 |
| anonymous | EN-GB | Male | 18 to 25 |
| anonymous | EN-GB | Male | 18 to 25 |
| anonymous | EN-GB | Male | 18 to 25 |
| anonymous | EN-GB | Male | 18 to 25 |
| anonymous | EN-GB | Male | 18 to 25 |
| anonymous | EN-GB | Female | 18 to 25 |
| anonymous | EN-GB | Male | 18 to 25 |
| anonymous | EN-GB | Female | 18 to 25 |
| anonymous | EN-GB | Female | 46 to 55 |
| anonymous | EN-GB | Female | 18 to 25 |
| anonymous | EN-GB | Female | 18 to 25 |
| anonymous | EN-GB | Female | 18 to 25 |

| | | | |
|---|---|---|---|
| anonymous | EN-GB | Female | 18 to 25 |
| anonymous | EN-GB | Male | 18 to 25 |
| anonymous | EN-GB | Female | 18 to 25 |
| anonymous | EN-GB | Female | 36 to 45 |
| anonymous | EN-GB | Female | 18 to 25 |
| anonymous | EN-GB | Male | 18 to 25 |
| anonymous | EN-GB | Female | 18 to 25 |
| anonymous | EN-GB | Female | 18 to 25 |
| anonymous | EN-GB | Female | 18 to 25 |
| anonymous | EN-GB | Female | 18 to 25 |
| anonymous | EN-GB | Male | 18 to 25 |
| anonymous | EN-GB | Male | 18 to 25 |
| anonymous | EN-GB | Male | 18 to 25 |
| anonymous | EN-GB | Male | 18 to 25 |
| anonymous | EN-GB | Male | 18 to 25 |
| anonymous | EN-GB | Male | 18 to 25 |
| anonymous | EN-GB | Female | 18 to 25 |
| anonymous | EN-GB | Male | 18 to 25 |
| anonymous | EN-GB | Female | 18 to 25 |
| anonymous | EN-GB | Female | 18 to 25 |
| anonymous | EN-GB | Male | 18 to 25 |
| anonymous | EN-GB | Female | 18 to 25 |
| anonymous | EN-GB | Female | 18 to 25 |
| anonymous | EN-GB | Male | 18 to 25 |
| anonymous | EN-GB | Male | 18 to 25 |
| anonymous | EN-GB | Male | 18 to 25 |
| anonymous | EN-GB | Male | 18 to 25 |
| anonymous | EN-GB | Female | 18 to 25 |
| preview | EN-GB | Male | 18 to 25 |
| preview | EN-GB | Female | 26 to 35 |
| anonymous | EN-GB | Male | 18 to 25 |
| anonymous | EN-GB | Male | 18 to 25 |
| anonymous | EN-GB | Female | 18 to 25 |

| Q3 |
| --- |
| Have you ever usedpsychostimulants drugs/ nootropics for memory/ concentration/ learning abilit |
| No (Thank you for participating and ‘your survey is completed') |
| No (Thank you for participating and ‘your survey is completed') |
| No (Thank you for participating and ‘your survey is completed') |
| No (Thank you for participating and ‘your survey is completed') |
| No (Thank you for participating and ‘your survey is completed') |
| No (Thank you for participating and ‘your survey is completed') |
| No (Thank you for participating and ‘your survey is completed') |
| No (Thank you for participating and ‘your survey is completed') |
| No (Thank you for participating and ‘your survey is completed') |
| |
| Yes |
| |
| Yes |
| No (Thank you for participating and ‘your survey is completed') |
| No (Thank you for participating and ‘your survey is completed') |
| No (Thank you for participating and ‘your survey is completed') |
| No (Thank you for participating and ‘your survey is completed') |
| No (Thank you for participating and ‘your survey is completed') |
| Yes |
| No (Thank you for participating and ‘your survey is completed') |
| No (Thank you for participating and ‘your survey is completed') |
| No (Thank you for participating and ‘your survey is completed') |
| No (Thank you for participating and ‘your survey is completed') |
| No (Thank you for participating and ‘your survey is completed') |
| No (Thank you for participating and ‘your survey is completed') |
| No (Thank you for participating and ‘your survey is completed') |
| No (Thank you for participating and ‘your survey is completed') |
| Yes |
| No (Thank you for participating and ‘your survey is completed') |
| No (Thank you for participating and ‘your survey is completed') |
| No (Thank you for participating and ‘your survey is completed') |
| No (Thank you for participating and ‘your survey is completed') |
| No (Thank you for participating and ‘your survey is completed') |
| Yes |
| No (Thank you for participating and ‘your survey is completed') |
| No (Thank you for participating and ‘your survey is completed') |
| No (Thank you for participating and ‘your survey is completed') |
| No (Thank you for participating and ‘your survey is completed') |
| No (Thank you for participating and ‘your survey is completed') |
| No (Thank you for participating and ‘your survey is completed') |
| No (Thank you for participating and ‘your survey is completed') |
| No (Thank you for participating and ‘your survey is completed') |
| No (Thank you for participating and ‘your survey is completed') |
| No (Thank you for participating and ‘your survey is completed') |
| No (Thank you for participating and ‘your survey is completed') |
| No (Thank you for participating and ‘your survey is completed') |
| No (Thank you for participating and ‘your survey is completed') |

No (Thank you for participating and ‘your survey is completed')
No (Thank you for participating and ‘your survey is completed')
No (Thank you for participating and ‘your survey is completed')
Yes
No (Thank you for participating and ‘your survey is completed')
No (Thank you for participating and ‘your survey is completed')
No (Thank you for participating and ‘your survey is completed')
No (Thank you for participating and ‘your survey is completed')
No (Thank you for participating and ‘your survey is completed')
No (Thank you for participating and ‘your survey is completed')
Yes
No (Thank you for participating and ‘your survey is completed')
No (Thank you for participating and ‘your survey is completed')
No (Thank you for participating and ‘your survey is completed')
Yes
No (Thank you for participating and ‘your survey is completed')
No (Thank you for participating and ‘your survey is completed')
No (Thank you for participating and ‘your survey is completed')
Do not know/unsure
No (Thank you for participating and ‘your survey is completed')
No (Thank you for participating and ‘your survey is completed')
No (Thank you for participating and ‘your survey is completed')
No (Thank you for participating and ‘your survey is completed')
No (Thank you for participating and ‘your survey is completed')
No (Thank you for participating and ‘your survey is completed')
No (Thank you for participating and ‘your survey is completed')
No (Thank you for participating and ‘your survey is completed')
No (Thank you for participating and ‘your survey is completed')
Do not know/unsure
No (Thank you for participating and ‘your survey is completed')
No (Thank you for participating and ‘your survey is completed')
Yes
No (Thank you for participating and ‘your survey is completed')
Yes
No (Thank you for participating and ‘your survey is completed')
No (Thank you for participating and ‘your survey is completed')
Yes
No (Thank you for participating and ‘your survey is completed')
Do not know/unsure
No (Thank you for participating and ‘your survey is completed')
No (Thank you for participating and ‘your survey is completed')
No (Thank you for participating and ‘your survey is completed')
No (Thank you for participating and ‘your survey is completed')
Yes
Yes
No (Thank you for participating and ‘your survey is completed')
No (Thank you for participating and ‘your survey is completed')
No (Thank you for participating and ‘your survey is completed')

Yes
Yes
No (Thank you for participating and ‘your survey is completed')
No (Thank you for participating and ‘your survey is completed')
No (Thank you for participating and ‘your survey is completed')
No (Thank you for participating and ‘your survey is completed')
No (Thank you for participating and ‘your survey is completed')
No (Thank you for participating and ‘your survey is completed')
No (Thank you for participating and ‘your survey is completed')
No (Thank you for participating and ‘your survey is completed')
No (Thank you for participating and ‘your survey is completed')
No (Thank you for participating and ‘your survey is completed')
No (Thank you for participating and ‘your survey is completed')
Yes

Yes
Yes
Yes
No (Thank you for participating and ‘your survey is completed')
Yes
Yes
Yes
Yes
Yes
Yes
Yes
Yes
Yes
Yes
No (Thank you for participating and ‘your survey is completed')
Yes
Yes
Yes
Yes
Yes
No (Thank you for participating and ‘your survey is completed')
Yes
Yes
Yes
No (Thank you for participating and ‘your survey is completed')
No (Thank you for participating and ‘your survey is completed')
Yes
Yes
No (Thank you for participating and ‘your survey is completed')
Yes
Yes

Yes
Yes
Yes
No (Thank you for participating and 'your survey is completed')
No (Thank you for participating and 'your survey is completed')
Yes
No (Thank you for participating and 'your survey is completed')
No (Thank you for participating and 'your survey is completed')
Yes
No (Thank you for participating and 'your survey is completed')
No (Thank you for participating and 'your survey is completed')
No (Thank you for participating and 'your survey is completed')
No (Thank you for participating and 'your survey is completed')
Yes
No (Thank you for participating and 'your survey is completed')
Yes
No (Thank you for participating and 'your survey is completed')

Yes
Yes
No (Thank you for participating and 'your survey is completed')
No (Thank you for participating and 'your survey is completed')
No (Thank you for participating and 'your survey is completed')
No (Thank you for participating and 'your survey is completed')
No (Thank you for participating and 'your survey is completed')
Yes
No (Thank you for participating and 'your survey is completed')
No (Thank you for participating and 'your survey is completed')
Yes
Yes
No (Thank you for participating and 'your survey is completed')
No (Thank you for participating and 'your survey is completed')
Yes
Yes
Yes
No (Thank you for participating and 'your survey is completed')
No (Thank you for participating and 'your survey is completed')

No (Thank you for participating and 'your survey is completed')
No (Thank you for participating and 'your survey is completed')
No (Thank you for participating and 'your survey is completed')
No (Thank you for participating and 'your survey is completed')
No (Thank you for participating and 'your survey is completed')
No (Thank you for participating and 'your survey is completed')
Yes
Yes

No (Thank you for participating and ‘your survey is completed')
No (Thank you for participating and ‘your survey is completed')
No (Thank you for participating and ‘your survey is completed')
Yes
Yes
No (Thank you for participating and ‘your survey is completed')
No (Thank you for participating and ‘your survey is completed')
No (Thank you for participating and ‘your survey is completed')
No (Thank you for participating and ‘your survey is completed')
No (Thank you for participating and ‘your survey is completed')
Yes
No (Thank you for participating and ‘your survey is completed')
No (Thank you for participating and ‘your survey is completed')
No (Thank you for participating and ‘your survey is completed')
Yes
No (Thank you for participating and ‘your survey is completed')
No (Thank you for participating and ‘your survey is completed')
No (Thank you for participating and ‘your survey is completed')
No (Thank you for participating and ‘your survey is completed')
No (Thank you for participating and ‘your survey is completed')

No (Thank you for participating and ‘your survey is completed')
Yes
No (Thank you for participating and ‘your survey is completed')
No (Thank you for participating and ‘your survey is completed')
No (Thank you for participating and ‘your survey is completed')
No (Thank you for participating and ‘your survey is completed')
No (Thank you for participating and ‘your survey is completed')
No (Thank you for participating and ‘your survey is completed')
No (Thank you for participating and ‘your survey is completed')
Yes
No (Thank you for participating and ‘your survey is completed')
Yes
No (Thank you for participating and ‘your survey is completed')
Yes
No (Thank you for participating and ‘your survey is completed')
Yes
No (Thank you for participating and ‘your survey is completed')
Yes
No (Thank you for participating and ‘your survey is completed')
No (Thank you for participating and ‘your survey is completed')
No (Thank you for participating and ‘your survey is completed')
No (Thank you for participating and ‘your survey is completed')
No (Thank you for participating and ‘your survey is completed')
No (Thank you for participating and ‘your survey is completed')
No (Thank you for participating and ‘your survey is completed')
No (Thank you for participating and ‘your survey is completed')
No (Thank you for participating and ‘your survey is completed')

No (Thank you for participating and ‘your survey is completed')
No (Thank you for participating and ‘your survey is completed')
No (Thank you for participating and ‘your survey is completed')
No (Thank you for participating and ‘your survey is completed')
No (Thank you for participating and ‘your survey is completed')
No (Thank you for participating and ‘your survey is completed')
No (Thank you for participating and ‘your survey is completed')
No (Thank you for participating and ‘your survey is completed')
No (Thank you for participating and ‘your survey is completed')
Yes
No (Thank you for participating and ‘your survey is completed')
No (Thank you for participating and ‘your survey is completed')
No (Thank you for participating and ‘your survey is completed')
No (Thank you for participating and ‘your survey is completed')
Do not know/unsure
Yes
No (Thank you for participating and ‘your survey is completed')
No (Thank you for participating and ‘your survey is completed')
No (Thank you for participating and ‘your survey is completed')
No (Thank you for participating and ‘your survey is completed')
No (Thank you for participating and ‘your survey is completed')
No (Thank you for participating and ‘your survey is completed')
No (Thank you for participating and ‘your survey is completed')
No (Thank you for participating and ‘your survey is completed')
Yes
No (Thank you for participating and ‘your survey is completed')
No (Thank you for participating and ‘your survey is completed')
No (Thank you for participating and ‘your survey is completed')
No (Thank you for participating and ‘your survey is completed')
No (Thank you for participating and ‘your survey is completed')
No (Thank you for participating and ‘your survey is completed')
No (Thank you for participating and ‘your survey is completed')
No (Thank you for participating and ‘your survey is completed')
Yes
No (Thank you for participating and ‘your survey is completed')
Yes
No (Thank you for participating and ‘your survey is completed')
Yes
No (Thank you for participating and ‘your survey is completed')
No (Thank you for participating and ‘your survey is completed')
No (Thank you for participating and ‘your survey is completed')
No (Thank you for participating and ‘your survey is completed')
No (Thank you for participating and ‘your survey is completed')
No (Thank you for participating and ‘your survey is completed')
No (Thank you for participating and ‘your survey is completed')
No (Thank you for participating and ‘your survey is completed')
No (Thank you for participating and ‘your survey is completed')
No (Thank you for participating and ‘your survey is completed')

No (Thank you for participating and ‘your survey is completed')
No (Thank you for participating and ‘your survey is completed')
No (Thank you for participating and ‘your survey is completed')
No (Thank you for participating and ‘your survey is completed')
Do not know/unsure
No (Thank you for participating and ‘your survey is completed')
No (Thank you for participating and ‘your survey is completed')
No (Thank you for participating and ‘your survey is completed')
No (Thank you for participating and ‘your survey is completed')
No (Thank you for participating and ‘your survey is completed')
No (Thank you for participating and ‘your survey is completed')
No (Thank you for participating and ‘your survey is completed')
No (Thank you for participating and ‘your survey is completed')
No (Thank you for participating and ‘your survey is completed')
No (Thank you for participating and ‘your survey is completed')
No (Thank you for participating and ‘your survey is completed')
Yes
No (Thank you for participating and ‘your survey is completed')
No (Thank you for participating and ‘your survey is completed')
No (Thank you for participating and ‘your survey is completed')
No (Thank you for participating and ‘your survey is completed')
No (Thank you for participating and ‘your survey is completed')
No (Thank you for participating and ‘your survey is completed')
No (Thank you for participating and ‘your survey is completed')
No (Thank you for participating and ‘your survey is completed')
No (Thank you for participating and ‘your survey is completed')
No (Thank you for participating and ‘your survey is completed')
No (Thank you for participating and ‘your survey is completed')
No (Thank you for participating and ‘your survey is completed')
No (Thank you for participating and ‘your survey is completed')
No (Thank you for participating and ‘your survey is completed')
No (Thank you for participating and ‘your survey is completed')
Yes
No (Thank you for participating and ‘your survey is completed')
No (Thank you for participating and ‘your survey is completed')
No (Thank you for participating and ‘your survey is completed')
No (Thank you for participating and ‘your survey is completed')
No (Thank you for participating and ‘your survey is completed')
No (Thank you for participating and ‘your survey is completed')
No (Thank you for participating and ‘your survey is completed')
No (Thank you for participating and ‘your survey is completed')
No (Thank you for participating and ‘your survey is completed')
No (Thank you for participating and ‘your survey is completed')
No (Thank you for participating and ‘your survey is completed')
No (Thank you for participating and ‘your survey is completed')
No (Thank you for participating and ‘your survey is completed')
No (Thank you for participating and ‘your survey is completed')
Yes

No (Thank you for participating and ‘your survey is completed')
Yes
No (Thank you for participating and ‘your survey is completed')
No (Thank you for participating and ‘your survey is completed')
No (Thank you for participating and ‘your survey is completed')
No (Thank you for participating and ‘your survey is completed')
No (Thank you for participating and ‘your survey is completed')
No (Thank you for participating and ‘your survey is completed')
No (Thank you for participating and ‘your survey is completed')
No (Thank you for participating and ‘your survey is completed')
No (Thank you for participating and ‘your survey is completed')
No (Thank you for participating and ‘your survey is completed')
No (Thank you for participating and ‘your survey is completed')
No (Thank you for participating and ‘your survey is completed')
No (Thank you for participating and ‘your survey is completed')
No (Thank you for participating and ‘your survey is completed')
No (Thank you for participating and ‘your survey is completed')
No (Thank you for participating and ‘your survey is completed')
No (Thank you for participating and ‘your survey is completed')
No (Thank you for participating and ‘your survey is completed')
No (Thank you for participating and ‘your survey is completed')
No (Thank you for participating and ‘your survey is completed')
No (Thank you for participating and ‘your survey is completed')
No (Thank you for participating and ‘your survey is completed')
No (Thank you for participating and ‘your survey is completed')
No (Thank you for participating and ‘your survey is completed')
No (Thank you for participating and ‘your survey is completed')
Yes
No (Thank you for participating and ‘your survey is completed')
No (Thank you for participating and ‘your survey is completed')
No (Thank you for participating and ‘your survey is completed')
No (Thank you for participating and ‘your survey is completed')
No (Thank you for participating and ‘your survey is completed')
No (Thank you for participating and ‘your survey is completed')
No (Thank you for participating and ‘your survey is completed')
No (Thank you for participating and ‘your survey is completed')
No (Thank you for participating and ‘your survey is completed')
Yes
No (Thank you for participating and ‘your survey is completed')
No (Thank you for participating and ‘your survey is completed')
Do not know/unsure
No (Thank you for participating and ‘your survey is completed')
No (Thank you for participating and ‘your survey is completed')
No (Thank you for participating and ‘your survey is completed')
Yes
No (Thank you for participating and ‘your survey is completed')
Yes
Yes

No (Thank you for participating and ‘your survey is completed')
No (Thank you for participating and ‘your survey is completed')
No (Thank you for participating and ‘your survey is completed')
Yes
No (Thank you for participating and ‘your survey is completed')
No (Thank you for participating and ‘your survey is completed')
No (Thank you for participating and ‘your survey is completed')
No (Thank you for participating and ‘your survey is completed')
No (Thank you for participating and ‘your survey is completed')
No (Thank you for participating and ‘your survey is completed')
No (Thank you for participating and ‘your survey is completed')
No (Thank you for participating and ‘your survey is completed')
No (Thank you for participating and ‘your survey is completed')
No (Thank you for participating and ‘your survey is completed')
No (Thank you for participating and ‘your survey is completed')
No (Thank you for participating and ‘your survey is completed')
No (Thank you for participating and ‘your survey is completed')
No (Thank you for participating and ‘your survey is completed')
No (Thank you for participating and ‘your survey is completed')
No (Thank you for participating and ‘your survey is completed')
No (Thank you for participating and ‘your survey is completed')
No (Thank you for participating and ‘your survey is completed')
No (Thank you for participating and ‘your survey is completed')
No (Thank you for participating and ‘your survey is completed')
No (Thank you for participating and ‘your survey is completed')
No (Thank you for participating and ‘your survey is completed')
No (Thank you for participating and ‘your survey is completed')
No (Thank you for participating and ‘your survey is completed')
No (Thank you for participating and ‘your survey is completed')
No (Thank you for participating and ‘your survey is completed')
No (Thank you for participating and ‘your survey is completed')
No (Thank you for participating and ‘your survey is completed')
No (Thank you for participating and ‘your survey is completed')
No (Thank you for participating and ‘your survey is completed')
No (Thank you for participating and ‘your survey is completed')
No (Thank you for participating and ‘your survey is completed')
No (Thank you for participating and ‘your survey is completed')
No (Thank you for participating and ‘your survey is completed')

Yes
Yes
Yes
No (Thank you for participating and ‘your survey is completed')
No (Thank you for participating and ‘your survey is completed')
No (Thank you for participating and ‘your survey is completed')
No (Thank you for participating and ‘your survey is completed')
No (Thank you for participating and ‘your survey is completed')
No (Thank you for participating and ‘your survey is completed')

No (Thank you for participating and ‘your survey is completed')
Do not know/unsure
No (Thank you for participating and ‘your survey is completed')
No (Thank you for participating and ‘your survey is completed')
No (Thank you for participating and ‘your survey is completed')
No (Thank you for participating and ‘your survey is completed')

No (Thank you for participating and ‘your survey is completed')
No (Thank you for participating and ‘your survey is completed')
Yes
Yes
Yes
Yes
Yes
Yes
Yes
Yes
Yes
Yes
No (Thank you for participating and ‘your survey is completed')
No (Thank you for participating and ‘your survey is completed')
No (Thank you for participating and ‘your survey is completed')
No (Thank you for participating and ‘your survey is completed')
Yes
No (Thank you for participating and ‘your survey is completed')
Yes
No (Thank you for participating and ‘your survey is completed')
No (Thank you for participating and ‘your survey is completed')
No (Thank you for participating and ‘your survey is completed')
No (Thank you for participating and ‘your survey is completed')
No (Thank you for participating and ‘your survey is completed')
No (Thank you for participating and ‘your survey is completed')
No (Thank you for participating and ‘your survey is completed')
No (Thank you for participating and ‘your survey is completed')
No (Thank you for participating and ‘your survey is completed')
No (Thank you for participating and ‘your survey is completed')
No (Thank you for participating and ‘your survey is completed')
No (Thank you for participating and ‘your survey is completed')
Yes
No (Thank you for participating and ‘your survey is completed')
Yes
No (Thank you for participating and ‘your survey is completed')
No (Thank you for participating and ‘your survey is completed')
No (Thank you for participating and ‘your survey is completed')
Yes
No (Thank you for participating and ‘your survey is completed')
No (Thank you for participating and ‘your survey is completed')
No (Thank you for participating and ‘your survey is completed')

Yes
No (Thank you for participating and ‘your survey is completed')
No (Thank you for participating and ‘your survey is completed')
No (Thank you for participating and ‘your survey is completed')
No (Thank you for participating and ‘your survey is completed')
Yes

Yes
Yes
Yes
No (Thank you for participating and ‘your survey is completed')
No (Thank you for participating and ‘your survey is completed')
No (Thank you for participating and ‘your survey is completed')
No (Thank you for participating and ‘your survey is completed')
No (Thank you for participating and ‘your survey is completed')
No (Thank you for participating and ‘your survey is completed')
No (Thank you for participating and ‘your survey is completed')
Yes
No (Thank you for participating and ‘your survey is completed')
No (Thank you for participating and ‘your survey is completed')
No (Thank you for participating and ‘your survey is completed')
Yes
Yes
No (Thank you for participating and ‘your survey is completed')
No (Thank you for participating and ‘your survey is completed')
No (Thank you for participating and ‘your survey is completed')
No (Thank you for participating and ‘your survey is completed')
No (Thank you for participating and ‘your survey is completed')
Yes
No (Thank you for participating and ‘your survey is completed')
No (Thank you for participating and ‘your survey is completed')
No (Thank you for participating and ‘your survey is completed')
No (Thank you for participating and ‘your survey is completed')
No (Thank you for participating and ‘your survey is completed')
No (Thank you for participating and ‘your survey is completed')
No (Thank you for participating and ‘your survey is completed')
No (Thank you for participating and ‘your survey is completed')
No (Thank you for participating and ‘your survey is completed')
No (Thank you for participating and ‘your survey is completed')
No (Thank you for participating and ‘your survey is completed')
No (Thank you for participating and ‘your survey is completed')
No (Thank you for participating and ‘your survey is completed')
No (Thank you for participating and ‘your survey is completed')
No (Thank you for participating and ‘your survey is completed')
No (Thank you for participating and ‘your survey is completed')
No (Thank you for participating and ‘your survey is completed')
No (Thank you for participating and ‘your survey is completed')
No (Thank you for participating and ‘your survey is completed')

No (Thank you for participating and ‘your survey is completed')
Yes
No (Thank you for participating and ‘your survey is completed')
Yes
No (Thank you for participating and ‘your survey is completed')
Yes
No (Thank you for participating and ‘your survey is completed')
Yes
No (Thank you for participating and ‘your survey is completed')
No (Thank you for participating and ‘your survey is completed')
No (Thank you for participating and ‘your survey is completed')
Yes
No (Thank you for participating and ‘your survey is completed')
Yes
Yes
No (Thank you for participating and ‘your survey is completed')
Yes
Yes
Yes
No (Thank you for participating and ‘your survey is completed')
No (Thank you for participating and ‘your survey is completed')
No (Thank you for participating and ‘your survey is completed')
Yes
Yes
No (Thank you for participating and ‘your survey is completed')
Yes
No (Thank you for participating and ‘your survey is completed')
Yes
No (Thank you for participating and ‘your survey is completed')
Yes
Yes
Yes
Yes

| Q4<br>If 'Yes', which one? (select all that apply) |
|---|
| |
| Modafinil,Adderall/amphetamines mixture,Ritalin/amphetamine,Super strength caffeine pills,Other drug or substanc |
| Super strength caffeine pills |
| |
| Super strength caffeine pills |
| |
| Modafinil |
| |
| Alpha Brain/vitamin B6,Cobalamin/ vitamin B12 |

Cobalamin/ vitamin B12

Super strength caffeine pills

Super strength caffeine pills,Alpha Brain/vitamin B6,Cobalamin/ vitamin B12,Other drug or substance used

Cobalamin/ vitamin B12

Super strength caffeine pills

Super strength caffeine pills,Alpha Brain/vitamin B6,Cobalamin/ vitamin B12

Super strength caffeine pills

Alpha Brain/vitamin B6,Cobalamin/ vitamin B12

Other drug or substance used

Ritalin/amphetamine
MDMA/ ecstasy,Cobalamin/ vitamin B12

Super strength caffeine pills,Guarana
Modafinil

Modafinil,Cobalamin/ vitamin B12

Adderall/amphetamines mixture
Super strength caffeine pills
Super strength caffeine pills

Super strength caffeine pills,Cobalamin/ vitamin B12
Modafinil
Modafinil,Super strength caffeine pills
Modafinil,Super strength caffeine pills,Cobalamin/ vitamin B12
Modafinil,Cobalamin/ vitamin B12
Cobalamin/ vitamin B12
Modafinil
Modafinil,Cobalamin/ vitamin B12
Modafinil,Cobalamin/ vitamin B12
Super strength caffeine pills

Modafinil,Super strength caffeine pills
Modafinil,Super strength caffeine pills,Guarana
Super strength caffeine pills,Cobalamin/ vitamin B12
Modafinil,Super strength caffeine pills,Cobalamin/ vitamin B12
Super strength caffeine pills

Modafinil,Cobalamin/ vitamin B12
Modafinil
Adderall/amphetamines mixture

Modafinil,Super strength caffeine pills
Adderall/amphetamines mixture

Adderall/amphetamines mixture,Ritalin/amphetamine
Modafinil,Cobalamin/ vitamin B12

Modafinil,Cobalamin/ vitamin B12
Modafinil,Super strength caffeine pills
Adderall/amphetamines mixture,Ritalin/amphetamine

Modafinil,Super strength caffeine pills

Adderall/amphetamines mixture,Ritalin/amphetamine

Modafinil,Cobalamin/ vitamin B12

MDMA/ ecstasy

Modafinil
Modafinil,Super strength caffeine pills

Ritalin/amphetamine

Modafinil
Modafinil

Guarana,Piracetam
Adderall/amphetamines mixture
Modafinil

Modafinil
Modafinil,Super strength caffeine pills

Modafinil
Modafinil

Modafinil,Ritalin/amphetamine

Modafinil,Ritalin/amphetamine

Modafinil

Super strength caffeine pills

Modafinil,Super strength caffeine pills

Super strength caffeine pills

Modafinil,Guarana

Modafinil,Super strength caffeine pills

Other drug or substance used

Other drug or substance used
Cobalamin/ vitamin B12

Ritalin/amphetamine,Alpha Brain/vitamin B6,Cobalamin/ vitamin B12

Cobalamin/ vitamin B12

Ritalin/amphetamine

Cobalamin/ vitamin B12

Alpha Brain/vitamin B6,Cobalamin/ vitamin B12

Cobalamin/ vitamin B12,Other drug or substance used

Cobalamin/ vitamin B12

MDMA/ ecstasy,Alpha Brain/vitamin B6

Alpha Brain/vitamin B6

Ritalin/amphetamine

Super strength caffeine pills

Cobalamin/ vitamin B12

Super strength caffeine pills

Other drug or substance used
Other drug or substance used

Super strength caffeine pills

Modafinil

Super strength caffeine pills,Cobalamin/ vitamin B12

Super strength caffeine pills,Cobalamin/ vitamin B12

Modafinil,Cobalamin/ vitamin B12
Super strength caffeine pills,Cobalamin/ vitamin B12
Super strength caffeine pills,Cobalamin/ vitamin B12
Super strength caffeine pills,Cobalamin/ vitamin B12
Modafinil,Cobalamin/ vitamin B12
Adderall/amphetamines mixture
Modafinil,Alpha Brain/vitamin B6
Super strength caffeine pills,Cobalamin/ vitamin B12
Modafinil,Cobalamin/ vitamin B12
Modafinil,Cobalamin/ vitamin B12

Adderall/amphetamines mixture,Ritalin/amphetamine

Super strength caffeine pills,Alpha Brain/vitamin B6,Cobalamin/ vitamin B12

Super strength caffeine pills,Cobalamin/ vitamin B12

Ritalin/amphetamine

Super strength caffeine pills,Cobalamin/ vitamin B12

Adderall/amphetamines mixture

Modafinil

Modafinil,Cobalamin/ vitamin B12,Vinpocetine
Adderall/amphetamines mixture
Ritalin/amphetamine

Super strength caffeine pills,Cobalamin/ vitamin B12

Super strength caffeine pills,Guarana,Cobalamin/ vitamin B12
Super strength caffeine pills,Cobalamin/ vitamin B12

Super strength caffeine pills,Cobalamin/ vitamin B12

Other drug or substance used

Modafinil,Super strength caffeine pills,Cobalamin/ vitamin B12

Super strength caffeine pills,Alpha Brain/vitamin B6

Super strength caffeine pills

Super strength caffeine pills
Alpha Brain/vitamin B6

Cobalamin/ vitamin B12
Cobalamin/ vitamin B12,Vinpocetine
Alpha Brain/vitamin B6,Cobalamin/ vitamin B12

Super strength caffeine pills,Vinpocetine
Modafinil

Modafinil

Cobalamin/ vitamin B12

Modafinil
Modafinil
Super strength caffeine pills,Alpha Brain/vitamin B6
Super strength caffeine pills

| Q5<br>Nationality - Selected Choice | Q5_6_TEXT<br>Nationality - Other, (please specify) - Text |
|---|---|
| | |
| | |
| | |
| | |
| Other, (please specify) | Dutch |
| UK | |
| UK | |
| EU | |
| | |
| | |
| UK | |
| | |
| EU | |
| | |
| UK | |
| UK | |
| Arab Nationality | |
| UK | |
| Other, (please specify) | |
| | |
| | |
| Other, (please specify) | |
| | |
| | |
| | |
| UK | |
| UK | |
| Arab Nationality | |
| Other, (please specify) | Hertfordshire |
| UK | |
| EU | |
| Other, (please specify) | Indian |
| Arab Nationality | |
| Arab Nationality | |
| UAE | |
| | |
| | |
| Other, (please specify) | Indian |
| | |
| | |
| Arab Nationality | |
| | |
| | |
| Other, (please specify) | Tanzania |

| | |
|---|---|
| UAE | |
| Arab Nationality | |
| UAE | |
| Arab Nationality | |
| Arab Nationality | |
| Arab Nationality | |
| Other, (please specify) | Ras Al Khaimah |
| | |
| Other, (please specify) | Indian |
| Other, (please specify) | Pakistani |
| Other, (please specify) | Dubai |
| | |
| Arab Nationality | |
| | |
| | |
| | |
| Arab Nationality | |
| Arab Nationality | |
| Other, (please specify) | indain |
| | |
| | |
| | |
| Other, (please specify) | Indian |
| | |
| UK | |
| | |
| UK | |
| | |
| | |
| UK | |
| | |
| UAE | |
| | |
| | |
| UK | |
| | |
| UK | |
| | |
| UK | |
| UK | |
| | |
| Other, (please specify) | Indian |
| Other, (please specify) | Indian |
| Arab Nationality | |

UK
UAE

Arab Nationality

UAE

UK
UAE

UAE
UAE
UAE

UAE
UAE
UAE
UAE
UAE
Arab Nationality
Arab Nationality
UAE
UAE
UAE
UAE
Arab Nationality
UAE
UAE
UAE
UAE

UAE
Arab Nationality
Arab Nationality

UAE
UAE

UAE
UAE

UAE
UAE
Arab Nationality

UAE

UAE

Arab Nationality

Arab Nationality

Arab Nationality
Arab Nationality

Other, (please specify) India

UAE
UAE

UAE
UAE
UAE

UK
Arab Nationality

UAE
UAE

Arab Nationality

Arab Nationality

Arab Nationality

UAE

UAE

UAE

Arab Nationality

UAE

Arab Nationality

| | |
|---|---|
| Other, (please specify) | Indian |
| Other, (please specify) | Indian |
| Other, (please specify) | Indian |
| Arab Nationality | |
| Other, (please specify) | Tanzanian |
| Other, (please specify) | Bangladesh |
| USA | |
| UK | |
| Other, (please specify) | South African |
| Arab Nationality | |
| Other, (please specify) | African |
| Other, (please specify) | indian |
| Other, (please specify) | Indian |
| Other, (please specify) | Nigerian |
| Other, (please specify) | Indian |
| Arab Nationality | |
| Other, (please specify) | indian |
| Arab Nationality | |
| UAE | |
| Arab Nationality | |
| Other, (please specify) | Indian |
| Other, (please specify) | India |
| UAE | |

| | |
|---|---|
| Other, (please specify) | Indian |
| UAE | |
| UK | |
| Other, (please specify) | Nigerian |
| UAE | |
| Other, (please specify) | Pakistani |
| UAE | |
| Other, (please specify) | srilanka |
| Other, (please specify) | Indian |
| Arab Nationality | |
| Arab Nationality | |
| Other, (please specify) | Nigeria |
| Arab Nationality | |
| Arab Nationality | |
| UAE | |
| Other, (please specify) | Indian |
| Other, (please specify) | India |
| Other, (please specify) | India |
| Other, (please specify) | Indian |
| Other, (please specify) | Nigerian |
| Other, (please specify) | |
| Other, (please specify) | Indian |
| Other, (please specify) | Abu Dhabi |
| Arab Nationality | |

| | |
|---|---|
| Arab Nationality | |
| Other, (please specify) | Philippines |
| UAE | |
| Other, (please specify) | INDIAN |
| Other, (please specify) | Pakistani |
| Arab Nationality | |
| Other, (please specify) | Pakistani |
| Arab Nationality | |
| Other, (please specify) | Indian |
| Other, (please specify) | |
| Other, (please specify) | Nigerian |
| Other, (please specify) | Indian |
| Other, (please specify) | |
| Other, (please specify) | Nigeria |
| Arab Nationality | |
| Other, (please specify) | MI |
| Other, (please specify) | Indian |
| Other, (please specify) | Indian |
| Other, (please specify) | India |
| Other, (please specify) | pakistan |
| Other, (please specify) | Indian |
| Arab Nationality | |
| Other, (please specify) | chinese and syrian |
| Arab Nationality | |
| Arab Nationality | |
| Other, (please specify) | INDIAN |
| EU | |

| | | |
|---|---|---|
| Other, (please specify) | | |
| | | |
| Arab Nationality | | |
| Other, (please specify) | ZImbabwe | |
| Arab Nationality | | |
| Other, (please specify) | Indian | |
| | | |
| Other, (please specify) | Indian | |
| | | |
| | | |
| | | |
| Other, (please specify) | pakistan | |
| Arab Nationality | | |
| | | |
| | | |
| | | |
| | | |
| | | |
| Other, (please specify) | Indian | |
| | | |
| | | |
| Other, (please specify) | | |
| | | |
| | | |
| | | |
| Other, (please specify) | Iran | |
| | | |
| USA | | |
| Other, (please specify) | Indian | |
| | | |
| | | |
| | | |
| | | |
| | | |
| Other, (please specify) | | إمارة دبيّ |
| | | |
| | | |
| | | |
| | | |
| UAE | | |
| UAE | | |
| Arab Nationality | | |

| | |
|---|---|
| Other, (please specify) | India |
| Other, (please specify) | India |
| Other, (please specify) | Persian |
| Other, (please specify) | Indian |
| UAE | |
| UAE | |
| Arab Nationality | |
| Arab Nationality | |
| Arab Nationality | |
| Other, (please specify) | Indian |
| UAE | |
| UAE | |
| Arab Nationality | |
| Arab Nationality | |
| Other, (please specify) | Indian |
| Other, (please specify) | Indian |
| Other, (please specify) | Iran |
| Arab Nationality | |
| Other, (please specify) | INDIAN |
| Other, (please specify) | Indian |
| Arab Nationality | |
| Arab Nationality | |
| Arab Nationality | |
| Arab Nationality | |
| Other, (please specify) | Pakistan |
| Arab Nationality | |
| UAE | |

| | |
|---|---|
| Arab Nationality | |
| Other, (please specify) | india |
| UAE | |
| Arab Nationality | |
| Arab Nationality | |
| Other, (please specify) | Pakistan |
| Arab Nationality | |
| UAE | |
| Other, (please specify) | |
| Other, (please specify) | Indian |
| Other, (please specify) | India |

UAE

Other, (please specify) Palastine

UAE

UAE

Arab Nationality

UAE
Arab Nationality

Arab Nationality
UAE
UK

UAE
Arab Nationality

Arab Nationality

Arab Nationality

UK
Arab Nationality
Other, (please specify) Indian
Arab Nationality

| Q6 | Q7 |
|---|---|
| What degree are youstudying | Year of Education |
| | |
| | |
| | |
| Pharmacy | First year |
| Pharmacy | First year |
| Pharmacy | First year |
| Pharmacy | First year |
| | |
| Other, (please specify) | First year |
| | |
| Pharmacy | Third year |
| | |
| Engineering | Second year |
| Engineering | Second year |
| Pharmacy | Second year |
| Engineering | Second year |
| Nursing | Third year |
| | |
| Nursing | Third year |
| | |
| Engineering | Third year |
| Nursing | Third year |
| Engineering | Third year |
| | |
| Pharmacy | Second year |
| Pharmacy | Second year |
| Medicine | Third year |
| Nursing | Third year |
| Dentistry | Fourth year |
| Dentistry | Fourth year |
| | |
| Medicine | Third year |
| | |
| Medicine | Fourth year |
| | |
| Medicine | First year |

| | |
|---|---|
| Medicine | Fourth year |
| Medicine | Fourth year |
| Nursing | First year |
| Dentistry | First year |
| Pharmacy | Third year |
| Dentistry | Second year |
| Medicine | Third year |
| | |
| Medicine | Fourth year |
| Nursing | First year |
| | |
| | |
| Medicine | Second year |
| | |
| | |
| | |
| Medicine | First year |
| Nursing | Fourth year |
| Medicine | Fourth year |
| | |
| | |
| | |
| Medicine | Third year |
| | |
| Pharmacy | First year |
| | |
| Pharmacy | Third year |
| | |
| | |
| Pharmacy | First year |
| | |
| Pharmacy | Second year |
| | |
| | |
| Pharmacy | Second year |
| | |
| Pharmacy | First year |
| | |
| Pharmacy | Second year |
| Pharmacy | Fourth year |
| | |
| Medicine | First year |
| Medicine | Fourth year |
| Dentistry | Fourth year |

| | |
|---|---|
| Pharmacy | Third year |
| Medicine | Fourth year |
| Dentistry | First year |
| Medicine | Second year |
| Medicine | Third year |
| Medicine | Fourth year |
| Medicine | Fourth year |
| Medicine | Second year |
| Medicine | Fourth year |
| Medicine | Third year |
| Medicine | Fourth year |
| Medicine | Fifth year |
| Medicine | Fourth year |
| Medicine | Fourth year |
| Medicine | Third year |
| Pharmacy | Third year |
| Pharmacy | Third year |
| Medicine | Third year |
| Medicine | Third year |
| Pharmacy | Fourth year |
| Medicine | Fifth year |
| Medicine | Third year |
| Medicine | Third year |
| Medicine | Second year |
| Medicine | Fourth year |
| Pharmacy | Third year |
| Medicine | Fourth year |
| Pharmacy | Third year |
| Medicine | Fifth year |
| Medicine | Fifth year |
| Medicine | Fourth year |

| | |
|---|---|
| Medicine | Third year |
| Medicine | Fourth year |
| Medicine | Fifth year |
| Medicine | Fourth year |
| Medicine | Fifth year |
| Pharmacy | Fourth year |
| Medicine | Fifth year |
| Pharmacy | Third year |
| Pharmacy | Fourth year |
| Medicine | Third year |
| Pharmacy | Third year |
| Medicine | Fourth year |
| Dentistry | Fourth year |
| Dentistry | Third year |
| Pharmacy | Second year |
| Engineering | Third year |
| Medicine | Fifth year |

| | |
|---|---|
| Medicine | Fifth year |
| Medicine | Fourth year |
| Medicine | Fifth year |
| Engineering | Third year |
| Other, (please specify) | Sixth year |
| Dentistry | Fourth year |
| Pharmacy | Third year |
| Medicine | Fourth year |
| Dentistry | Fourth year |
| Medicine | Fourth year |
| Dentistry | Second year |

| | |
|---|---|
| Other, (please specify) | Third year |
| | |
| Medicine | Fourth year |
| Other, (please specify) | Third year |
| Dentistry | Third year |
| | |
| Medicine | Fourth year |
| Other, (please specify) | Second year |
| | |
| Medicine | Third year |
| | |
| Pharmacy | Second year |
| Medicine | Second year |
| | |
| Dentistry | Fourth year |
| Medicine | First year |
| | |
| Other, (please specify) | Third year |
| | |
| | |
| Medicine | Fourth year |
| Pharmacy | Second year |
| Other, (please specify) | First year |
| | |
| Medicine | Fourth year |
| Dentistry | Third year |
| | |
| Dentistry | Second year |
| Medicine | Second year |
| Medicine | Sixth year |
| | |
| Nursing | First year |
| | |
| | |
| Other, (please specify) | First year |
| Other, (please specify) | First year |
| Other, (please specify) | Third year |
| Pharmacy | First year |

| | |
|---|---|
| Pharmacy | Second year |
| Dentistry | First year |
| Dentistry | Second year |
| Other, (please specify) | Third year |
| Medicine | First year |
| Medicine | Fifth year |
| Dentistry | Fifth year |
| Medicine | First year |
| Medicine | First year |
| Other, (please specify) | Second year |
| Medicine | Fourth year |
| Pharmacy | First year |
| Medicine | Third year |
| Pharmacy | Fifth year |
| Other, (please specify) | Fourth year |
| Pharmacy | Second year |
| Medicine | Fourth year |
| Nursing | Second year |
| Medicine | Fifth year |
| Dentistry | First year |
| Pharmacy | Fourth year |
| Pharmacy | Fifth year |
| Dentistry | Third year |

| | |
|---|---|
| Other, (please specify) | First year |
| Medicine | Third year |
| Medicine | Third year |
| Medicine | First year |
| Pharmacy | Third year |
| Other, (please specify) | Fourth year |
| Medicine | Second year |
| Medicine | Second year |
| Dentistry | First year |
| Other, (please specify) | Third year |
| Medicine | Third year |
| Nursing | First year |
| Medicine | Second year |
| Medicine | First year |
| Medicine | Third year |
| Medicine | Second year |
| Other, (please specify) | Second year |
| Other, (please specify) | First year |
| Medicine | Third year |
| Medicine | Third year |
| Other, (please specify) | Second year |
| Dentistry | Fourth year |
| Pharmacy | Second year |
| Dentistry | First year |

| | |
|---|---|
| Dentistry | First year |
| Medicine | Second year |
| Medicine | First year |
| Medicine | Third year |
| Other, (please specify) | First year |
| Medicine | Fifth year |
| Other, (please specify) | First year |
| Medicine | Third year |
| Medicine | First year |
| Medicine | Fourth year |
| Medicine | Fifth year |
| Other, (please specify) | First year |
| Medicine | Second year |
| Other, (please specify) | Fourth year |
| Engineering | Third year |
| Engineering | Second year |
| Engineering | Fourth year |

| | |
|---|---|
| Other, (please specify) | Second year |
| Other, (please specify) | Fourth year |
| Dentistry | Third year |
| Pharmacy | First year |
| Engineering | Third year |
| Engineering | Fourth year |
| Engineering | Third year |
| Dentistry | Third year |
| Engineering | Fourth year |
| Medicine | Fourth year |
| Engineering | Third year |
| Dentistry | Fourth year |
| Engineering | Fourth year |
| Dentistry | Third year |
| Other, (please specify) | Second year |
| Other, (please specify) | Second year |
| Medicine | Fourth year |
| Dentistry | Fourth year |
| Medicine | Third year |
| Medicine | Third year |
| Other, (please specify) | Third year |
| Other, (please specify) | First year |
| Dentistry | Third year |
| Engineering | Fourth year |
| Other, (please specify) | Second year |
| Other, (please specify) | Fourth year |
| Dentistry | Third year |

| | | |
|---|---|---|
| Medicine | | Third year |
| Dentistry | | Fourth year |
| Medicine | | Fourth year |
| Engineering | | Third year |
| Engineering | | Third year |
| Engineering | | Third year |
| Medicine | | Fifth year |
| Medicine | | Fourth year |
| | 4 | Second year |
| | 6 | Second year |
| | 6 | Fourth year |

| | | |
|---|---|---|
| | 6 | |
| Pharmacy | | Fourth year |
| | 4 | Second year |
| | 5 | Fourth year |
| Medicine | | Third year |
| | 5 | Fourth year |
| Medicine | | Second year |
| Pharmacy | | Fourth year |
| | 3 | Fourth year |
| | 5 | Third year |
| Pharmacy | | Second year |
| Medicine | | Fifth year |
| Medicine | | Third year |
| Pharmacy | | Fourth year |
| Medicine | | Fourth year |
| | 6 | Third year |
| Pharmacy | | Fourth year |
| Medicine | | Fourth year |
| | 5 | Fourth year |

| Q8 |
|---|
| Pleasespecify the reason (s) for use? (select all that apply) - Selected Choice |
| To improve concentration |
| To increase alertness |
| To improve academic performance |
| To improve academic performance<br>Other, please specify |
| To improve concentration |

To improve your memory

Other, please specify

To improve concentration

To improve concentration

To improve your memory

To improve concentration

To improve academic performance

To improve concentration

To improve concentration

Other, please specify

To improve your memory
To improve your memory
To improve academic performance

To improve concentration
To improve concentration

To improve concentration

To improve concentration
To increase alertness
To improve concentration

To improve concentration
To increase alertness
To increase alertness
To improve concentration
To improve your memory
To improve your memory
To increase alertness
To increase alertness
To improve concentration
To increase alertness

To increase alertness
To improve your memory
To improve concentration
To improve concentration
To increase alertness

To increase alertness
To improve concentration
To improve your memory

To increase alertness
To improve your memory

To improve academic performance
To improve your memory

To improve your memory
To increase alertness
To improve academic performance

To improve academic performance

To improve academic performance

To improve academic performance

To improve academic performance

To increase alertness
To increase alertness

To improve academic performance

To improve concentration
To improve concentration

To improve concentration
To improve academic performance
To increase alertness

To increase alertness
To increase alertness

To increase alertness
To improve concentration

To improve concentration

To improve academic performance

To improve concentration

To improve concentration

To increase alertness

To improve concentration

To increase alertness

To improve concentration

To improve concentration

To increase alertness
Other, please specify

To increase alertness
To improve concentration

Other, please specify

Other, please specify

Other, please specify

To improve concentration

Other, please specify

To improve academic performance

Other, please specify

To improve concentration

To improve concentration

Other, please specify
To improve academic performance

Other, please specify
To increase alertness

To increase alertness
To improve academic performance

To improve concentration
Other, please specify

To improve concentration

Other, please specify

To increase alertness

Other, please specify
To improve academic performance

To increase alertness

Other, please specify

Other, please specify

To improve concentration
To improve academic performance
To improve academic performance

To improve academic performance

To improve academic performance
To improve academic performance
To increase alertness
To improve academic performance
To improve academic performance
To improve academic performance
To improve academic performance
To improve academic performance
To improve academic performance
To improve academic performance

To improve academic performance

To improve academic performance

To improve academic performance

To improve academic performance

To improve academic performance

To improve academic performance

To improve academic performance

To improve academic performance

To improve academic performance
To improve academic performance
To improve academic performance

To increase alertness

To increase alertness
To improve concentration

To improve concentration

To improve concentration

To improve concentration

To increase alertness

To increase alertness

To increase alertness
To improve academic performance

To improve academic performance
To improve academic performance

To improve academic performance
To improve academic performance

To increase alertness

To improve academic performance

To improve concentration
To increase alertness
To improve academic performance
To increase alertness

| Q8_5_TEXT<br>Pleasespecify the reason (s) for use? (select all that apply) - Other, please specify - Text |
| --- |
| 07384109354 |

0582062899

I eat almonds(nuts) for improving memory so it is not a drug. it is food.

None

not used

All of the above

I was trying the vegan diet , the supply of B12 was only applicable through syringes or pills

Gmu is unfair university don’t I will told Allah about them in fair day ,they don’t let me go to dentistry because that m

supplementation

general health benefits

0501383649

2489778169

because it was deficient

Reduce stress

I dont use

0569706766

| Q9 |
| --- |
| Do you get positive effects from the use of these drugs/ substances (e.g. euphoria, alertness)? - Selected Choice |
| |
| Yes. If yes, please specify |
| No |
| No |
| No |
| |
| Yes. If yes, please specify |
| Yes. If yes, please specify |
| |
| Yes. If yes, please specify |

No

Yes. If yes, please specify

Yes. If yes, please specify
No

Yes. If yes, please specify

No

No

Yes. If yes, please specify

No

No

No
No

No
Yes. If yes, please specify
No

Yes. If yes, please specify
No

Yes. If yes, please specify

No
Yes. If yes, please specify
Yes. If yes, please specify

No
Yes. If yes, please specify
Yes. If yes, please specify
Yes. If yes, please specify
Yes. If yes, please specify
Yes. If yes, please specify
No
Yes. If yes, please specify
Yes. If yes, please specify
Yes. If yes, please specify

Yes. If yes, please specify
No
Yes. If yes, please specify
Yes. If yes, please specify
Yes. If yes, please specify

Yes. If yes, please specify
Yes. If yes, please specify
Yes. If yes, please specify

Yes. If yes, please specify
Yes. If yes, please specify

Yes. If yes, please specify
Yes. If yes, please specify

Yes. If yes, please specify
Yes. If yes, please specify
Yes. If yes, please specify

Yes. If yes, please specify

Yes. If yes, please specify

No

No

Yes. If yes, please specify
Yes. If yes, please specify

Yes. If yes, please specify

Yes. If yes, please specify
Yes. If yes, please specify

Yes. If yes, please specify
Yes. If yes, please specify
Yes. If yes, please specify

Yes. If yes, please specify
Yes. If yes, please specify

Yes. If yes, please specify
Yes. If yes, please specify

Yes. If yes, please specify

Yes. If yes, please specify

Yes. If yes, please specify

Yes. If yes, please specify

Yes. If yes, please specify

Yes. If yes, please specify

Yes. If yes, please specify

Yes. If yes, please specify

No
Yes. If yes, please specify

No
No

No

Yes. If yes, please specify

Yes. If yes, please specify

No

Yes. If yes, please specify

No

No

No

Yes. If yes, please specify

Yes. If yes, please specify

No

No

Yes. If yes, please specify
No

No

No
Yes. If yes, please specify

No
Yes. If yes, please specify

Yes. If yes, please specify

No
No

Yes. If yes, please specify

No
Yes. If yes, please specify

Yes. If yes, please specify

No

No

Yes. If yes, please specify
Yes. If yes, please specify
Yes. If yes, please specify

No

No

Yes. If yes, please specify
Yes. If yes, please specify
Yes. If yes, please specify
Yes. If yes, please specify
Yes. If yes, please specify
Yes. If yes, please specify
Yes. If yes, please specify
Yes. If yes, please specify
Yes. If yes, please specify
Yes. If yes, please specify
No
No

Yes. If yes, please specify

Yes. If yes, please specify

Yes. If yes, please specify

Yes. If yes, please specify

Yes. If yes, please specify

Yes. If yes, please specify

Yes. If yes, please specify

Yes. If yes, please specify
Yes. If yes, please specify
Yes. If yes, please specify

Yes. If yes, please specify

Yes. If yes, please specify
Yes. If yes, please specify

Yes. If yes, please specify

Yes. If yes, please specify

Yes. If yes, please specify

Yes. If yes, please specify

Yes. If yes, please specify

Yes. If yes, please specify
Yes. If yes, please specify

Yes. If yes, please specify
Yes. If yes, please specify
No

Yes. If yes, please specify
Yes. If yes, please specify

Yes. If yes, please specify

Yes. If yes, please specify

Yes. If yes, please specify
Yes. If yes, please specify
Yes. If yes, please specify
Yes. If yes, please specify

| Q9_9_TEXT<br>Do you get positive effects from the use of these drugs/ substances (e.g. euphoria, alertness)? - Yes. If yes, please specify |
|---|
| Cognitive and physical euphoria, wakefulness, concentration |
| Work work work work work, if you’re not genetically gifted then you have to do what it takes. Even at the expense of o |
| Al10 9UW |
| Reduces stress and helps to stay focus |

none

Increased alertness , concentration

alertness

Alertness
alretness

alretness
alertness
alertness

alertness

alertness

alertness

alertness

alretness

memory

To active

I dont take them

not  used

Study faster and more efficiently and perform better on tests and assignments.

Longer and sharper concentration

alertness

Motivation

good mood, alertness

Increased alertness and focus

48331

Increased focus

Alertness and focus
alertness

| Q10 | Q11 |
| --- | --- |
| When was the time of yourmost recer | How long did you take the CE for? |
| During exam weeks | 1 month but less than 6 months |
| During exam weeks | Less than 1 month |
| During exam weeks | Less than 1 month |
| During course work deadlines | Less than 1 month |
| During exam weeks | Less than 1 month |

| | |
|---|---|
| During exam weeks | Less than 1 month |
| During exam weeks | Less than 1 month |
| During exam weeks | 1 year but less than 2 years |
| During studying | Less than 1 month |
| During exam weeks | 1 month but less than 6 months |
| During studying | 1 month but less than 6 months |
| During exam weeks | Less than 1 month |
| During exam weeks | 1 month but less than 6 months |
| During studying | Less than 1 month |
| During exam weeks | Less than 1 month |
| During exam weeks | Less than 1 month |
| During course work deadlines | Less than 1 month |

| | |
|---|---|
| During exam weeks | 1 month but less than 6 months |
| During exam weeks | Less than 1 month |
| During exam weeks | Less than 1 month |
| During exam weeks | Less than 1 month |
| During exam weeks | 1 month but less than 6 months |
| During exam weeks | Less than 1 month |
| During exam weeks | Less than 1 month |
| During exam weeks | Less than 1 month |
| During exam weeks | 1 month but less than 6 months |
| During exam weeks | 1 month but less than 6 months |
| During exam weeks | 1 month but less than 6 months |
| During exam weeks | 1 month but less than 6 months |
| During exam weeks | Less than 1 month |
| During exam weeks | 1 month but less than 6 months |
| During exam weeks | 1 month but less than 6 months |
| During exam weeks | 1 month but less than 6 months |
| During exam weeks | 1 month but less than 6 months |
| During exam weeks | Less than 1 month |
| During exam weeks | 1 month but less than 6 months |
| During exam weeks | 1 month but less than 6 months |
| During exam weeks | 1 month but less than 6 months |
| During exam weeks | 1 month but less than 6 months |
| During exam weeks | 6 months but less than 1 year |
| During exam weeks | Less than 1 month |
| During exam weeks | 1 month but less than 6 months |
| During exam weeks | 1 month but less than 6 months |
| During exam weeks | 1 month but less than 6 months |
| During exam weeks | 1 month but less than 6 months |

| | |
|---|---|
| During exam weeks | 1 month but less than 6 months |
| During exam weeks | 1 month but less than 6 months |
| During exam weeks | 1 month but less than 6 months |
| During exam weeks | 1 month but less than 6 months |
| During exam weeks | 1 month but less than 6 months |
| During exam weeks | 1 month but less than 6 months |
| During exam weeks | Less than 1 month |
| During exam weeks | Less than 1 month |
| During exam weeks | 1 year but less than 2 years |
| During exam weeks | 1 month but less than 6 months |
| During exam weeks | 1 month but less than 6 months |
| During exam weeks | 6 months but less than 1 year |
| During exam weeks | 6 months but less than 1 year |
| During studying | 1 month but less than 6 months |
| During exam weeks | 1 month but less than 6 months |
| During exam weeks | Less than 1 month |
| During exam weeks | 1 month but less than 6 months |

| | |
|---|---|
| During exam weeks | 1 month but less than 6 months |
| During exam weeks | 1 month but less than 6 months |
| | 1 month but less than 6 months |
| During exam weeks | Less than 1 month |
| During exam weeks | Less than 1 month |
| During exam weeks | 1 month but less than 6 months |
| During exam weeks | 1 month but less than 6 months |
| During exam weeks | Less than 1 month |
| During exam weeks | 1 month but less than 6 months |
| During exam weeks | 1 month but less than 6 months |

| | |
|---|---|
| During exam weeks | Less than 1 month |
| During course work deadlines | Less than 1 month |
| Daily basis | 2 years and more |
| During exam weeks | Less than 1 month |
| During exam weeks | Less than 1 month |
| During course work deadlines | 6 months but less than 1 year |
| Daily basis | Less than 1 month |

| | |
|---|---|
| During exam weeks | 1 month but less than 6 months |
| Daily basis | 1 month but less than 6 months |
| Daily basis | Less than 1 month |
| During studying | Less than 1 month |
| During exam weeks | Less than 1 month |
| During exam weeks | Less than 1 month |

| | |
|---|---|
| During exam weeks | Less than 1 month |
| During exam weeks | 1 month but less than 6 months |
| Daily basis | 1 month but less than 6 months |
| During exam weeks | Less than 1 month |
| During studying | 6 months but less than 1 year |
| During exam weeks | Less than 1 month |
| During exam weeks | Less than 1 month |
| During exam weeks | Less than 1 month |

During exam weeks

| | |
|---|---|
| During exam weeks | 1 month but less than 6 months |
| During exam weeks | 6 months but less than 1 year |
| During exam weeks | 1 month but less than 6 months |

| | |
|---|---|
| During exam weeks | 1 month but less than 6 months |
| During exam weeks | 1 month but less than 6 months |
| During exam weeks | 1 month but less than 6 months |
| During exam weeks | 1 month but less than 6 months |
| During exam weeks | 1 month but less than 6 months |
| During exam weeks | 1 month but less than 6 months |
| During exam weeks | 1 month but less than 6 months |
| During exam weeks | 1 month but less than 6 months |
| During exam weeks | 1 month but less than 6 months |
| During exam weeks | 1 month but less than 6 months |
| During exam weeks | 1 month but less than 6 months |
| During exam weeks | 6 months but less than 1 year |
| During studying | |
| During exam weeks | 1 month but less than 6 months |
| During studying | Less than 1 month |
| During exam weeks | 1 month but less than 6 months |

| | |
|---|---|
| During exam weeks | Less than 1 month |
| During exam weeks | Less than 1 month |
| During exam weeks | 1 month but less than 6 months |
| During exam weeks | 1 month but less than 6 months |
| During exam weeks | 1 month but less than 6 months |
| During studying | 1 month but less than 6 months |
| During studying | 6 months but less than 1 year |
| During studying | 6 months but less than 1 year |

| | |
|---|---|
| During exam weeks | 1 month but less than 6 months |
| During exam weeks | Less than 1 month |
| During exam weeks | 6 months but less than 1 year |
| During exam weeks | 1 month but less than 6 months |
| During studying | 1 month but less than 6 months |
| During exam weeks | 1 month but less than 6 months |
| During studying | 6 months but less than 1 year |
| During exam weeks | 1 month but less than 6 months |
| During exam weeks | 6 months but less than 1 year |
| Daily basis | 2 years and more |
| During studying | 6 months but less than 1 year |
| During exam weeks | 1 month but less than 6 months |
| During exam weeks | 1 month but less than 6 months |
| During exam weeks | 6 months but less than 1 year |
| During course work deadlines | Less than 1 month |
| During studying | 6 months but less than 1 year |
| During studying | 1 month but less than 6 months |
| During studying | 1 month but less than 6 months |

| Q12 | Q13 |
| --- | --- |
| Approximately, how frequentlydid you use t | Where did you obtain thesedrugs/substances? - Sel |
| Daily | I purchased them online |
| Weekly | I purchased them online |
| Once a semester/term | I purchased them online |
| Daily | They were given to me by a friend |
| Once a semester/term | They are prescribed for me |

| | |
|---|---|
| Daily | Other, please specify |
| Less than once a semester/term | Other, please specify |
| Weekly | I purchased them online |
| Weekly | Other, please specify |
| Daily | They were given to me by a friend |
| Daily | I purchased them online |
| Daily | I purchased them online |
| Daily | Other, please specify |
| Once a semester/term | Other, please specify |
| Less than once a semester/term | They are prescribed for me |
| Less than once a semester/term | They were given to me by a friend |
| Once a semester/term | They were prescribed for somebody else |

| | |
|---|---|
| Daily | I purchased them online |
| Less than once a semester/term | I purchased them online |
| Less than once a semester/term | I purchased them online |
| Less than once a semester/term | They were given to me by a friend |
| Once a semester/term | I purchased them online |
| Daily | I purchased them online |
| Daily | They were given to me by a friend |
| Daily | I purchased them online |
| Daily | I purchased them online |
| Daily | I purchased them online |
| Daily | I purchased them online |
| Daily | I purchased them online |
| Daily | I purchased them online |
| Daily | I purchased them online |
| Daily | I purchased them online |
| Daily | I purchased them online |
| Daily | I purchased them online |
| Once a semester/term | I purchased them online |
| Once a semester/term | I purchased them online |
| Daily | I purchased them online |
| Daily | I purchased them online |
| Daily | I purchased them online |
| Daily | I purchased them online |
| Daily | They were given to me by a friend |
| Daily | I purchased them online |
| Once a semester/term | They were given to me by a friend |
| Daily | They were given to me by a friend |
| Daily | I purchased them online |

| | |
|---|---|
| Daily | |
| Daily | I purchased them online |
| Daily | I purchased them online |
| Daily | I purchased them online |
| Daily | They were given to me by a friend |
| Weekly | I purchased them online |
| Less than once a semester/term | They were given to me by a friend |
| Once a semester/term | I purchased them online |
| Once a semester/term | I purchased them online |
| Once a semester/term | They were given to me by a friend |
| Once a semester/term | I purchased them online |
| Once a semester/term | I purchased them online |
| Once a semester/term | I purchased them online |
| Weekly | They were given to me by a friend |
| Monthly | I purchased them online |
| Once a semester/term | I purchased them online |
| Less than once a semester/term | I purchased them online |

| | |
|---|---|
| Less than once a semester/term | I purchased them online |
| Once a semester/term | I purchased them online |
| Once a semester/term | I purchased them online |
| Once a semester/term | They were given to me by a friend |
| Monthly | They are prescribed for me |
| Monthly | I purchased them online |
| Monthly | I purchased them online |
| Once a semester/term | I purchased them online |
| Once a semester/term | I purchased them online |
| Once a semester/term | I purchased them online |

| | |
|---|---|
| Once a semester/term | They were given to me by a friend |
| Less than once a semester/term | They were prescribed for somebody else |
| Daily | I purchased them online |
| | Other, please specify |
| Daily | They are prescribed for me |
| Daily | I purchased them online |
| Weekly | They are prescribed for me |
| Monthly | Other, please specify |

| | |
|---|---|
| Less than once a semester/term | I purchased them online |
| Daily | They are prescribed for me |
| Less than once a semester/term | Other, please specify |
| Less than once a semester/term | |
| Monthly | They are prescribed for me |
| Less than once a semester/term | I purchased them online |

| | |
|---|---|
| Daily | Other, please specify |
| Monthly | They are prescribed for me |
| Daily | They are prescribed for me |
| Once a semester/term | I purchased them online |
| Less than once a semester/term | They are prescribed for me |
| Once a semester/term | I purchased them online |
| Less than once a semester/term | They are prescribed for me |
| Once a semester/term | I purchased them online |

| | |
|---|---|
| Once a semester/term | I purchased them online |

| | |
|---|---|
| Once a semester/term | I purchased them online |
| Less than once a semester/term | I purchased them online |
| Less than once a semester/term | I purchased them online |

| | |
|---|---|
| Less than once a semester/term | I purchased them online |
| Less than once a semester/term | I purchased them online |
| Once a semester/term | I purchased them online |
| Once a semester/term | I purchased them online |
| Less than once a semester/term | I purchased them online |
| Once a semester/term | They were given to me by a friend |
| Less than once a semester/term | I purchased them online |
| Once a semester/term | I purchased them online |
| Once a semester/term | I purchased them online |
| Once a semester/term | I purchased them online |
| Once a semester/term | I purchased them online |
| Less than once a semester/term | I purchased them online |
| Once a semester/term | I purchased them online |
| Less than once a semester/term | They were given to me by a friend |
| Less than once a semester/term | I purchased them online |

| | |
|---|---|
| Less than once a semester/term | They were given to me by a friend |
| Less than once a semester/term | They were given to me by a friend |
| Less than once a semester/term | I purchased them online |
| Once a semester/term | They were given to me by a friend |
| Once a semester/term | They were given to me by a friend |
| Weekly | I purchased them online |
| Weekly | I purchased them online |
| Weekly | I purchased them online |

| | |
|---|---|
| Once a semester/term | I purchased them online |
| Once a semester/term | They are prescribed for me |
| Once a semester/term | I purchased them online |
| Once a semester/term | They were given to me by a friend |
| Monthly | Other, please specify |
| Once a semester/term | I purchased them online |
| Weekly | Other, please specify |
| Once a semester/term | Other, please specify |
| Monthly | I purchased them online |
| Daily | Other, please specify |
| Weekly | Other, please specify |
| Once a semester/term | They were given to me by a friend |
| Once a semester/term | They were given to me by a friend |
| Once a semester/term | I purchased them online |
| Daily | They were given to me by a friend |
| Monthly | I purchased them online |
| Monthly | I purchased them online |
| Once a semester/term | I purchased them online |

| Q13_6_TEXT |
| --- |
| Where did you obtain thesedrugs/substances? - Other, please specify - Text |

Over counter

Bought it from supplement store

i bought them from the pharmacy

purchased from boots

ASDA, Morrisons, Tesco (it is not a drug) it is just almonds that I use for improving my memory.

not used

Not your business

Does caffeinated drinks count??

I taked from pharmacy or hospital

Pharmacy

Pharmacy

Pharmacy

Over the counter

Pharmacy

| Q14<br>How do you consider thecost of the drugs/ | Q15<br>Do you know anyone who usesthese (dr |
|---|---|
| Cheap | Yes |
| Fair | No |
| | No |
| Fair | Yes |
| Expensive | Yes |
| | No |
| Fair | No |
| | No |

| | |
|---|---|
| | No |
| Expensive | Yes |
| | No |
| | Yes |
| | No |
| Expensive | Yes |
| | No |
| Fair | No |
| Expensive | No |
| Fair | Yes |
| Fair | Yes |
| Fair | Yes |
| Fair | Yes |
| Fair | Yes |
| Fair | No |
| Expensive | Yes |
| Cheap | No |

| | |
|---|---|
| Expensive | Yes |
| Expensive | Yes |
| Expensive | No |
| Expensive | Yes |
| Fair | Yes |
| Fair | Yes |
| Expensive | Yes |
| Expensive | No |
| Expensive | No |
| Expensive | Yes |
| Expensive | No |
| Expensive | Yes |
| Expensive | No |
| Expensive | No |
| Expensive | Yes |
| Expensive | Yes |
| Expensive | No |
| Fair | Yes |
| Fair | Yes |
| Fair | Yes |
| Fair | Yes |
| Expensive | Yes |
| Expensive | Yes |
| Fair | Yes |
| Expensive | No |
| Expensive | Yes |
| Expensive | Yes |
| Expensive | Yes |

| | |
|---|---|
| Fair | Yes |
| Fair | Yes |
| Fair | Yes |
| Fair | Yes |
| Fair | Yes |
| Expensive | Yes |
| Fair | Yes |
| Expensive | Yes |
| Expensive | Yes |
| Fair | Yes |
| Expensive | Yes |
| Fair | Yes |
| Expensive | Yes |
| Fair | Yes |
| Expensive | Yes |
| Expensive | Yes |

| | |
|---|---|
| Expensive | Yes |
| Expensive | Yes |
| Expensive | Yes |
| Expensive | Yes |
| Expensive | Yes |
| Fair | Yes |
| Expensive | Yes |
| Fair | Yes |
| Expensive | Yes |
| Expensive | Yes |

| | |
|---|---|
| | No |
| Expensive | No |
| Fair | Yes |
| Expensive | No |
| Fair | Yes |
| | No |
| Very expensive | No |
| | No |
| Fair | No |
| | No |
| Very expensive | Yes |
| Expensive | No |
| | No |

| | |
|---|---|
| Fair | No |
| | No |
| | No |
| Cheap | No |
| | No |
| | No |
| Fair | Yes |
| | No |
| | Yes |
| Expensive | Yes |

| | |
|---|---|
| Fair | No |
| Fair | Yes |
| | No |
| | No |
| | Yes |
| | No |
| Fair | No |
| Expensive | Yes |
| | No |
| Fair | Yes |
| | No |
| Expensive | Yes |
| Fair | Yes |
| Expensive | Yes |

| | |
|---|---|
| Fair | Yes |
| | No |
| Expensive | Yes |
| Expensive | Yes |
| Expensive | Yes |

| | |
|---|---|
| | No |
| Fair | Yes |
| Expensive | Yes |
| Expensive | Yes |
| Expensive | Yes |
| Expensive | Yes |
| Expensive | Yes |
| Expensive | Yes |
| Expensive | Yes |
| Expensive | Yes |
| Expensive | Yes |
| | No |
| | No |
| Expensive | Yes |
| Expensive | Yes |
| | No |
| Fair | Yes |
| Fair | Yes |
| Fair | Yes |

| | |
|---|---|
| Fair | Yes |
| Fair | Yes |
| Fair | Yes |
| Fair | Yes |
| Fair | Yes |
| Fair | Yes |
| Fair | Yes |
| Fair | Yes |
| | No |

| | |
|---|---|
| Fair | Yes |
| Expensive | No |
| Expensive | Yes |
| Fair | Yes |
| | |
| Fair | Yes |
| Fair | Yes |
| Expensive | Yes |
| Expensive | Yes |
| Fair | Yes |
| Fair | No |
| | |
| Fair | Yes |
| Fair | Yes |
| Expensive | Yes |
| Fair | Yes |
| Fair | Yes |
| Expensive | Yes |
| Fair | Yes |
| Fair | Yes |

| Q16 | Q17 |
|---|---|
| Where have you heard aboutthese dr | Will you recommend these drugs to others? |
| Internet | No |
| Internet | No |
| Friends | Yes |
| Friends | Yes |
| Other | No |
| Other | No |
| Other | |

| | |
|---|---|
| | No |
| Scientific literature | Yes |
| Other | Yes |
| Scientific literature | No |
| Social media | No |
| Scientific literature | Yes |
| Family | No |
| | No |
| Social media | No |
| Internet | No |
| Friends | Yes |
| | No |
| Friends | No |
| Family | No |
| Friends | No |
| Friends | No |
| Scientific literature | Yes |

| | |
|---|---|
| Friends | Yes |
| Friends | No |
| Friends | Yes |
| Friends | No |
| Friends | Yes |
| Friends | Yes |
| Friends | No |
| Social media | No |
| Social media | Yes |
| Friends | No |
| Social media | No |
| Friends | No |
| Friends | |
| Friends | No |
| Friends | No |
| Friends | No |
| Friends | No |
| Friends | No |
| Friends | No |
| Friends | No |
| Social media | No |
| Friends | No |
| Friends | No |
| Friends | No |
| Social media | No |
| Friends | No |
| Friends | No |
| Friends | No |

| | |
|---|---|
| Social media | No |
| Social media | No |
| Friends | No |
| | |
| Internet | No |
| | |
| Friends | No |
| | |
| | |
| Internet | No |
| Friends | No |
| | |
| Social media | No |
| Social media | No |
| | |
| | |
| Friends | Yes |
| | |
| Friends | No |
| Friends | Yes |
| | |
| Friends | Yes |
| Friends | Yes |
| Internet | No |
| | |
| Internet | Yes |
| Internet | No |

| | |
|---|---|
| Friends | No |
| Friends | No |
| Internet | No |
| Friends | No |
| Social media | No |
| Internet | No |
| Internet | No |
| Internet | No |
| Internet | No |
| Internet | No |

| | |
|---|---|
| Internet | No |
| Internet | No |
| Friends | No |
| Internet | No |
| Internet | No |
| | No |
| Other | No |
| Scientific literature | No |
| Social media | Yes |
| Other | No |
| | No |

| | |
|---|---|
| Internet | No |
| Internet | No |
| Other | No |
| Family | Yes |
| | No |
| Scientific literature | No |
| Scientific literature | Yes |
| Other | No |
| Family | No |
| Internet | No |

| | |
|---|---|
| Other | No |
| Family | Yes |
| Scientific literature | No |
| | No |
| Internet | |
| Other | No |
| Internet | Yes |
| Social media | No |
| Other | Yes |
| | No |
| Friends | No |
| Family | Yes |
| Family | Yes |

| | |
|---|---|
| Social media | No |
| Internet | No |
| Scientific literature | |
| Internet | No |
| Friends | No |
| Friends | Yes |

| | |
|---|---|
| | No |
| Friends | No |
| Friends | No |
| Friends | No |
| Internet | Yes |
| Internet | Yes |
| Friends | No |
| Internet | Yes |
| Internet | Yes |
| Internet | No |
| Internet | No |
| Social media | No |
| Social media | No |
| Internet | Yes |
| Internet | No |
| Social media | No |
| Internet | No |
| Internet | No |
| Internet | No |

| | |
|---|---|
| Friends | No |
| Friends | No |
| Internet | Yes |
| Friends | Yes |
| Friends | Yes |
| Internet | Yes |
| Internet | Yes |
| Internet | Yes |

| | |
|---|---|
| Internet | Yes |
| Other | No |
| Friends | Yes |
| Friends | Yes |
| | |
| Friends | Yes |
| Friends | Yes |
| Family | Yes |
| Friends | Yes |
| Friends | Yes |
| Scientific literature | No |
| | |
| Friends | Yes |
| Friends | Yes |
| Friends | Yes |
| Friends | Yes |
| Scientific literature | Yes |
| Friends | Yes |
| Friends | Yes |
| Friends | Yes |

QID52
Thank you for participating to the study. - Selected Choice

(If you want to take part of winning the amazon voucher please leave your email on the text below to be contacted)

(If you want to take part of winning the amazon voucher please leave your email on the text below to be contacted)

(If you want to take part of winning the amazon voucher please leave your email on the text below to be contacted)

(If you want to take part of winning the amazon voucher please leave your email on the text below to be contacted)

(If you want to take part of winning the amazon voucher please leave your email on the text below to be contacted)

(If you want to take part of winning the amazon voucher please leave your email on the text below to be contacted)

(If you want to take part of winning the amazon voucher please leave your email on the text below to be contacted)

(If you want to take part of winning the amazon voucher please leave your email on the text below to be contacted)

(If you want to take part of winning the amazon voucher please leave your email on the text below to be contacted)

(If you want to take part of winning the amazon voucher please leave your email on the text below to be contacted)

(If you want to take part of winning the amazon voucher please leave your email on the text below to be contacted)

(If you want to take part of winning the amazon voucher please leave your email on the text below to be contacted)

(If you want to take part of winning the amazon voucher please leave your email on the text below to be contacted)

(If you want to take part of winning the amazon voucher please leave your email on the text below to be contacted)

(If you want to take part of winning the amazon voucher please leave your email on the text below to be contacted)

(If you want to take part of winning the amazon voucher please leave your email on the text below to be contacted)
(If you want to take part of winning the amazon voucher please leave your email on the text below to be contacted)

(If you want to take part of winning the amazon voucher please leave your email on the text below to be contacted)
(If you want to take part of winning the amazon voucher please leave your email on the text below to be contacted)

(If you want to take part of winning the amazon voucher please leave your email on the text below to be contacted)
(If you want to take part of winning the amazon voucher please leave your email on the text below to be contacted)
(If you want to take part of winning the amazon voucher please leave your email on the text below to be contacted)
(If you want to take part of winning the amazon voucher please leave your email on the text below to be contacted)
(If you want to take part of winning the amazon voucher please leave your email on the text below to be contacted)

(If you want to take part of winning the amazon voucher please leave your email on the text below to be contacted)

(If you want to take part of winning the amazon voucher please leave your email on the text below to be contacted)

(If you want to take part of winning the amazon voucher please leave your email on the text below to be contacted)

(If you want to take part of winning the amazon voucher please leave your email on the text below to be contacted)

(If you want to take part of winning the amazon voucher please leave your email on the text below to be contacted)
(If you want to take part of winning the amazon voucher please leave your email on the text below to be contacted)

(If you want to take part of winning the amazon voucher please leave your email on the text below to be contacted)

(If you want to take part of winning the amazon voucher please leave your email on the text below to be contacted)
(If you want to take part of winning the amazon voucher please leave your email on the text below to be contacted)
(If you want to take part of winning the amazon voucher please leave your email on the text below to be contacted)
(If you want to take part of winning the amazon voucher please leave your email on the text below to be contacted)
(If you want to take part of winning the amazon voucher please leave your email on the text below to be contacted)

(If you want to take part of winning the amazon voucher please leave your email on the text below to be contacted)
(If you want to take part of winning the amazon voucher please leave your email on the text below to be contacted)
(If you want to take part of winning the amazon voucher please leave your email on the text below to be contacted)
(If you want to take part of winning the amazon voucher please leave your email on the text below to be contacted)
(If you want to take part of winning the amazon voucher please leave your email on the text below to be contacted)

(If you want to take part of winning the amazon voucher please leave your email on the text below to be contacted)

(If you want to take part of winning the amazon voucher please leave your email on the text below to be contacted)
(If you want to take part of winning the amazon voucher please leave your email on the text below to be contacted)
(If you want to take part of winning the amazon voucher please leave your email on the text below to be contacted)

(If you want to take part of winning the amazon voucher please leave your email on the text below to be contacted)

(If you want to take part of winning the amazon voucher please leave your email on the text below to be contacted)
(If you want to take part of winning the amazon voucher please leave your email on the text below to be contacted)

(If you want to take part of winning the amazon voucher please leave your email on the text below to be contacted)

(If you want to take part of winning the amazon voucher please leave your email on the text below to be contacted)

(If you want to take part of winning the amazon voucher please leave your email on the text below to be contacted)
(If you want to take part of winning the amazon voucher please leave your email on the text below to be contacted)

(If you want to take part of winning the amazon voucher please leave your email on the text below to be contacted)
(If you want to take part of winning the amazon voucher please leave your email on the text below to be contacted)
(If you want to take part of winning the amazon voucher please leave your email on the text below to be contacted)
(If you want to take part of winning the amazon voucher please leave your email on the text below to be contacted)

(If you want to take part of winning the amazon voucher please leave your email on the text below to be contacted)
(If you want to take part of winning the amazon voucher please leave your email on the text below to be contacted)
(If you want to take part of winning the amazon voucher please leave your email on the text below to be contacted)
(If you want to take part of winning the amazon voucher please leave your email on the text below to be contacted)
(If you want to take part of winning the amazon voucher please leave your email on the text below to be contacted)

(If you want to take part of winning the amazon voucher please leave your email on the text below to be contacted)
(If you want to take part of winning the amazon voucher please leave your email on the text below to be contacted)

(If you want to take part of winning the amazon voucher please leave your email on the text below to be contacted)

(If you want to take part of winning the amazon voucher please leave your email on the text below to be contacted)
(If you want to take part of winning the amazon voucher please leave your email on the text below to be contacted)

(If you want to take part of winning the amazon voucher please leave your email on the text below to be contacted)

(If you want to take part of winning the amazon voucher please leave your email on the text below to be contacted)

(If you want to take part of winning the amazon voucher please leave your email on the text below to be contacted)

(If you want to take part of winning the amazon voucher please leave your email on the text below to be contacted)
(If you want to take part of winning the amazon voucher please leave your email on the text below to be contacted)
(If you want to take part of winning the amazon voucher please leave your email on the text below to be contacted)

(If you want to take part of winning the amazon voucher please leave your email on the text below to be contacted)
(If you want to take part of winning the amazon voucher please leave your email on the text below to be contacted)

(If you want to take part of winning the amazon voucher please leave your email on the text below to be contacted)
(If you want to take part of winning the amazon voucher please leave your email on the text below to be contacted)

(If you want to take part of winning the amazon voucher please leave your email on the text below to be contacted)

(If you want to take part of winning the amazon voucher please leave your email on the text below to be contacted)
(If you want to take part of winning the amazon voucher please leave your email on the text below to be contacted)

(If you want to take part of winning the amazon voucher please leave your email on the text below to be contacted)

(If you want to take part of winning the amazon voucher please leave your email on the text below to be contacted)

(If you want to take part of winning the amazon voucher please leave your email on the text below to be contacted)
(If you want to take part of winning the amazon voucher please leave your email on the text below to be contacted)

(If you want to take part of winning the amazon voucher please leave your email on the text below to be contacted)
(If you want to take part of winning the amazon voucher please leave your email on the text below to be contacted)

(If you want to take part of winning the amazon voucher please leave your email on the text below to be contacted)

(If you want to take part of winning the amazon voucher please leave your email on the text below to be contacted)
(If you want to take part of winning the amazon voucher please leave your email on the text below to be contacted)
(If you want to take part of winning the amazon voucher please leave your email on the text below to be contacted)
(If you want to take part of winning the amazon voucher please leave your email on the text below to be contacted)
(If you want to take part of winning the amazon voucher please leave your email on the text below to be contacted)
(If you want to take part of winning the amazon voucher please leave your email on the text below to be contacted)

(If you want to take part of winning the amazon voucher please leave your email on the text below to be contacted)
(If you want to take part of winning the amazon voucher please leave your email on the text below to be contacted)
(If you want to take part of winning the amazon voucher please leave your email on the text below to be contacted)
(If you want to take part of winning the amazon voucher please leave your email on the text below to be contacted)
(If you want to take part of winning the amazon voucher please leave your email on the text below to be contacted)

(If you want to take part of winning the amazon voucher please leave your email on the text below to be contacted)

(If you want to take part of winning the amazon voucher please leave your email on the text below to be contacted)

(If you want to take part of winning the amazon voucher please leave your email on the text below to be contacted)
(If you want to take part of winning the amazon voucher please leave your email on the text below to be contacted)
(If you want to take part of winning the amazon voucher please leave your email on the text below to be contacted)

(If you want to take part of winning the amazon voucher please leave your email on the text below to be contacted)
(If you want to take part of winning the amazon voucher please leave your email on the text below to be contacted)

(If you want to take part of winning the amazon voucher please leave your email on the text below to be contacted)

(If you want to take part of winning the amazon voucher please leave your email on the text below to be contacted)

(If you want to take part of winning the amazon voucher please leave your email on the text below to be contacted)
(If you want to take part of winning the amazon voucher please leave your email on the text below to be contacted)

(If you want to take part of winning the amazon voucher please leave your email on the text below to be contacted)

(If you want to take part of winning the amazon voucher please leave your email on the text below to be contacted)

(If you want to take part of winning the amazon voucher please leave your email on the text below to be contacted)
(If you want to take part of winning the amazon voucher please leave your email on the text below to be contacted)

(If you want to take part of winning the amazon voucher please leave your email on the text below to be contacted)

(If you want to take part of winning the amazon voucher please leave your email on the text below to be contacted)

(If you want to take part of winning the amazon voucher please leave your email on the text below to be contacted)
(If you want to take part of winning the amazon voucher please leave your email on the text below to be contacted)

(If you want to take part of winning the amazon voucher please leave your email on the text below to be contacted)
(If you want to take part of winning the amazon voucher please leave your email on the text below to be contacted)
(If you want to take part of winning the amazon voucher please leave your email on the text below to be contacted)

(If you want to take part of winning the amazon voucher please leave your email on the text below to be contacted)
(If you want to take part of winning the amazon voucher please leave your email on the text below to be contacted)

(If you want to take part of winning the amazon voucher please leave your email on the text below to be contacted)

(If you want to take part of winning the amazon voucher please leave your email on the text below to be contacted)
(If you want to take part of winning the amazon voucher please leave your email on the text below to be contacted)
(If you want to take part of winning the amazon voucher please leave your email on the text below to be contacted)
(If you want to take part of winning the amazon voucher please leave your email on the text below to be contacted)
(If you want to take part of winning the amazon voucher please leave your email on the text below to be contacted)
(If you want to take part of winning the amazon voucher please leave your email on the text below to be contacted)
(If you want to take part of winning the amazon voucher please leave your email on the text below to be contacted)

(If you want to take part of winning the amazon voucher please leave your email on the text below to be contacted)

(If you want to take part of winning the amazon voucher please leave your email on the text below to be contacted)
(If you want to take part of winning the amazon voucher please leave your email on the text below to be contacted)

(If you want to take part of winning the amazon voucher please leave your email on the text below to be contacted)
(If you want to take part of winning the amazon voucher please leave your email on the text below to be contacted)
(If you want to take part of winning the amazon voucher please leave your email on the text below to be contacted)

(If you want to take part of winning the amazon voucher please leave your email on the text below to be contacted)
(If you want to take part of winning the amazon voucher please leave your email on the text below to be contacted)

(If you want to take part of winning the amazon voucher please leave your email on the text below to be contacted)

(If you want to take part of winning the amazon voucher please leave your email on the text below to be contacted)

(If you want to take part of winning the amazon voucher please leave your email on the text below to be contacted)

(If you want to take part of winning the amazon voucher please leave your email on the text below to be contacted)
(If you want to take part of winning the amazon voucher please leave your email on the text below to be contacted)

(If you want to take part of winning the amazon voucher please leave your email on the text below to be contacted)
(If you want to take part of winning the amazon voucher please leave your email on the text below to be contacted)
(If you want to take part of winning the amazon voucher please leave your email on the text below to be contacted)

(If you want to take part of winning the amazon voucher please leave your email on the text below to be contacted)

(If you want to take part of winning the amazon voucher please leave your email on the text below to be contacted)
(If you want to take part of winning the amazon voucher please leave your email on the text below to be contacted)

(If you want to take part of winning the amazon voucher please leave your email on the text below to be contacted)
(If you want to take part of winning the amazon voucher please leave your email on the text below to be contacted)

(If you want to take part of winning the amazon voucher please leave your email on the text below to be contacted)

(If you want to take part of winning the amazon voucher please leave your email on the text below to be contacted)
(If you want to take part of winning the amazon voucher please leave your email on the text below to be contacted)

(If you want to take part of winning the amazon voucher please leave your email on the text below to be contacted)

(If you want to take part of winning the amazon voucher please leave your email on the text below to be contacted)

(If you want to take part of winning the amazon voucher please leave your email on the text below to be contacted)
(If you want to take part of winning the amazon voucher please leave your email on the text below to be contacted)

(If you want to take part of winning the amazon voucher please leave your email on the text below to be contacted)
(If you want to take part of winning the amazon voucher please leave your email on the text below to be contacted)

(If you want to take part of winning the amazon voucher please leave your email on the text below to be contacted)

(If you want to take part of winning the amazon voucher please leave your email on the text below to be contacted)
(If you want to take part of winning the amazon voucher please leave your email on the text below to be contacted)

(If you want to take part of winning the amazon voucher please leave your email on the text below to be contacted)

(If you want to take part of winning the amazon voucher please leave your email on the text below to be contacted)

| |
|---|
| QID52_1_TEXT |
| Thank you for participating to the study. - (If you want to take part of winning the amazon voucher please leave your er |

2017bm12@mygmu.ac.ae
2019pt07

seeniasusan@gmail.com
2017pcs02@mygmu.ac.ae
2019pt07@mygmu.ac.ae
saarahbuddha10@gmail.com

shreyarao935@gmail.com
saarahbuddha10@gmail.com
2017d051@mygmu.ac.ae

Majedalghoul@gmail.com

mabelmm98@gmail.com
vaibhavahuja99@gmail.com

aliraxstar900@gmail.com

2015PH06@MYGMU.AC.AE
Themonumentoflife5@gmail.com

daniyah.humair2200@gmail.com

msh1611@gmail.com

Huda.obaid.92@hotmail.com
khadeeja.hala@hotmail.co.uk
2018d054@gmail.com

fathimarahasal@gmail.com

remona1998@gmail.com
aimsorathia@gmail.com

fathima552000@gmail.com

2015m048@gmu.ac.ae
kfariya94@gmail.com

2019m027@mygmu.ac.ae

2015M041@mygmu.ac.ae

rishda_basheer190@hotmail.com
nishatmahveen32@gmail.com

farahismail4997@gmail.com
Waleed_thafer@outlook.com

Najiaziasa@gmail.com

Nayerak113@gmail.com

asoomah01@gmail.com
mizbah_zuber@yahoo.com
christyvinod99@gmail.com
adhna007@gmail.com

minumeer@gmail.com
athira6589@gmail.com
2015m011@mygmu.ac.ae
amna3024@gmail.com
hifsa_randhawa@hotmail.com.au

Sonia_bader@yahoo.com

nahal_gh1@yahoo.com

sharrol456@gmail.com
2019ph19@mygmu.ac.ae
amiraazraq@gmail.com

f0y0a0a0k@gmail.com
mohammedr811@yahoo.com

sadiq.jafrin@gmail.com

2017ph36@mygmu.ac.ae

2018pcs01@mygmu.ac.ae

Fatimamasood99@hotmail.com

2019pt03@mygmu.ac.ae

amirajunaid01@gmail.com

sundus.asim@me.com

marawe14@gmail.com
burhan5253@gmail.com

2019ns16@mygmu.ac.ae
Aliasbali@hotmail.com
basimw14@gmail.com

examsfor12@gmail.com
Sheikha.ahmed.gd@gmail.com

n-ghanem@hotmail.com

2017m032@mygmu.ac.ae
abizerfiroz@gmail.com
alia.zubair07@gmail.com
shreyadhanak@gmail.com
2017m030@mygmu.ac.ae
mariyamda1@gmail.com
jahmd5594@gmail.com

2018ph08@mygmu.ac.ae

tadiwafaranisi1@gmail.com
Alaaa6806@gmail.com

2016m012@mygmu.ac.ae
alenjoy126@gmail.com
raheesahere@gmail.com

2017pcs17@mygmu.ac.ae

Safa.blossom@yahoo.com

2018pcs28@mygmu.ac.ae

aroojtm@hotmail.com

2017m047@mygmu.ac.ae
masudmohammed999@gmail.com

Hamdaalsabouri@gmail.com
zainababdullah2411@gmail.com

nusrath324@gmail.com

2015ph01@mygmu.ac.ae
fateemandanusa@yahoo.com

zainabashraff2@gmail.com

ajmalsainul@gmail.com

2017m021@mygmu.ac.ae
2017m036@mygmu.ac.ae

Anesthesia983@gmail.com

aymannajafi786@gmail.com

2017m068@mygmu.ac.ae

sannashaji@gmail.com

a.saqaaf@gmail.com

Bamigbade.evie@gmail.com
