# Supplementary material for: Assessing prevalence, knowledge and use of cognitive enhancers among university students in the United Arab Emirates: A quantitative study
Source: PLoS One. 2022 Jan 26;17(1):e0262704. doi: 10.1371/journal.pone.0262704 (PMC8791475; doi:10.1371/journal.pone.0262704)
Supplement: S1 Appendix — (DOCX) [file pone.0262704.s003.docx]

### **S1 Appendix**

### **Survey Questionnaire**

*This is a survey to explore your knowledge and use of CE among the university students in the UK and UAE. Kindly fill in this survey to the best of your knowledge and understanding. This survey will take approximately five minutes to complete. The information provided, will be strictly kept confidential and anonymous.*

**‘Please tick as appropriate’**

1. Gender:

- Male
- Female
- Prefer not to say

1. Age:

- 18 to 25
- 26 to 35
- 36 to 45
- 46 to 55
- 56 to 65
- 66+

1. Have you ever used psychostimulants drugs/ nootropics for memory/ concentration/ learning ability enhancement?

- Yes
- No (*Thank you for participating and ‘your survey is completed’!*)
- Do not know/unsure

1. *If ‘Yes’ which one? (select all that apply)*

- Modafinil
- Adderall/amphetamines mixture
- Ritalin/Methylphenidate
- Alpha Brain/vitamin B6
- Super strength caffeine pills
- Guarana
- Piracetam
- Vinpocetine
- Cobalamin/vitamin B12
- Other drug or substance used

1. Nationality:

- UK
- EU
- USA
- UAE
- Arab nationality
- Other, (please specify)

1. What degree are you studying?

- Medicine
- Pharmacy
- Dentistry
- Nursing
- Engineering
- Other, (please specify)

1. Year of Education

- First year
- Second year
- Third year
- Fourth year
- Fifth year
- Sixth year

1. Please specify the reason (s) for use? *(select all that apply)*

- To improve concentration
- To improve your memory
- To increase alertness
- To improve academic performance
- Other, please specify……………………………………………………….

1. Do you get positive effects from the use of these drugs/substances (e.g. euphoria, alertness)?

- Yes. If yes, please, specify………………………………………………..
- No

1. When was the time of your most recent consumption?

- During exam weeks
- During course work deadlines
- During studying
- Daily basis

1. How long did you take the CE for?

- Less than 1 month
- 1 month but less than 6 months
- 6 months but less than 1 year
- 1 year but less than 2 years
- 2 years and more

1. Approximately, how frequently did you use the drugs/substances?

- Daily
- Weekly
- Monthly
- Once a semester/term
- Less than once a semester/term

1. Where did you obtain these drugs/substances?

- They are prescribed for me
- They were given to me by a friend
- They were given to me by a stranger
- They were prescribed for somebody else
- I purchased them online
- Other, please specify

1. How do you consider the cost of the drugs/ substances are?

- Very expensive
- Expensive
- Fair
- Cheap
- Very cheap

1. Do you know anyone who uses these (drugs/substances)?

- Yes
- No

1. Where have you heard about these drugs/substances?

- Social media
- Scientific literature
- Internet
- Friends
- Family
- Other

1. Will you recommend these drugs to others?

- Yes
- No

**Thank you for your time**
